# Benzoylation of Tetrols: A Comparison of Regioselectivity Patterns for *O-* and *S-*Glycosides of d-Galactose

**DOI:** 10.1021/acs.joc.4c01508

**Published:** 2024-09-12

**Authors:** Jack Porter, Jacob Roberts, Gavin J. Miller

**Affiliations:** Centre for Glycoscience and School of Chemical and Physical Sciences, Keele University, Keele, Staffordshire ST5 5BG, U.K.

## Abstract

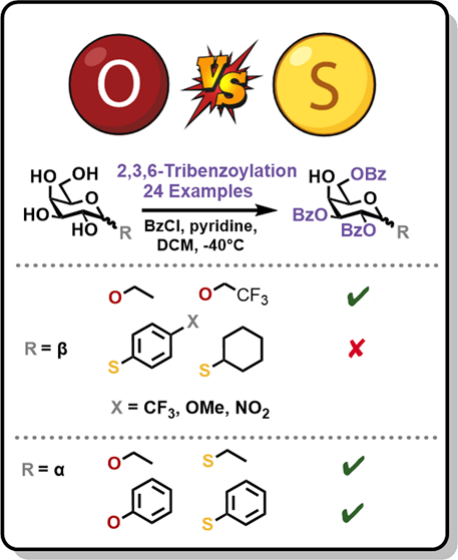

Efficient protecting group strategies are important for glycan synthesis and represent a unique synthetic challenge in differentiating sugar ring hydroxyl groups. Direct methods to enable regioselective protecting group installation are thus desirable. Herein, we explore a one-step regioselective benzoylation to deliver 2,3,6-protected d-galactose building blocks from tetrols across a variety of α- and β-, *O*- and *S*-glycoside substrates. We focus on benzoyl chloride as the esterifying reagent and a reaction temperature of −40 °C to screen the regioselectivity outcome for twenty-two different glycosides, based on isolated yields. Using this methodology, we demonstrate the capability for α-linked aryl and alkyl glycosides (*O*- and *S*- d-galactosides, d-galactosamines, and l-fucose), delivering consistent isolated yields (>65%) for 2,3,6-benzoylated products. We extend to explore β-linked systems, where the observed regioselectivity is not paralleled. We posit that both steric and electronic factors from the anomeric substituent contribute to modulating the reactivity competition between 2-OH and 4-OH, enabling the formation of regioisomeric mixtures. However, a certain balance of these factors within the aglycon can deliver 2,3,6-regioselectivity, notably for *β-O*-Et and *β-O*-CH_2_CF_3_ glycosides. The methodology contributes toward understanding the peculiarities of regioselective carbohydrate-protecting group installation, exploring the importance of the anomeric substituent upon ring hydroxyl group reactivity.

## Introduction

**Figure 1:**
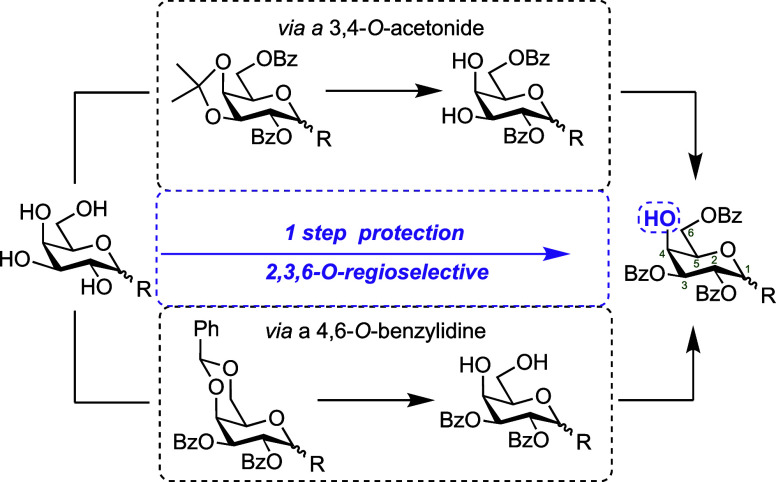
Common strategies to complete regioselective 2,3,6-*O*-benzoate installation in d-galactopyranosides. Remaining C4-hydroxyl is highlighted in purple. R = α- or β- *O*- or *S*-glycoside, Bz = benzoate.

Protecting group strategy is a cornerstone of modern carbohydrate synthesis and represents a unique synthetic challenge compared to the relative requirements for amino acids. Reactivity differences between constituent ring hydroxyl groups convey both difficulty and opportunity in enabling the regioselective installation of appropriate protecting groups. Successful regioselective hydroxyl group protection expedites efficient access to valuable building blocks, for further modification and incorporation into complex glycan or oligosaccharide targets, i.e., through blocking the positions not required in subsequent glycosylation or functionalization reactions. Direct methods to complete regioselective protection are preferred as they reduce the number of synthetic steps and thus generally have an improved atom economy.^1^

d-Galactopyranose, the C4-epimer of d-glucopyranose, is an essential monosaccharide and key component within related glycoconjugates, e.g., occupying the semiterminal reducing position of several *N*-linked glycans and as one-half of the disaccharide repeating unit within the glycosaminoglycan keratan sulfate. Relatedly, as part of a wider program concerning the chemical synthesis of heteroatom-modified glycan targets,^2−5^ we required a robust, early-stage entry to 2,3,6-protected d-galactose building blocks (Figure 1). Upon consulting the literature we noted that many of the available examples of such materials utilized synthetic strategies incorporating 4,6-*O*-acetal or 3,4-*O*-ketal protection.^6−14^ While effective, such approaches typically require up to four steps to deliver appropriate 2,3,6-protected materials and can also require tin-containing reagents, presenting unwanted toxicity considerations.^15,16^ Alternatively, low-temperature, one-step benzoylation protocols exploiting the reactivity differences of secondary alcohol groups around the d-galactopyranose ring are possible (Figure 1).

Hydroxyl group reactivity patterns to accomplish one-step esterification of simple monosaccharide *O-*glycosides were proposed in the 1960s.^17−19^ For d-galactose, the axial 4-OH is the least reactive of the three secondary hydroxyl group ring positions, opening up a prospect for regioselective protection. Since then further examples have evidenced the utility of this approach toward 2,3,6-*O*-benzoate installation in d-galactopyranosides,^20−22^ including for the preparation of methyl 2,3,6-*O*-benzoylated α-d-galactose and 1,2,3-*O*-benzoylated 6-deoxy-α-l-galactopyranose.^23^ Notable also is a variation in the yields reported and oftentimes a need for complex regioisomer separations (from over- and under benzoylated side products).^20,22,24−27^ Relatedly, a regioselective d-galactose protection methodology was developed using BzCN and DMAP at low temperature (−78 °C), to effect 4-OH acylation from the corresponding 3,4-diol.^28^ The selectivity observed was attributed to a “cyanide effect”, and this methodology could be extended to a one-pot protection of tetrol starting materials,^29^ and regioselective benzoylation of 2,3-*O*-unprotected α-galactopyranosides.^30^ Given this context, we also noted that the aforementioned examples concerned mostly α-linked substrates for protection of *O*-glycosides of d-galactose and that this afforded an opportunity for exploration of β-anomeric stereochemistry alongside synthetically malleable *S*-glycosides.

## Results and Discussion

To investigate the substrate scope for regioselective 2,3,6-tri-*O*-benzoylation, we synthesized a range of appropriate d-galactopyranosides (see the Supporting Information for details). These materials were subjected to benzoylation, and the results from this are outlined in Table 1. Conditions were selected with the aim of reducing the amount of per-esterified byproduct, as had been observed to be problematic previously.^22,25^ Therefore, the equivalents of benzoyl chloride were restricted to 3.1, and a reaction temperature of −40 °C was selected.

**Table 1:**
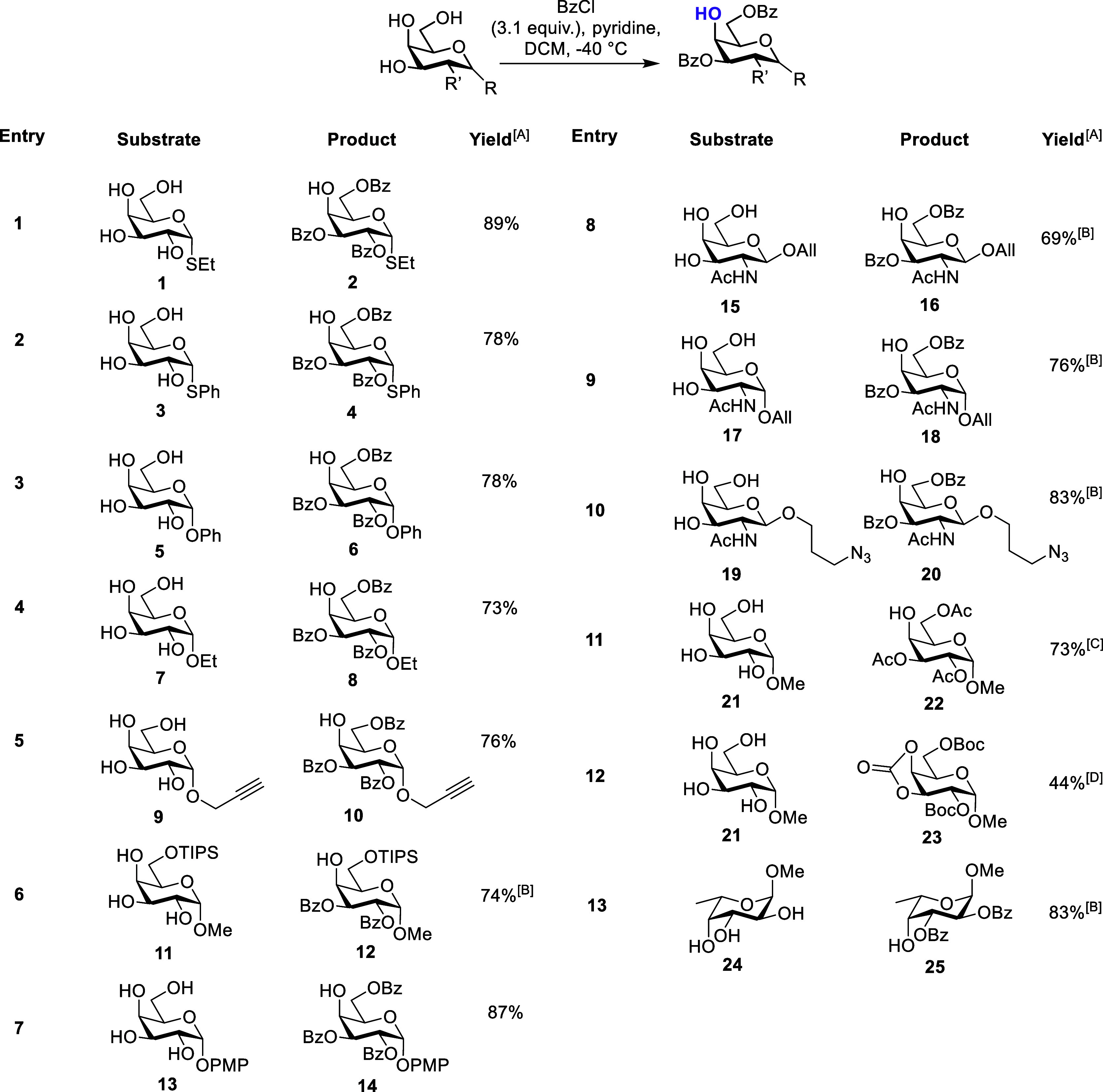
Initial Substrate Scope for Regioselective Benzoylation

A Isolated yield following column chromatography.

B 2.1 equiv; BzCl used.

C AcCl used in place of BzCl.

D Boc_2_O used in place of BzCl; R = anomeric group, R’ = C2 substituent.

We first chose α-SEt substrate **1** and after 30 min, reaction completion was indicated by TLC, revealing one major new spot. Material isolation and characterization by NMR showed this to be the desired product **2** (89%, Table 1, entry 1). HMBC analysis confirmed benzoyl group location through carbonyl to proton cross-peaks [(δ_H-2_ = 5.88 ppm to δ_C_ = 165.7 ppm), (δ_H-3_ = 5.64 ppm to δ_C_ = 165.6 ppm) and (δ_H-6a/b_ = 4.70 and 4.57 ppm to δ_C_ = 166.5 ppm)]. Encouraged by this result, α-SPh thioglycoside **3** was screened next (Table 1, entry 2). The reaction proceeded in a similar fashion, delivering 2,3,6-tri-*O*-benzoate **4** in 78% yield. Following these results with *S*-glycosides, we evaluated a comparative pair of *O*-glycosides (Table 1, entries 3–4). For α-OPh glycoside **5**, after 1 h of reaction at −40 °C, TLC revealed a single new spot. Purification and NMR analysis delivered 2,3,6-tri-*O*-benzoylated galactoside **6** in 78% yield. For α-OEt galactopyranoside **7**, the desired glycoside **8** was delivered in 73% yield. To explore functional anomeric components, we screened d-galactoside **9** possessing an α-anomeric propargyl alkyne, isolating the desired 2,3,6-tri-*O*-benzoate **10** in 76% yield (Table 1, entry 5). A 6-*O*-triisopropylsily protected methyl glycoside **11** yielded the desired product **12** in 74% yield and an α-*O*-*para*-methoxyphenyl derivative **14** was furnished in 87% yield from **13** (Table 1, entries 6–7). Based on these initial results, we extended to d-galactosamine derivatives, synthesizing d-GalNAc derivatives **15** and **17** bearing an anomeric allyl group. When screened, low-temperature benzoylation (now employing 2.1 equiv of BzCl) successfully delivered the respective 3,6-di-*O-*benzoates **16** and **18** in consistent yields (69 and 76% respectively, Table 1, entries 8–9) across both α- and β-anomeric substituents. Furthermore, β-azidopropyl derivative **19** furnished the corresponding 4,6-di-*O*-benzoate **20** in 83% yield (Table 1, entry 10).

To explore scope for compatible protecting groups beyond benzoyl, a range of additional electrophilic reagents were examined with galactose methyl glycoside **21** as substrate. Acetyl chloride successfully delivered 2,3,6-tri-*O*-acetate **22** in 73% yield (Table 1, entry 11). Switching to the bulkier electrophile pivaloyl chloride saw no conversion at −40 °C, aligning with previous results indicating the need for excess reagent, elevated temperature, and extended reaction time for bulky ester and silicon (TBS) groups.^31,32^ Investigating carbonates, using 3.1 equiv of Boc_2_O at −40 °C saw no change by TLC. Raising the temperature to ambient, followed by the addition of three further equivalents of the anhydride saw conversion to a new spot. Upon isolation of this material, we observed the incorporation of two ^*t*^Bu groups by ^1^H NMR, but also three carbonyl environments by ^13^C NMR. Following further HMBC and HRMS analyses we here tentatively assign this product as 2,6-bis-*O*-Boc protected-3,4-cyclic carbonate **23**, formed in 44% yield, (Table 1, entry 12). It is possible that the desired 2,3,6-tri-*O*-Boc material forms and cyclization from O4 then delivers the *cis*-five membered carbonate **23**. However, a 2,3-bis-*O*-Boc protected-4,6-cyclic carbonate may also form; similar 4,6- and 2,3-cyclic carbonates have been observed for glucose methyl glycosides.^33^

**Figure 2:**
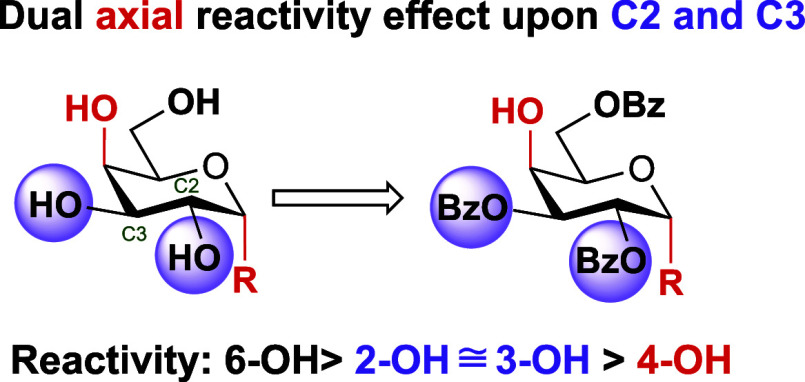
A dual axial effect for regioselective benzoylation within α-d-galactopyranosides. R = *O*- or *S*-glycoside.

The results from Table 1 align with an alkoxy group mediated diol effect,^34^ causing equatorial *trans* diols (C2/C3-OH within d-galactose) with a vicinal alkoxy group to react with preference at the hydroxyl adjacent to an axial substituent (Figure 2). For the examples in Table 1, this promotes the preferential reaction of the equatorial C2- and C3-OH positions over C4-OH, delivering 2,3,6-tri-*O*-benzoylated products in one step and in good yields for the range of α-linked *O-* and *S*-glycosides explored. In the case of d-GalNAc derivatives, the competing equatorial C2-OH is removed. Finally, and considering the results for d-galactosides, we selected l-fucose methyl glycoside (also possessing equatorial *trans* diols with vicinal axial alkoxy/hydroxyl groups). As expected, 2,3-di-*O*-benzoate **25** was isolated in 83% yield (Table 1, entry 13).

### Exploring the Influence of Anomeric Stereochemistry Upon Regioselectivity

β-Configured glycosyl donors are often selected during glycan synthesis owing to their increased reactivity when compared to their α-counterparts.^35^ Widespread use of protecting groups capable of anchimeric assistance during glycosylation further contributes to the prevalence of β-configured d-galactose donors.^36−39^ With these points in mind, we returned to thioglycosides, synthesizing β-linked systems **26** and **31** (comparable to **1** and **3**) and exposing them to benzoylation. Starting with β-SPh **26** (Table 2, entry 1) a regioisomeric mixture was obtained in 72% overall yield and with three separate spots visible by TLC. Upon separation and identification of these materials, the lowest *R*_f_ spot was characterized as 3,6-di-*O-*benzoate **30** (20% isolated yield), alongside the highest *R*_f_ spot as per-benzoylated thioglycoside **27** (13%).^38^ Characterization of the middle spot revealed two inseparable sugars, 3,4,6-tri-*O*-benzoate **28** (19%) and 2,3,6-tri-*O*-benzoate **29** (20%), as determined by HMBC analysis (See Supporting Information, Figure S4). While the overall yield for this transformation was comparable to previously (Table 1), the loss of esterification regioselectivity indicated that the relative nucleophilicity of C2-OH was lower for β-thioglycoside **26** than for α-**3**, evidenced through formation of **28** and **30**. Furthermore, the ability of C4-OH to compete with C2-OH delivered **27** and **28**.

**Table 2:**
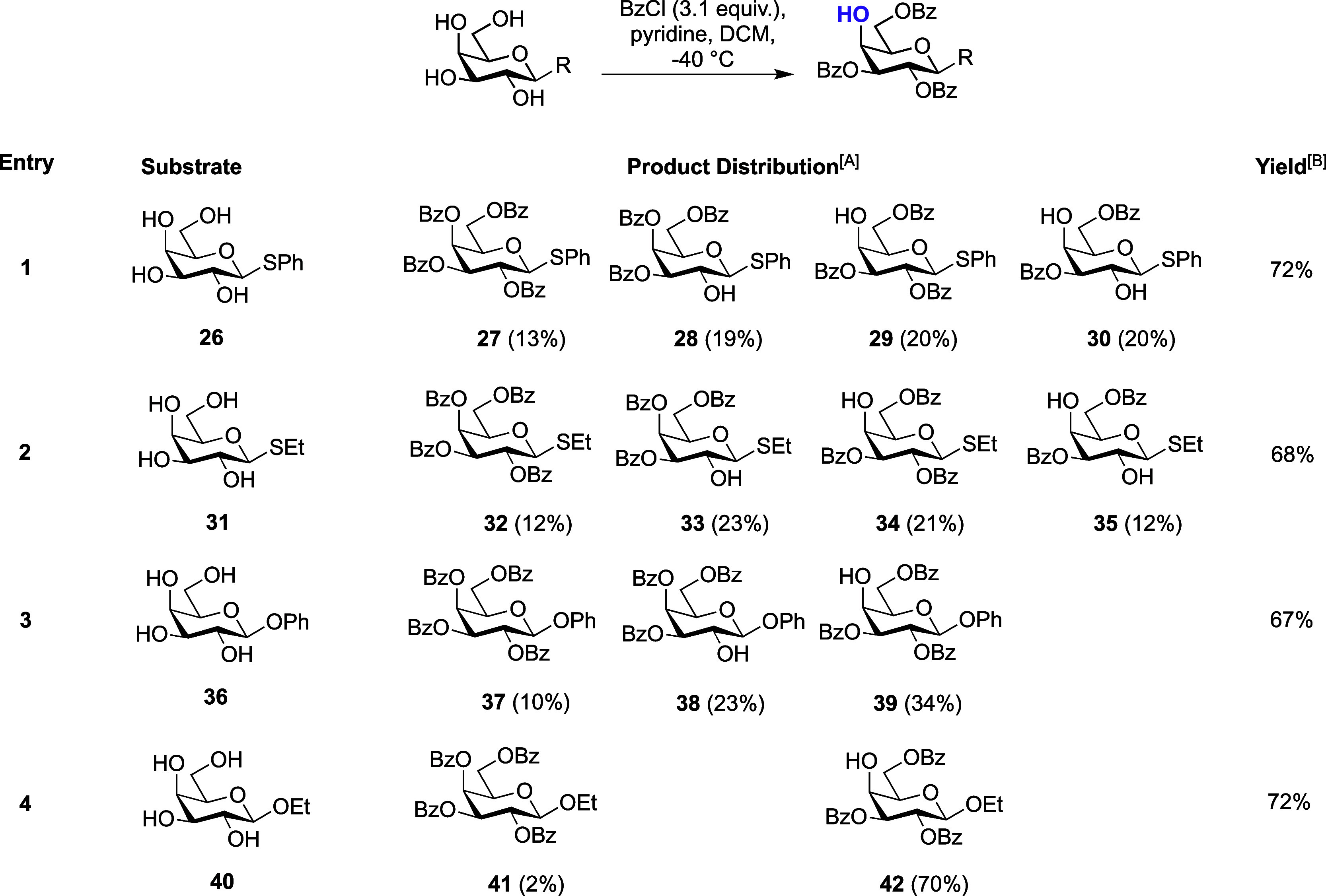
Benzoylation of β-linked *O*- and *S*-galactopyranosides

A Product distribution percentages based on the isolated yield.

B Overall isolated yield.

We next screened β-SEt substrate **31**, observing similar results (Table 2, entry 2), further indicating a competitive nucleophilicity between 2-OH and 4-OH for β-linked thioglycosides. We repeated the reactions for **26** and **31** at −78 °C but this had no effect on the regioselectivity outcome. We were curious to see whether this loss of regioselectivity mapped to β-linked *O*-glycosides. Starting with β-OPh glycoside **36** and following a 2-h reaction, TLC analysis indicated two components and complete consumption of starting material (Table 2, entry 3). Following purification, analysis by NMR revealed a fully benzoylated product **37** (10%), alongside the second spot as an inseparable mixture of desired 2,3,6-tri-*O*-benzoylated **39** (34%) and 3,4,6-tri-*O*-benzoylated **38** (23%). Parallels to the β-thioglycoside results were evident but with an increased preference for the desired product (34% **39** versus 20% for **29**).

Finally, we evaluated β-OEt glycoside **40** (Table 2, entry 4) and were somewhat surprised to isolate 2,3,6-tri-*O*-benzoate **42** as the major product in 70% yield (2% per-benzoylated **41** was also isolated). This demonstrated a similar outcome to that observed for all α-linked examples. Considering these results in the context of Figure 2, the observations for **26**, **31**, and **36** are somewhat expected; removal of the axial anomeric substituent at C1 reduces the nucleophilic prevalence of 2-OH versus 4-OH and enables component mixtures to form. To support this hypothesis, a mixing experiment utilizing β-SEt 3,6-di-*O*-benzoate **35** and its α-linked counterpart (see Supporting Information, compound S1) was performed. Following the subjection of an equimolar mixture of these compounds to 1.0 equiv of BzCl, the major product observed was 2,3,6-tri-*O*-benzoate **2**, with **35** remaining largely unreacted (see Supporting Information for details). This confirmed an experimentally observed preference for increased reactivity of 2-OH in the α-SEt form.

However, this did not explain the almost complete regioselectivity observed for β-OEt glycoside **40**. We reasoned that the steric bulk of the aglycon may influence the regioselectivity of esterification by masking the 2-OH, given that all other reaction parameters remained constant (and assuming acylation of 6-OH and 3-OH completed first). The least sterically demanding substrate, β-OEt **40**, exhibits a complete reaction at the 2-OH, while β-OPh **36** exhibits only 34% (in forming **39**), indicating an influence of the steric change from Et to Ph within the aglycon. Switching to thioglycosides and increasing the size of the aglycon heteroatom directly attached to the anomeric center, β-SEt **31** and β-SPh **26** exhibit lower selectivity (20% for **29** and 21% for **34**). These observations establish a general pattern that was (unexpectedly) not finite with respect to the regioselectivity outcome for α-linked glycosides versus β-linked systems, and we decided to investigate further β-linked anomeric substituents.

### Exploring the Influence of a β-anomeric Substituents Upon Regioselectivity

We considered whether the electronic nature of the β-anomeric substituent could influence the regioselectivity outcome. Noting the lack of regioselectivity observed for β-SPh **26** and β-SEt **31** (Table 2, entries 1 and 2),^40^ we synthesized substrates **43** and **49**, selecting a cyclohexyl aglycon thus removing aromaticity,^40^ alongside 2,2,2-trifluoroethyl tetrols **45** and **54**, to gauge the effect of a small, strongly electron-withdrawing alkyl aglycon.

Thioglycoside **43** bearing a cyclohexyl aglycon was subjected to benzoylation and after 20 min of reaction only a single spot was observed by TLC (Table 3, entry 1). This was characterized as 2,4-diol **44**. The reaction was repeated for 24 h but did not progress beyond this product, again affording **44** in 64% yield. β-*S*-2,2,2-Trifluoroethyl glycoside **45** produced a small amount of fully benzoylated product **46** (14%) along with a mixture of 2-*O* and 4-*O*-benzoyl regioisomers, **47** and **48** (27 and 42%, Table 3, entry 2). Notably, this inseparable mixture favored the desired 2,3,6-tri-*O*-benzoate **48**, offering the best regioselectivity thus far for any β-configured thioglycoside (42% for **48** versus 20% for **29** and 21% for **34**). These results indicate that the electron-withdrawing 2,2,2-trifluoroethyl aglycon slightly increased the selectivity for the 2,3,6-tri-*O*-benzoate.

**Table 3:**
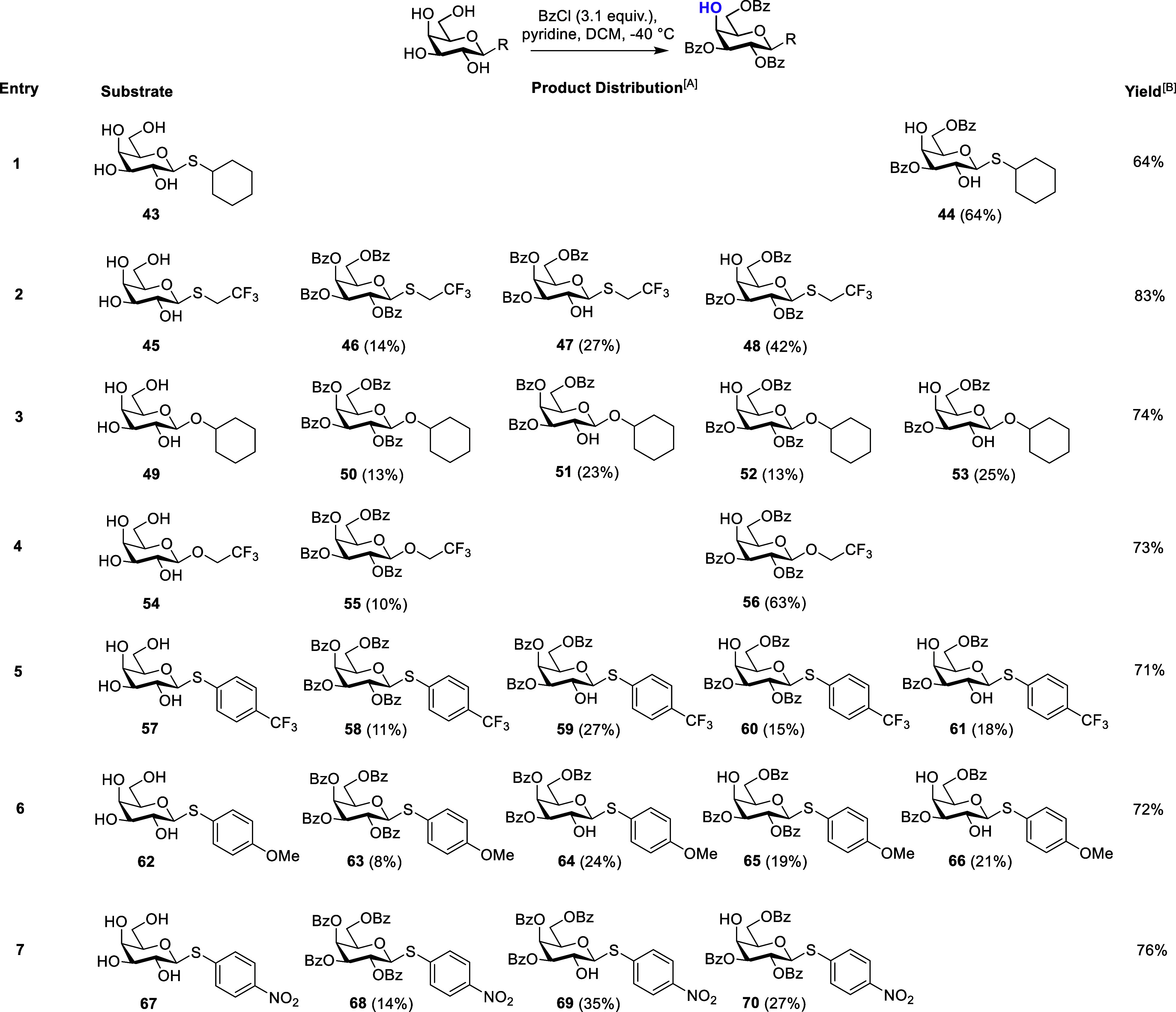
Benzoylation of β-*S*-galactopyranosides Varying the Electronic Nature of the Aglycon

A Product distribution percentages based on isolated yield.

B Overall isolated yield.

To compare with *O*-glycosides, cyclohexyl β-*O*-glycoside **49** delivered a 13% yield of the desired benzoate **52** (Table 3 entry 3) and was the first example where an under-acylated 2,4-diol was isolated for a β-*O*-glycoside (25% for **53**). The other major product from this reaction was 3,4,6-tri-*O*-benzoate **51**, accounting for 23% of the isolated yield. A β-*O*-2,2,2-trifluoroethyl substrate, **54**, produced a small amount of fully benzoylated material **55** (10%), but the major product was the desired 2,3,6-tri-*O*-benzoate **56** (63%, Table 3 entry 4).

Having established low-temperature benzoylation could be extended to β-*O*-galactosides to deliver 2,3,6-tri-*O*-benzoates in good yields (70% for **42**, Table 2, entry 4 and 63% for **56**, Table 3 entry 4), we returned to focus on synthetically malleable β-thioglycosides, noting an earlier unfavorable outcome for β-SPh and β-SEt 2,3,6-tri-*O*-benzoates **29** and **34** (20 and 21% yields respectively, Table 2, entries 1 and 2). We reasoned to explore the effect of introducing electron donating and withdrawing groups to the phenyl component of the aglycon, as tractability for this was more accessible than for comparative β-*S*-alkyl systems (beyond the results already obtained for **45**). Accordingly, we synthesized β*-S*-*para-*nitrophenyl, β*-S*-*para-*trifluorophenyl and β*-S*-*para-*methoxyphenyl derivatives **57**, **62** and **67** and these substrates were screened under the standard conditions (Table 3, entries 5–7). An electron-withdrawing β*-S*-*para-*trifluorophenyl substrate **57** produced a mixture of regioisomers alongside under- and overbenzoylation products in a manner similar to β-SPh glycoside **26**. Switching to an electron-donating *para*-substituent, β*-S*-*para-*methoxyphenyl **62**, the result was similar, with the major isolated component the 3,4,6-tri-*O*-benzoate (Table 3, entry 6). Finally, a strong electron-withdrawing β*-S*-*para-*nitrophenyl galactoside **67** gave a product distribution that mirrored β*-S*-CH_2_CF_3_ glycoside **45** (i.e., no under-benzoylation) and differed to a previous result reported for **67** (57% yield of **70** with 5% of **68** and <3% of other regioisomers).^41^

Considering the results presented in Table 3, the electronic nature of the aglycon also influences the regioisomeric outcome for β-galactoside tetrol benzoylation, albeit tensioned against the steric effects upon 2-OH reactivity discussed earlier. When the aglycon is large and not aromatic, e.g., *O*-cyclohexyl substrate **49** (Table 3, entry 3), selectivity for the 2,3,6-tri-*O*-benzoate decreases, with 48% of the product distribution having no reaction at the 2-OH (**51** and **53**). Furthermore, changing to a larger heteroatom, β-*S*-cyclohexyl, results in no reaction taking place at 2-OH at all (only diol **44** was isolated upon the acylation of tetrol **43**). When the β-anomeric substituent is electron-withdrawing and small alkyl (2,2,2-trifluoroethyl), a preference toward the 2,3,6-tri-*O*-benzoate is observed (Table 3, entries 2 and 4). Notably, this is intertwined with the identity of the glycosidic linkage heteroatom, with the size of sulfur (versus oxygen) hindering complete acylation regioselectivity (42% for **48** versus 63% for **56**).

The introduction of a *para-*substituent within β-*S*-phenyl glycosides did not confer an improvement in regioselectivity outcome (compare parent β-*S*Ph **26** with **57**, **62**, and **67**). Examining β*-S*-*para-*trifluorophenyl substrate **57**, the 4-OH appeared more reactive than the 2-OH (27% for **59** versus 15% for **60**). This effect was mirrored in substrates **62** and **67** (24% for **64** versus 35% for **69**), and all three examples indicated a slight preference for the 3,4,6-tri-*O*-benzoate as the major product in these mixtures. Comparatively, the absence of a *para*-substituent (β-SPh **26**) saw similar amounts of 2,3,6- and 3,4,6-tri-*O*-benzoates isolated (19% for **28** versus 20% for **29**).

**Figure 3:**
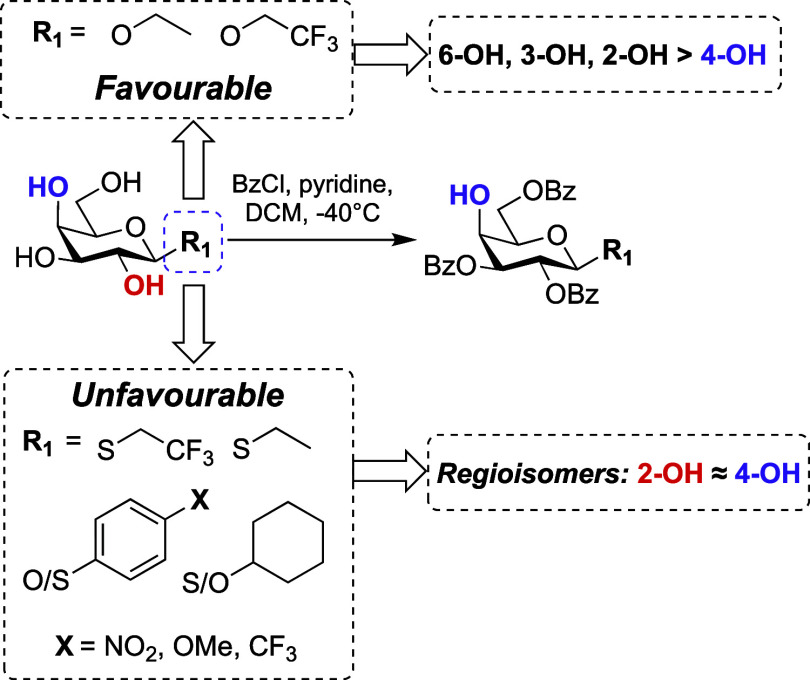
Observed trends for the regioselective benzoylation of β-d-galactoside tetrols.

Taken together, these results build upon observations established for α-galactoside ring hydroxyl reactivity, generally considered to be 6-OH > 3-OH > 2-OH > 4-OH,^17−19^ but introduce new patterns for β-anomeric substituents, highlighted in Figure 3. All α-configured substrates examined align with an alkoxy group mediated diol effect, allowing for efficient regioselective synthesis of 2,3,6-tri-*O*-benzoates using simple, low-temperature esterification with benzoyl chloride and no other additives. Furthermore, this can now be extended to α-thioglycosides and synthetic planning incorporating this acylation methodology should be straightforward as high-yielding syntheses of α-configured thioglycosides have been developed.^42,43^

In the case of β-galactosides (switching to a system that does not exert an alkoxy group mediated diol effect), a combination of size and electronic nature of the aglycon play a role in the observed regioselectivity outcome. Larger aglycons appear to prohibit selective protection. This is evident for all the β-thioglycosides evaluated here (**26**, **31**, **43**, **45, 57, 62, 67**), where complete regioselectivity for the 2,3,6-tri-*O*-benzoate was not observed. It is plausible that the larger size of the aglycon heteroatom and the identity of the adjoining ring/chain restrict the reaction with the electrophile at 2-OH, thus enabling increased competing reaction at 4-OH for the remaining electrophile. The inclusion of different functional groups within the aryl component of β-*S*-phenyl glycosides does not support a regioselective outcome and further design and experimentation is required to fully explore this concept (e.g., size, position, and number of aryl substituents). Conversely, a smaller β-aglycon with an electronegative heteroatom enables regioselective protection, notably for *O*-glycosides **40** and **54**. However, these steric considerations must be tensioned with electronic effects, most notable where high regioselectivity is lost upon changing the heteroatom from oxygen in **54** to sulfur in **45**, and reactivity of 4-OH competes within these 2,2,2-trifluoroethyl systems. Furthermore, the inclusion of a strong electron-withdrawing aglycon can reduce overall regioselectivity outcome, comparing *O*-Et **40** and *O*-CH_2_CF_3_**54**, possibly due to a remote electronic effect reducing the reactivity of the 2-OH. Related reports of such remote electronic effects within C2 *para*-substituted ether-protecting groups have been observed to fine-tune the reactivity of thioglycoside donors.^44^

## Conclusions

Methods to efficiently effect regioselective hydroxyl group protection within carbohydrates are of significant value. We have explored a protocol for the preparation of 2,3,6-*O*-benzoylated d-galactose building blocks, differentiating the reactivity for three of the four hydroxyl groups within tetrol starting materials. Capability is demonstrated for variation of the α-*O*-anomeric substituent (e.g., propargyl or alkyl azido), *S*-glycosides, d-galactosamines and l-fucose with isolated yields observed >65%. We extend this method to investigate β-linked systems, establishing that both steric and electronic factors contribute to an overall reduced nucleophilic capability of the 2-OH (and increased reaction at 4-OH), impacting regioselectivity outcome. However, certain aglycon components are available to parallel the outcome for α-linked systems, namely, β-*O*-Et and β-*O*-CH_2_CF_3_. Inherent reactivity differences for the selective protection of glycosides can be achieved by altering reagents and reaction conditions to exploit subtle differences between identical functional groups.^45^ By exploring the configuration and identity of the aglycon in effecting the outcome of such reactions, similar patterns can be developed from the perspective of the substrate and these results will help to inform the future design of suitably protected *O*- and *S*-galactoside building blocks, notably also opening the opportunity for the exploration of other sugars.^46−48^

## Data Availability

The data underlying this study are available in the published article and its Supporting Information.

## Supporting Information Available

Experimental procedures for all compounds (PDF)
Relevant 1D and 2D NMR spectra for all compounds (PDF)
