# Supplementary material for: Benzoylation of Tetrols: A Comparison of Regioselectivity Patterns for O- and S-Glycosides of d-Galactose
Source: J Org Chem. 2024 Sep 12;89(19):14090–7. doi: 10.1021/acs.joc.4c01508 (PMC11460728; doi:10.1021/acs.joc.4c01508)
Supplement: Supplementary file 1 — jo4c01508_si_001.pdf [file jo4c01508_si_001.pdf]

Benzoylation of tetrols: a comparison of regioselectivity patterns for *O*- and *S*-glycosides of D-galactose

Jack Porter, Jacob Roberts and Gavin J. Miller*

Centre for Glycoscience and School of Chemical and Physical Sciences, Keele University, Keele, Staffordshire, ST5 5BG, United Kingdom

* Email: g.j.miller@keele.ac.uk

Supporting Information 1

Experimental

Table of Contents

1.0 General Experimental S2

1.1 General Acylation Procedure..S3

1.2 General Deacetylation Procedure...S3

1.3 Mixing Experiment demonstrating increased 2-OH reactivity for α-S1 compared with β-35...S3

1.4 HMBC NMR of β-thioglycoside regiosiomers 28 (purple) and 29 (red)................S5

2. Substrate Synthesis S6

3. Benzoylation Experiments..S21

4. References S45

1.0 General Experimental

All chemicals were purchased from Acros Organics, Alfa Aesar, Biosynth, Fisher Scientific, Fluorochem, Sigma Aldrich or TCI Chemicals and were used without further purification unless otherwise stated. NMR spectra were recorded on a Bruker Avance 400 spectrometer. For reactions that required heating, DrySyn heating blocks were used as the heat source. The chemical shift data for each signal are given as δ in units of parts per million (ppm) relative to tetramethylsilane, where δ = 0.00 ppm. The number of protons (n) for a given resonance is indicated by nH. The multiplicity of each signal is indicated by s (singlet), bs (broad singlet), as (apparent singlet), ad (apparent doublet), d (doublet), t (triplet), q (quartet), p (pentet), sep (septet), dd (doublet of doublets), ddd (doublet of doublet of doublets), dddd (doublet of doublet of doublet of doublets), dt (doublet of triplets), tt (triplet of triplets), dqd (doublet of quartets of doublets) or m (multiplet). Coupling constants (*J*) are quoted in Hz and calculated to the nearest 0.1 Hz. Structural assignments were made with additional information from gCOSY, gHSQC, and gHMBC experiments. Anhydrous DCM and pyridine were obtained from Sure/Seal™ bottles *via* chemical suppliers. Anhydrous THF, DCM and toluene were obtained by passing solvent through activated alumina columns and dispensed from a PureSolv MD ASNA solvent purification system and stored over 4 Å molecular sieves. Unless otherwise stated, all reactions were conducted using anhydrous solvents, under an atmosphere of N_2 which was passed through a Drierite® drying column. HRMS were recorded on a ThermoScientific LTQ Orbitrap XL at the ESPRC National Mass Spectrometry Facility at Swansea University. Analytical thin layer chromatography (TLC) was carried out on pre-coated 0.25 mm Merck KgaA 60 F254 silica gel plates. Visualisation was by adsorption of UV light, or thermal development after dipping in a methanolic solution of sulfuric acid (5% v/v). Automatic flash chromatography was carried out on silica gel (Reveleris® X2 system) under a positive pressure of compressed N_2. Assignment of ^{1}H and $^{f\{d\}}$ atoms in NMR follows standard pyranose ring numbering. Optical rotations were recorded on a Bellingham + Stanley ADP430 (specific rotation, tube length: 50 mm, concentrations in g per 100 mL). RT: room temperature. Generic carbohydrate ring numbering shown below.

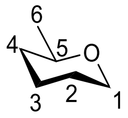

1.1 General Acylation Procedure

A solution of the substrate (1.0 equiv.) in pyridine/DCM (1:1, 50mM) was cooled to -40 °C (acetone/liquid N_2) and treated dropwise with a solution of electrophile (3.1 or 2.1 equiv.) in DCM (1 mL). The reaction mixture was maintained at -40 °C until reaction completion, as seen by TLC. Saturated aqueous $NaHCO_3$ (50 mL) was then added with stirring and the aqueous layer extracted with DCM (3 × 50 mL). The combined organic phases were washed with water (50 mL), dried ($MgSO_4$), filtered and evaporated under reduced pressure to yield the crude product, which was purified by column chromatography.

1.2 General Deacetylation Procedure

To the peracetylated glycoside (1.0 equiv.) was added Na (0.1 equiv.) in MeOH (5 mL), the resulting mixture was stirred at RT. Upon reaction completion as seen by TLC Amberlite IR120 (H^+) ion exchange resin was added. The mixture was stirred until neutral (confirmed using Johnson® pH paper) after which the mixture was filtered and washed with MeOH (100 mL). The combined organic filtrates were concentrated under reduced pressure and the crude residue purified by column chromatography.

1.3 Mixing Experiment demonstrating increased 2-OH reactivity for α-S1 *versus* β-35

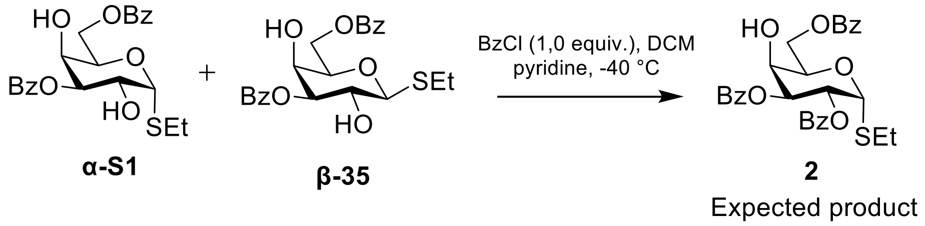

Scheme S1: Following the general acylation procedure, ethyl 3,6-di-*O*-benzoyl-1-thio-α/β-D-galactopyranoside (38.0 mg. 87.9μmol, 1.0 equiv., α/β, 1:1) and BzCl (10.2 μL, 87.9 μmol, 1.0 equiv.) were reacted. From this product **2** was observed as the major product as highlighted in ***Figures S1-S3***.

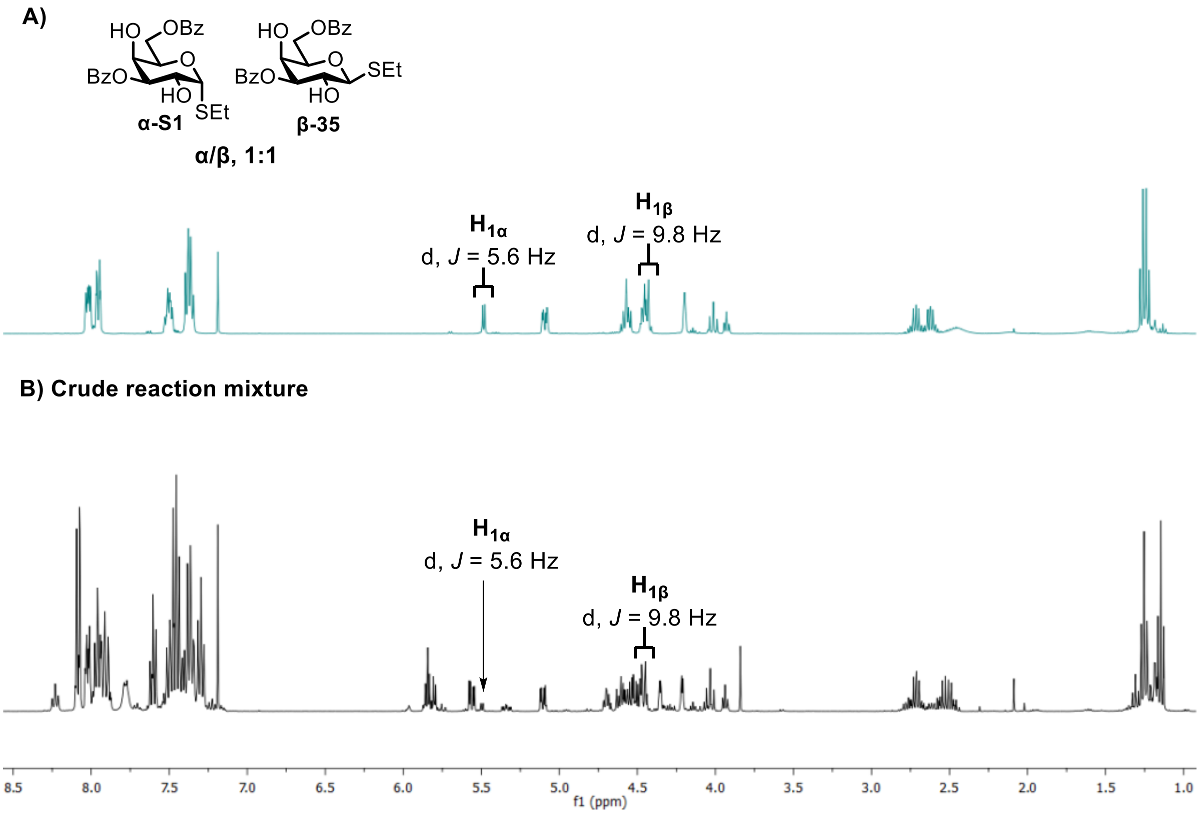

Figure S1: A) ^{1}H NMR ($CDCl_3$, 400 MHz) spectrum of the 2,6-di-*O*-benzoate α/β mix prior to benzoylation; B) ^{1}H NMR ($CDCl_3$, 400 MHz) spectrum of the crude reaction mixture following benzoylation.

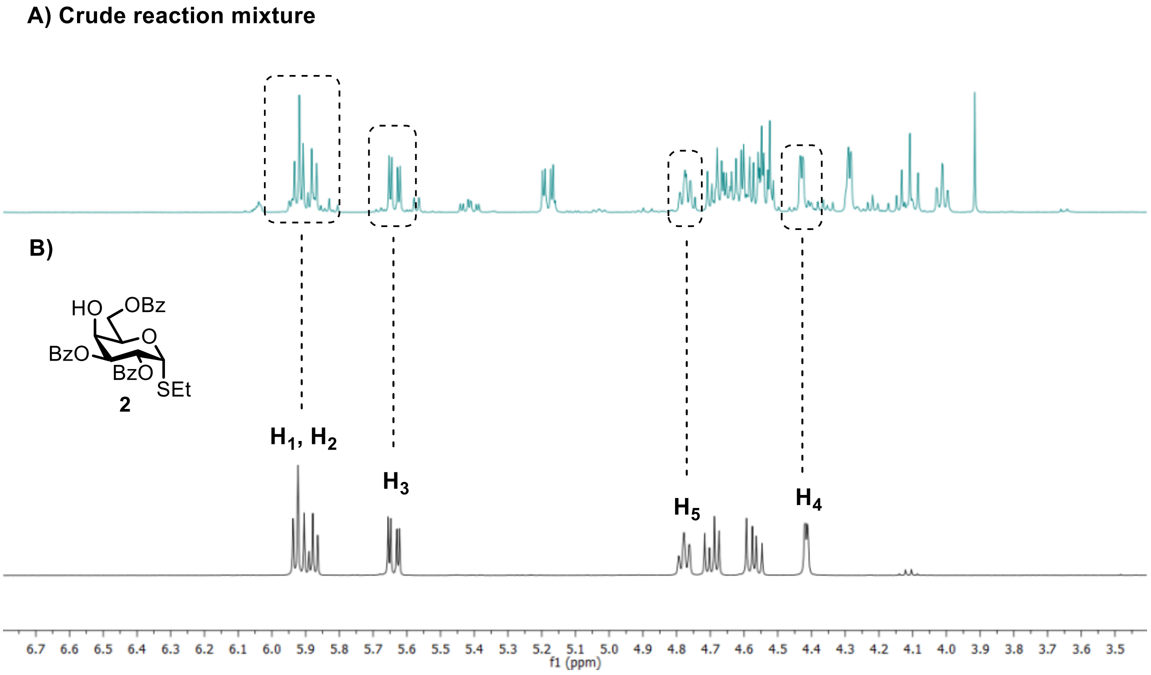

Figure S2: Comparison of the crude reaction mixture with pure **2**; A) ^{1}H NMR ($CDCl_3$, 400 MHz) spectrum of the crude reaction mixture following benzoylation; B) ^{1}H NMR ($CDCl_3$, 400 MHz) spectrum of **2**.

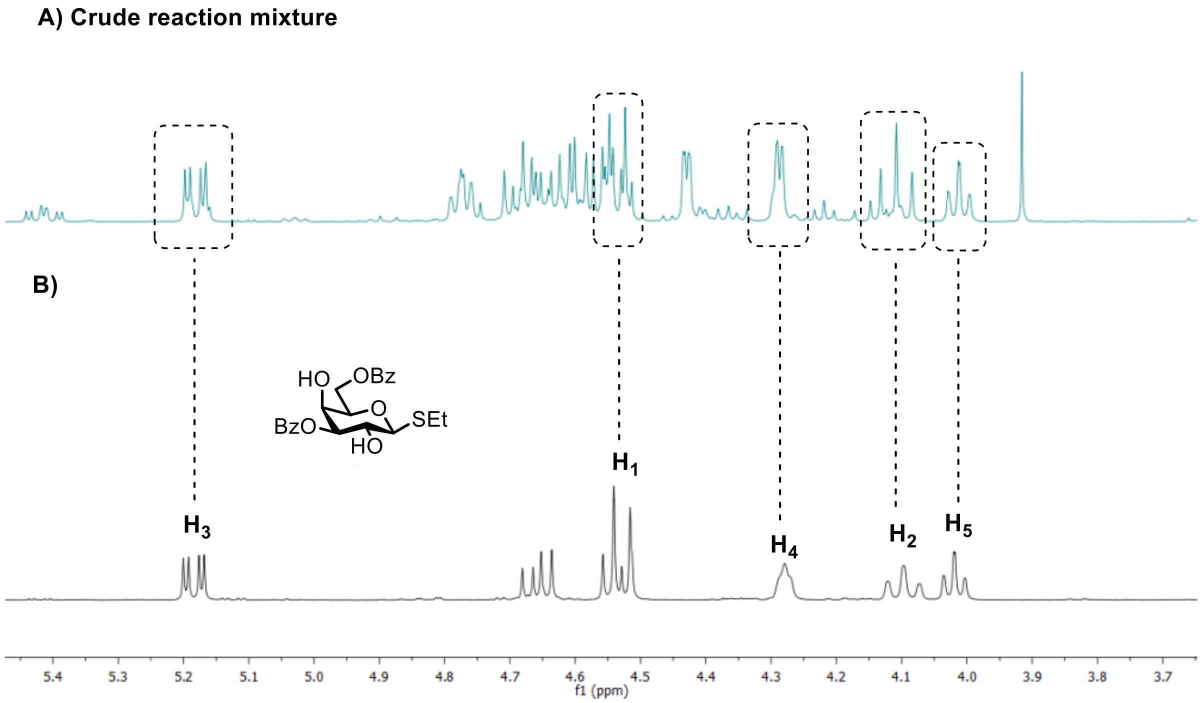

Figure S3: Comparison of the crude reaction mixture with **35**; A) ^{1}H NMR ($CDCl_3$, 400 MHz) spectrum of the crude reaction mixture following benzoylation; B) ^{1}H NMR ($CDCl_3$, 400 MHz) spectrum of **35**.

1.4. HMBC NMR of β-thioglycoside regiosiomers 28 (purple) and 29 (red)

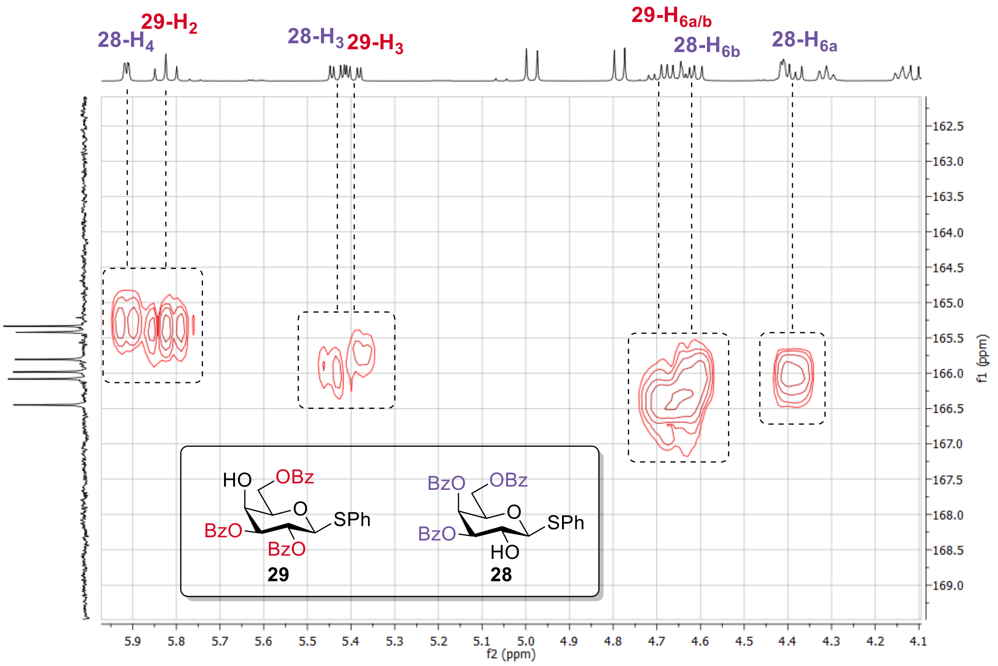

Figure S4: HMBC NMR of β-thioglycoside regiosiomers **28** (purple) and **29** (red), highlighting ^{13}C-^{1}H correlations for benzoylation of respective ring positions.

2. Substrate Synthesis

3-Azidopropyl (2-acetamido-2-deoxy)-β-D-galactopyranoside **19** used in this work was synthesised as reported previously by *Keenan et al.*[1]

Phenyl 1-thio-α/β-D-galactopyranoside 3 & 26

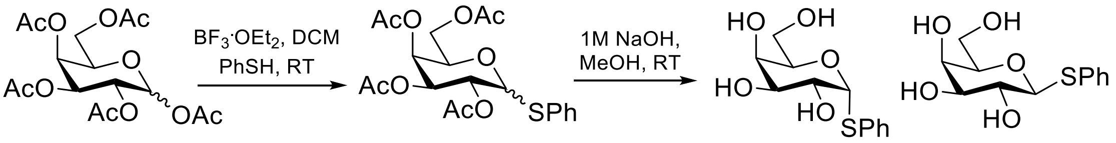

α-3 **β-26**

1,2,3,4,6-Penta-*O*-acetyl-α/β-D-galactopyranoside (6.50 g, 16.7 mmol, 1.0 equiv.) was dissolved in DCM (25 mL) and $BF_3{\cdot}OEt_2$ (8.24 mL, 66.8 mmol, 4.0 equiv.) and HSPh (2.55 mL, 25.1 mmol, 1.5 equiv.) added dropwise at RT and stirred overnight. The reaction mixture was diluted with DCM (100 mL) and washed successively with saturated aqueous $NaHCO_3$ (3 × 100 mL). The combined organic layers were dried ($MgSO_4$), filtered and evaporated under reduced pressure and the crude residue progressed without further purification. To the crude material was added 1M NaOH (5 mL) and MeOH (5 mL), the resulting mixture was stirred at RT for 1 hour (R_f = α 0.37, β 0.24 acetone/toluene, 6:4). Amberlite IR20 (H^+) ion exchange resin was added, and the mixture stirred until neutral, filtered and washed with MeOH (100 mL). The combined organic filtrates were concentrated under reduced pressure and the crude residue purified by column chromatography (toluene/acetone, 0-100%) to yield the title compounds **α-3** (1.95 g, 7.16 mmol, 43%) and **β-26** (1.72 g, 6.32 mmol, 38%) individually as white solids. **α-3:** ^{1}H NMR (400 MHz, MeOD) δ 7.56 – 7.53 (m, 2H, Ar*H*), 7.32 – 7.22 (m, 3H, Ar*H*), 5.58 (d, *J* = 5.5 Hz, 1H, H-1), 4.34 (ddd, *J* = 6.6, 6.0, 0.9 Hz, 1H, H-5), 4.16 (dd, *J* = 10.2, 5.5 Hz, 1H, H-2), 3.97 (dd, *J* = 3.3, 1.2 Hz, 1H, H-4), 3.73 (dd, *J* = 11.4, 5.8 Hz, 1H, H-6a), 3.69 – 3.63 (m, 2H, H-3, H-6b); ^{13}C{^{1}H} NMR (101 MHz, MeOD) δ 134.8 (Ar-C), 132.0 (Ar-C), 128.5, (Ar-C) 126.8 (Ar-C), 90.3 (C1), 71.8 (C4), 70.9 (C3), 69.4 (C5), 68.6 (C2), 60.9 (C6); **β-26:** ^{1}H NMR (400 MHz, MeOD) δ 7.59 – 7.51 (m, 2H, Ar*H*), 7.33 – 7.18 (m, 3H, Ar*H*), 4.58 (d, *J* = 9.7 Hz, 1H, H-1), 3.90 (dd, *J* = 3.3, 0.8 Hz, 1H, H-4), 3.76 (dd, *J* = 11.4, 6.8 Hz, 1H, H-6a), 3.70 (dd, *J* = 11.4, 5.3 Hz, 1H, H-6b), 3.60 (t, *J* = 9.4 Hz, 1H, H-2), 3.56 (ddd, *J* = 6.6, 5.3, 1.0 Hz, 1H, H-5), 3.49 (dd, *J* = 9.2, 3.3 Hz, 1H, H-3); ^{13}C{^{1}H} NMR (101 MHz, MeOD) δ 134.7 (Ar-C), 130.7 (Ar-C), 128.5 (Ar-C), 126.6 (Ar-C), 88.9 (C1), 79.2 (C4), 75.0 (C3), 69.6 (C2), 69.0 (C5), 61.2 (C6); HRMS *m/z* (ES^-) Found: $(M-H)^-$ 271.0647, $C_{12}H_{15}O_5S$, requires M^- 271.0646. Data matched those reported previously.[2]

Ethyl 2,3,4,6-tetra-*O*-acteyl-1-thio-α/β-D-galactopyranoside S2 & S3

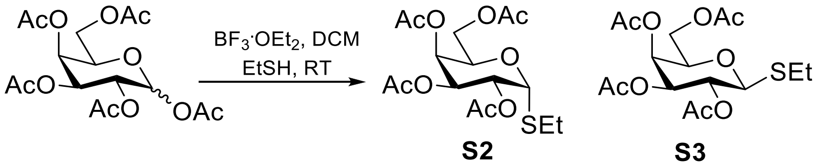

S2 **S3**

1,2,3,4,6-Penta-*O*-acetyl-α/β-D-galactopyranoside (2.00 g, 5.12 mmol, 1.0 equiv.) was dissolved in DCM (20 mL) and $BF_3{\cdot}OEt_2$ (2.53 mL, 20.5 mmol, 4.0 equiv.) and HSEt (1.85 mL, 25.1 mmol, 1.5 equiv.) added dropwise at RT and stirred until complete consumption of the starting material, as seen by TLC (R_f = β 0.70, α 0.65, hexane/EtOAc, 6/4). The reaction mixture was diluted with DCM (100 mL) and washed with saturated aqueous $NaHCO_3$ (3 × 100 mL). The combined organic layers were dried ($MgSO_4$), filtered and evaporated under reduced pressure. The crude residue purified by column chromatography (0-50%, hexane/EtOAc) to yield the title compounds **α-S2** (525 mg, 1.34 mmol, 26%) and **β-S3** (756 mg, 1.93 mmol, 38%) individually as white solids. **α-S2** ^{1}H NMR (400 MHz, $CDCl_3$) δ 5.75 (d, *J* = 5.4 Hz, 1H, H-1), 5.45 (dd, *J* = 3.1, 0.9 Hz, 1H, H-4), 5.28 (dd, *J* = 10.9, 5.4 Hz, 1H, H-2), 5.22 (dd, *J* = 10.6, 2.9 Hz, 1H, H-3), 4.60 (t, *J* = 6.5 Hz, 1H, H-5), 4.12 (as, 1H, H-6a), 4.10 (s, 1H, H-6b), 2.66 – 2.47 (m, 2H, CH_2), 2.15 (s, 3H, OAc), 2.08 (s, 3H, OAc), 2.04 (s, 3H, OAc), 1.99 (s, 3H, OAc), 1.28 (t, *J* = 7.4 Hz, 3H, CH_3); ^{13}C{^{1}H} NMR (101 MHz, $CDCl_3$) δ 170.4 (C=O, Ac), 170.2 (C=O, Ac), 169.9 (C=O, Ac), 82.0 (C1), 68.2 (C3), 68.00 (C2), 67.97 (C4), 66.5 (C5), 61.8 (C6), 24.0 (CH_2), 20.9 (Ac-CH_3), 20.68 (Ac-CH_3), 20.65 (Ac-CH_3), 20.6 (Ac-CH_3), 14.7 (CH_3); **β-S3:** ^{1}H NMR (400 MHz, $CDCl_3$) δ 5.43 (dd, *J* = 3.4, 0.9 Hz, 1H, H-4), 5.24 (t, *J* = 10.0 Hz, 1H, H-2), 5.05 (dd, *J* = 10.0, 3.4 Hz, 1H, H-3), 4.50 (d, *J* = 9.9 Hz, 1H, H-1), 4.17 (dd, *J* = 11.3, 6.7 Hz, 1H, H-6a), 4.11 (dd, *J* = 11.3, 6.6 Hz, 1H, H-6b), 3.94 (td, *J* = 6.6, 1.0 Hz, 1H, H-5), 2.82 – 2.63 (m, 2H, CH_2), 2.15 (s, 3H, OAc), 2.07 (s, 3H, OAc), 2.05 (s, 3H, OAc), 1.99 (s, 3H, OAc), 1.29 (t, *J* = 7.5 Hz, 3H, CH_3); ^{13}C {1H} NMR (101 MHz, $CDCl_3$) δ 170.4 (C=O, Ac), 170.2 (C=O, Ac), 170.1 (C=O, Ac), 169.6 (C=O, Ac), 84.1 (C1), 74.4 (C5), 71.9 (C3), 67.3 (C2), 67.2 (C4), 61.5 (C6), 24.4 (CH_2), 20.8 (Ac-CH_3), 20.7 (Ac-CH_3), 20.6 (Ac-CH_3), 14.9 (CH_3): HRMS *m/z* (ES^+) Found: $(M+Na)^+$ 415.1042, $C_{16}H_{24}O_9SNa$, requires M^+ 415.1038. Data matched those reported previously.[3]

Ethyl 1-thio-β-D-galactopyranoside 31

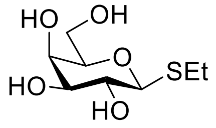

Following the general deacetylation procedure, ethyl 2,3,4,6-tetra-*O*-acteyl-1-thio-β-D-galactopyranoside **S3** (520 mg, 1.33 mmol, 1.0 equiv.) was deprotected using Na (3.00 mg, 0.133 mmol, 0.1 equiv.) in MeOH (5 mL). Following column chromatography (DCM/MeOH, 0-20%) the title compound **31** was obtained as a colourless oil (260 mg, 1.16 mmol, 87%). R_f = 0.54 (DCM/MeOH, 8:2); ^{1}H NMR (400 MHz, MeOD) δ 4.33 (d, *J* = 9.5 Hz, 1H, H-1), 3.90 (dd, *J* = 3.2, 0.8 Hz, 1H, H-4), 3.74 (dd, *J* = 11.4, 6.8 Hz, 1H, H-6a), 3.69 (dd, *J* = 11.4, 5.3 Hz, 1H, H-6b), 3.58 – 3.51 (m, 2H, H-2, H-5), 3.48 (dd, *J* = 9.2, 3.3 Hz, 1H, H-3), 2.84 – 2.65 (m, 2H, CH_2), 1.28 (t, *J* = 7.4 Hz, 3H, CH_3); ^{13}C{^{1}H} NMR (101 MHz, $CDCl_3$) δ 90.0 (C1), 83.2 (C2), 78.8 (C3), 73.9 (C5), 73.1 (C4), 65.2 (C6), 27.5 (CH_2), 18.0 (CH_3); HRMS *m/z* (ES^-) Found: $(M-H)^-$ 223.0646, $C_8H_{15}O_5S$, requires M^- 223.0646. Data matched those reported previously.[4]

Ethyl 1-thio-α-D-galactopyranoside 1

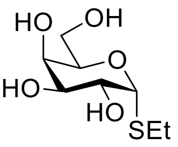

Following the general deacetylation procedure, ethyl 2,3,4,6-tetra-*O*-acteyl-1-thio-α-D-galactopyranoside **S2** (520 mg, 1.33 mmol, 1.0 equiv.) was deprotected using Na (3.00 mg, 0.133 mmol, 0.1 equiv.) in MeOH (5 mL). Following column chromatography (DCM/MeOH, 0-20%) the title compound **1** was obtained as a colourless oil (236 mg, 0.87 mmol, 66%). R_f = 0.54 (DCM/MeOH, 8:2); ^{1}H NMR (400 MHz, MeOD) δ 5.41 (d, *J* = 5.6 Hz, 1H, H-1), 4.18 (ddd, *J* = 6.5, 5.9, 0.8 Hz, 1H, H-5), 4.08 (dd, *J* = 10.1, 5.6 Hz, 1H, H-2), 3.89 (dd, *J* = 3.3, 1.1 Hz, 1H, H-4), 3.72 (as, 1H, H-6a), 3.71 (s, 1H, H-6b), 3.60 (dd, *J* = 10.1, 3.3 Hz, 1H, H-3), 2.70 – 2.51 (m, 2H, CH_2), 1.28 (t, *J* = 7.4 Hz, 3H, CH_3); $^{13}C\{^1H\}$ NMR (101 MHz, MeOD) δ 85.6 (C1), 71.3 (C5), 70.9 (C3), 69.5 (C4), 68.3 (C2), 61.2 (C6), 23.3 (CH_2), 13.8 (CH_3); HRMS *m/z* (ES^-) Found: $(M-H)^-$ 223.0646, $C_8H_{15}O_5S$ requires M^- 223.0646. Data matched those reported previously.[5]

Phenyl 2,3,4,6-tetra-*O*-acteyl-α/β-D-galactopyranoside S4 & S5

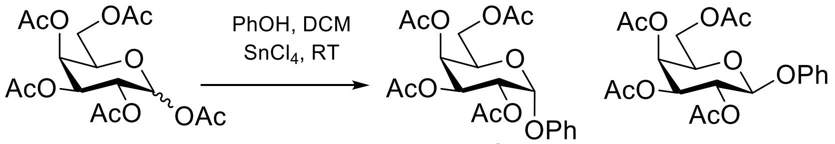

α-S4 **β-S5**

1,2,3,4,6-Penta-*O*-acetyl-α/β-D-galactopyranoside (2.00 g, 5.12 mmol, 1.0 equiv.) was dissolved in DCM (25 mL) then treated with phenol (964 mg, 10.24 mmol, 2.0 equiv.) and $SnCl_4$ (588 μL, 5.12 mmol, 1.0 equiv.). The reaction was stirred at RT until complete consumption of the starting material, as seen by TLC (R_f = α 0.44, β 0.40, hexane/EtOAc, 7:3). The reaction mixture was diluted with DCM (100 mL) and washed successively with saturated aqueous $NaHCO_3$ (3 × 100 mL). The combined organic layers were dried ($MgSO_4$), filtered and evaporated under reduced pressure. The crude residue was purified by column chromatography (hexane/EtOAc, 0-30%) yielding **α-S4** and **β-S5** (1.29 g, 3.04 mmol, 59%, α/β, 84:16) individually as white solids. **α-S4:** ^{1}H NMR (400 MHz, $CDCl_3$) δ 7.32 – 7.27 (m, 2H, Ar*H*), 7.10 – 7.03 (m, 3H, Ar*H*), 5.78 (d, *J* = 3.6 Hz, 1H, H-1), 5.59 (dd, *J* = 10.8, 3.4 Hz, 1H, H-3), 5.53 (dd, *J* = 3.4, 1.2 Hz, 1H, H-4), 5.29 (dd, *J* = 10.8, 3.6 Hz, 1H, H-2), 4.39 – 4.32 (t, *J* = 6.2 Hz, 1H, H-5), 4.13 (dd, *J* = 11.2, 6.1 Hz, 1H, H-6a), 4.06 (dd, *J* = 11.3, 7.1 Hz, 1H, H-6b), 2.17 (s, 3H, OAc), 2.07 (s, 3H, OAc), 2.04 (s, 3H, OAc), 1.94 (s, 3H, OAc); $^{13}C\{^1H\}$ NMR (101 MHz, $CDCl_3$) δ 170.4 (C=O, Ac), 170.3 (C=O, Ac), 170.2 (C=O, Ac), 170.0 (C=O, Ac), 156.3 (Ar-C), 129.6 (Ar-C), 123.0 (Ar-C), 116.8 (Ar-C), 94.9 (C1), 67.9 (C4), 67.8 (C2), 67.6 (C3), 67.2 (C5), 61.5 (C6), 20.71 (Ac-CH_3), 20.66 (Ac-CH_3), 20.6 (Ac-CH_3), 20.5 (Ac-CH_3). **β-S5:** ^{1}H NMR (400 MHz, $CDCl_3$) δ 7.30 (m, 2H, Ar*H*), 7.11 – 6.98 (m, 3H, Ar*H*), 5.50 (dd, *J* = 10.5, 8.0 Hz, 1H, H-2), 5.46 (dd, *J* = 3.4, 0.9 Hz, 1H, H-4), 5.12 (dd, *J* = 10.5, 3.4 Hz, 1H, H-3), 5.06 (d, *J* = 8.0 Hz, 1H, H-1), 4.24 (dd, *J* = 11.3, 7.0 Hz, 1H, H-6a), 4.16 (dd, *J* = 10.8, 5.8 Hz, 1H, H-6b), 4.09 – 4.05 (t, *J* = 6.1 Hz, 1H, H-5), 2.18 (s, 3H, OAc), 2.07 (s, 3H, OAc), 2.06 (s, 3H, OAc), 2.02 (s, 3H, OAc); $^{13}C\{^1H\}$ NMR (101 MHz, $CDCl_3$) δ 170.34 (C=O,

Ac), 170.25 (C=O, Ac), 170.1 (C=O, Ac), 169.4 (C=O, Ac), 157.0 (Ar-C), 129.6 (Ar-C), 123.3 (Ar-C), 117.0 (Ar-C), 99.7 (C1), 71.0 (C5), 70.9 (C3), 68.7 (C2), 66.9 (C4), 61.4 (C6), 20.72 (Ac-CH_3), 20.65 (Ac-CH_3), 20.64 (Ac-CH_3), 20.58 (Ac-CH_3); HRMS *m/z* (ES^+) Found: $(M+NH_4)^+$ 442.1705, $C_{20}H_{28}O_{10}N$ requires M^+ 442.1708. Data matched those reported previously.[6]

Phenyl α-D-galactopyranoside 5

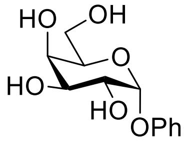

Following the general deacetylation procedure, phenyl 2,3,4,6-tetra-*O*-acteyl-α-D-galactopyranoside **S4** (436 mg, 1.03 mmol, 1.0 equiv.) was deprotected using Na (3.00 mg, 0.103 mmol, 0.1 equiv.) in MeOH (5 mL). Following column chromatography (DCM/MeOH, 0-20%) the title compound **5** was obtained as a colourless oil (203 mg, 0.79 mmol, 77%); R_f = 0.33 (DCM/MeOH, 9:1); ^{1}H NMR (400 MHz, MeOD) δ 7.31 – 7.25 (m, 2H, Ar*H*), 7.20 – 7.13 (m, 2H, Ar*H*), 7.02 – 6.93 (m, 1H, Ar*H*), 5.49 (d, *J* = 2.8 Hz, 1H, H-1), 4.00 – 3.90 (m, 4H, H-2, H-3, H-4, H-5), 3.74 – 3.62 (m, 2H, H-6a, H-6b); ^{13}C{^{1}H} NMR (101 MHz, MeOD) δ 157.5 (Ar-C), 129.0 (Ar-C), 122.0 (Ar-C), 117.0 (Ar-C), 98.4 (C1), 71.7, 70.0, 69.4, 68.6, 61.0 (C6); HRMS *m/z* (ES^+) Found: $(M+NH_4)^+$ 274.1295, $C_{12}H_{20}O_6N$ requires M^+ 274.1290. Data matched those reported previously.[7]

Phenyl β-D-galactopyranoside 36

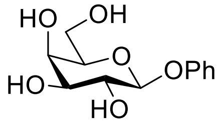

Following the general deacetylation procedure, phenyl 2,3,4,6-tetra-*O*-acteyl-β-D-galactopyranoside **S5** (212 mg, 0.50 mmol, 1.0 equiv.) was deprotected using Na (1.00 mg, 50.0 μmol, 0.1 equiv.) in MeOH (5 mL). Following column chromatography (DCM/MeOH, 0-20%) the title compound **36** was obtained as a colourless oil (101 mg, 0.395 mmol, 79%); R_f = 0.30 (DCM/MeOH, 9:1); ^{1}H NMR (400 MHz, MeOD) δ 7.29 – 7.24 (m, 2H, Ar*H*), 7.15 – 7.07 (m, 2H, Ar*H*), 7.03 – 6.97 (m, 1H, Ar*H*), 4.85 (d, *J* = 8.3 Hz, 1H, H-1), 3.90 (dd, *J* = 3.3, 0.6 Hz, 1H, H-4), 3.82 – 3.74 (m, 3H, H-2, H-6a, H-6b), 3.70 – 3.65 (m, 1H, H-5), 3.58 (dd, *J* = 9.7, 3.4 Hz, 1H, H-3); ^{13}C{^{1}H} NMR (101 MHz, MeOD) δ 157.9 (Ar-C), 129.0 (Ar-C), 121.9 (Ar-C), 116.4 (Ar-C), 101.6 (C1), 75.5 (C5), 73.5 (C3), 70.9 (C2), 68.8 (C4), 61.0 (C6); HRMS *m/z* (ES^+) Found: $(M+NH_4)^+$ 274.1295, $C_{12}H_{20}O_6N$ requires M^+ 274.1290. Data matched those reported previously.[8]

Ethyl 2,3,4,6-tetra-*O*-acteyl-β-D-galactopyranoside S6

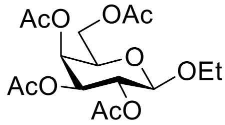

1,2,3,4,6-Penta-*O*-acetyl-α/β-D-galactopyranoside (2.00 g, 5.12 mmol, 1.0 equiv.) was dissolved in DCM (10 mL) and HBr in acetic acid (5 mL) 33% (*w/v*) added dropwise at 0 °C. The mixture was allowed to reach RT and stirred until complete consumption of the starting material, as seen by TLC (R_f = 0.23, hexane/EtOAc, 7:3). The reaction mixture was diluted with DCM (100 mL) and washed successively with saturated aqueous $NaHCO_3$ (3 × 100 mL). The combined organic layers were dried ($MgSO_4$), filtered and evaporated under reduced pressure to deliver 1-bromo-2,3,4,6-tetra-*O*-acetyl-α-D-galactopyranoside as a colourless oil which was used in the next step without further purification. Ethanol (40 mL) was added to 1-bromo-2,3,4,6-tetra-*O*-acetyl-α-D-galactopyranosyl (5.12 mmol, 1.0 equiv.) followed by silver triflate (1.32 g, 5.12 mmol, 1.0 equiv.) and silver carbonate (1.41 g, 5.12 mmol, 1.0 equiv.). The suspension was stirred for 20 hours at RT, TLC analysis revealed reaction completion (R_f = 0.33, hexane/EtOAc, 7:3) and the mixture was diluted with DCM (100 mL) and filtered through a pad of Celite® to remove the silver salts. The solution was concentrated, and the yellow residue was purified by column chromatography (hexane/EtOAc, 0-40%) to yield the title compound **S6** as a clear oil (1.48 g, 3.94 mmol, 77%). R_f= 0.33 (hexane/EtOAc, 7:3); ^{1}H NMR (400 MHz, $CDCl_3$) δ 5.39 (dd, *J* = 3.4, 1.0 Hz, 1H, H-4), 5.20 (dd, *J* = 10.5, 8.0 Hz, 1H, H-2), 5.02 (dd, *J* = 10.5, 3.4 Hz, 1H, H-3), 4.48 (d, *J* = 8.0 Hz, 1H, H-1), 4.19 (dd, *J* = 11.2, 6.5 Hz, 1H, H-6a), 4.13 (dd, *J* = 11.2, 6.9 Hz, 1H, H-6b), 3.98 – 3.86 (m, 2H, H-5, OC*H*H), 3.59 (dq, *J* = 9.7, 7.1 Hz, 1H, OC*H*H), 2.15 (s, 3H, OAc), 2.06 (s, 3H, OAc), 2.05 (s, 3H, OAc), 1.99 (s, 3H, OAc), 1.22 (t, *J* = 7.1 Hz, 3H, CH_3); $^{13}C\{^1H\}$ NMR (101 MHz, $CDCl_3$) δ 170.4 (C=O, Ac), 170.3 (C=O, Ac), 170.2 (C=O, Ac), 169.5 (C=O, Ac), 101.1 (C1), 71.0 (C3), 70.6 (C5), 69.0 (C2), 67.1 (C4), 65.7 (CH_2), 61.3 (C6), 20.8 (Ac-CH_3), 20.7 (Ac-CH_3), 20.6 (Ac-CH_3), 15.1 (CH_3); HRMS *m/z* (ES^+) Found: $(M+NH_4)^+$ 394.1709, $C_{16}H_{28}O_{10}N$ requires M^+ 394.1708. Data matched those reported previously.[9]

Ethyl β-D-galactopyranoside 40

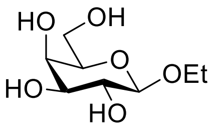

Following the general deacetylation procedure, ethyl 2,3,4,6-tetra-*O*-acteyl-β-D-galactopyranoside **S6** (366 mg, 0.973 mmol, 1.0 equiv.) was deprotected using Na (2.00 mg, 97.0 µmol, 0.1 equiv.) in MeOH (5 mL). Following column chromatography (DCM/MeOH, 0-20%) the title **40** compound was obtained as a colourless oil (172 mg, 0.83 mmol, 85%). R_f = 0.44 (DCM/MeOH, 8:2); ^{1}H NMR (400 MHz, MeOD) δ 4.21 (d, *J* = 7.2 Hz, 1H, H-1), 3.95 (dq, *J* = 9.5, 7.1 Hz, 1H, OC*H*H), 3.82 (d, *J* = 3.0 Hz, 1H, H-4), 3.75 (dd, *J* = 9.8, 5.2 Hz, 1H,

H-6a), 3.71 (dd, J = 9.8, 4.1 Hz, 1H, H-6b), 3.61 (dq, J = 9.6, 7.1 Hz, 1H, OCHH), 3.52 – 3.43 (m, 3H, H-2, H-3, H-5), 1.23 (t, J = 7.1 Hz, 1H, CH_3); $^{13}C\{^1H\}$ NMR (101 MHz, MeOD) δ 103.3 (C1), 75.2 (C3), 73.6 (C5), 71.1 (C2), 68.9 (C4), 64.7 (CH_2), 61.1 (C6), 14.1 (CH_3); HRMS *m/z* (ES^-) Found: $(M-H)^-$ 207.0875, $C_8H_{15}O_6$ requires M^- 207.0874. Data matched those reported previously.[10]

Ethyl α-D-galactopyranoside 7

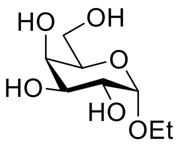

1,2,3,4,6-Penta-*O*-acetyl-α/β-D-galactopyranoside (1.00 g, 2.56 mmol, 1.0 equiv.) was dissolved in DCM (25 mL). Ethanol (5 mL) and $SnCl_4$ (293 µL, 2.56 mmol, 1.0 equiv.) were added at RT and stirred until reaction completion, as seen by TLC (R_f = 0.23, hexane/EtOAc, 7:3). The reaction mixture was diluted with DCM (100 mL) and washed successively with saturated aqueous $NaHCO_3$ (3 × 100 mL). The combined organic layers were dried ($MgSO_4$), filtered and evaporated under reduced pressure. The crude residue was purified by column chromatography (hexane/EtOAc, 0-30%) yielding ethyl 2,3,4,6-tetra-*O*-acteyl-α/β-D-galactopyranoside (530 mg, 1.41 mmol, 55%, α/β, 7:3) as an inseparable mixture. The mixture was deprotected following the general deacetylation procedure, using Na (3.24 mg, 141 µmol, 0.1 equiv.) in MeOH (5 mL) and following column chromatography (DCM/MeOH, 0-20%) a small amount of the title compound **7** was crystallized from acetone as a white solid (32.0 mg, 0.15 mmol, 11%). R_f = 0.44 (DCM/MeOH, 8:2); ^1H NMR (400 MHz, MeOD) δ 4.84 (d, J = 3.2 Hz, 1H, H-1), 3.92 – 3.89 (m, 1H, H-3), 3.85 – 3.80 (m, 2H, H-5, OCHH), 3.79 – 3.74 (m, 2H, H-2, H-4), 3.74 – 3.69 (m, 2H, H-6a, H-6b), 3.58 – 3.48 (m, 1H, OCHH), 1.26 (t, J = 7.1 Hz, 3H, CH_3); $^{13}C\{^1H\}$ NMR (101 MHz, MeOD) δ 98.7 (C1), 70.9 (C5), 70.2 (C2), 69.7 (C3), 68.8 (C4), 63.1 (CH_2), 61.4 (C6), 13.9 (CH_3); HRMS *m/z* (ES^-) Found: $(M-H)^-$ 207.0875, $C_8H_{15}O_6$ requires M^- 207.0874. Data matched those reported previously.[10]

Cyclohexyl 1-thio-β-D-galactopyranoside 43

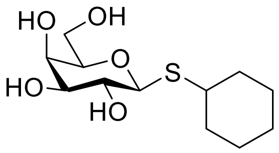

1,2,3,4,6-Penta-*O*-acetyl-α/β-D-galactopyranoside (1.00 g, 2.56 mmol, 1.0 equiv.) was dissolved in DCM (10 mL) and HBr in acetic acid [3 mL, 33% (*w/v*)] added dropwise at 0 °C. The mixture was allowed to reach RT and stirred until complete consumption of the starting material, as seen by TLC (R_f = 0.23, hexane/EtOAc, 7:3). The reaction mixture was diluted with DCM (100 mL) and washed with saturated aqueous $NaHCO_3$ (3 × 100 mL). The combined organic layers were dried ($MgSO_4$), filtered and evaporated under reduced pressure to deliver 1-bromo-2,3,4,6-tetra-*O*-acetyl-α-D-galactopyranoside as a colourless oil which was used in the next step without further purification. To the glycosyl bromide in acetone (20 mL)

was added NaOH (102 mg, 2.56 mmol, 1.0 equiv.) and cyclohexyl mercaptan (345 μL, 2.82 mmol, 1.1 equiv.) and the mixture stirred at RT. After 2 hours TLC analysis revealed complete consumption of the starting material (R_f = 0.20, hexane/EtOAc, 7:3). The mixture was diluted with DCM (50 mL) washed with saturated aqueous $NaHCO_3$ (3 × 100 mL) and the combined organic layers dried ($MgSO_4$), filtered and evaporated under reduced pressure. The crude residue was purified by column chromatography (hexane/EtOAc, 0-40%) yielding cyclohexyl 2,3,4,6-tetra-*O*-acteyl-1-thio-β-D-galactopyranoside as a colourless oil (526 mg, 1.18 mmol, 46%). Following the general deacetylation procedure, cyclohexyl 2,3,4,6-tetra-*O*-acteyl-1-thio-β-D-galactopyranoside (526 mg, 1.18 mmol, 1.0 equiv.) was deprotected using Na (2.71 mg, 0.118 mmol, 0.1 equiv.) in MeOH (5 mL), following column chromatography (DCM/MeOH, 0-20%) the title compound **43** was obtained as a colourless solid (261 mg, 0.94 mmol, 79%); R_f = 0.69 (DCM/MeOH, 8:2); ^{1}H NMR (400 MHz, MeOD) δ 4.40 (d, *J* = 9.1 Hz, 1H, H-1), 3.87 (d, *J* = 3.0 Hz, 1H, H-4), 3.78 – 3.63 (m, 2H, H-6a, H-6b), 3.58 – 3.43 (m, 3H, H-2, H-3, H-5), 3.08 – 2.91 (m, 1H, CH), 2.13 – 1.93 (m, 2H, CH_2), 1.80 – 1.66 (m, 2H, CH_2), 1.65 – 1.50 (m, 1H, C*H*H), 1.47 – 1.17 (m, 5H, 2 × CH_2, C*H*H); ^{13}C{^{1}H} NMR (101 MHz, $CDCl_3$) δ 89.5 (C1), 83.0, 78.9, 74.2, 73.0 (C4), 65.2 (C6), 46.7 (CH), 38.0 (CH_2), 37.8 (CH_2), 29.7 (CH_2), 29.5 (2 × CH_2); HRMS *m/z* (ES^-) Found: $(M-H)^-$ 277.1119, $C_{12}H_{21}O_5S$ requires M^- 277.1119. Data matched those reported previously.[11]

Trifluoroethyl 2,3,4,6-tetra-*O*-acetyl-1-thio-β-D-galactopyranoside S7

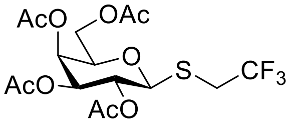

1,2,3,4,6-Penta-*O*-acetyl-α/β-D-galactopyranoside (3.00 g, 7.70 mmol, 1.0 equiv.) was dissolved in DCM (10 mL) and HBr in acetic acid [5 mL, 33% (*w/v*)] added dropwise at 0 °C. The mixture was allowed to reach RT and stirred until complete consumption of the starting material, as seen by TLC (R_f = 0.23, hexane/EtOAc, 7:3). The reaction mixture was diluted with DCM (100 mL) and washed with saturated aqueous $NaHCO_3$ (3 × 100 mL). The combined organic layers were dried ($MgSO_4$), filtered and evaporated under reduced pressure to deliver 1-bromo-2,3,4,6-tetra-*O*-acetyl-α-D-galactopyranoside as a colourless oil which was used in the next step without further purification. To the glycosyl bromide in acetone (20 mL) was added NaOH (308 mg, 7.70 mmol, 1.0 equiv.) and trifluoroethanethiol (759 μL, 8.50 mmol, 1.1 equiv.) and the mixture stirred at RT. After 2 hours TLC analysis revealed complete consumption of the starting material (R_f = 0.20, hexane/EtOAc, 7:3). The mixture was diluted with DCM (50 mL) washed with saturated aqueous $NaHCO_3$ (3 × 100 mL) and the combined organic layers dried ($MgSO_4$), filtered and evaporated under reduced pressure. The crude residue was purified by column chromatography (hexane/EtOAc, 0-40%) yielding the title compound **S7** as a colourless oil (1.49 g, 3.34 mmol, 43%). R_f = 0.20 (hexane/EtOAc, 7:3), $[\alpha]_D^{23}$ = +14.8 (*c* = 1.0, $CHCl_3$); ^{1}H NMR (400 MHz, $CDCl_3$) δ 5.38 (dd, *J* = 3.3, 1.0 Hz, 1H, H-4), 5.12 (t, *J* = 9.9 Hz, 1H, H-2), 4.99 (dd, *J* = 10.0, 3.4 Hz, 1H, H-3), 4.57 (d, *J* = 9.9 Hz, 1H, H-1), 4.11 (dd, *J* = 11.4, 6.9 Hz, 1H, H-6a), 4.05 (dd, *J* = 11.4, 6.3 Hz, 1H, H-6b), 3.91 – 3.87 (m, 1H, H-5), 3.35 (dq, *J* = 15.4, 9.7 Hz, 1H, SC*H*H), 3.08 (dq, *J* = 15.4, 10.0 Hz, 1H,

SC*H*H), 2.10 (s, 3H, OAc), 2.01 (s, 3H, OAc), 1.98 (s, 3H OAc), 1.92 (s, 3H, OAc); $^{13}C\{^1H\}$ NMR (101 MHz, $CDCl_3$) δ 170.4 (C=O, Ac), 170.1 (C=O, Ac), 170.0 (C=O, Ac) 169.7 (C=O, Ac), 125.2 (q, $^1J_{C\text{-}F}$ = 276.2 Hz, CF_3), 82.8 (C1), 74.8 (C5), 71.6 (C3), 67.5 (C2), 67.1 (C4), 61.4 (C6), 31.7 (q, $^2J_{C\text{-}F}$ = 33.7 Hz, CH_2), 20.64 (Ac-CH_3), 20.62 (Ac-CH_3), 20.58 (Ac-CH_3), 20.5 (Ac-CH_3); ^{19}F NMR (376 MHz, $CDCl_3$) δ -66.19 (t, *J* = 9.8 Hz); HRMS *m/z* (ES^+) [Found: $(M+NH_4)^+$ 464.1187, $C_{16}H_{25}F_3O_9SN$ requires M^+ 464.1202.

Trifluoroethyl 1-thio-β-D-galactopyranoside 45

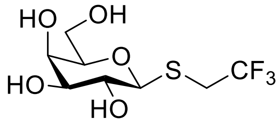

Following the general deacetylation procedure, trifluoroethyl 2,3,4,6-tetra-*O*-acetyl-1-thio-β-D-galactopyranoside **S7** (740 mg, 1.66 mmol, 1.0 equiv.) was deprotected using Na (3.82 mg, 0.166 mmol, 0.1 equiv.) in MeOH (5 mL), following column chromatography (DCM/MeOH, 0-20%) the title compound **45** was obtained as a colourless oil (398 mg, 1.43 mmol, 86%). R_f = 0.35 (DCM/MeOD, 8:2); $[\alpha]_D^{23}$ = -6.5 (*c* = 1.0, H_2O); ^1H NMR (400 MHz, MeOD) δ 4.45 (d, *J* = 9.3 Hz, 1H, H-1), 3.88 (dd, *J* = 3.2, 1.0 Hz, 1H, H-4), 3.77 (dd, *J* = 11.5, 7.1 Hz, 1H, H-6a), 3.69 (dd, *J* = 11.5, 4.9 Hz, 1H, H-6b), 3.65 – 3.56 (m, 1H, SC*H*H), 3.56 – 3.49 (m, 2H, H-2, H-5), 3.46 (dd, *J* = 9.2, 3.3 Hz, 1H, H-3), 3.39 – 3.32 (m, 1H, SC*H*H); $^{13}C\{^1H\}$ NMR (101 MHz, MeOD) δ 126.1 (q, $^1J_{C\text{-}F}$ = 284.4 Hz, CF_3), 84.6 (C1), 79.5 (C5), 74.7 (C3), 70.6 (C2), 69.1 (C4), 61.3 (C6), 30.1 (q, $^2J_{C\text{-}F}$ = 32.9 Hz, CH_2); ^{19}F NMR (376 MHz, MeOD) δ -67.8 (t, *J* = 10.4 Hz); HRMS *m/z* (ES^+) Found: $(M+Na)^+$ 301.0322, $C_8H_{13}F_3O_5SNa$ requires $C_8H_{13}F_3O_5SNa$ M^+ 301.0333.

Trifluoroethyl 2,3,4,6-tetra-*O*-acetyl-β-D-galactopyranoside S8

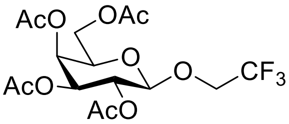

1,2,3,4,6-Penta-*O*-acetyl-α/β-D-galactopyranoside (2.00 g, 5.12 mmol, 1.0 equiv.) was dissolved in DCM (10 mL) and HBr in acetic acid [5 mL, 33% (*w/v*)] added dropwise at 0 °C. The mixture was allowed to reach RT and stirred until complete consumption of the starting material, as seen by TLC (R_f = 0.23, hexane/EtOAc, 7:3). The reaction mixture was diluted with DCM (100 mL) and washed with saturated aqueous $NaHCO_3$ (3 × 100 mL). The combined organic layers were dried ($MgSO_4$), filtered and evaporated under reduced pressure to deliver 1-bromo-2,3,4,6-tetra-*O*-acetyl-α-D-galactopyranoside as a colourless oil which was used in the next step without further purification. To the glycosyl bromide was added trifluoroethanol (405 μL, 5.63 mmol, 1.1 equiv.) followed by silver triflate (1.32 g, 5.12 mmol, 1.0 equiv.) and silver carbonate (1.41 g, 5.12 mmol, 1.0 equiv.). The suspension was stirred for 20 hours at RT, TLC analysis revealed reaction completion (R_f= 0.44, hexane/EtOAc, 1:1) and the reaction mixture was diluted with DCM (50 mL) and filtered through a pad of Celite® to remove the silver salts. The solution was concentrated under reduced pressure, and the crude

residue purified by column chromatography (hexane/EtOAc, 0-50%) to yield the title compound **S8** as a colourless oil (1.10 g, 2.56 mmol, 50%). R_f = 0.44 (hexane/EtOAc, 1:1); $[\alpha]_D^{23}$ = +14.0 (*c* = 1.0, $CHCl_3$); ^{1}H NMR (400 MHz, $CDCl_3$) δ 5.41 (dd, *J* = 3.4, 1.0 Hz, 1H, H-4), 5.24 (dd, *J* = 10.5, 7.9 Hz, 1H, H-2), 5.04 (dd, *J* = 10.5, 3.4 Hz, 1H, H-3), 4.62 (d, *J* = 7.9 Hz, 1H, H-1), 4.22 – 4.06 (m, 3H, H-6a, H-6b, OC*H*H), 4.06 – 3.91 (m, 2H, H-5, OC*H*H), 2.17 (s, 3H, OAc), 2.07 (s, 3H, OAc), 2.06 (s, 3H, OAc), 1.99 (s, 3H, OAc); ^{13}C{^{1}H} NMR (101 MHz, $CDCl_3$) δ 170.4 (C=O, Ac), 170.14 (C=O, Ac), 170.06 (C=O, Ac), 169.4 (C=O, Ac), 123.4 (q, $^1J_{C\text{-}F}$ = 278.7 Hz, CF_3), 101.3 (C1), 71.1 (C5), 70.5 (C3), 68.2 (C2), 66.8 (C4), 65.8 (t, $^2J_{C\text{-}F}$ = 35.0 Hz, CH_2), 61.2 (C6), 20.62 (Ac-CH_3), 20.61 (Ac-CH_3), 20.52 (Ac-CH_3), 20.47 (Ac-CH_3); ^{19}F NMR (376 MHz, $CDCl_3$) δ -74.4 (t, *J* = 8.5 Hz); HRMS *m/z* (ES^+) Found: $(M+NH_4)^+$ 448.1417, $C_8H_{17}F_3O_6N$ requires M^+ 448.1430.

Trifluoroethyl 2,3,4,6-tetra-*O*-acetyl-β-D-galactopyranoside 54

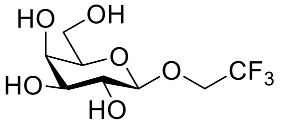

Following the general deacetylation procedure, trifluoroethyl 2,3,4,6-tetra-*O*-acetyl-β-D-galactopyranoside **S8** (450 mg, 1.05 mmol, 1.0 equiv.) was deprotected using Na (2.42 mg, 0.105 mmol, 0.1 equiv.) in MeOH (5 mL), following column chromatography (DCM/MeOH, 0-20%) the title compound **54** was obtained as a colourless oil (198 mg, 0.76 mmol, 72%). R_f = 0.30 (DCM/MeOD, 8:2); $[\alpha]_D^{23}$ = -3.7 (*c* = 1.0, H_2O); ^{1}H NMR (400 MHz, MeOD) δ 4.34 (d, *J* = 7.6 Hz, 1H, H-1), 4.26 (dq, *J* = 12.3, 9.1 Hz, 1H, OC*H*H), 4.09 (dq, *J* = 12.3, 8.9 Hz, 1H, OC*H*H), 3.83 (dd, *J* = 3.3, 0.9 Hz, 1H, H-4), 3.78 (dd, *J* = 11.4, 7.1 Hz, 1H, H-6a), 3.72 (dd, *J* = 11.4, 5.0 Hz, 1H, H-6b), 3.58 – 3.51 (m, 2H, H-2, H-5), 3.47 (dd, *J* = 9.7, 3.4 Hz, 1H, H-3); ^{13}C{^{1}H} NMR (101 MHz, MeOD) δ 124.1 (q, $^1J_{C\text{-}F}$ = 277.1 Hz, CF_3), 103.4 (C1), 75.6 (C5), 73.4 (C3), 70.8 (C2), 68.8 (C4), 65.1 (q, ^2J = 34.7 Hz, CH_2), 61.1 (C6); ^{19}F NMR (376 MHz, MeOD) δ -75.7 (t, *J* = 9.0 Hz); HRMS *m/z* (ES^+) Found: $(M+NH_4)^+$ 280.0994, $C_8H_{17}F_3O_6N$ requires M^+ 280.1007.

Cyclohexyl β-D-galactopyranoside 49

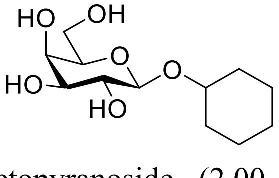

1,2,3,4,6-Penta-*O*-acetyl-α/β-D-galactopyranoside (2.00 g, 5.12 mmol, 1.0 equiv.) was dissolved in DCM (10 mL) and HBr in acetic acid [5 mL, 33% (*w/v*)] added dropwise at 0 °C. The mixture was allowed to reach RT and stirred until complete consumption of the starting material, as seen by TLC (R_f = 0.23, hexane/EtOAc, 7:3). The reaction mixture was diluted with DCM (100 mL) and washed successively with saturated aqueous $NaHCO_3$ (3 × 100 mL). The combined organic layers were dried ($MgSO_4$) and evaporated under reduced pressure to deliver 1-bromo-2,3,4,6-tetra-*O*-acetyl-α-D-galactopyranoside as a colourless oil which was used in the next step without further purification. To the glycosyl bromide was added

cyclohexanol (20 mL, 189 mmol, 36.8 equiv.) followed by silver triflate (1.32 g, 5.12 mmol, 1.0 equiv.) and silver carbonate (1.41 g, 5.12 mmol, 1.0 equiv.). The suspension was stirred for 20 hours at RT, TLC analysis revealed reaction completion (R_f = 0.75, hexane/EtOAc, 1:1). The reaction mixture was diluted with DCM (50 mL) and filtered through a pad of Celite® to remove the silver salts. The solution was concentrated, and the crude residue purified by column chromatography (hexane/EtOAc, 0-50%) to yield cyclohexyl 2,3,4,6-tetra-*O*-acetyl-β-D-galactopyranoside (1.62 g, 3.76 mmol, 73%) as a colourless oil. Following the general deacetylation procedure, cyclohexyl 2,3,4,6-tetra-*O*-acetyl-β-D-galactopyranoside (435 mg, 1.01 mmol, 1.0 equiv.) was deprotected using Na (2.32 mg, 0.101 mmol, 0.1 equiv.) in MeOH (5 mL), following column chromatography (DCM/MeOH, 0-20%) the title compound **49** was obtained as a white solid (215 mg, 0.82 mmol, 81%). R_f = 0.45 (DCM/MeOD, 8:2); ^{1}H NMR (400 MHz, MeOD) δ 4.32 (d, J = 7.3 Hz, 1H, H-1), 3.86 – 3.80 (m, 1H), 3.78 – 3.65 (m, 3H, H-6a, H-6b, CH), 3.54 – 3.41 (m, 3H), 1.94 (s, 2H, CH_2), 1.85 – 1.68 (m, 2H, CH_2), 1.60 – 1.50 (m, 1H, C*H*H), 1.40 – 1.21 (m, 5H, 2 × CH_2, C*H*H); $^{13}C\{^1H\}$ NMR (101 MHz, $CDCl_3$) δ 105.6 (C1), 80.8, 79.1, 77.6, 75.2, 72.8, 65.0 (C6), 37.3 (CH_2), 35.4 (CH_2), 29.4 (CH_2), 27.8 (CH_2), 27.6 (CH_2); HRMS *m/z* (ES^+) Found: $(M+Na)^+$ 285.1308, $C_{12}H_{22}O_6Na$ requires M^+ 285.1314. Data matched those reported previously.[12]

p-(Methoxy)-phenyl α-D-galactopyranoside 13

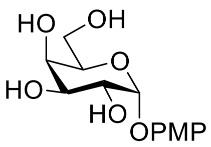

1,2,3,4,6-Penta-*O*-acetyl-α/β-D-galactopyranoside (2.31 g, 5.92 mmol, 1.0 equiv.) was dissolved in DCM (25 mL), *p*-methoxy phenol (634 mg, 5.92 mmol, 1.0 equiv.) and $SnCl_4$ (677 µL, 5.92 mmol, 1.0 equiv.) were then added. The mixture was stirred at RT until reaction completion, as seen by TLC (R_f = 0.36, hexane/EtOAc, 7:3). The reaction mixture was diluted with DCM (100 mL) and washed with saturated aqueous $NaHCO_3$ (3 × 100 mL). The combined organic layers were dried ($MgSO_4$), filtered and evaporated under reduced pressure and the crude residue was purified by column chromatography (hexane/EtOAc, 0-30%) yielding *p*-methoxy phenyl 2,3,4,6-tetra-*O*-acteyl-α-D-galactopyranoside (1.22 g, 2.68 mmol, 45%) as a white foam. R_f = 0.36 (hexane/EtOAc, 7:3). Following the general deacetylation procedure, *p*-methoxy phenyl 2,3,4,6-tetra-*O*-acteyl-α-D-galactopyranoside (858 mg, 1.89 mmol, 1.0 equiv.) was deprotected using Na (4.34 mg, 0.189 mmol, 0.1 equiv.) in MeOH (5 mL), following column chromatography (DCM/MeOH, 0-20%) the title compound **13** was obtained as a white solid (452 mg, 1.58 mmol, 84%). R_f = 0.63 (DCM/MeOH, 8:2); ^{1}H NMR (400 MHz, MeOD) δ 7.12 – 7.07 (m, 2H, Ar*H*), 6.86 – 6.80 (m, 2H, Ar*H*), 5.34 (d, J = 2.9 Hz, 1H, H-1), 4.01 – 3.94 (m, 2H, H-4, H-5), 3.94 – 3.91 (m, 2H, H-2, H-3), 3.73 (s, 3H, OCH_3), 3.74 – 3.70 (m, 1H, H-6a), 3.68 (dd, J = 9.7, 4.9 Hz, 1H, H-6b); $^{13}C\{^1H\}$ NMR (101 MHz, MeOD) δ 155.3 (Ar-C), 151.5 (Ar-C), 118.5 (Ar-C), 114.1 (Ar-C), 99.4 (C1), 71.6, 70.0, 69.5, 68.7, 61.1 (C6), 54.7 (OCH_3); HRMS *m/z* (ES^-) Found: $(M-H)^-$ 285.0981, $C_{13}H_{17}O_7$ requires M^- 285.0980. Data matched those reported previously.[13]

Methyl 6-*O*-triisopropylsilyl-α-D-galactopyranoside 11

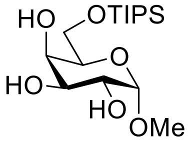

A solution of methyl α-D-galactopyranoside (250 mg, 1.29 mmol, 1.0 equiv.) and imidazole (176 mg, 2.58 mmol, 2.0 equiv.) in pyridine (20 mL) was treated with TIPSCl (552 µL, 2.58 mmol, 2.0 equiv.) and the solution stirred at RT for 24 hours. At this point TLC analysis revealed complete conversion to another spot (R_f = 0.22 (hexane/EtOAc, 1:1) and the reaction was quenched with MeOH (5 mL). The solution was diluted with DCM (100 mL), washed with 1M HCl (2 x 75 mL), saturated aqueous $NaHCO_3$ (100 mL) and water (75 mL). The combined organic phases were dried ($MgSO_4$), filtered and concentrated under reduced pressure. The crude residue was purified by column chromatography (hexane/EtOAc, 0-60%) to deliver the title compound **11** (375 mg, 1.07 mmol, 83%) as a white solid. ^{1}H NMR (400 MHz, $CDCl_3$) δ 4.81 (d, *J* = 3.9 Hz, 1H, H-1), 4.12 – 4.10 (m, 1H, H-4), 3.99 (dd, *J* = 10.4, 5.6 Hz, 1H, H-6a), 3.93 (dd, *J* = 10.3, 5.0 Hz, 1H, H-6b), 3.87 (bs, 1H, H-2), 3.79 – 3.72 (m, 2H, H-3, H-5), 3.42 (s, 3H, OCH_3), 3.31 (s, 1H, 4-OH), 3.20 (s, 1H, 3-OH), 2.56 (s, 1H, 2-OH), 1.10 – 1.03 (m, 21H, ^{i}PrSi); ^{13}C{^{1}H} NMR (101 MHz, $CDCl_3$) δ 99.6 (C1), 71.4 (C3), 70.0 (C5), 69.79 (C2), 69.76 (C4), 63.6, (C6), 55.4 (CH_3), 17.91 (CH_3, ^{i}Pr), 17.89 (CH_3, ^{i}Pr), 11.8 (CH, ^{i}Pr); HRMS *m/z* (ES$^-$) Found: (M-H)$^-$ 349.2056, $C_{16}H_{33}O_6Si$ requires M$^-$ 349.2052. Data matched those reported previously.[14]

Allyl 2-acetamido-2-deoxy-β-D-galactopyranoside 15

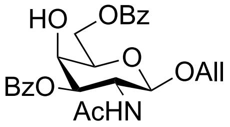

To 1,3,4,6-tetra-*O*-acetyl-2-acetamido-2-deoxy-β-D-galactopyranoside (4.00 g, 10.3 mmol, 1.0 equiv.) in dichloroethane (50 mL) was added $FeCl_3$ (2.50 g, 15.4 mmol, 1.5 equiv.), followed by subsequent addition of allyl alcohol (1.39 mL, 20.5 mmol, 2.0 equiv.). The resulting yellow coloured solution was stirred at RT for 24 hours. At this point reaction completion was confirmed by TLC (R_f = 0.11, EtOAc). Saturated aqueous $NaHCO_3$ (175 mL) was added and the biphasic mixture stirred vigorously at RT for 30 mins. The mixture was diluted with DCM (150 mL), layers separated and the aqueous phase washed with DCM (3 × 150 mL). The combined organic phases were dried over $MgSO_4$, filtered, and concentrated under reduced pressure. To this crude residue was added hexane/EtOAc (1:1, 100 mL) and the reaction flask settled for 15 mins. The resulting solid was filtered, followed by washing with cold hexane (100 mL) to yield allyl 3,4,6-tri-*O*-acetyl-2-acetamido-2-deoxy-β-D-galactopyranoside (2.90 g, 7.50 mmol, 73%) as a white solid. A solution of allyl 3,4,6-tri-*O*-acetyl-2-acetamido-2-deoxy-β-D-galactopyranoside (2.51 g, 6.48 mmol, 1.0 equiv.) in MeOH (10 mL) was treated with 1M NaOH (5 mL) and the reaction mixture was left stirring at RT. After 30 mins TLC analysis revealed full conversion of the starting material to a single lower spot (R_f= 0.35, DCM/MeOH, 8:2). The reaction mixture was neutralised with Amberlite IR120 (H^+) ion exchange resin. The mixture was filtered and washed with MeOH (100 mL), and the filtrate concentrated under

reduced pressure to give an off-white solid. EtOAc (50 mL) was added to the crude residue followed by DCM (150 mL) and the mixture was heated at reflux (50 °C). After 15 mins the round bottom flask was placed into an ice bath and left without stirring for 30 mins giving a white precipitate which was filtered to deliver the title compound **15** as a white solid (1.41 g, 5.38 mmol, 83%). R_f = 0.35 (DCM/MeOH, 8:2); ^{1}H NMR (400 MHz, MeOD) δ 5.89 (dddd, *J* = 15.5, 10.6, 5.7, 5.0 Hz, 1H, =CH-), 5.27 (ddd, *J* = 17.3, 3.5, 1.7 Hz, 1H, CH_2=), 5.12 (dd, *J* = 10.5, 1.8 Hz, 1H, CH_2=), 4.41 (d, *J* = 8.4 Hz, 1H, H-1), 4.33 (ddt, *J* = 13.3, 4.9, 1.6 Hz, 1H, OC*H*H), 4.07 (ddt, *J* = 13.3, 5.8, 1.4 Hz, 1H, OC*H*H), 3.94 (dd, *J* = 10.5, 8.6 Hz, 1H, H-2), 3.83 (d, *J* = 2.4 Hz, 1H, H-4), 3.80 – 3.71 (m, 2H, H-6a, H-6b), 3.59 (dd, *J* = 10.7, 3.2 Hz, 1H, H-3), 3.48 (dd, *J* = 6.6, 5.5 Hz, 1H, H-5), 1.97 (s, 3H, Ac); ^{13}C{^{1}H} NMR (101 MHz, MeOD) δ 172.7 (C=O, NHAc), 134.3 (=CH-), 115.5 (CH_2=), 100.8 (C1), 75.3 (C5), 71.9 (C3), 69.2 (OCH_2), 68.3 (C4), 61.1 (C6), 52.9 (C2), 21.6 (Ac-CH_3); HRMS *m/z* (ES^-) Found: $(M-H)^-$ 260.1134, $C_{11}H_{18}N_1O_6$ requires M^- 260.1140. Data matched those reported previously.[15]

Allyl 2-acetamido-2-deoxy-α-D-galactopyranoside 17

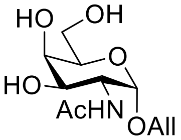

Galactosamine hydrochloride (5.00 g, 23.2 mmol, 1.0 equiv.) and NaOMe (1.51 g, 27.8 mmol, 1.2 equiv.) in MeOH (50 mL) was stirred for 30 mins and filtered. The filtrate was cooled to 0 °C and Ac_2O added (2.41 mL, 25.5 mmol, 1.1 equiv.). After 1 hour of stirring the solvent was removed under reduced pressure and to the crude residue was added allyl alcohol (50 mL). To this mixture was added $BF_3 \cdot OEt_2$ (2.86 mL, 23.2 mmol, 1.0 equiv.) and the reaction heated at 70 °C for 2 hours. TLC analysis revealed full consumption of the starting material (R_f = 0.63, DCM/MeOH, 8:2). The solution was cooled to RT and the solvent was evaporated under reduced pressure. Addition of EtOH (100 mL) precipitated the title compound **17** as a white solid (791 mg, 3.03 mmol, 13%). R_f = 0.63 (DCM/MeOH, 8:2); ^{1}H NMR (400 MHz, D_2O) δ 5.89 (ddd, *J* = 22.5, 10.9, 5.7 Hz, 1H, =CH-), 5.27 (d, *J* = 17.3 Hz, 1H, CH_2=), 5.18 (d, *J* = 10.4 Hz, 1H, CH_2=), 4.87 (d, *J* = 3.7 Hz, 1H, H-1), 4.18 – 4.05 (m, 2H, H-2, OC*H*H), 3.99 – 3.81 (m, 4H, H-3, H-6a, H-6b, OC*H*H), 3.77 – 3.62 (m, 2H, H-4, H-5), 1.96 (s, 3H, Ac); ^{13}C{^{1}H} NMR (101 MHz, D_2O) δ 174.6 (C=O, Ac), 133.7 (=CH-), 117.9 (CH_2=), 96.2 (C1), 71.0, 68.50, 68.46, 67.7, 61.2, 49.9 (C2), 21.9 (Ac-CH_3); HRMS *m/z* (ES^-) Found: $(M-H)^-$ 260.1135, $C_{11}H_{18}N_1O_6$ requires M^- 260.1140. Data matched those reported previously.[16]

p-(Trifluoromethyl)-phenyl 2,3,4,6-tetra-*O*-acetyl-1-thio-β-D-galactopyranoside S9

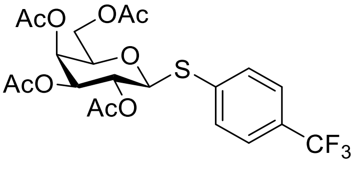

1,2,3,4,6-Penta-*O*-acetyl-α/β-D-galactopyranoside (3.00 g, 7.70 mmol, 1.0 equiv.) was dissolved in DCM (10 mL) and HBr in acetic acid [5 mL, 33% (*w/v*)] added dropwise at 0 °C. The mixture was allowed to reach RT and stirred until complete consumption of the starting material, as seen by TLC (R_f = 0.23, hexane/EtOAc, 7:3). The reaction mixture was diluted with DCM (100 mL) and washed successively with saturated aqueous $NaHCO_3$ (3 × 100 mL). The combined organic layers were dried ($MgSO_4$), filtered and evaporated under reduced pressure to deliver 1-bromo-2,3,4,6-tetra-*O*-acetyl-α-D-galactopyranoside as a colourless oil which was used in the next step without further purification. To the glycosyl bromide (1.15 g, 2.55 mmol, 1.0 equiv.) in acetone (7 mL) was added NaOH (100 mg, 2.55 mmol, 1.0 equiv.) and 4-trifluoromethylthiophenol (390 µL, 2.83 mmol, 1.1 equiv.) and the mixture stirred at RT. After 2 hours TLC analysis revealed complete consumption of the starting material (R_f = 0.47, hexane/EtOAc, 7:3). The mixture was diluted with DCM (20 mL) washed with saturated aqueous $NaHCO_3$ (3 × 35 mL) and the combined organic layers dried ($MgSO_4$), filtered and evaporated under reduced pressure. The crude residue was purified by column chromatography (hexane/EtOAc, 7:3) yielding the title compound **S9** compound as an off white solid (1.20 g, 2.36 mmol, 92%). R_f = 0.47 (hexane/EtOAc, 7:3); Melting point: 103-104°C; $[\alpha]_D^{22}$ = -3.7 (*c* = 1.0, MeOH); ^{1}H NMR (400 MHz, $CDCl_3$) δ 7.58 (q, *J* = 8.5 Hz, 4H, Ar*H*), 5.43 (dd, *J* = 3.3, 1.1 Hz, 1H, H-4), 5.24 (t, *J* = 10.0 Hz, 1H, H-2), 5.06 (dd, *J* = 9.9, 3.3 Hz, 1H, H-3), 4.77 (d, *J* = 9.9 Hz, 1H, H-1), 4.22 – 4.08 (m, 2H, H-6a, H-6b), 3.99 (m, 1H, H-5), 2.11 (s, 3H, OAc), 2.08 (s, 3H, OAc), 2.04 (s, 3H, OAc), 1.97 (s, 3H, OAc); ^{13}C{^{1}H} NMR (101 MHz, $CDCl_3$) δ) 170.4 (C=O, Ac), 170.2 (C=O, Ac), 170.1 (C=O, Ac) 169.5 (C=O, Ac), 137.6 (Ar-C), 131.9 (Ar-C), 130.6 (Ar-C), 130.2 (Ar-C), 129.9 (Ar-C), 129.6 (Ar-C), 125.8 (Ar-C), 125.7 (Ar-C), 124.01 (q, $^1J_{C\text{-}F}$ = 272.0 Hz), 85.6 (C1), 74.8 (C5), 72.0 (C3), 67.2 (C4), 67.1 (C2), 61.8 (C6), 20.9 (Ac-CH_3), 20.8 (Ac-CH_3), 20.7 (Ac-CH_3); ^{19}F NMR (376 MHz, $CDCl_3$) δ -62.7; HRMS *m/z* (ES^+) Found $(M+NH_4)^+$ 526.1353, $C_{21}H_{27}F_3O_9SN$ requires M^+ 526.1353.

p-(Nitro)-phenyl 2,3,4,6-tetra-*O*-acetyl-1-thio-β-D-galactopyranoside S10

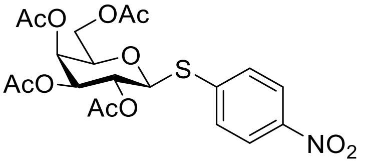

1,2,3,4,6-Penta-*O*-acetyl-α/β-D-galactopyranoside (3.00 g, 7.70 mmol, 1.0 equiv.) was dissolved in DCM (10 mL) and HBr in acetic acid [5 mL, 33% (*w/v*)] added dropwise at 0 °C. The mixture was allowed to reach RT and stirred until complete consumption of the starting material, as seen by TLC (R_f = 0.23, hexane/EtOAc, 7:3). The reaction mixture was diluted with DCM (100 mL) and washed with saturated aqueous $NaHCO_3$ (3 × 100 mL). The combined organic layers were dried ($MgSO_4$), filtered and evaporated under reduced pressure to deliver 1-bromo-2,3,4,6-tetra-*O*-acetyl-α-D-galactopyranoside as a colourless oil which was used in the next step without further purification. To the glycosyl bromide (1.15 g, 2.55 mmol, 1.0 equiv.) in acetone (7 mL) was added NaOH (100 mg, 2.55 mmol, 1.0 equiv.) and 4-nitrothiophenol (439 mg, 2.83 mmol, 1.1 equiv.) and the mixture stirred at RT. After 2 hours TLC analysis revealed complete consumption of the starting material (R_f = 0.28, hexane/EtOAc, 7:3). The mixture was diluted with DCM (20 mL) washed with saturated

aqueous $NaHCO_3$ (3 × 35 mL) and the combined organic layers dried ($MgSO_4$), filtered and evaporated under reduced pressure. The crude residue was purified by column chromatography (hexane/EtOAc, 7:3) yielding the title compound **S10** as a yellow solid (1.00 g, 2.06 mmol, 81%); R_f = 0.28 (hexane/EtOAc, 7:3); ^{1}H NMR (400 MHz, $CDCl_3$) δ 8.16 (m, 2H, Ar*H*), 7.61 (m, 2H, Ar*H*), 5.47 (dd, *J* = 3.4, 1.2 Hz, 1H, H-4), 5.29 (t, *J* = 10.0 Hz, 1H, H-2), 5.10 (dd, *J* = 10.0, 3.4 Hz, 1H, H-3), 4.86 (d, *J* = 9.9 Hz, 1H, H-1), 4.17 (m, 2H, H-6a, H-6b), 4.03 (m, 1H, H-5), 2.16 (s, 3H, OAc), 2.08 (s, 3H, OAc), 2.07 (s, 3H, OAc), 1.98 (s, 3H, OAc); ^{13}C{^{1}H} NMR (101 MHz, $CDCl_3$) δ, 170.4 (C=O, OAc), 170.2 (C=O, OAc), 170.1 (C=O, OAc) 169.5 (C=O, OAc), 147.0 (Ar-C), 142.5 (Ar-C), 130.6 (Ar-C), 126.5 (Ar-C), 124.6 (Ar-C), 124.0 (Ar-C), 85.0 (C1), 75.0 (C5), 71.9 (C3), 67.2 (C4), 66.9 (C2), 61.8 (C6), 20.9 (OAc-CH_3), 20.8 (OAc -CH_3), 20.7 (OAc -CH_3); HRMS *m/z* (ES^+) Found $(M+NH_4)^+$ 503.1325, $C_{20}H_{27}NO_{11}SN$ requires M^+ 503.1330. Data matched those reported previously.[17]

p-(Methoxy)-phenyl 2,3,4,6-tetra-*O*-acetyl-1-thio-β-D-galactopyranoside S11

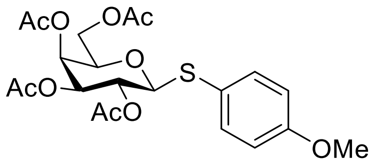

1,2,3,4,6-Penta-*O*-acetyl-α/β-D-galactopyranoside (3.00 g, 7.70 mmol, 1.0 equiv.) was dissolved in DCM (10 mL) and HBr in acetic acid [5 mL, 33% (*w/v*)] added dropwise at 0 °C. The mixture was allowed to reach RT and stirred until complete consumption of the starting material, as seen by TLC (R_f = 0.23, hexane/EtOAc, 7:3). The reaction mixture was diluted with DCM (100 mL) and washed successively with saturated aqueous $NaHCO_3$ (3 × 100 mL). The combined organic layers were dried ($MgSO_4$), filtered and evaporated under reduced pressure to deliver 1-bromo-2,3,4,6-tetra-*O*-acetyl-α-D-galactopyranoside as a colourless oil which was used in the next step without further purification. To the glycosyl bromide (1.15 g, 2.55 mmol, 1.0 equiv.) in acetone (7 mL) was added NaOH (100 mg, 2.55 mmol, 1.0 equiv.) and 4-methoxythiophenol (390 µL, 2.83 mmol, 1.1 equiv.) and the mixture stirred at RT. After 2 hours TLC analysis revealed complete consumption of the starting material (R_f = 0.26, hexane/EtOAc, 7:3). The mixture was diluted with DCM (20 mL) washed with saturated aqueous $NaHCO_3$ (3 × 35 mL) and the combined organic layers dried ($MgSO_4$), filtered and evaporated under reduced pressure. The crude residue was purified by column chromatography (hexane/EtOAc, 7:3) yielding the title **S11** compound as a white solid (950 mg, 2.02 mmol, 79%); R_f= 0.26 (hexane/EtOAc, 7:3); ^{1}H NMR (400 MHz, $CDCl_3$) δ 7.44 (dt, *J* = 8.6, 3.3 Hz, 2H, Ar*H*), 6.82 (dt, *J* = 8.9, 3.3 Hz, 2H, Ar*H*), 5.36 (dd, *J* = 3.3, 1.2 Hz, 1H, H-4), 5.14 (t, *J* = 10.0 Hz, 1H, H-2), 5.00 (dd, *J* = 9.9, 3.3 Hz, 1H, H-3), 4.54 (d, *J* = 9.9 Hz, 1H, H-1), 4.15 (dd, *J* = 11.4, 6.8 Hz, 1H, H-6a), 4.07 (dd, *J* = 11.2, 6.4 Hz, 1H, H-6b), 3.86 (td, *J* = 6.5, 1.2 Hz, 1H, H-5), 3.78 (s, 3H, OCH_3), 2.08 (s, 3H, OAc), 2.06 (s, 3H, OAc), 2.01 (s, 3H, OAc), 1.94 (s, 3H, OAc); ^{13}C{^{1}H} NMR (101 MHz, $CDCl_3$) δ 170.4 (C=O, OAc), 170.2 (C=O, OAc), 170.1 (C=O, OAc), 169.5 (C=O, OAc), 160.3 (Ar-C), 136.0 (Ar-C), 122.1 (Ar-C), 114.4 (Ar-C), 87.0 (C1), 74.3 (C5), 72.1 (C3), 67.4 (C4), 67.3 (C2), 61.6 (C6), 55.4 (OCH_3), 20.9 (Ac-CH_3), 20.7 (Ac-CH_3), 20.7 (Ac-CH_3), 20.6 (OAc-CH_3). HRMS *m/z* (ES^+) Found $(M+NH_4)^+$ 488.1588, $C_{21}H_{30}O_{10}SN$ requires M^+ 488.1585. Data matched those reported previously.[18]

p-(Trifluoromethyl)-phenyl 1-thio-β-D-galactopyranoside 57

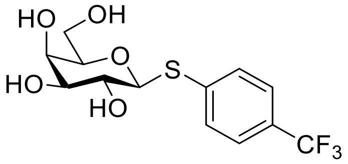

p-(Trifluoromethyl)-phenyl 2,3,4,6-tetra-*O*-acetyl-1-thio-β-D-galactopyranoside (1.04 g, 2.04 mmol, 1.0 equiv.) was deprotected using Na_2CO_3 (75.0 mg, 0.71 mmol, 0.35 equiv.) in MeOH (8 mL). After 3 hours TLC analysis revealed complete consumption of the starting material (R_f = 0.41 9:1 DCM/MeOH), Amberlite IR120 (H^+) ion exchange resin was added and the mixture stirred until neutral (confirmed using Johnson® pH paper) after which the mixture was filtered and washed with MeOH (100 mL). The combined organic filtrates were concentrated under reduced pressure. Following column chromatography (DCM/MeOH, 0-20%) the title compound **57** was obtained as a white solid (590 mg, 1.73 mmol, 84%); R_f = 0.41 (9:1 DCM/MeOH); $[\alpha]_D^{22}$ = -46.2 (*c* = 1.0, MeOH); ^{1}H NMR (400 MHz, MeOD) δ 7.67 (d, *J* = 8.1 Hz, 1H, Ar*H*), 7.55 (d, *J* = 8.2 Hz, 1H, Ar*H*), 4.73 (d, *J* = 9.8 Hz, 1H, H-1), 3.92 (d, *J* = 3.4 Hz, 1H, H-4), 3.78 (dd, *J* = 11.5, 7.1 Hz, 1H, H-6a), 3.74 – 3.70 (m, 1H, H-6b), 3.70 – 3.61 (m, 2H, H-2, H-5), 3.53 (dd, *J* = 9.2, 3.3 Hz, 1H, H-3); $^{13}C\{^1H\}$ NMR (101 MHz, MeOD) δ 142.2 (Ar-C), 130.8 (Ar-C), 129.7 (Ar-C), 129.4 (Ar-C), 129.1 (Ar-C), 126.6 (Ar-C), 126.5 (Ar-C), 126.4 (Ar-C), (125.6, q, $^1J_{C-F}$ = 270 Hz), 88.9 (C1), 80.7 (C5), 76.3 (C3), 70.8 (C2), 70.4 (C4), 62.7 (C6); ^{19}F NMR (376 MHz, $CDCl_3$) δ -63.9; HRMS *m/z* (ES^+) Found $(M+NH_4)^+$ 358.0940, $C_{13}H_{19}F_3O_5SN$ requires M^+ 358.0930.

p-(Nitro)-phenyl 1-thio-β-D-galactopyranoside 67

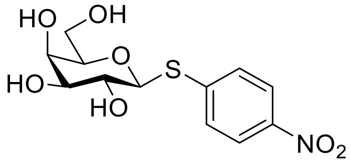

p-(Nitro)-phenyl 2,3,4,6-tetra-*O*-acetyl-1-thio-β-D-galactopyranoside (1.00 g, 2.06 mmol, 1.0 equiv.) was deprotected using Na_2CO_3 (75.0 mg, 0.71 mmol, 0.35 equiv.) in MeOH (8 mL). After 3 hours TLC analysis revealed complete consumption of the starting material (R_f = 0.34, 9:1 DCM/MeOH,) Amberlite IR120 (H^+) ion exchange resin was added, and the mixture stirred until neutral (confirmed using Johnson® pH paper) after which the mixture was filtered and washed with MeOH (100 mL). The combined organic filtrates were concentrated under reduced pressure. Following column chromatography (DCM/MeOH, 0-20%) the title compound **67** was obtained as a pale yellow solid (498 mg, 1.57 mmol, 76%); R_f = 0.41 (9:1 DCM/MeOH); ^{1}H NMR (400 MHz, MeOD) δ 8.13 (m, 2H, Ar*H*), 7.66 (m, 2H, Ar*H*), 4.84 (d, *J* = 9.7 Hz, 1H, H-1), 3.95 (d, *J* = 3.3 Hz, 1H, H-4), 3.83 – 3.68 (m, 4H, H-2, H-5, H-6), 3.57 (dd, *J* = 9.2, 3.3 Hz, 1H, H-3); $^{13}C\{^1H\}$ NMR(101 MHz, MeOD) δ 147.1 (Ar-C), 147.0 (Ar-C), 129.6 (Ar-C), 124.7 (Ar-C), 88.1 (C1), 80.8 (C5), 76.2 (C3), 70.7 (C2), 70.4 (C4), 62.7 (C6); HRMS *m/z* (ES^+) Found $(M+Na)^+$ 340.0470, $C_{12}H_{15}NO_7SNa$ requires M^+ 340.0461. Data matched those reported previously.[19]

p-(Methoxy)-phenyl 1-thio-β-D-galactopyranoside 62

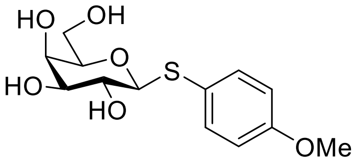

p-(Methoxy)-phenyl 2,3,4,6-tetra-*O*-acetyl-1-thio-β-D-galactopyranoside (800 mg, 1.70 mmol, 1.0 equiv.) was deprotected using Na_2CO_3 (64.0 mg, 0.60 mmol, 0.35 equiv.) in MeOH (7 mL). After 3 hours TLC analysis revealed complete consumption of the starting material (R_f = 0.43 9:1 DCM/MeOH,) Amberlite IR120 (H^+) ion exchange resin was added and the mixture stirred until neutral (confirmed using Johnson® pH paper) after which the mixture was filtered and washed with MeOH (100 mL). The combined organic filtrates were concentrated under reduced pressure. Following column chromatography (DCM/MeOH, 0-20%) the title compound **62** was obtained as a white solid (490 mg, 1.62 mmol, 95%); R_f = 0.43 (9:1 DCM/MeOH); ^{1}H NMR (400 MHz, MeOD) δ 7.53 (m, 2H, Ar*H*), 6.87 (m, 2H, Ar*H*), 4.41 (d, *J* = 9.4 Hz, 1H, H-1), 3.89 (d, *J* = 4.4 Hz, 1H, H-4), 3.78 (s, 3H, OCH_3), 3.72 (m, 2H, H-6a, H-6b), 3.58 – 3.45 (m, 3H, H-2, H-3, H-5); $^{13}C\{^1H\}$ NMR (101 MHz, MeOD) δ 161.1 (Ar-C), 135.8 (Ar-C), 125.4 (Ar-C), 115.4 (Ar-C), 91.1 (C1), 80.5 (C5), 76.3 (C3), 70.9 (C2), 70.4 (C4), 62.6 (C6), 55.8 (OCH_3); HRMS *m/z* (ES^+) Found $(M+NH_4)^+$ 320.1169, $C_{13}H_{22}O_6SN$ requires M^+ 320.1162.[18,20]

3. Benzoylation Experiments

Ethyl 2,3,6-tri-*O*-benzoyl-1-thio-α-D-galactopyranoside 2

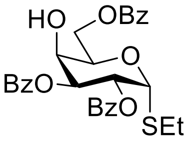

Following the general acylation procedure, ethyl 1-thio-α-D-galactopyranoside **1** (111 mg, 0.495 mmol, 1.0 equiv.) and BzCl (178 µL, 1.53 mmol, 3.1 equiv.) were reacted. Purification by column chromatography (hexane/EtOAc, 0-30%) yielded the title compound **2** (236 mg, 0.440 mmol, 89%) as white foam. R_f = 0.58 (hexane/EtOAc, 7:3); $[\alpha]_D^{24}$ = +81.4 (*c* = 1.0, $CHCl_3$); ^{1}H NMR (400 MHz, $CDCl_3$) δ 8.06 – 7.95 (m, 6H, Ar*H*), 7.60 – 7.55 (m, 1H, Ar*H*), 7.54 – 7.49 (m, 2H, Ar*H*), 7.47 – 7.41 (m, 2H, Ar*H*), 7.41 – 7.35 (m, 4H, Ar*H*), 5.93 (d, *J* = 5.8 Hz, 1H, H-1), 5.88 (dd, *J* = 10.3, 5.8 Hz, 1H, H-2), 5.64 (dd, *J* = 10.3, 3.2 Hz, 1H, H-3), 4.78 (t, *J* = 6.3 Hz, 1H, H-5), 4.70 (dd, *J* = 11.5, 5.4 Hz, 1H, H-6a), 4.57 (dd, *J* = 11.5, 7.0 Hz, 1H, H-6b), 4.42 (d, *J* = 2.4 Hz, 1H, H-4), 2.67 – 2.50 (m, 2H, CH_2), 1.22 (t, *J* = 7.4 Hz, 3H, CH_3); $^{13}C\{^1H\}$ NMR (101 MHz, $CDCl_3$) δ 166.5 (C=O, Bz), 165.7 (C=O, Bz), 165.6 (C=O, Bz), 133.5 (Ar-C), 133.4 (Ar-C), 133.3 (Ar-C), 129.9 (Ar-C), 129.8 (Ar-C), 129.7 (Ar-C), 129.6 (Ar-C), 129.19 (Ar-C), 129.18 (Ar-C), 128.5 (Ar-C), 128.4 (Ar-C), 82.2 (C1), 71.4 (C3), 68.4 (C2), 68.14 (C4), 68.12 (C5), 63.3 (C6), 23.9 (CH_2), 14.6 (CH_3); HRMS *m/z* (ES^+) Found: $(M+H)^+$ 537.1572, $C_{29}H_{29}O_8S$, requires M^+ 537.1578.

Phenyl 2,3,6-tri-*O*-benzoyl-1-thio-α-D-galactopyranoside 4

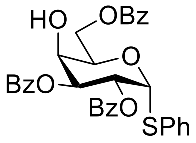

Following the general acylation procedure, phenyl 1-thio-α-D-galactopyranoside **3** (194 mg, 0.712 mmol, 1.0 equiv.) and BzCl (257 µL, 2.21 mmol, 3.1 equiv.) were reacted. Purification by column chromatography (hexane/EtOAc, 0-30%) yielded the title compound **4** (325 mg, 0.556 mmol, 78%) as a white foam. R_f = 0.63 (hexane/EtOAc, 7:3); $[\alpha]_D^{23}$ = +119.6 (*c* = 1.0, $CHCl_3$); ^{1}H NMR (400 MHz, $CDCl_3$) δ 8.04 – 7.99 (m, 4H, Ar*H*), 7.97 – 7.93 (m, 2H, Ar*H*), 7.58 – 7.50 (m, 3H, Ar*H*), 7.46 – 7.35 (m, 8H, Ar*H*), 7.18 – 7.07 (m, 3H, Ar*H*), 6.16 (d, *J* = 5.7 Hz, 1H, H-1), 5.97 (dd, *J* = 10.8, 5.7 Hz, 1H, H-2), 5.70 (dd, *J* = 10.8, 3.1 Hz, 1H, H-3), 4.94 (dd, *J* = 6.9, 5.4 Hz, 1H, H-5), 4.67 (dd, *J* = 11.7, 4.9 Hz, 1H, H-6a), 4.56 (dd, *J* = 11.7, 7.5 Hz, 1H, H-6b), 4.47 (d, *J* = 2.1 Hz, 1H, H-4); $^{13}C\{^1H\}$ NMR (101 MHz, $CDCl_3$) δ 166.5 (C=O, Bz), 165.74 (C=O, Bz), 165.7 (C=O, Bz), 133.6 (Ar-C), 133.5 (Ar-C), 133.2 (Ar-C), 132.8 (Ar-C), 132.0 (Ar-C), 130.0 (Ar-C), 129.9 (Ar-C), 129.8 (Ar-C), 129.6 (Ar-C), 129.2 (Ar-C), 129.1 (Ar-C), 129.0 (Ar-C), 128.6 (Ar-C), 128.5 (Ar-C), 128.4 (Ar-C), 127.5 (Ar-C), 86.1 (C1), 71.4 (C3), 69.0 (C5), 68.5 (C2), 68.2 (C4), 63.6 (C6); HRMS *m/z* (ES^+) Found: $(M+Na)^+$ 607.1392, $C_{33}H_{28}O_8NaS$ requires M^+ 607.1397.

Phenyl 2,3,6-tri-*O*-benzoyl-α-D-galactopyranoside 6

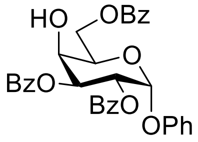

Following the general acylation procedure, phenyl α-D-galactopyranoside **5** (137 mg, 0.534 mmol, 1.0 equiv.) and BzCl (192 µL, 1.66 mmol, 3.1 equiv.) were reacted. Purification by column chromatography (hexane/EtOAc, 0-30%) yielded the title compound **6** (237 mg, 0.417 mmol, 78%) as a colourless oil. R_f = 0.39 (hexane/EtOAc, 7:3); $[\alpha]_D^{24}$ = +117.1 (*c* = 1.0, $CHCl_3$); ^{1}H NMR (400 MHz, $CDCl_3$) δ 8.14 – 8.09 (m, 1H, Ar*H*), 8.06 – 7.96 (m, 4H, Ar*H*), 7.92 – 7.86 (m, 2H, Ar*H*), 7.60 – 7.45 (m, 4H, Ar*H*), 7.38 (dd, *J* = 10.7, 4.7 Hz, 4H, Ar*H*), 7.23 – 7.18 (m, 2H, Ar*H*), 7.15 – 7.09 (m, 2H, Ar*H*), 7.04 – 6.95 (m, 1H, Ar*H*), 6.01 – 5.95 (m, 2H, H-2, H-3), 5.98 (d, *J* = 3.7 Hz, 1H, H-1), 5.90 (dd, *J* = 10.7, 3.5 Hz, 1H, H-2), 4.66 – 4.55 (m, 3H, H-5, H-6a, H-6b), 4.50 (d, *J* = 3.0 Hz, 1H, H-4); $^{13}C\{^1H\}$ NMR (101 MHz, $CDCl_3$) δ 166.5 (C=O, Bz), 166.1 (C=O, Bz), 165.9 (C=O, Bz), 133.5 (Ar-C), 133.38 (Ar-C), 133.35 (Ar-C), 133.2 (Ar-C), 130.1 (Ar-C), 129.9 (Ar-C), 129.8 (Ar-C), 129.6 (Ar-C), 129.5 (Ar-C), 129.24 (Ar-C), 129.22 (Ar-C), 128.5 (Ar-C), 128.44 (Ar-C), 128.42 (Ar-C), 128.35 (Ar-C), 122.8 (Ar-C), 95.5 (C1), 80.0 (C3), 68.9 (C5), 68.4 (C2), 68.1 (C4), 63.5 (C6); HRMS *m/z* (ES^+) Found: $(M+NH_4)^+$ 586.2073, $C_{33}H_{32}O_9N$ requires M^+ 586.2072.

Ethyl 2,3,6-tri-*O*-benzoyl-α-D-galactopyranoside 8

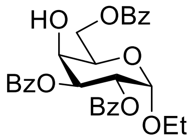

Following the general acylation procedure, ethyl α-D-galactopyranoside **7** (32.0 mg, 0.154 mmol, 1.0 equiv.) and BzCl (55.3 μL, 0.476 mmol, 3.1 equiv.) were reacted. Purification by column chromatography (hexane/EtOAc, 0-30%) yielded the title compound **8** (58.5 mg, 0.112 mmol, 73%) as a colourless oil. R_f = 0.40 (hexane/EtOAc, 7:3); $[\alpha]_D^{24}$ = +111.2 (*c* = 1.0, $CHCl_3$); ^{1}H NMR (400 MHz, $CDCl_3$) δ 8.06 – 7.96 (m, 6H, Ar*H*), 7.60 – 7.55 (m, 1H, Ar*H*), 7.54 – 7.48 (m, 2H, Ar*H*), 7.48 – 7.42 (m, 2H, Ar*H*), 7.41 – 7.35 (m, 4H, Ar*H*), 5.77 (dd, *J* = 10.7, 3.1 Hz, 1H, H-3), 5.68 (dd, *J* = 10.7, 3.7 Hz, 1H, H-2), 5.32 (d, *J* = 3.7 Hz, 1H, H-1), 4.67 (dd, *J* = 11.4, 6.1 Hz, 1H, H-6a), 4.55 (dd, *J* = 11.4, 6.7 Hz, 1H, H-6b), 4.45 – 4.38 (m, 2H, H-4, H-5), 3.81 (dq, *J* = 9.9, 7.1 Hz, 1H, OC*H*H), 3.56 (dq, *J* = 10.0, 7.0 Hz, 1H, OC*H*H), 1.21 (t, *J* = 7.1 Hz, 3H, CH_3); $^{13}C\{^1H\}$ NMR (101 MHz, $CDCl_3$) δ 166.5 (C=O, Bz), 166.1 (C=O, Bz), 165.8 (C=O, Bz), 133.4 (Ar-C), 133.3 (Ar-C), 133.2 (Ar-C), 129.83 (Ar-C), 129.82 (Ar-C), 129.73 (Ar-C), 129.67 (Ar-C), 129.5 (Ar-C), 129.4 (Ar-C), 128.49 (Ar-C), 128.47 (Ar-C), 128.4 (Ar-C), 96.4 (C1), 71.0 (C3), 68.9 (C2), 68.2 (C4), 67.7 (C5), 64.1 (CH_2), 63.3 (C6), 15.1 (CH_3); HRMS *m/z* (ES^+) Found: $(M+NH_4)^+$ 538.2071, $C_{29}H_{32}O_9N$ requires M^+ 538.2072.

Propargyl 2,3,6-tri-*O*-benzoyl-α-D-galactopyranoside 10

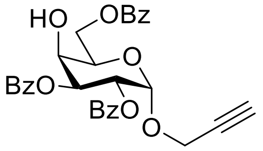

Following the general acylation procedure, propargyl-α-D-galactopyranoside **9** (47.0 mg, 0.215 mmol, 1.0 equiv.) and BzCl (77.6 μL, 0.668 mmol, 3.1 equiv.) were reacted. Purification by column chromatography (hexane/EtOAc, 0-30%) yielded the title compound **10** as a white foam (87.0 mg, 0.164 mmol, 76%). R_f = 0.49 (hexane/EtOAc, 7:3); $[\alpha]_D^{24}$ = +124.2; ^{1}H NMR (400 MHz, MeOD) δ 8.14 – 7.84 (m, 6H, Ar*H*), 7.71 – 7.54 (m, 2H, Ar*H*), 7.56 – 7.47 (m, 4H, Ar*H*), 7.45 – 7.36 (m, 4H, Ar*H*), 5.72 (dd, *J* = 10.8, 3.5 Hz, 1H, H-2), 5.67 (dd, *J* = 10.8, 2.9 Hz, 1H, H-3), 5.50 (d, *J* = 3.4 Hz, 1H, H-1), 4.59 (dd, *J* = 11.2, 7.4 Hz, 1H, H-6a), 4.53 (dd, *J* = 11.2, 5.0 Hz, 1H, H-6b), 4.48 – 4.40 (m, 3H, H-4, H-5, C*H*H), 4.34 (dd, *J* = 15.9, 2.4 Hz, 1H, C*H*H), 2.78 (t, *J* = 2.4 Hz, 1H, CH); $^{13}C\{^1H\}$ NMR (101 MHz, MeOD) δ 166.3 (C=O, Bz), 166.00 (C=O, Bz), 165.96 (C=O, Bz), 133.12 (Ar-C), 133.07 (Ar-C), 133.0 (Ar-C), 129.8 (Ar-C), 129.6 (Ar-C), 129.33 (Ar-C), 129.30 (Ar-C), 129.27 (Ar-C), 129.2 (Ar-C), 128.3 (Ar-C), 128.2 (Ar-C), 128.1 (Ar-C), 95.0 (C1), 78.1 (propargyl-C), 75.1 (propargyl-CH), 70.9 (C3), 68.9 (C5), 68.7 (C2), 67.4 (C4), 63.6 (CH_2), 54.3 (C6); HRMS *m/z* (ES^+) Found: $(M+H)^+$ 531.1639, $C_{30}H_{27}O_9$ requires M^+ 531.1655.

Methyl 2,3-di-*O*-benzoyl-6-*O*-triisopropylsilyl-α-D-galactopyranoside 12

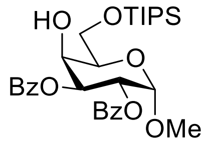

Following the general acylation procedure, methyl 6-*O*-triisopropylsilyl-α-D-galactopyranoside **11** (100 mg, 0.285 mmol, 1.0 equiv.) and BzCl (69.6 µL, 0.599 mmol, 2.1 equiv.) were reacted. Purification by column chromatography (hexane/EtOAc, 0-30%) yielded the title compound **12** as a colourless oil (117 mg, 0.209 mmol, 74%). R_f = 0.44 (hexane/EtOAc, 8:2); $[\alpha]_D^{24}$ = +124.8 (*c* = 1.0, CHCl3); ^{1}H NMR (400 MHz, MeOD) δ 8.01 – 7.97 (m, 2H, Ar*H*), 7.96 – 7.91 (m, 2H, Ar*H*), 7.58 – 7.48 (m, 2H, Ar*H*), 7.44 – 7.33 (m, 4H, Ar*H*), 5.65 (dd, *J* = 10.7, 3.5 Hz, 1H, H-2), 5.59 (dd, *J* = 10.7, 2.9 Hz, 1H, H-3), 5.13 (d, *J* = 3.5 Hz, 1H, H-1), 4.35 (d, *J* = 2.5 Hz, 1H, H-4), 4.06 – 3.98 (m, 2H, H-5, H-6a), 3.94 (dd, *J* = 9.5, 5.9 Hz, 1H, H-6b), 3.45 (s, 3H, OCH_3), 1.14 – 1.10 (m, 21H, ^{i}PrSi); ^{13}C{^{1}H} NMR (101 MHz, MeOD) δ 166.1 (C=O, Bz), 166.0 (C=O, Bz), 133.1 (Ar-C), 133.0 (Ar-C), 129.7 (Ar-C), 129.3 (Ar-C), 129.2 (Ar-C), 128.1 (Ar-C), 128.1 (Ar-C), 97.5 (C1), 71.5 (C3), 71.0 (C5), 69.3 (C2), 67.4 (C4), 62.5 (C6), 54.3 (CH_3), 17.06 (CH_3, ^{i}Pr), 17.05 (CH_3, ^{i}Pr), 11.8 (CH, ^{i}Pr); HRMS *m/z* (ES^+) Found: $(M+Na)^+$ 581.2523, $C_{30}H_{42}O_8NaSi$ requires M^+ 581.2541.

p-(Methoxy)-phenyl 2,3,6-tri-*O*-benzoyl-α-D-galactopyranoside 14

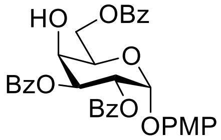

Following the general acylation procedure, *p*-(methoxy)-phenyl α-D-galactopyranoside **13** (286 mg, 1.00 mmol, 1.0 equiv.) and BzCl (360 µL, 3.10 mmol, 3.1 equiv.) were reacted. Purification by column chromatography (hexane/EtOAc, 0-30%) yielded the title compound **14** (521 mg, 0.870 mmol, 87%) as a colourless oil. R_f = 0.41 (hexane/EtOAc, 7:3); $[\alpha]_D^{24}$ = +139.0 (*c* = 2.0, CHCl3); ^{1}H NMR (400 MHz, CDCl3) δ 8.06 – 7.99 (m, 4H, Ar*H*), 7.98 – 7.90 (m, 2H, Ar*H*), 7.61 – 7.47 (m, 3H, Ar*H*), 7.47 – 7.34 (m, 6H, Ar*H*), 7.07 – 7.01 (m, 2H, Ar*H*), 6.75 – 6.68 (m, 2H, Ar*H*), 5.98 – 5.94 (m, 1H, H-3), 5.87 – 5.81 (m, 2H, H-1, H-2), 4.65 (dd, *J* = 14.3, 8.3 Hz, 1H, H-6a), 4.61 – 4.55 (m, 2H, H-5, H-6b), 4.48 (t, *J* = 2.9 Hz, 1H, H-4), 3.71 (s, 3H, OCH_3), 2.63 (d, *J* = 3.9 Hz, 1H, 4-OH); ^{13}C{^{1}H} NMR (101 MHz, CDCl3) δ 166.4 (C=O, Bz), 166.0 (C=O, Bz), 165.8 (C=O, Bz), 155.4 (Ar-C), 150.5 (Ar-C), 133.6 (Ar-C), 133.4 (Ar-C), 133.2 (Ar-C), 129.9 (Ar-C), 129.8 (Ar-C), 129.6 (Ar-C), 129.24 (Ar-C), 129.21 (Ar-C), 128.6 (Ar-C), 128.5 (Ar-C), 128.4 (Ar-C), 118.5 (Ar-C), 114.6 (Ar-C), 96.3 (C1), 70.9 (C3), 68.6 (C5), 68.5 (C2), 68.2 (C4), 63.5 (C6), 55.6 (OCH_3); HRMS *m/z* (ES^+) Found: $(M+NH_4)^+$ 616.2177, $C_{34}H_{34}O_{10}N$ requires M^+ 616.2177.

Allyl 3,6-di-*O*-benzoyl-2-acetamido-2-deoxy-β-D-galactopyranoside 16

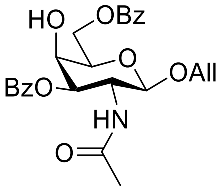

Following the general acylation procedure, allyl 2-acetamido-2-deoxy-β-D-galactopyranoside **15** (1.07 g, 4.10 mmol, 1.0 equiv.) and BzCl (1.00 mL, 8.60 mmol, 2.1 equiv.) were reacted. Purification by column chromatography (DCM/EtOAc, 0-50%) yielded the title compound **16** (1.32 g, 2.81 mmol, 69%) as a white solid. $R_f = 0.43$ (DCM/EtOAc, 8:2); ^{1}H NMR (400 MHz, MeOD) δ 8.16 – 7.89 (m, 4H, Ar*H*), 7.71 – 7.58 (m, 2H, Ar*H*), 7.58 – 7.41 (m, 4H, Ar*H*), 5.98 – 5.86 (m, 1H, =CH), 5.26 (ddd, *J* = 17.3, 3.5, 1.7 Hz, 1H, C*H*H=), 5.19 – 5.11 (m, 2H, H-3, C*H*H=), 4.69 (d, *J* = 8.5 Hz, 1H, H-1), 4.63 (dd, *J* = 11.3, 7.4 Hz, 1H, H-6a), 4.57 – 4.46 (m, 2H, H-2, H-6b), 4.34 (ddt, *J* = 13.2, 5.0, 1.6 Hz, 1H, OC*H*H), 4.28 (d, *J* = 2.8 Hz, 1H, H-4), 4.15 (ddt, *J* = 13.2, 6.0, 1.4 Hz, 1H, OC*H*H), 4.09 – 4.04 (m, 1H, H-5), 1.87 (s, 3H, Ac); ^{13}C{^{1}H} NMR (101 MHz, MeOD) δ 172.1 (C=O, Ac), 166.4 (C=O, Bz), 166.2 (C=O, Bz), 134.1 (=CH-), 133.1 (Ar-C), 133.0 (Ar-C), 129.8 (Ar-C), 129.7 (Ar-C), 129.5 (Ar-C), 129.2 (Ar-C), 128.3 (Ar-C), 128.2 (Ar-C), 116.0 (CH_2=), 100.5 (C1), 74.4 (C3), 72.5 (C5), 69.5 (CH_2) 65.9 (C4), 63.5 (C6), 50.1 (C2), 21.4 (Ac-CH_3); HRMS *m/z* (ES^+) Found: $(M+H)^+$ 470.1809, $C_{25}H_{28}O_8N$ requires M^+ 470.1802. Data matched those reported previously.[21]

Allyl 3,6-di-*O*-benzoyl-2-acetamido-2-deoxy-α-D-galactopyranoside 18

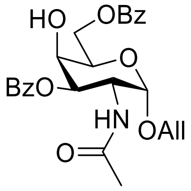

Following the general acylation procedure, allyl 2-acetamido-2-deoxy-α-D-galactopyranoside **17** (782 mg, 3.00 mmol, 1.0 equiv.) and BzCl (732 µL, 6.30 mmol, 2.1 equiv.) were reacted. Purification by column chromatography (hexane/EtOAc, 0-100%) yielded the title compound **18** as a white solid (1.07 g, 2.28 mmol, 76%). $R_f = 0.58$ (EtOAc); ^{1}H NMR (400 MHz, $CDCl_3$) δ 8.13 – 7.96 (m, 4H, Ar*H*), 7.60 – 7.53 (m, 2H, Ar*H*), 7.48 – 7.38 (m, 4H, Ar*H*), 5.96 – 5.85 (m, 1H, =CH-), 5.79 (d, *J* = 9.8 Hz, 1H, NH), 5.37 (dd, *J* = 11.1, 3.0 Hz, 1H, H-3), 5.27 (dq, *J* = 17.2, 1.5 Hz, 1H, C*H*H=), 5.20 (dd, *J* = 10.3, 1.3 Hz, 1H, C*H*H=), 4.99 (d, *J* = 3.7 Hz, 1H, H-1), 4.92 (ddd, *J* = 11.1, 9.9, 3.7 Hz, 1H, H-2), 4.62 (dd, *J* = 11.5, 5.6 Hz, 1H, H6-a), 4.55 (dd, *J* = 11.5, 6.9 Hz, 1H, H-6b), 4.30 – 4.26 (m, 2H, H-4, H-5), 4.26 – 4.20 (m, 1H, OC*H*H), 4.07 – 4.01 (m, 1H, OC*H*H), 1.88 (s, 3H, Ac); ^{13}C{^{1}H} NMR (101 MHz, $CDCl_3$) δ 170.1 (C=O, Ac), 166.6 (C=O, Bz), 166.4 (C=O, Bz), 133.5 (Ar-C), 133.4 (=CH-), 133.2 (Ar-C), 130.0 (Ar-C), 129.74 (Ar-C), 129.67 (Ar-C), 129.3 (Ar-C), 128.6 (Ar-C), 128.4 (Ar-C), 118.2 (CH_2=), 97.1 (C1), 72.0 (C3), 68.6 (CH_2), 68.4 (C5), 67.5 (C4), 63.6 (C6), 47.3 (C2), 23.3 (Ac-CH_3); HRMS *m/z* (ES^+) Found: $(M+H)^+$ 470.1809, $C_{25}H_{28}O_8N$ requires M^+ 470.1802. Data matched those reported previously.[22]

3-Azidopropyl-3,6-di-*O*-benzoyl-2-deoxy-2-acetamido-β-D-galactopyranoside 20

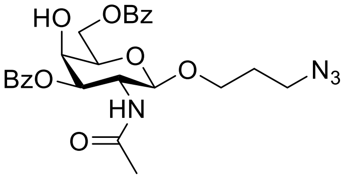

Following the general acylation procedure, 3-azidopropyl-2-deoxy-2-acetamido-β-D-galactopyranoside **19** (37.0 mg, 122 µmol, 1.0 equiv.) and BzCl (29.7 µL, 255 µmol, 2.1 equiv.) were reacted. Purification by column chromatography (DCM/EtOAc, 0-50%) yielded the title compound **20** as a white solid (52.0 mg, 102 µmol, 83%). R_f = 0.30 (1:1 hexane/EtOAc); ^{1}H NMR (400 MHz, $CDCl_3$) δ 8.08 – 7.97 (m, 4H, Ar*H*), 7.61 – 7.46 (m, 2H, Ar*H*), 7.43 – 7.34 (m, 4H, Ar*H*), 6.04 (d, *J* = 8.9 Hz, 1H, NH), 5.37 (dd, *J* = 11.2 Hz, 3.1 Hz, 1H, H-3), 4.71 (d, *J* = 8.3 Hz, 1H, H-1), 4.67 – 4.54 (m, 2H, H-6a, H-6b), 4.44 (dt, *J* = 11.1, 8.8 Hz, 1H, H-2), 4.25 (ad, *J* = 2.8 Hz, 1H, H-4), 4.09 – 4.00 (m, 1H, H-5), 3.97 (dt, *J* = 10.7, 5.5 Hz, 1H, OC*H*H), 3.63 (ddd, *J* = 9.8 Hz, 8.3 Hz, 4.8 Hz, 1H, OC*H*H), 3.45 – 3.32 (m, 2H, CH_2), 1.89 (s, 3H, OAc), 1.80 – 0.94 (m, 2H, CH_2); ^{13}C{^{1}H} NMR (101 MHz, $CDCl_3$) δ 170.6 (C=O, Ac), 166.5 (C=O, Bz), 166.4 (C=O, Bz), 133.6 (Ar-C), 133.3 (Ar-C), 129.9 (Ar-C), 129.7 (Ar-C), 129.7 (Ar-C), 129.1 (Ar-C), 128.54 (Ar-C), 128.45 (Ar-C), 101.4 (C1), 73.6 (C3), 72.3 (C5), 66.9 (C4), 66.1 (CH_2), 63.0 (C6), 50.8 (C2), 48.1 (CH_2), 29.0 (CH_2), 23.3 (Ac-CH_3); HRMS *m/z* (ES^+) Found: $(M+H)^+$ 513.1977, $C_{25}H_{29}O_8N_4$ requires M^+ 513.1985. Data matched those reported previously.[1]

Methyl 2,3,6-tri-*O*-acetyl-α-D-galactopyranoside 22

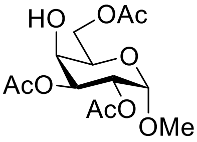

Following the general acylation procedure, methyl α-D-galactopyranoside (100 mg, 0.515 mmol, 1.0 equiv.) and AcCl (113 µL, 1.60 mmol, 3.1 equiv.) were reacted. Purification by column chromatography (hexane/EtOAc, 0-30%) yielded the title compound **22** (120 mg, 0.376 mmol, 73%) as a colourless oil. R_f = 0.49 (hexane/EtOAc, 6:4); ^{1}H NMR (400 MHz, $CDCl_3$) δ 5.22 (dd, *J* = 10.7, 2.9 Hz, 1H, H-4), 5.17 (dd, *J* = 10.7, 3.3 Hz, 1H, H-3), 4.91 (d, *J* = 3.3 Hz, 1H, H-1), 4.28 (dd, *J* = 11.5, 5.9 Hz, 1H, H-6a), 4.18 (dd, *J* = 11.5, 6.7 Hz, 1H, H-6b), 4.05 (as, 1H, H-4), 3.97 (t, *J* = 6.3 Hz, 1H, H-5), 3.34 (s, 3H, OCH_3), 2.35 (d, *J* = 4.1 Hz, 1H, 4-OH), 2.04 (s, 3H, OAc), 2.02 (s, 3H, OAc), 2.02 (s, 3H, OAc); ^{13}C {1H} NMR (101 MHz, $CDCl_3$) δ 170.9 (C=O, Ac), 170.4 (C=O, Ac), 170 (C=O, Ac), 97.2 (C1), 70.0 (C2), 68.1 (C3), 67.9 (C4), 67.3 (C5), 62.8 (C6), 55.4 (OCH_3), 20.9 (Ac-CH_3), 20.8 (2Ac-CH_3); HRMS *m/z* (ES^+) Found: $(M+Na)^+$ 343.0992, $C_{13}H_{20}O_9Na$ requires M^+ 343.1000. Data matched those reported previously.[23]

Methyl 2,6-di-*O*-*tert*-butylcarbamoyl-3,4-*O*-carbonyl-α-D-galactopyranoside 23

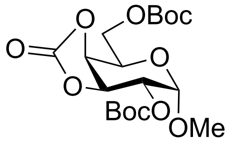

To a solution of methyl α-D-galactopyranoside (100 mg, 0.52 mmol, 1.0 equiv.) in pyridine (2 mL) was added $(Boc)_2O$ (351 mg, 1.61 mmol, 3.1 equiv.) and Et_3N (0.19 mL, 2.58 mmol, 5.0 equiv.). The reaction mixture was first stirred at -40 °C and then at RT for 18 h. The reaction was diluted with DCM (20 mL), washed with sat.aq. $NaHCO_3$ (20 mL), dried ($MgSO_4$) and filtered. The combined organic filtrates were concentrated under reduced pressure and following column chromatography (hexane/EtOAc, 0-20%) the title compound **23** was obtained as an orange oil (89.4 mg, 0.22 mmol, 44%); R_f = 0.85 (6:4 hexane/EtOAc); $[\alpha]_D^{21}$ = +15.7 (*c* = 1.0, $CHCl_3$); ^1H NMR (400 MHz, $CDCl_3$) δ 5.01 (d, *J* = 3.5 Hz, 1H, H-1), 4.92 – 4.80 (m, 3H, H-3, H-2, H-4), 4.37 – 4.27 (m, 2H, H-6a, H-6b), 4.25 (ddd, *J* = 7.4, 5.4, 2.2 Hz, 1H, H-5), 3.43 (s, 3H, OCH_3), 1.50 [s, 9H, $(CH_3)_3$], 1.49 [s, 9H, $(CH_3)_3$]; $^{13}C\{^1H\}$ NMR (101 MHz, $CDCl_3$) δ 153.2 (C=O), 152.9 (C=O), 152.1 (C=O), 96.01 (C1), 83.9 ($C(CH_3)_3$), 83.0 ($C(CH_3)_3$), 74.5 (C4), 74.1 (C3), 71.2 (C2), 64.5 (C5), 64.3 (C6), 56.1 (OCH_3), 27.7 [$C(CH_3)_3$], 27.6 [$C(CH_3)_3$]; HRMS *m/z* (ES^+) Found: $(M+NH_4)^+$ 438.1974, $C_{18}H_{32}O_{11}N$ requires 438.1970.

Methyl 2,3-di-*O*-benzoyl-α-L-fucopyranoside 25

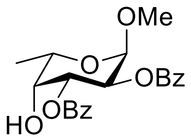

Following the general acylation procedure, methyl α-L-fucopyranoside (300 mg, 1.68 mmol, 1.0 equiv.) and BzCl (0.41 mL, 3.53 mmol, 2.1 equiv.) were reacted. Purification by column chromatography (hexane/EtOAc 2:1) yielded the title compound **25** as a white solid (537 mg, 1.39 mmol, 83%), R_f = 0.50 (hexane:EtOAc 1:1); ^1H NMR (400 MHz, $CDCl_3$) δ 7.98 (ddd, *J* = 8.5, 3.5, 1.4 Hz, 4H, Ar*H*), 7.54 – 7.46 (m, 2H, Ar*H*), 7.36 (dd, *J* = 8.2, 6.9 Hz, 4H, Ar*H*), 5.70 (dd, *J* = 10.7, 3.0 Hz, 1H, H-3), 5.62 (dd, *J* = 10.7, 3.7 Hz, 1H, H-2), 5.12 (d, *J* = 3.6 Hz, 1H, H-1), 4.20 (qd, *J* = 6.2, 1.0 Hz, 1H, H-5), 4.14 (dd, *J* = 3.1, 1.2 Hz, 1H, H-4), 3.42 (s, 3H, OCH_3), 1.36 (d, *J* = 6.6 Hz, 3H, CH_3); $^{13}C\{^1H\}$ (101 MHz, $CDCl_3$) δ 166.3 (C=O, Bz), 166.0 (C=O, Bz), 133.4 (Ar-C), 133.3 (Ar-C), 130.0 (Ar-C), 129.9 (Ar-C), 129.6 (Ar-C), 128.6 (Ar-C), 128.5 (Ar-C), 97.6 (C1), 71.7 (C3), 71.0 (C4), 69.0 (C2), 65.6 (C5), 55.6 (OCH_3), 16.2 (CH_3); HRMS *m/z* (ES^+) Found: $(M+Na)^+$ 409.1263, $C_{21}H_{22}O_7Na$ requires M^+ 409.1258. Data matched those previously reported.[24]

Benzoylation of phenyl 1-thio-β-D-galactopyranoside 26

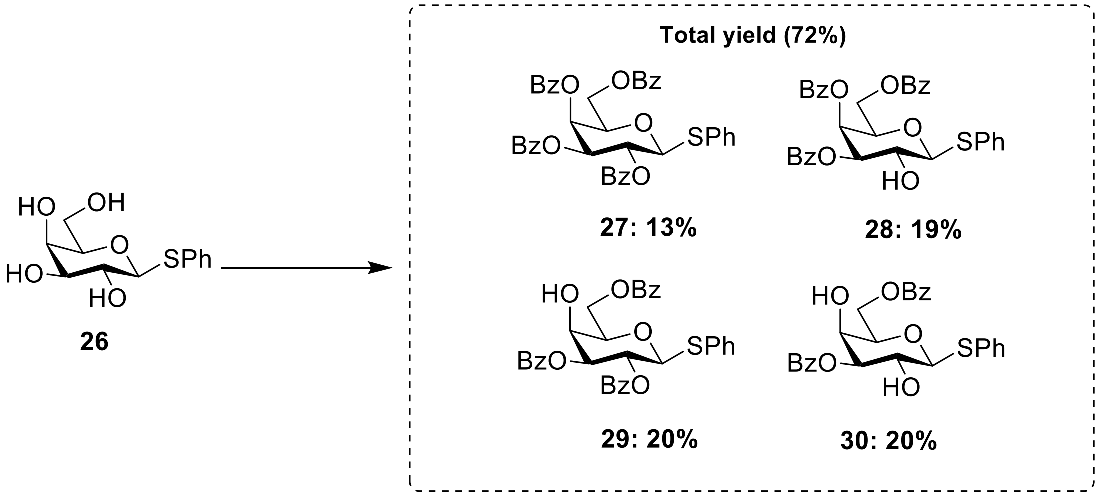

Following the general acylation procedure, phenyl 1-thio-β-D-galactopyranoside **26** (74.0 mg, 0.272 mmol, 1.0 equiv.) and BzCl (98.0 µL, 0.843 mmol, 3.1 equiv.) were reacted. Purification by column chromatography (hexane/EtOAc, 0-30%) yielded the below compounds in a total yield of 72%.

Phenyl 2,3,4,6-tetra-*O*-benzoyl-1-thio-β-D-galactopyranoside 27

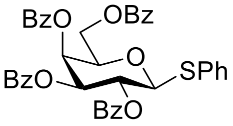

27 (25.3 mg, 36.7 µmol, 13%) as a colourless oil. R_f = 0.48 (hexane/EtOAc, 7:3); ^{1}H NMR (400 MHz, $CDCl_3$) δ 8.05 – 7.96 (m, 4H, Ar*H*), 7.92 – 7.88 (m, 2H, Ar*H*), 7.79 – 7.72 (m, 2H, Ar*H*), 7.65 – 7.52 (m, 5H, Ar*H*), 7.49 – 7.33 (m, 8H, Ar*H*), 7.30 – 7.20 (m, 4H, Ar*H*), 6.00 (d, *J* = 2.6 Hz, 1H, H-4), 5.76 (t, *J* = 9.9 Hz, 1H, H-2), 5.60 (dd, *J* = 9.9, 3.3 Hz, 1H, H-3), 5.04 (d, *J* = 9.9 Hz, 1H, H-1), 4.65 (dd, *J* = 11.1, 6.7 Hz, 1H, H-6a), 4.49 – 4.38 (m, 2H, H-5, H-6b); $^{13}C\{^1H\}$ NMR (101 MHz, $CDCl_3$) δ 166.1 (C=O, Bz), 165.5 (C=O, Bz), 165.4 (C=O, Bz), 165.2 (C=O, Bz), 134.0 (Ar-C), 133.6 (Ar-C), 133.4 (Ar-C), 133.30 (Ar-C), 133.28 (Ar-C), 131.2 (Ar-C), 130.0 (Ar-C), 129.83 (Ar-C), 129.78 (Ar-C), 129.4 (Ar-C), 129.3 (Ar-C), 128.90 (Ar-C), 128.86 (Ar-C), 128.7 (Ar-C), 128.6 (Ar-C), 128.5 (Ar-C), 128.4 (Ar-C), 128.3 (Ar-C), 85.6 (C1), 75.1 (C5), 73.0 (C3), 68.3 (C4), 67.9 (C2), 62.5 (C6); HRMS *m/z* (ES^+) Found: $(M+NH_4)^+$ 706.2110, $C_{40}H_{36}O_{1}NS$ requires M^+ 706.2105. Data matched those reported previously.[25]

Phenyl 3,4,6-tri-*O*-benzoyl-1-thio-β-D-galactopyranoside 28 & phenyl 2,3,6-tri-*O*-benzoyl-1-thio-β-D-galactopyranoside 29

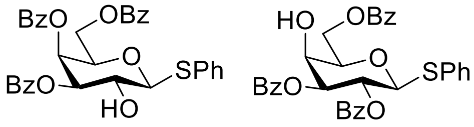

28: 19% **29: 20%**

28/29 were isolated as an inseparable mixture appearing as a white foam (61.2 mg, 0.105 mmol, **28/29**, 1:1.07); ratio determined from ^{1}H NMR integration values. R_f = 0.35 (hexane/EtOAc, 7:3); The following were observed for both regioisomers **28** and **29**: ^{1}H NMR (400 MHz, $CDCl_3$) δ 8.13 – 7.95 (m, 9H, Ar*H*), 7.89 – 7.86 (m, 2H, Ar*H*), 7.83 – 7.79 (m, 2H, Ar*H*), 7.69 – 7.65 (m, 2H, Ar*H*), 7.62 – 7.52 (m, 4H, Ar*H*), 7.50 – 7.27 (m, 20H, Ar*H*), 7.20 – 7.14 (m, 2H, Ar*H*); $^{13}C\{^{1}H\}$ NMR (101 MHz, $CDCl_3$) δ 133.9 (Ar-C), 133.6 (Ar-C), 133. 55 (Ar-C), 133.54 (Ar-C), 133.4 (Ar-C), 133.31 (Ar-C), 133.29 (Ar-C), 132.9 (Ar-C), 132.4 (Ar-C), 130.7 (Ar-C), 130.1 (Ar-C), 129.94 (Ar-C), 129.90 (Ar-C), 129.8 (2 × Ar-C), 129.7 (Ar-C), 129.6 (Ar-C), 129.5 (Ar-C), 129.4 (Ar-C), 129.2 (Ar-C), 129.14 (Ar-C), 129.07 (Ar-C), 128.94 (Ar-C), 128.91 (Ar-C), 128.6 (Ar-C), 128.50 (Ar-C), 128.46 (Ar-C), 128.44 (Ar-C), 128.42 (Ar-C), 128.3 (Ar-C), 128.0 (Ar-C); HRMS *m/z* (ES^+) Found: $(M+Na)^+$ 607.1390, $C_{33}H_{28}O_8NaS$ requires M^+ 607.1397; **28:** ^{1}H NMR (400 MHz, $CDCl_3$) 5.91 (dd, *J* = 3.3, 0.8 Hz, 1H, H-4), 5.42 (dd, *J* = 9.7, 3.3 Hz, 1H, H-3), 4.77 (d, *J* = 9.6 Hz, 1H, H-1), 4.62 (dd, *J* = 11.3, 6.8 Hz, 1H, H-6a), 4.39 (dd, *J* = 11.3, 5.9 Hz, 1H, H-6b), 4.31 (t, *J* = 6.4 Hz, 1H, H-5), 4.06 (t, *J* = 9.6 Hz, 1H, H-2); $^{13}C\{^{1}H\}$ NMR (101 MHz, $CDCl_3$) δ 166.0 (C=O, Bz), 165.9 (C=O, Bz), 165.3 (C=O, Bz), 88.2 (C1), 75.1 (C5), 74.6 (C3), 68.6 (C4), 67.5 (C2), 62.5 (C6); **29:** ^{1}H NMR (400 MHz, $CDCl_3$) δ 5.82 (t, *J* = 10.0 Hz, 1H, H-2), 5.39 (dd, *J* = 9.9, 3.1 Hz, 1H, H-3), 4.99 (d, *J* = 10.1 Hz, 1H, H-1), 4.72 – 4.56 (m, 2H, H-6a, H-6b), 4.44 – 4.40 (m, 1H, H-4), 4.16 – 4.12 (m, 1H, H-5); $^{13}C\{^{1}H\}$ NMR (101 MHz, $CDCl_3$) δ 166.5 (C=O, Bz), 165.8 (C=O, Bz), 165.4 (C=O, Bz), 86.9 (C1), 76.3 (C5), 75. (C3), 67.9 (C2), 67.6 (C4), 63.4 (C6).

Phenyl 3,6-di-*O*-benzoyl-1-thio-β-D-galactopyranoside 30

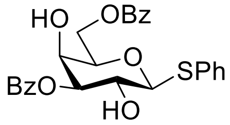

30 (26.5 mg, 55.1 μmol, 20%) as a white foam. R_f = 0.25 (hexane/EtOAc, 7:3); $[\alpha]_D^{23}$ = +12.4 (*c* = 1.0, $CHCl_3$); ^{1}H NMR (400 MHz, $CDCl_3$) δ 8.14 – 7.98 (m, 4H, Ar*H*), 7.64 – 7.57 (m, 4H, Ar*H*), 7.51 – 7.39 (m, 4H, Ar*H*), 7.30 – 7.18 (m, 3H, Ar*H*), 5.18 (dd, *J* = 9.6, 3.2 Hz, 1H, H-3), 4.71 (d, *J* = 9.7 Hz, 1H, H-1), 4.66 (dd, *J* = 11.6, 5.8 Hz, 1H, H-6a), 4.57 (dd, *J* = 11.6, 6.9 Hz, 1H, H-6b), 4.27 (s, *J* = 14.8 Hz, 1H, H-4), 4.14 – 4.00 (m, 2H, H-2, H-5); $^{13}C\{^{1}H\}$ NMR (101 MHz, $CDCl_3$) δ 166.4 (C=O, Bz), 166.1 (C=O, Bz), 133.6 (Ar-C), 133.4 (Ar-C), 132.6 (Ar-C), 132.1 (Ar-C), 129.9 (Ar-C), 129.8 (Ar-C), 129.6 (Ar-C), 129.4 (Ar-C), 129.1 (Ar-C), 128.53 (Ar-C), 128.49 (Ar-C), 128.2 (Ar-C), 89.4 (C1), 76.6 (C3), 76.2 (C5), 67.6 (C4), 67.5 (C2), 63.1 (C6); HRMS *m/z* (ES^-) found: $(M-H^-)$ 479.1175, $C_{26}H_{23}O_7S$ requires M^- 479.1170.

Benzoylation of ethyl 1-thio-β-D-galactopyranoside 31

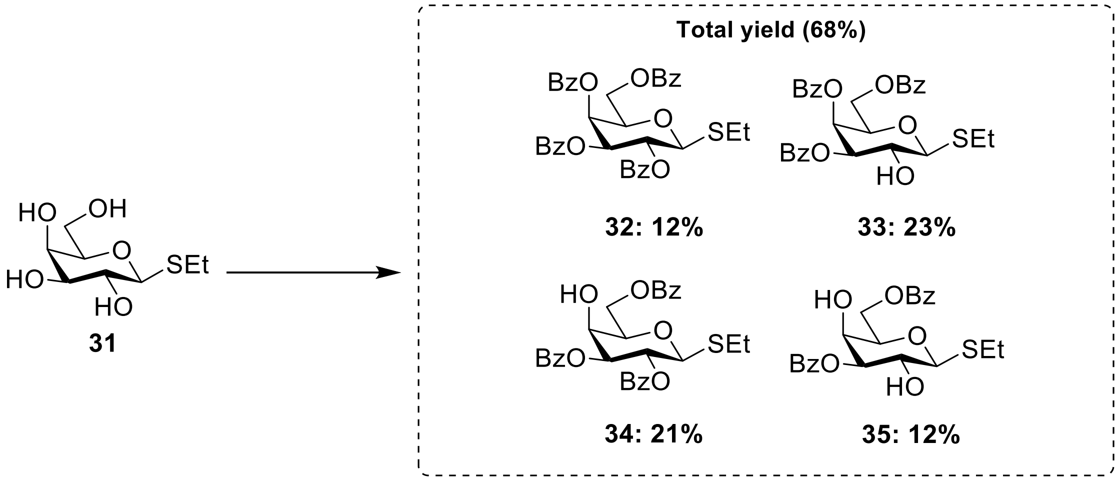

Following the general acylation procedure, ethyl 1-thio-β-D-galactopyranoside **31** (117 mg, 0.522 mmol, 1.0 equiv.) and BzCl (188 µL, 1.62 mmol, 3.1 equiv.) were reacted. Purification by column chromatography (hexane/EtOAc, 0-30%) yielded the below compounds in a total yield of 68%.

Ethyl 2,3,4,6-tetra-*O*-benzoyl-1-thio-β-D-galactopyranoside 32

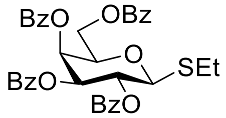

32 (39.1 mg, 61.0 µmmol, 12%) as a white foam. R_f = 0.40 (hexane/EtOAc, 7:3); ^{1}H NMR (400 MHz, $CDCl_3$) δ 8.09 – 8.07 (m, 2H, Ar*H*), 8.05 – 7.99 (m, 2H, Ar*H*), 7.98 – 7.94 (m, 2H, Ar*H*), 7.79 – 7.76 (m, 2H, Ar*H*), 7.65 – 7.60 (m, 1H Ar*H*), 7.58 – 7.46 (m, 4H, Ar*H*), 7.46 – 7.35 (m, 5H, Ar*H*), 7.25 – 7.21 (m, 2H, Ar*H*), 6.04 (dd, *J* = 3.3, 0.6 Hz, 1H, H-4), 5.84 (t, *J* = 10.0 Hz, 1H, H-2), 5.66 (dd, *J* = 10.0, 3.4 Hz, 1H, H-3), 4.88 (d, *J* = 10.0 Hz, 1H, H-1), 4.67 (dd, *J* = 10.9, 6.2 Hz, 1H, H-6a), 4.44 – 4.35 (m, 2H, H-5, H-6b), 2.92 – 2.76 (m, 2H, CH_2), 1.32 (t, *J* = 7.4 Hz, 3H, CH_3); $^{13}C\{^1H\}$ NMR (101 MHz, $CDCl_3$) δ 166.1 (C=O, Bz), 165.54 (C=O, Bz), 165.53 (C=O, Bz), 165.4 (C=O, Bz), 133.6 (Ar-C), 133.34 (Ar-C), 133.31 (Ar-C), 130.0 (Ar-C), 129.84 (Ar-C), 129.79 (Ar-C), 129.78 (Ar-C), 129.4 (Ar-C), 129.2 (Ar-C), 129.1 (Ar-C), 128.8 (Ar-C), 128.7 (Ar-C), 128.5 (Ar-C), 128.4 (Ar-C), 128.3 (Ar-C), 84.3 (C1), 75.1 (C5), 72.8 (C3), 68.4 (C4), 68.3 (C2), 62.3 (C6), 24.6 (CH_2), 15.0 (CH_3); HRMS *m/z* (ES^+) Found: $(M+Na)^+$ 663.1659, $C_{36}H_{32}O_9NaS$ requires M^+ 663.1659. Data matched those reported previously.[26]

Ethyl 2,3,6-tri-*O*-benzoyl-1-thio-β-D-galactopyranoside 34

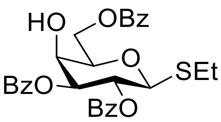

34 (59.7 mg, 0.111 mmol, 21%) as a white foam. R_f = 0.34 (hexane/EtOAc, 7:3); $[\alpha]_D^{23}$ = +26.9 (*c* = 1.0, $CHCl_3$); ^{1}H NMR (400 MHz, $CDCl_3$) δ 8.06 – 8.03 (m, 2H, Ar*H*), 7.99 – 7.95 (m, 4H, Ar*H*), 7.61 – 7.55 (m, 1H, Ar*H*), 7.53 – 7.42 (m, 4H, Ar*H*), 7.40 – 7.34 (m, 4H, Ar*H*), 5.83 (t, *J* = 10.0 Hz, 1H, H-2), 5.40 (dd, *J* = 9.9, 3.1 Hz, 1H, H-3), 4.76 (d, *J* = 10.0 Hz, 1H, H-1), 4.69 (dd, *J* = 11.5, 6.4 Hz, 1H, H-6a), 4.58 (dd, *J* = 11.5, 6.4 Hz, 1H, H-6b), 4.39 (s, 1H, H-4), 4.10 (dd, *J* = 6.8, 6.1 Hz, 1H, H-5), 2.89 – 2.70 (m, 2H, CH_2), 1.27 (t, *J* = 7.4 Hz, 3H, CH_3); $^{13}C\{^1H\}$ NMR (101 MHz, $CDCl_3$) δ 166.5 (C=O, Bz), 165.8 (C=O, Bz), 165.5 (C=O, Bz), 133.5 (Ar-C), 133.4 (Ar-C), 133.2 (Ar-C), 129.9 (Ar-C), 129.82 (Ar-C), 129.79 (Ar-C), 129.5 (Ar-C), 129.4 (Ar-C), 129.0 (Ar-C), 128.51 (Ar-C), 128.49 (Ar-C), 128.4 (Ar-C), 84.0 (C1), 76.1 (C5), 75.1 (C3), 67.9 (C2), 67.5 (C4), 62.9 (C6), 24.2 (CH_2), 15.0 (CH_3); HRMS *m/z* (ES^+) Found: $(M+NH_4)^+$ 554.1838, $C_{29}H_{32}O_8NS$ requires M^+ 554.1843. Data matched those reported previously.[27]

Ethyl 3,4,6-tri-*O*-benzoyl-1-thio-β-D-galactopyranoside 33

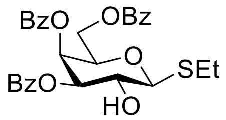

33 (65.5 mg, 0.122 mmol, 23%) as a white foam. R_f = 0.28 (hexane/EtOAc, 7:3); $[\alpha]_D^{23}$ = +6.7 (*c* = 0.5, $CHCl_3$); ^{1}H NMR (400 MHz, $CDCl_3$) δ 8.08 – 8.04 (m, 2H, Ar*H*), 8.04 – 8.00 (m, 2H, Ar*H*), 7.90 – 7.84 (m, 2H, Ar*H*), 7.65 – 7.60 (m, 1H, Ar*H*), 7.58 – 7.53 (m, 1H, Ar*H*), 7.50 – 7.39 (m, 5H, Ar*H*), 7.33 – 7.28 (m, 2H, Ar*H*), 5.95 (dd, *J* = 3.4, 0.8 Hz, 1H, H-4), 5.41 (dd, *J* = 9.6, 3.4 Hz, 1H, H-3), 4.65 (d, *J* = 9.7 Hz, 1H, H-1), 4.62 (dd, *J* = 11.1, 6.6 Hz, 1H, H-6a), 4.36 (dd, *J* = 11.2, 6.4 Hz, 1H, H-6b), 4.28 (dt, *J* = 6.5, 3.2 Hz, 1H, H-5), 4.14 (t, *J* = 9.7 Hz, 1H, H-2), 2.91 – 2.79 (m, 2H, CH_2), 1.39 (t, *J* = 7.4 Hz, 3H, CH_3); $^{13}C\{^1H\}$ NMR (101 MHz, $CDCl_3$) δ 166.1 (C=O, Bz), 165.9 1 (C=O, Bz), 165.5 1 (C=O, Bz), 133.6 (Ar-C), 133.3 (Ar-C), 129.9 (Ar-C), 129.84 (Ar-C), 129.77 (Ar-C), 129.44 (Ar-C), 129.28 (Ar-C), 129.25 (Ar-C), 128.6 (Ar-C), 128.5 (Ar-C), 128.3 (Ar-C), 87.2 (C1), 75.0 (C5), 74.3 (C3), 68.51 (C4), 68.45 (C2), 62.3 (C6), 25.1 (CH_2), 15.4 (CH_3); HRMS *m/z* (ES^+) Found: $(M+NH_4)^+$ 554.1838, $C_{29}H_{32}O_8NS$ requires M^+ 554.1843.

Ethyl 3,6-di-*O*-benzoyl-1-thio-β-D-galactopyranoside 35

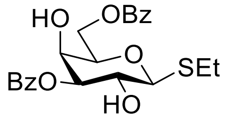

35 (27.9 mg, 64.5 μmol, 12%) as a white foam. R_f = 0.22 (hexane/EtOAc, 7:3); $[\alpha]_D^{23}$ = +0.20 (*c* = 1.0, $CHCl_3$); ^{1}H NMR (400 MHz, $CDCl_3$) δ 8.13 – 8.08 (m, 2H, Ar*H*), 8.05 – 8.01 (m, 2H, Ar*H*), 7.62 – 7.55 (m, 2H, Ar*H*), 7.50 – 7.40 (m, 4H, Ar*H*), 5.17 (dd, *J* = 9.6, 3.2 Hz, 1H, H-3), 4.65 (dd, *J* = 11.5, 6.6 Hz, 1H, H-6a), 4.55 – 4.50 (m, 1H, H-6b), 4.52 (d, *J* = 9.6 Hz, 1H, H-1), 4.27 (s, 1H, H-4), 4.11 – 4.05 (m, 1H, H-2), 4.01 (dd, *J* = 6.9, 6.1 Hz, 1H, H-5), 2.89 – 2.72 (m, 2H, CH_2), 1.34 (t, *J* = 7.4 Hz, 1H, CH_3); $^{13}C\{^1H\}$ NMR (101 MHz, $CDCl_3$) δ 166.5

(C=O, Bz), 166.1 (C=O, Bz), 133.5 (Ar-C), 133.4 (Ar-C), 129.9 (Ar-C), 129.8 (Ar-C), 129.50 (Ar-C), 129.48 (Ar-C), 128.52 (Ar-C), 128.47 (Ar-C), 87.1 (C1), 76.5 (C3), 76.1 (C5), 67.9 (C2), 67.5 (C4), 62.8 (C6), 24.9 (CH_2), 15.4 (CH_3); HRMS *m/z* (ES^-) Found: $(M-H)^-$ 431.1174, $C_{22}H_{23}O_7S$ requires M^- 431.1170.

Benzoylation of Phenyl β-D-galactopyranoside 36

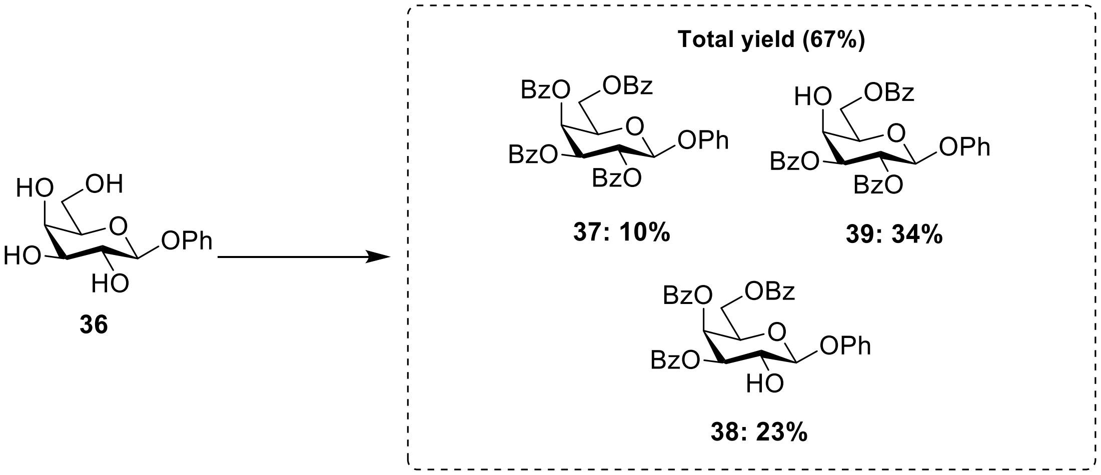

Following the general acylation procedure, phenyl β-D-galactopyranoside **36** (78.0 mg, 0.304 mmol, 1.0 equiv.) and BzCl (110 µL, 0.943 mmol, 3.1 equiv.) were reacted. Purification by column chromatography (hexane/EtOAc, 0-30%) yielded the below compounds in a total yield of 67%.

Phenyl 2,3,4,6-tetra-*O*-benzoyl-β-D-galactopyranoside 37

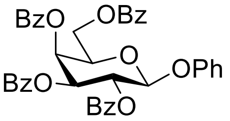

37 (20.6 mg, 30.6 µmol, 10%) as a colourless oil. R_f = 0.48 (hexane/EtOAc, 7:3); ^{1}H NMR (400 MHz, $CDCl_3$) δ 8.19 – 8.10 (m, 4H, Ar*H*), 8.08 – 8.02 (m, 2H, Ar*H*), 7.99 – 7.93 (m, 2H, Ar*H*), 7.85 – 7.76 (m, 2H, Ar*H*), 7.72 – 7.64 (m, 1H, Ar*H*), 7.64 – 7.56 (m, 2H, Ar*H*), 7.56 – 7.43 (m, 6H, Ar*H*), 7.39 – 7.32 (m, 2H, Ar*H*), 7.24 – 7.12 (m, 2H, Ar*H*), 7.09 – 6.96 (m, 2H, Ar*H*), 6.08 (dd, *J* = 10.4, 8.0 Hz, 1H, H-2), 6.05 (dd, *J* = 3.4, 0.7 Hz, 1H, H-4), 5.69 (dd, *J* = 10.4, 3.5 Hz, 1H, H-3), 5.38 (d, *J* = 8.0 Hz, 1H, H-1), 4.67 (dd, *J* = 11.3, 7.5 Hz, 1H, H-6a), 4.55 (dd, *J* = 11.3, 5.4 Hz, 1H, H-6b), 4.52 – 4.47 (m, 1H, H-5); $^{13}C\{^1H\}$ NMR (101 MHz, $CDCl_3$) δ 166.0 (C=O, Bz), 165.57 (C=O, Bz), 165.56 (C=O, Bz), 165.3 (C=O, Bz), 157.1 (Ar-C), 134.5 (Ar-C), 133.7 (Ar-C), 133.4 (Ar-C), 133.3 (Ar-C), 130.6 (Ar-C), 130.1 (Ar-C), 129.83 (Ar-C), 129.80 (Ar-C), 129.76 (Ar-C), 129.5 (Ar-C), 129.4 (Ar-C), 129.2 (Ar-C), 128.9 (Ar-C), 128.7 (Ar-C), 128.5 (Ar-C), 128.4 (Ar-C), 128.3 (Ar-C), 123.3 (Ar-C), 117.3 (Ar-C), 100.2 (C1), 71.8 (C3), 71.7 (C5), 69.5 (C2), 68.0 (C4), 62.3 (C6); HRMS *m/z* (ES^+) Found: $(M+NH_4)^+$ 690.2340, $C_{40}H_{36}O_{10}N$ requires M^+ 690.2334. Data matched those reported previously.[28]

Phenyl 3,4,6-tri-*O*-benzoyl-β-D-galactopyranoside 38 & phenyl 2,3,6-tri-*O*-benzoyl-β-D-galactopyranoside 39

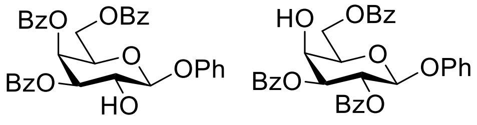

38: 23% **39: 34%**

38/39 were isolated as an inseparable mixture appearing as a colourless oil (98.6 mg, 0.173 mmol, **38/39**, 0.33:1.00); ratio determined from ^{1}H NMR integration values. The following were observed for both regio-isomers **38** and **39**: R_f = 0.38 (hexane/EtOAc, 7:3); ^{1}H NMR (400 MHz, $CDCl_3$) δ 8.12 – 7.92 (m, 7H, Ar*H*), 7.91 – 7.84 (m, 0.5H, Ar*H*), 7.63 – 7.55 (m, 1.5H, Ar*H*), 7.51 – 7.41 (m, 5.5H, Ar*H*), 7.40 – 7.27 (m, 4.5H, Ar*H*), 7.24 – 7.21 (m, 1H, Ar*H*), 7.18 – 7.11 (m, 2.5H, Ar*H*), 7.11 – 6.91 (m, 3.5H, Ar*H*); $^{13}C\{^{1}H\}$ NMR (101 MHz, $CDCl_3$) δ 133.7 (Ar-C), 133.6 (Ar-C), 133.4 (Ar-C), 133.33 (Ar-C), 133.27 (Ar-C), 130.03 (Ar-C), 129.95 (Ar-C), 129.86 (Ar-C), 129.81 (Ar-C), 129.77 (Ar-C), 129.61 (Ar-C), 129.60 (Ar-C), 129.48 (Ar-C), 129.45 (Ar-C), 129.4 (Ar-C), 129.2 (Ar-C), 129.1 (Ar-C), 128.9 (Ar-C), 128.64 (Ar-C), 128.55 (Ar-C), 128.51 (Ar-C), 128.49 (Ar-C), 128.41 (Ar-C), 128.35 (Ar-C), 123.23 (Ar-C), 123.20 (Ar-C), 117.32 (Ar-C), 116.99 (Ar-C); HRMS *m/z* (ES^+) Found: $(M+NH_4)^+$ 586.2079, $C_{33}H_{32}O_9N$ requires M^+ 586.2072; **39:** ^{1}H NMR (400 MHz, $CDCl_3$) δ 6.06 (dd, *J* = 10.3, 8.0 Hz, 1H, H-2), 5.43 (dd, *J* = 10.3, 3.2 Hz, 1H, H-3), 5.28 (d, *J* = 8.0 Hz, 1H, H-1), 4.75 (dd, *J* = 11.6, 5.4 Hz, 1H, H-6a), 4.66 (dd, *J* = 11.6, 7.4 Hz, 1H, H-6b), 4.43 – 4.38 (m, 1H, H-4), 4.26 – 4.21 (m, 1H, H-5); $^{13}C\{^{1}H\}$ NMR (101 MHz, $CDCl_3$) δ 166.4 (C=O, Bz), 165.9 (C=O, Bz), 165.4 (C=O, Bz), 100.0 (C1), 74.1 (C3), 72.8 (C5), 69.3 (C4), 67.3 (C2), 63.0 (C6); **38:** ^{1}H NMR (400 MHz, $CDCl_3$) δ 5.96 (dd, *J* = 3.5, 0.8 Hz, 1H, H-4), 5.50 (dd, *J* = 10.1, 3.5 Hz, 1H, H-3), 5.16 (d, *J* = 7.8 Hz, 1H, H-1), 4.61 (dd, *J* = 11.3, 7.6 Hz, 1H, H-6a), 4.48 (dd, *J* = 11.4, 5.4 Hz, 1H, H-6b), 4.45 – 4.43 (m, 2H, H-2, H-5); ^{13}C {1H} NMR (101 MHz, $CDCl_3$) δ 166.0 (C=O, Bz), 165.5 (C=O, Bz), 101.5 (C1), 73.3 (C3), 71.7 (C2), 69.7 (C5), 68.1 (C4), 62.4 (C6).

Benzoylation of ethyl β-D-galactopyranoside 40

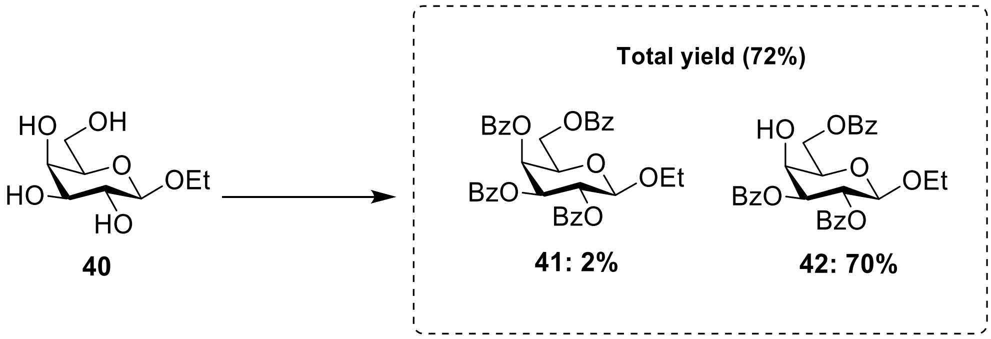

Following the general acylation procedure, ethyl β-D-galactopyranoside **40** (66.0 mg, 0.317 mmol, 1.0 equiv.) and BzCl (114 μL, 0.983 mmol, 3.1 equiv.) were reacted. Purification by column chromatography (hexane/EtOAc, 0-30%) yielded the below compounds in a total yield of 72%.

Ethyl 2,3,4,6-tri-*O*-benzoyl-β-D-galactopyranoside 41

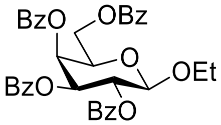

41 (4.00 mg, 6.4 μmol, 2%) as a colourless oil. R_f = 0.40 (hexane/EtOAc, 7:3); $[\alpha]_D^{19}$ = 31.0 (*c* = 1.0, $CHCl_3$); ^1H NMR (400 MHz, $CDCl_3$) δ 8.11 – 7.83 (m, 6H, Ar*H*), 7.79 – 7.62 (m, 2H, Ar*H*), 7.59 – 7.24 (m, 10H, Ar*H*), 7.19 – 7.14 (m, 2H, Ar*H*), 5.92 (dd, *J* = 3.5, 1.0 Hz, 1H, H-4), 5.72 (dd, *J* = 10.4, 7.9 Hz, 1H, H-2), 5.53 (dd, *J* = 10.4, 3.5 Hz, 1H, H-3), 4.76 (d, *J* = 8.0 Hz, 1H, H-1), 4.62 (dd, *J* = 11.2, 6.5 Hz, 1H, H-6a), 4.35 (dd, *J* = 11.2, 6.8 Hz, 1H, H-6b), 4.25 (td, *J* = 6.6, 1.0 Hz, 1H, H-5), 3.94 (dq, *J* = 9.8, 7.1 Hz, 1H, C*H*H), 3.62 (dq, *J* = 9.8, 7.0 Hz, 1H, C*H*H), 1.13 (t, *J* = 7.1 Hz, 3H, CH_3); $^{13}C\{^1H\}$ NMR (101 MHz, $CDCl_3$) δ 166.1 (C=O, Bz), 165.6 (C=O, Bz), 165.3 (C=O, Bz), 133.57 (Ar-C), 133.55 (Ar-C), 133.28 (Ar-C), 133.26 (Ar-C), 133.2 (Ar-C), 130.1 (Ar-C), 129.8 (Ar-C), 129.7 (Ar-C), 129.52 (Ar-C), 129.48 (Ar-C), 129.1 (Ar-C), 128.8 (Ar-C), 128.6 (Ar-C), 128.5 (Ar-C), 128.4 (Ar-C), 128.3 (Ar-C), 101.5 (C1), 71.8 (C3), 71.3 (C5), 69.9 (C2), 68.2 (C4), 66.0 (CH_2), 62.1 (C6), 15.1 (CH_3); HRMS *m/z* (ES^+) Found: $(M+H)^+$ 625.2074, $C_{36}H_{32}O_9$ requires M^+ 625.2073.

Ethyl 2,3,6-tri-*O*-benzoyl-β-D-galactopyranoside 42

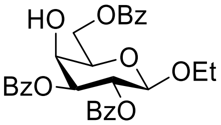

42 (115 mg, 0.221 mmol, 70%) as a colourless oil. R_f = 0.38 (hexane/EtOAc, 7:3); $[\alpha]_D^{24}$ = +26.0 (*c* = 1.0, $CHCl_3$); ^1H NMR (400 MHz, $CDCl_3$) δ 8.08 – 8.04 (m, 2H, Ar*H*), 8.01 – 7.95 (m, 4H, Ar*H*), 7.62 – 7.55 (m, 1H, Ar*H*), 7.55 – 7.49 (m, 2H, Ar*H*), 7.48 – 7.43 (m, 2H, Ar*H*), 7.41 – 7.36 (m, 4H, Ar*H*), 5.75 (dd, *J* = 10.3, 7.9 Hz, 1H, H-2), 5.35 (dd, *J* = 10.3, 3.2 Hz, 1H, H-3), 4.74 (d, *J* = 7.9 Hz, 1H, H-1), 4.70 (dd, *J* = 11.4, 6.6 Hz, 1H, H-6a), 4.61 (dd, *J* = 11.4, 6.4 Hz, 1H, H-6b), 4.35 (dd, *J* = 3.2, 0.7 Hz, 1H, H-4), 4.07 (td, *J* = 6.5, 0.8 Hz, 1H, H-5), 3.96 (dq, *J* = 9.8, 7.1 Hz, 1H, OC*H*H), 3.65 (dq, *J* = 9.8, 7.0 Hz, 1H, OC*H*H), 1.17 (t, *J* = 7.1 Hz, 3H, CH_3); $^{13}C\{^1H\}$ NMR (101 MHz, $CDCl_3$) δ 166.5 (C=O, Bz), 165.9 (C=O, Bz), 165.4 (C=O, Bz), 133.5 (Ar-C), 133.4 (Ar-C), 133.1 (Ar-C), 129.9 (Ar-C), 129.8 (Ar-C), 129.7 (Ar-C), 129.63 (Ar-C), 129.59 (Ar-C), 129.0 (Ar-C), 128.50 (Ar-C), 128.46 (Ar-C), 128.4 (Ar-C), 101.2 (C1), 74.2 (C3), 72.3 (C5), 69.6 (C2), 67.3 (C4), 65.6 (CH_2), 62.7 (C6), 15.1 (CH_3); HRMS m/z (ES^+) Found: $(M+NH_4)^+$ 538.2073, $C_{29}H_{32}O_9N$ requires M^+ 538.2072.

Cyclohexyl 3,6-di-*O*-benzoyl-1-thio-β-D-galactopyranoside 44

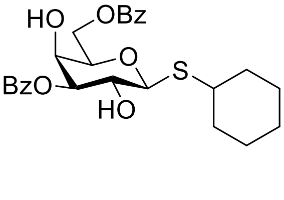

Following the general acylation procedure, cyclohexyl 1-thio-β-D-galactopyranoside **43** (66.0 mg, 0.237 mmol, 1.0 equiv.) and BzCl (85.4 μL, 0.735 mmol, 3.1 equiv.) were reacted. Purification by column chromatography (hexane/EtOAc, 0-30%) yielded the title compound **44** (74.0 mg, 0.152 mmol, 64%) as a white foam. R_f = 0.33 (hexane/EtOAc, 7:3); $[\alpha]_D^{23}$ = +15.5 (c = 1.0, $CHCl_3$); ^{1}H NMR (400 MHz, $CDCl_3$) δ 8.12 – 8.07 (m, 2H, Ar*H*), 8.06 – 7.98 (m, 2H, Ar*H*), 7.62 – 7.52 (m, 2H, Ar*H*), 7.51 – 7.40 (m, 4H, Ar*H*), 5.17 (dd, *J* = 9.6, 3.2 Hz, 1H, H-3), 4.65 – 4.50 (m, 3H, H-1, H-6a, H-6b), 4.28 – 4.24 (m, 1H, H-4), 4.02 (m, 2H, H-2, H-5), 2.96 (tt, *J* = 10.8, 3.7 Hz, 1H, CH), 2.53 – 2.47 (m, 2H, 2-OH, 4-OH), 2.06 – 1.96 (m, 2H, CH_2), 1.75 – 1.67 (m, 2H, CH_2), 1.61 – 1.56 (m, 1H, C*H*H), 1.51 – 1.35 (m, 2H, CH_2), 1.34 – 1.17 (m, 3H, CH_2, C*H*H); $^{13}C\{^1H\}$ NMR (101 MHz, $CDCl_3$) δ 166.4 (C=O, Bz), 166.1 (C=O, Bz), 133.5 (Ar-C), 133.3 (Ar-C), 129.9 (Ar-C), 129.7 (Ar-C), 129.6 (Ar-C), 129.5 (Ar-C), 128.50 (Ar-C), 128.45 (Ar-C), 86.6 (C1), 76.5 (C3), 76.1 (C2), 68.1 (C5), 67.6 (C4), 63.2 (C6), 44.6 (CH), 34.6 (CH_2), 33.9 (CH_2), 26.0 (CH_2), 25.5 (CH_2); HRMS *m/z* (ES^+) Found: $(M+Na)^+$ 509.1584, $C_{26}H_{30}O_7SNa$ requires M^+ 509.1609.

Benzoylation of trifluoroethyl 1-thio-β-D-galactopyranoside 45

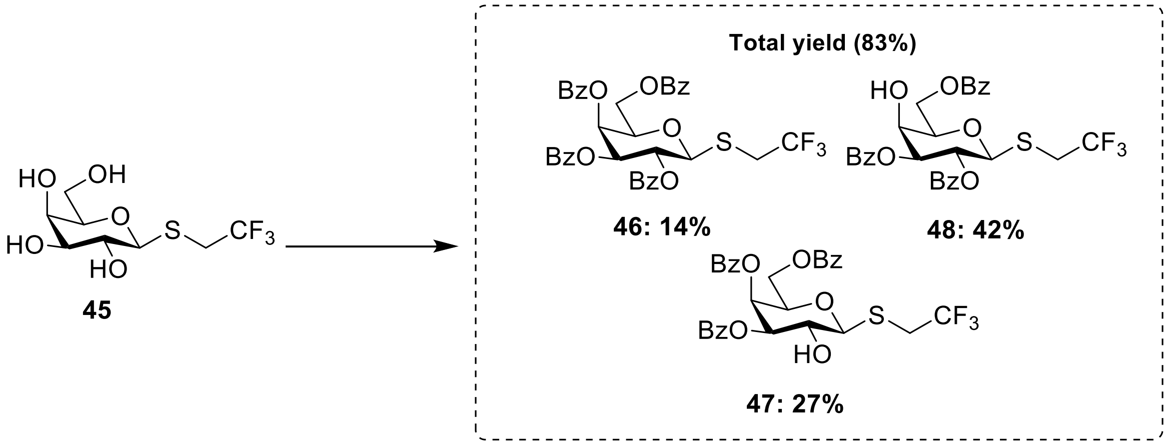

Following the general acylation procedure, trifluoroethyl 1-thio-β-D-galactopyranoside **45** (107 mg, 0.385 mmol, 1.0 equiv.) and BzCl (138 μL, 1.19 mmol, 3.1 equiv.) were reacted. Purification by column chromatography (hexane/EtOAc, 0-40%) yielded the below compounds in a total yield of 83%.

Trifluoroethyl 2,3,4,6-tetra-*O*-benzoyl-1-thio-β-D-galactopyranoside 46

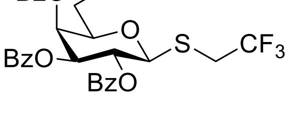

46 (38.0 mg, 54.7 μmol), 14%) as an off-white foam. $[\alpha]_D^{23}$ = +94.1 (c = 1.0, $CHCl_3$); R_f = 0.31 (hexane/EtOAc, 6:4); ^{1}H NMR (400 MHz, $CDCl_3$) δ 8.03 – 7.96 (m, 2H, Ar*H*), 7.96 – 7.90 (m, 2H, Ar*H*), 7.89 – 7.83 (m, 2H, Ar*H*), 7.73 – 7.64 (m, 2H, Ar*H*), 7.60 – 7.51 (m, 1H, Ar*H*), 7.51 – 7.40 (m, 4H, Ar*H*), 7.39 – 7.27 (m, 5H, Ar*H*), 7.22 – 7.09 (m, 2H, Ar*H*), 5.97 (d, *J* = 2.7 Hz, 1H, H-4), 5.72 (t, *J* = 9.9 Hz, 1H, H-2), 5.59 (dd, *J* = 9.9, 3.4 Hz, 1H, H-3), 4.96 (d, *J* = 9.9 Hz,

1H, H-1), 4.57 (td, *J* = 10.9, 4.5 Hz, 1H, H-6a), 4.43 – 4.23 (m, 2H, H-6b, H-5), 3.47 (dq, *J* = 15.5, 9.7 Hz, 1H, SC*H*H), 3.13 (dq, *J* = 15.5, 10.0 Hz, 1H, SC*H*H); $^{13}C\{^{1}H\}$ NMR (101 MHz, $CDCl_3$) δ 166.0 (C=O, Bz), 165.49 (C=O, Bz), 165.46 (C=O, Bz), 133.8 (Ar-C), 133.6 (Ar-C), 133.41 (Ar-C), 133.40 (Ar-C), 130.0 (Ar-C), 129.9 (Ar-C), 129.78 (Ar-C), 129.75 (Ar-C), 129.3 (Ar-C), 128.9 (Ar-C), 128.8 (Ar-C), 128.74 (Ar-C), 128.66 (Ar-C), 128.51 (Ar-C), 128.49 (Ar-C), 128.4 (Ar-C), 125.3 (d, $^{1}J_{C\text{-}F}$ = 276.4 Hz, CF_3), 82.8 (C1), 75.6 (C5), 72.5 (C3), 68.6 (C2), 68.3 (C4), 62.2 (C6), 31.6 (q, $^{2}J_{C\text{-}F}$ = 33.7 Hz, CH_2); ^{19}F NMR (376 MHz, $CDCl_3$) δ -65.98 (t, *J* = 9.8 Hz); HRMS m/z (ES^+) Found: $(M+NH_4)^+$ 712.1813, $C_{36}H_{33}F_3O_9SN$ requires M^+ 712.1828.

Trifluoroethyl 2,3,6-tri-*O*-benzoyl-1-thio-β-D-galactopyranoside 48 & trifluoroethyl 3,4,6-tri-*O*-benzoyl-1-thio-β-D-galactopyranoside 47

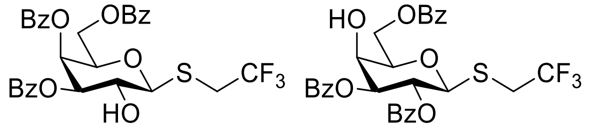

47: 27% **48: 42%**

48/47 were isolated as an inseparable mixture as a yellow foam (158 mg, 0.268 mmol, **48/47**, 1.55:1.0); ratio determined from ^{19}F NMR integration values. R_f = 0.26 (hexane/EtOAc, 6:4); ^{1}H NMR (400 MHz, $CDCl_3$) δ 8.12 – 7.94 (m, 8H, Ar*H*), 7.91 – 7.80 (m, 1H, Ar*H*), 7.72 – 7.26 (m, 14H, Ar*H*); $^{13}C\{^{1}H\}$ NMR (101 MHz, $CDCl_3$) δ 133.7 (Ar-C), 133.6 (Ar-C), 133.5 (Ar-C), 133.4 (Ar-C), 129.91 (Ar-C), 129.86 (Ar-C), 129.84 (Ar-C), 129.75 (Ar-C), 129.7 (Ar-C), 129.4 (Ar-C), 129.3 (Ar-C), 129.1 (Ar-C), 129.04 (Ar-C), 129.01 (Ar-C), 128.8 (Ar-C), 128.7 (Ar-C), 128.52 (Ar-C), 128.49 (Ar-C), 128.45 (Ar-C), 128.4 (Ar-C); HRMS *m/z* (ES^+) Found: $(M+NH_4)^+$ 608.1556, $C_{29}H_{29}F_3O_8SN$ requires M^+ 608.1566; **48:** ^{1}H NMR (400 MHz, $CDCl_3$) δ 5.80 (t, *J* = 9.9 Hz, 1H, H-2), 5.42 (dd, *J* = 9.9, 3.1 Hz, 1H, H-3), 4.91 (d, *J* = 10.0 Hz, 1H, H-1), 4.69 (dd, *J* = 11.6, 5.8 Hz, 1H, H-6a), 4.63 – 4.58 (m, 1H, H-6b), 4.43 – 4.40 (m, 1H, H-4), 4.12 (t, *J* = 6.3 Hz, 1H, H-5), 3.61 – 3.42 (m, 1H, SC*H*H), 3.16 (dq, *J* = 15.4, 10.1 Hz, 1H, SC*H*H), 2.94 (d, *J* = 4.6 Hz, 1H, 4-OH); $^{13}C\{^{1}H\}$ NMR (101 MHz, $CDCl_3$) δ 166.5 (C=O, Bz), 165.8 (C=O, Bz), 165.6 (C=O, Bz), 125.4 (q, $^{1}J_{C\text{-}F}$ = 276.3 Hz, CF_3), 84.7 (C1), 75.4 (C5), 74.6 (C3), 69.4 (C2), 68.5 (C4), 62.3 (C6), 31.3 (q, $^{2}J_{C\text{-}F}$ = 33.5 Hz, CH_2); ^{19}F NMR (376 MHz, $CDCl_3$) δ -66.16 (t, *J* = 9.9 Hz); **47:** ^{1}H NMR (400 MHz, $CDCl_3$) δ 5.95 (dd, *J* = 3.4, 0.7 Hz, 1H, H-4), 5.43 – 5.40 (m, 1H, H-3), 4.80 (d, *J* = 9.6 Hz, 1H, H-1), 4.60 – 4.58 (m, 1H, H-6a), 4.38 (dd, *J* = 11.4, 5.8 Hz, 1H, H-6b), 4.32 – 4.28 (m, 1H, H-5), 4.16 (dd, *J* = 9.3, 5.1 Hz, H-2), 3.58 – 3.43 (m, 1H, SC*H*H), 3.37 – 3.23 (m, 1H, SC*H*H), 2.88 (d, *J* = 4.3 Hz, 1H, 2-OH); $^{13}C\{^{1}H\}$ NMR (101 MHz, $CDCl_3$) δ 166.12 (C=O, Bz), 166.06 (C=O, Bz), 165.4 (C=O, Bz), 125.4 (q, $^{1}J_{C\text{-}F}$ = 276.3 Hz, CF_3), 84.7 (C1), 75.4 (C5), 74.6 (C3), 69.4 (C2), 68.5 (C4), 62.3 (C6), 31.3 (q, $^{2}J_{C\text{-}F}$ = 33.5 Hz, CH_2); ^{19}F NMR (376 MHz, $CDCl_3$) δ -66.16 (t, *J* = 9.9 Hz).

Benzoylation of cyclohexyl β-D-galactopyranoside 49

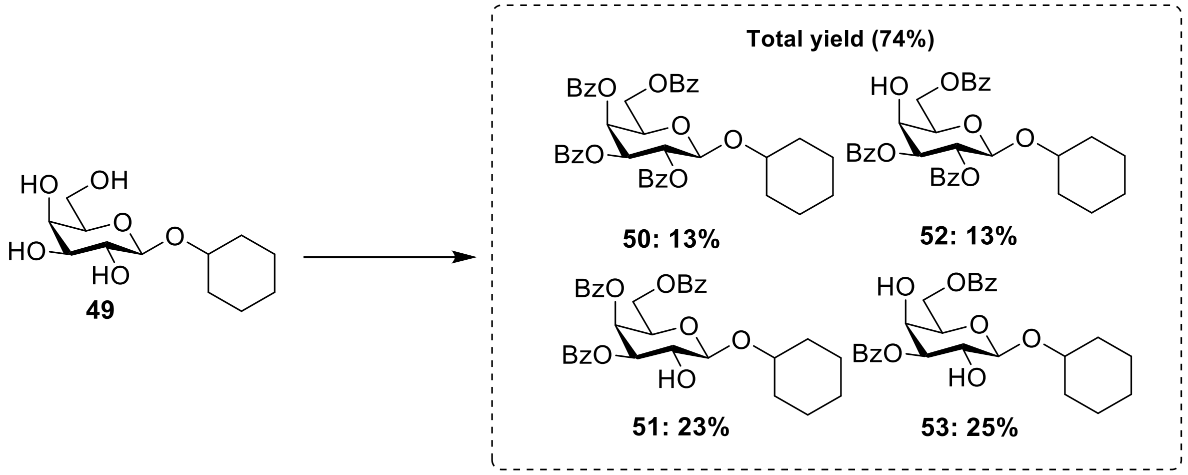

Following the general acylation procedure, cyclohexyl β-D-galactopyranoside **49** (97.0 mg, 0.370 mmol, 1.0 equiv.) and BzCl (134 µL, 1.15 mmol, 3.1 equiv.) were reacted. Purification by column chromatography (hexane/EtOAc, 0-40%) yielded the below compounds in a total yield of 74%.

Cyclohexyl 2,3,4,6-tetra-*O*-benzoyl-β-D-galactopyranoside 50

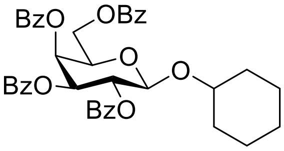

50 (33.9 mg, 49.9 µmol, 13%) as a white solid. R_f = 0.71 (hexane/EtOAc, 7:3); ^{1}H NMR (400 MHz, $CDCl_3$) δ 8.05 – 8.00 (m, 2H, Ar*H*), 7.97 – 7.94 (m, 2H, Ar*H*), 7.91 – 7.87 (m, 2H, Ar*H*), 7.72 (dd, *J* = 8.3, 1.2 Hz, 2H, Ar*H*), 7.57 – 7.45 (m, 2H, Ar*H*), 7.44 – 7.26 (m, 8H, Ar*H*), 7.19 – 7.13 (m, 2H, Ar*H*), 5.91 (dd, *J* = 3.4, 0.8 Hz, 1H, H-4), 5.70 (dd, *J* = 10.4, 8.0 Hz, 1H, H-2), 5.52 (dd, *J* = 10.4, 3.5 Hz, 1H, H-3), 4.83 (d, *J* = 8.0 Hz, 1H, H-1), 4.60 (dd, *J* = 11.2, 6.8 Hz, 1H, H-6a), 4.35 (dd, *J* = 11.2, 6.6 Hz, 1H, H-6b), 4.24 (td, *J* = 6.6, 0.7 Hz, 1H, H-5), 3.79 – 3.41 (m, 1H, CH), 1.95 – 1.82 (m, 1H, C*H*H), 1.75 – 1.35 (m, 6H, 3 × CH_2), 1.34 – 0.95 (m, 3H, CH_2, CH); $^{13}C\{^1H\}$ NMR (101 MHz, $CDCl_3$) δ 166.1 (C=O, Bz), 165.72 (C=O, Bz), 165.65 (C=O, Bz), 165.2 (C=O, Bz), 133.6 (Ar-C), 133.3 (Ar-C), 133.2 (Ar-C), 133.1 (Ar-C), 130.1 (Ar-C), 129.81 (Ar-C), 129.78 (Ar-C), 129.7 (Ar-C), 129.6 (Ar-C), 129.5 (Ar-C), 129.1 (Ar-C), 128.9 (Ar-C), 128.6 (Ar-C), 128.5 (Ar-C), 128.4 (Ar-C), 128.3 (Ar-C), 100.4 (C1), 78.8 (CH), 71.3 (C3), 70.0 (C5), 68.2 (C2), 62.1 (C6), 33.4 (CH_2), 31.7 (CH_2), 25.4 (CH_2), 23.9 (CH_2), 23.7 (CH_2); HRMS *m/z* (ES^+) Found: $(M+NH_4)^+$ 696.2803, $C_{40}H_{42}O_{10}N$ requires M^+ 696.2808. Data matched those reported previously.[29]

Cyclohexyl 3,4,6-tri-*O*-benzoyl-β-D-galactopyranoside 51 & cyclohexyl 2,3,6-tri-*O*-benzoyl-β-D-galactopyranoside 52

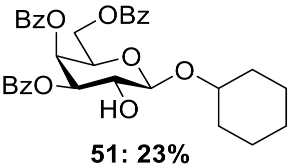

51: 23%

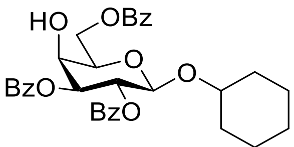

52: 13%

51/52 were isolated as an inseparable mixture appearing as a white foam (78.3 mg, 0.14 mmol, **51/52**, 1.0:0.55); ratio determined from ^{1}H NMR integration values. R_f = 0.40 (hexane/EtOAc, 7:3); The following were observed for both regioisomers **51** and **52**: ^{1}H NMR (400 MHz, $CDCl_3$) δ 8.16 – 7.80 (m, 10H, Ar*H*), 7.65 – 7.27 (m, 13H, Ar*H*), 2.00 – 1.88 (m, 2H), 1.89 – 1.76 (m, 0.5H), 1.74 – 1.26 (m, 8.5H), 1.26 – 1.00 (m, 7H); $^{13}C\{^{1}H\}$ NMR (101 MHz, $CDCl_3$) δ 130.2 (Ar-C), 130.1 (Ar-C), 129.94 (Ar-C), 129.88 (Ar-C), 129.82 (Ar-C), 129.78 (Ar-C), 129.77 (Ar-C), 129.7 (Ar-C), 129.50 (Ar-C), 129.45 (Ar-C), 129.2 (Ar-C), 128.7 (Ar-C), 128.59 (Ar-C), 128.57 (Ar-C), 128.5 (Ar-C), 128.4 (Ar-C); HRMS *m/z* (ES^+) Found: $(M+NH_4)^+$ 592.2536, $C_{33}H_{38}O_9N$ requires M^+ 592.2546; **51:** ^{1}H NMR (400 MHz, $CDCl_3$) δ 5.89 (dd, *J* = 3.7, 1.2 Hz, 1H, H-4), 5.42 (dd, *J* = 10.2, 3.6 Hz, 1H, H-3), 4.70 – 4.59 (m, 2H, H-1, H-6a), 4.40 – 4.35 (m, 1H, H-6b), 4.23 (td, *J* = 6.7, 1.2 Hz, 1H, H-5), 4.10 (dd, *J* = 10.2, 7.7 Hz, 1H, H-2), 3.74 (tt, *J* = 9.8, 4.0 Hz, 1H, CH); $^{13}C\{^{1}H\}$ NMR (101 MHz, $CDCl_3$) δ 166.2 (C=O, Bz), 166.0 (C=O, Bz), 165.8 (C=O, Bz), 102.0 (C1), 79.0 (CH), 73.3 (C3), 71.4 (C5), 70.0 (C2), 68.4 (C4), 62.3 (C6), 33.8 (CH_2), 32.1 (CH_2), 25.6 (CH_2), 24.34 (CH_2), 24.27 (CH_2); **52:** ^{1}H NMR (400 MHz, $CDCl_3$) δ 5.74 (dd, *J* = 10.3, 7.9 Hz, 1H, H-2), 5.34 (dd, *J* = 10.3, 3.2 Hz, 1H, H-3), 4.81 (d, *J* = 7.9 Hz, 1H, H-1), 4.69 – 4.57 (m, 2H, H-6a, H-6b), 4.36 – 4.33 (m, 1H, H-4), 4.09 – 4.03 (m, 1H, H-5), 3.65 (td, *J* = 9.0, 4.3 Hz, 1H, CH); $^{13}C\{^{1}H\}$ NMR (101 MHz, $CDCl_3$) δ 166.5 (C=O, Bz), 166.1 (C=O, Bz), 165.5 (C=O, Bz), 100.3 (C1), 78.5 (CH), 74.5 (C3), 72.4 (C5), 70.0 (C2), 67.5 (C4), 62.8 (C6), 33.4 (CH_2), 31.8 (CH_2), 25.5 (CH_2), 23.9 (CH_2), 23.7 (CH_2).

Cyclohexyl 3,6-di-*O*-benzoyl-β-D-galactopyranoside 53

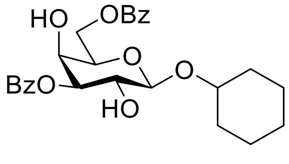

53 (43.2 mg, 91.8 µmol, 25%) as a colourless oil; R_f = 0.26 (hexane/EtOAc, 7:3); $[\alpha]_D^{24}$ = +13.5 (*c* = 1.0, $CHCl_3$); ^{1}H NMR (400 MHz, $CDCl_3$) δ 8.04 – 7.98 (m, 2H, Ar*H*), 7.99 – 7.93 (m, 2H, Ar*H*), 7.54 – 7.46 (m, 2H, Ar*H*), 7.42 – 7.32 (m, 4H, Ar*H*), 5.08 (dd, *J* = 10.1, 3.3 Hz, 1H, H-3), 4.60 – 4.47 (m, 2H, H-6a, H-6b), 4.45 (d, *J* = 7.7 Hz, 1H, H-1), 4.19 – 4.13 (m, 1H, H-4), 3.96 (dd, *J* = 10.1, 7.7 Hz, 1H, H-2), 3.89 (td, *J* = 6.5, 0.6 Hz, 1H, H-5), 3.68 – 3.54 (m, 1H, CH), 1.98 – 1.80 (m, 2H, CH_2), 1.71 – 1.58 (m, 2H, CH_2), 1.47 – 1.05 (m, 6H, 3 × CH_2); $^{13}C\{^{1}H\}$ NMR (101 MHz, $CDCl_3$) δ 166.4 (C=O, Bz), 166.1 (C=O, Bz), 133.6 (Ar-C), 133.4 (Ar-C), 133.3 (Ar-C), 130.2 (Ar-C), 129.9 (Ar-C), 129.7 (Ar-C), 129.6 (Ar-C), 128.5 (Ar-C), 101.9 (C1), 78.5 (CH), 75.4 (C3), 72.3 (C5), 69.4 (C2), 67.4 (C4), 62.8 (C6), 33.6 (CH_2), 32.0 (CH_2), 25.5 (CH_2), 24.13 (CH_2), 24.07 (CH_2); HRMS *m/z* (ES^+) Found: $(M+NH_4)^+$ 493.1843, $C_{26}H_{34}O_8N$ requires M^+ 493.1838.

Benzoylation of trifluoroethyl β-D-galactopyranoside 54

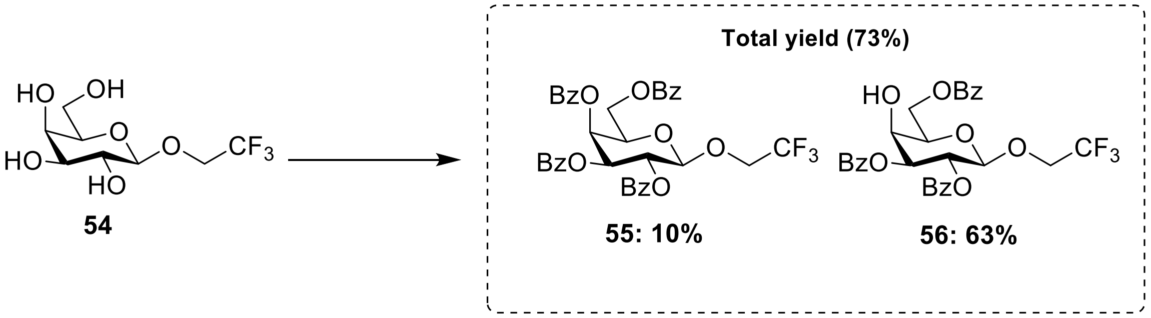

Following the general acylation procedure, trifluoroethyl β-D-galactopyranoside **54** (121 mg, 0.462 mmol, 1.0 equiv.) and BzCl (167 μL, 1.43 mmol, 3.1 equiv.) were reacted. Purification by column chromatography (hexane/EtOAc, 0-40%) yielded the below mix of compounds in a total yield of 73%.

Trifluoroethyl 2,3,4,6-tetra-*O*-benzoyl-β-D-galactopyranoside 55

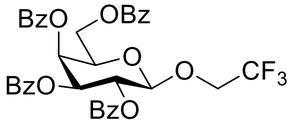

55 (32.0 mg, 47.2 μmol, 10%) as a colourless oil. R_f = 0.57 (hexane/EtOAc, 7:3); $[\alpha]_D^{23}$ = -68.8 (*c* = 1.0, $CHCl_3$); ^{1}H NMR (400 MHz, $CDCl_3$) δ 8.14 – 8.05 (m, 2H, Ar*H*), 8.05 – 8.00 (m, 2H, Ar*H*), 7.99 – 7.91 (m, 2H, Ar*H*), 7.82 – 7.77 (m, 2H, Ar*H*), 7.68 – 7.54 (m, 3H, Ar*H*), 7.54 – 7.31 (m, 9H, Ar*H*), 6.01 (dd, *J* = 3.4, 0.9 Hz, 1H, H-4), 5.83 (dd, *J* = 10.4, 7.9 Hz, 1H, H-2), 5.61 (dd, *J* = 10.4, 3.4 Hz, 1H, H-3), 4.99 (d, *J* = 7.9 Hz, 1H, H-1), 4.69 (dd, *J* = 11.2, 6.4 Hz, 1H, H-6a), 4.44 (dd, *J* = 11.2, 6.5 Hz, 1H, H-6b), 4.37 (td, *J* = 6.4, 0.9 Hz, 1H, H-5), 4.21 (dq, *J* = 12.8, 8.7 Hz, 1H, OC*H*H), 4.15 – 4.03 (m, 1H, OC*H*H); $^{13}C\{^1H\}$ NMR (101 MHz, $CDCl_3$) δ 166.1 (C=O, Bz), 165.54 (C=O, Bz), 165.48 (C=O, Bz), 165.3 (C=O, Bz), 133.8 (Ar-C), 133.7 (Ar-C), 133.4 (Ar-C), 133.3 (Ar-C), 130.2 (Ar-C), 130.0 (Ar-C), 129.83 (Ar-C), 129.79 (Ar-C), 129.7 (Ar-C), 129.1 (Ar-C), 128.9 (Ar-C), 128.70 (Ar-C), 128.65 (Ar-C), 128.5 (Ar-C), 128.4 (Ar-C), 128.3 (Ar-C), 123.4 (q, ^1J = 279.1 Hz, CF_3), 101.4 (C1), 71.8 (C5), 71.5 (C3), 69.3 (C2), 67.9 (C4), 65.8 (q, ^2J = 35.0 Hz, CH_2), 61.9 (C6); ^{19}F NMR (376 MHz, $CDCl_3$) δ -74.2 (t, *J* = 8.5 Hz); HRMS *m/z* (ES^+) Found: $(M+Na)^+$ 701.1584, $C_{36}H_{29}F_3O_{10}Na$ requires M^+ 701.1610.

Trifluoroethyl 2,3,6-tri-*O*-benzoyl-β-D-galactopyranoside 56

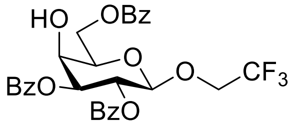

56 (167 mg, 0.291 mmol, 63%) as a white foam. R_f = 0.51 (hexane/EtOAc, 7:3); $[\alpha]_D^{23}$ = -50.0 (*c* = 1.0, $CHCl_3$); ^{1}H NMR (400 MHz, $CDCl_3$) δ 8.06 – 8.03 (m, 2H, Ar*H*), 7.99 – 7.94 (m, 4H, Ar*H*), 7.62 – 7.56 (m, 1H, Ar*H*), 7.56 – 7.44 (m, 4H, Ar*H*), 7.44 – 7.33 (m, 4H, Ar*H*), 5.83

(dd, J = 10.3, 7.9 Hz, 1H, H-2), 5.37 (dd, J = 10.3, 3.2 Hz, 1H, H-3), 4.91 (d, J = 8.0 Hz, 1H, H-1), 4.72 (dd, J = 11.5, 6.3 Hz, 1H, H-6a), 4.62 (dd, J = 11.5, 5.2 Hz, 1H, H-6b), 4.39 (dd, J = 3.1, 0.7 Hz, 1H, H-4), 4.19 – 4.00 (m, 3H, CH_2, H-5); $^{13}C\{^1H\}$ NMR (101 MHz, $CDCl_3$) δ 166.5 (C=O, Bz), 165.8 (C=O, Bz), 165.5 (C=O, Bz), 133.6 (Ar-C), 133.5 (Ar-C), 133.3 (Ar-C), 130.2 (Ar-C), 129.9 (Ar-C), 129.8 (Ar-C), 129.7 (Ar-C), 129.4 (Ar-C), 129.3 (Ar-C), 128.8 (Ar-C), 128.5 (Ar-C), 128.4 (Ar-C), 123.5 (q, $^1J_{C\text{-}F}$ = 278.9 Hz, CF_3), 101.1 (C1), 73.8 (C3), 72.8 (C5), 68.9 (C2), 67.1 (C4), 65.3 (q, $^2J_{C\text{-}F}$ = 35.0 Hz, CH_2), 62.6 (C6); ^{19}F NMR (376 MHz, $CDCl_3$) δ -74.2 (t, J = 8.5 Hz); HRMS *m/z* (ES^+) Found: $(M+NH_4)^+$ 592.1786, $C_{29}H_{29}F_3O_9N$ requires M^+ 592.1794.

Benzoylation of *p*-(trifluoromethyl)-phenyl 1-thio-β-D-galactopyranoside 57

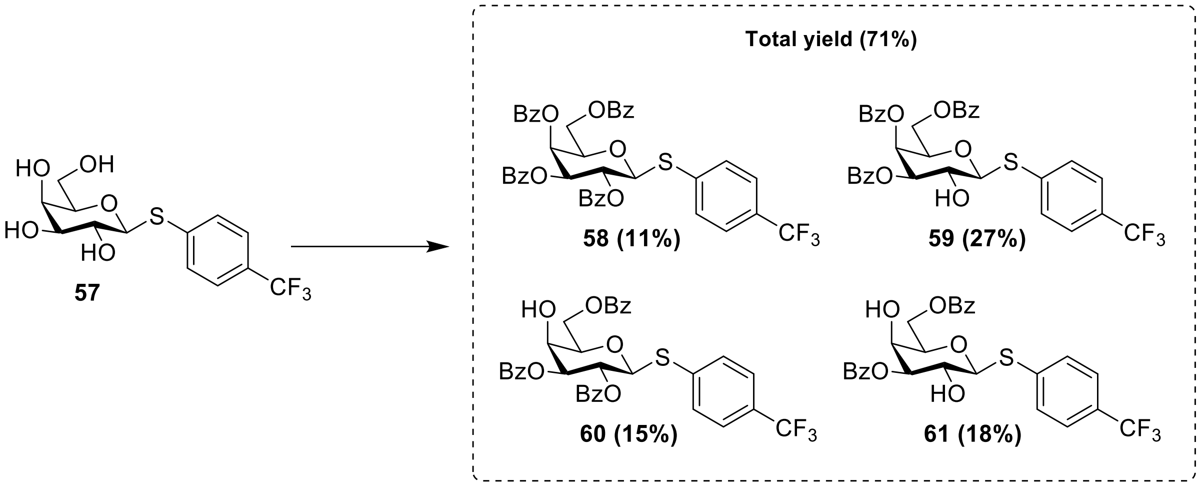

Following the general acylation procedure, *p*-(trifluoromethyl)-phenyl 1-thio-β-D-galactopyranoside **57** (156 mg, 0.458 mmol, 1.0 equiv.) and BzCl (165 µL, 1.42 mmol, 3.1 equiv.) were reacted. Purification by column chromatography (hexane/EtOAc, 0-40%) yielded the below compounds in a total yield of 71%.

p-(Trifluoromethyl)-phenyl-2,3,4,6-tetra-*O*-benzoyl-1-thio-β-D-galactopyranoside 58

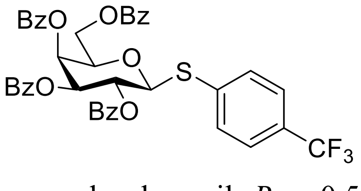

58 (39.0 mg, 51.5 µmol, 11%) as a colourless oil. R_f = 0.58 (hexane/EtOAc, 7:3); $[\alpha]_D^{22}$ = +36.0 (c = 1.0, $CHCl_3$); ^1H NMR (400 MHz, $CDCl_3$) δ 8.07 – 8.02 (m, 2H, Ar*H*), 8.00 – 7.91 (m, 4H, Ar*H*), 7.79 – 7.72 (m, 2H, Ar*H*), 7.69 – 7.51 (m, 5H, Ar*H*), 7.50 – 7.37 (m, 9H, Ar*H*), 7.25 – 7.21 (m, 2H, Ar*H*), 6.03 (dd, J = 3.3, 0.6 Hz, 1H, H-4), 5.81 (t, J = 9.9 Hz, 1H, H-2), 5.66 (dd, J = 9.9, 3.3 Hz, 1H, H-3), 5.11 (d, J = 9.9 Hz, 1H, H-1), 4.66 (dd, J = 11.5, 7.3 Hz, 1H, H-6a), 4.51 (dd, J = 11.5, 5.0 Hz, 1H, H-6b), 4.47 – 4.42 (m, 1H, H-5); $^{13}C\{^1H\}$ NMR (101 MHz, $CDCl_3$) δ 166.1 (C=O, Bz), 165.5 (2× C=O, Bz), 165.2 (C=O, Bz), 136.9 (Ar-C), 133.8 (Ar-C), 133.53 (Ar-C), 133.49 (Ar-C), 133.4 (Ar-C), 132.5 (Ar-C), 130.2 (Ar-C), 129.93 (Ar-C), 129.85 (Ar-C), 129.80 (Ar-C), 129.77 (Ar-C), 129.4 (Ar-C), 129.0 (Ar-C), 128.8 (Ar-

C), 128.7 (Ar-C), 128.6 (Ar-C), 128.54 (Ar-C), 128.51 (Ar-C), 128.3 (Ar-C), 125.69 (Ar-C), 125.65 (Ar-C), 125.62 (Ar-C), 125.58 (Ar-C), 125.3 (Ar-C), 122.6 (Ar-C), 85.4 (C1), 75.5 (C5), 72.7 (C3), 68.4 (C4), 67.8 (C2), 62.7 (C6); ^{19}F NMR (376 MHz, $CDCl_3$) δ -62.6 (s); HRMS *m/z* (ES^+) Found: $(M+NH_4)^+$ 774.1991, $C_{41}H_{35}F_3O_9NS$ requires M^+ 774.1979.

p-(Trifluoromethyl)-phenyl-3,4,6-tri-*O*-benzoyl-1-thio-β-D-galactopyranoside 59 & *p*-(trifluoromethyl)-phenyl 2,3,6-tri-*O*-benzoyl-1-thio-β-D-galactopyranoside 60

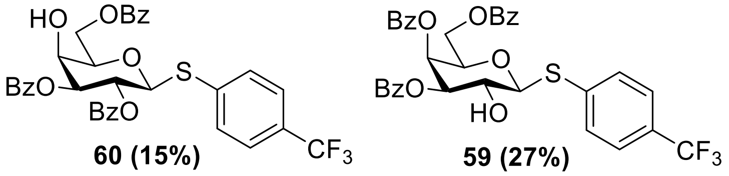

60 (15%) **59 (27%)**

59/60 were isolated as an inseparable mixture appearing as a colourless oil (126 mg, 0.193 mmol, **59**/**60**, 1:0.49, 42%); ratio determined from ^{1}H NMR integration values. The following were observed for both regioisomers **59** and **60**: R_f = 0.30 (hexane/EtOAc,); ^{1}H NMR (400 MHz, $CDCl_3$) δ 8.14 – 8.03 (m, 2H, Ar*H*), 8.04 – 7.98 (m, 2H, Ar*H*), 7.98 – 7.90 (m, 4H, Ar*H*), 7.86 – 7.78 (m, 2H, Ar*H*), 7.76 – 7.71 (m, 2H, Ar*H*), 7.67 – 7.26 (m, 16.5H Ar*H*); $^{13}C\{^1H\}$ NMR (101 MHz, $CDCl_3$) δ 166.5 (Ar-C), 166.14 (Ar-C), 166.10 (Ar-C), 165.8 (Ar-C), 165.5 (Ar-C), 133.8 (Ar-C), 133.7 (Ar-C), 133.61 (Ar-C), 133.55 (Ar-C), 133.5 (Ar-C), 132.21 (Ar-C), 132.18 (Ar-C), 130.8 (Ar-C), 130.2 (Ar-C), 129.9 (Ar-C), 129.84 (Ar-C), 129.79 (Ar-C), 129.5 (Ar-C), 129.4 (Ar-C), 129.1 (Ar-C), 128.63 (Ar-C), 128.57 (Ar-C), 128.52 (Ar-C), 128.49 (Ar-C), 128.4 (Ar-C), 125.79 (Ar-C), 125.75 (Ar-C), 125.70 (Ar-C), 125.66 (Ar-C); HRMS *m/z* (ES^+) Found: $(M+NH_4)^+$ 670.1726, $C_{34}H_{31}F_3O_8NS$ requires M^+ 670.1717; **59:** ^{1}H NMR (400 MHz, $CDCl_3$) δ 5.95 (dd, *J* = 3.4, 1.1 Hz, 1H, H-4), 5.44 (dd, *J* = 9.5, 3.4 Hz, 1H, H-3), 4.88 (d, *J* = 9.7 Hz, 1H, H-1), 4.63 (dd, *J* = 11.5, 7.3 Hz, 1H, H-6a), 4.45 (dd, *J* = 11.6, 5.1 Hz, 1H, H-6b), 4.39 – 4.31 (m, 1H, H-5), 4.13 (t, *J* = 9.6 Hz, 1H, H-2); $^{13}C\{^1H\}$ NMR (101 MHz, $CDCl_3$) δ 87.6 (C1), 75.4 (C5), 74.7 (C3), 68.7 (C4), 67.9 (C2), 62.8 (C6); **60:** ^{1}H NMR (400 MHz, $CDCl_3$) δ 5.86 (t, *J* = 10.0 Hz, 1H, H-2), 5.43 (dd, *J* = 9.8, 3.1 Hz, 1H, H-3), 5.05 (d, *J* = 10.1 Hz, 1H, H-1), 4.72 (dd, *J* = 11.8, 5.0 Hz, 1H, H-6a), 4.66 (dd, *J* = 11.8, 7.4 Hz, 1H, H-6b), 4.47 – 4.41 (m, 1H, H-4), 4.20 (ddd, *J* = 7.4, 4.9, 1.1 Hz, 1H, H-5); $^{13}C\{^1H\}$ NMR (101 MHz, $CDCl_3$) δ 85.8 (C1), 76.6 (C5), 75.1 (C3), 67.8 (C2), 67.7 (C4), 63.7 (C6).

p-(Trifluoromethyl)-phenyl 3,6-di-*O*-benzoyl-1-thio-β-D-galactopyranoside 61

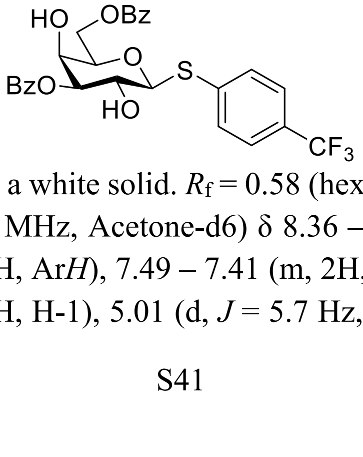

61 (45.0 mg, 82.0 μmol, 18%) as a white solid. R_f = 0.58 (hexane/EtOAc, 7:3); $[\alpha]_D^{22}$ = -6.6 (*c* = 1.0, Acetone); ^{1}H NMR (400 MHz, Acetone-d6) δ 8.36 – 8.06 (m, 4H, Ar*H*), 7.78 – 7.60 (m, 4H, Ar*H*), 7.60 – 7.50 (m, 4H, Ar*H*), 7.49 – 7.41 (m, 2H, Ar*H*), 5.22 (dd, *J* = 9.6, 3.3 Hz, 1H, H-3), 5.19 (d, *J* = 9.8 Hz, 1H, H-1), 5.01 (d, *J* = 5.7 Hz, 1H, 2-OH), 4.84 (d, *J* = 5.4 Hz,

1H, 4-OH), 4.67 (dd, *J* = 11.5, 8.1 Hz, 1H, H-6a), 4.57 (dd, *J* = 11.5, 4.0 Hz, 1H, H-6b), 4.45 – 4.43 (m, 1H, H-4), 4.42 – 4.37 (m, 1H, H-5), 4.30 – 4.22 (m, 1H, H-2); $^{13}C\{^1H\}$ NMR (101 MHz, Acetone-d6) δ 165.7 (C=O, Bz), 165.6 (C=O, Bz), 133.2 (Ar-C), 133.1 (Ar-C), 130.4 (Ar-C), 130.2 (Ar-C), 129.7 (Ar-C), 129.4 (Ar-C), 129.1 (Ar-C), 128.6 (Ar-C), 128.4 (Ar-C), 125.5 (Ar-C), 125.42 (Ar-C), 125.39 (Ar-C), 125.35 (Ar-C), 86.8 (C1), 77.9 (C3), 76.3 (C5), 67.2 (C4), 67.1 (C2), 64.3 (C6); HRMS *m/z* (ES^-) Found: $(M-H)^-$ 547.1035, $C_{27}H_{22}F_3O_7S$ requires M^- 547.1044.

Benzoylation of *p*-(Methoxy)-phenyl 1-thio-β-D-galactopyranoside 62

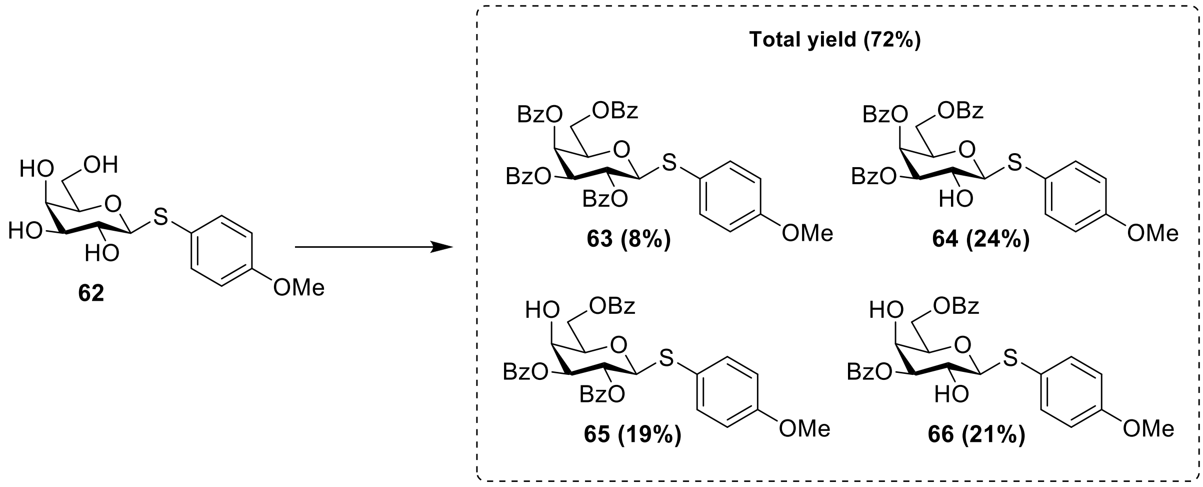

Following the general acylation procedure, *p*-(methoxy)-phenyl 1-thio-β-D-galactopyranoside **62** (138 mg, 0.516 mmol, 1.0 equiv.) and BzCl (165 µL, 1.42 mmol, 3.1 equiv.) were reacted. Purification by column chromatography (hexane/EtOAc, 0-40%) yielded the below compounds in a total yield of 72%.

p-(Methoxy)-phenyl 2,3,4,6-tetra-*O*-benzoyl-1-thio-β-D-galactopyranoside 63

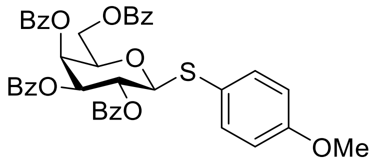

63 (28.0 mg, 39.0 µmol, 8%) as a colourless oil. R_f = 0.58 (hexane/EtOAc, 7:3); ^1H NMR (400 MHz, $CDCl_3$) δ 8.06 – 7.96 (m, 4H, Ar*H*), 7.84 – 7.70 (m, 4H, Ar*H*), 7.67 – 7.50 (m, 5H, Ar*H*), 7.50 – 7.35 (m, 7H, Ar*H*), 7.25 – 7.17 (m, 2H, Ar*H*), 7.00 – 6.64 (m, 2H, Ar*H*), 5.97 (d, *J* = 3.2 Hz, 1H, H-4), 5.70 (t, *J* = 9.9 Hz, 1H, H-2), 5.57 (dd, *J* = 9.9, 3.2 Hz, 1H, H-3), 4.90 (d, *J* = 9.8 Hz, 1H, H-1), 4.64 (dd, *J* = 10.3, 5.7 Hz, 1H, H-6a), 4.43 – 4.32 (m, 2H, H-5, H-6b), 3.81 (s, 3H, OCH_3); $^{13}C\{^1H\}$ NMR (101 MHz, $CDCl_3$) δ 166.1 (C=O, Bz), 165.3 (C=O, Bz), 165.2 (C=O, Bz), 160.5 (C=O, Bz), 137.4 (Ar-C), 133.7 (Ar-C), 133.64 (Ar-C), 133.55 (Ar-C), 133.34 (Ar-C), 133.27 (Ar-C), 133.25 (Ar-C), 130.2 (Ar-C), 130.0 (Ar-C), 129.84 (Ar-C), 129.78 (Ar-C), 129.5 (Ar-C), 129.4 (Ar-C), 128.9 (Ar-C), 128.8 (Ar-C), 128.51 (Ar-C), 128.45 (Ar-C), 128.3 (Ar-C), 120.4 (Ar-C), 114.3 (Ar-C), 85.4 (C1), 74.9 (C5), 73.1 (C3), 68.2 (C4)

67.8 (C2), 62.4 (C6), 55.3 (OCH_3); HRMS *m/z* (ES^+) Found: $(M+NH_4)^+$ 736.2227, $C_{41}H_{38}O_{10}NS$ requires M^+ 736.2211. Data matched those reported previously.[30]

p-(Methoxy)-phenyl 3,4,6-tri-*O*-benzoyl-1-thio-β-D-galactopyranoside 64 & *p*-(methoxy)-phenyl 2,3,6-tri-*O*-benzoyl-1-thio-β-D-galactopyranoside 65

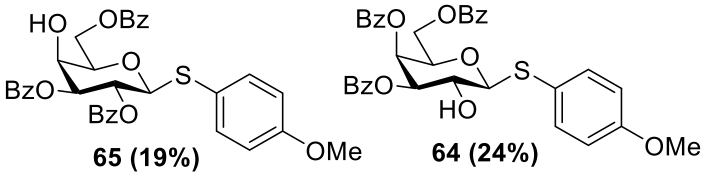

65 (19%) **64 (24%)**

64/65 were isolated as an inseparable mixture appearing as a white foam (135 mg, 0.220 mmol, **64/65**, 1:0.77, 43%); ratio determined from ^{1}H NMR integration values. The following were observed for both regioisomers **64** and **65**: R_f = 0.38 (hexane/EtOAc, 7:3); ^{1}H NMR (400 MHz, $CDCl_3$) δ 8.07 – 7.95 (m, 6H, Ar*H*), 7.84 – 7.75 (m, 3.5H, Ar*H*), 7.66 – 7.56 (m, 4.5H, Ar*H*), 7.55 – 7.33 (m, 15H , Ar*H*), 7.33 – 7.26 (m, 1.5H, Ar*H*), 6.92 – 6.82 (m, 2H, Ar*H*), 6.72 – 6.67 (m, 1.5H, Ar*H*); $^{13}C\{^1H\}$ NMR (101 MHz, $CDCl_3$) δ 166.4 (C=O, Bz), 166.1 (C=O, Bz), 166.0 (C=O, Bz), 165.8 (C=O, Bz), 165.4 (C=O, Bz), 165.3 (C=O, Bz), 160.5 (Ar-C), 160.1 (Ar-C), 137.0 (Ar-C), 135.8 (Ar-C), 133.7 (Ar-C), 133.54 (Ar-C), 133.52 (Ar-C), 133.33 (Ar-C), 133.28 (Ar-C), 133.25 (Ar-C), 130.2 (Ar-C), 130.0 (Ar-C), 129.9 (Ar-C), 129.8 (Ar-C), 129.7 (Ar-C), 129.5 (Ar-C), 129.3 (Ar-C), 129.1 (Ar-C), 129.0 (Ar-C), 128.5 (Ar-C), 128.44 (Ar-C), 128.42 (Ar-C), 128.28 (Ar-C), 122.34 (Ar-C), 120.0 (Ar-C), 114.6 (Ar-C), 114.4 (Ar-C); HRMS *m/z* (ES^+) Found: $(M+Na)^+$ 637.1512, $C_{34}H_{30}O_9NaS$ requires M^+ 637.1503; **65:** ^{1}H NMR (400 MHz, $CDCl_3$) δ 5.74 (t, *J* = 10.0 Hz, 1H, H-2), 5.36 (dd, *J* = 9.9, 3.1 Hz, 1H, H-3), 4.83 (d, *J* = 10.0 Hz, 1H, H-1), 4.67 – 4.54 (m, 2H, H-6a, H-6b), 4.42 – 4.32 (m, 1H, H-4), 4.28 (t, *J* = 6.6 Hz, 1H, H-5), 3.73 (s, 3H, OCH_3); $^{13}C\{^1H\}$ NMR (101 MHz, $CDCl_3$) δ 87.3 (C1), 76.2 (C5), 75.3 (C3), 68.0 (C2), 67.7 (C4), 63.3 (C6), 55.3 (OCH_3); **64:** ^{1}H NMR (400 MHz, $CDCl_3$) δ 5.88 (dd, *J* = 3.2, 0.7 Hz, 1H, H-4), 5.41 (dd, *J* = 9.7, 3.2 Hz, 1H, H-3), 4.64 (d, *J* = 9.6 Hz, 1H, H-1), 4.66 – 4.61 (m, 1H, H-6a), 4.39 – 4.32 (m, 1H, H-6b), 4.28 (t, *J* = 6.6 Hz, 1H, H-5), 3.98 (t, *J* = 9.6 Hz, 1H, H-2), 3.83 (s, 3H, OCH_3); $^{13}C\{^1H\}$ NMR (101 MHz, $CDCl_3$) δ 88.0 (C1), 75.0 (C5), 74.5 (C3), 68.6 (C4), 67.1 (C2), 62.4 (C6), 55.4 (OCH_3).

p-(Methoxy)-phenyl 3,6-di-*O*-benzoyl-1-thio-β-D-galactopyranoside 66

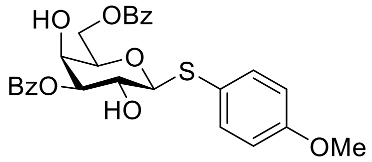

66 (56.0 mg, 0.110 mmol, 21%) as a colourless oil. R_f = 0.58 (hexane/EtOAc, 7:3); $[\alpha]_D^{22}$ = -5.4 (*c* = 1.0, $CHCl_3$); ^{1}H NMR (400 MHz, $CDCl_3$) δ 8.13 – 8.00 (m, 4H, Ar*H*), 7.63 – 7.42 (m, 8H, Ar*H*), 6.80 – 6.74 (m, 2H, Ar*H*), 5.16 (dd, *J* = 9.6, 3.2 Hz, 1H, H-3), 4.66 – 4.58 (m, 2H, H-6a, H-6b), 4.56 (d, *J* = 9.7 Hz, 1H, H-1), 4.25 (d, *J* = 3.1 Hz, 1H, H-4), 4.04 – 3.95 (m, 2H. H-2, H-5), 3.77 (s, 3H, OCH_3); $^{13}C\{^1H\}$ NMR (101 MHz, $CDCl_3$) δ 166.4 (C=O, Bz), 166.1 (C=O, Bz), 160.3 (Ar-C), 135.7 (Ar-C), 133.5 (Ar-C), 133.3 (Ar-C), 129.9 (Ar-C), 129.8 (Ar-

C), 129.6 (Ar-C), 129.4 (Ar-C), 128.51 (Ar-C), 128.48 (Ar-C), 121.6 (Ar-C), 114.6 (Ar-C), 89.7 (C1), 76.6 (C3), 76.2 (C5), 67.7 (C4), 67.4 (C2), 63.1 (C6), 55.3 (OCH_3); HRMS *m/z* (ES^-) Found: $(M-H)^-$ 509.1280, $C_{27}H_{25}O_8S$ requires M^- 509.1276.

Benzoylation of *p*-(nitro)-phenyl 1-thio-β-D-galactopyranoside 67

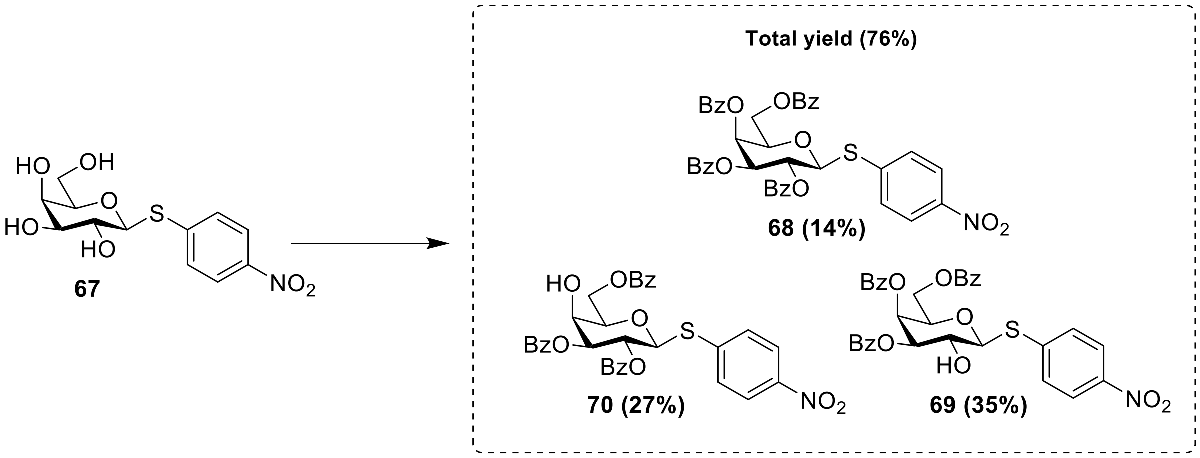

Following the general acylation procedure, *p*-(nitro)-phenyl 1-thio-β-D-galactopyranoside **67** (127 mg, 0.400 mmol, 1.0 equiv.) and BzCl (144 μL, 1.24 mmol, 3.1 equiv.) were reacted. Purification by column chromatography (hexane/EtOAc, 0-40%) yielded the below compounds in a total yield of 76%.

p-(Nitro)-phenyl 2,3,4,6-tetra-*O*-benzoy-1-thio-β-D-galactopyranoside 68

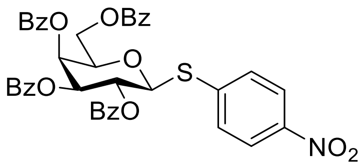

68 (41.1 mg, 56.0 μmol, 14%) as a colourless oil. R_f = 0.44 (hexane/EtOAc, 7:3); ^1H NMR (400 MHz, $CDCl_3$) δ 8.06 – 7.91 (m, 8H, Ar*H*), 7.80 – 7.71 (m, 2H, Ar*H*), 7.71 – 7.58 (m, 5H, Ar*H*), 7.58 – 7.51 (m, 1H, Ar*H*), 7.51 – 7.37 (m, 9H, Ar*H*), 7.25 – 7.20 (m, 2H, Ar*H*), 6.05 (dd, *J* = 3.3, 0.7 Hz, 1H, H-4), 5.83 (t, *J* = 9.9 Hz, 1H, H-2), 5.68 (dd, *J* = 9.9, 3.3 Hz, 1H, H-3), 5.19 (d, *J* = 9.9 Hz, 1H, H-1), 4.66 (dd, *J* = 11.5, 7.4 Hz, 1H, H-6a), 4.59 – 4.43 (m, 2H, H-5, H-6b); $^{13}C\{^1H\}$ NMR (101 MHz, $CDCl_3$) δ 171.0 (C=O, Bz), 166.0 (C=O, Bz), 165.4 (C=O, Bz), 165.2 (C=O, Bz), 147.1 (Ar-C), 141.5 (Ar-C), 133.9 (Ar-C), 133.8 (Ar-C), 133.66 (Ar-C), 133.65 (Ar-C), 133.5 (Ar-C), 131.7 (Ar-C), 130.2 (Ar-C), 129.92 (Ar-C), 129.85 (Ar-C), 129.8 (Ar-C), 129.3 (Ar-C), 129.2 (Ar-C), 128.83 (Ar-C), 128.76 (Ar-C), 128.7 (Ar-C), 128.6 (Ar-C), 128.54 (Ar-C), 128.51 (Ar-C), 128.4 (Ar-C), 123.8 (Ar-C), 84.8 (C1), 75.0 (C5), 72.6 (C3), 68.3 (C4), 67.7 (C2), 62.7 (C6); HRMS *m/z* (ES^+) Found: $(M+NH_4)^+$ 751.1965, $C_{40}H_{35}O_{11}N_2S$ requires M^+ 751.1956. Data matched those previously reported.[31]

p-(Nitro)-phenyl 2,3,6-tri-*O*-benzoyl-1-thio-β-D-galactopyranoside 70 & *p*-(nitro)-phenyl 3,4,6-tri-*O*-benzoyl-1-thio-β-D-galactopyranoside 69

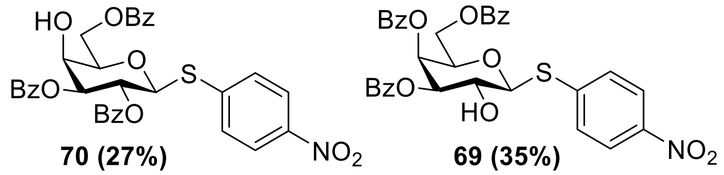

70 (27%) **69 (35%)**

69/70 were isolated as an inseparable mixture appearing as a white foam (156 mg, 0.248 mmol, **69/70**, 1.0:0.77 62%); ratio determined from ^{1}H NMR integration values. The following were observed for both regio-isomers **69** and **70**: R_f = 0.27 (hexane/EtOAc, 7:3); ^{1}H NMR (400 MHz, $CDCl_3$) δ 8.12 – 8.04 (m, 3H, Ar*H*), 8.04 – 7.97 (m, 6H, Ar*H*), 7.96 – 7.90 (m, 4.5H, Ar*H*), 7.86 – 7.78 (m, 2H, Ar*H*), 7.74 – 7.69 (m, 2H, Ar*H*), 7.68 – 7.57 (m, 3.5H, Ar*H*), 7.56 – 7.43 (m, 10H, Ar*H*), 7.41 – 7.27 (m, 4.5H, Ar*H*); ^{13}C{^{1}H} NMR (101 MHz, $CDCl_3$) δ 133.9 (Ar-C), 133.8 (Ar-C), 133.7 (Ar-C), 133.63 (Ar-C), 133.55 (Ar-C), 131.1 (Ar-C), 130.2 (Ar-C), 130.1 (Ar-C), 129.9 (Ar-C), 129.8 (Ar-C), 129.7 (Ar-C), 129.4 (Ar-C), 129.3 (Ar-C), 129.0 (Ar-C), 128.94 (Ar-C), 128.90 (Ar-C), 128.71 (Ar-C), 128.69 (Ar-C), 128.64 (Ar-C), 128.58 (Ar-C), 128.57 (Ar-C), 128.5 (Ar-C), 128.4 (Ar-C), 123.92 (Ar-C), 123.85 (Ar-C); HRMS *m/z* (ES^{+}) Found: $(M+NH_4)^+$ 647.1701, $C_{33}H_{31}O_{10}N_2S$ requires M^+ 647.1694; **69:** ^{1}H NMR (400 MHz, $CDCl_3$) δ 5.97 (d, *J* = 2.7 Hz, 1H, H-4), 5.45 (t, *J* = 3.4 Hz, 1H, H-3), 5.00 (d, *J* = 9.7 Hz, 1H, H-1), 4.62 (dd, *J* = 11.6, 7.6 Hz, 1H, H-6a), 4.48 (dd, *J* = 11.7, 4.5 Hz, 1H, H-6b), 4.44 – 4.38 (m, 1H, H-5), 4.20 (t, *J* = 9.7 Hz, 1H, H-2); ^{13}C{^{1}H} NMR (101 MHz, $CDCl_3$) δ 166.2 (C=O, Bz), 166.0 (C=O, Bz), 165.4 (C=O, Bz), 87.1 (C1), 75.5 (C5), 74.7 (C3), 68.5 (C4), 68.0 (C2), 62.7 (C6); **70:** ^{1}H NMR (400 MHz, $CDCl_3$) δ 5.91 (t, *J* = 9.9 Hz, 1H, H-2), 5.48 (t, *J* = 3.5 Hz, 1H, H-3), 5.14 (d, *J* = 10.0 Hz, 1H, H-1), 4.74 (dd, *J* = 11.8, 4.8 Hz, 1H, H-6a), 4.68 (dd, *J* = 11.8, 7.5 Hz, 1H, H-6b), 4.49 – 4.46 (m, 1H, H-4), 4.27 – 4.23 (m, 1H, H-5); ^{13}C{^{1}H} NMR (101 MHz, $CDCl_3$) δ 84.9 (C1), 76.6 (C5), 74.9 (C3), 67.6 (C4), 67.5 (C2), 63.5 (C6). Data matched those previously reported.[31]

4. References

(1) Keenan, T.; Hatton, N. E.; Porter, J.; Vendeville, J. B.; Wheatley, D. E.; Ghirardello, M.; Wahart, A. J. C.; Ahmadipour, S.; Walton, J.; Galan, M. C.; Linclau, B.; Miller, G. J.; Fascione, M. A. Reverse Thiophosphorylase Activity of a Glycoside Phosphorylase in the Synthesis of an Unnatural Manβ1,4GlcNAc Library. *Chem. Sci.* **2023**, *14*, 11638–11646. https://doi.org/10.1039/d3sc04169g.

(2) Venkatesh, R.; Tiwari, V.; Kandasamy, J. Copper(I)-Catalyzed Sandmeyer-Type *S*-Arylation of 1-Thiosugars with Aryldiazonium Salts under Mild Conditions. *J. Org. Chem.* **2022**, *87*, 11414–11432. https://doi.org/10.1021/acs.joc.2c00930.

(3) Wawryszyn, M.; Sauter, P. F.; Nieger, M.; Koos, M. R. M.; Koehler, C.; Luy, B.; Lemke, E. A.; Bräse, S. Synthesis of Azido-Glycans for Chemical Glycomodification of Proteins. *Eur. J. Org. Chem.* **2018**, 4296–4305. https://doi.org/10.1002/ejoc.201800602.

(4) Calosso, M.; Tambutet, G.; Charpentier, D.; St-Pierre, G.; Vaillancourt, M.; Bencheqroun, M.; Gratton, J. P.; Prévost, M.; Guindon, Y. Acyclic Tethers Mimicking

Subunits of Polysaccharide Ligands: Selectin Antagonists. *ACS Med. Chem. Lett.* **2014**, *5*, 1054–1059. https://doi.org/10.1021/ml500266x.

(5) Grube, M.; Lee, B. Y.; Garg, M.; Michel, D.; Vilotijević, I.; Malik, A.; Seeberger, P. H.; Varón Silva, D. Synthesis of Galactosylated Glycosylphosphatidylinositol Derivatives from Trypanosoma Brucei. *Chem. Eur. J.* **2018**, *24*, 3271–3282. https://doi.org/10.1002/chem.201705511.

(6) Verdelet, T.; Benmahdjoub, S.; Benmerad, B.; Alami, M.; Messaoudi, S. Copper-Catalyzed Anomeric *O*-Arylation of Carbohydrate Derivatives at Room Temperature. *J. Org. Chem.* **2019**, *84*, 9226–9238. https://doi.org/10.1021/acs.joc.9b01218.

(7) Muramatsu, W. Catalytic and Regioselective Oxidation of Carbohydrates To Synthesize Keto-Sugars under Mild Conditions. *Org. Lett.* **2014**, *16*, 4846-4849. https://doi.org/10.1021/ol502344h

(8) Hartog, A. F.; Wever, R. Substrate Engineering and Its Synthetic Utility in the Sulfation of Primary Aliphatic Alcohol Groups by a Bacterial Arylsulfotransferase. *Adv. Synth. Catal.* **2015**, *357*, 2629–2632. https://doi.org/10.1002/adsc.201500482.

(9) Halcomb, R. L.; Huang, H.; Wong, C.-H. Solution-and Solid-Phase Synthesis of Inhibitors of H. Pylori Attachment and E-Selectin-Mediated Leukocyte Adhesion. *J. Am. Chem. Soc.* **1994**, *116*, 11315-11322. https://doi.org/10.1021/ja00104a011

(10) Cha, J. M.; Kim, D. H.; Subedi, L.; Khan, Z.; Choi, S. U.; Kim, S. Y.; Kim, C. S. Chemical Constituents of Chaenomeles Sinensis Twigs and Their Biological Activity. *Beilstein J. Org. Chem.* **2020**, *16*, 3078–3085. https://doi.org/10.3762/BJOC.16.257.

(11) Nilsson, U. J.; J-L Fournier, E.; Hindsgaul, O. Solid-Phase Extraction on C18 Silica as a Purification Strategy in the Solution Synthesis of a 1-Thio-D-Galactopyranoside Library. *Bioorg. Med. Chem.* **1998**, *6*, 1563-1575. https://doi.org/10.1016/S0968-0896(98)00087-X

(12) Mamidyala, S. K.; Finn, M. G. Glycosylation Using Unprotected Alkynyl Donors. *J. Org. Chem.* **2009**, *74*, 8417–8420. https://doi.org/10.1021/jo901857x.

(13) Zou, L. J.; Pan, Q.; Li, C. Y.; Zhang, Z. T.; Zhang, X. W.; Hu, X. G. Cyanide-Free Synthesis of Glycosyl Carboxylic Acids and Application for the Synthesis of Scleropentaside A. *Org. Lett.* **2020**, *22*, 8302–8306. https://doi.org/10.1021/acs.orglett.0c02949.

(14) Markovic, D.; Tchawou, W. A.; Novosjolova, I.; Laclef, S.; Stepanovs, D.; Turks, M.; Vogel, P. Synthesis and Applications of Silyl 2-Methylprop-2-Ene-1-Sulfinates in Preparative Silylation and GC-Derivatization Reactions of Polyols and Carbohydrates. *Chem. Eur. J.* **2016**, *22*, 4196–4205. https://doi.org/10.1002/chem.201504380.

(15) Kumar, A.; Gannedi, V.; Rather, S. A.; Vishwakarma, R. A.; Ahmed, Q. N. Introducing Oxo-Phenylacetyl (OPAc) as a Protecting Group for Carbohydrates. *J. Org. Chem.* **2019**, *84*, 4131–4138. https://doi.org/10.1021/acs.joc.9b00126.

(16) Mamidyala, S. K.; Dutta, S.; Chrunyk, B. A.; Préville, C.; Wang, H.; Withka, J. M.; McColl, A.; Subashi, T. A.; Hawrylik, S. J.; Griffor, M. C.; Kim, S.; Pfefferkorn, J. A.;

Price, D. A.; Menhaji-Klotz, E.; Mascitti, V.; Finn, M. G. Glycomimetic Ligands for the Human Asialoglycoprotein Receptor. *J. Am. Chem. Soc.* **2012**, *134*, 1978–1981. https://doi.org/10.1021/ja2104679.

(17) Wei, J.; Lv, X.; Lü, Y.; Yang, G.; Fu, L.; Yang, L.; Wang, J.; Gao, J.; Cheng, S.; Duan, Q.; Jin, C.; Li, X. Glycosynthase with Broad Substrate Specificity-an Efficient Biocatalyst for the Construction of Oligosaccharide Library. *Eur. J. Org. Chem.* **2013**, *12*, 2414–2419. https://doi.org/10.1002/ejoc.201201507.

(18) Kooner, A. S.; Diaz, S.; Yu, H.; Santra, A.; Varki, A.; Chen, X. Chemoenzymatic Synthesis of Sialosides Containing 7- N- or 7,9-Di- N-Acetyl Sialic Acid as Stable *O*-Acetyl Analogues for Probing Sialic Acid-Binding Proteins. *J. Org. Chem.* **2021**, *86*, 14381–14397. https://doi.org/10.1021/acs.joc.1c01091.

(19) Zahorska, E.; Rosato, F.; Stober, K.; Kuhaudomlarp, S.; Meiers, J.; Hauck, D.; Reith, D.; Gillon, E.; Rox, K.; Imberty, A.; Römer, W.; Titz, A. Neutralizing the Impact of the Virulence Factor LecA from Pseudomonas Aeruginosa on Human Cells with New Glycomimetic Inhibitors. *Angew. Chem. Int. Ed.* **2023**, *135*, e202215535. https://doi.org/10.1002/anie.202215535.

(20) Li, X.; Wu, J.; Tang, W. General Strategy for the Synthesis of Rare Sugars via Ru(II)-Catalyzed and Boron-Mediated Selective Epimerization of 1,2- Trans-Diols to 1,2-Cis-Diols. *J. Am. Chem. Soc.* **2022**, *144*, 3727–3736. https://doi.org/10.1021/jacs.1c13399.

(21) Moule, C. J.; Clements, P. R.; Hopwood, J. J.; Crisp, G. T. Affinity Labelling at the 4-Hydroxyl Group of *N*- Acetylglucosamine and N-Acetylgalactosamine. *Synth. Commun.* **2000**, *30*, 1489–1501. https://doi.org/10.1080/00397910008087177.

(22) Schocker, N. S.; Portillo, S.; Brito, C. R. N.; Marques, A. F.; Almeida, I. C.; Michael, K. Synthesis of Galα(1,3)Galβ(1,4)GlcNAcα-Galβ(1,4)GlcNAcα- and GlcNAc-Containing Neoglycoproteins and Their Immunological Evaluation in the Context of Chagas Disease. *Glycobiol.* **2015**, *26*, 39–50. https://doi.org/10.1093/glycob/cwv081.

(23) Gavel, M.; Courant, T.; Joosten, A. Y. P.; Lecourt, T. Regio- A Nd Chemoselective Deprotection of Primary Acetates by Zirconium Hydrides. *Org. Lett.* **2019**, *21*, 1948–1952. https://doi.org/10.1021/acs.orglett.8b03947.

(24) Shimada, N.; Nakamura, Y.; Ochiai, T.; Makino, K. Catalytic Activation of Cis-Vicinal Diols by Boronic Acids: Site-Selective Acylation of Carbohydrates. *Org. Lett.* **2019**, *21*, 3789–3794. https://doi.org/10.1021/acs.orglett.9b01231.

(25) Krumb, M.; Jäger, M.; Voss, A.; Immig, L.; Peters, K.; Kowalczyk, D.; Bufe, A.; Opatz, T.; Holst, O.; Vogel, C.; Peters, M. Total Synthesis of a Partial Structure from Arabinogalactan and Its Application for Allergy Prevention. *Chem. Eur. J.* **2021**, *27*, 928–933. https://doi.org/10.1002/chem.202002287.

(26) Sail, D.; Kováč, P. Benzoylated Ethyl 1-Thioglycosides: Direct Preparation from per-*O*-Benzoylated Sugars. *Carbohydr. Res.* **2012**, *357*, 47–52. https://doi.org/10.1016/j.carres.2012.05.012.

(27) Fyrner, T.; Svensson, S. C. T.; Konradsson, P. Synthesis of Tri-, Penta-, and Heptasaccharides, Functionalized with Orthogonally N-Protected Amino Residues at the Reducing and Non-Reducing Ends. *Tetrahedron* **2012**, *68*, 6712–6720. https://doi.org/10.1016/j.tet.2012.05.118.

(28) Patil, P. R.; Ravindranathan Kartha, K. P. Solvent-Free Mechanochemical Synthesis of Aryl Glycosides. *J. Carbohydr. Chem.* **2008**, *27*, 411–419. https://doi.org/10.1080/07328300802402259.

(29) Cardona, A.; Boutureira, O.; Castillón, S.; Díaz, Y.; Matheu, M. I. Metal-Free and VOC-Free: *O*-Glycosylation in Supercritical CO_2. *Green Chem.* **2017**, *19*, 2687–2694. https://doi.org/10.1039/c7gc00722a.

(30) Berteina, S.; Fischer, J. C.; Lubineau, A. Thioglycosides As Potential Glycosyl Donors In Electrochemical Glycosylation Reactions. Part 1: Their Preparation and Reactivity Toward Simple Alcohols. *J. Carbohydr. Chem.* **1995**, *14*, 1217–1236. https://doi.org/10.1080/07328309508005406.

(31) Apparu, M.; Blanc-Muesser, M.; Defaye, J.; Driquez, H. Stereoselective Syntheses of *O*- and *S*- Nitrophenyl Glycosides. Part III. Syntheses in the α-Galactopyranose and α-Maltose Series. *Can. J. Chem.* **1981**, *59*, 314-320. https://doi.org/10.1139/v81-049.
