# Supplementary material for: Benzoylation of Tetrols: A Comparison of Regioselectivity Patterns for O- and S-Glycosides of d-Galactose
Source: J Org Chem. 2024 Sep 12;89(19):14090–7. doi: 10.1021/acs.joc.4c01508 (PMC11460728; doi:10.1021/acs.joc.4c01508)

Benzoylation of tetrols: a comparison of regioselectivity patterns for *O*- and *S*-glycosides of D-galactose

Jack Porter, Jacob Roberts and Gavin J. Miller*

Centre for Glycoscience and School of Chemical and Physical Sciences, Keele University, Keele, Staffordshire, ST5 5BG, United Kingdom

* Email: g.j.miller@keele.ac.uk

Supporting Information 2

Spectra

Table of Contents

Synthesis of substrates....S6
Compound 26....S6
Compound 3....S8
Compound S3....S10
Compound S2....S12
Compound 31....S14
Compound 1....S16
Compound S5....S18
Compound S4....S20
Compound 36....S22
Compound 5....S24
Compound S6....S26
Compound 40....S28
Compound 7....S30
Compound 43....S32
Compound S7....S34
Compound 45....S39
Compound S8....S44
Compound 54....S49

Compound 49 .... S54
Compound 13 .... S56
Compound 11 .... S58
Compound 15 .... S60
Compound 17 .... S62
Compound S9 .... S64
Compound S10 .... S69
Compound S11 .... S71
Compound 57 .... S73
Compound 67 .... S78
Compound 62 .... S80
Acylation experiments S82
Compound 2 .... S82
Compound 4 .... S87
Compound 6 .... S91
Compound 8 .... S96
Compound 10 .... S101
Compound 12 .... S106
Compound 14 .... S111
Compound 16 .... S116

Compound 18 .... S121
Compound 20 .... S126
Compound 22 .... S131
Compound 23 .... S133
Compound 25 .... S136
Compound 27 .... S138
Compounds 28/29 .... S140
Compound 30 .... S145
Compound 32 .... S150
Compound 34 .... S152
Compound 33 .... S157
Compound 35 .... S162
Compound 37 .... S167
Compound 38/39 .... S171
Compound 41 .... S176
Compound 42 .... S178
Compound 44 .... S183
Compound 46 .... S188
Compounds 47/48 .... S193
Compound 50 .... S199

Compound 51/52 ....S203
Compound 53....................S208
Compound 55....................S213
Compound 56....................S218
Compound 58....................S224
Compound 59/60 ....S229
Compound 61....................S235
Compound 63....................S240
Compound 64/65 ....S244
Compound 66....................S249
Compound 68....................S254
Compounds 69/70....................S258

Synthesis of substrates

Compound 26

^{1}H NMR (400 MHz, MeOD) Phenyl 1-thio-β-D-galactopyranoside 26

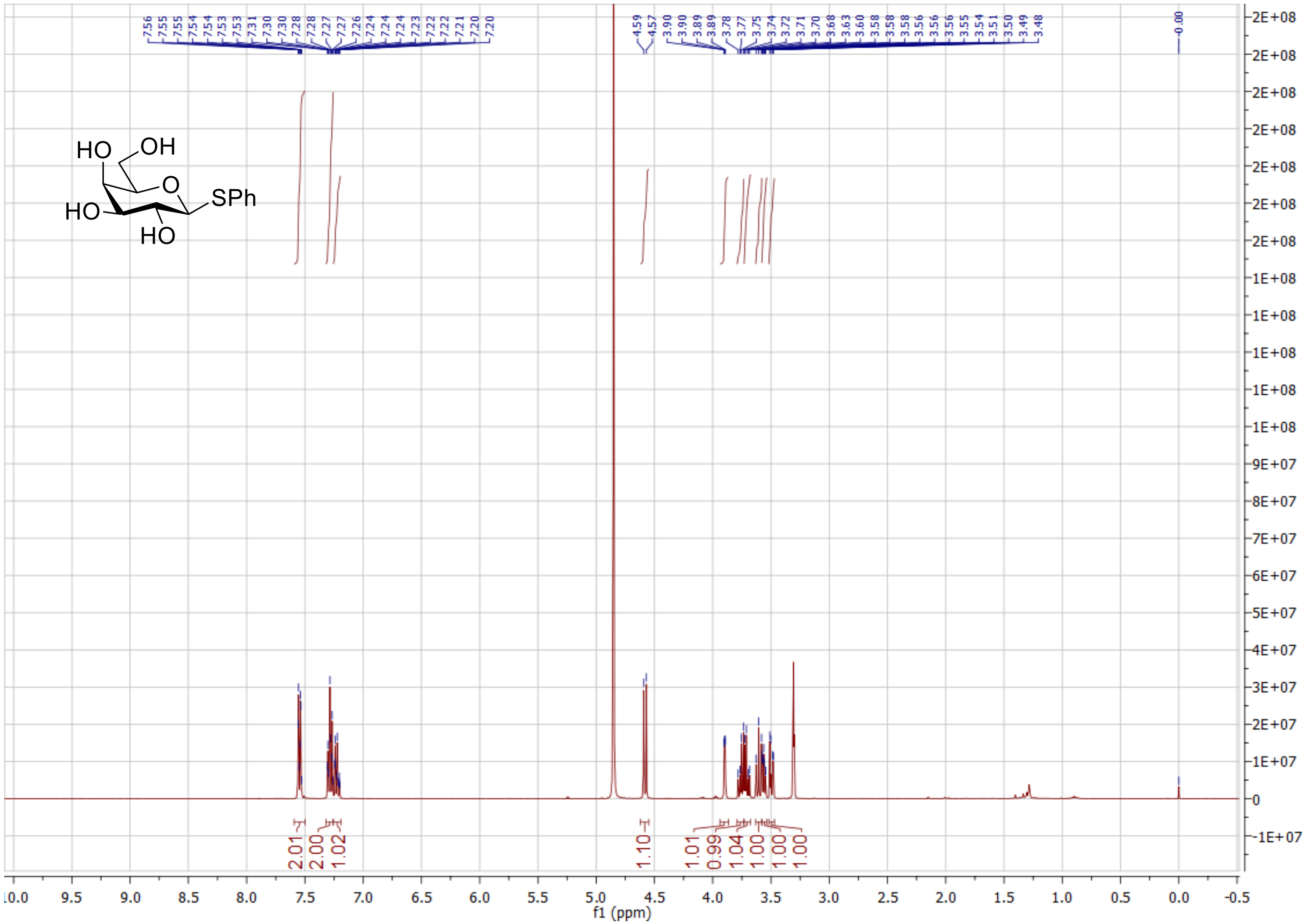

$^{13}C\{1H\}$ NMR (101 MHz, MeOD) Phenyl 1-thio-β-D-galactopyranoside 26

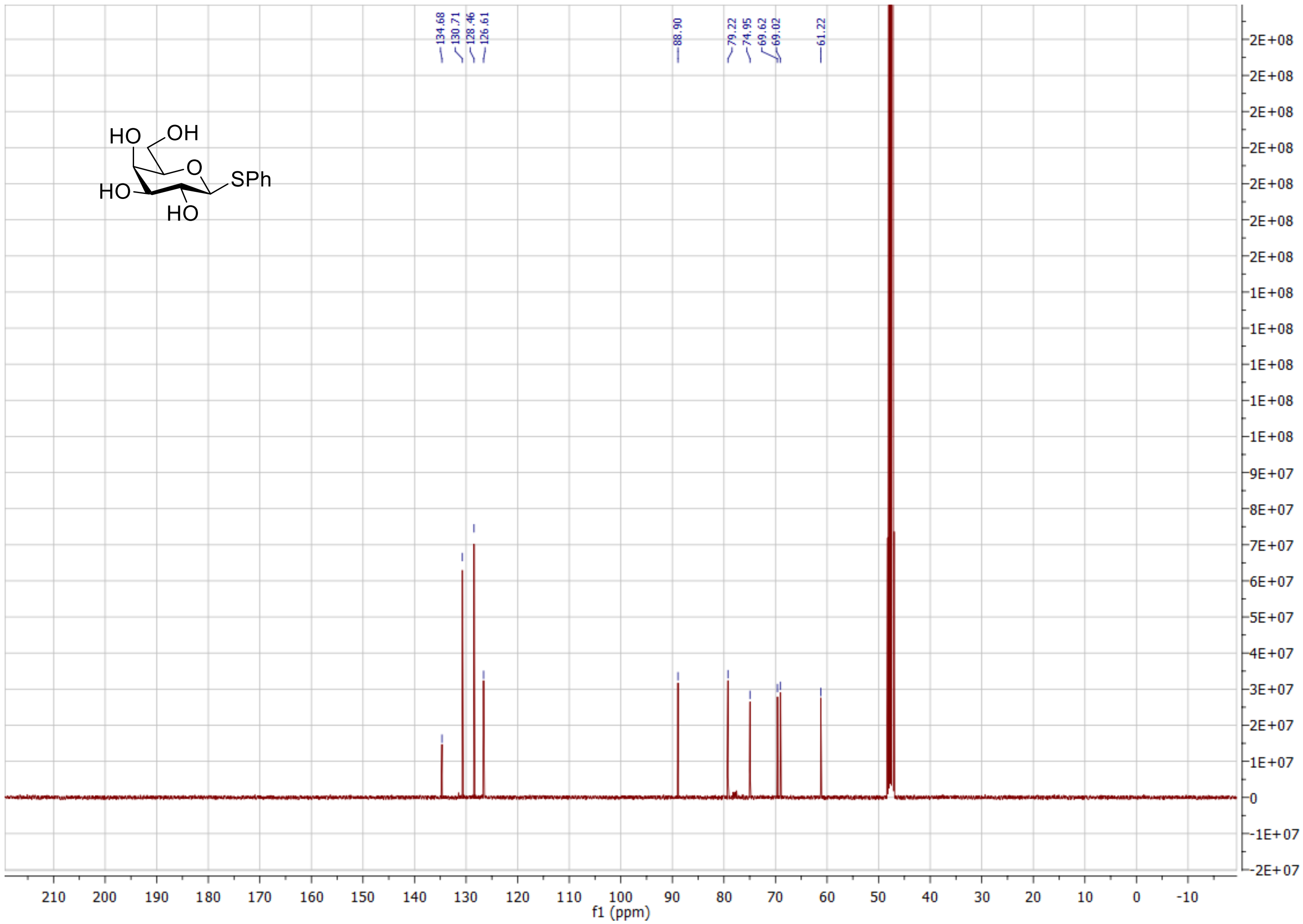

Compound 3

^{1}H NMR (400 MHz, MeOD) Phenyl 1-thio-α-D-galactopyranoside 3

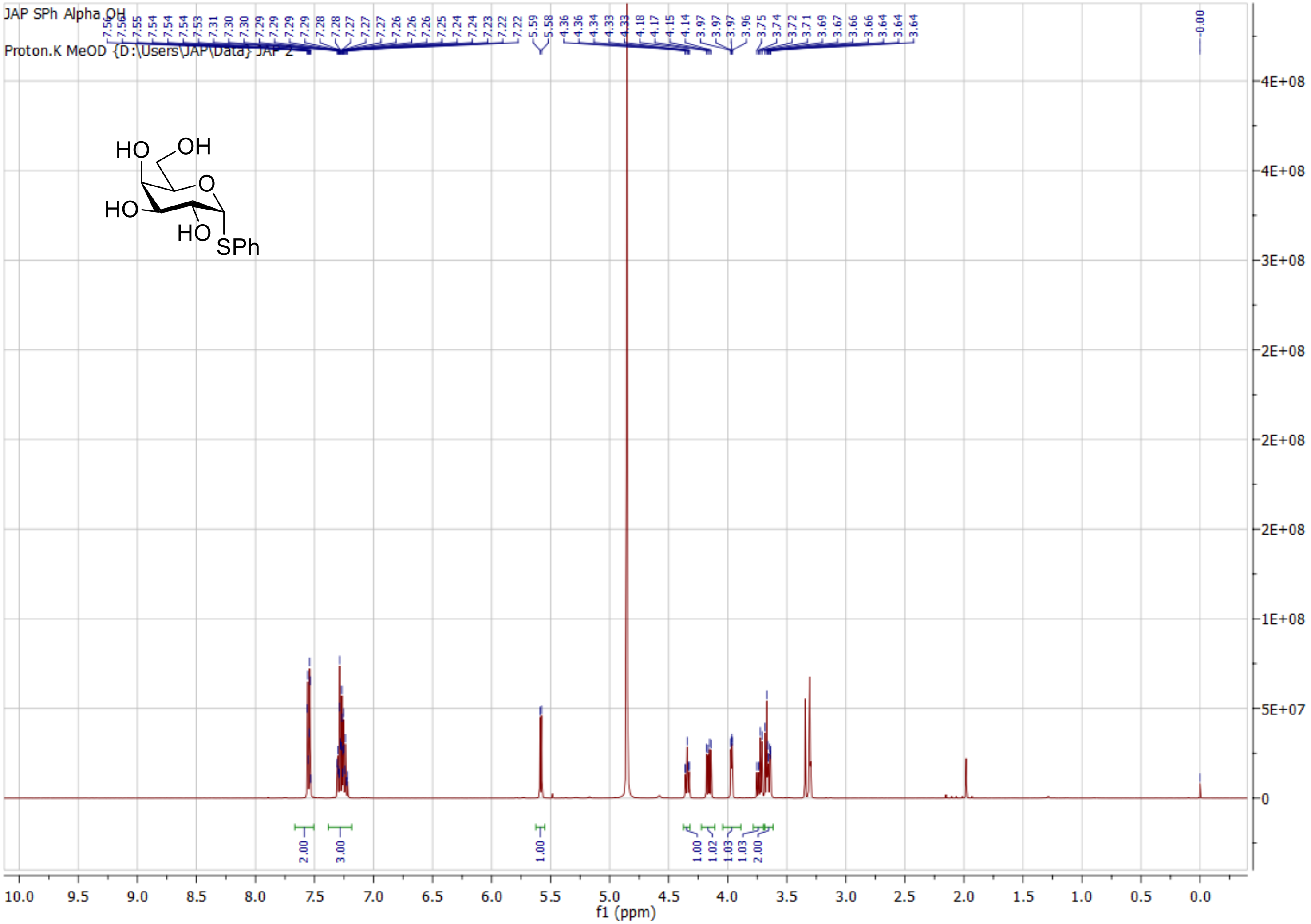

$^{13}C\{^1H\}$ NMR (101 MHz, MeOD) Phenyl 1-thio-β-D-galactopyranoside 3

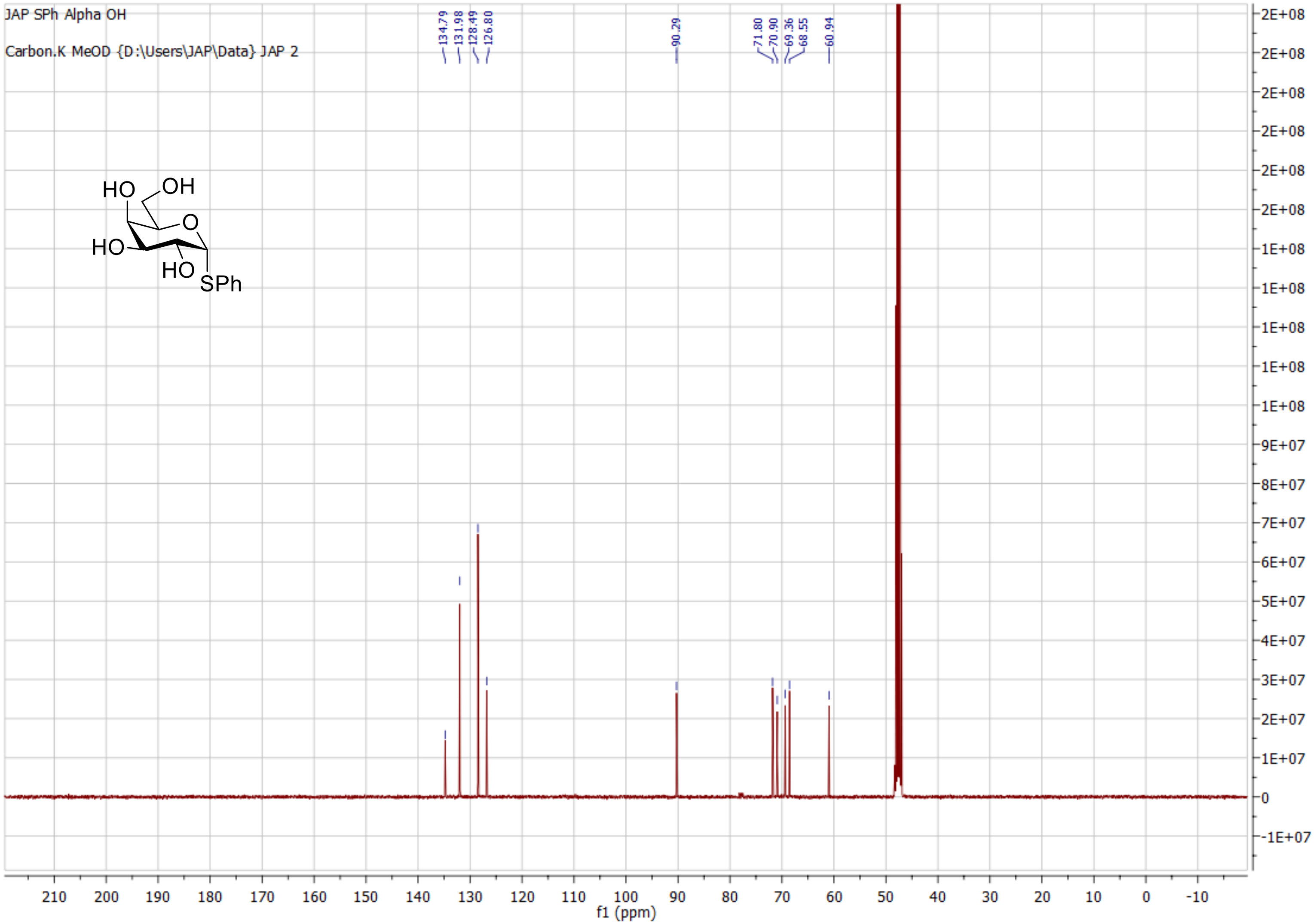

Compound S3

^{1}H NMR (400 MHz, CDCl$_3$) Ethyl 2,3,4,6-tetra-*O*-acteyl-1-thio-β-D-galactopyranoside S3

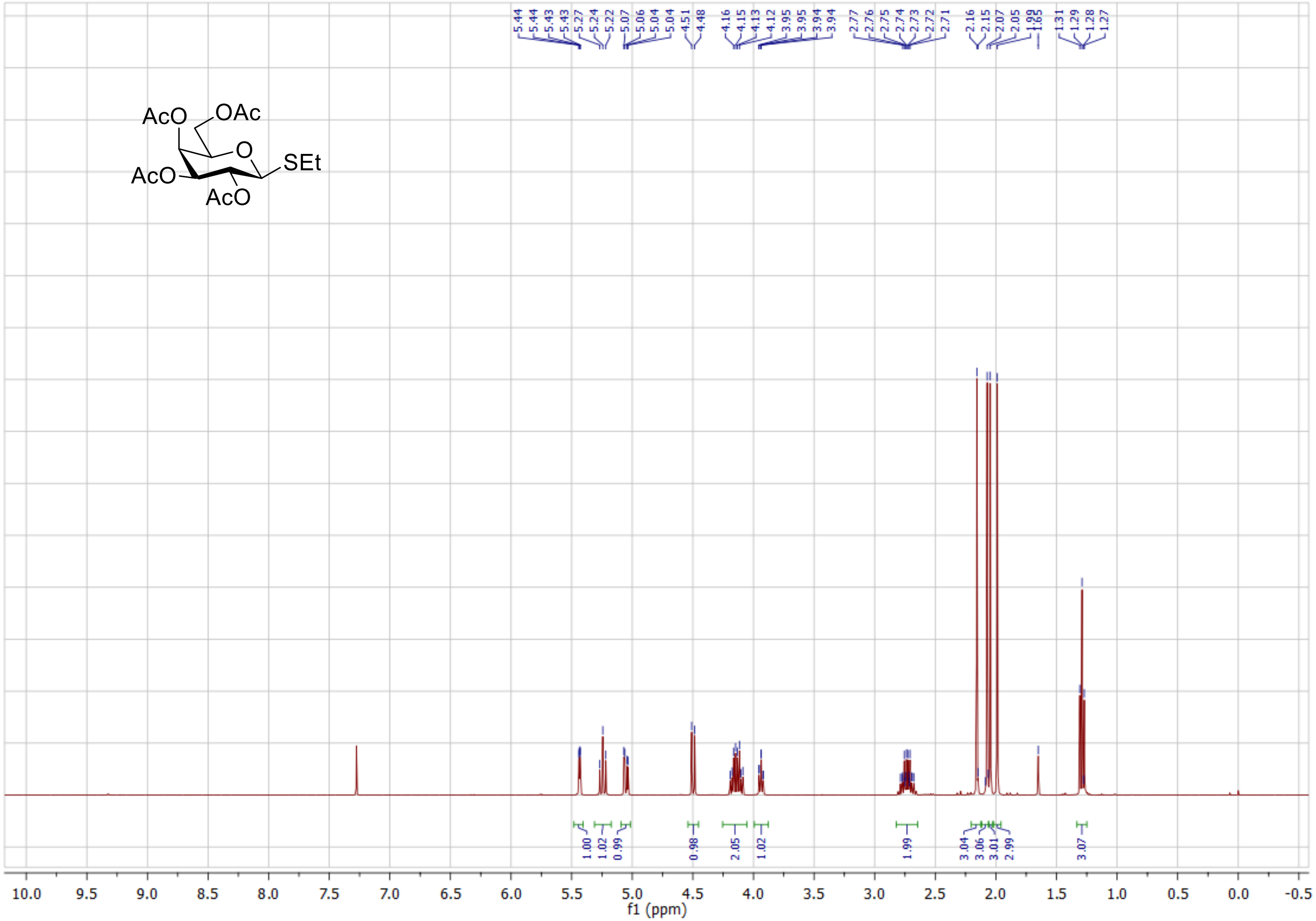

^{13}C{1H} NMR (101 MHz, $CDCl_3$) Ethyl 2,3,4,6-tetra-*O*-acteyl-1-thio-β-D-galactopyranoside S3

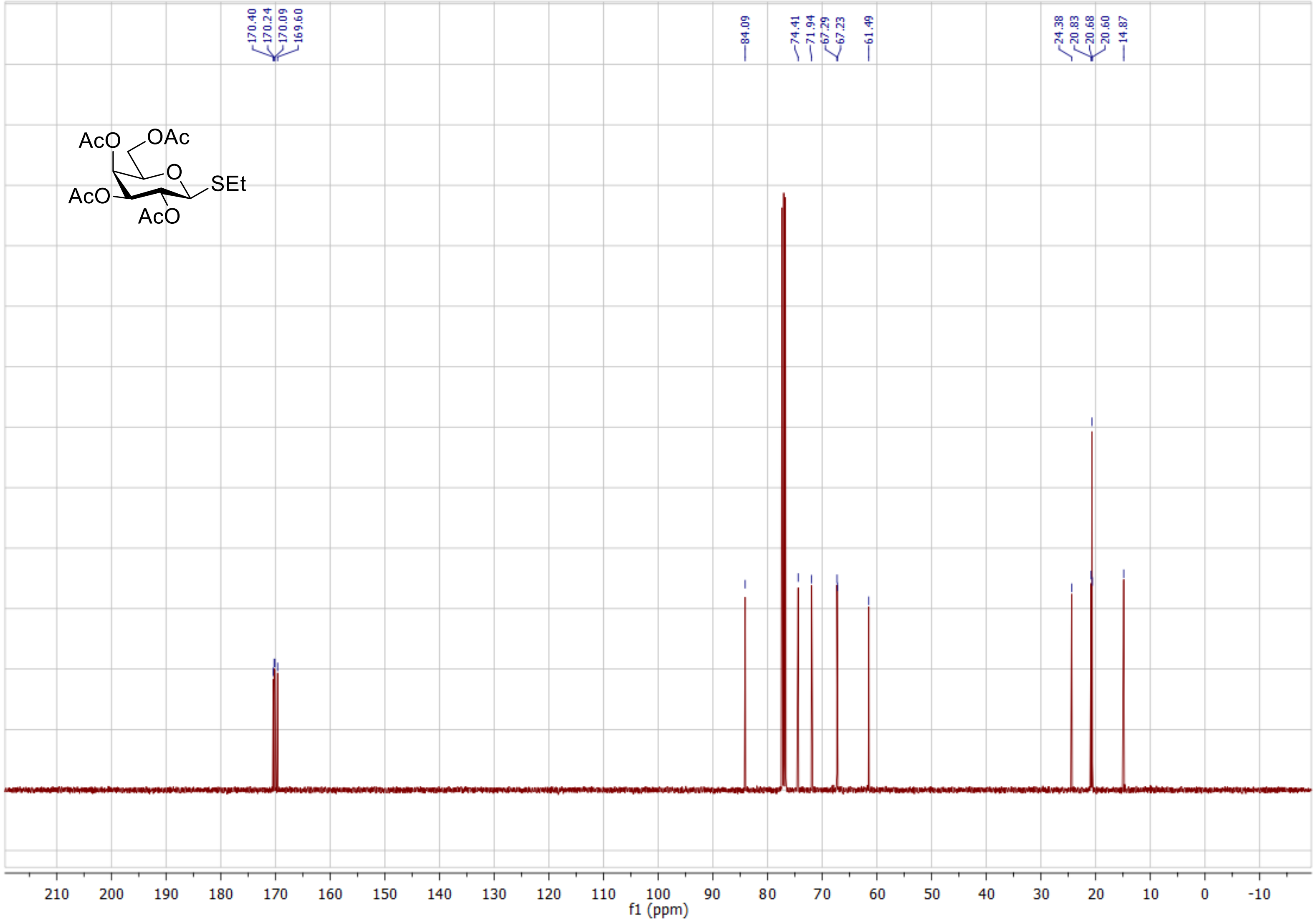

Compound S2

^{1}H NMR (400 MHz, $CDCl_3$) Ethyl 2,3,4,6-tetra-*O*-acteyl-1-thio-α-D-galactopyranoside S2

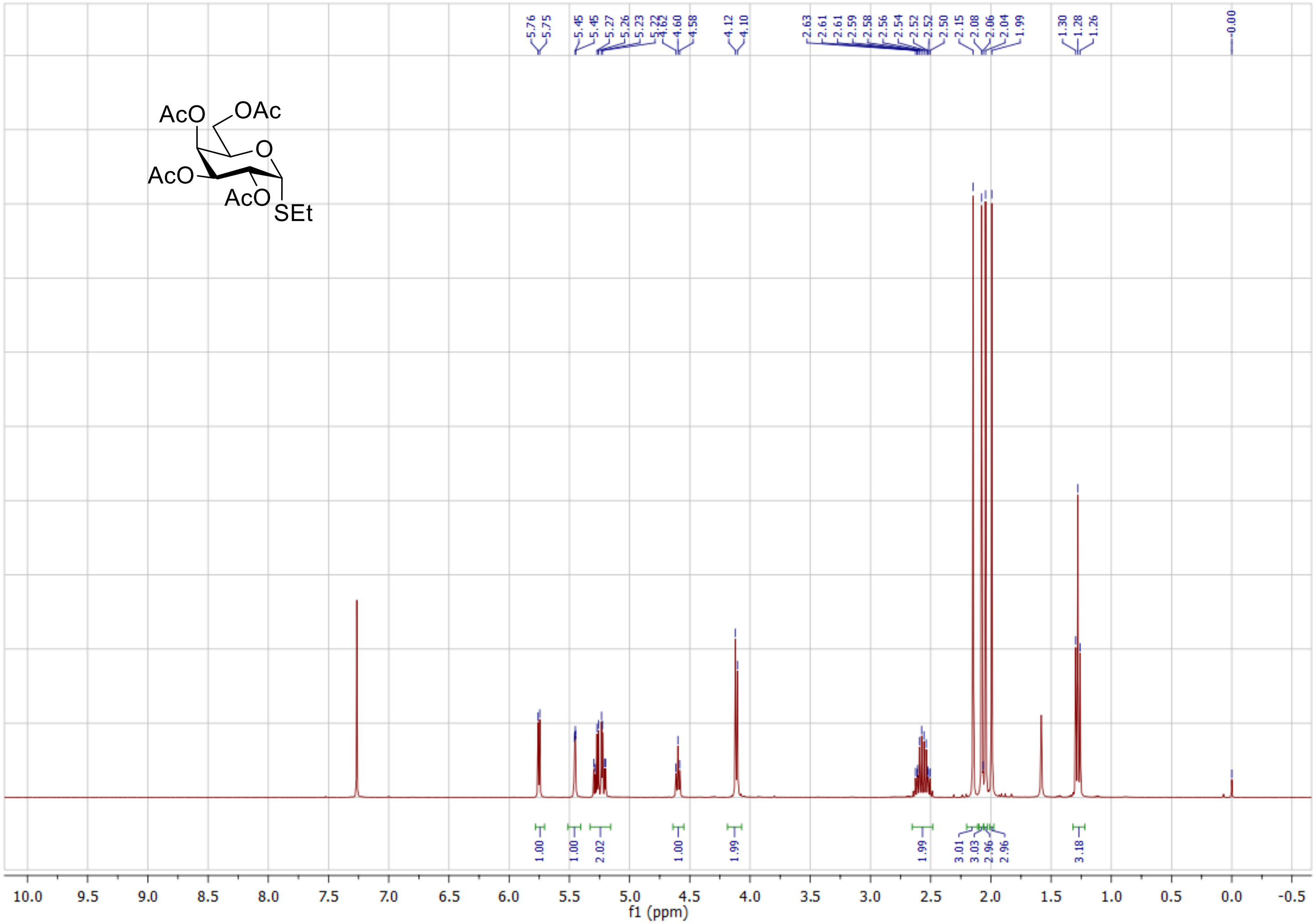

$^{13}C\{1H\}$ NMR (101 MHz, $CDCl_3$) Ethyl 2,3,4,6-tetra-*O*-acteyl-1-thio-α-D-galactopyranoside S2

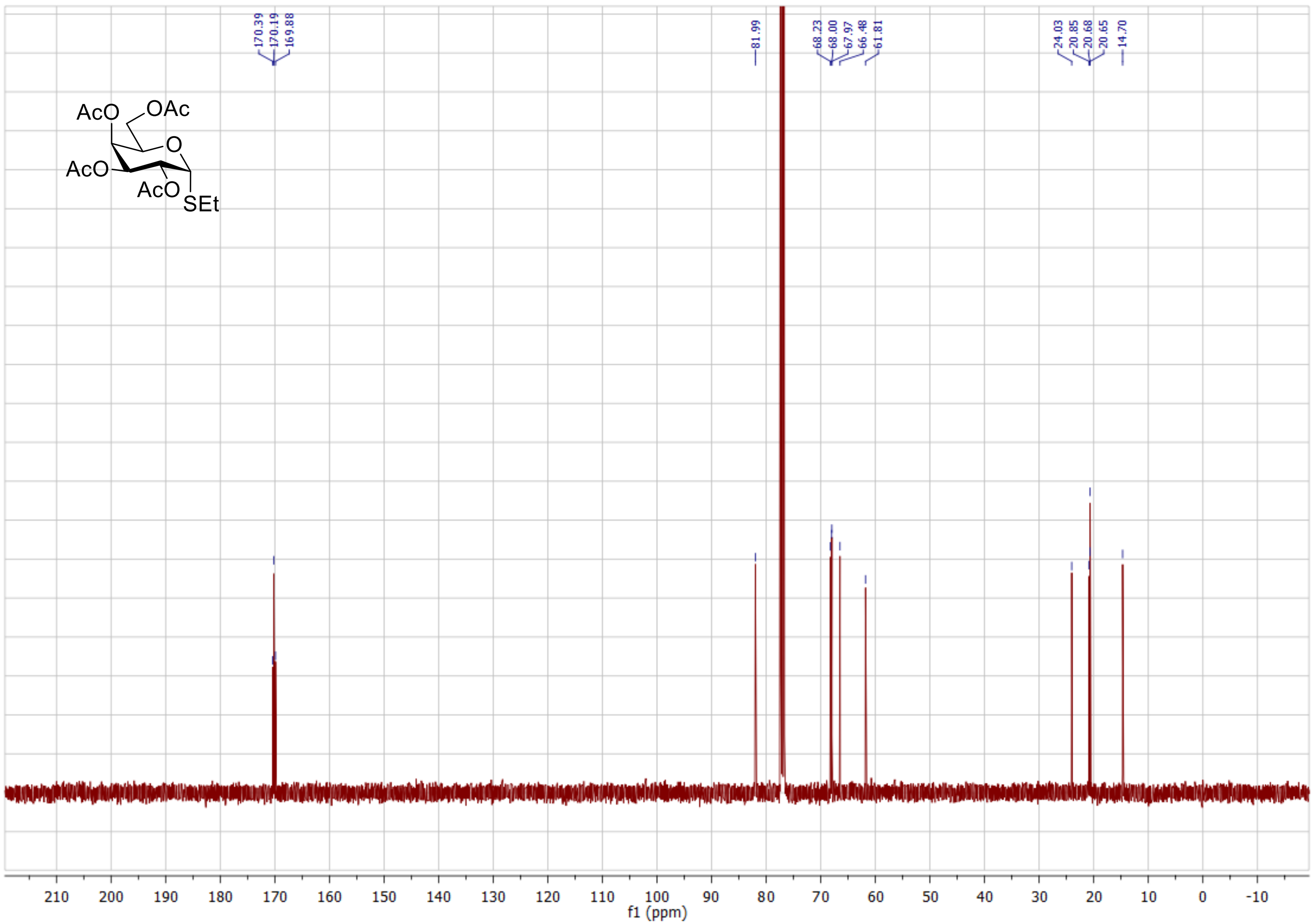

Compound 31

^{1}H NMR (400 MHz, MeOD) Ethyl 1-thio-β-D-galactopyranoside 31

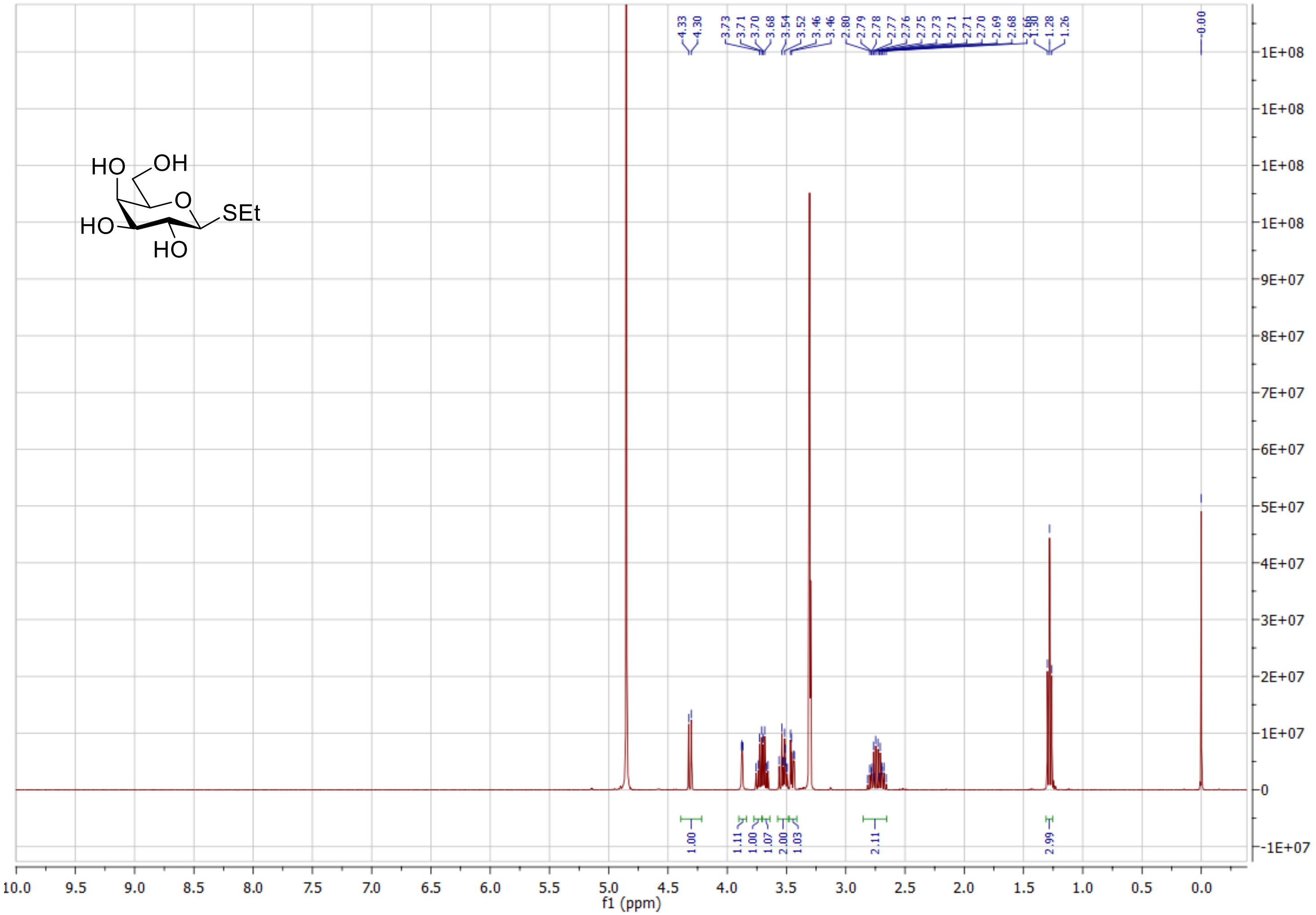

$^{13}C\{1H\}$ NMR (101 MHz, MeOD) Ethyl 1-thio-β-D-galactopyranoside 31

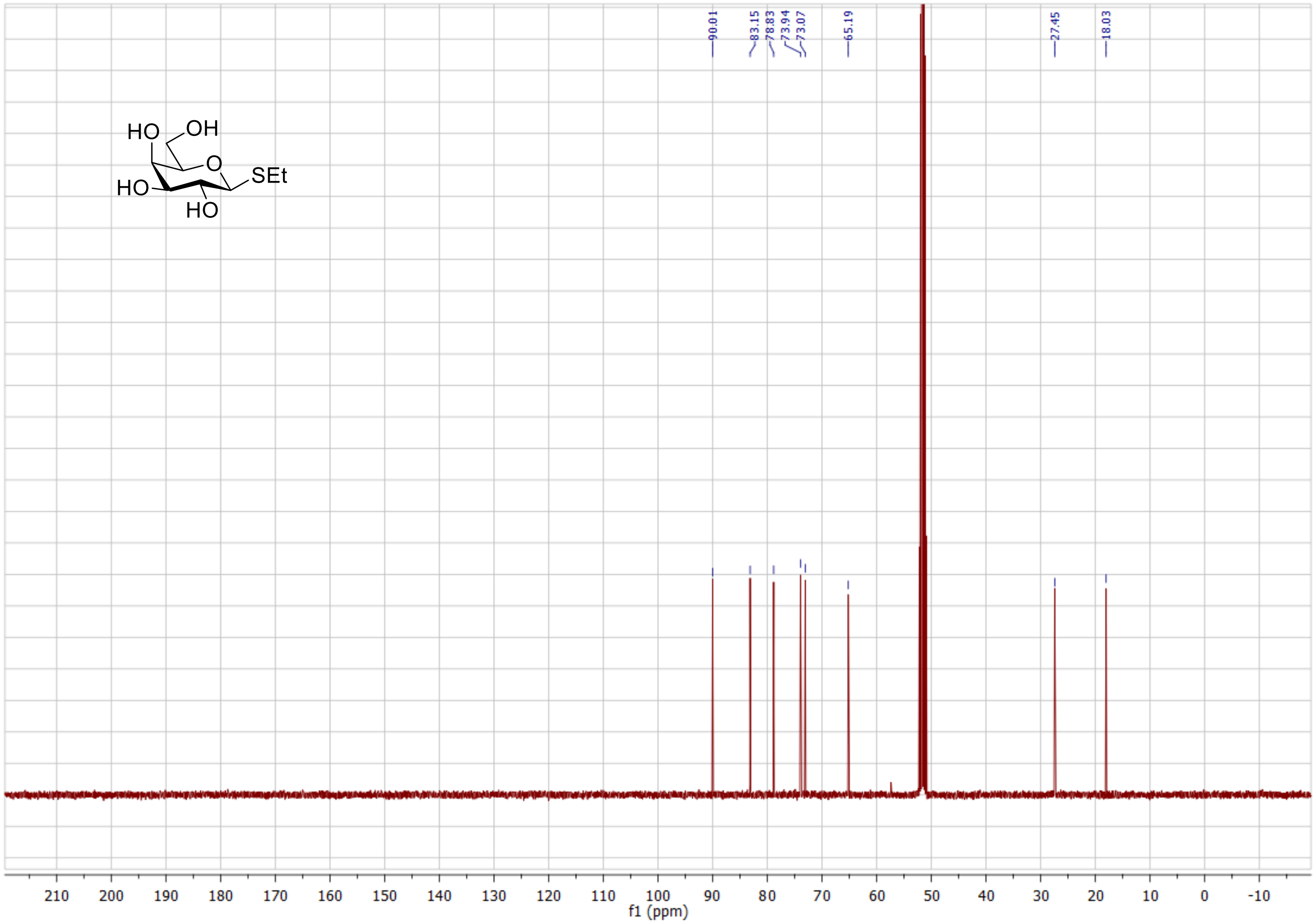

Compound 1

^{1}H NMR (400 MHz, MeOD) Ethyl 1-thio-α-ᴅ-galactopyranoside 1

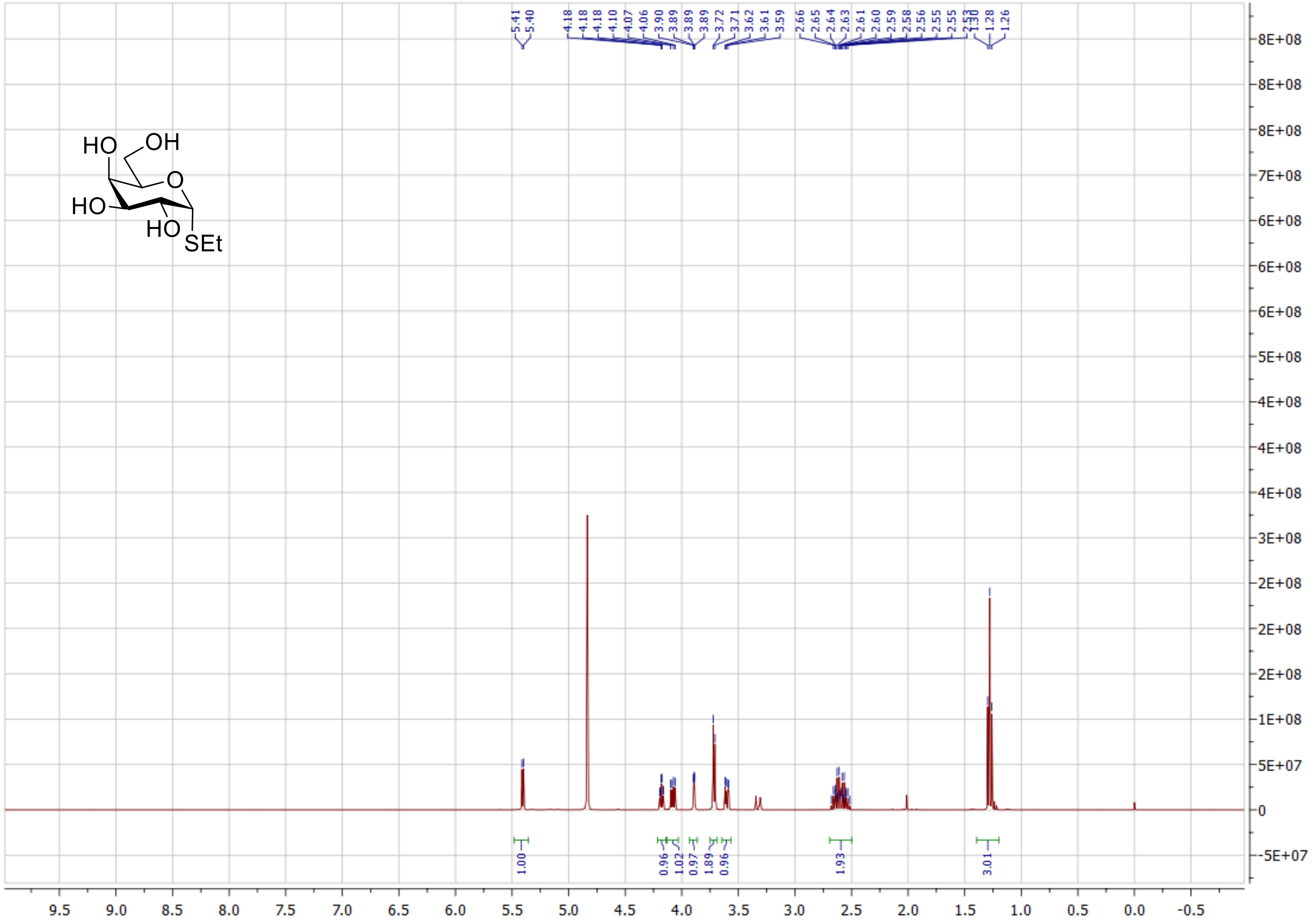

^{13}C{1H} NMR (101 MHz, MeOD) Ethyl 1-thio-α-D-galactopyranoside 1

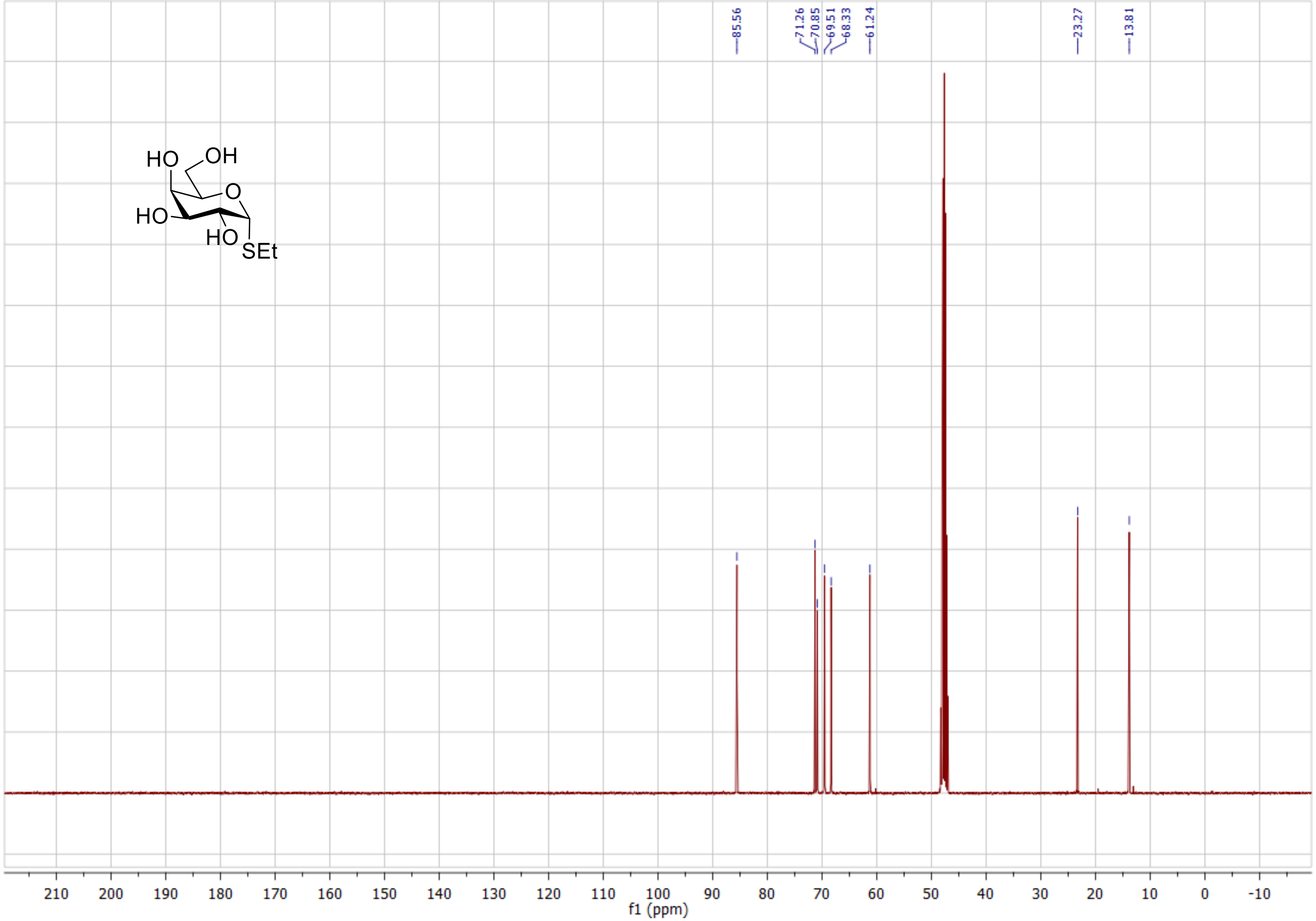

Compound S5

^{1}H NMR (400 MHz, CDCl3) Phenyl 2,3,4,6-tetra-*O*-acteyl-β-D-galactopyranoside S5

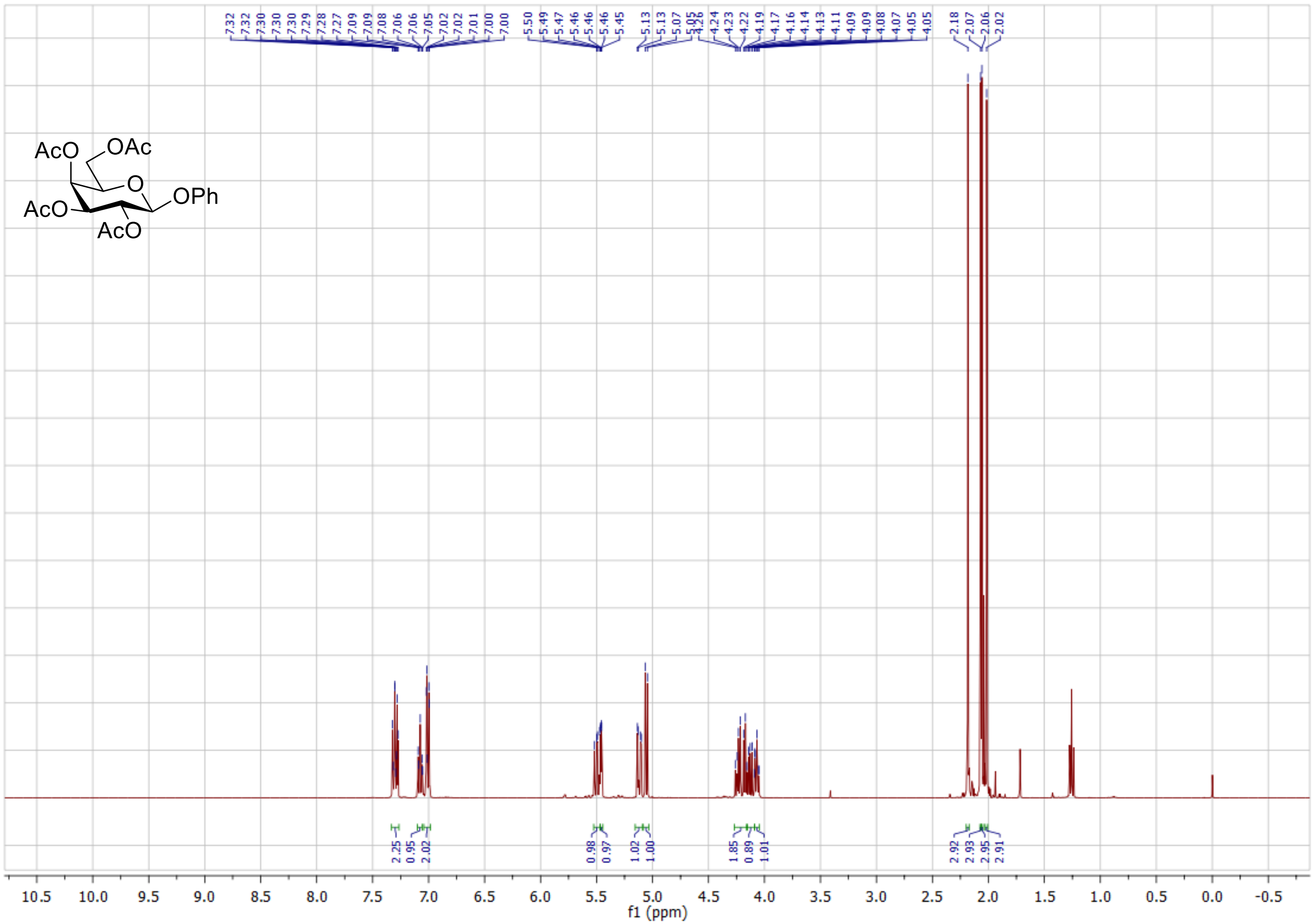

$^{13}C\{1H\}$ NMR (101 MHz, $CDCl_3$) Phenyl 2,3,4,6-tetra-*O*-acteyl-β-D-galactopyranoside S5

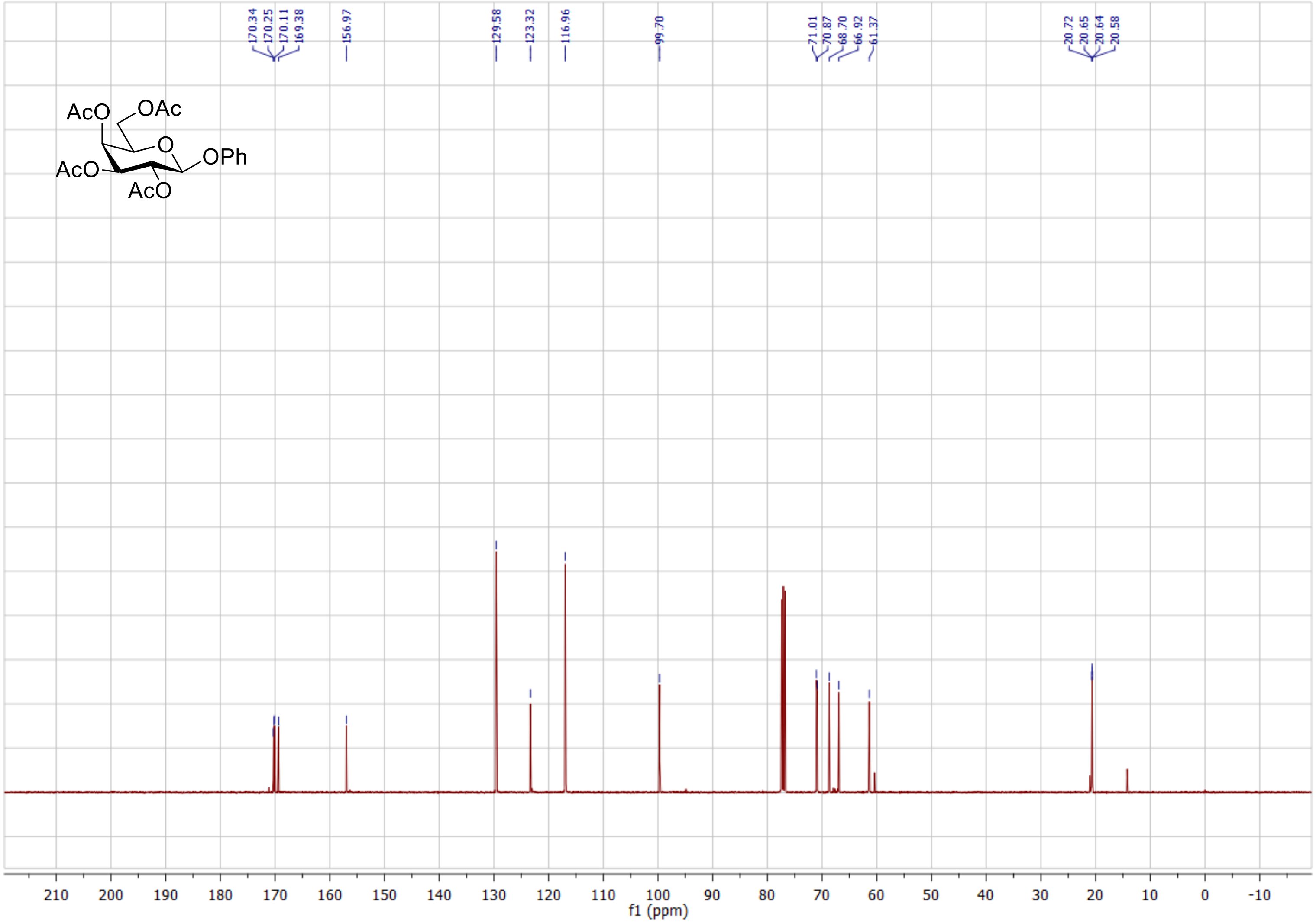

Compound S4

^{1}H NMR (400 MHz, $CDCl_3$) Phenyl 2,3,4,6-tetra-*O*-acteyl-α-D-galactopyranoside S4

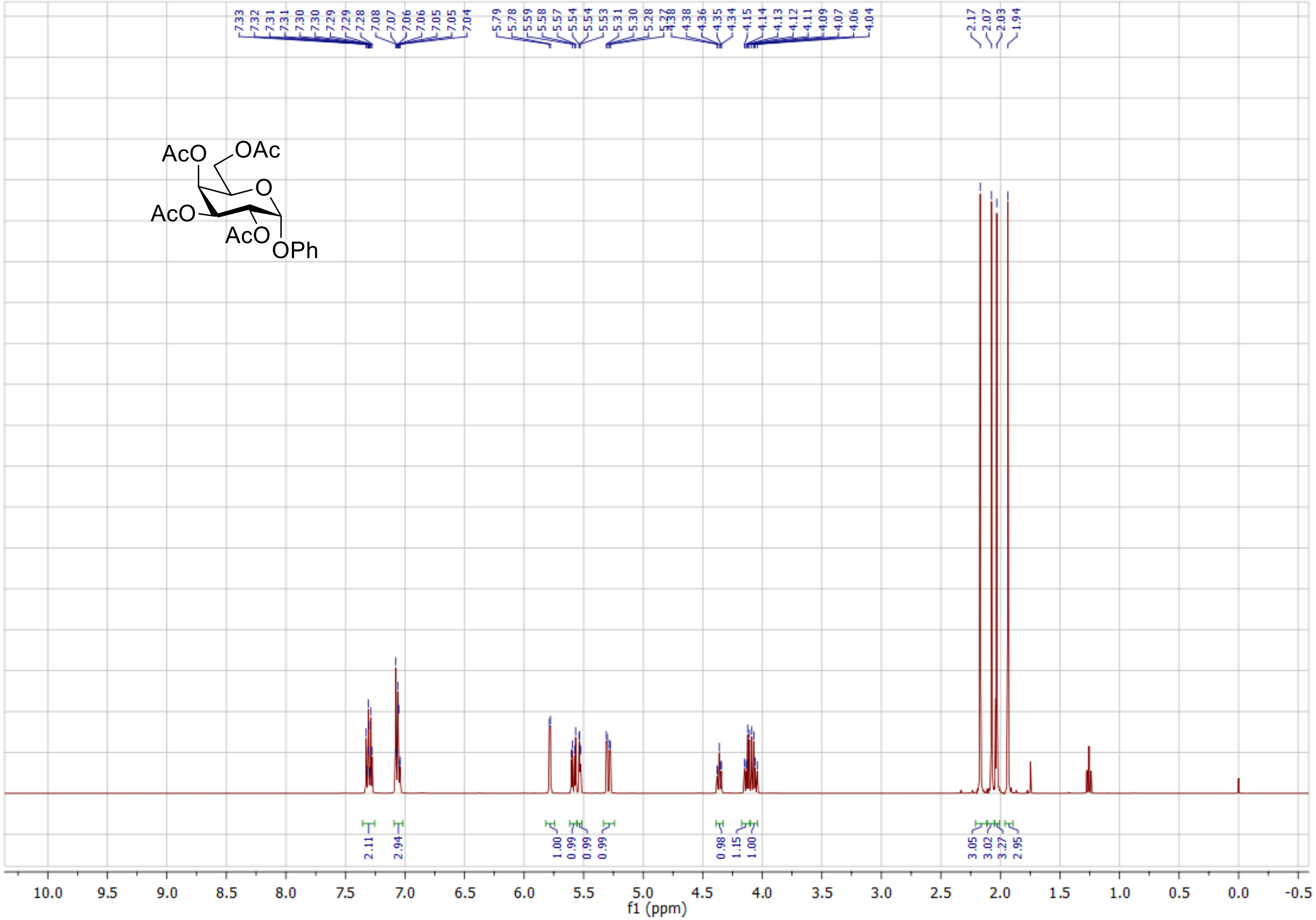

$^{13}C\{1H\}$ NMR (101 MHz, $CDCl_3$) Phenyl 2,3,4,6-tetra-*O*-acteyl-α-D-galactopyranoside S4

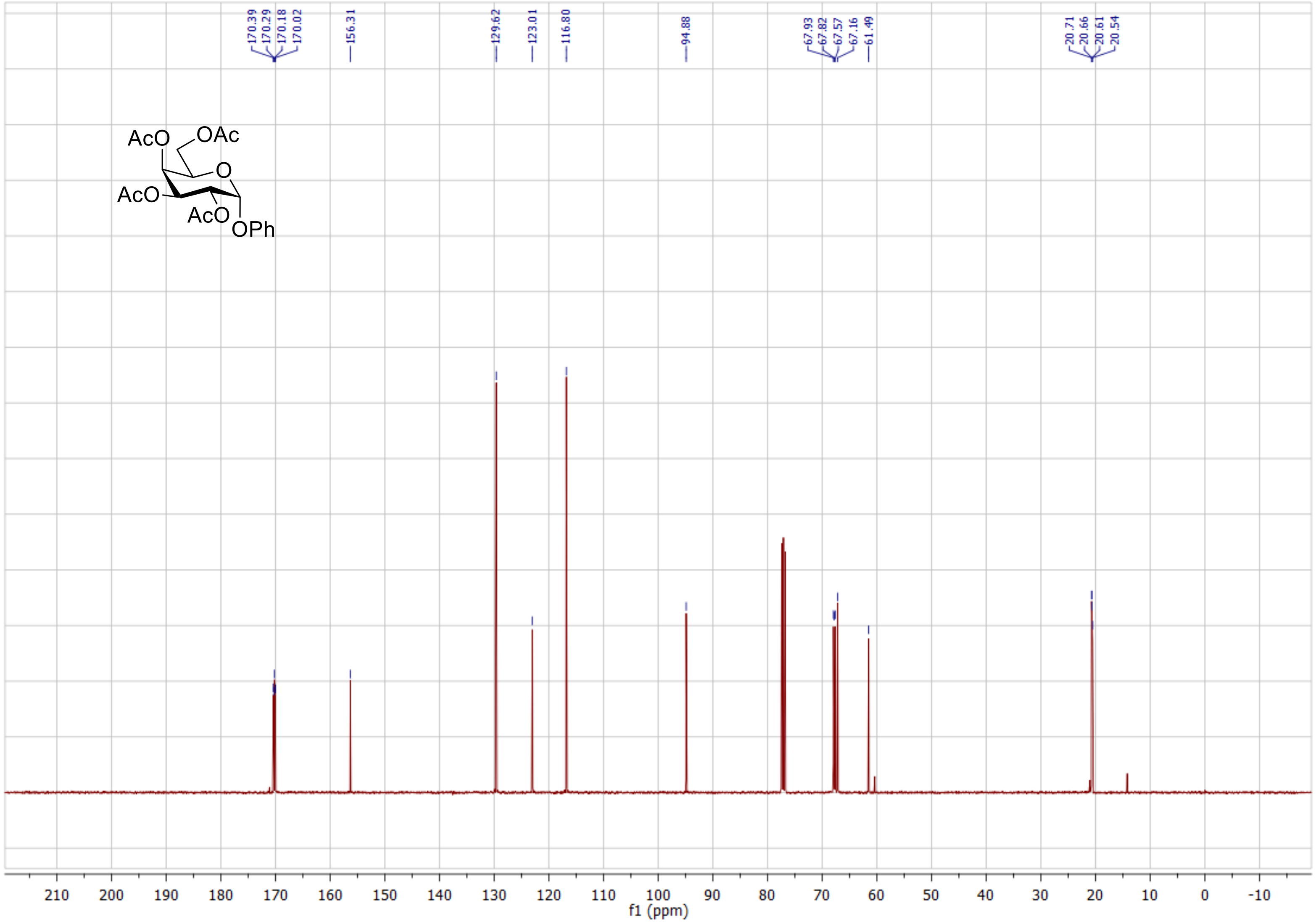

Compound 36

^{1}H NMR (400 MHz, MeOD) Phenyl β-D-galactopyranoside 36

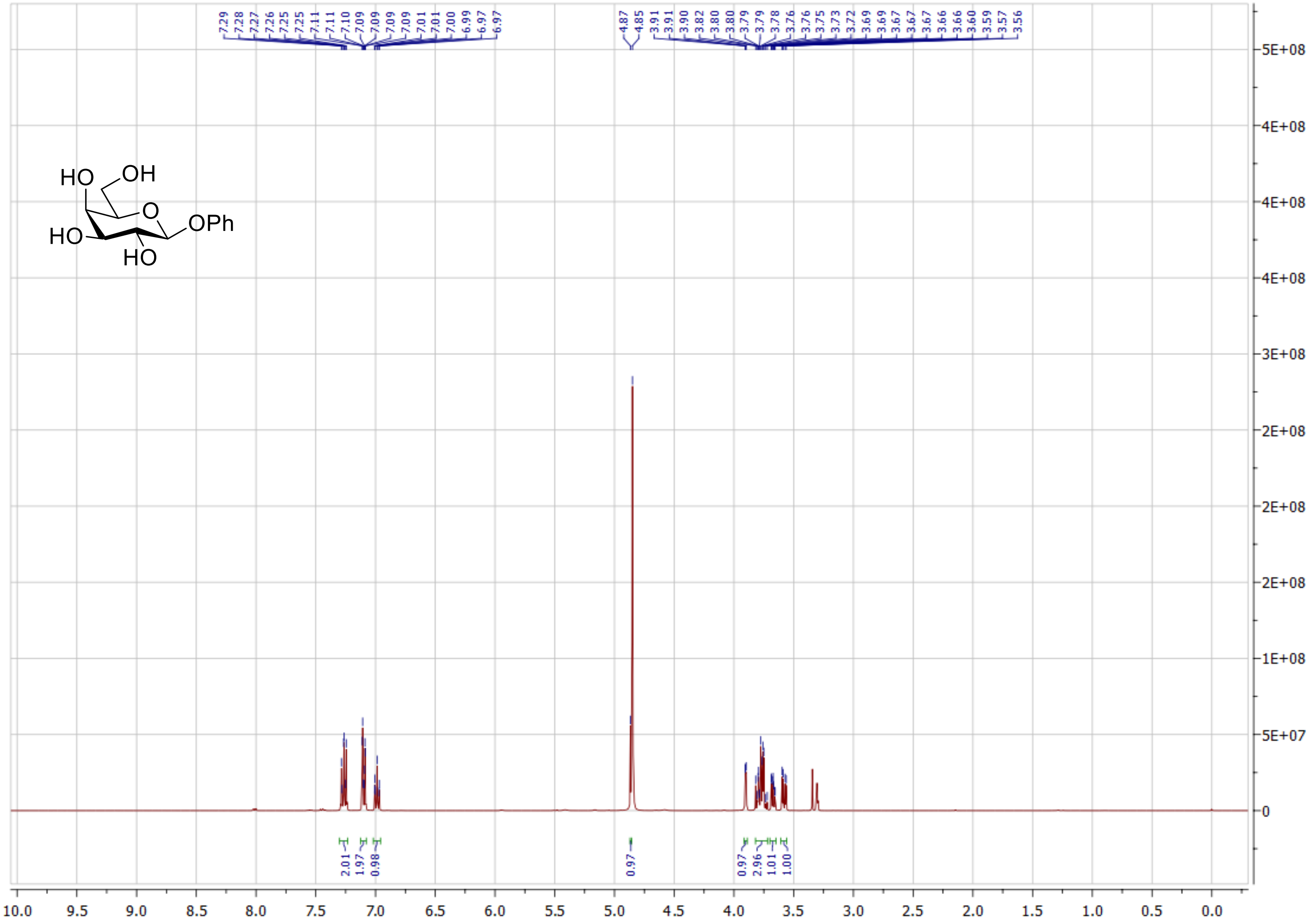

$^{13}C\{1H\}$ NMR (101 MHz, MeOD) Phenyl β-D-galactopyranoside 36

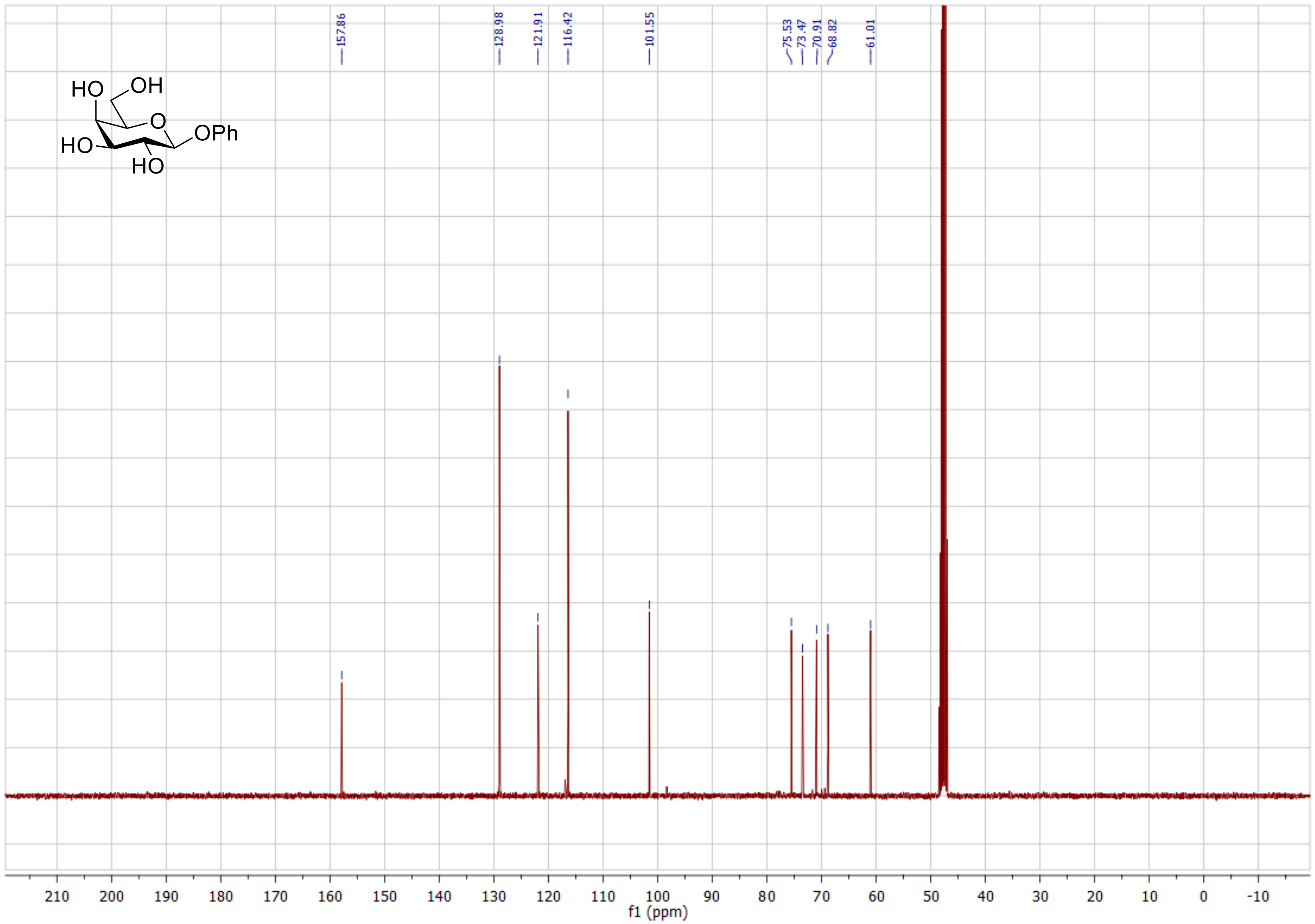

Compound 5

^{1}H NMR (400 MHz, MeOD) Phenyl α-D-galactopyranoside 5

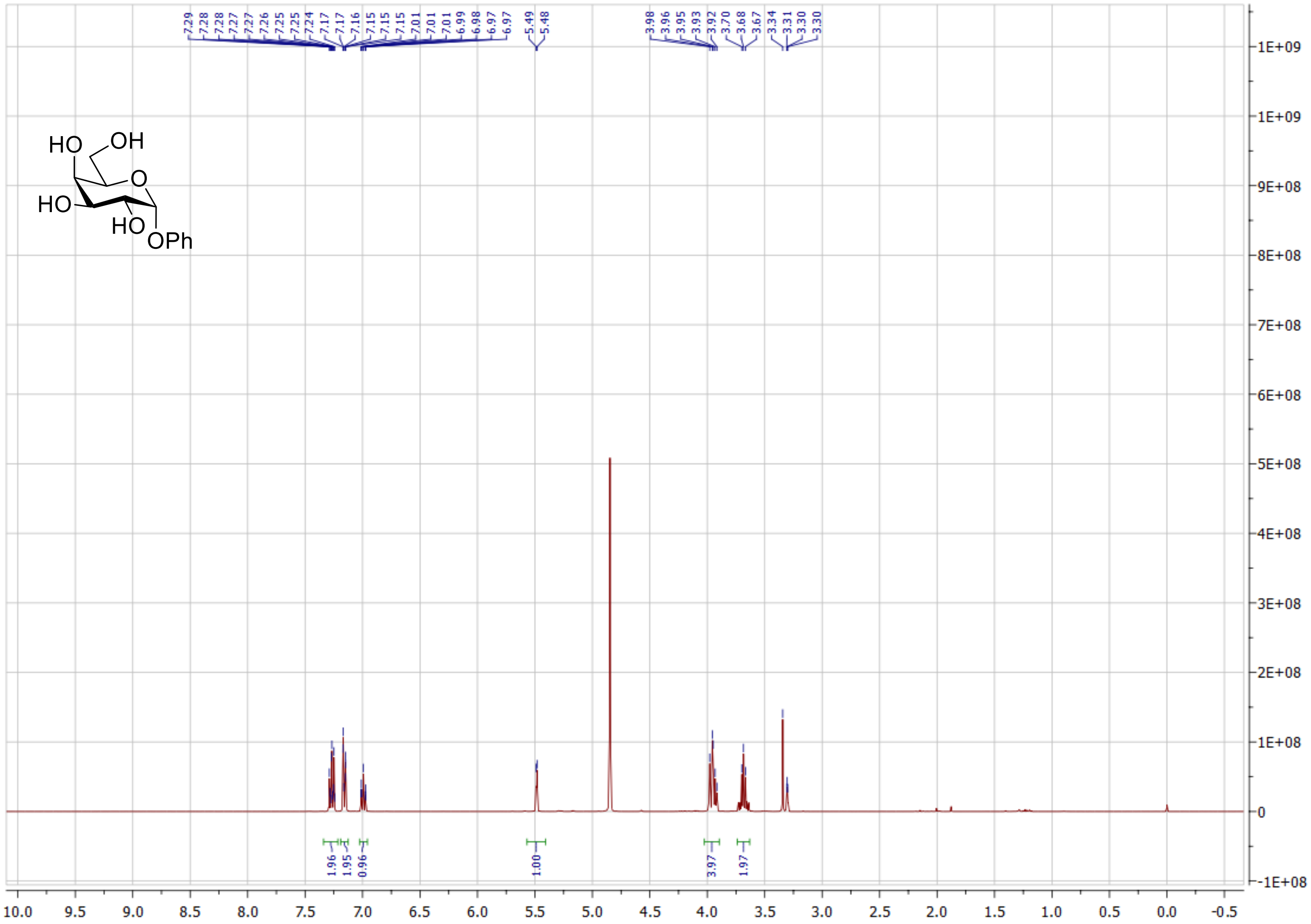

$^{13}C\{1H\}$ NMR (101 MHz, MeOD) Phenyl α-D-galactopyranoside 5

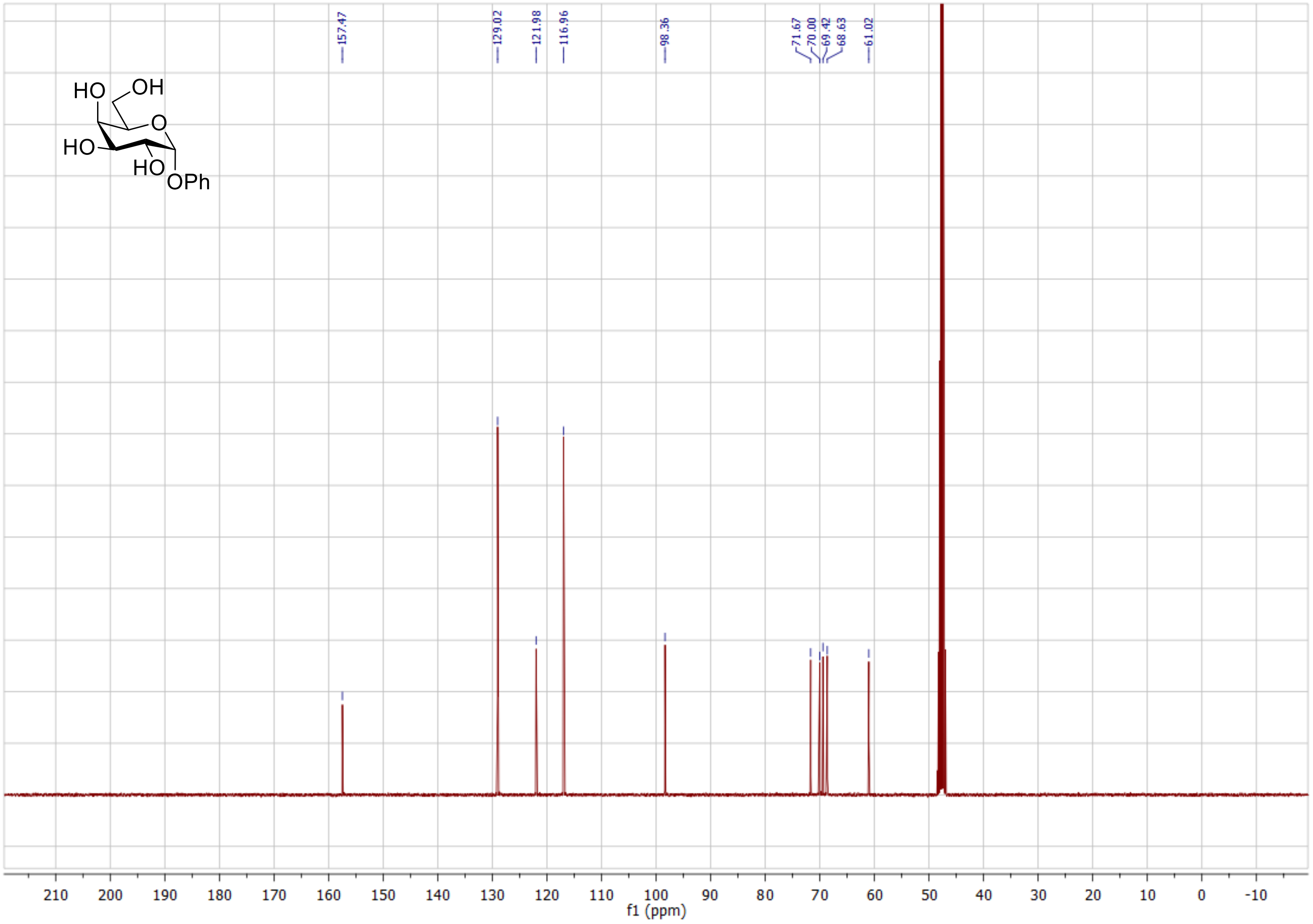

Compound S6

^{1}H NMR (400 MHz, CDCl$_3$) Ethyl 2,3,4,6-tetra-*O*-acteyl-β-D-galactopyranoside S6

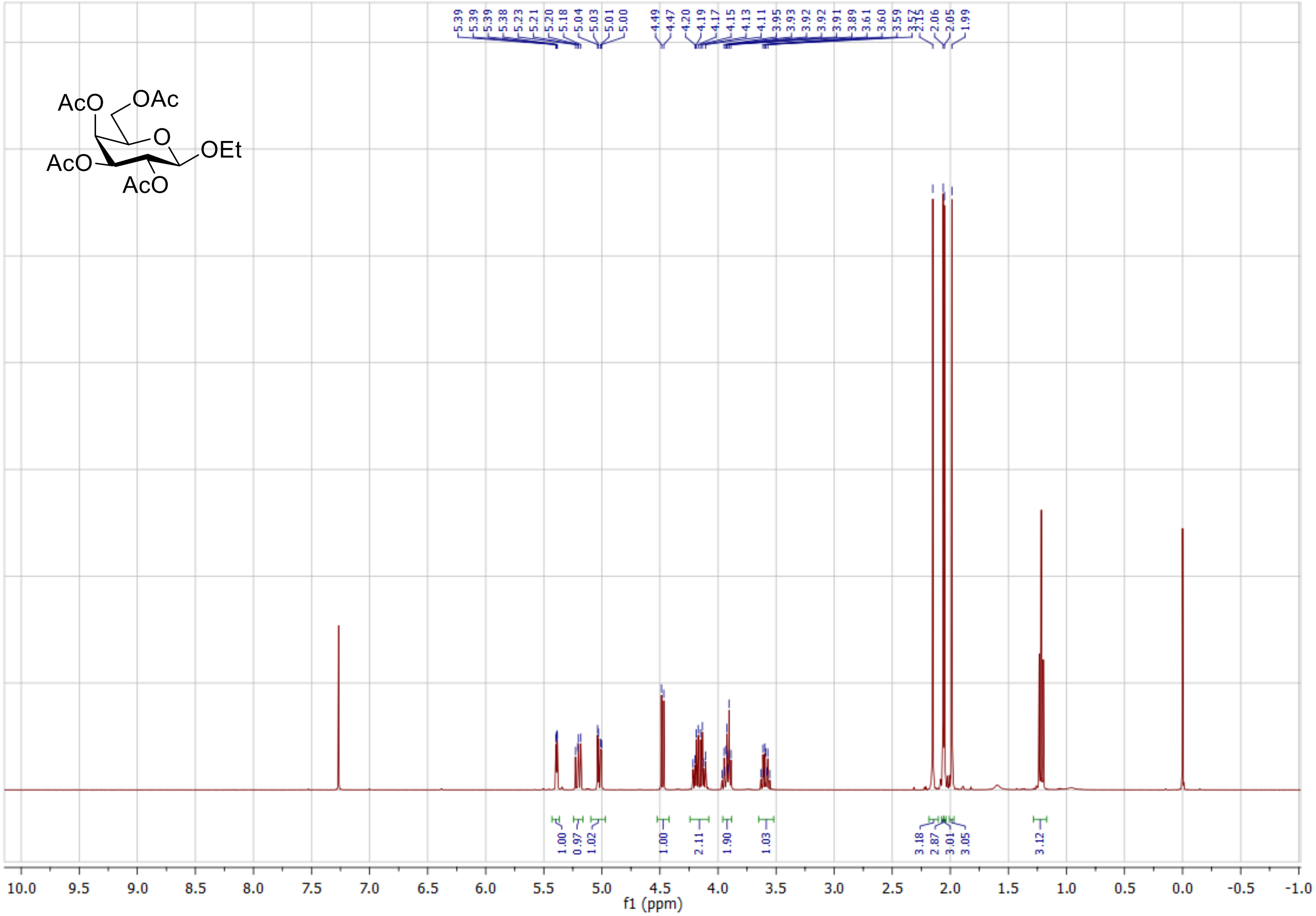

$^{13}C\{1H\}$ NMR (101 MHz, $CDCl_3$) Ethyl 2,3,4,6-tetra-*O*-acteyl-β-D-galactopyranoside S6

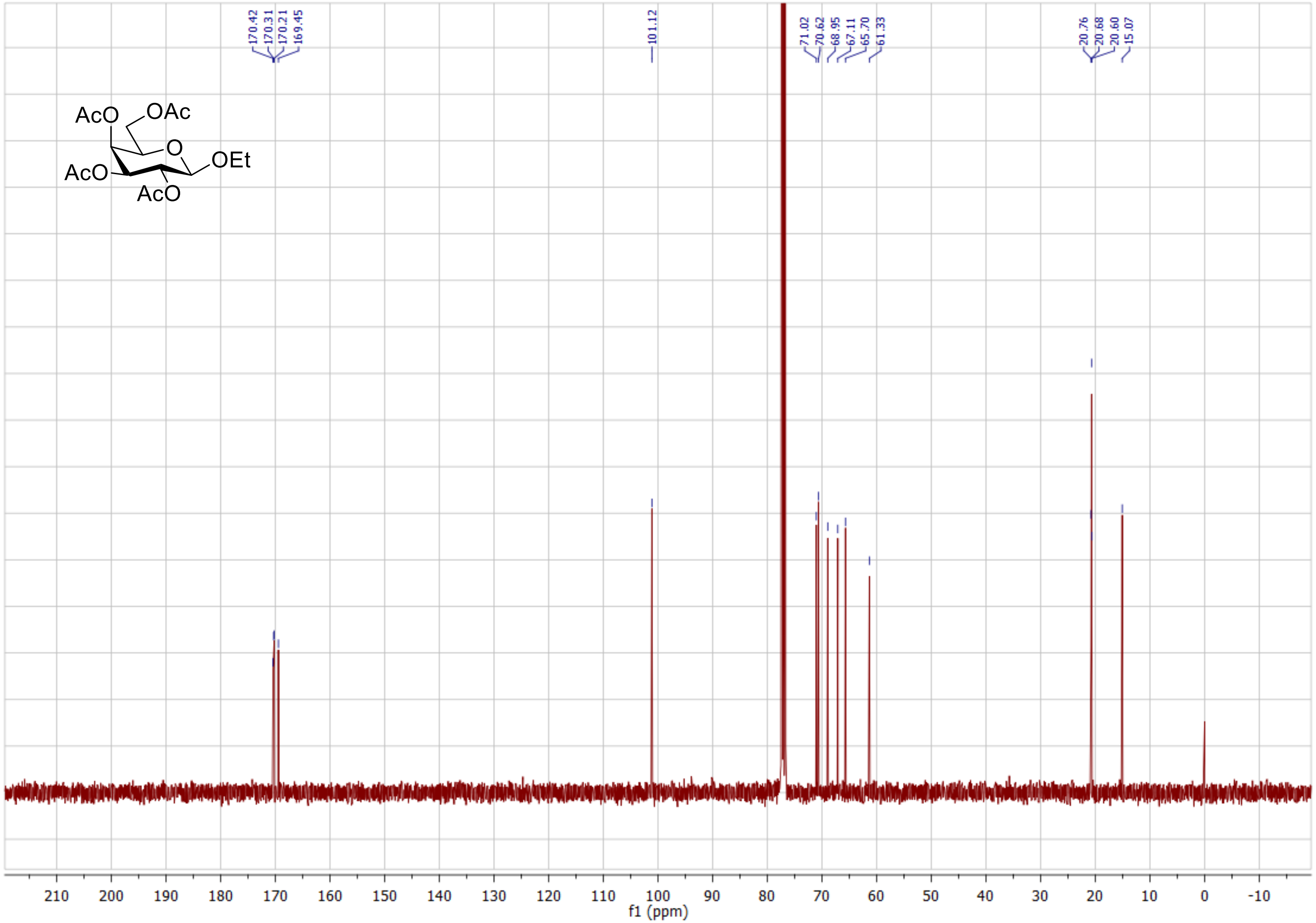

Compound 40

^{1}H NMR (400 MHz, MeOD) Ethyl β-D-galactopyranoside 40

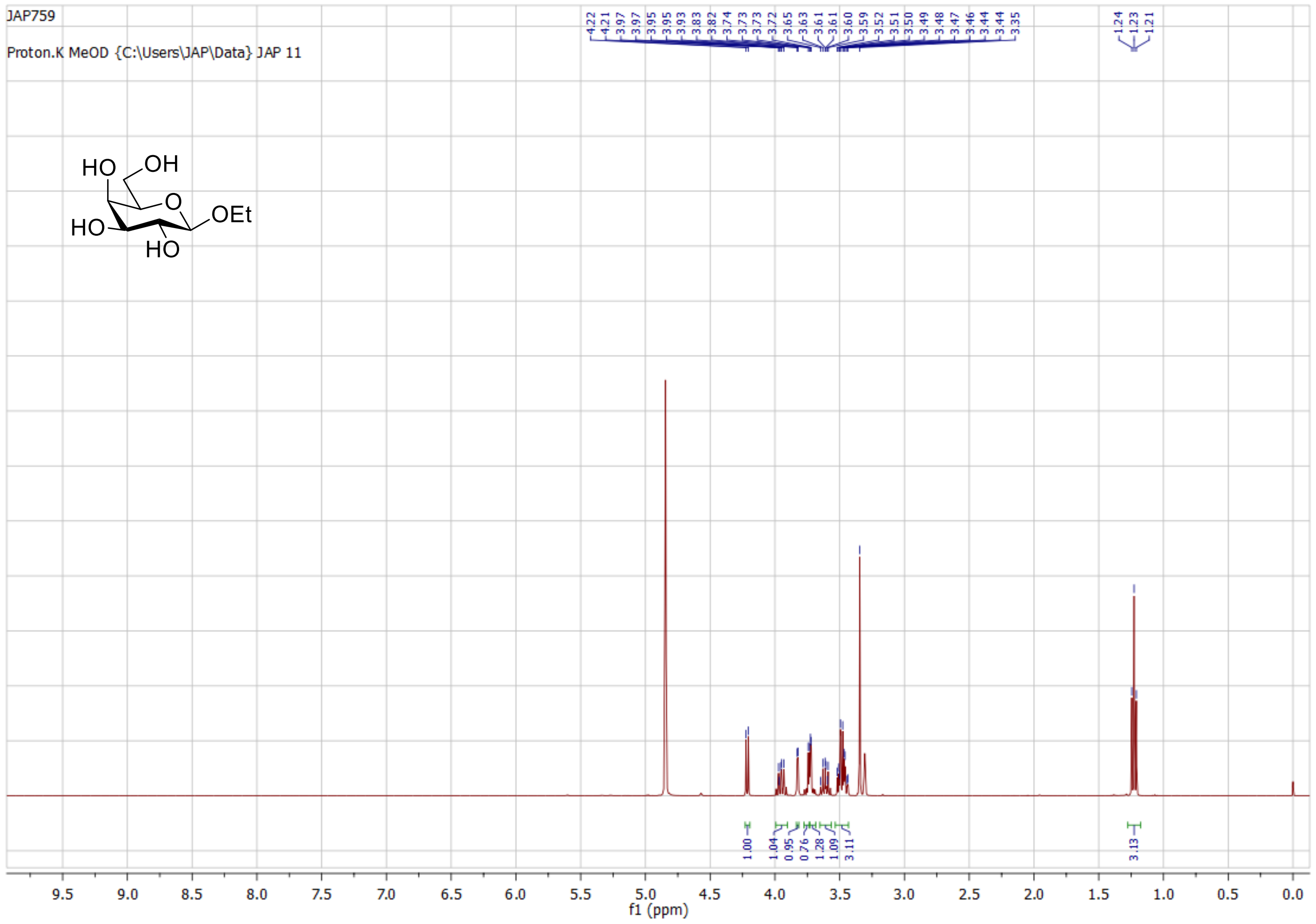

$^{13}C\{1H\}$ NMR (101 MHz, MeOD) Ethyl β-D-galactopyranoside 40

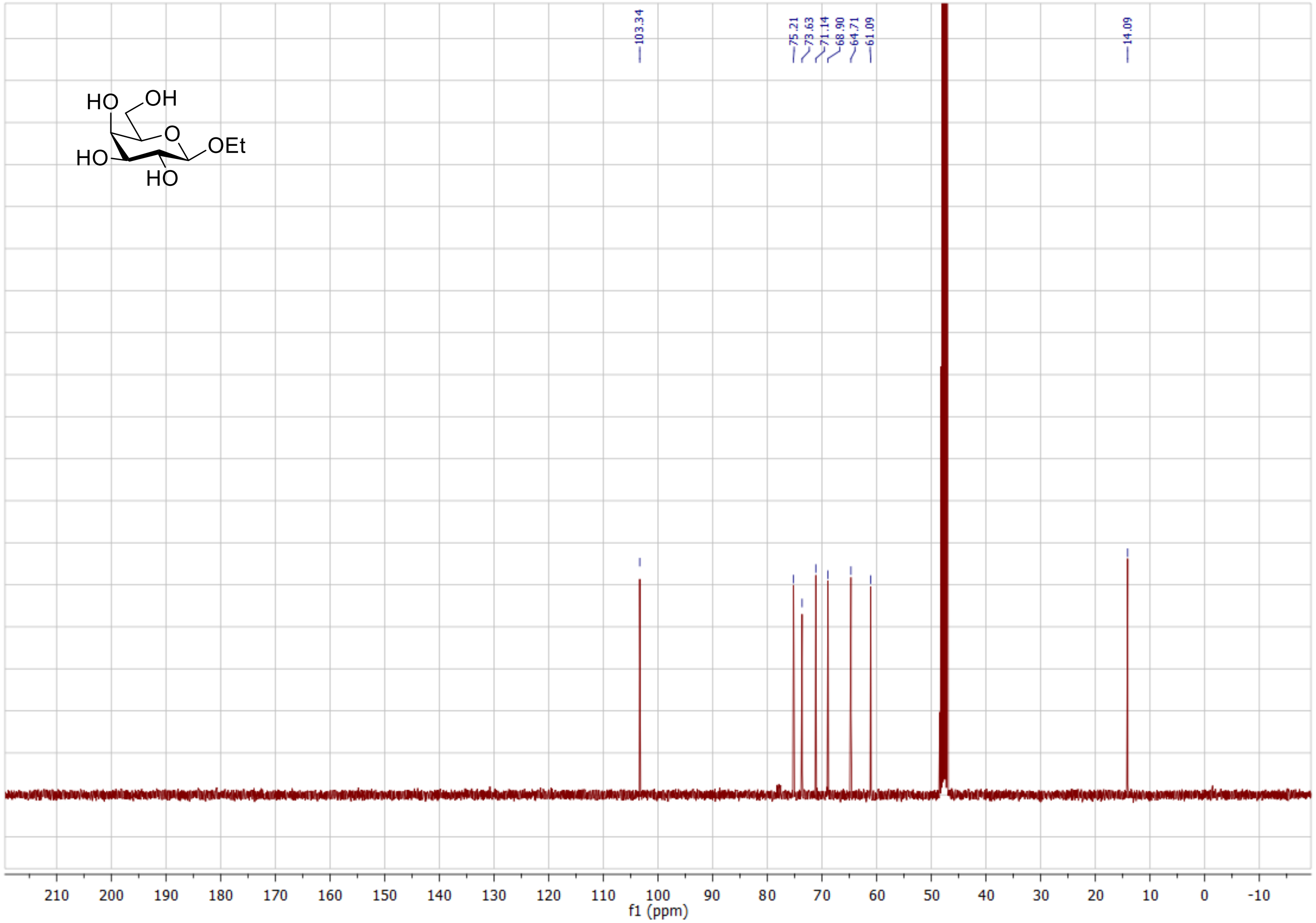

Compound 7

^{1}H NMR (400 MHz, MeOD) Ethyl α-D-galactopyranoside 7

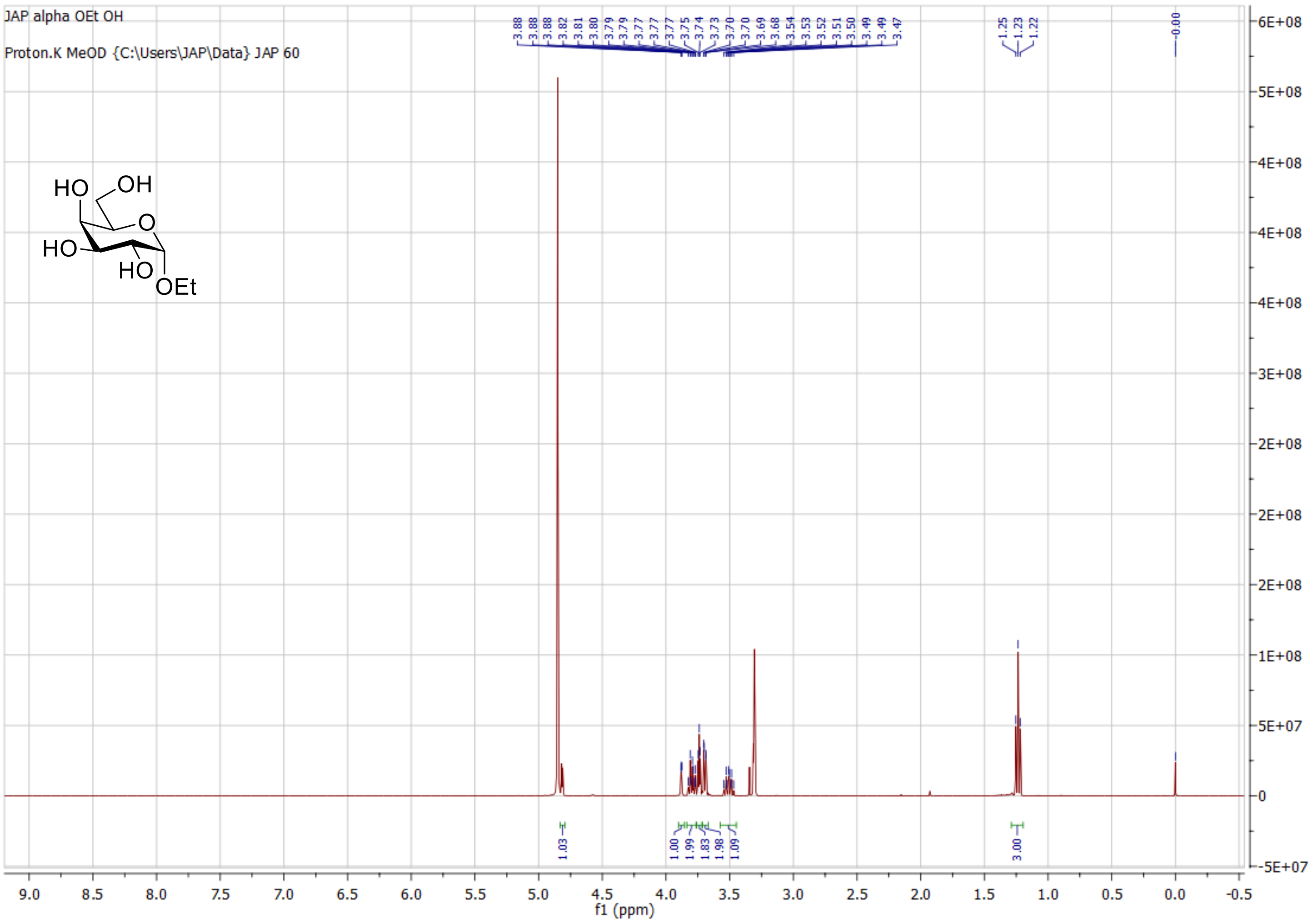

$^{13}C\{1H\}$ NMR (101 MHz, MeOD) Ethyl α-D-galactopyranoside 7

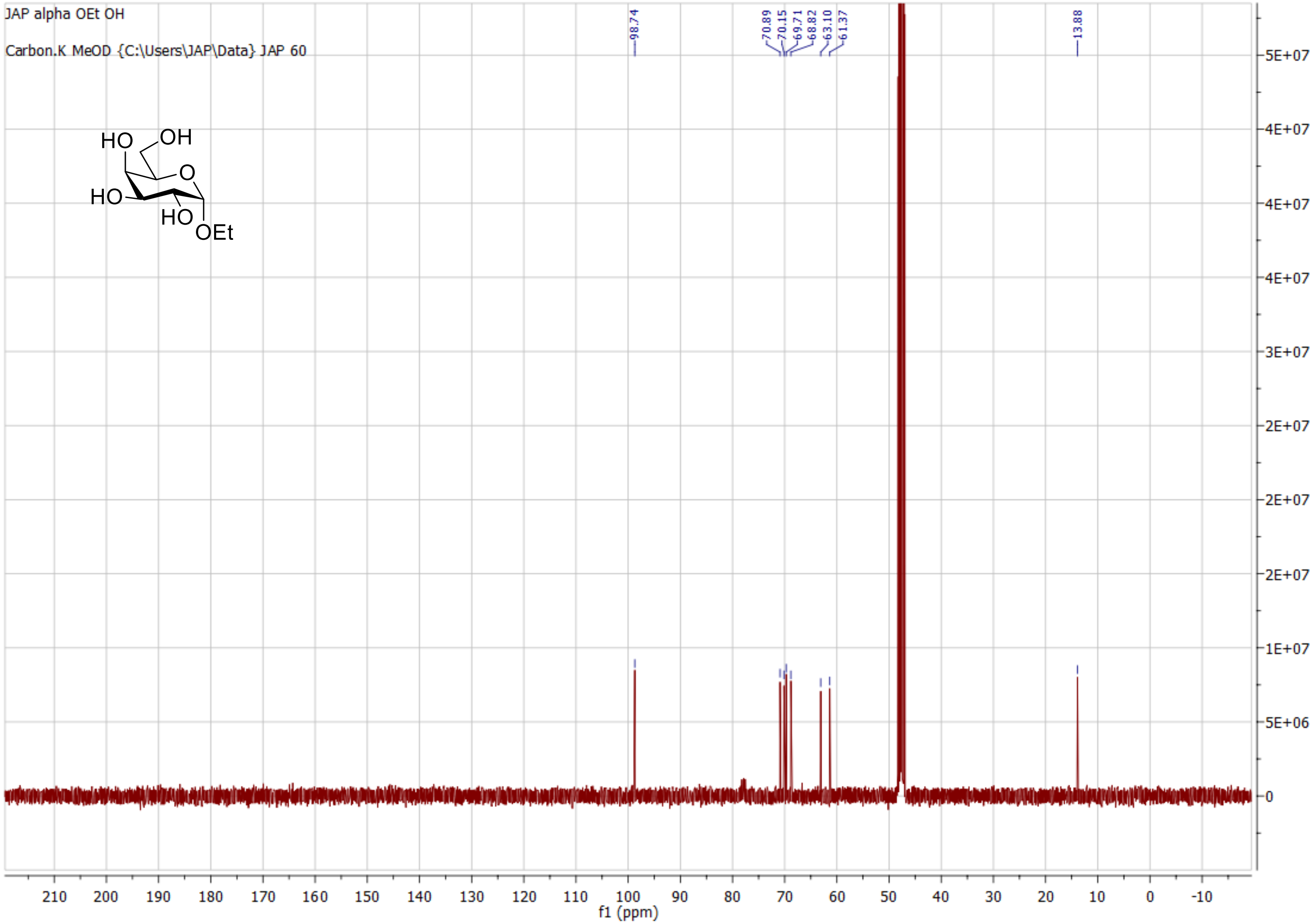

Compound 43

^{1}H NMR (400 MHz, MeOD): Cyclohexyl 1-thio-β-D-galactopyranoside 43

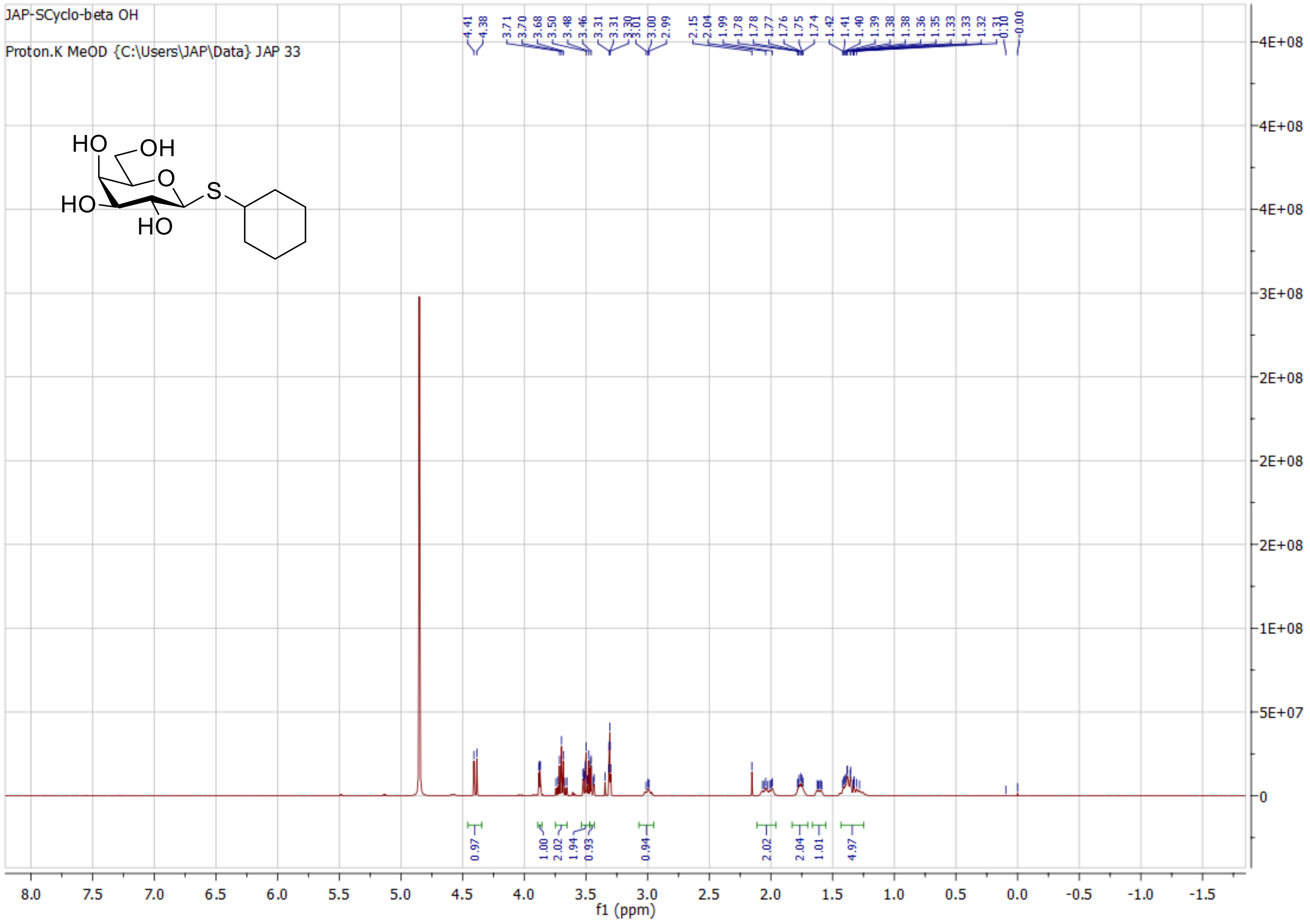

$^{13}C\{1H\}$ NMR (101 MHz, MeOD): Cyclohexyl 1-thio-β-D-galactopyranoside 43

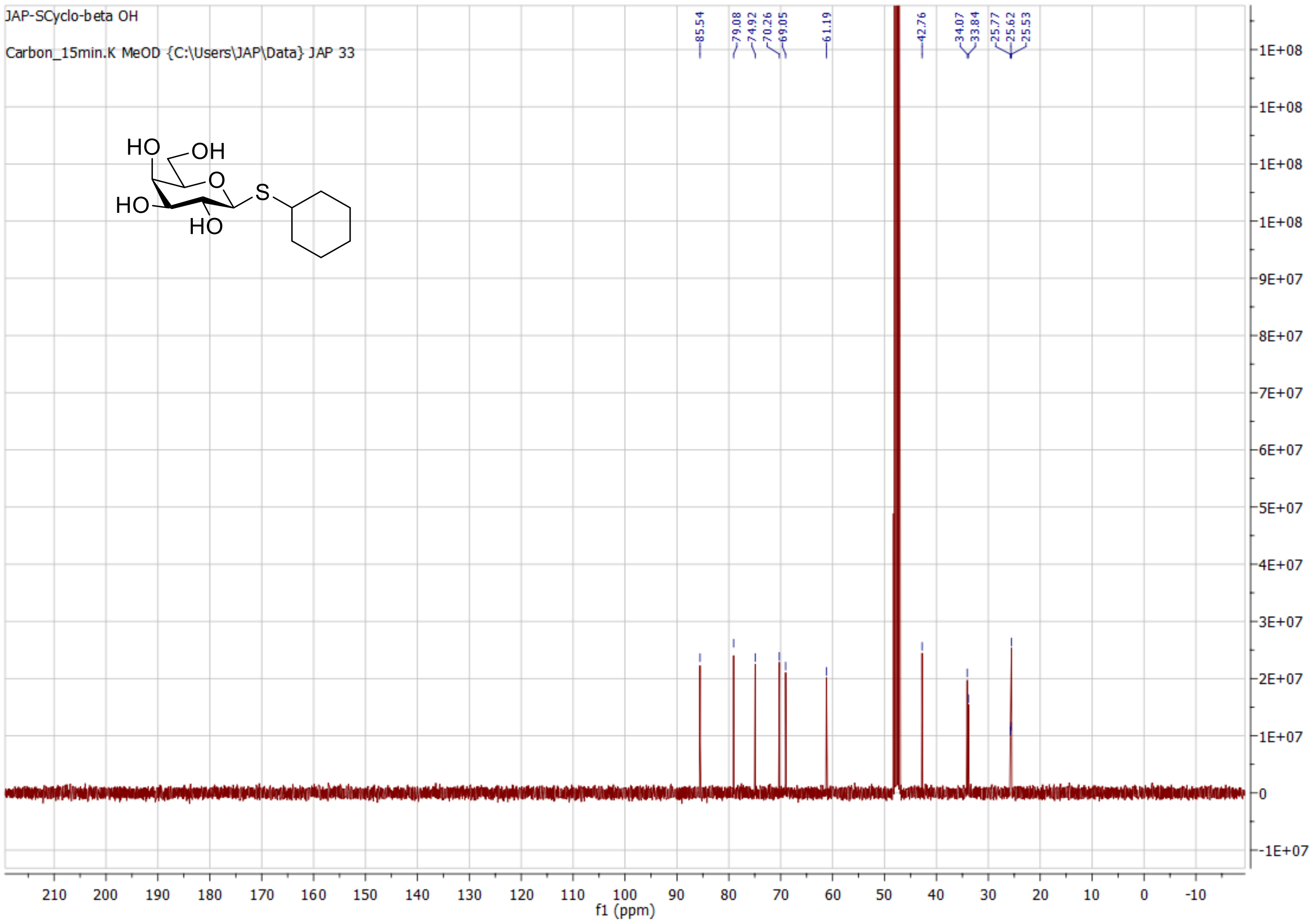

Compound S7

^{1}H NMR (400 MHz, $CDCl_3$): Trifluoroethyl 2,3,4,6-tetra-*O*-acetyl-1-thio-β-D-galactopyranoside S7

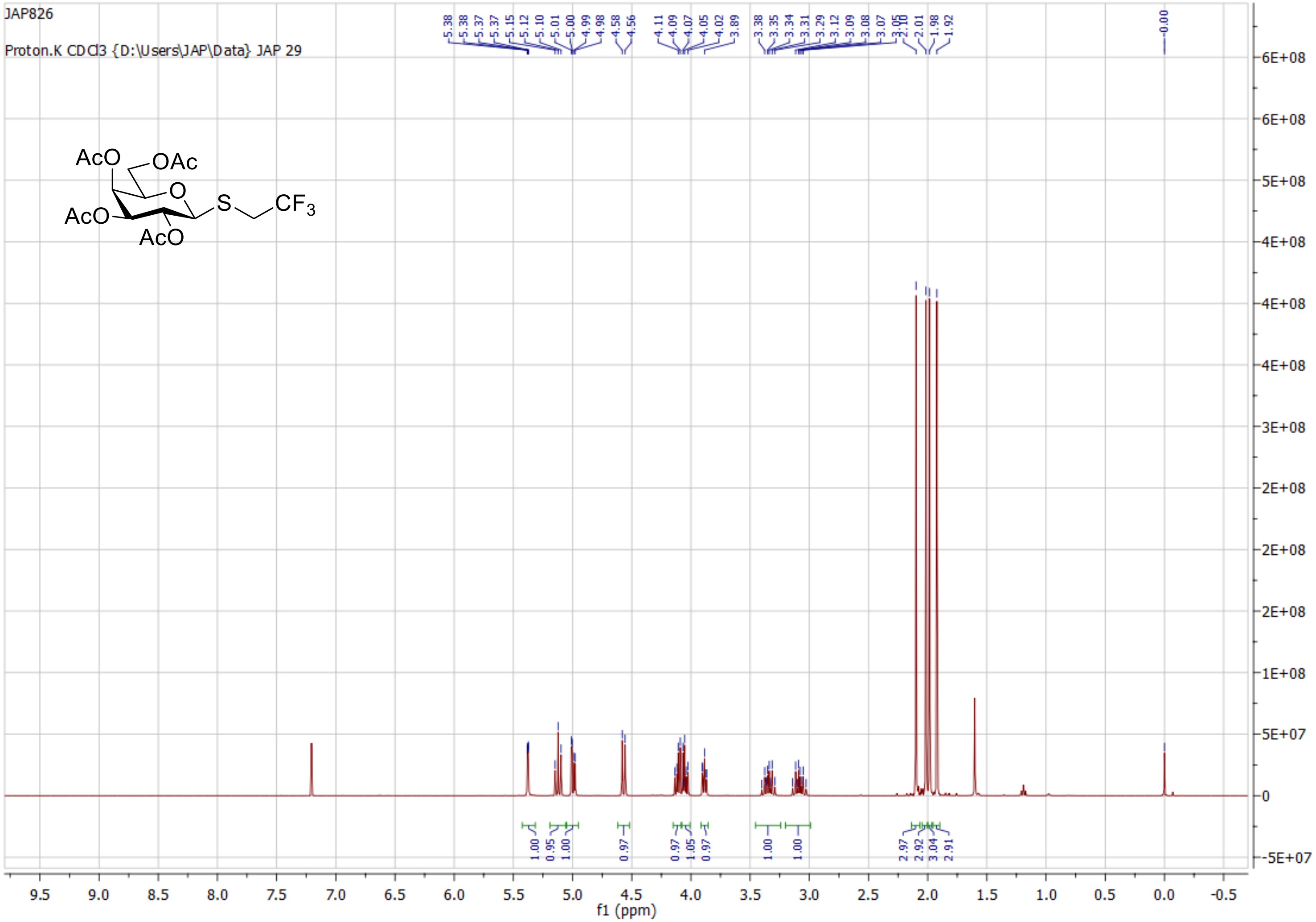

COSY (400 × 400 MHz, $CDCl_3$): Trifluoroethyl 2,3,4,6-tetra-*O*-acetyl-1-thio-β-D-galactopyranoside S7

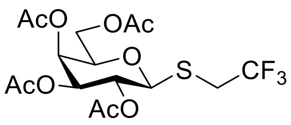

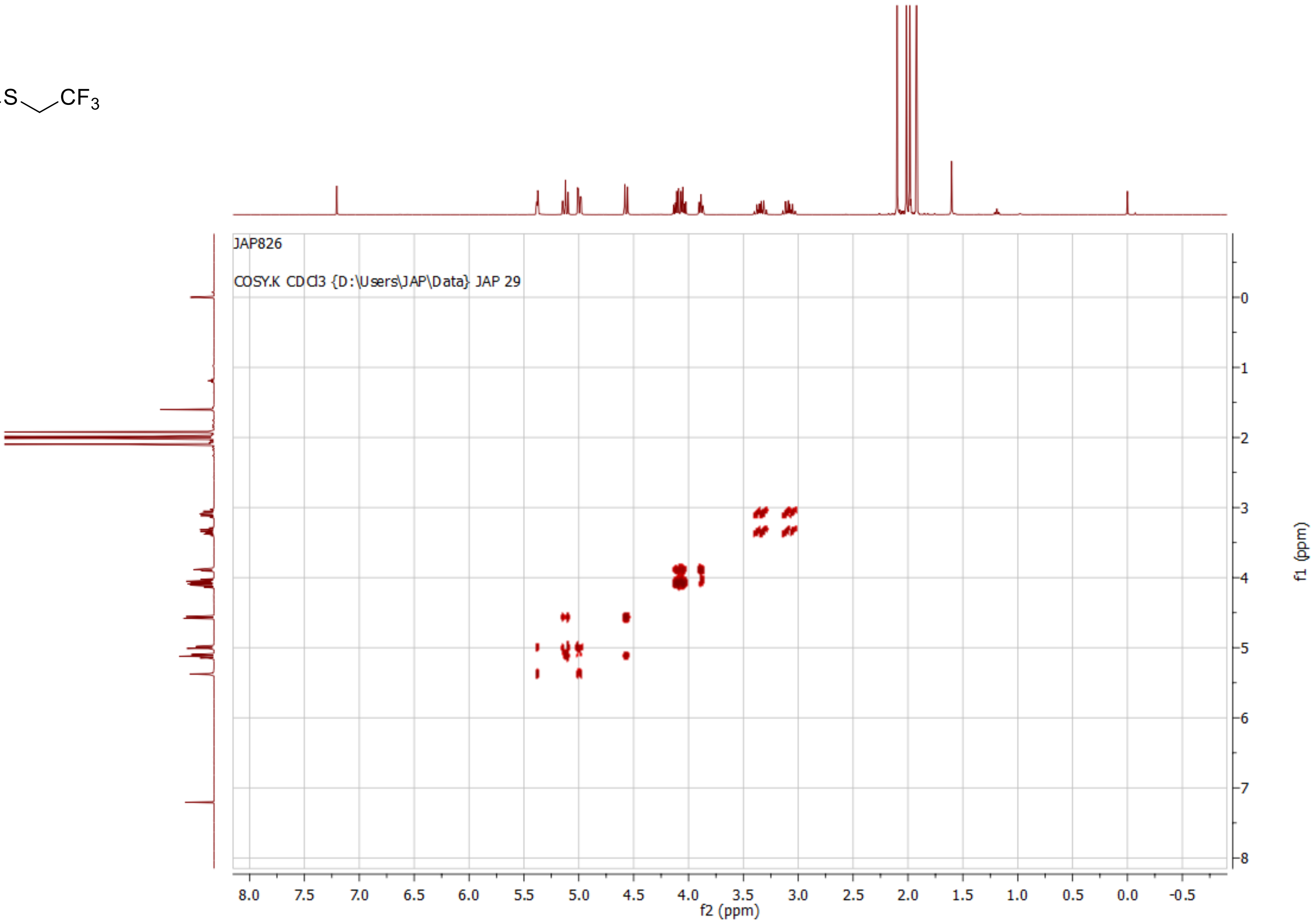

HSQC (400 × 101 MHz, CDCl₃): Trifluoroethyl 2,3,4,6-tetra-*O*-acetyl-1-thio-β-D-galactopyranoside S7

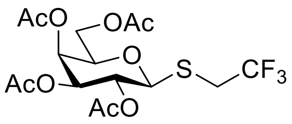

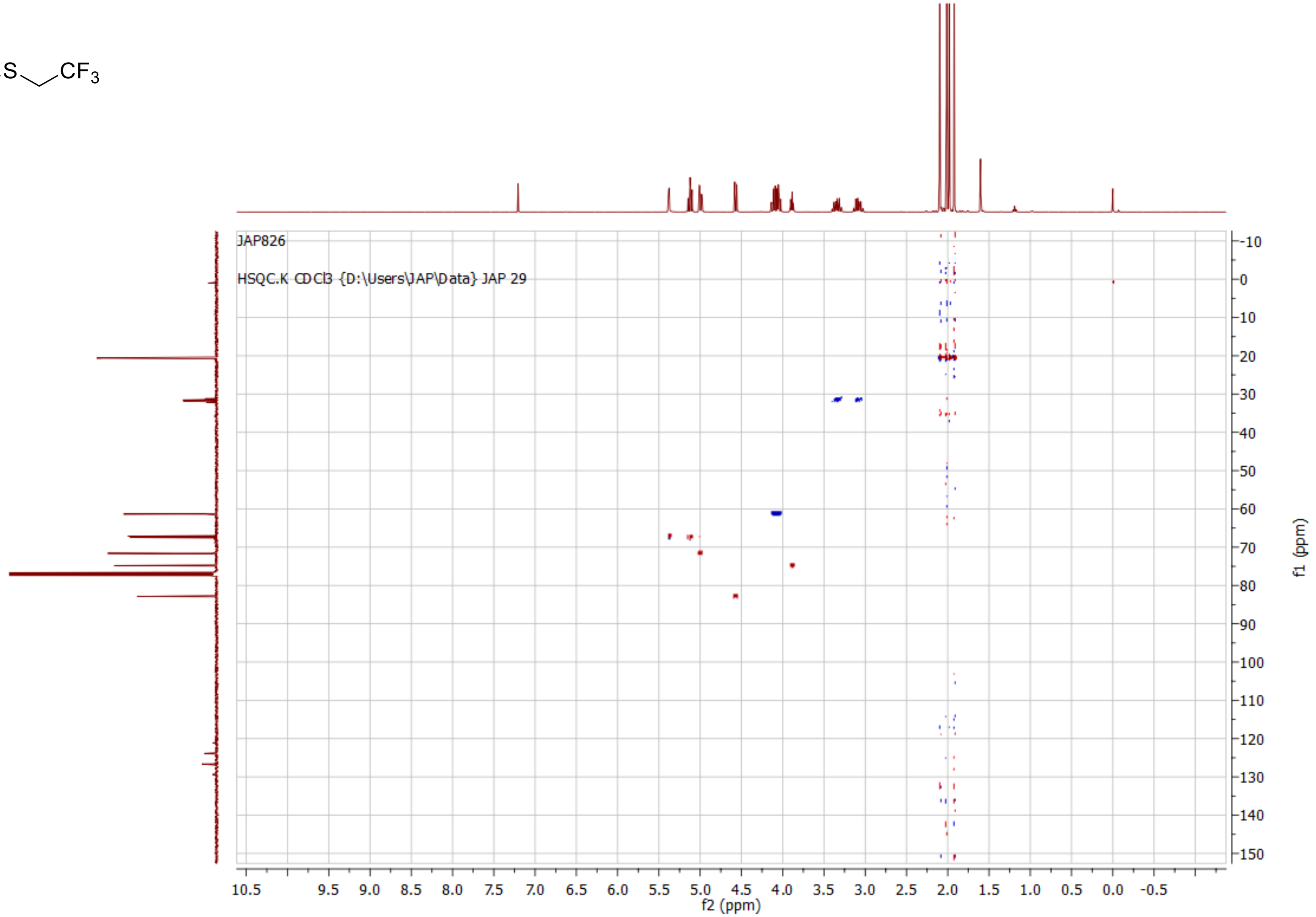

^{13}C{1H} NMR (101 MHz, CDCl₃): Trifluoroethyl 2,3,4,6-tetra-*O*-acetyl-1-thio-β-D-galactopyranoside S7

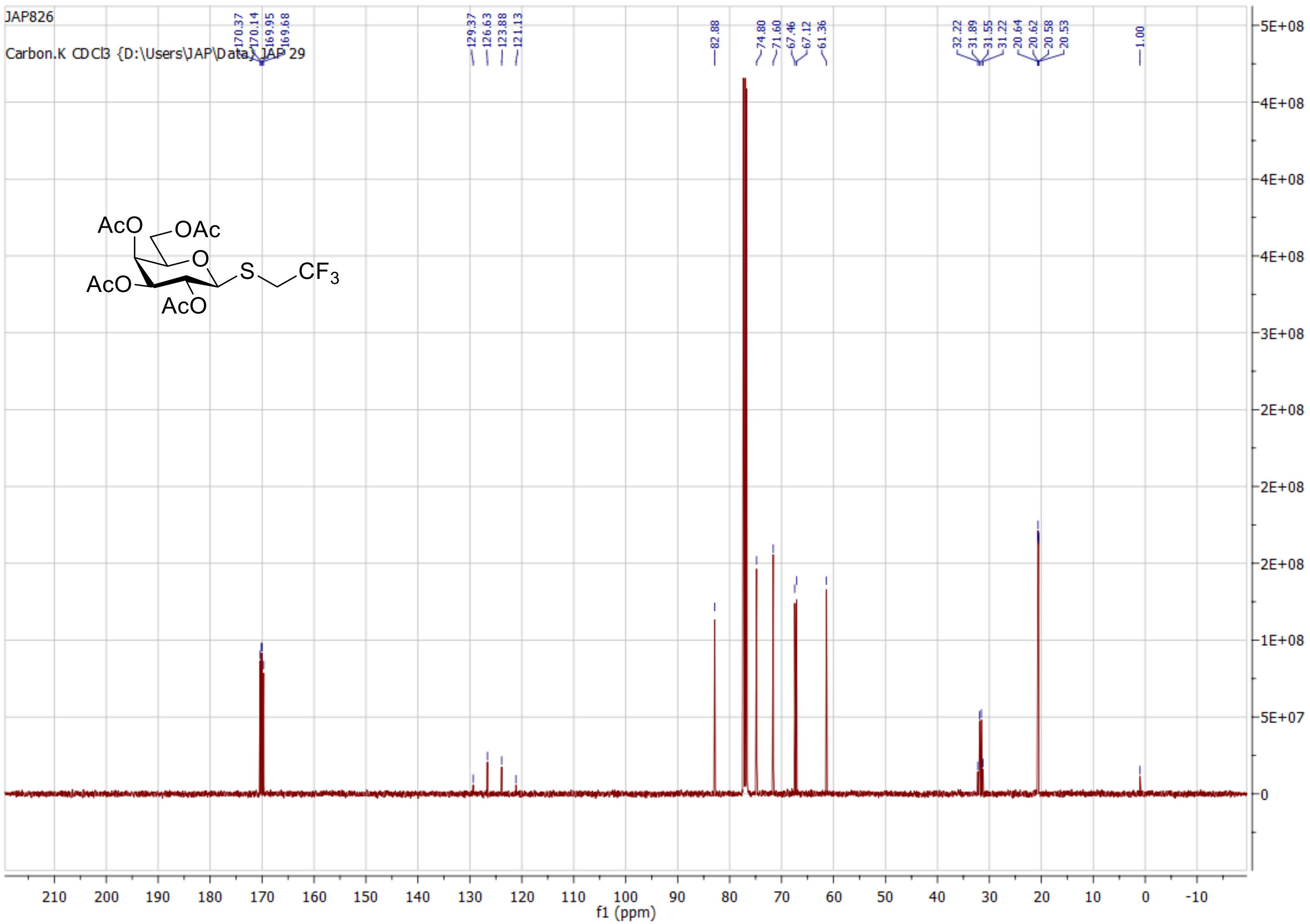

^{19}F NMR (376 MHz, CDCl3): Trifluoroethyl 2,3,4,6-tetra-*O*-acetyl-1-thio-β-D-galactopyranoside S7

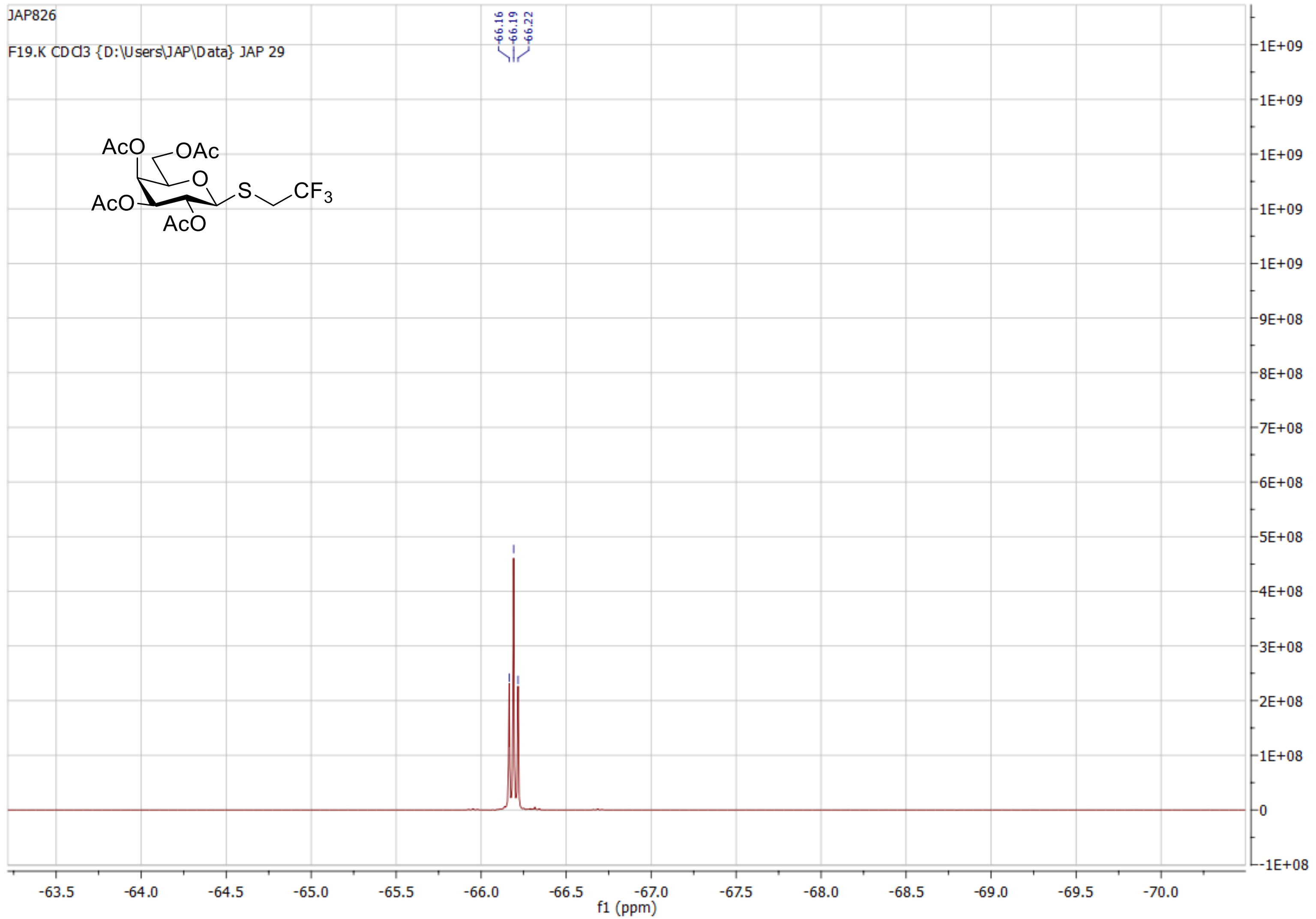

Compound 45

^{1}H NMR (400 MHz, MeOD): Trifluoroethyl 1-thio-β-D-galactopyranoside 45

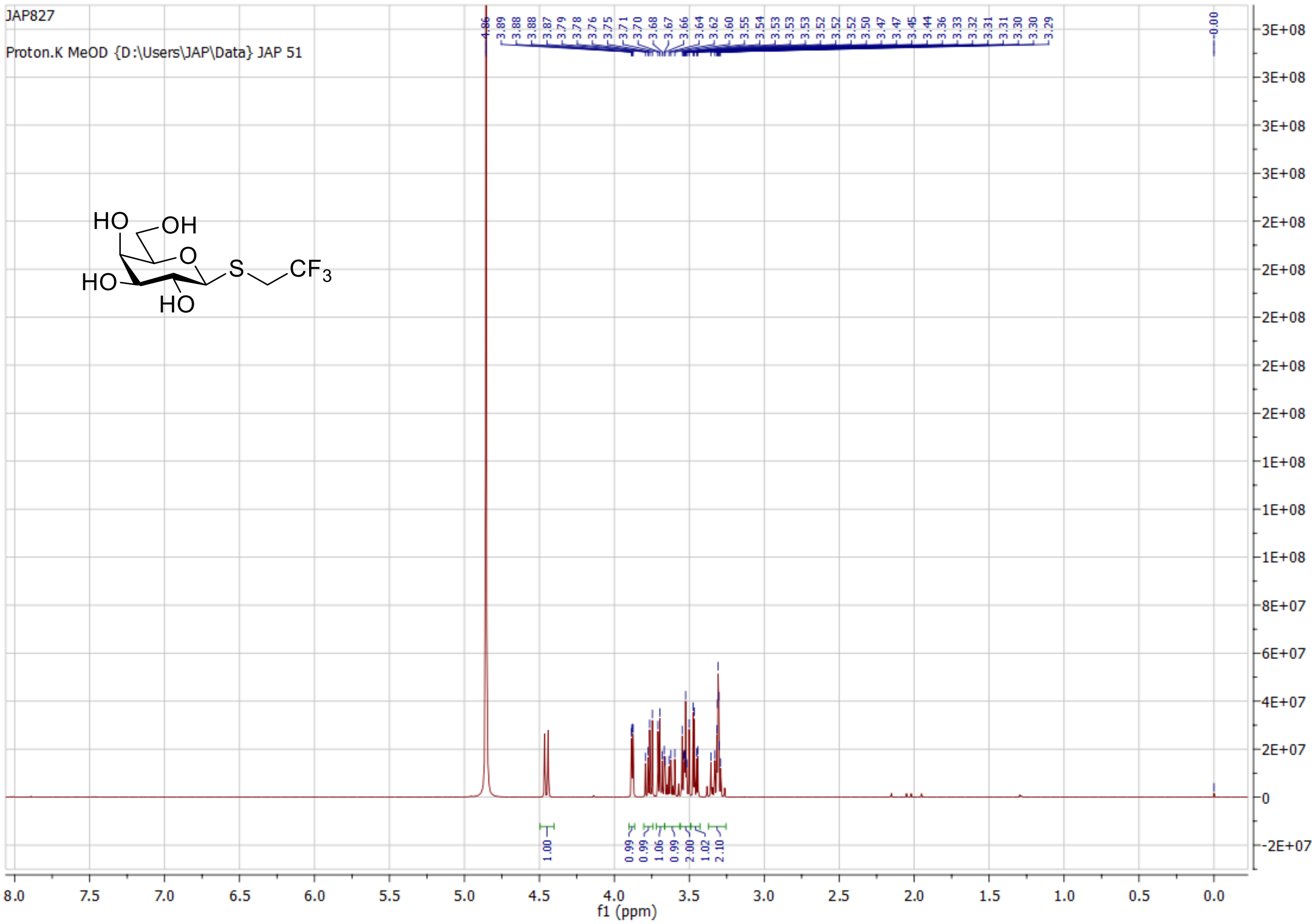

COSY (400 × 400 MHz, MeOD): Trifluoroethyl 1-thio-β-D-galactopyranoside 45

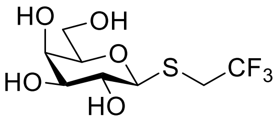

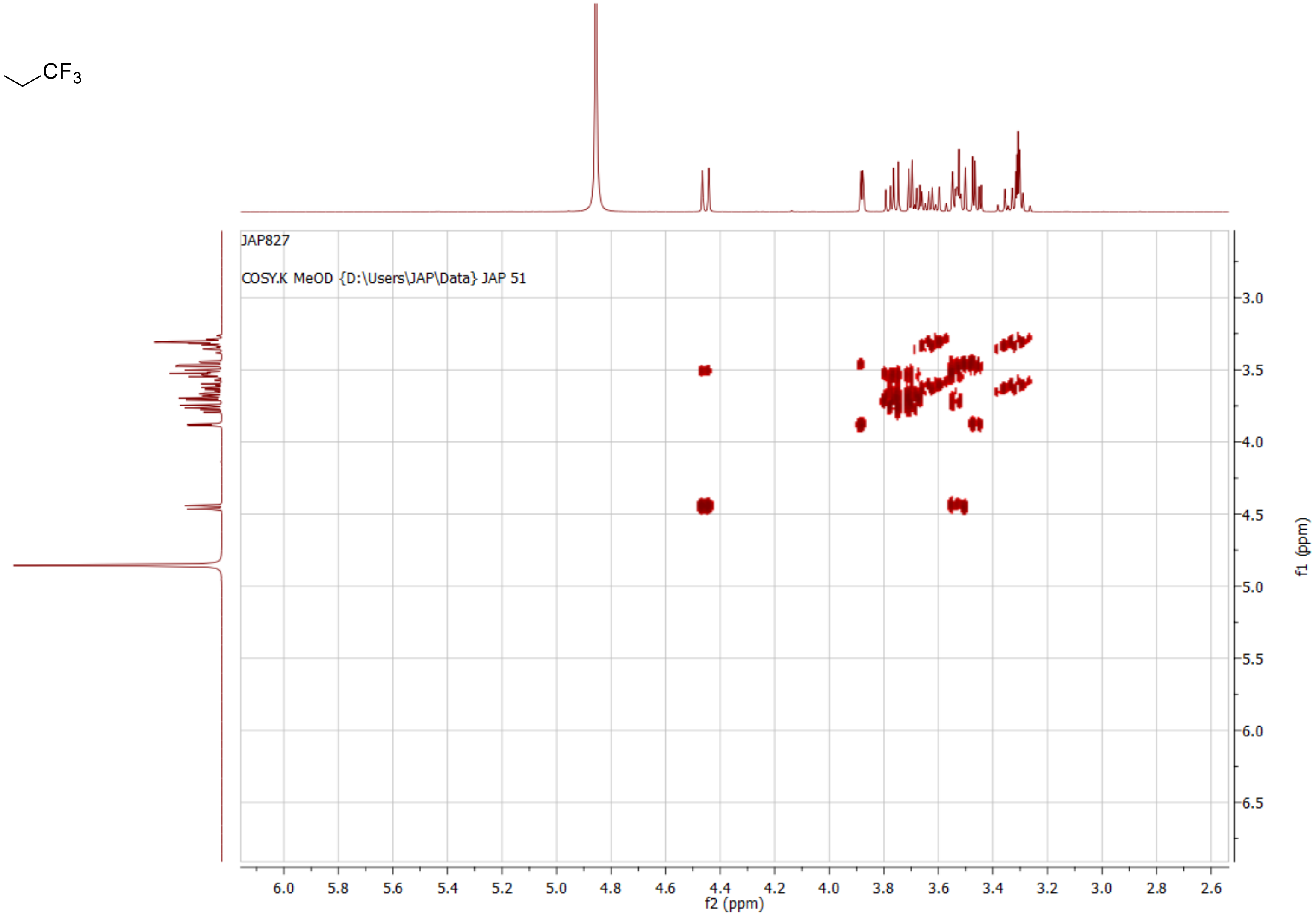

HSQC (400 × 101 MHz, MeOD): Trifluoroethyl 1-thio-β-D-galactopyranoside 45

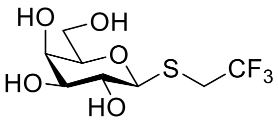

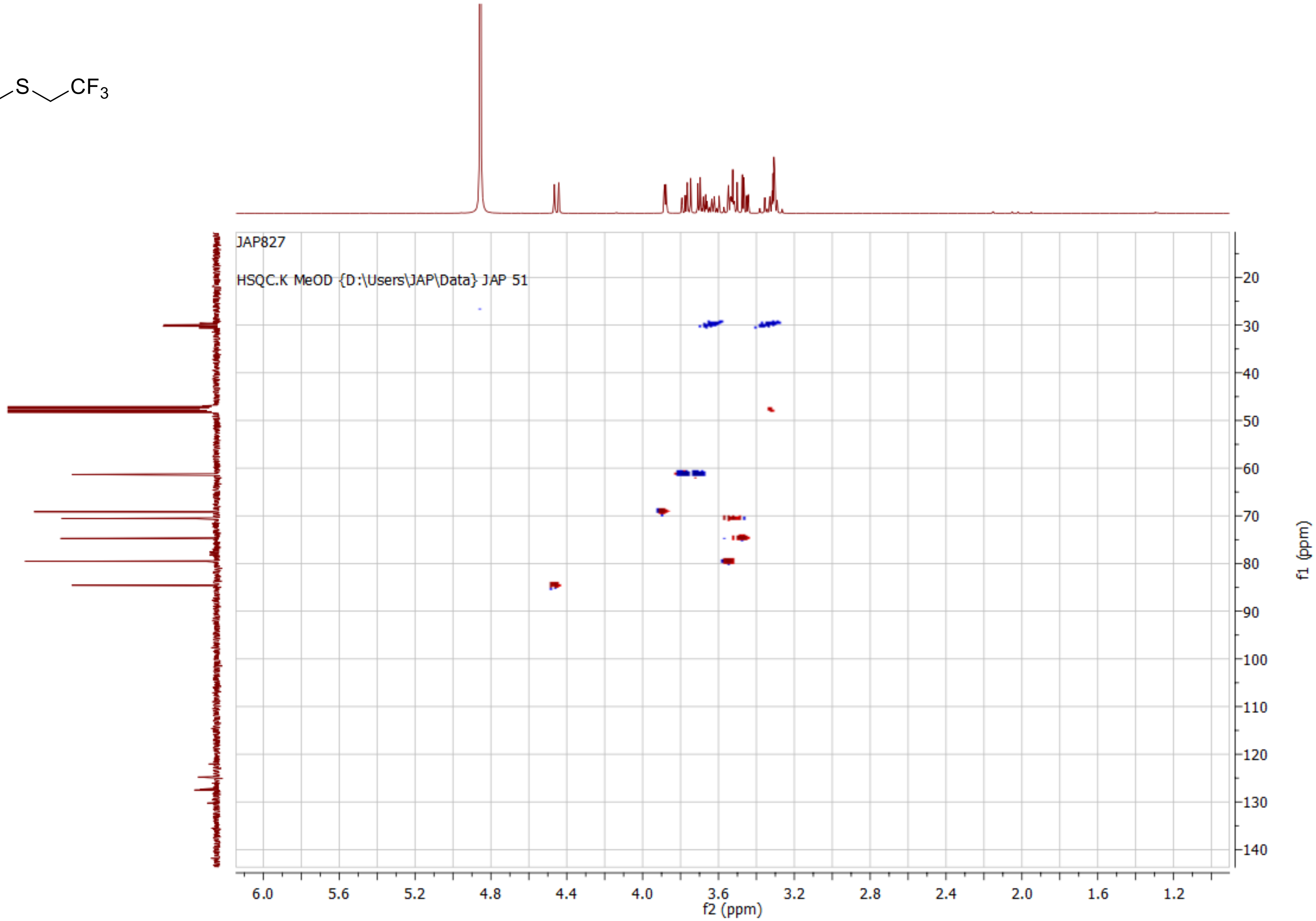

$^{13}C\{^1H\}$ NMR (101 MHz, MeOD): Trifluoroethyl 1-thio-β-D-galactopyranoside 45

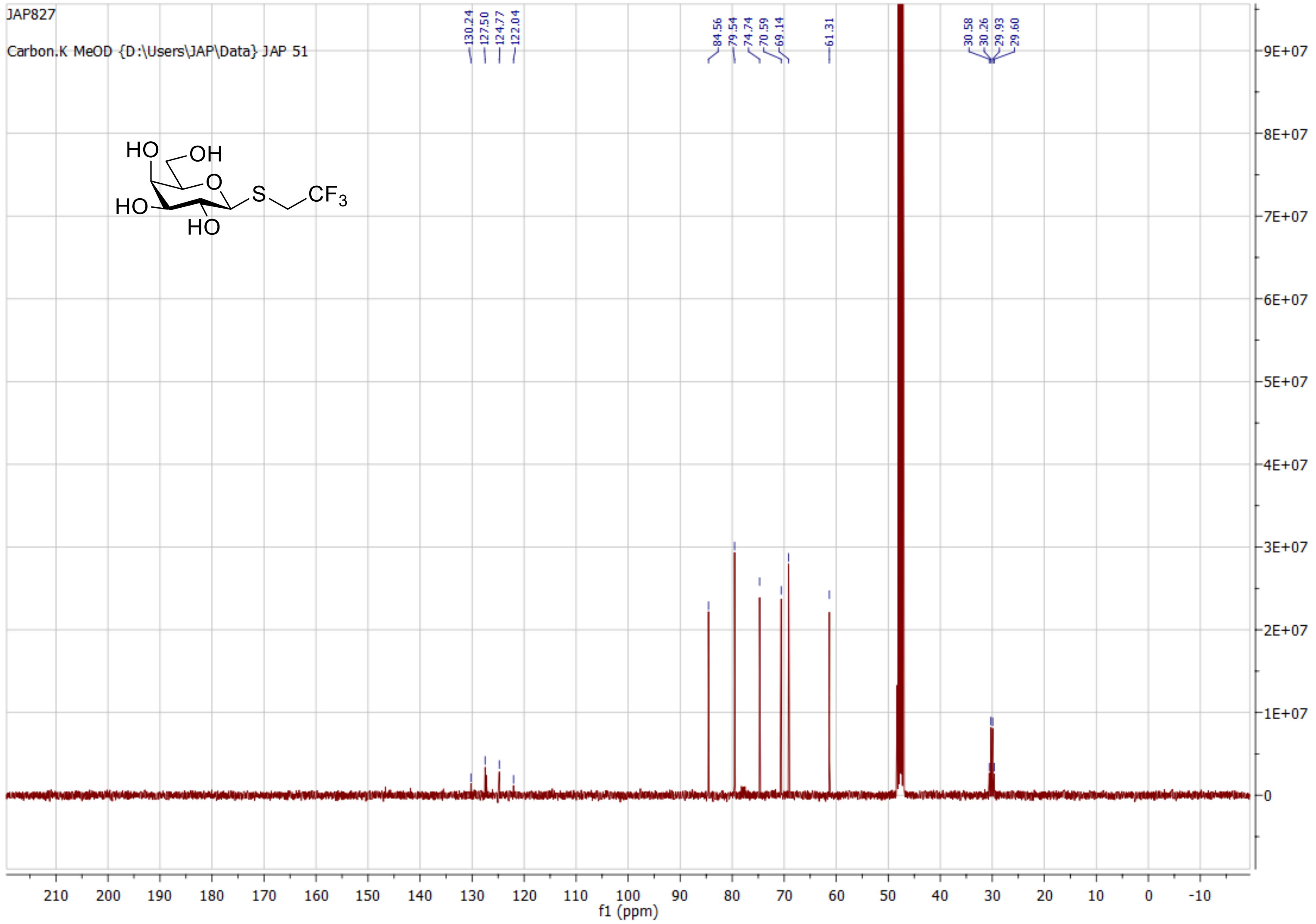

^{19}F NMR (376 MHz, MeOD): Trifluoroethyl 1-thio-β-D-galactopyranoside 45

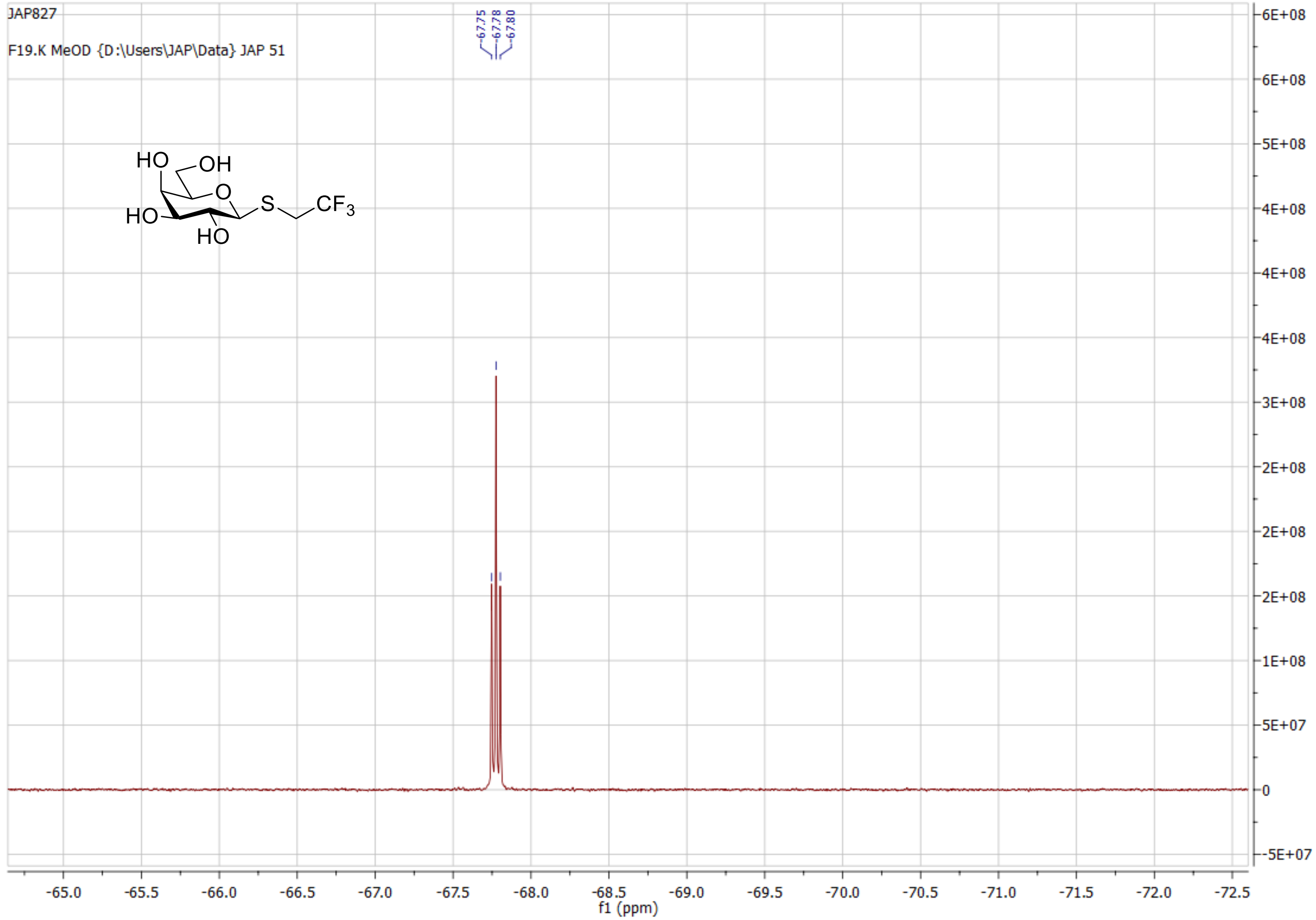

Compound S8

^{1}H NMR (400 MHz, $CDCl_3$): Trifluoroethyl 2,3,4,6-tetra-*O*-acetyl-β-D-galactopyranoside S8

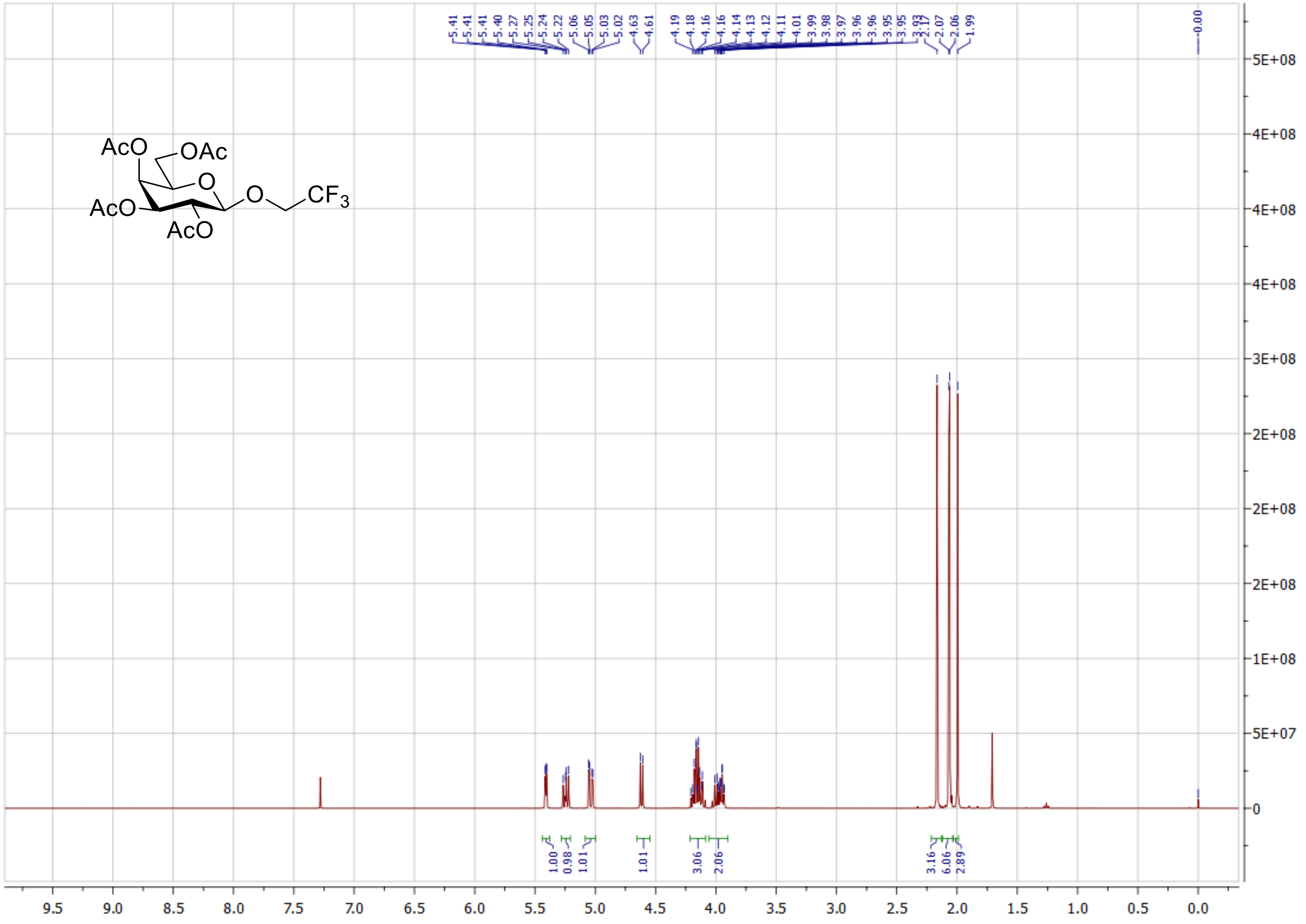

COSY (400 × 400 MHz, $CDCl_3$): Trifluoroethyl 2,3,4,6-tetra-*O*-acetyl-β-D-galactopyranoside S8

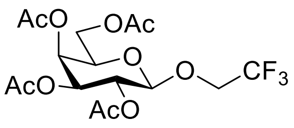

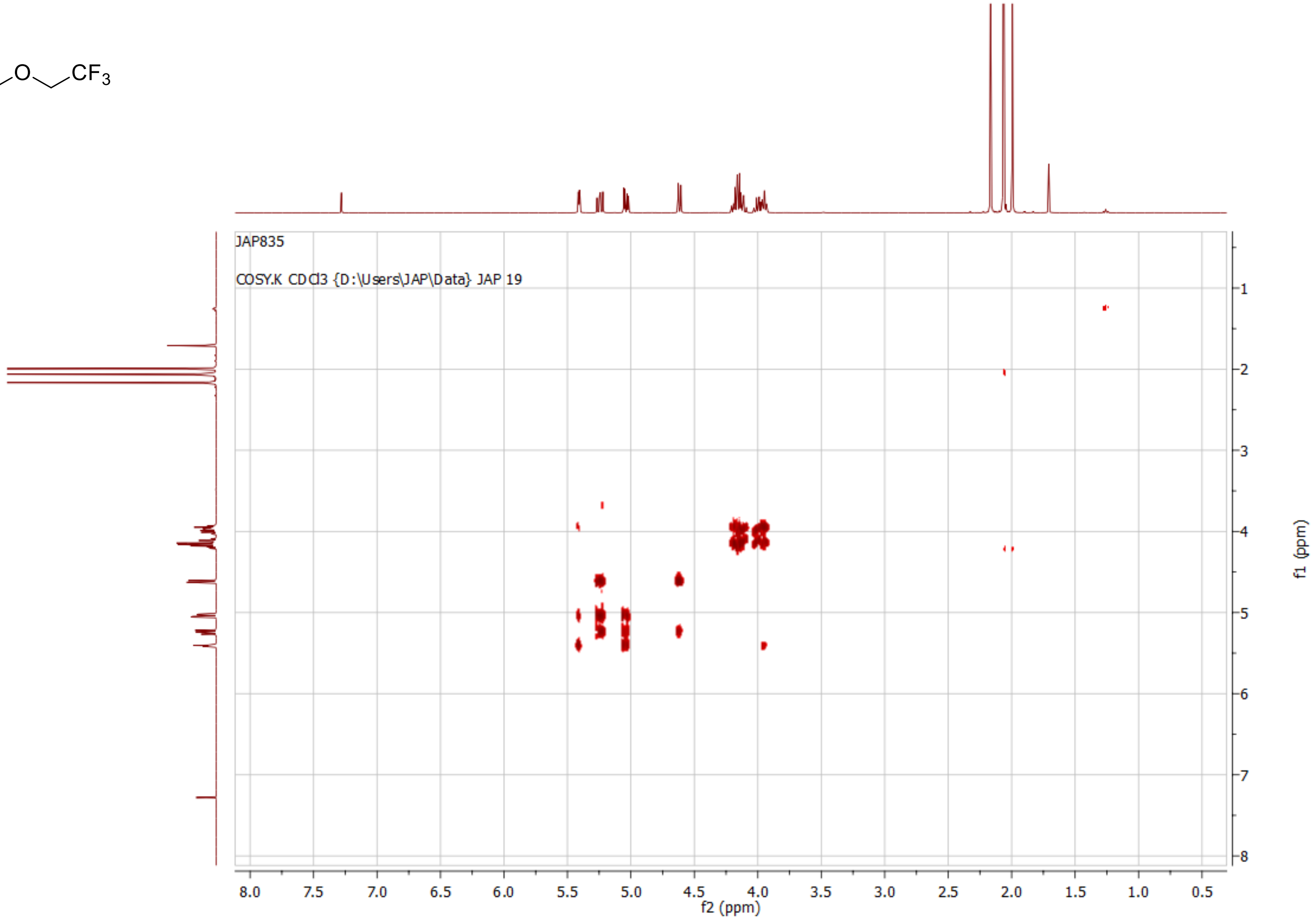

HSQC (400 × 101 MHz, $CDCl_3$): Trifluoroethyl 2,3,4,6-tetra-*O*-acetyl-β-D-galactopyranoside S8

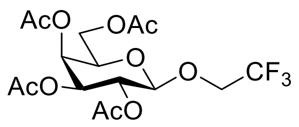

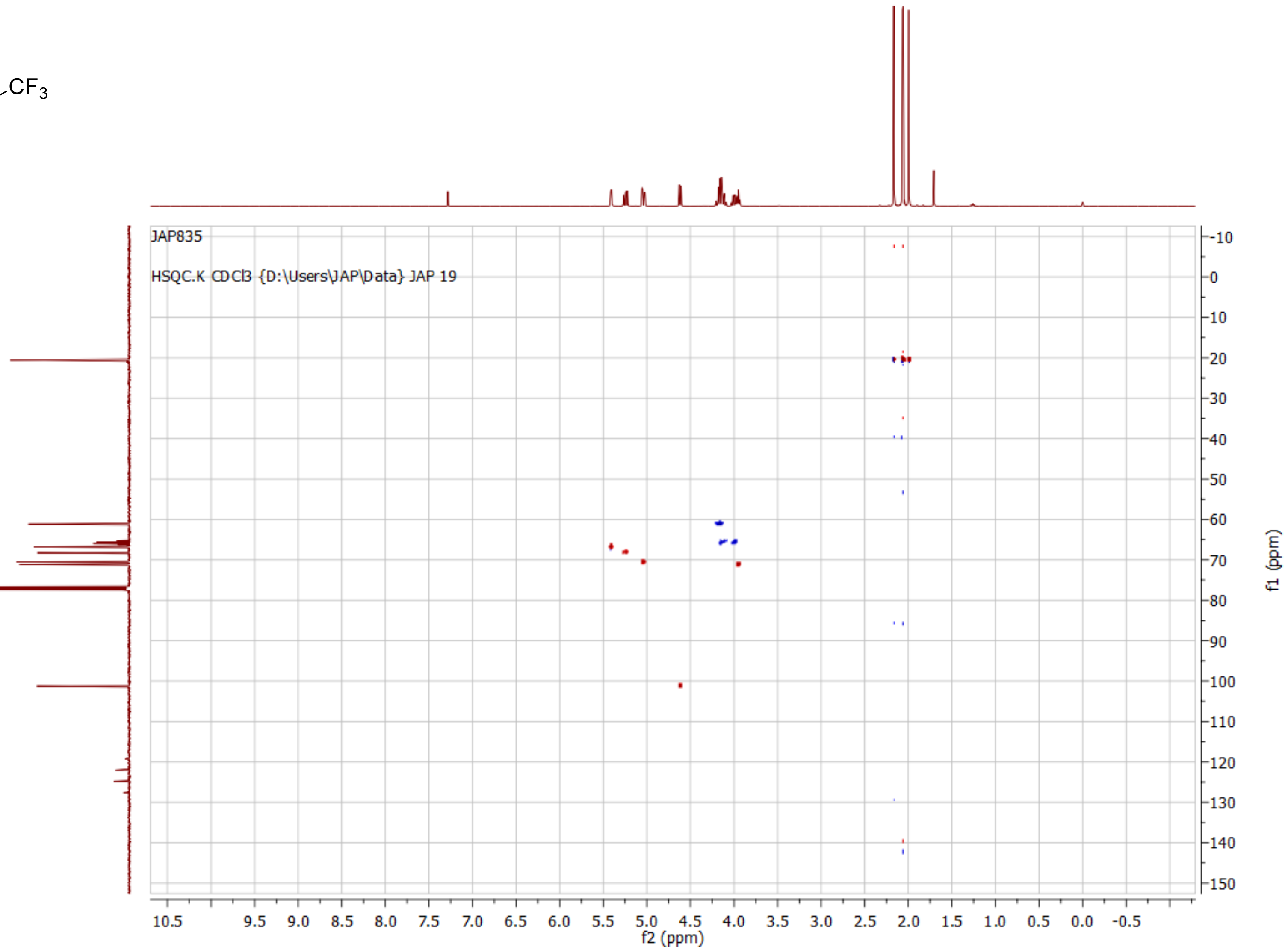

^{13}C{1H} NMR (101 MHz, CDCl₃): Trifluoroethyl 2,3,4,6-tetra-*O*-acetyl-β-D-galactopyranoside S8

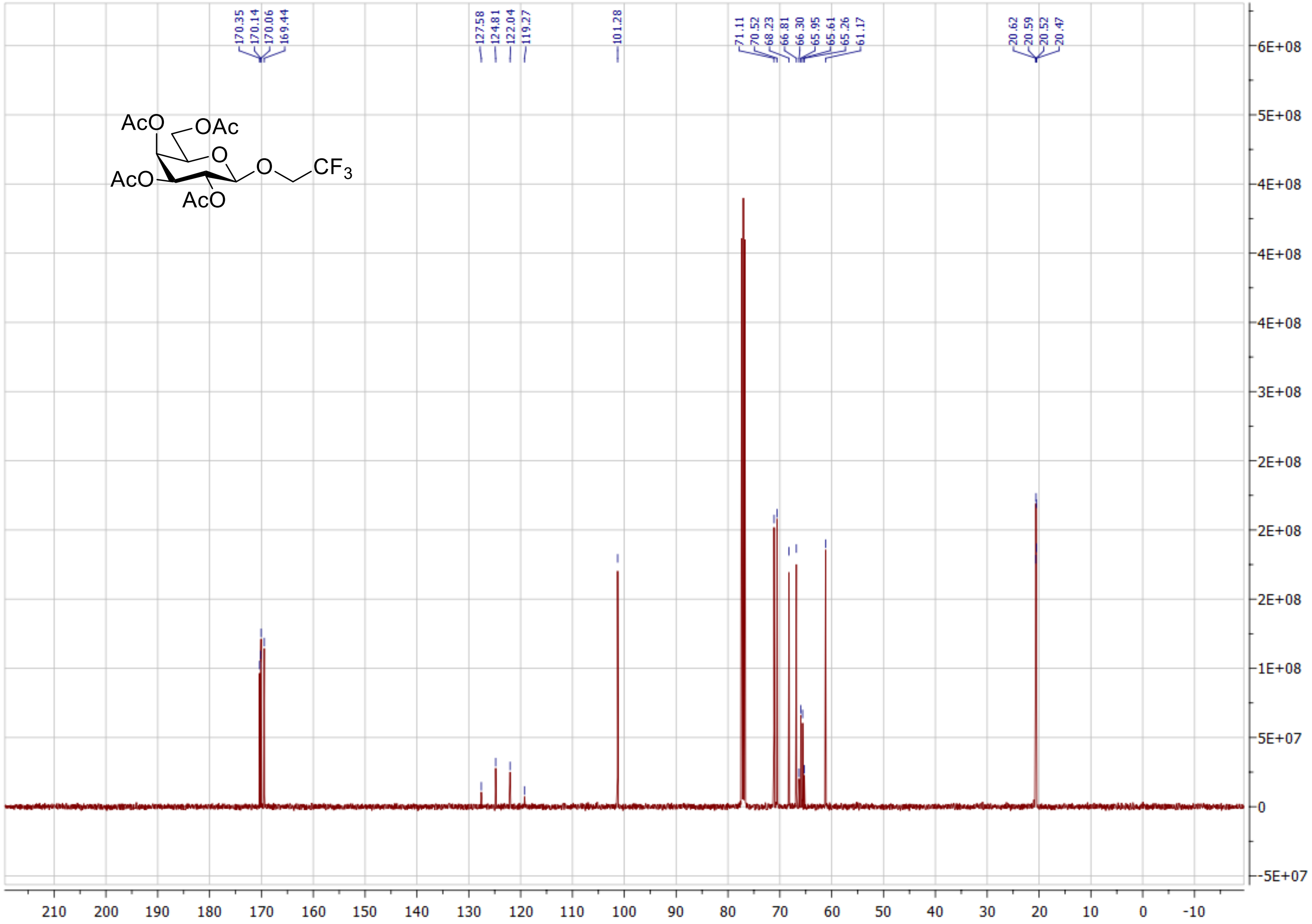

^{19}F NMR (376 MHz, CDCl₃): Trifluoroethyl 2,3,4,6-tetra-*O*-acetyl-β-D-galactopyranoside S8

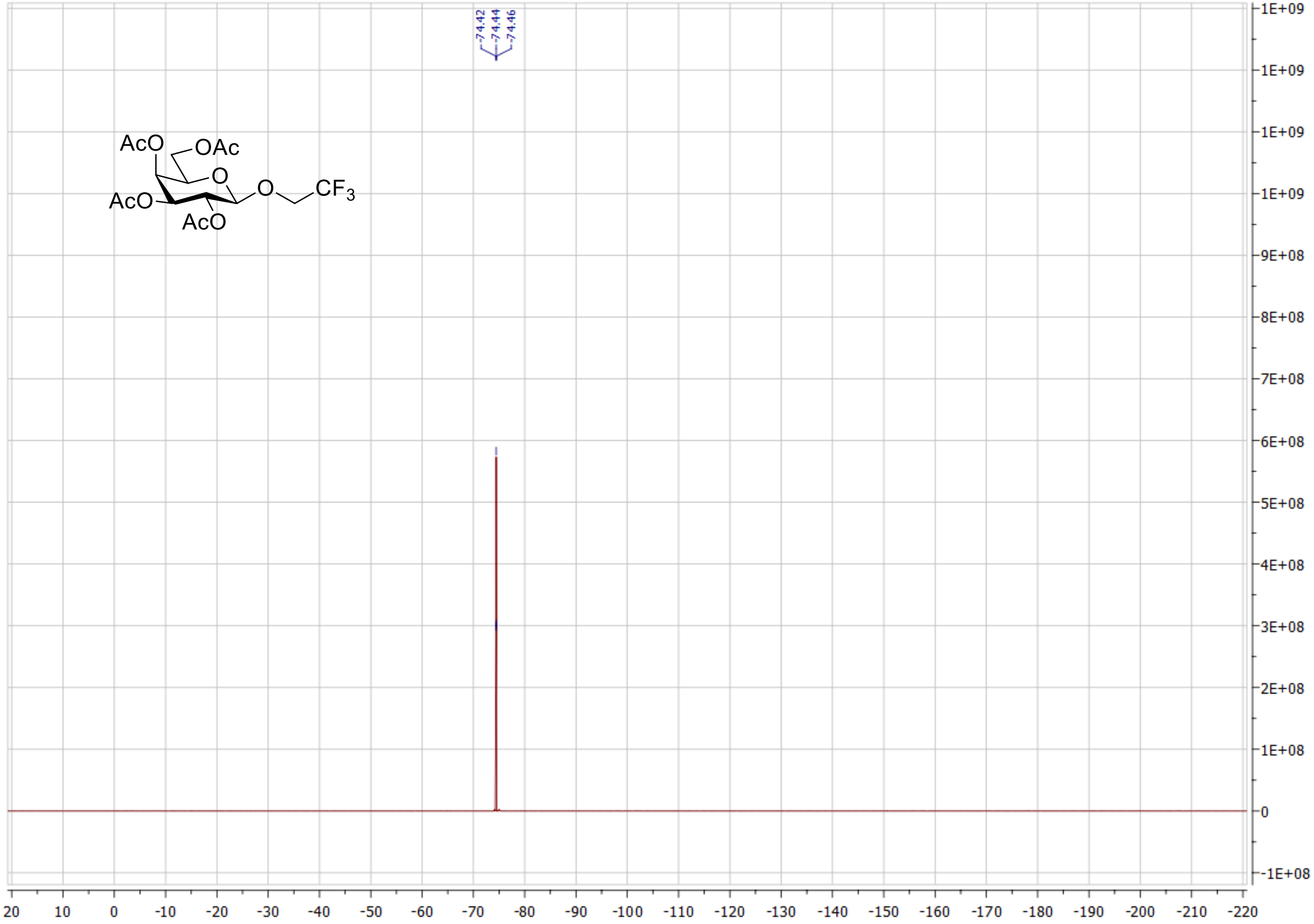

Compound 54

^{1}H NMR (400 MHz, MeOD): Trifluoroethyl β-D-galactopyranoside 54

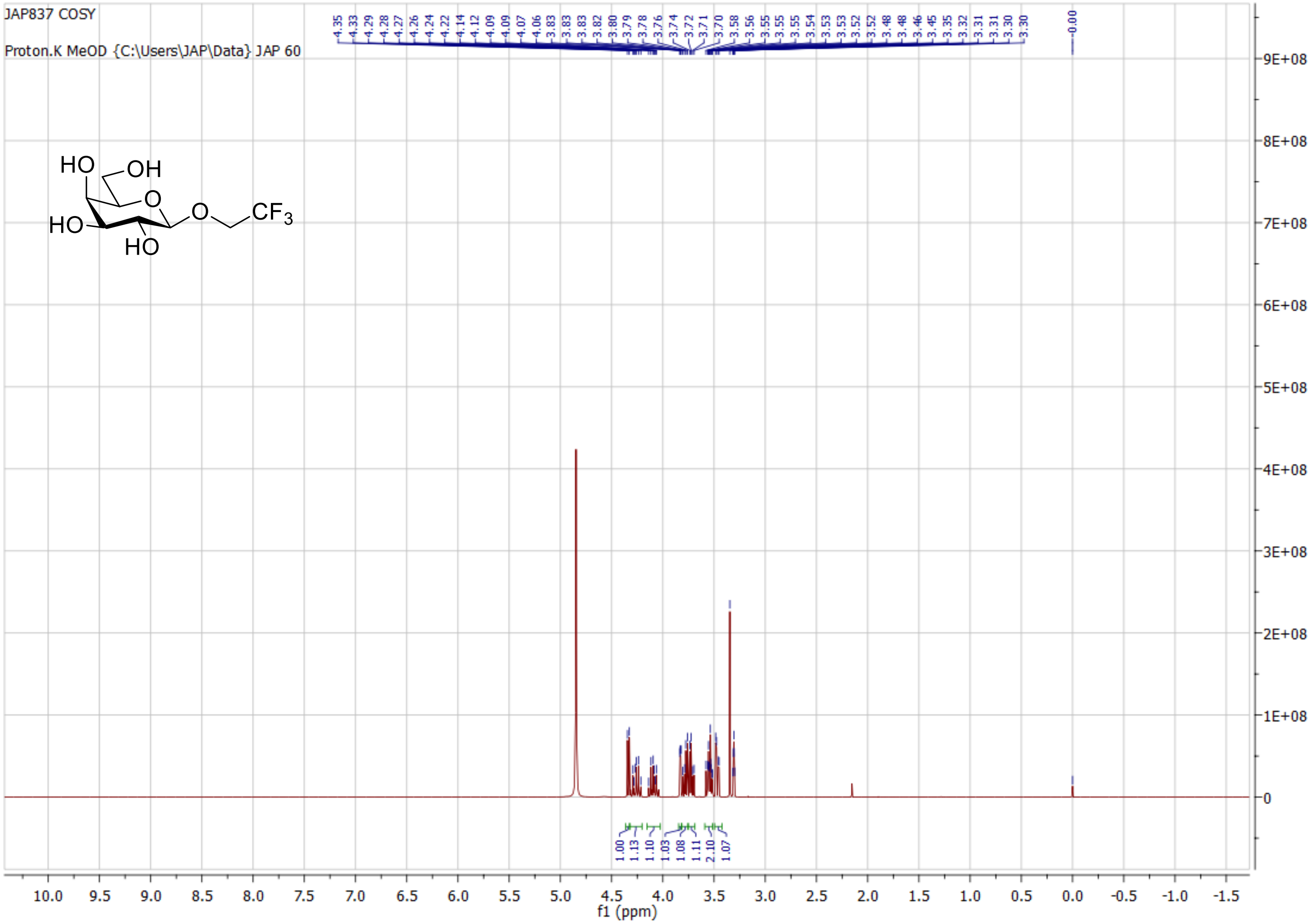

COSY (400 × 400 MHz, MeOD): Trifluoroethyl β-D-galactopyranoside 54

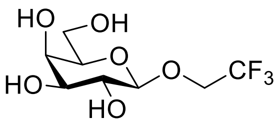

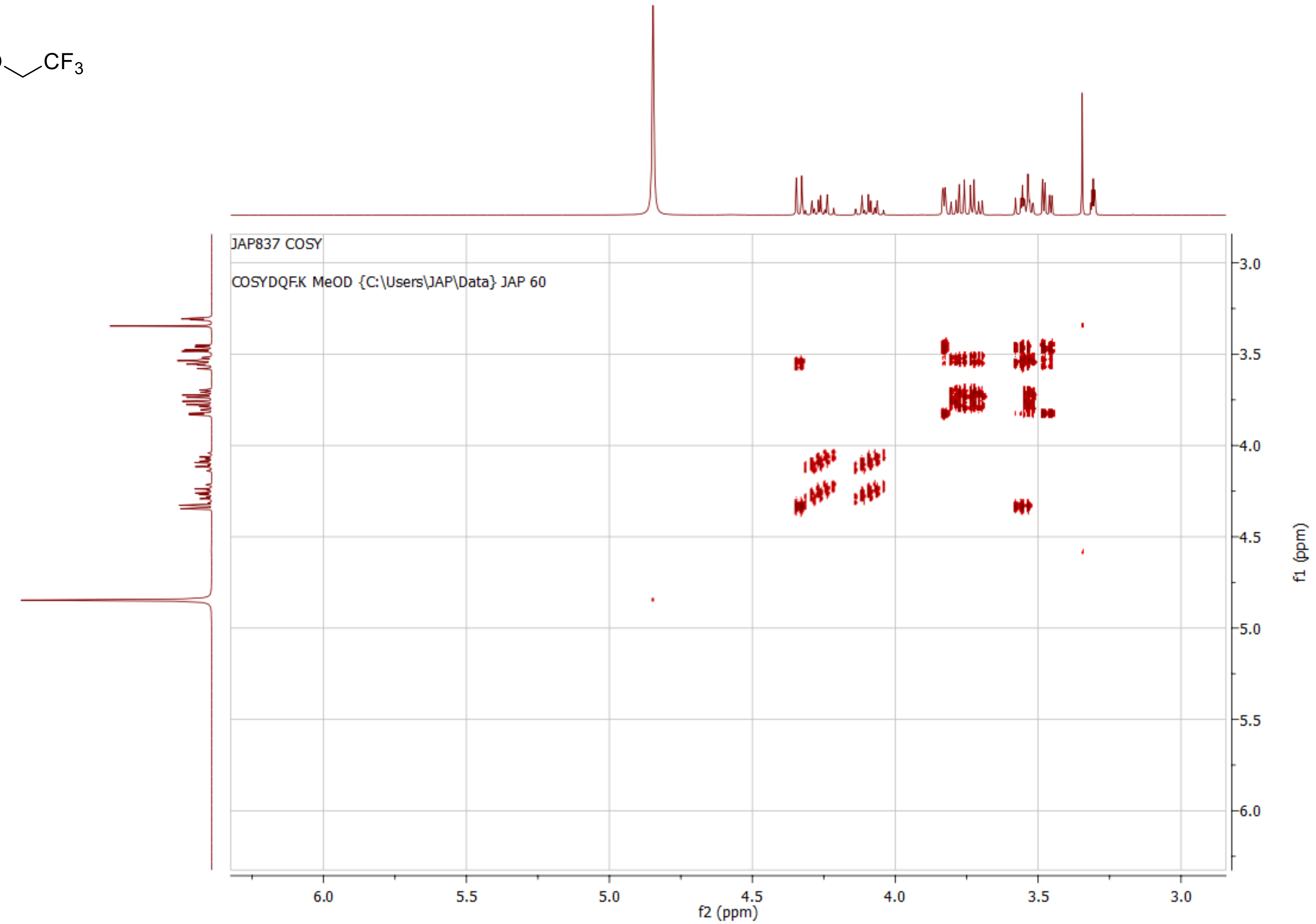

HSQC (400 × 101 MHz, MeOD): Trifluoroethyl β-D-galactopyranoside 54

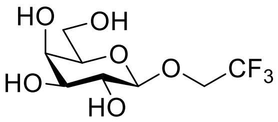

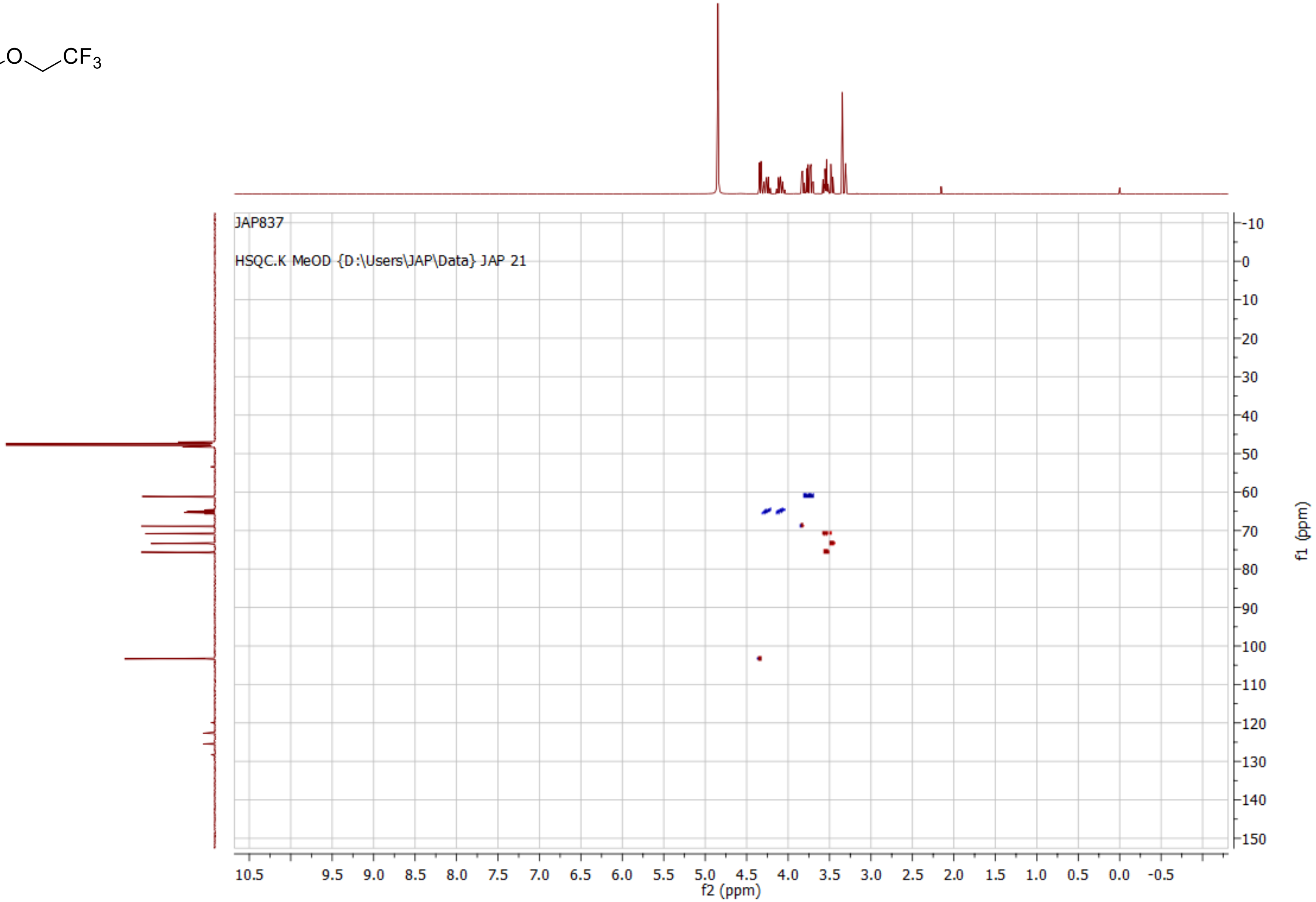

$^{13}C\{1H\}$ NMR (101 MHz, $CDCl_3$): Trifluoroethyl β-D-galactopyranoside 54

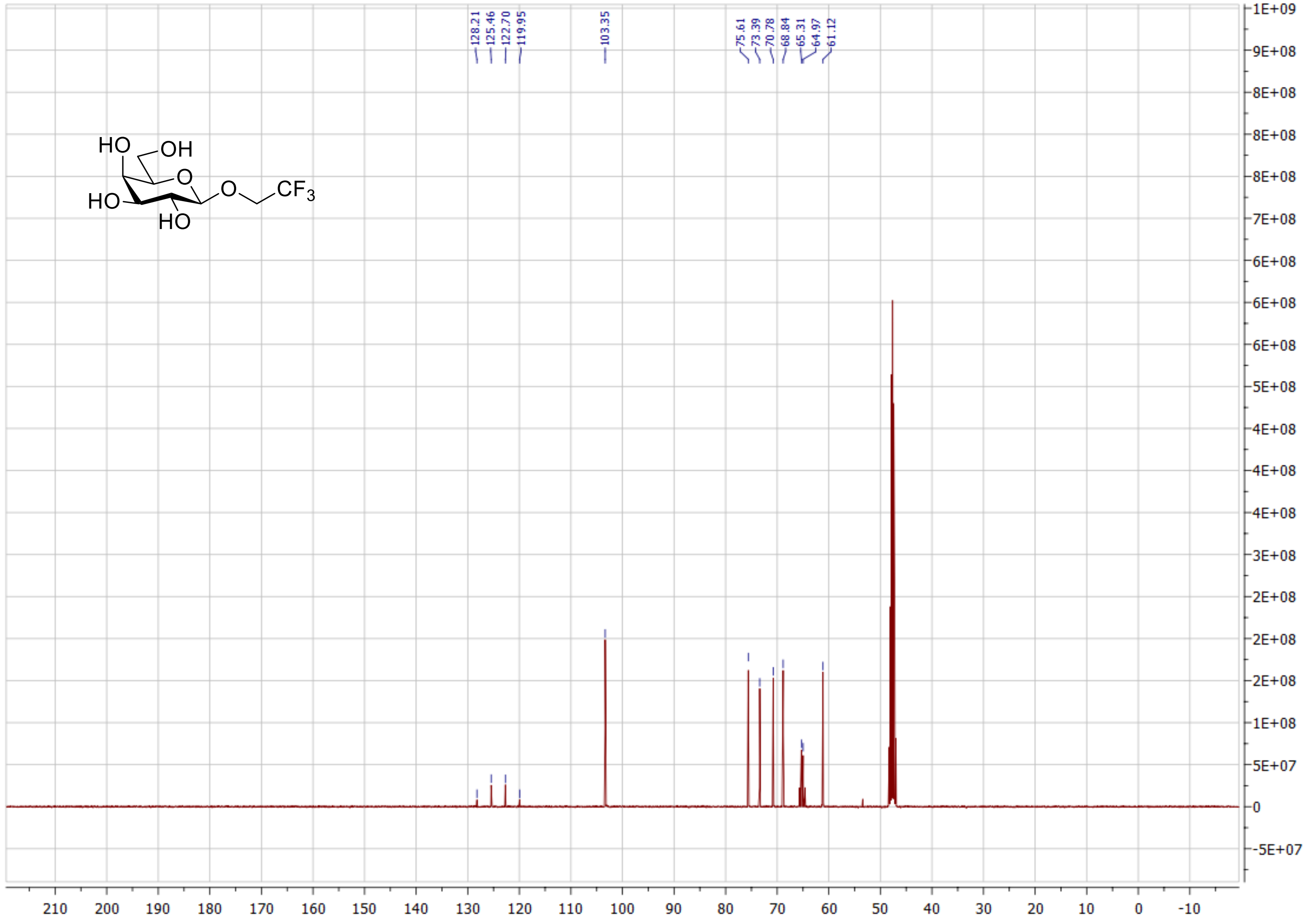

^{19}F NMR (376 MHz, $CDCl_3$): Trifluoroethyl β-D-galactopyranoside 54

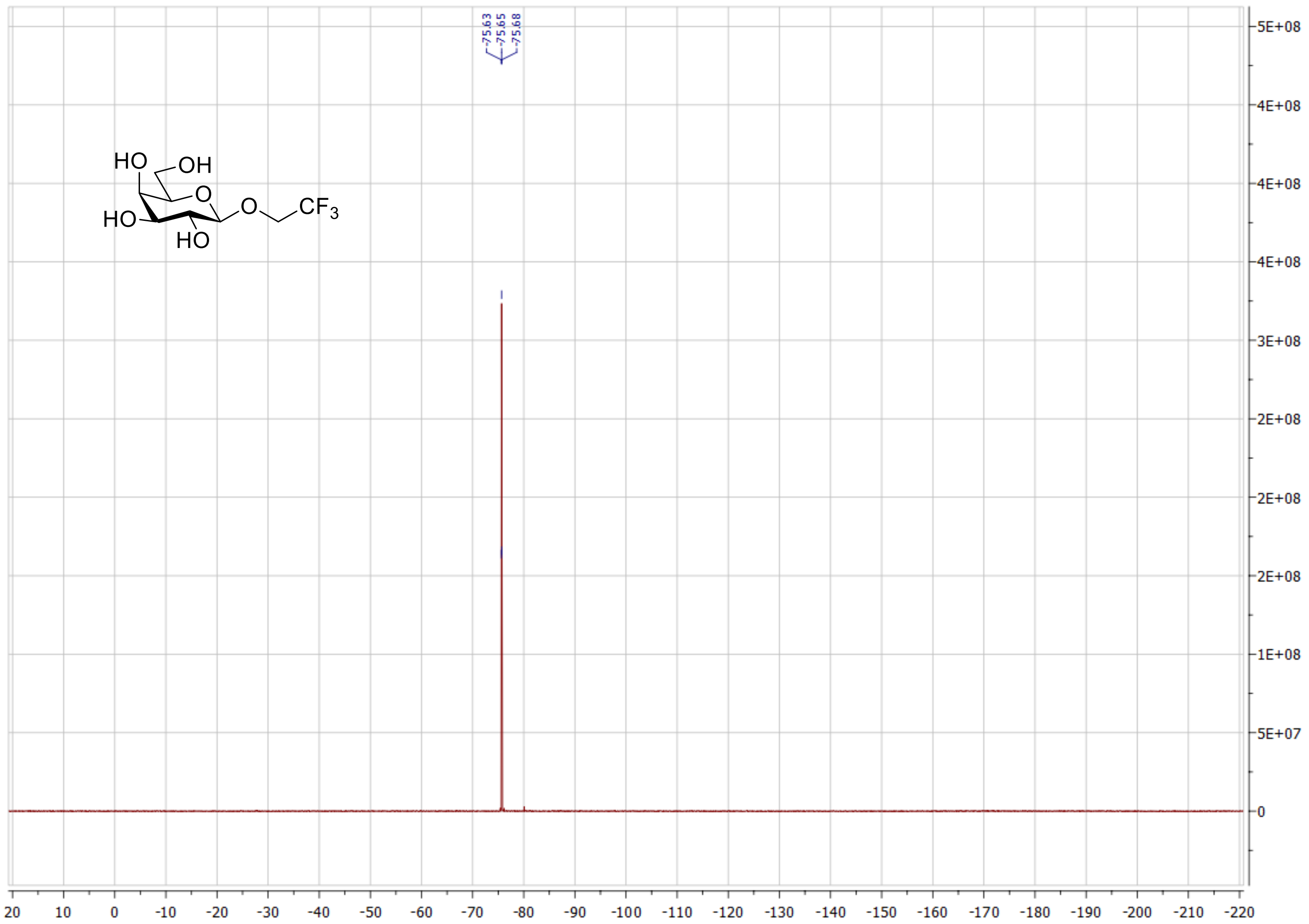

Compound 49

^{1}H NMR (400 MHz, MeOD): Cyclohexyl β-D-galactopyranoside 49

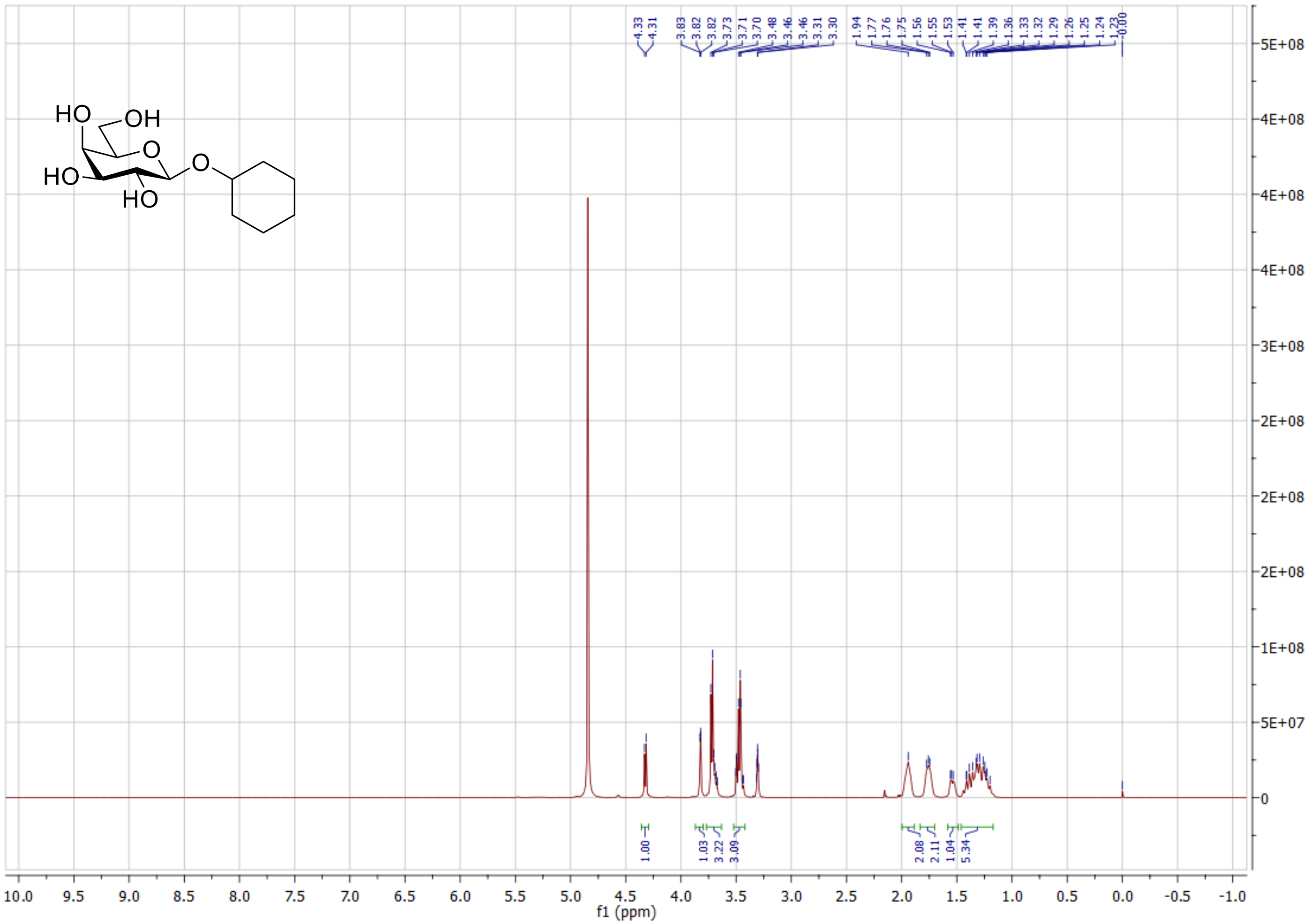

^{13}C{1H} NMR (101 MHz, MeOD): Cyclohexyl β-D-galactopyranoside 49

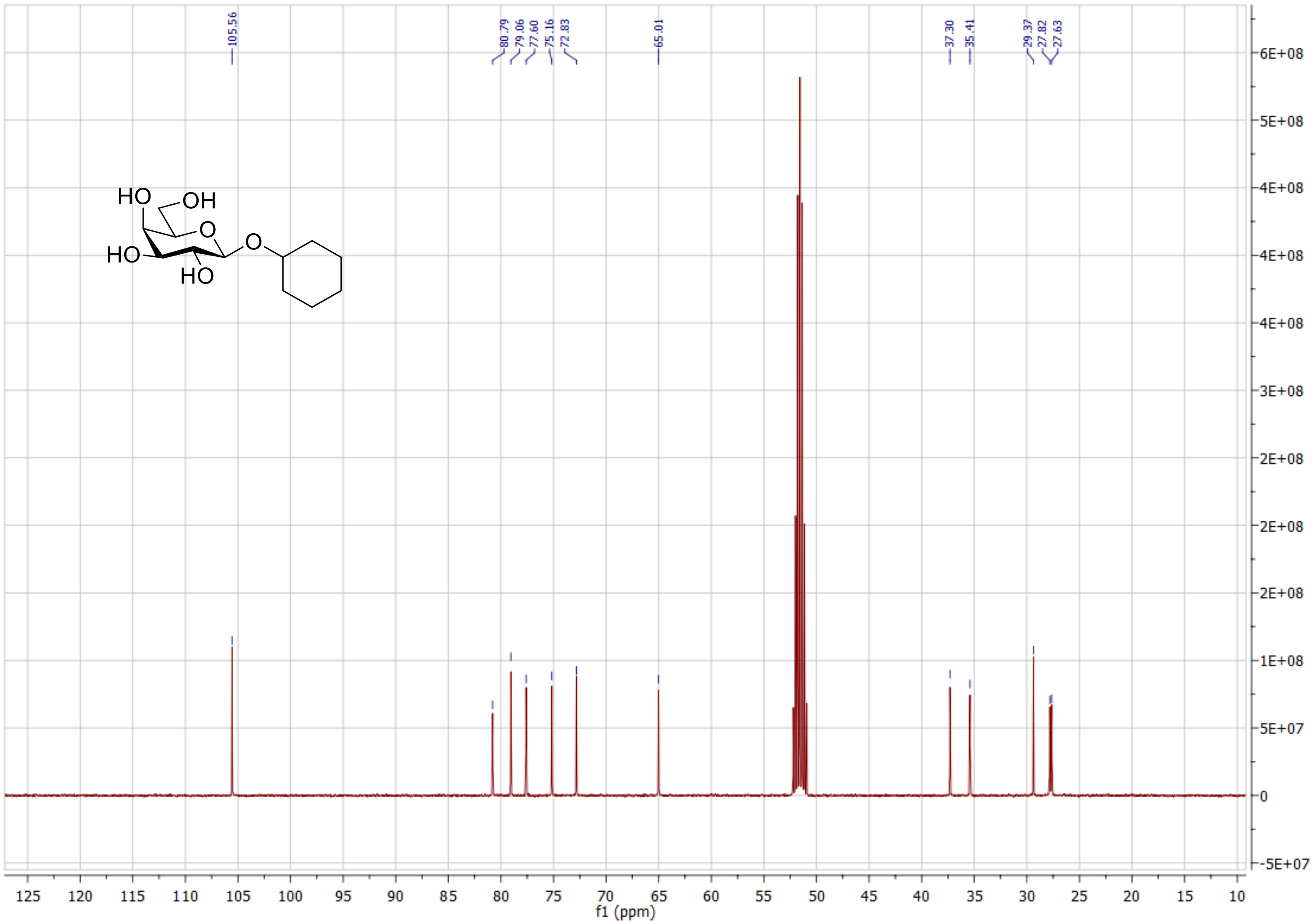

Compound 13

^{1}H NMR (400 MHz, MeOD): ***p*-Methoxy phenyl α-D-galactopyranoside 13**

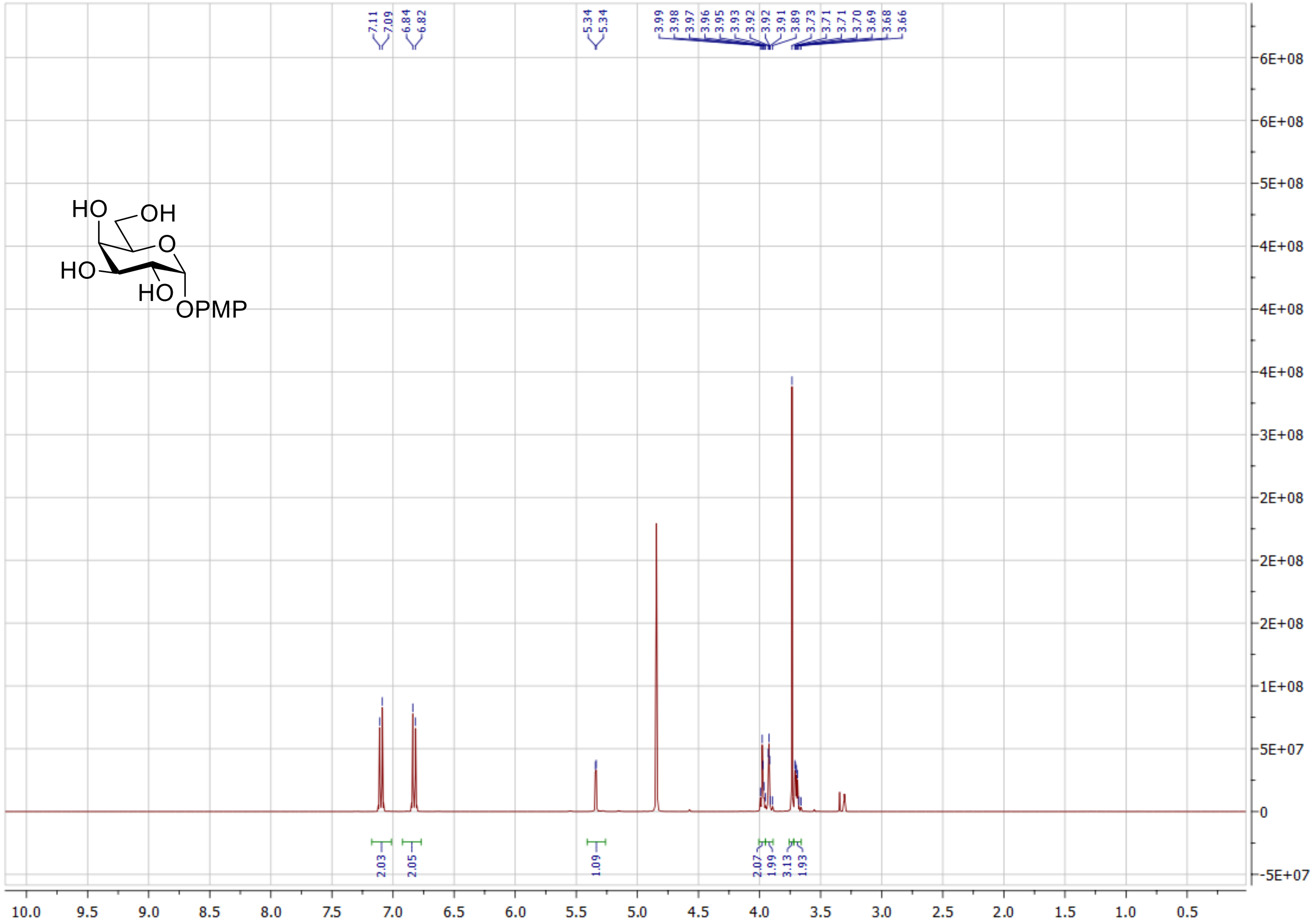

^{13}C{1H} NMR (101 MHz, MeOD): *p*-Methoxy phenyl α-D-galactopyranoside 13

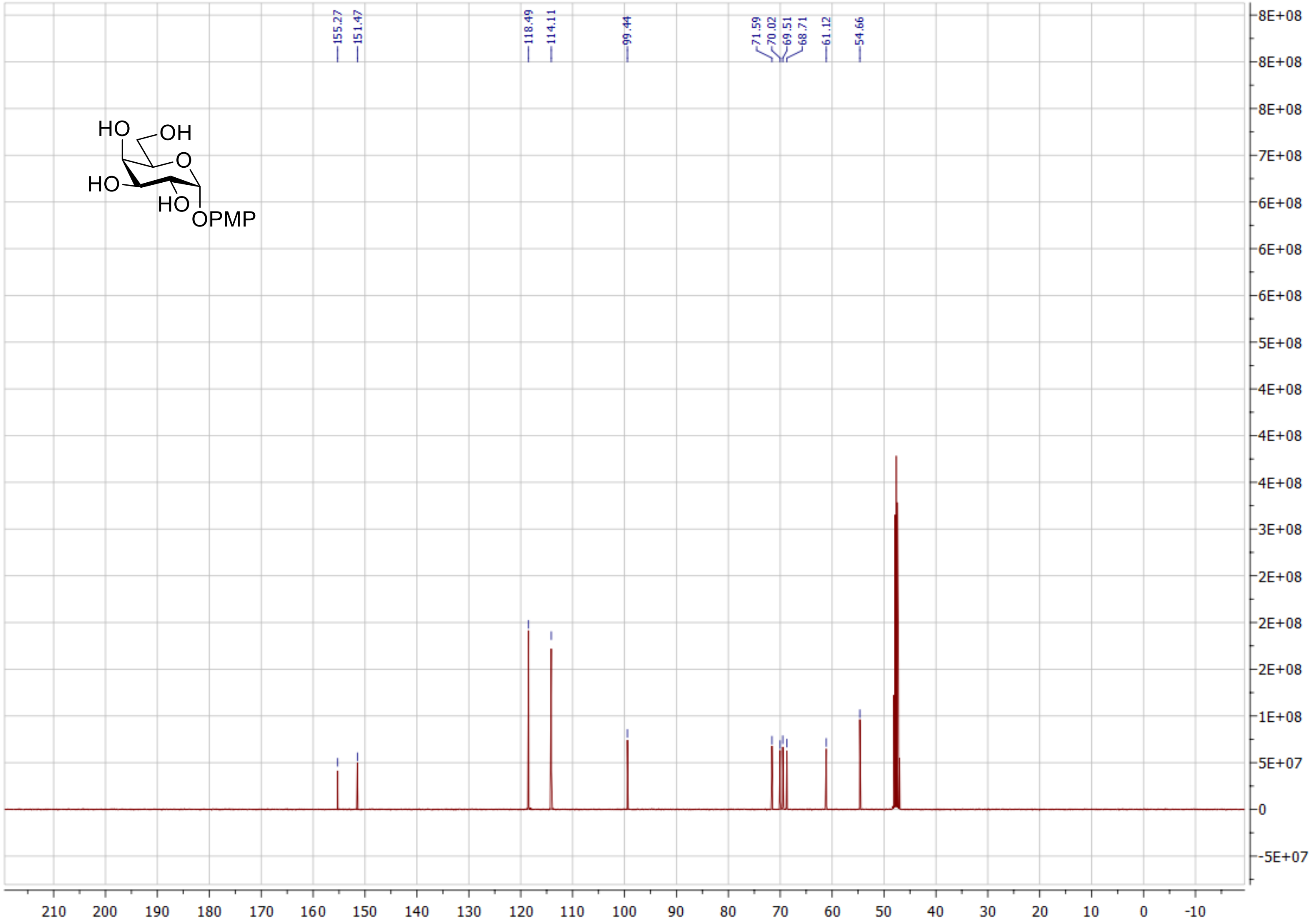

Compound 11

^{1}H NMR (400 MHz, CDCl₃): Methyl 6-*O*-triisopropylsilyl-α-D-galactopyranoside 11

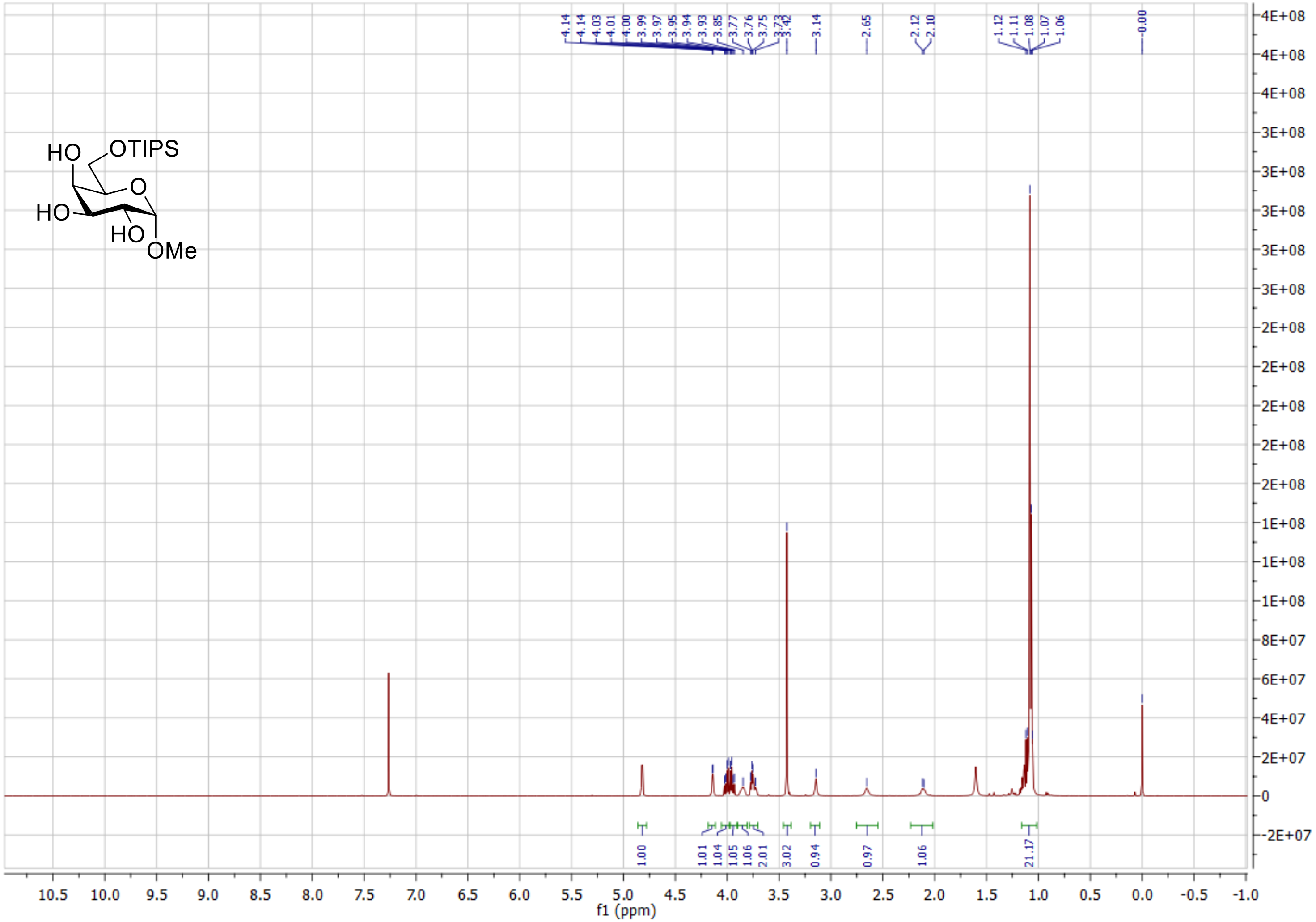

$^{13}C\{1H\}$ NMR (101 MHz, $CDCl_3$): Methyl 6-*O*-triisopropylsilyl-α-D-galactopyranoside 11

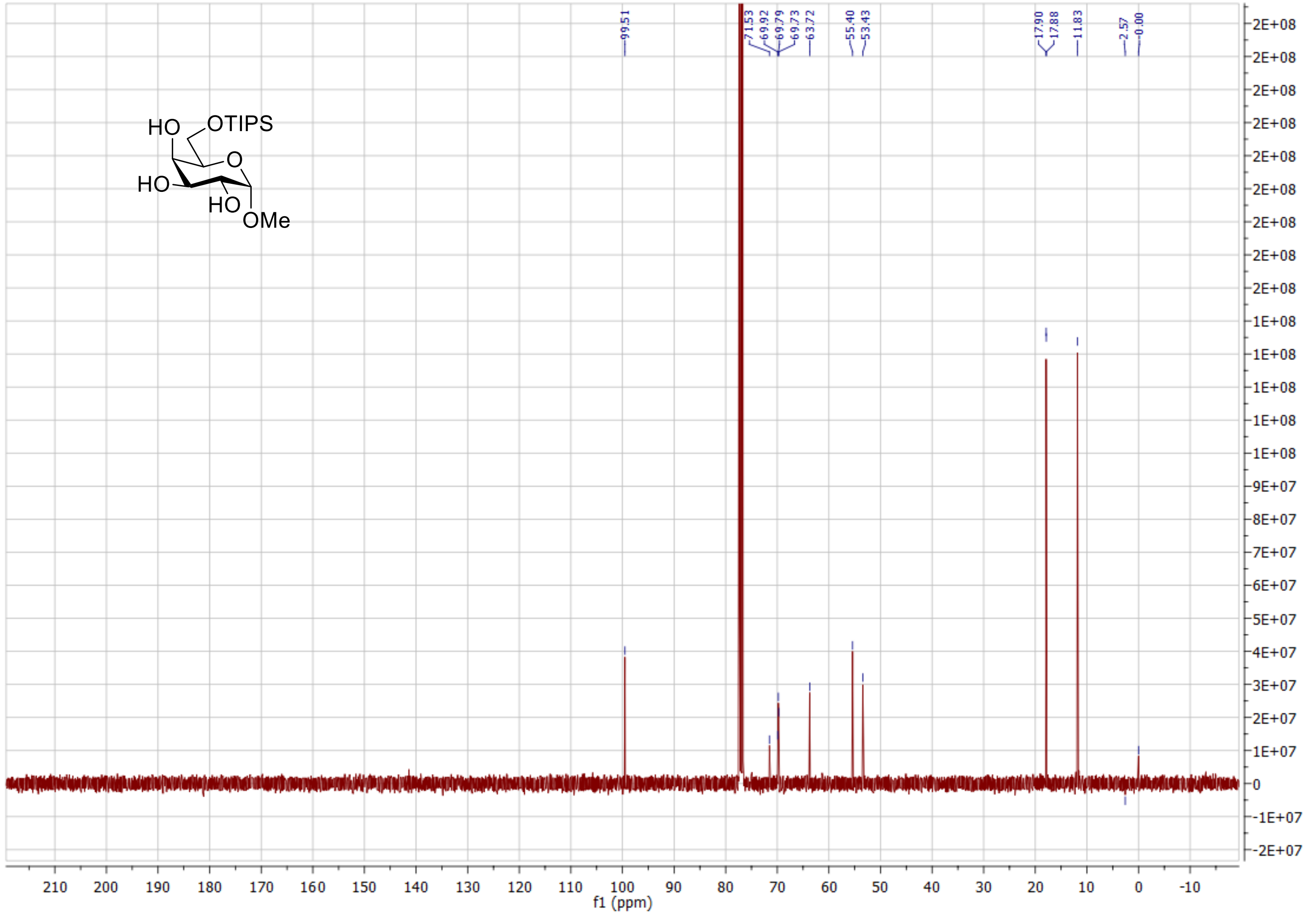

Compound 15

^{1}H NMR (400 MHz MeOD): Allyl 2-acetamido-2-deoxy-β-D-galactopyranoside 15

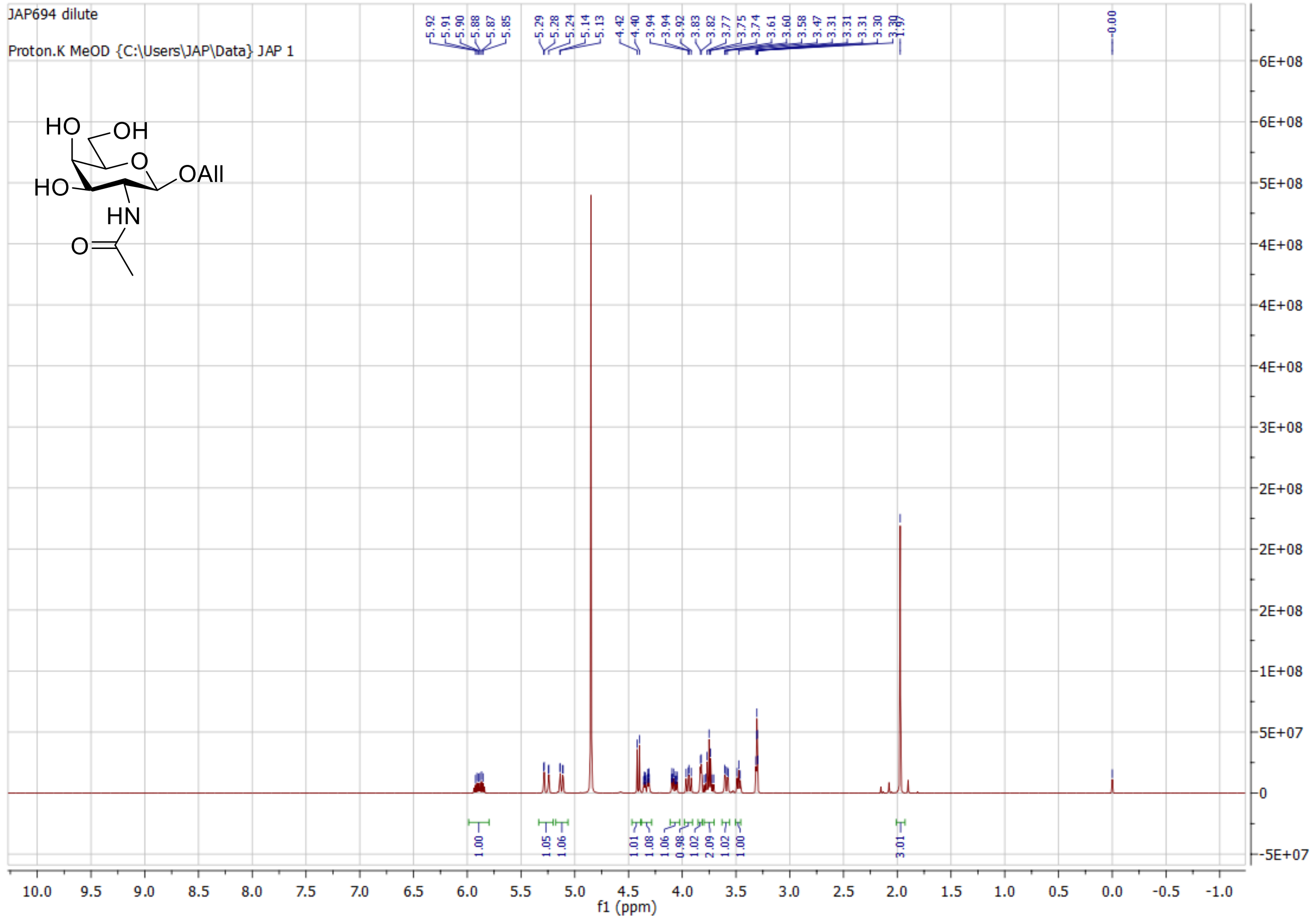

$^{13}C\{1H\}$ NMR (101 MHz MeOD): Allyl 2-acetamido-2-deoxy-β-D-galactopyranoside 15

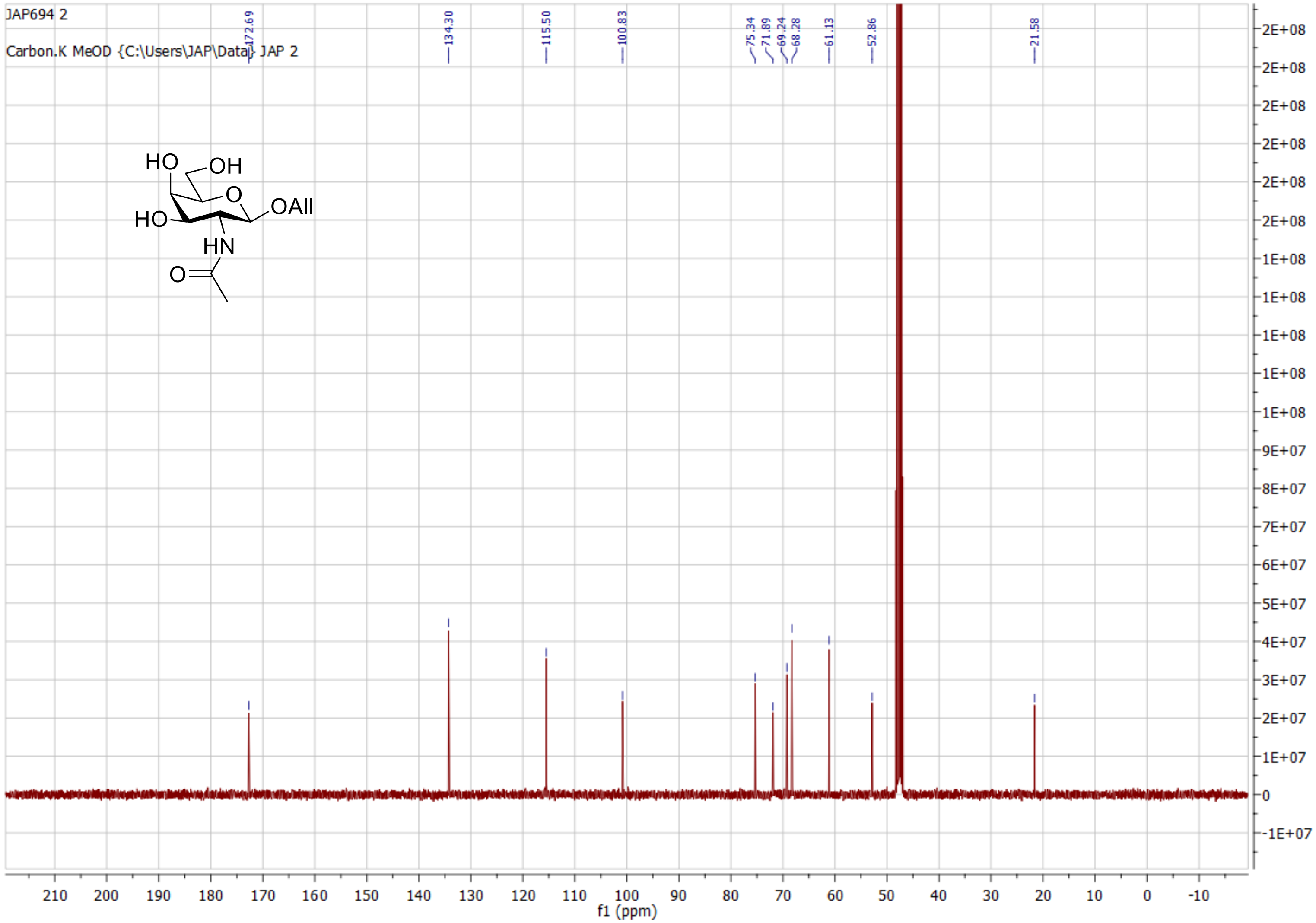

Compound 17

^{1}H NMR (400 MHz, CDCl₃): Allyl 2-deoxy-2-acetamido-α-D-galactopyranoside 17

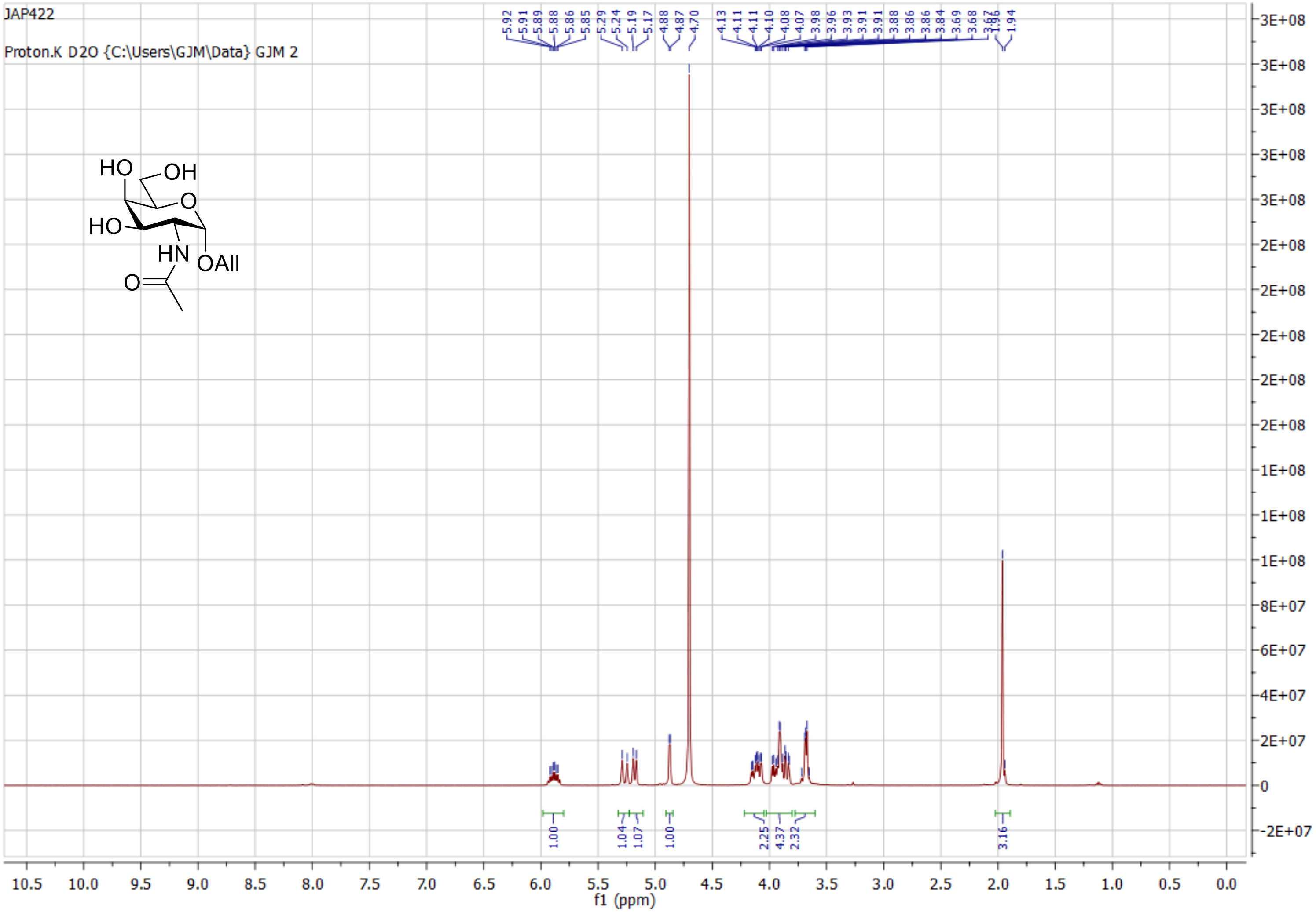

$^{13}C\{1H\}$ NMR (101 MHz, $CDCl_3$): Allyl 2-deoxy-2-acetamido-α-D-galactopyranoside 17

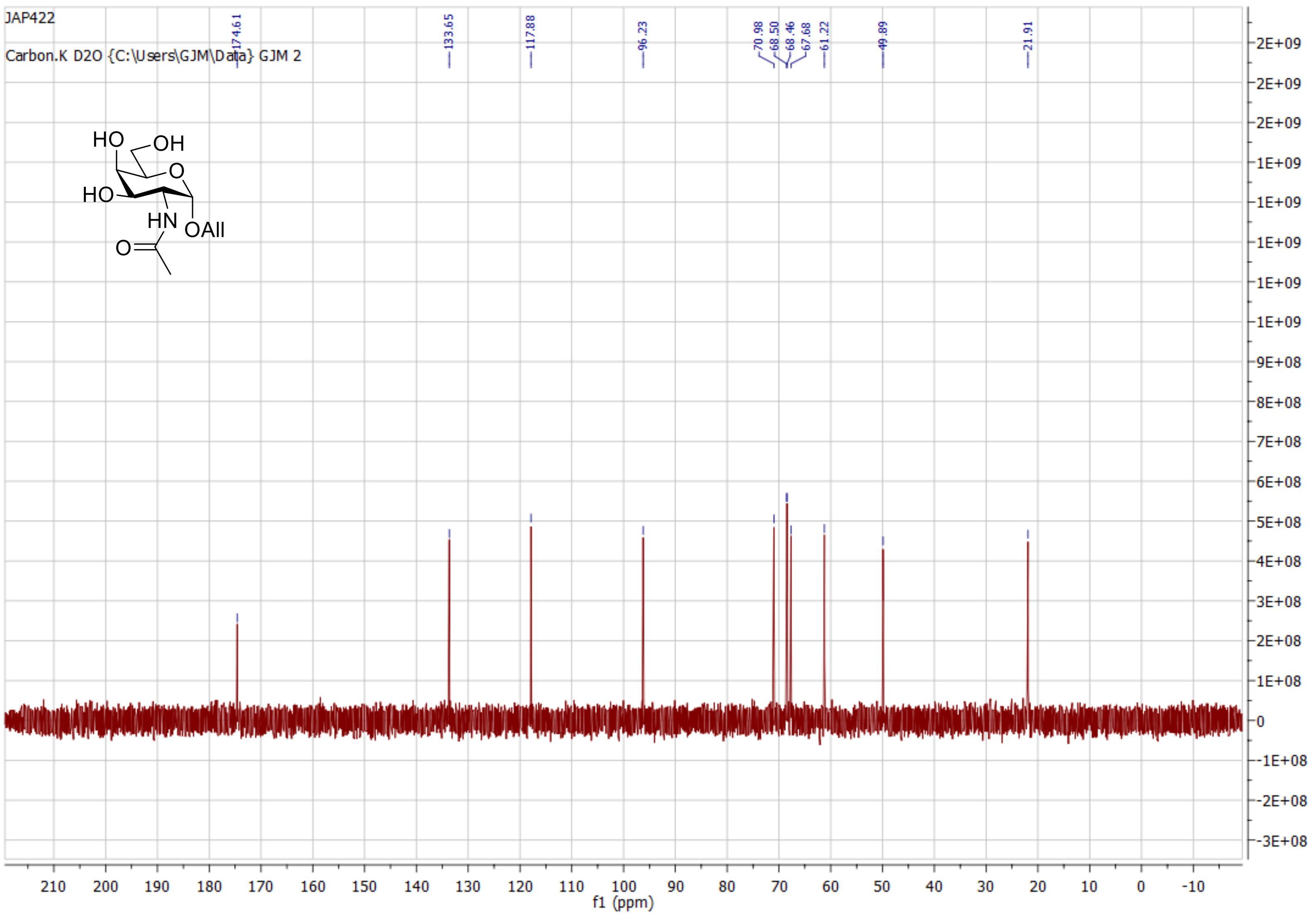

Compound S9

^{1}H NMR (400 MHz, CDCl$_3$): *p*-(Trifluoromethyl)-phenyl 2,3,4,6-tetra-*O*-acetyl-1-thio-β-D-galactopyranoside S9

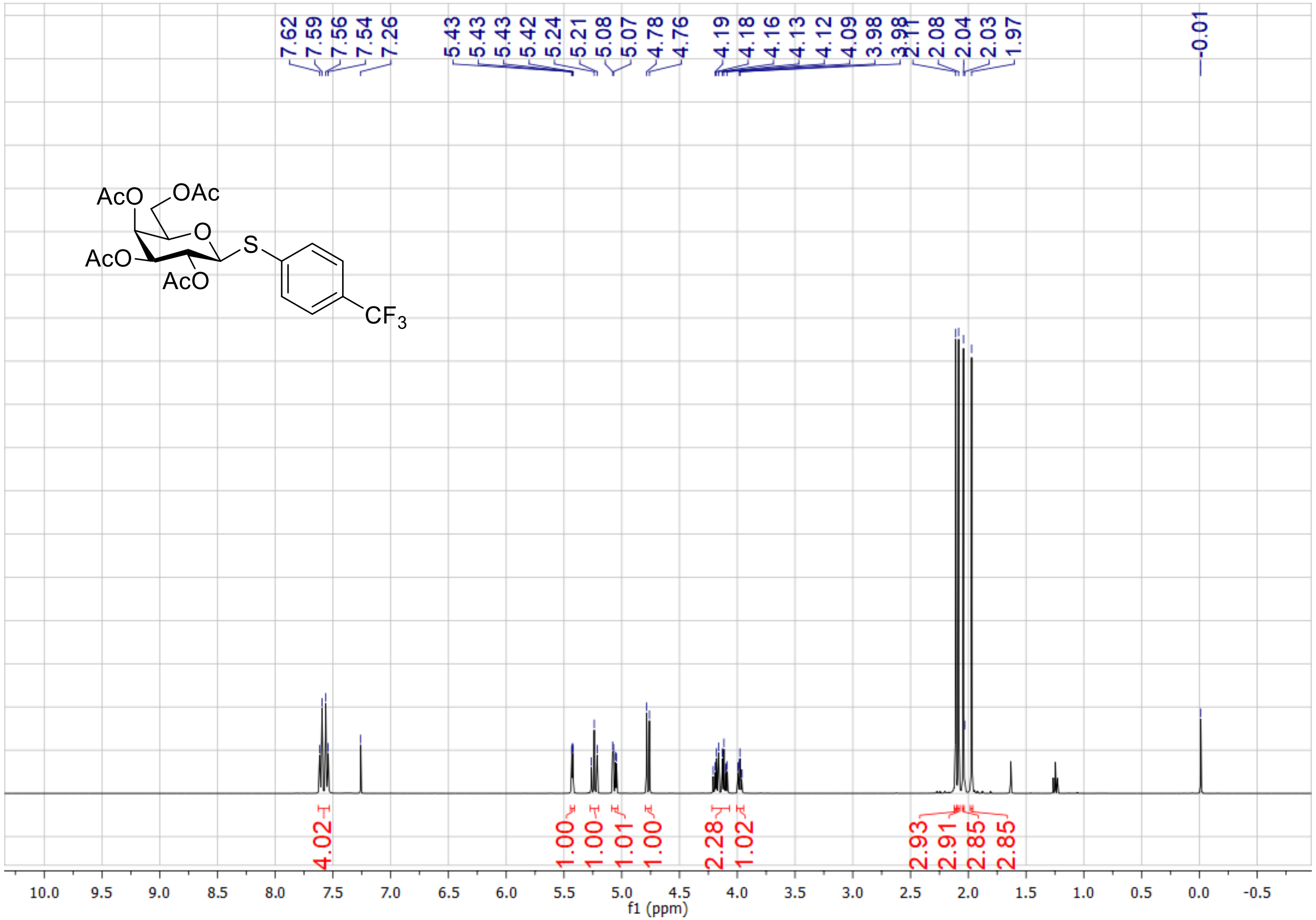

COSY (400 × 400 MHz, $CDCl_3$): *p*-(Trifluoromethyl)-phenyl 2,3,4,6-tetra-*O*-acetyl-1-thio-β-D-galactopyranoside S9

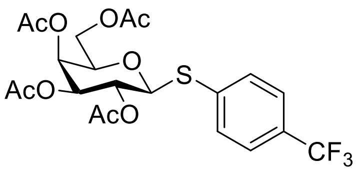

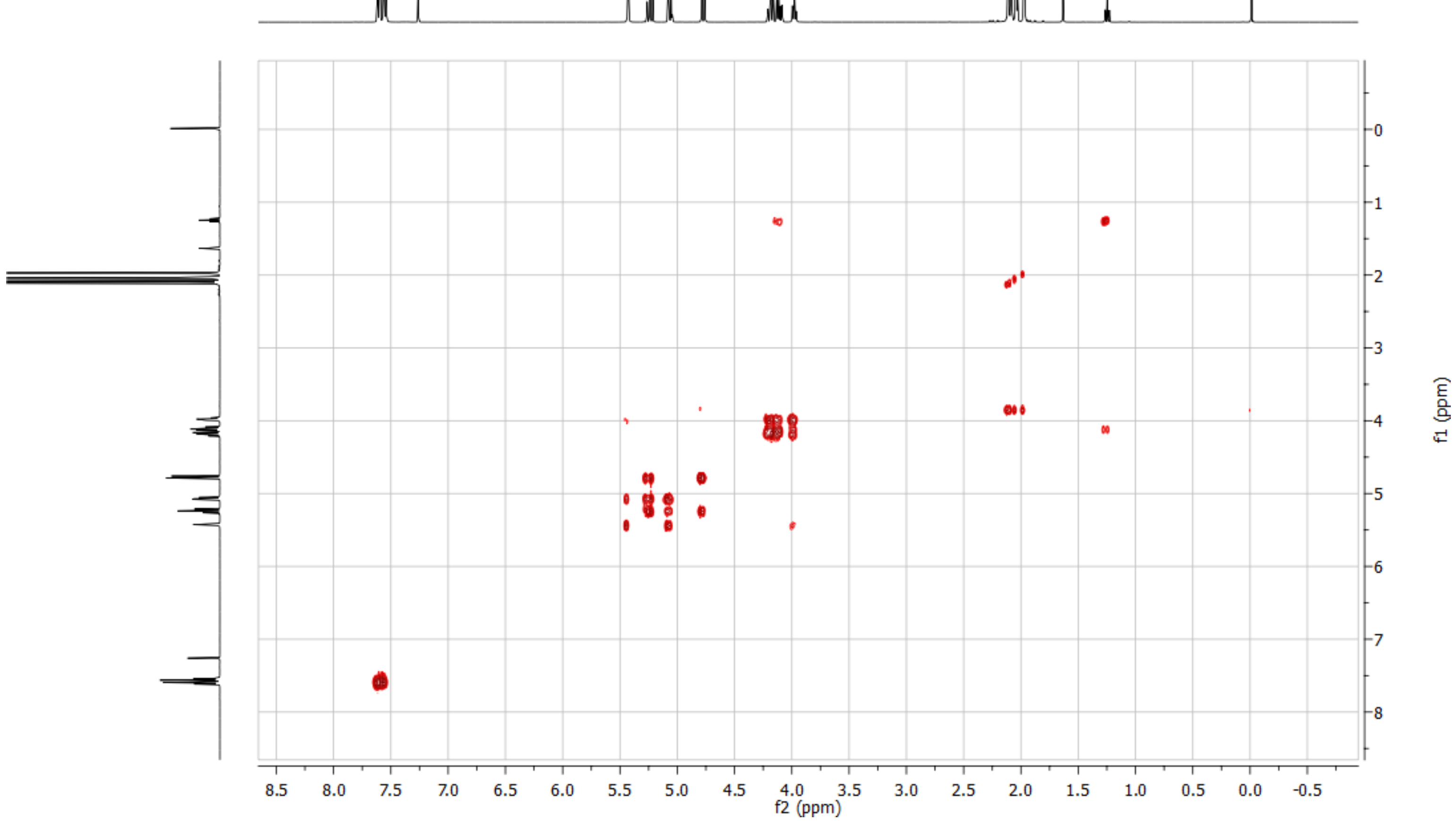

HSQC (400 × 101 MHz, CDCl$_3$): *p*-(Trifluoromethyl)-phenyl 2,3,4,6-tetra-*O*-acetyl-1-thio-β-D-galactopyranoside S9

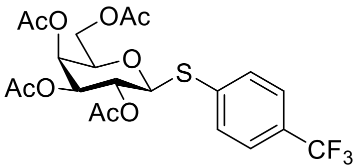

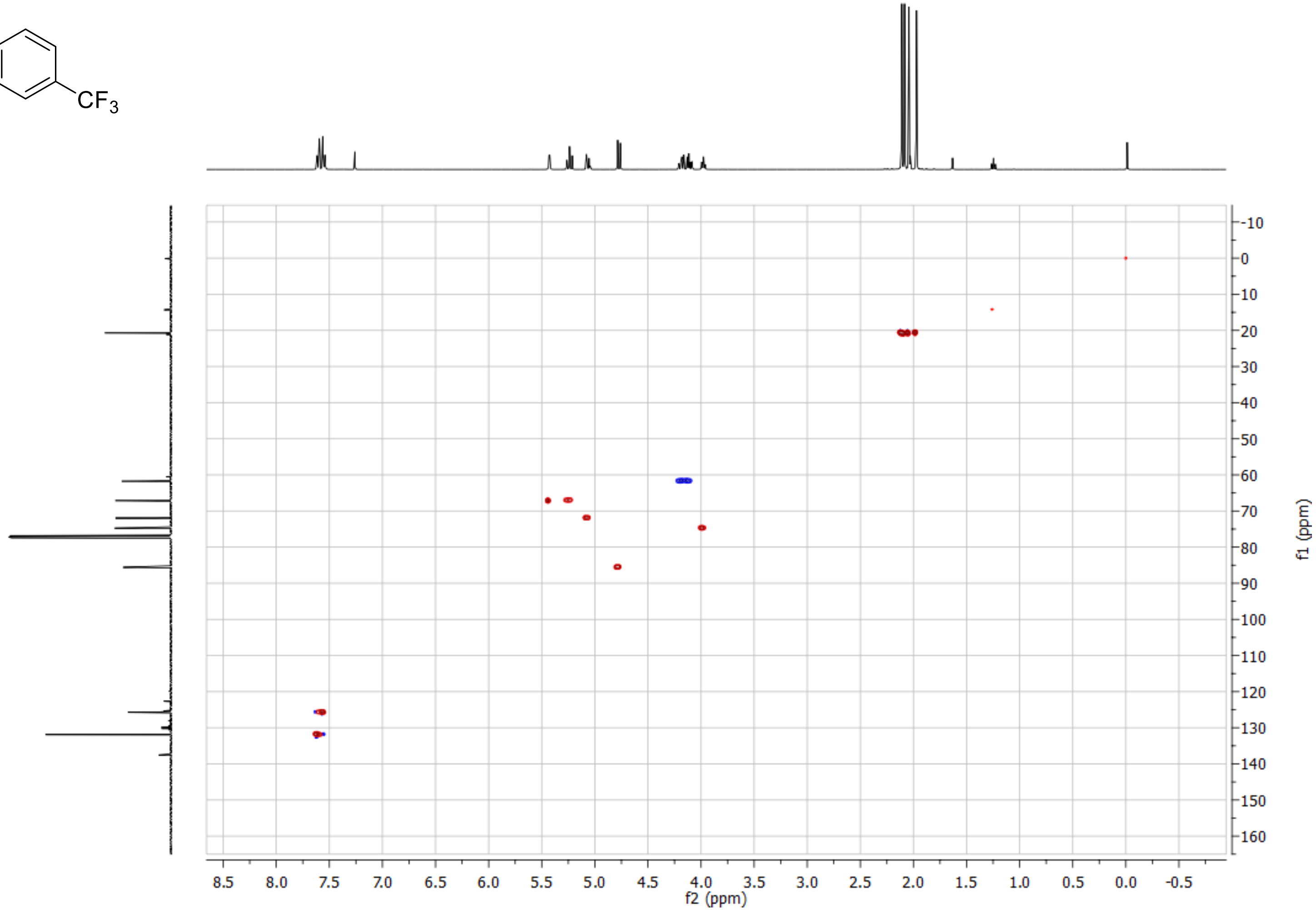

^{19}F NMR (376 MHz, CDCl$_3$): *p*-(Trifluoromethyl)-phenyl 2,3,4,6-tetra-*O*-acetyl-1-thio-β-D-galactopyranoside S9

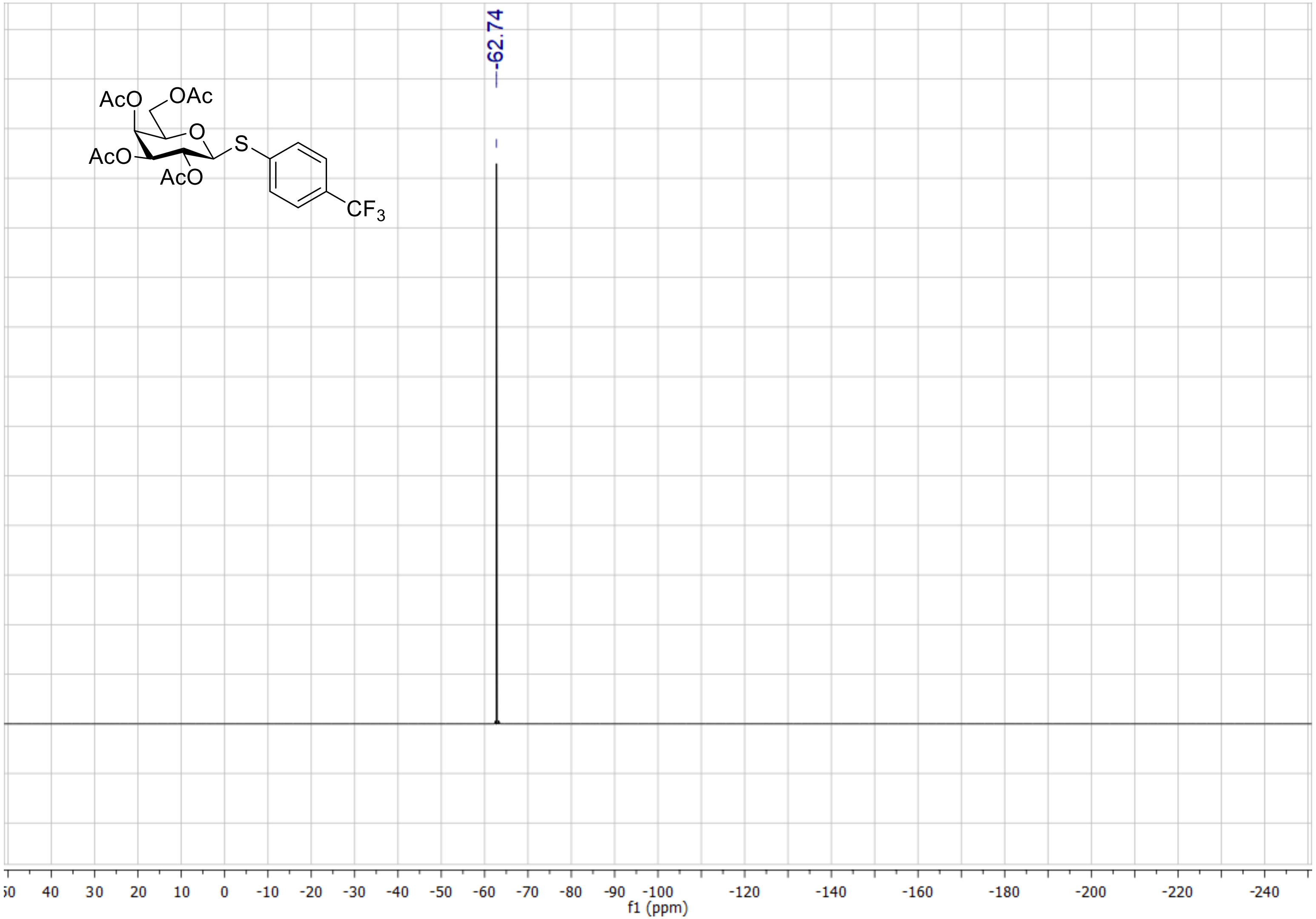

$^{13}C\{1H\}$ NMR (101 MHz, $CDCl_3$): *p*-(Trifluoromethyl)-phenyl 2,3,4,6-tetra-*O*-acetyl-1-thio-β-D-galactopyranoside S9

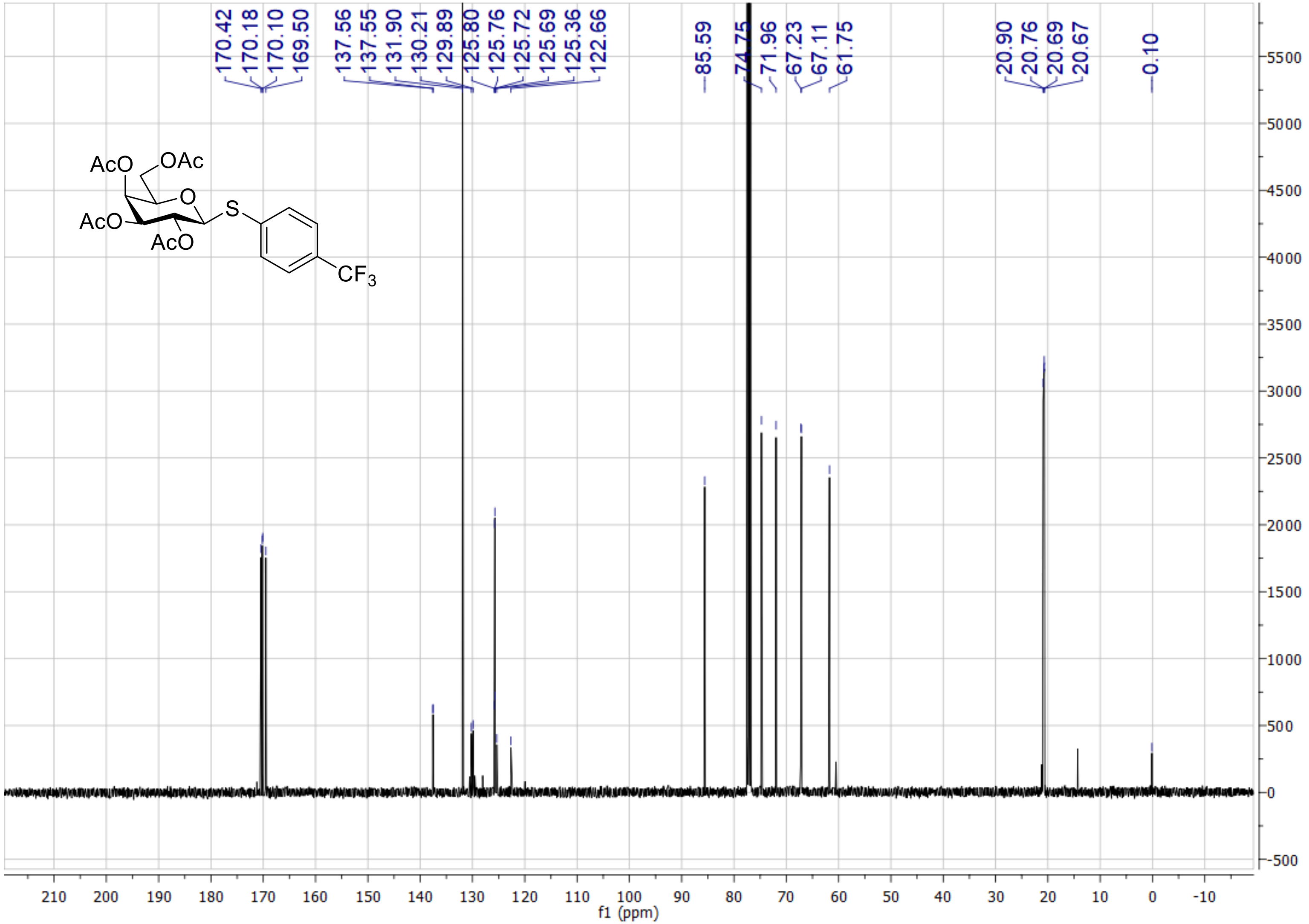

Compound S10

^{1}H NMR (400 MHz, CDCl3): *p*-(Nitro)-phenyl 2,3,4,6-tetra-*O*-acetyl-1-thio-β-D-galactopyranoside S10

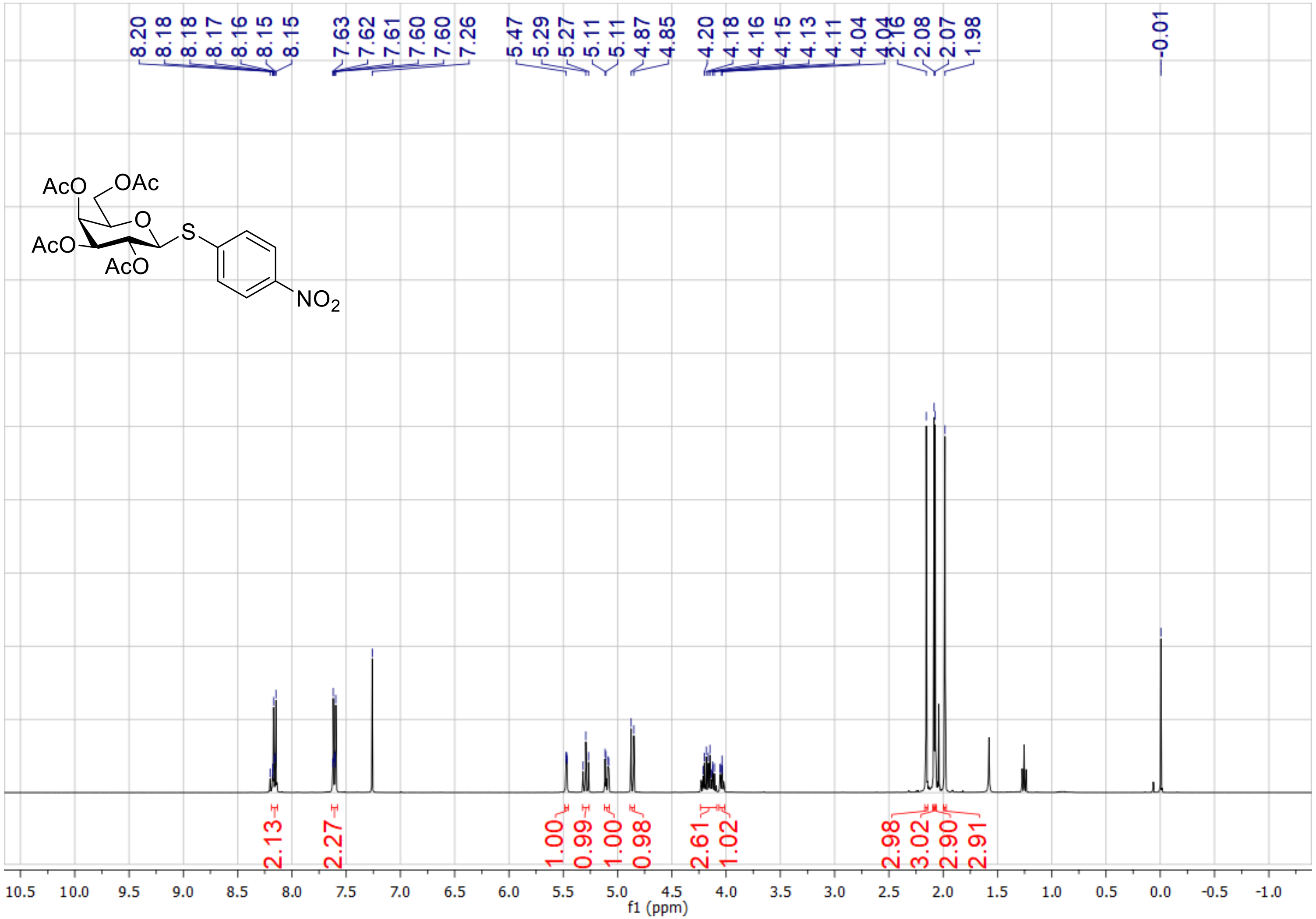

$^{13}C\{1H\}$ NMR (101 MHz, $CDCl_3$): *p*-(Nitro)-phenyl 2,3,4,6-tetra-*O*-acetyl-1-thio-β-D-galactopyranoside S10

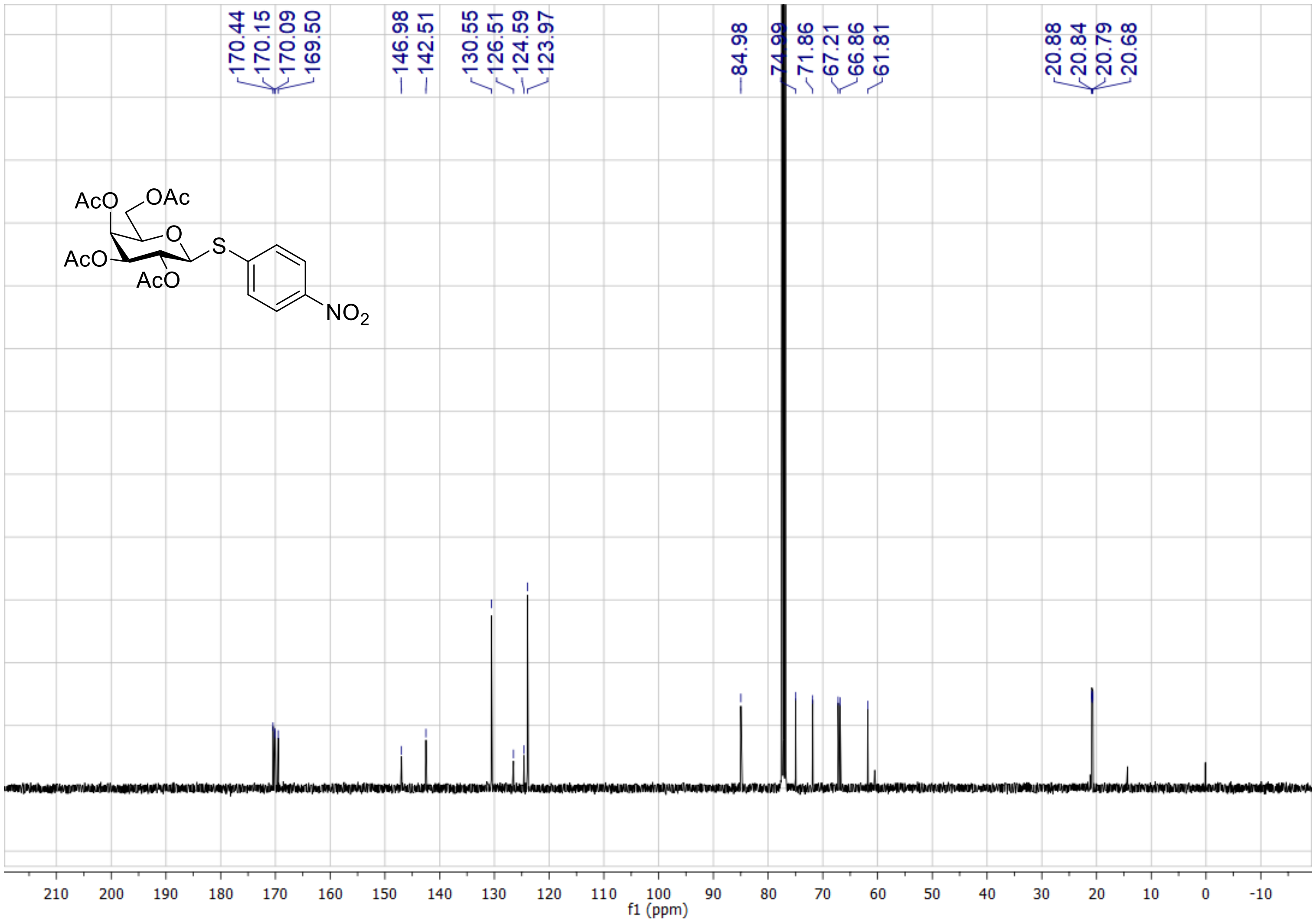

Compound S11

^{1}H NMR (400 MHz, CDCl$_3$): *p*-(Methoxy)-phenyl-2,3,4,6-tetra-*O*-acetyl-1-thio-β-D-galactopyranoside S11

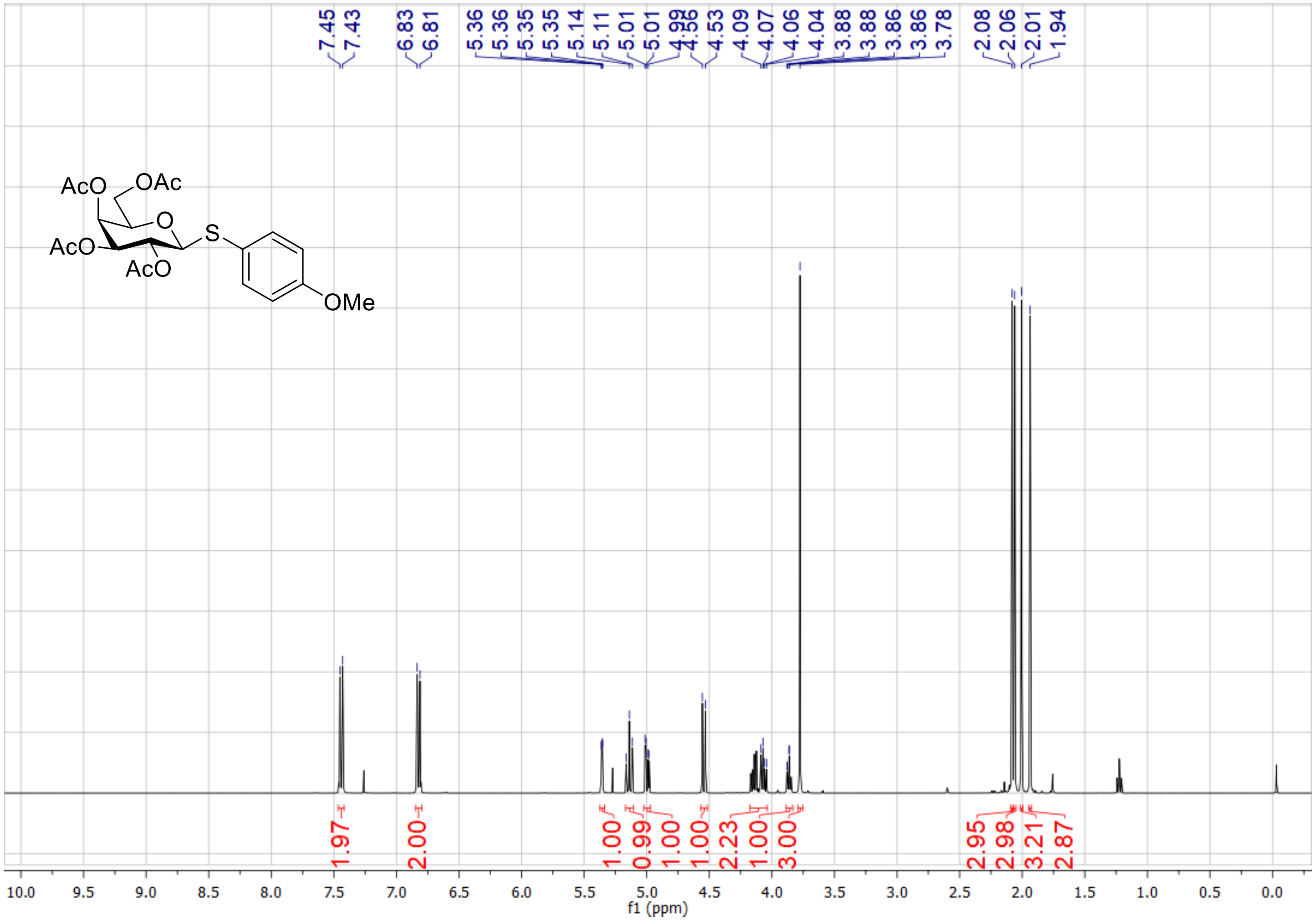

^{13}C{1H} NMR (101 MHz, $CDCl_3$): *p*-(Methoxy)-phenyl-2,3,4,6-tetra-*O*-acetyl-1-thio-β-D-galactopyranoside S11

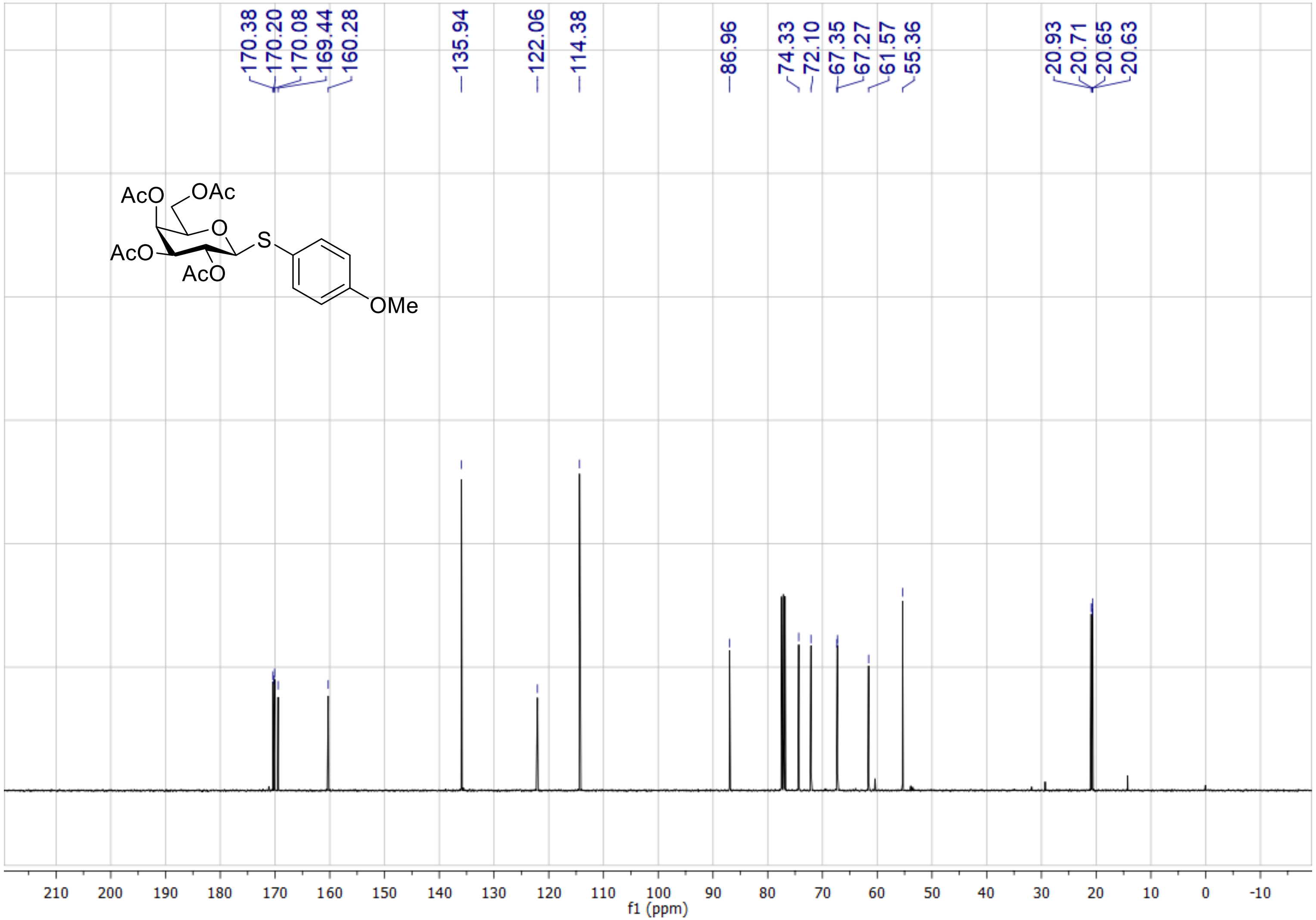

Compound 57

^{1}H NMR (400 MHz, MeOD): *p*-(Trifluoromethyl)-phenyl 1-thio-β-D-galactopyranoside 57

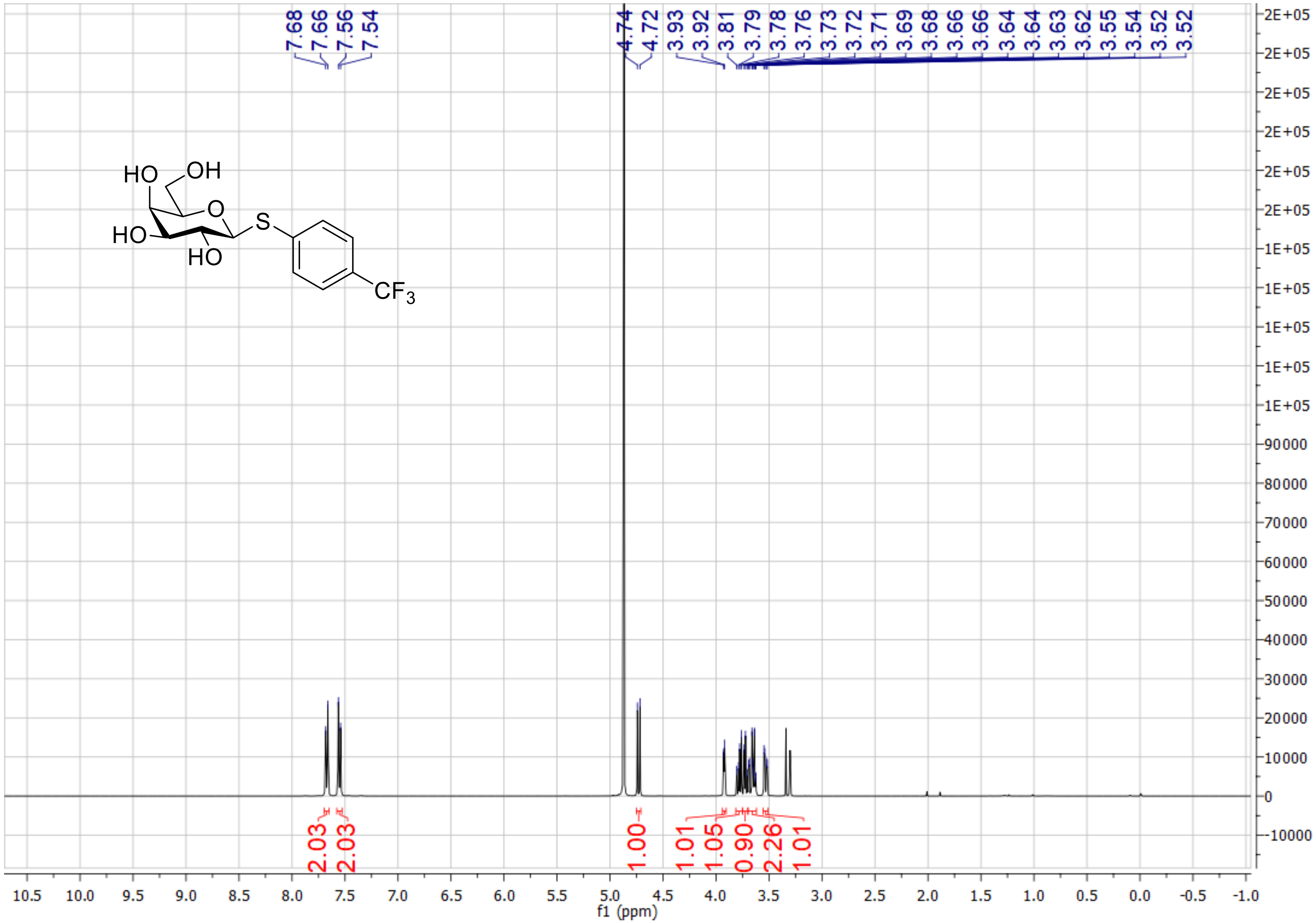

COSY (400 × 400 MHz, MeOD): *p*-(Trifluoromethyl)-phenyl 1-thio-β-D-galactopyranoside 57

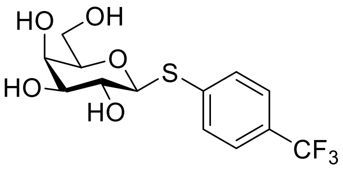

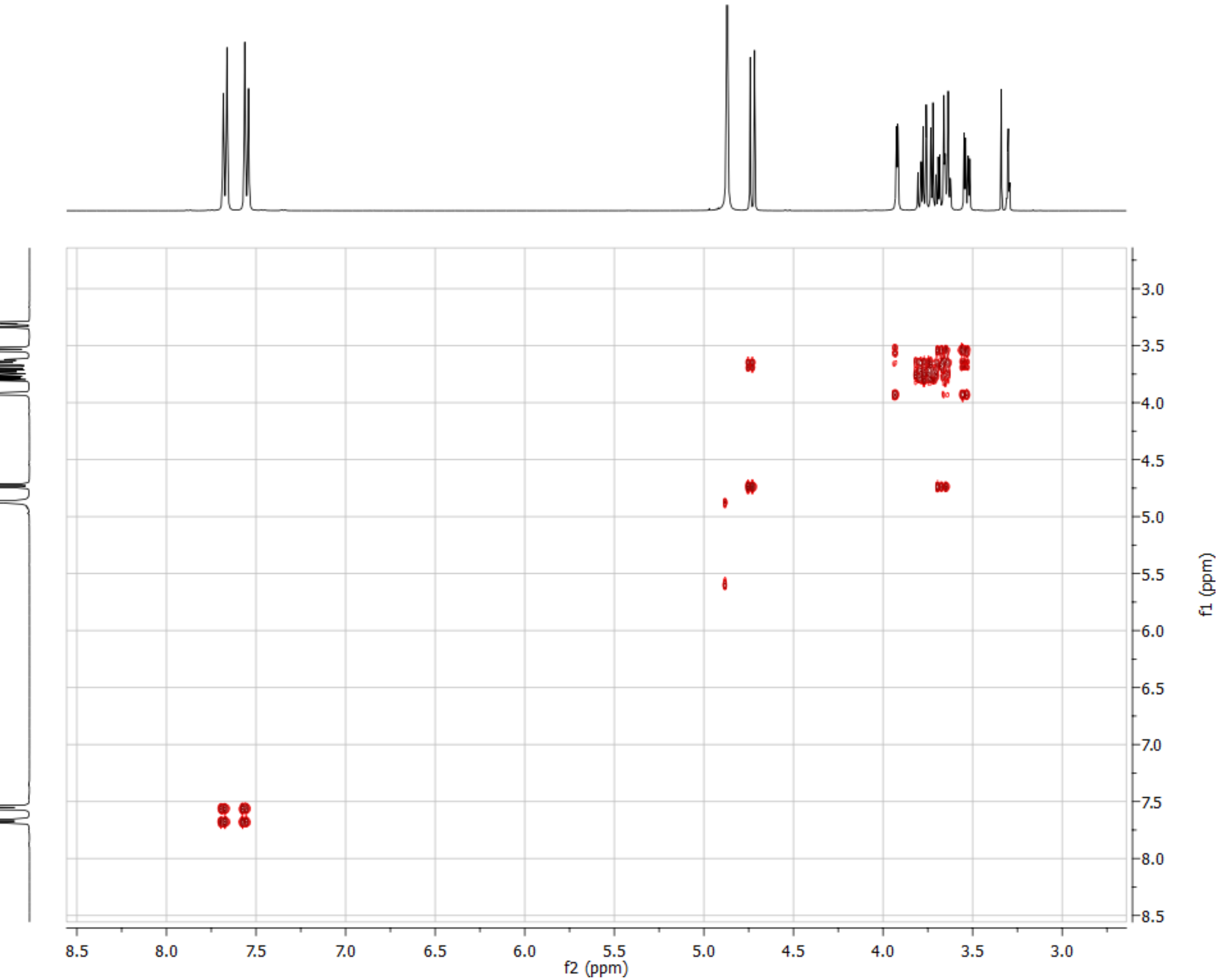

HSQC (400 × 101 MHz, MeOD): *p*-(Trifluoromethyl)-phenyl 1-thio-β-D-galactopyranoside 57

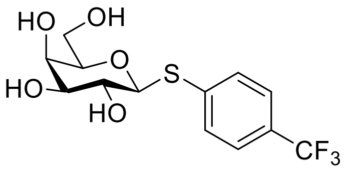

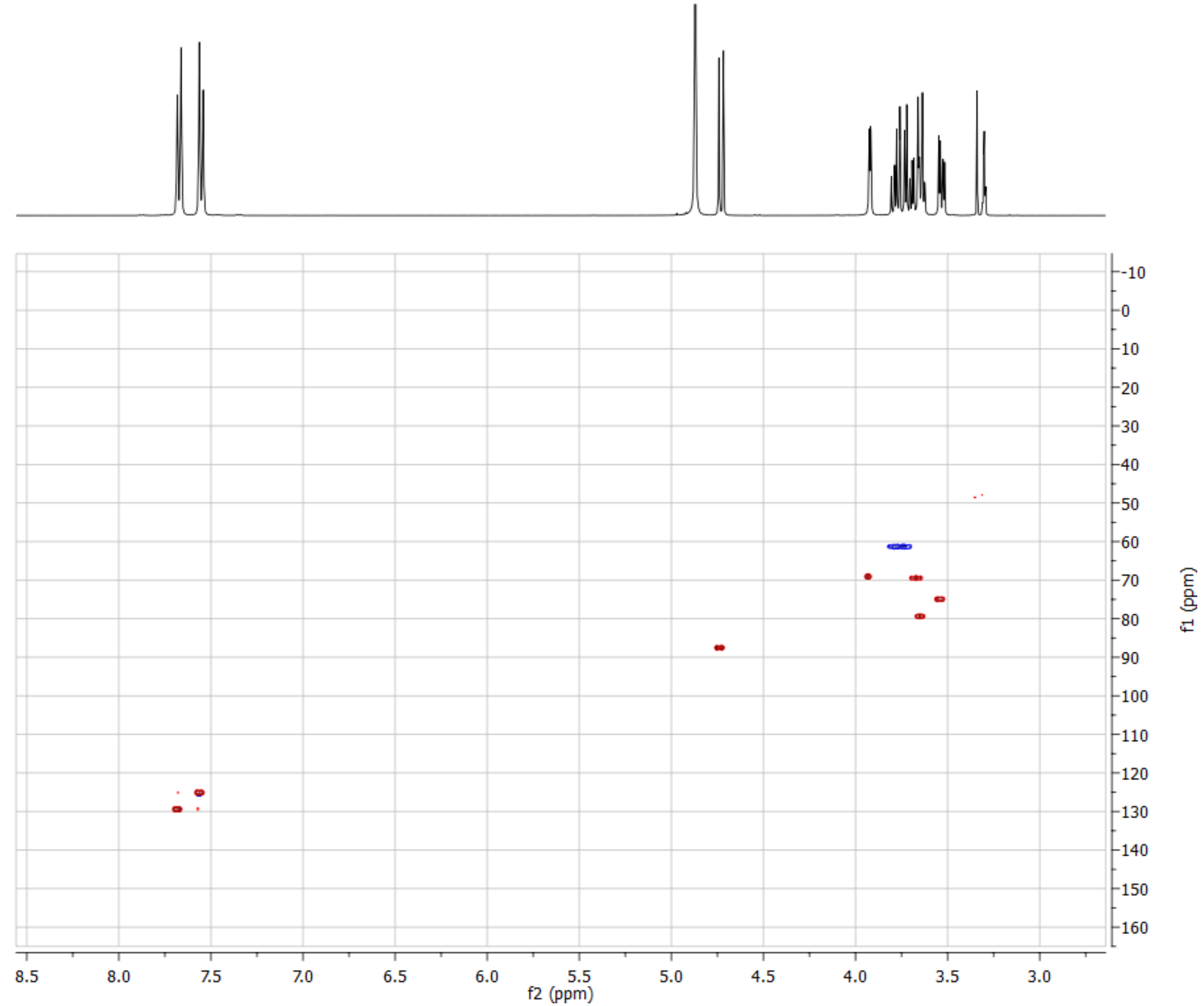

^{19}F NMR (376 MHz, MeOD): *p*-(Trifluoromethyl)-phenyl 1-thio-β-D-galactopyranoside 57

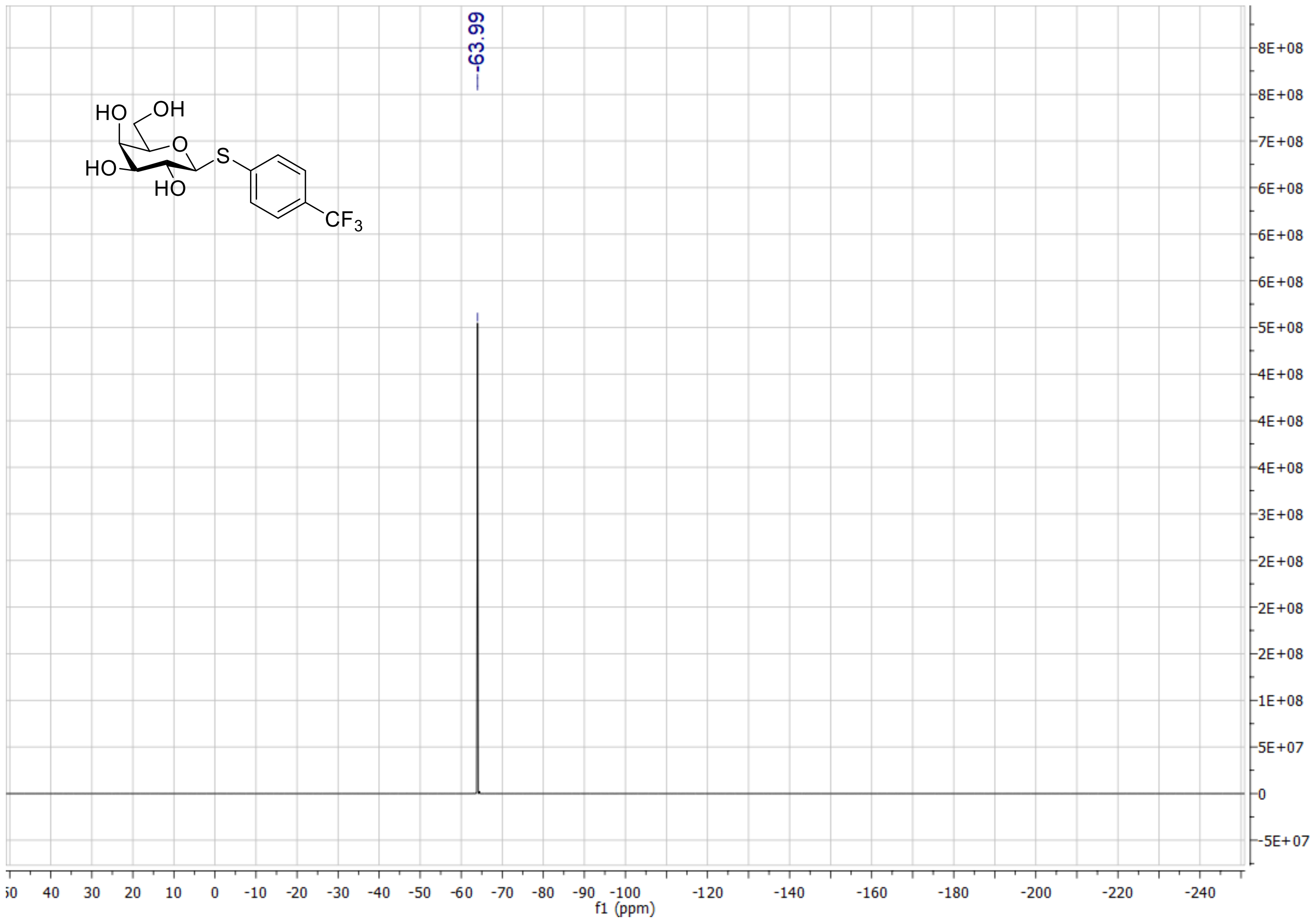

$^{13}C\{1H\}$ NMR (101 MHz, MeOD): *p*-(Trifluoromethyl)-phenyl 1-thio-β-D-galactopyranoside 57

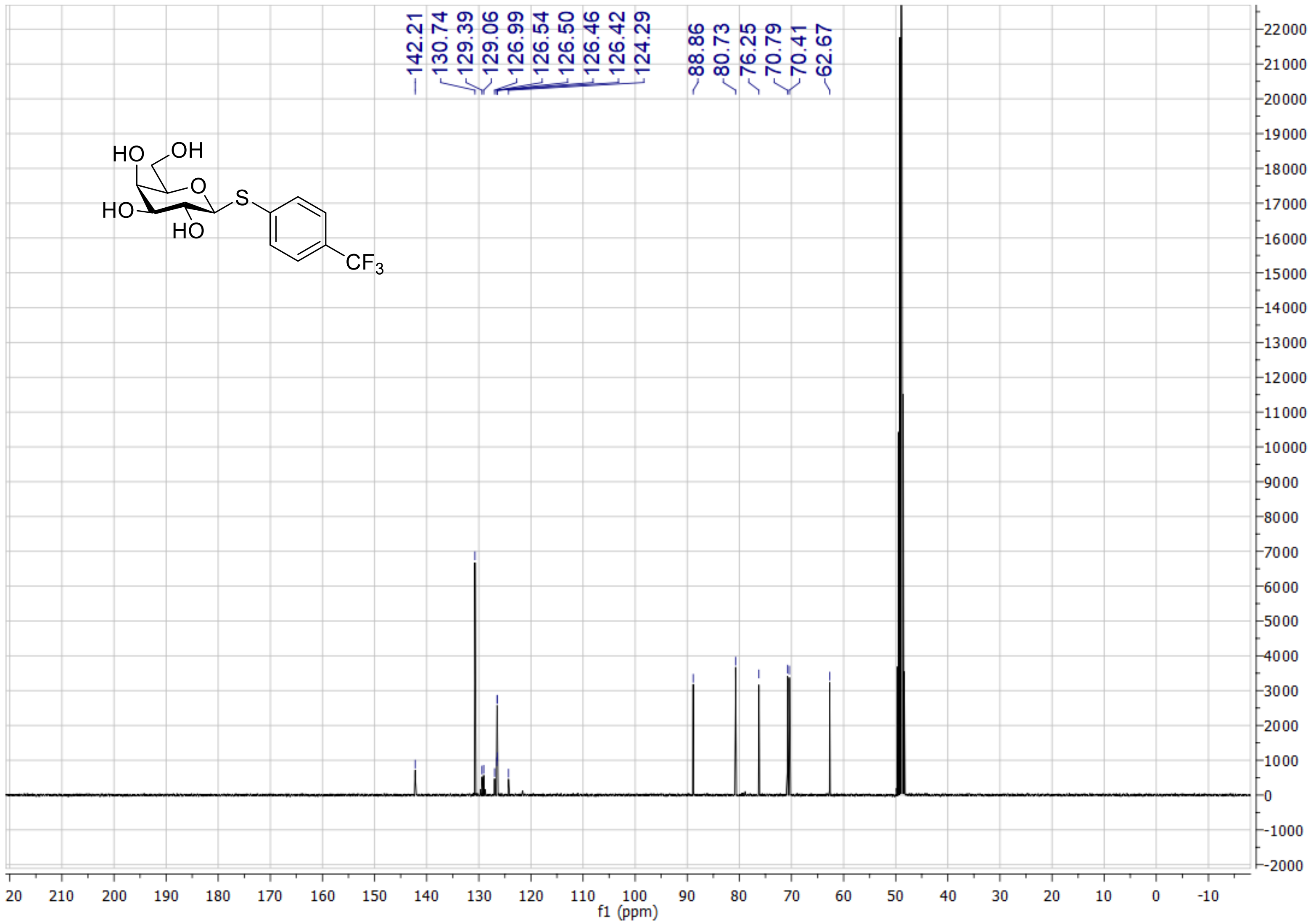

Compound 67

^{1}H NMR (400 MHz, MeOD): *p*-(Nitro)-phenyl 1-thio-β-D-galactopyranoside 67

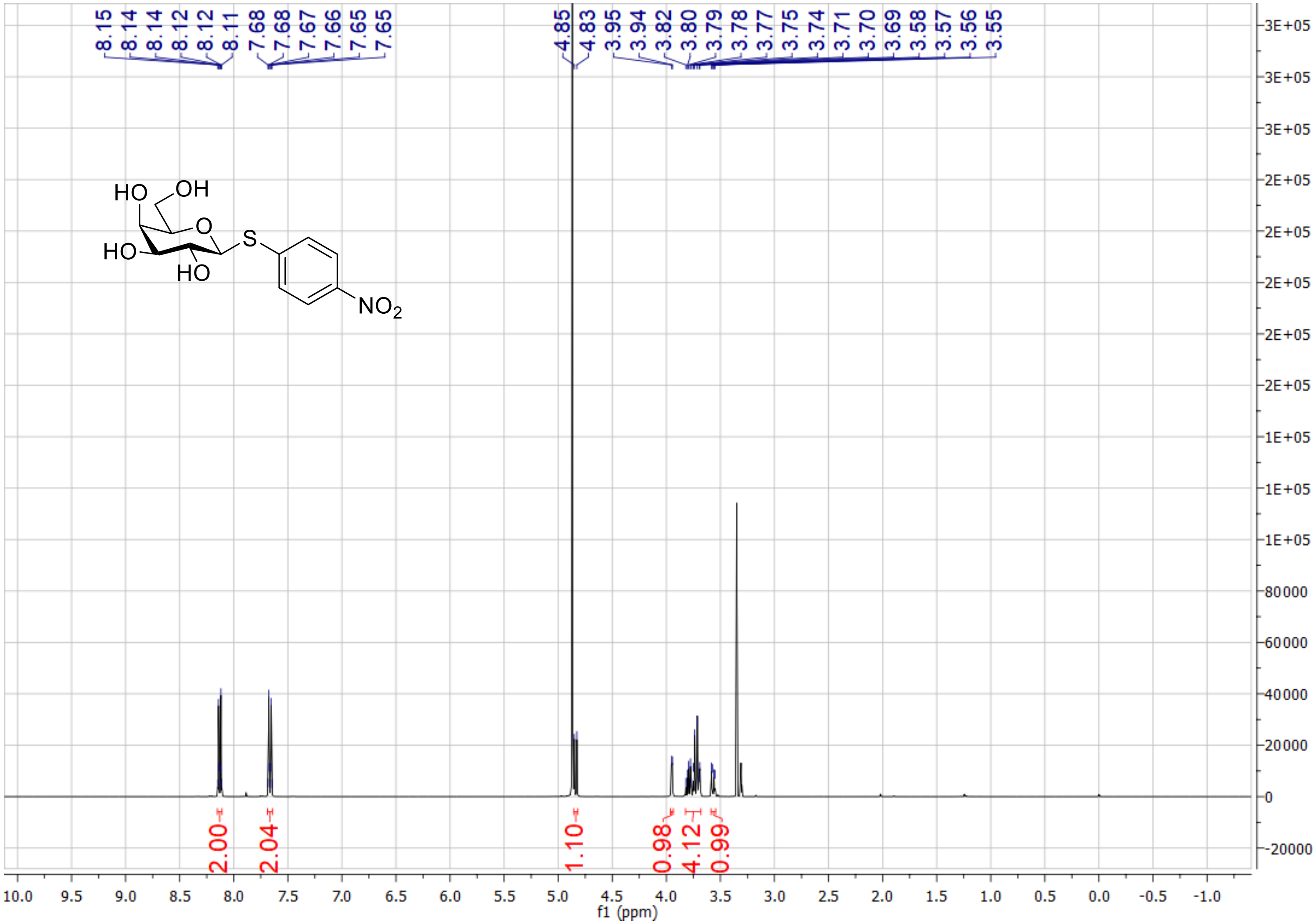

$^{13}C\{1H\}$ NMR (101 MHz, MeOD): *p*-(Nitro)-phenyl 1-thio-β-D-galactopyranoside 67

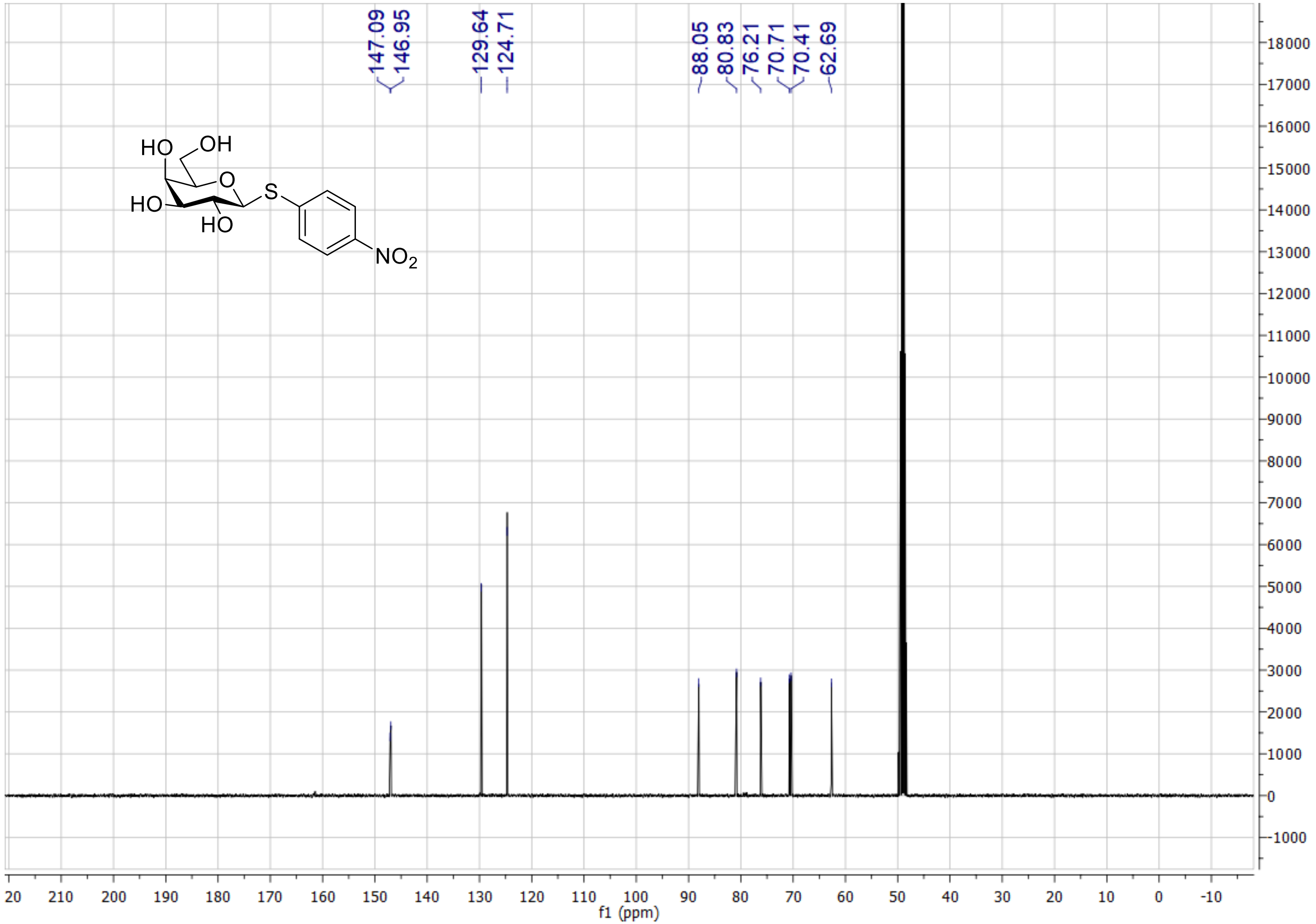

Compound 62

^{1}H NMR (400 MHz, MeOD): *p*-(Methoxy)-phenyl 1-thio-β-D-galactopyranoside 62

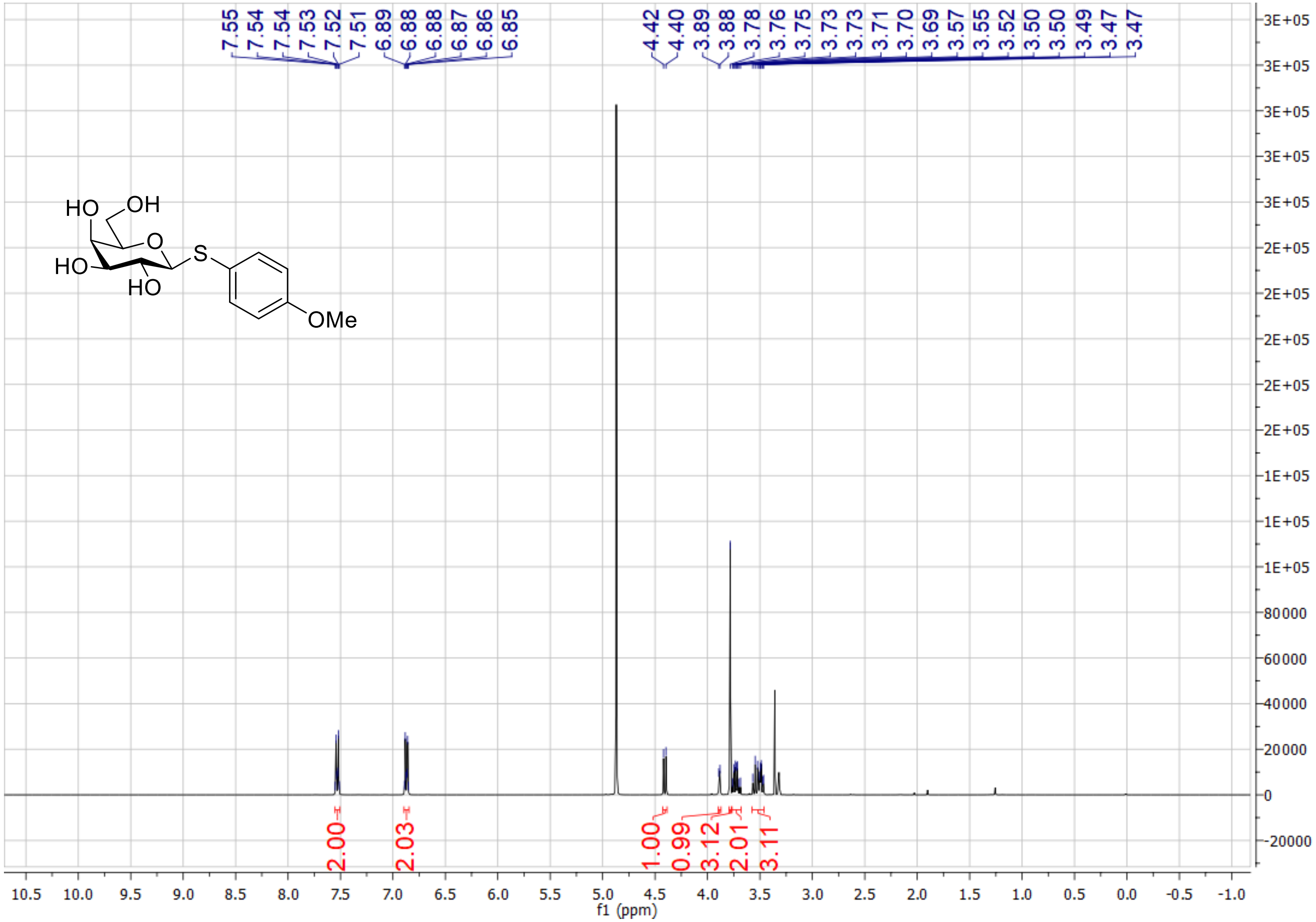

$^{13}C\{1H\}$ NMR (101 MHz, MeOD): *p*-(Methoxy)-phenyl 1-thio-β-D-galactopyranoside 62

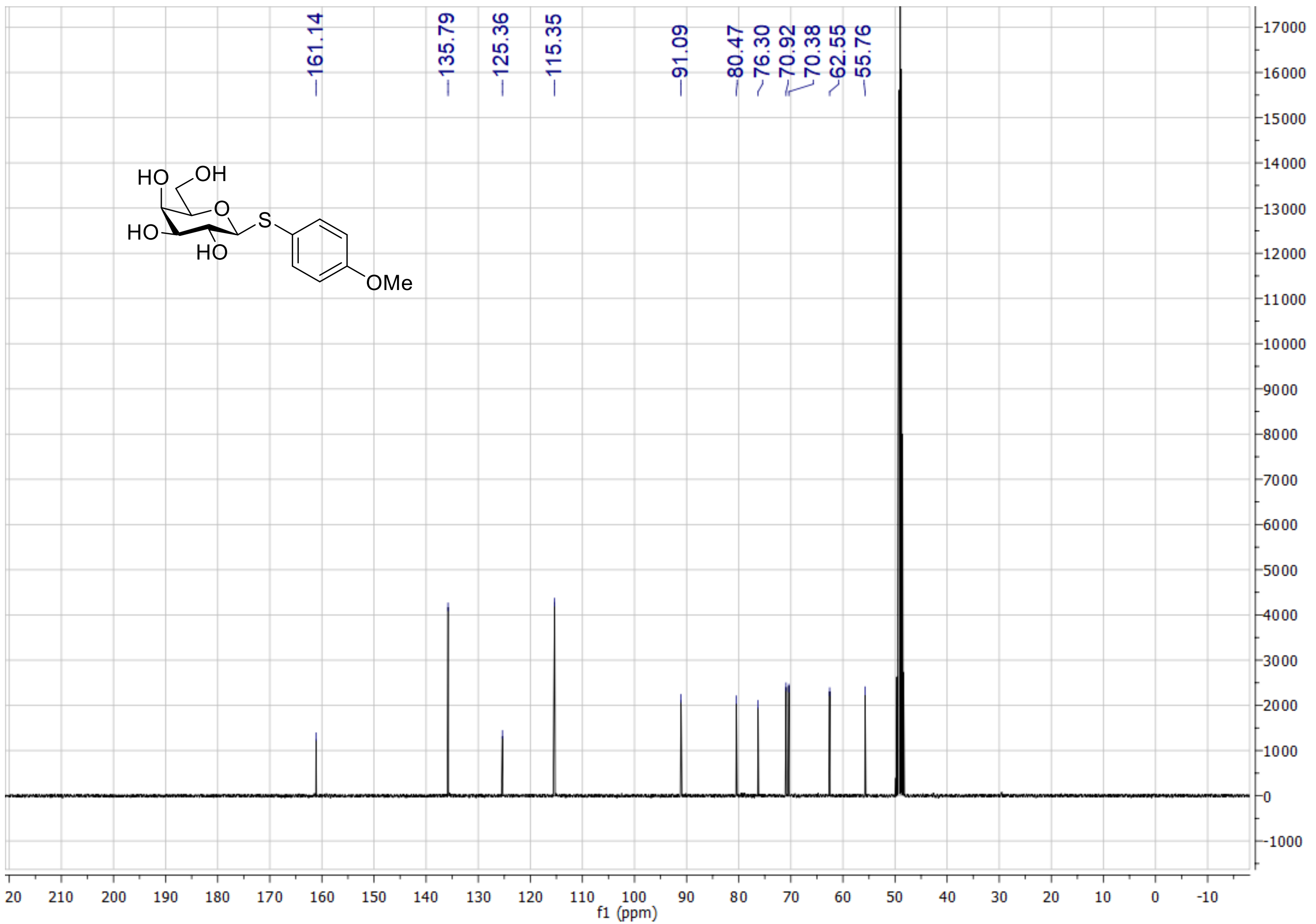

Acylation experiments

Compound 2

^{1}H NMR (400 MHz, $CDCl_3$): Ethyl 2,3,6-tri-*O*-benzoyl-1-thio-α-D-galactopyranoside 2

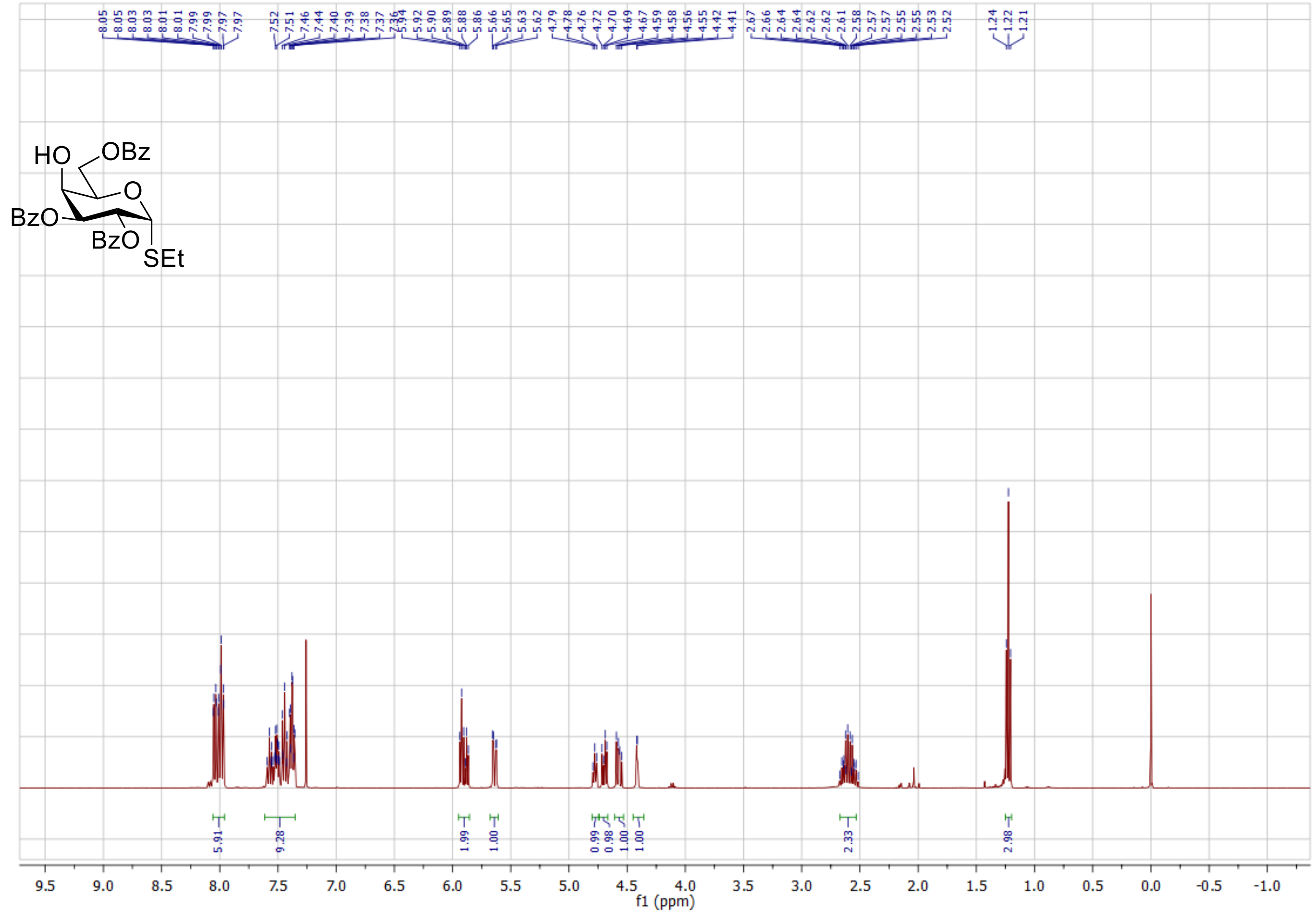

COSY (400 × 400 MHz, $CDCl_3$): Ethyl 2,3,6-tri-*O*-benzoyl-1-thio-α-D-galactopyranoside 2

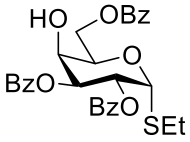

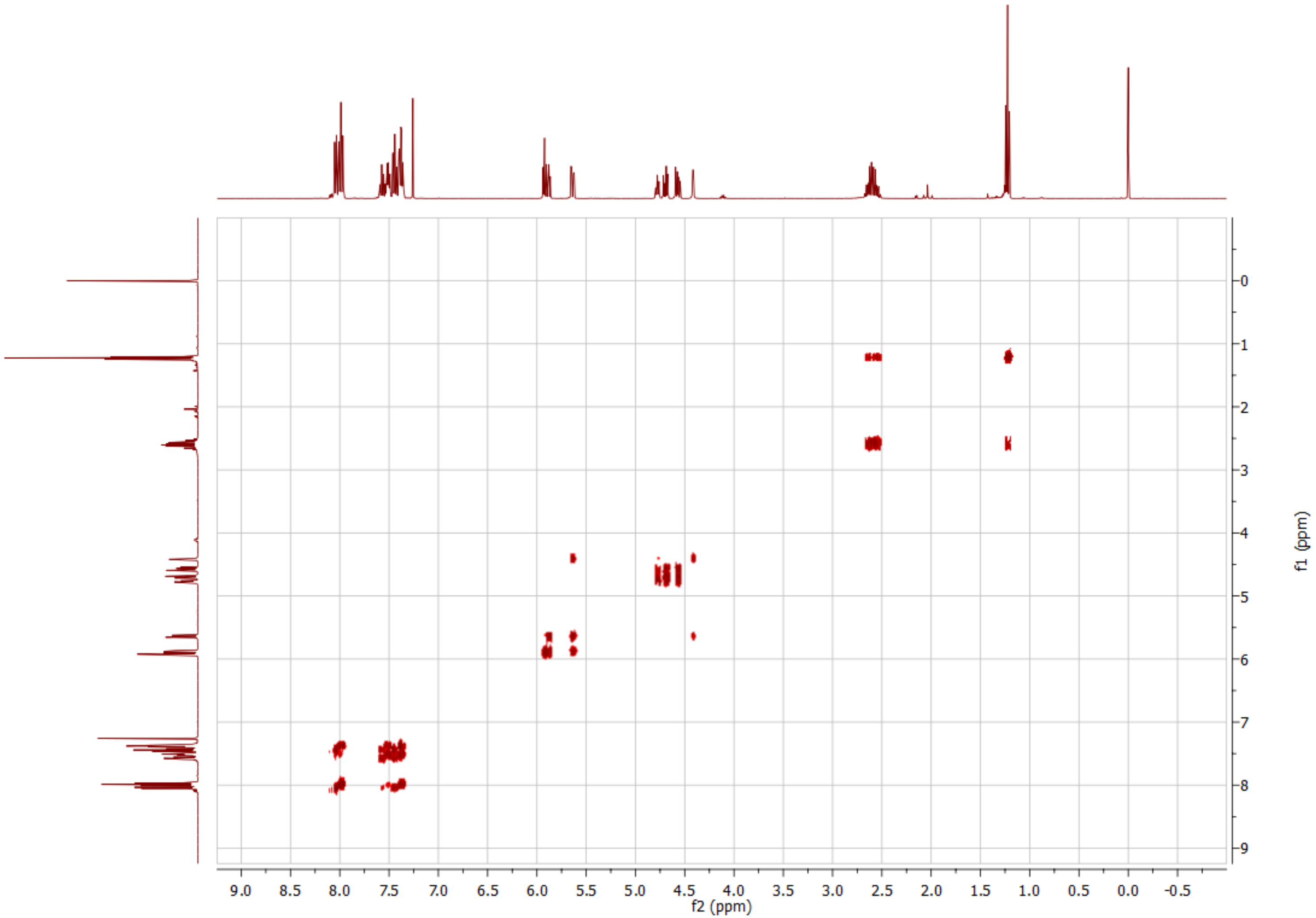

HSQC (400 × 101 MHz, CDCl$_3$): Ethyl 2,3,6-tri-*O*-benzoyl-1-thio-α-D-galactopyranoside 2

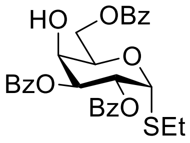

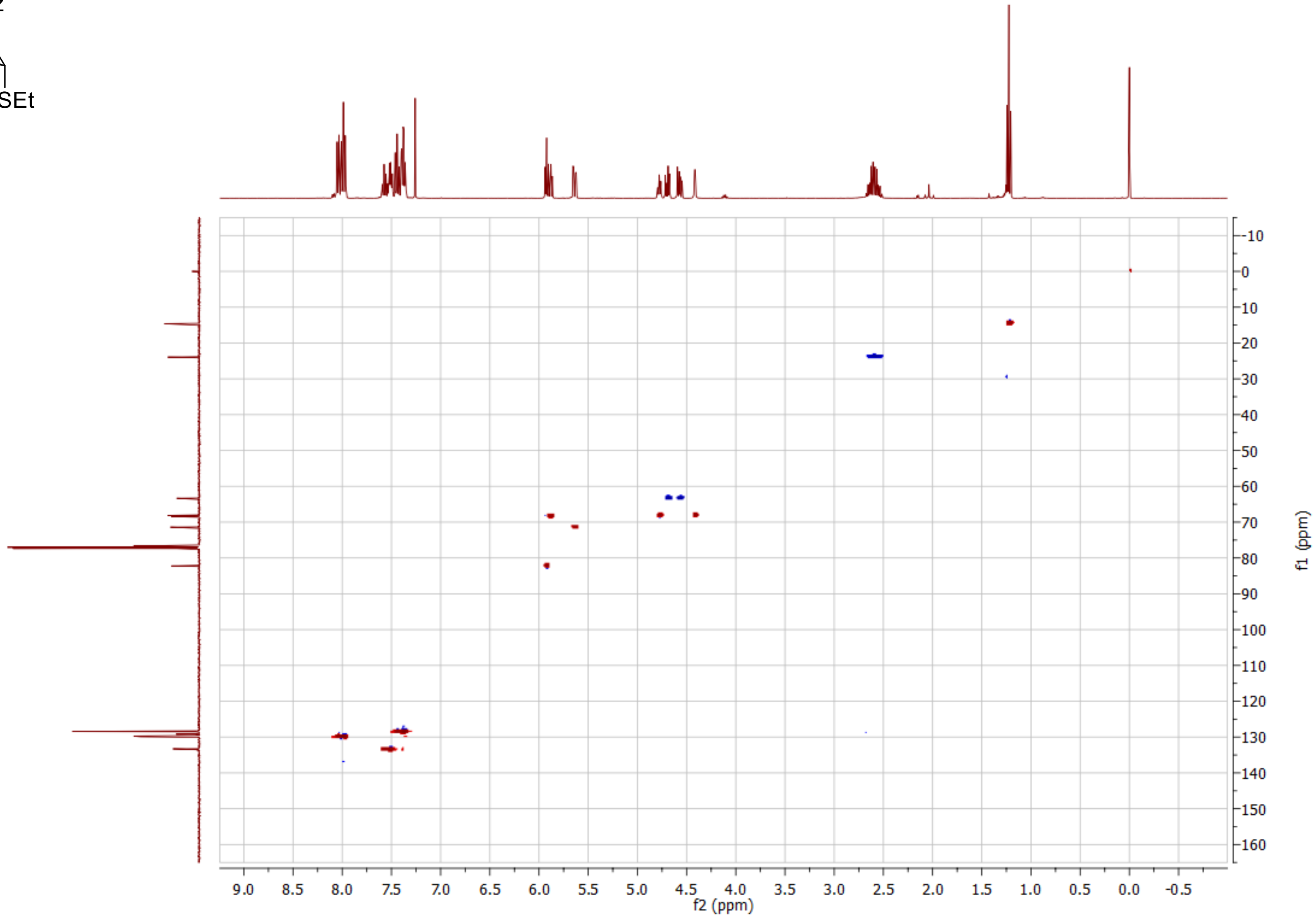

HMBC (400 × 101 MHz, CDCl3): Ethyl 2,3,6-tri-*O*-benzoyl-1-thio-α-D-galactopyranoside 2

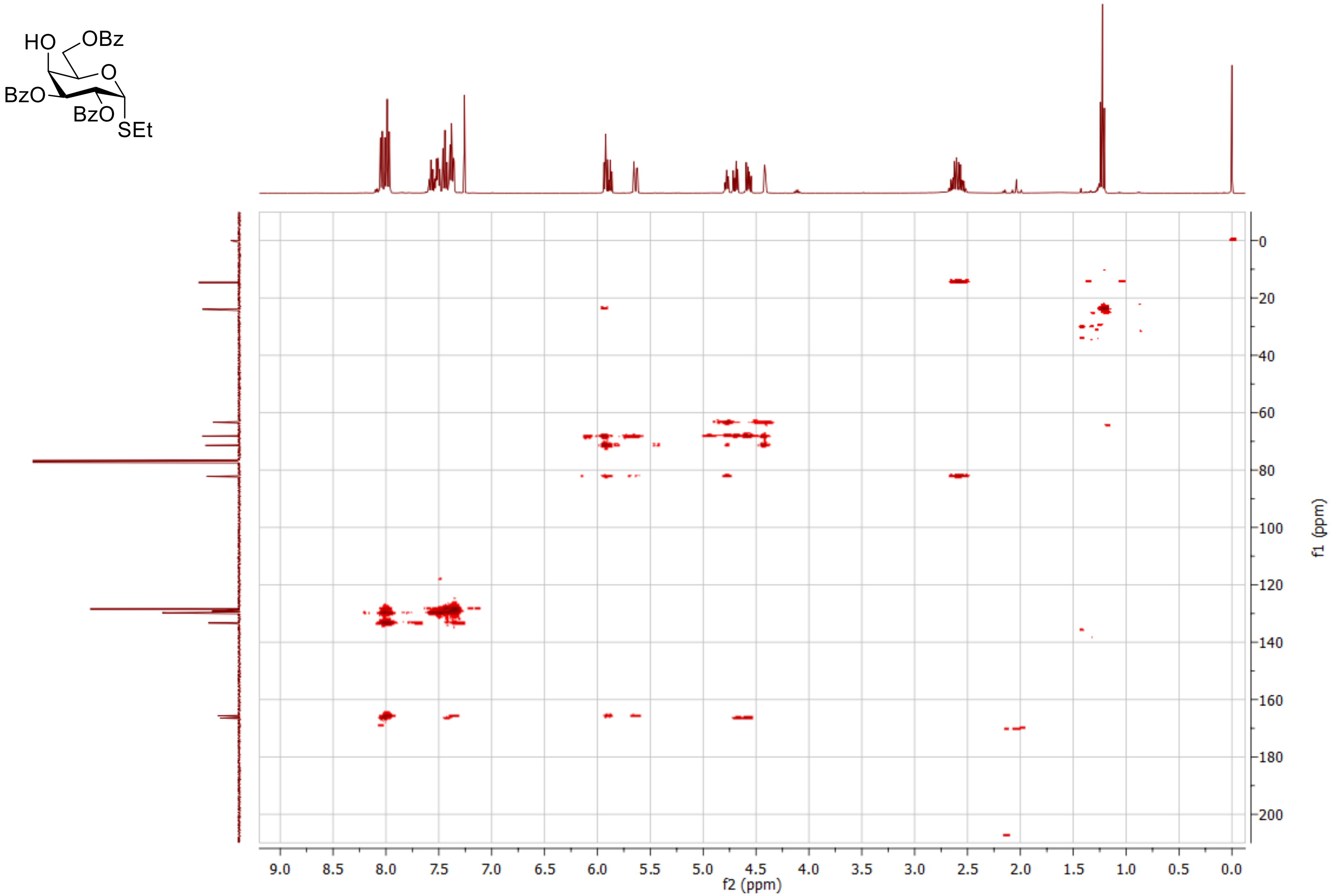

$^{13}C\{1H\}$ NMR (101 MHz, $CDCl_3$): Ethyl 2,3,6-tri-*O*-benzoyl-1-thio-α-D-galactopyranoside 2

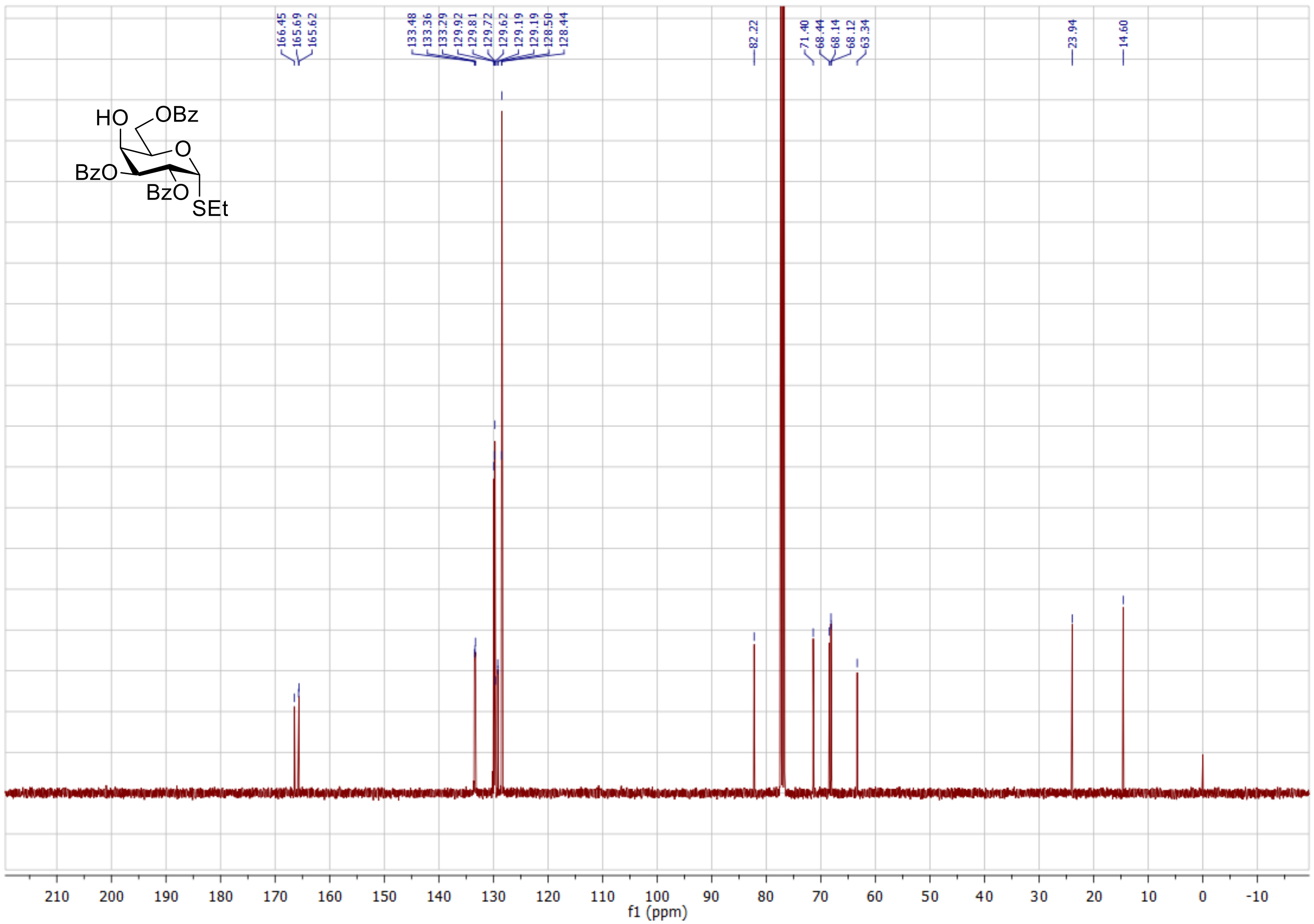

Compound 4

^{1}H NMR (400 MHz, CDCl3) Phenyl 2,3,6-tri-*O*-benzoyl-1-thio-α-D-galactopyranoside 4

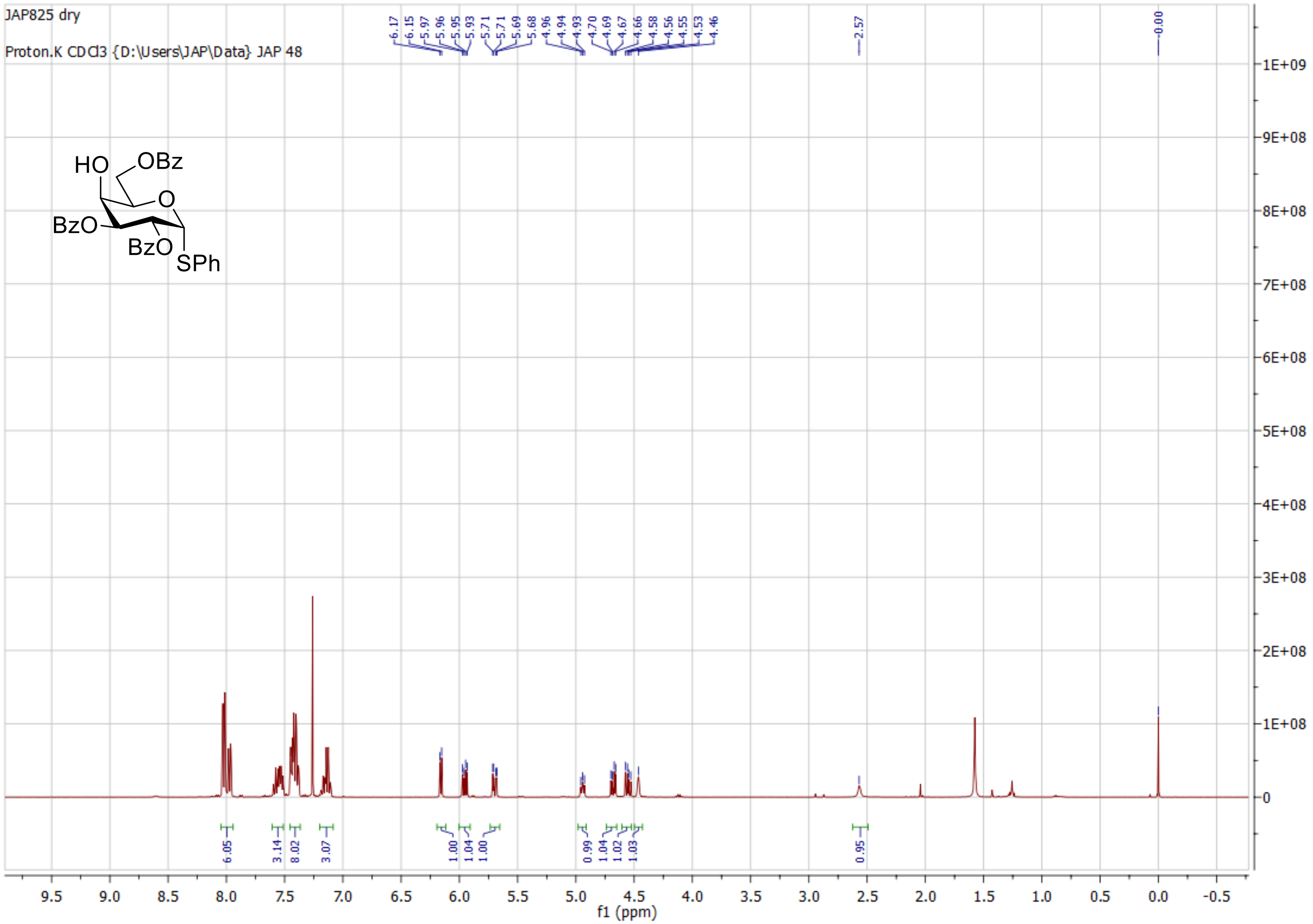

COSY (400 × 400 MHz, $CDCl_3$) Phenyl 2,3,6-tri-*O*-benzoyl-1-thio-α-D-galactopyranoside 4

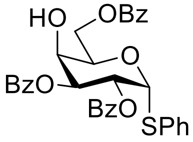

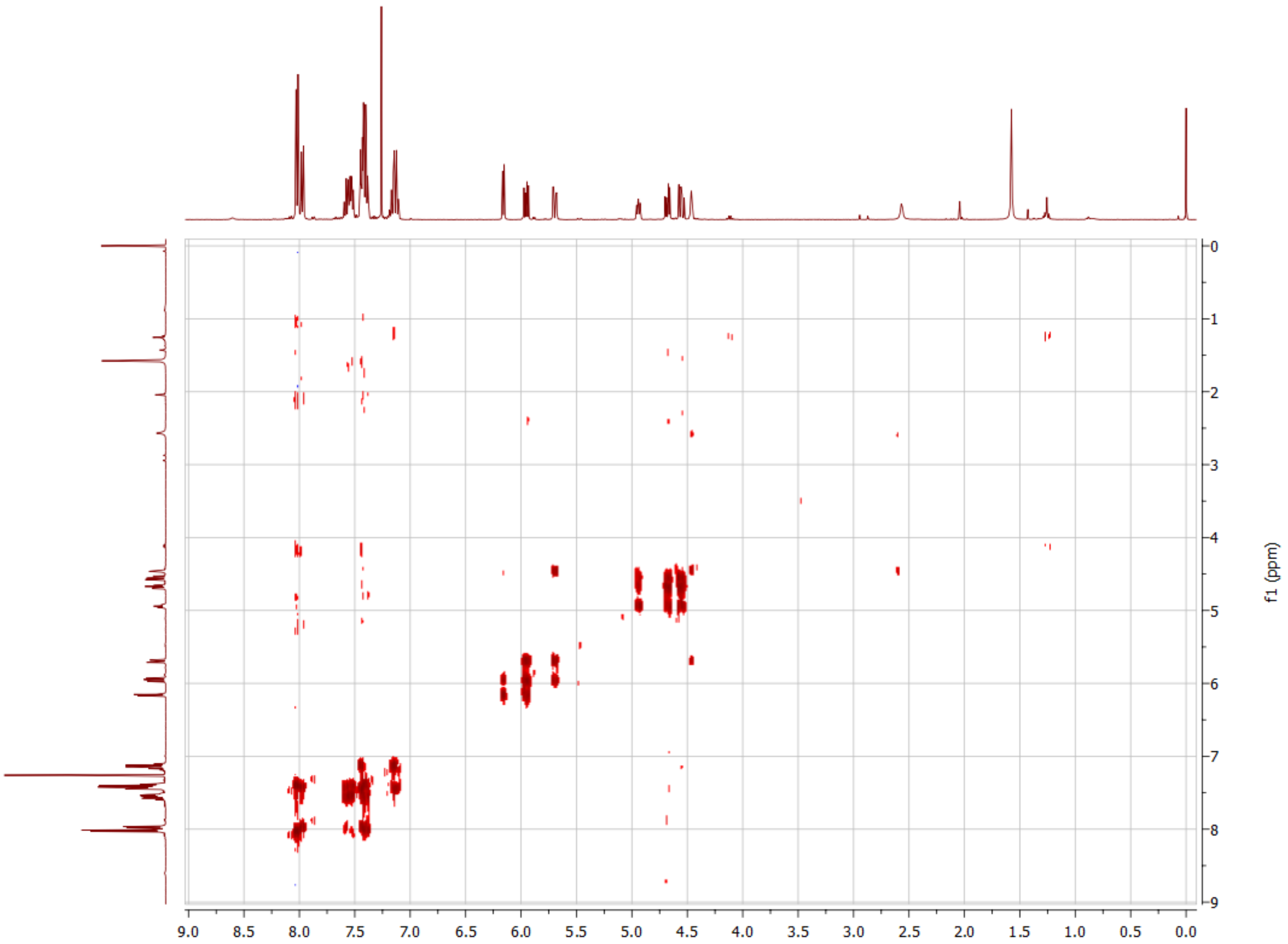

HSQC (400 × 101 MHz, CDCl3) Phenyl 2,3,6-tri-*O*-benzoyl-1-thio-α-D-galactopyranoside 4

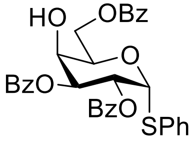

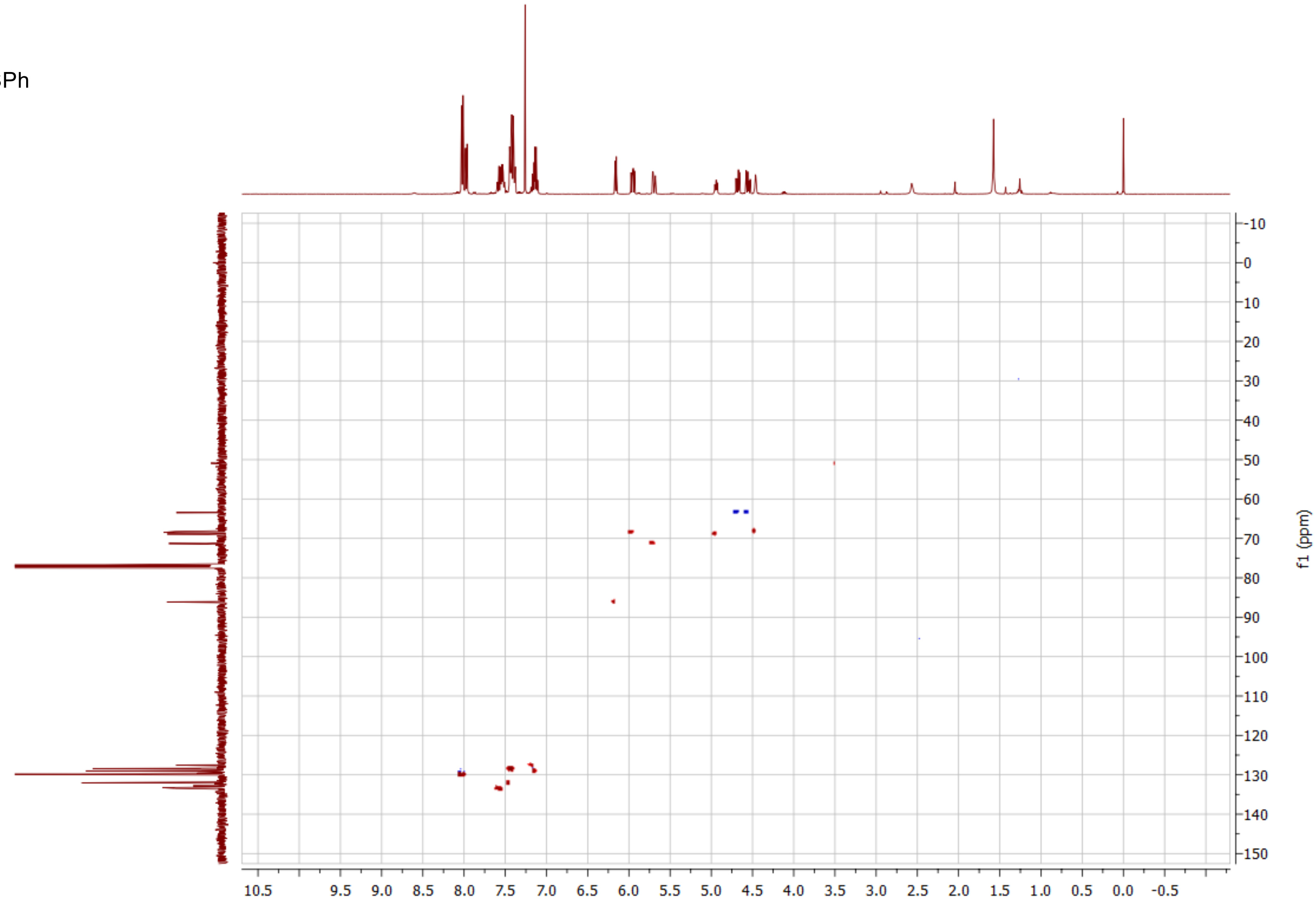

$^{13}C\{1H\}$ NMR (101 MHz, $CDCl_3$) Phenyl 2,3,6-tri-*O*-benzoyl-1-thio-α-D-galactopyranoside 4

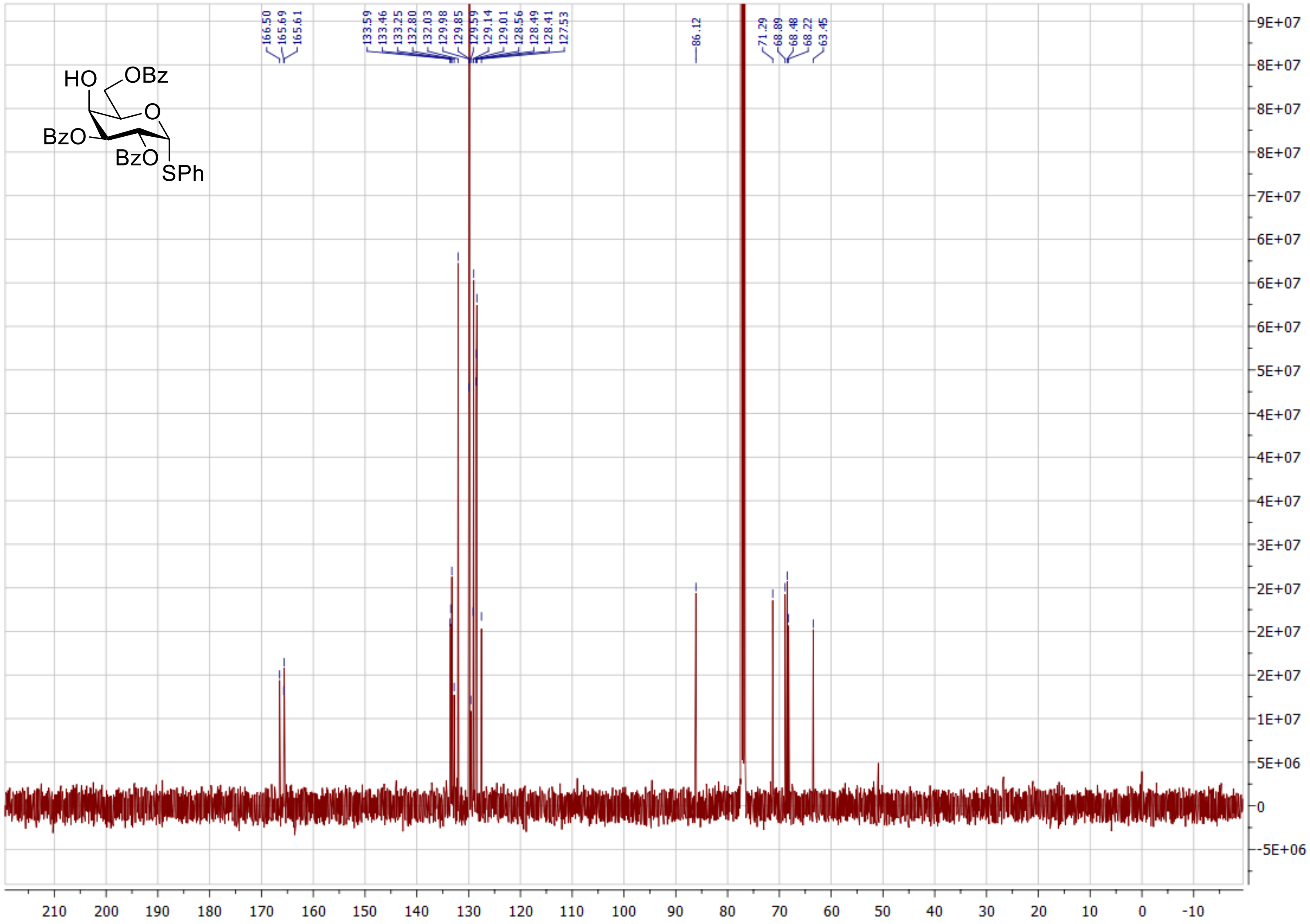

Compound 6

^{1}H NMR (400 MHz, CDCl3): Phenyl 2,3,6-tri-*O*-benzoyl-α-D-galactopyranoside 6

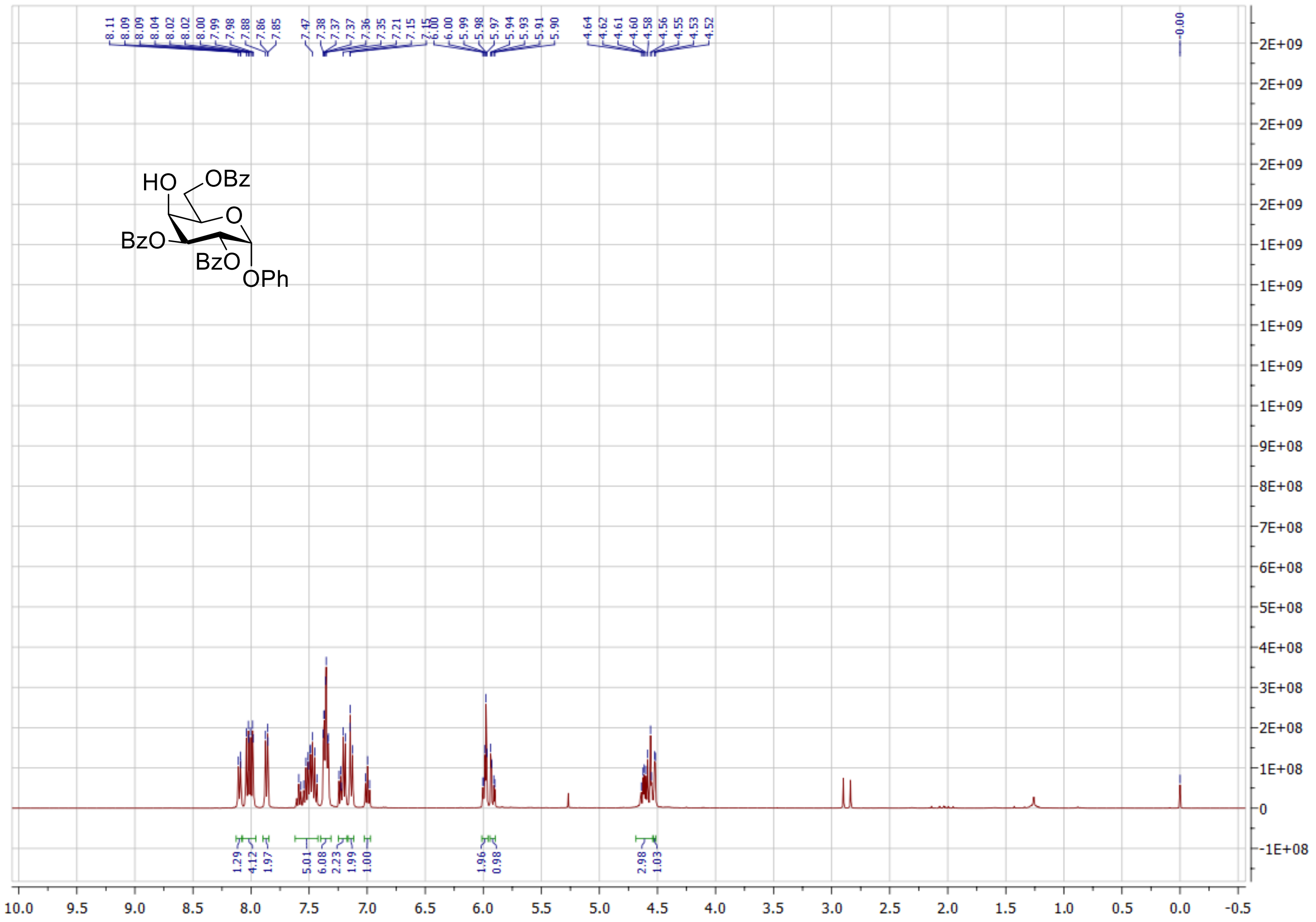

COSY (400 × 400 MHz, $CDCl_3$): Phenyl 2,3,6-tri-*O*-benzoyl-α-D-galactopyranoside 6

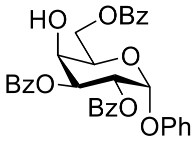

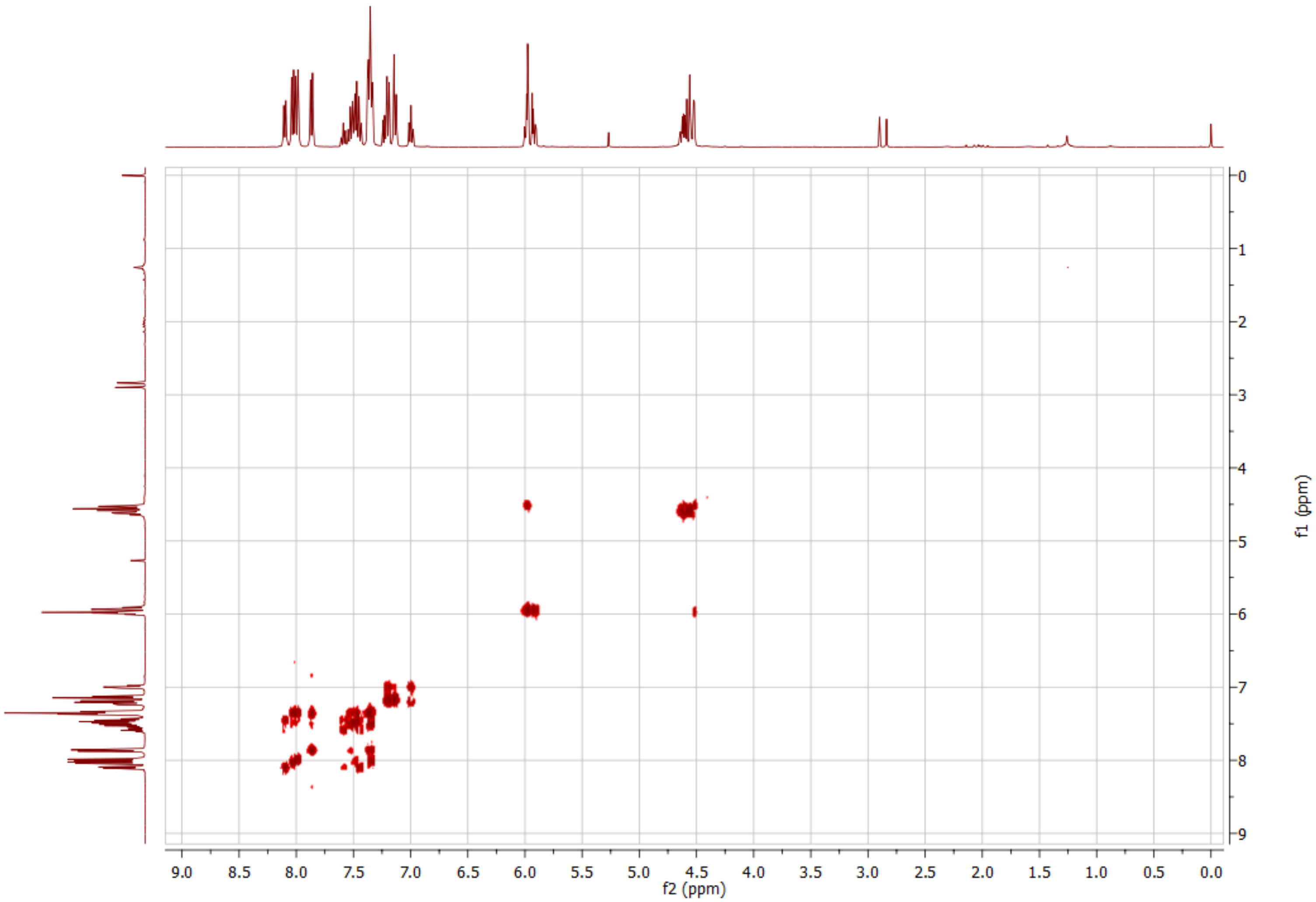

HSQC (CDCl$_3$): 400 × 101 MHz) Phenyl 2,3,6-tri-*O*-benzoyl-α-D-galactopyranoside 6

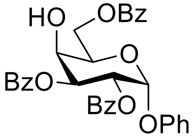

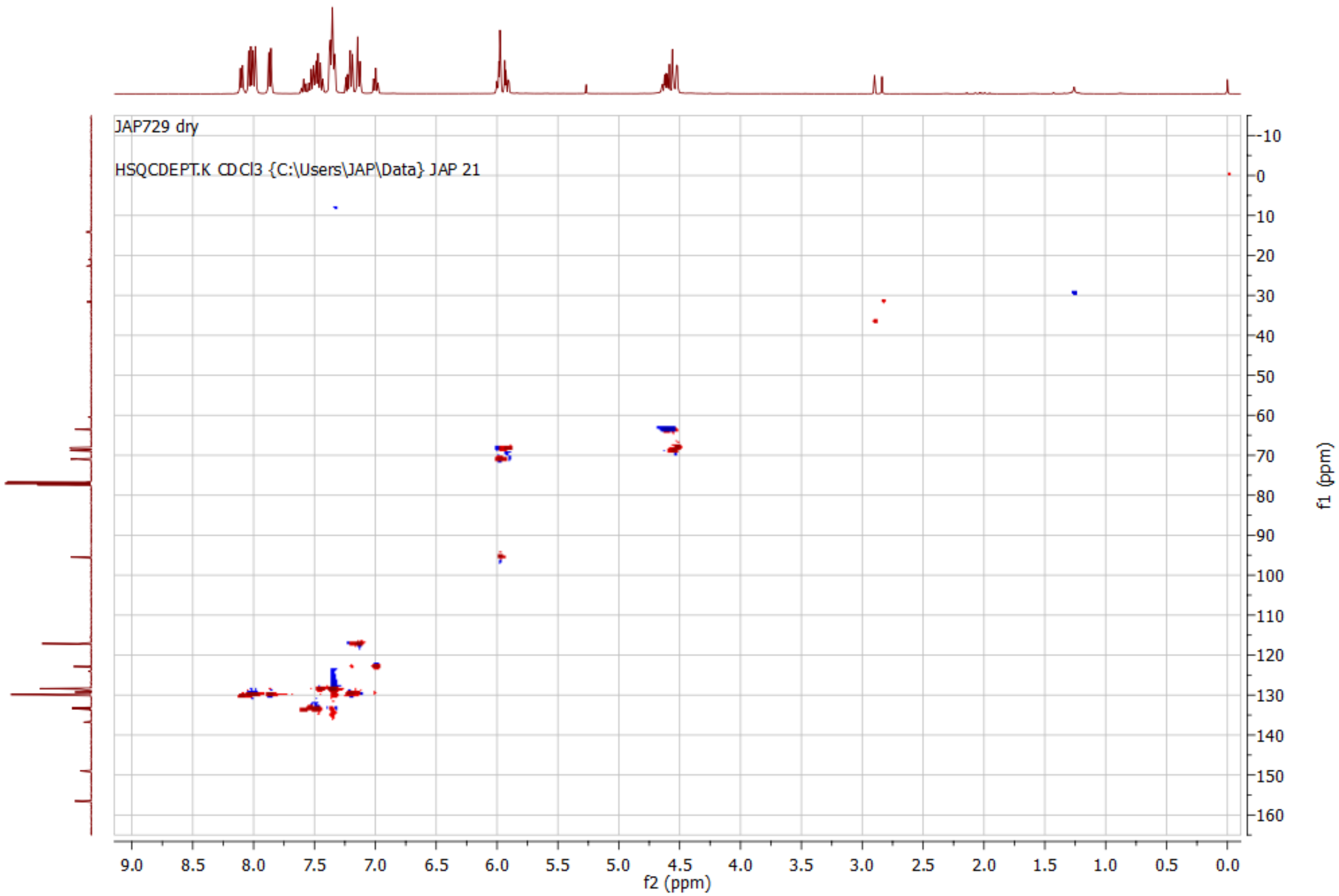

HMBC (400 × 101 MHz, CDCl₃): Phenyl 2,3,6-tri-*O*-benzoyl-α-D-galactopyranoside 6

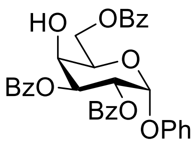

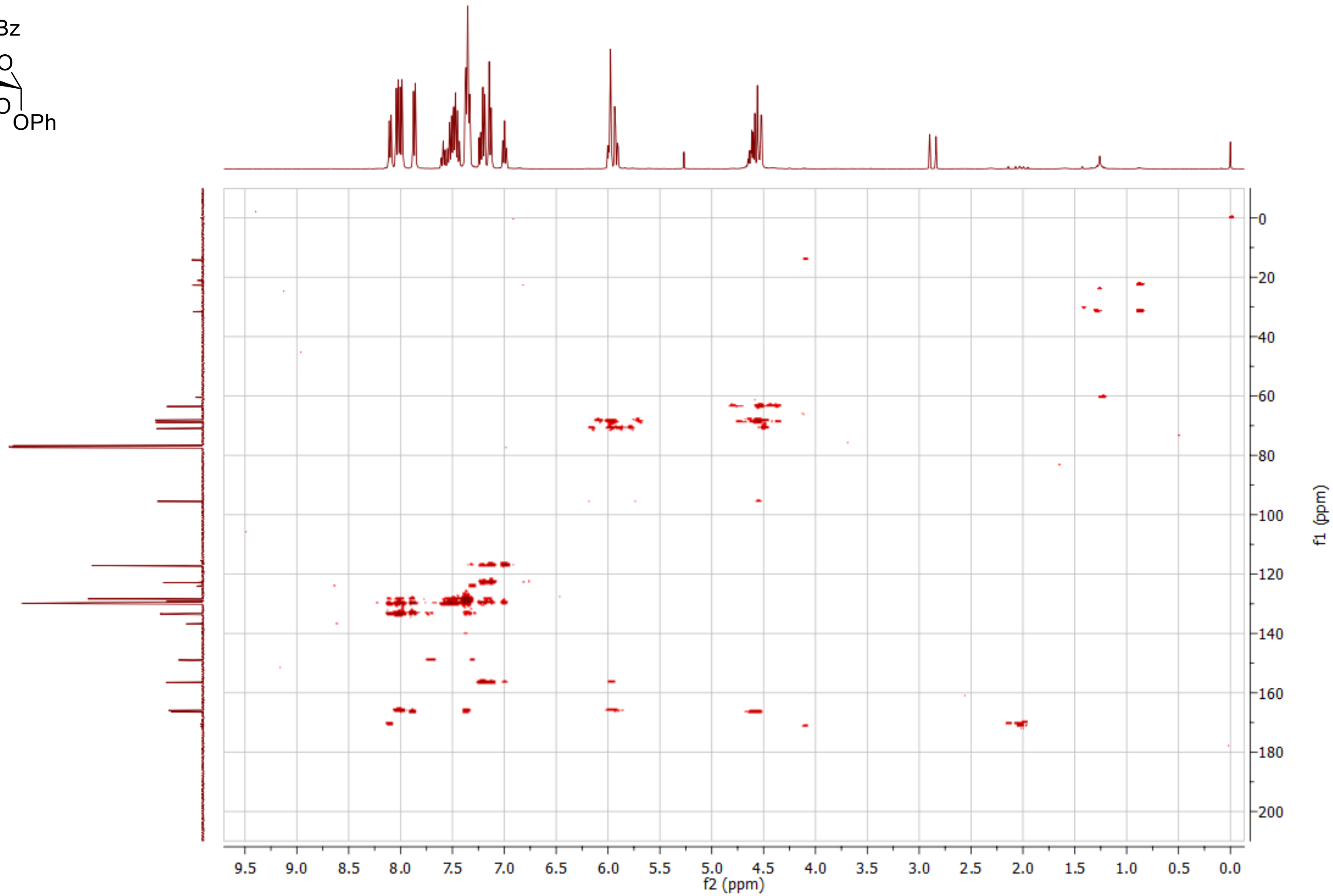

$^{13}C\{1H\}$ NMR (101 MHz, $CDCl_3$): Phenyl 2,3,6-tri-*O*-benzoyl-α-D-galactopyranoside 6

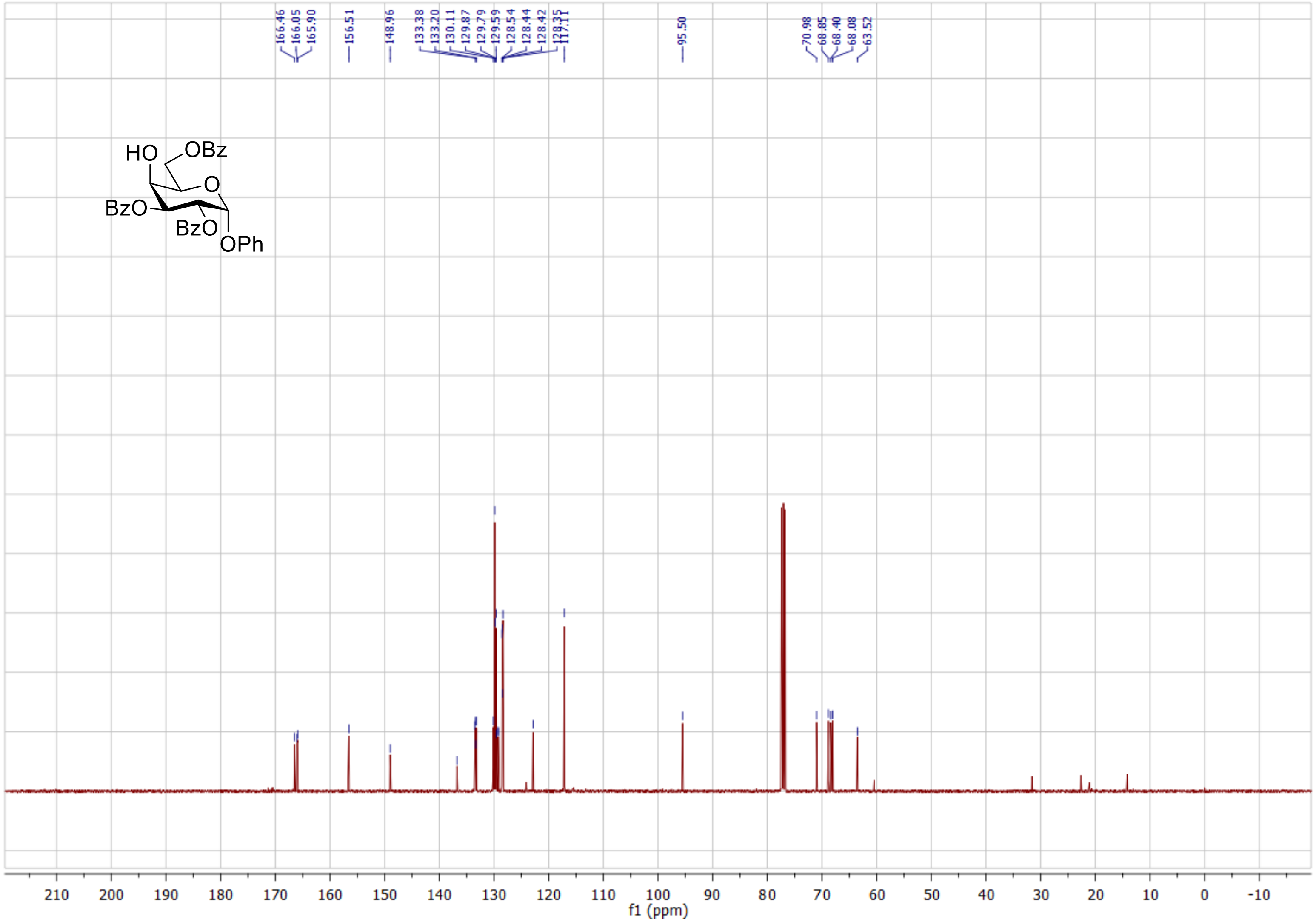

Compound 8

^{1}H NMR (400 MHz, CDCl₃): Ethyl 2,3,6-tri-*O*-benzoyl-α-D-galactopyranoside 8

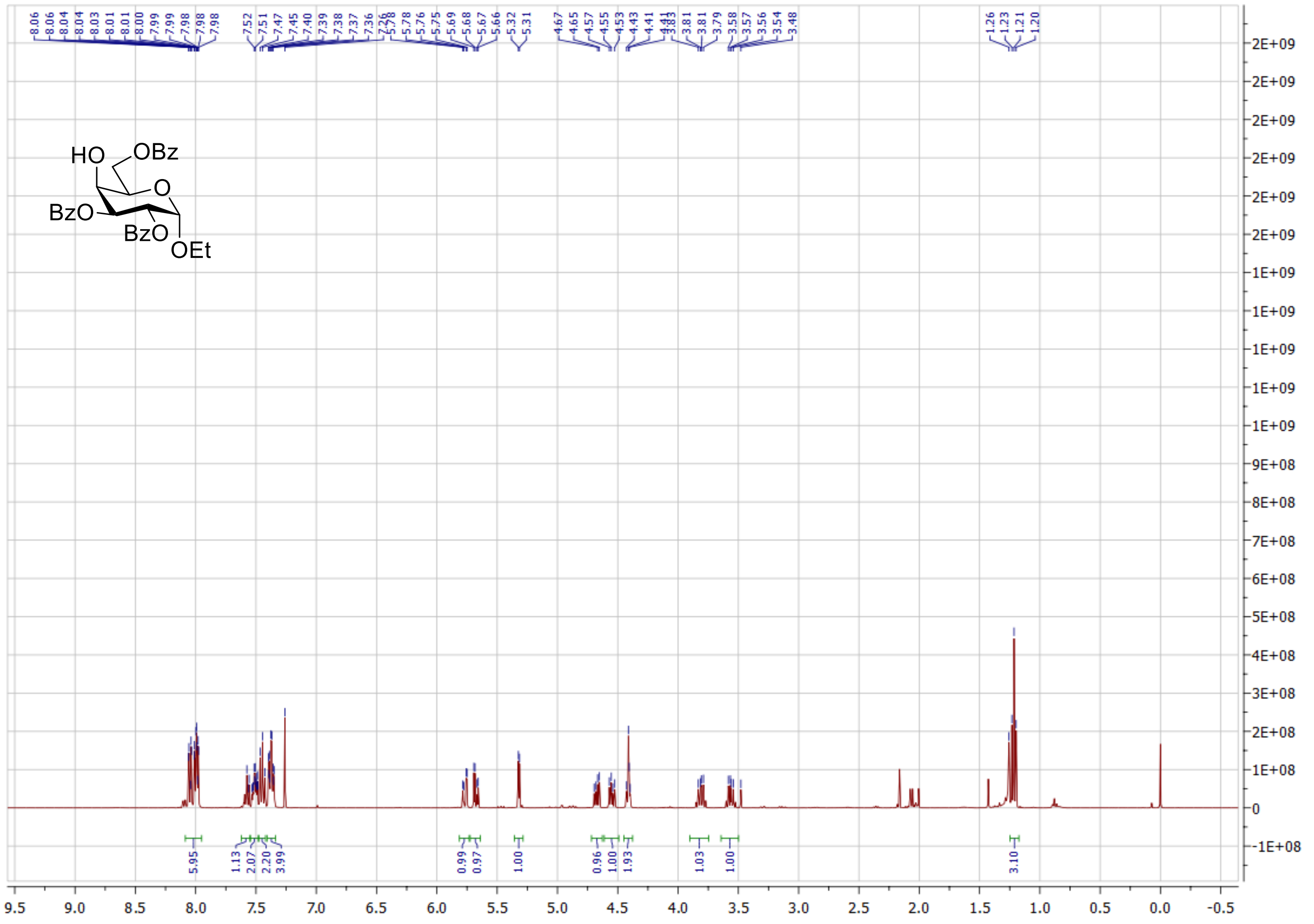

COSY (400 × 400 MHz, $CDCl_3$): Ethyl 2,3,6-tri-*O*-benzoyl-α-D-galactopyranoside 8

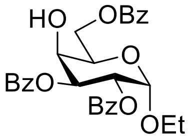

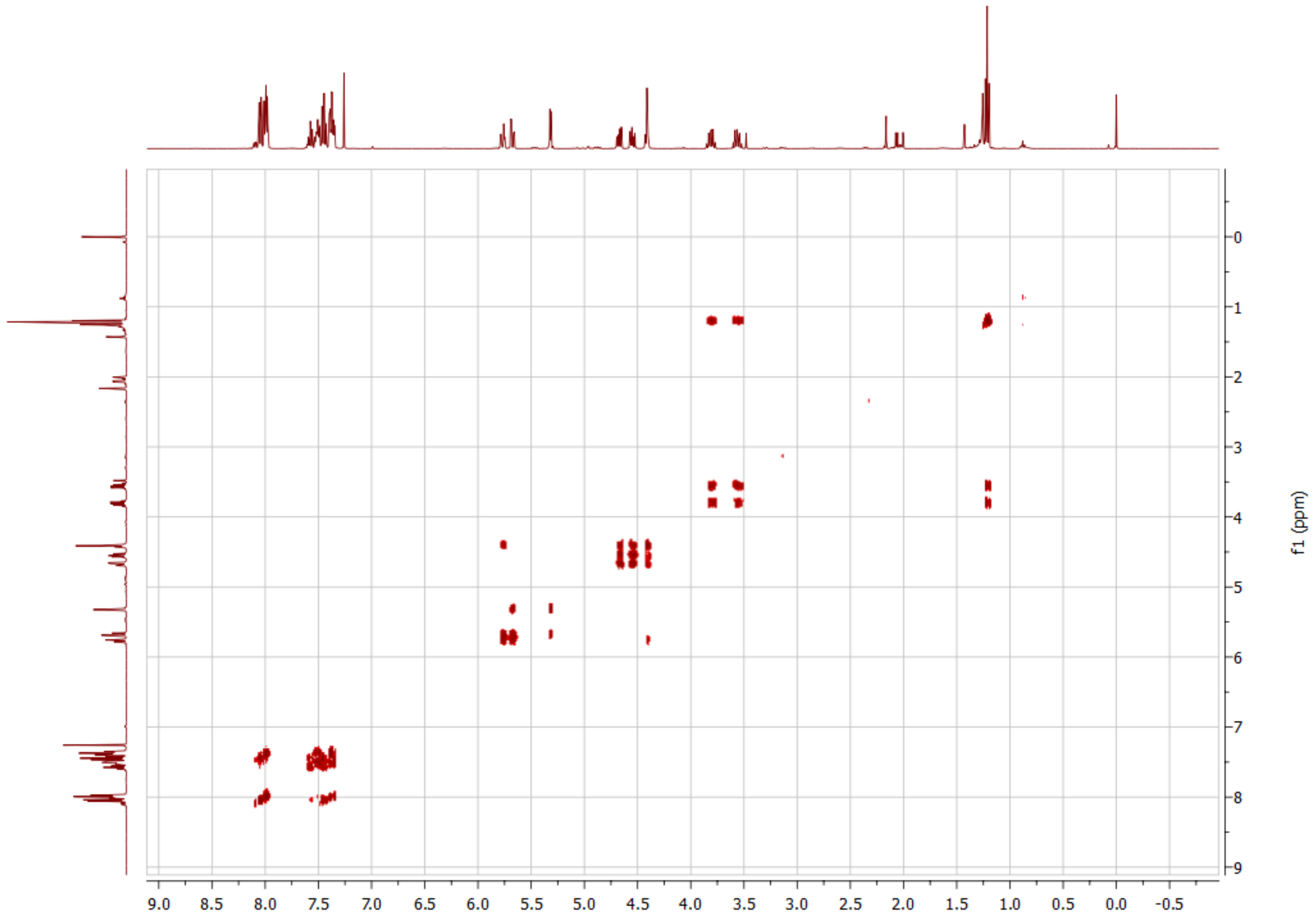

HSQC (400 × 101 MHz, $CDCl_3$): Ethyl 2,3,6-tri-*O*-benzoyl-α-D-galactopyranoside 8

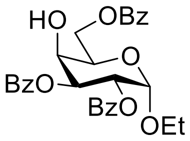

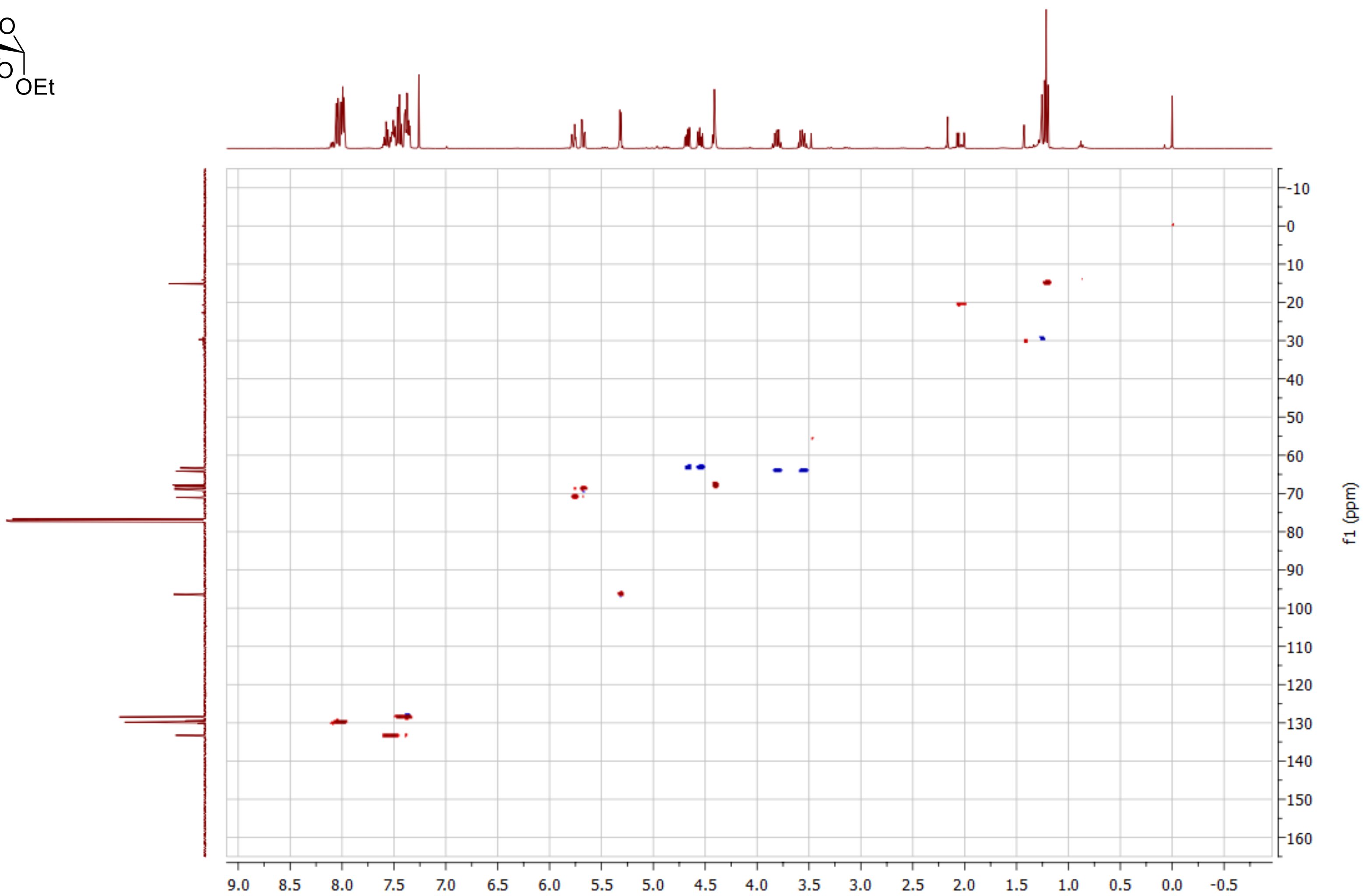

HMBC (400 × 101 MHz, CDCl₃): Ethyl 2,3,6-tri-*O*-benzoyl-α-D-galactopyranoside 8

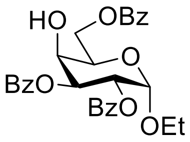

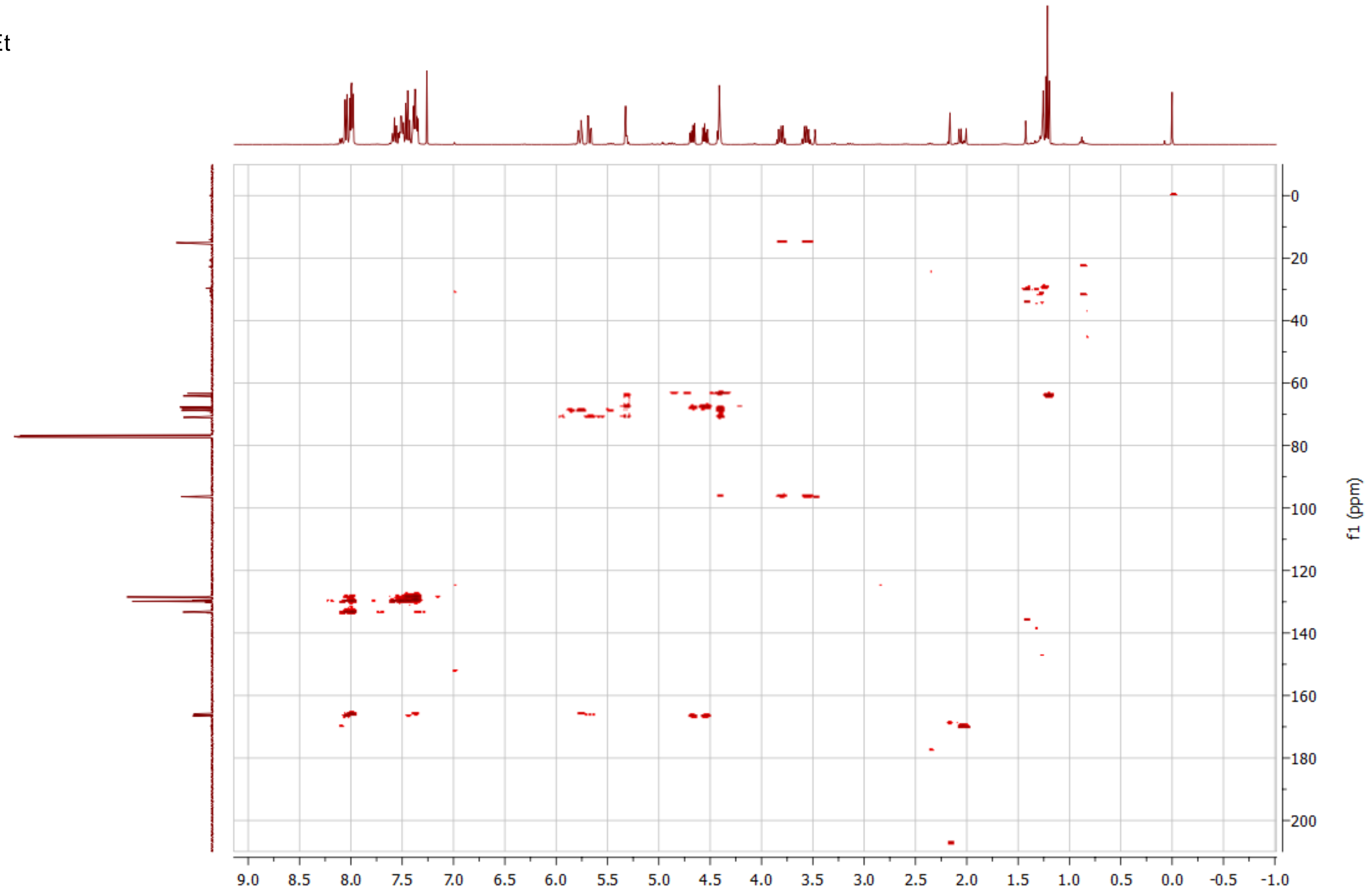

$^{13}C\{^1H\}$ NMR (101 MHz, $CDCl_3$): Ethyl 2,3,6-tri-*O*-benzoyl-α-D-galactopyranoside 8

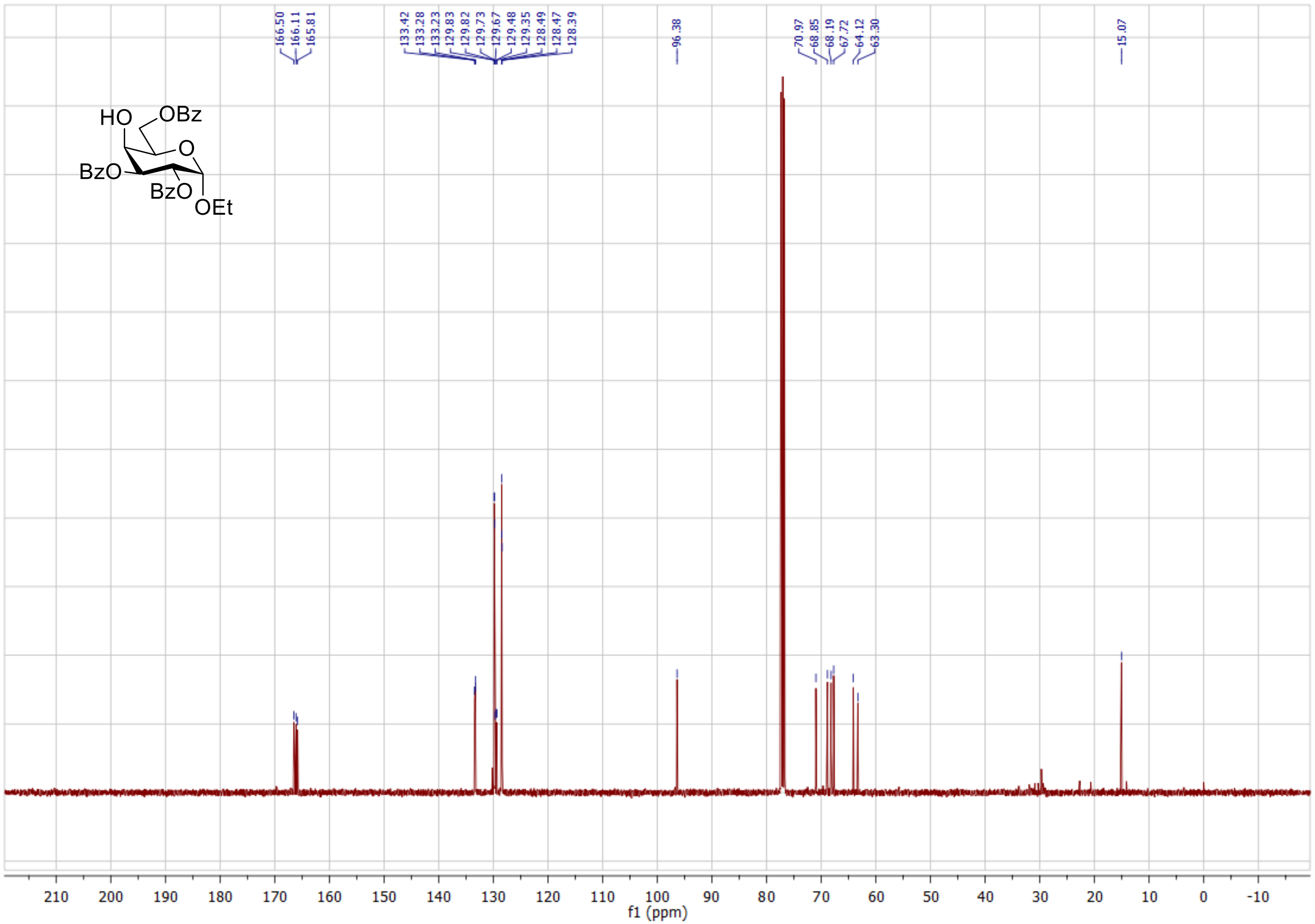

Compound 10

^{1}H NMR (400 MHz, CDCl₃): Propargyl 2,3,6-tri-*O*-benzoyl-α-D-galactopyranoside 10

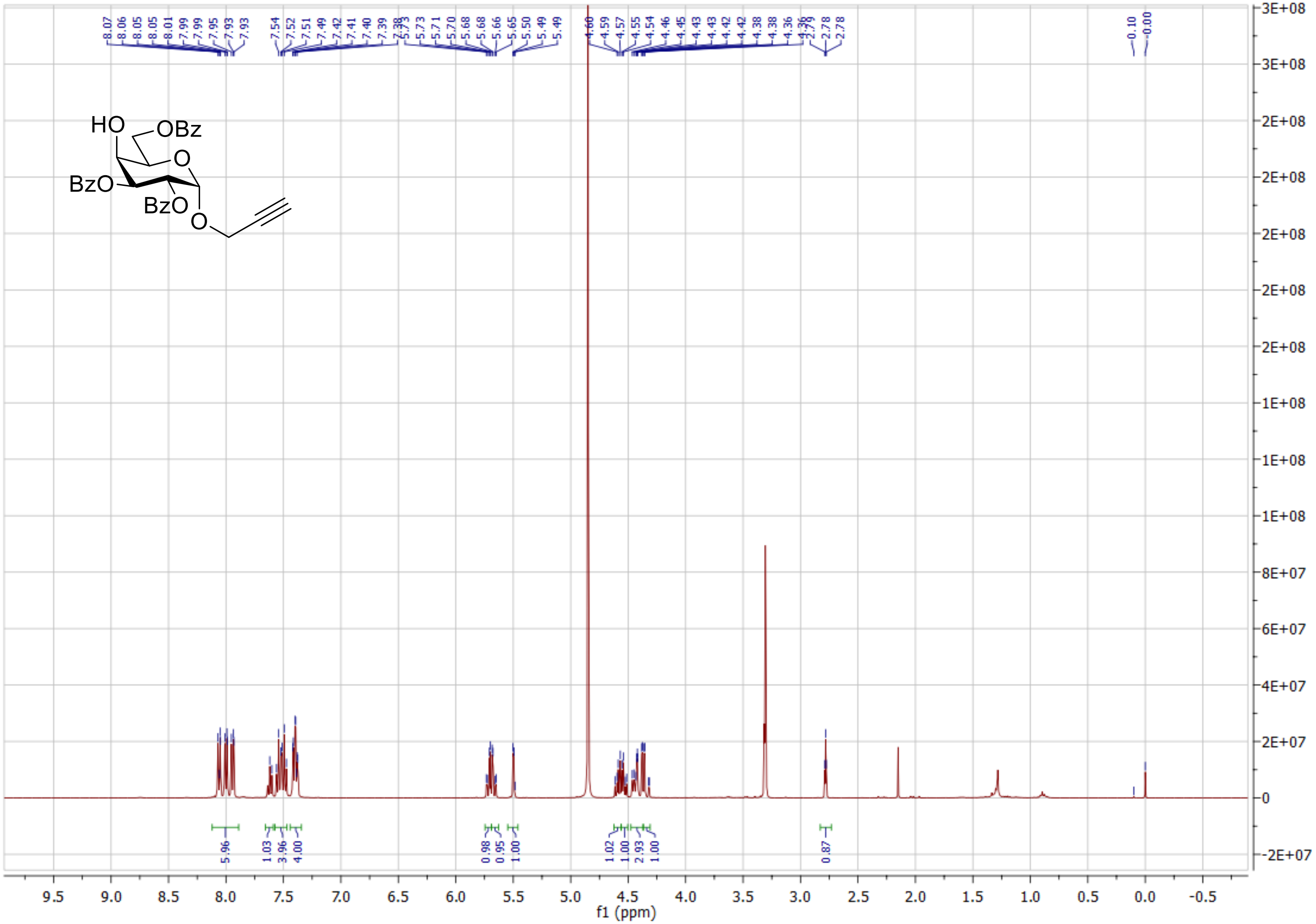

COSY (400 × 400 MHz, $CDCl_3$): Propargyl 2,3,6-tri-*O*-benzoyl-α-D-galactopyranoside 10

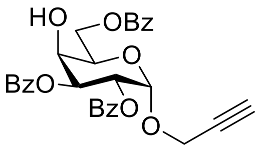

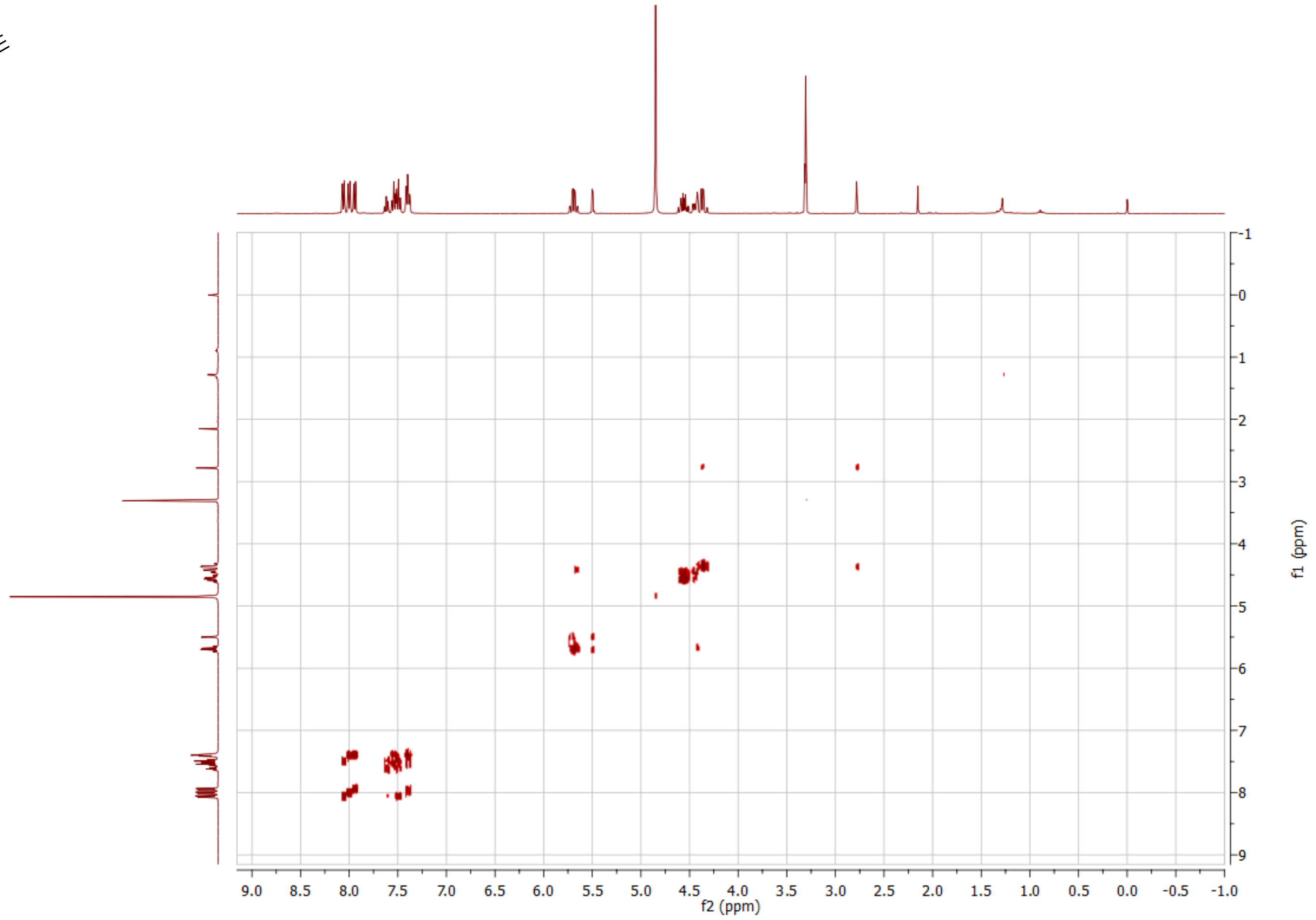

HSQC (400 × 101 MHz, CDCl₃): Propargyl 2,3,6-tri-*O*-benzoyl-α-D-galactopyranoside 10

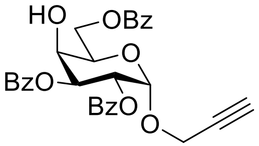

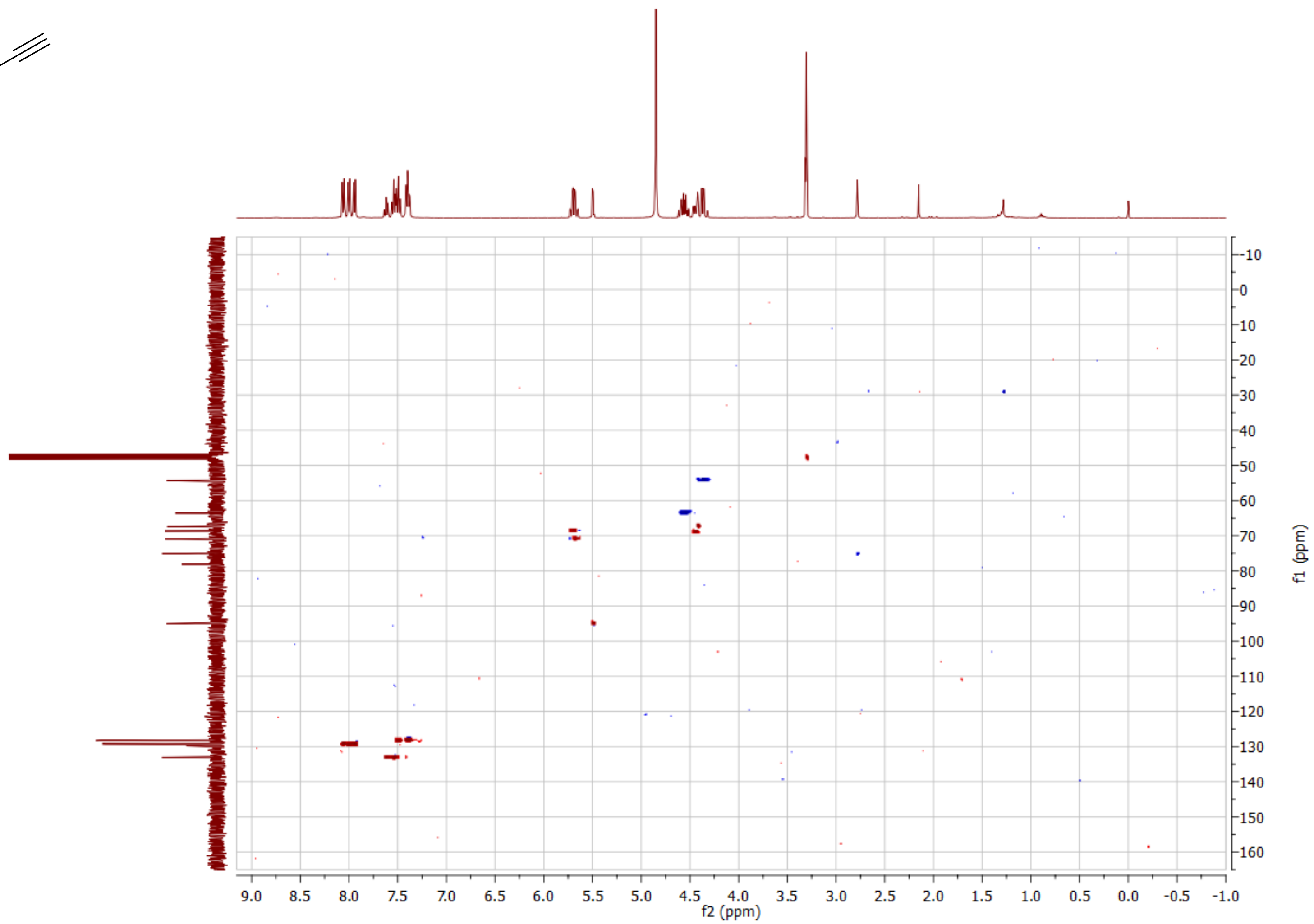

$^{13}C\{1H\}$ NMR (101 MHz, $CDCl_3$): Propargyl 2,3,6-tri-*O*-benzoyl-α-D-galactopyranoside 10

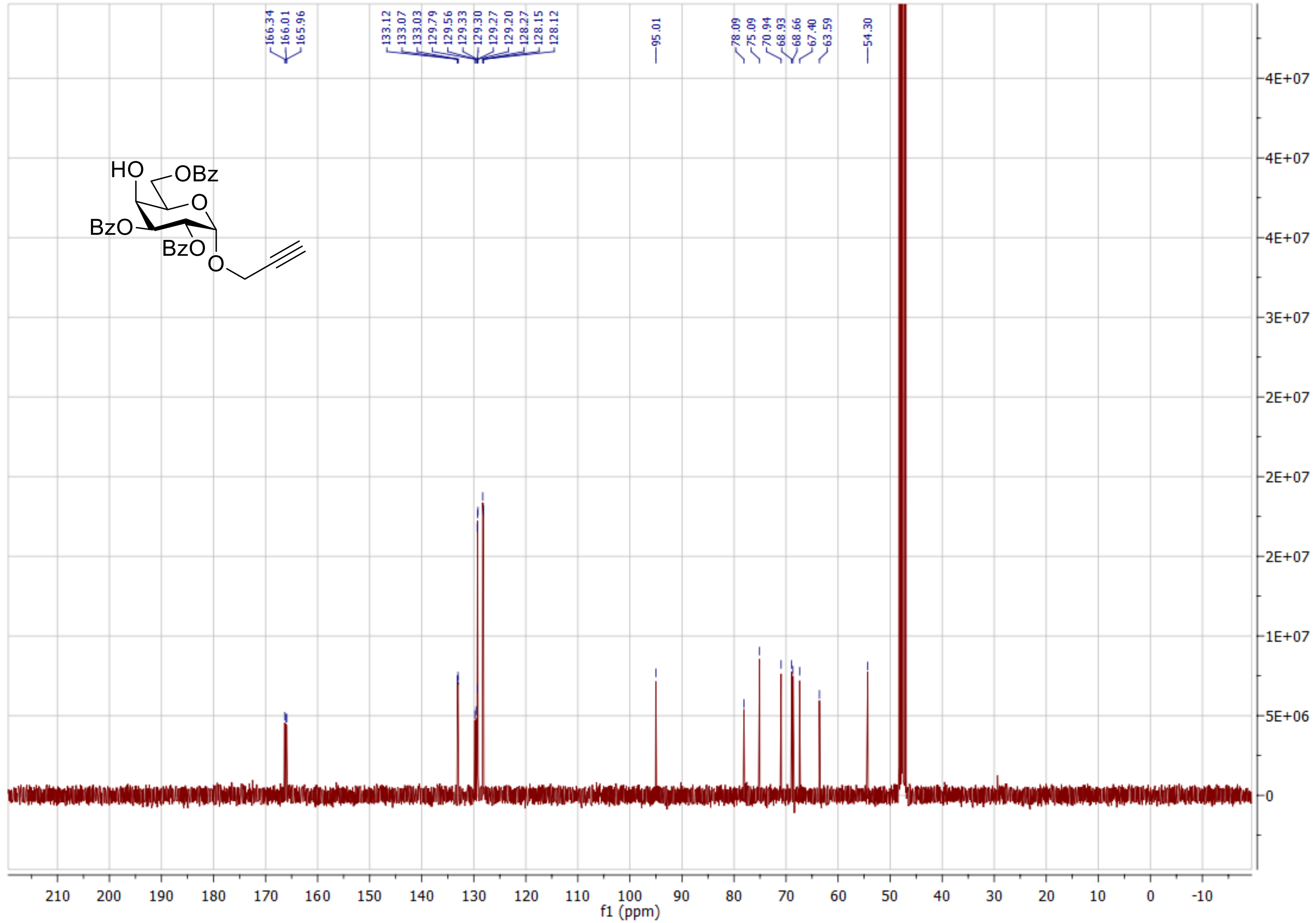

HMBC (400 × 101 MHz, CDCl3): Propargyl 2,3,6-tri-*O*-benzoyl-α-D-galactopyranoside 10

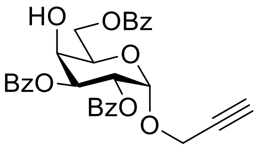

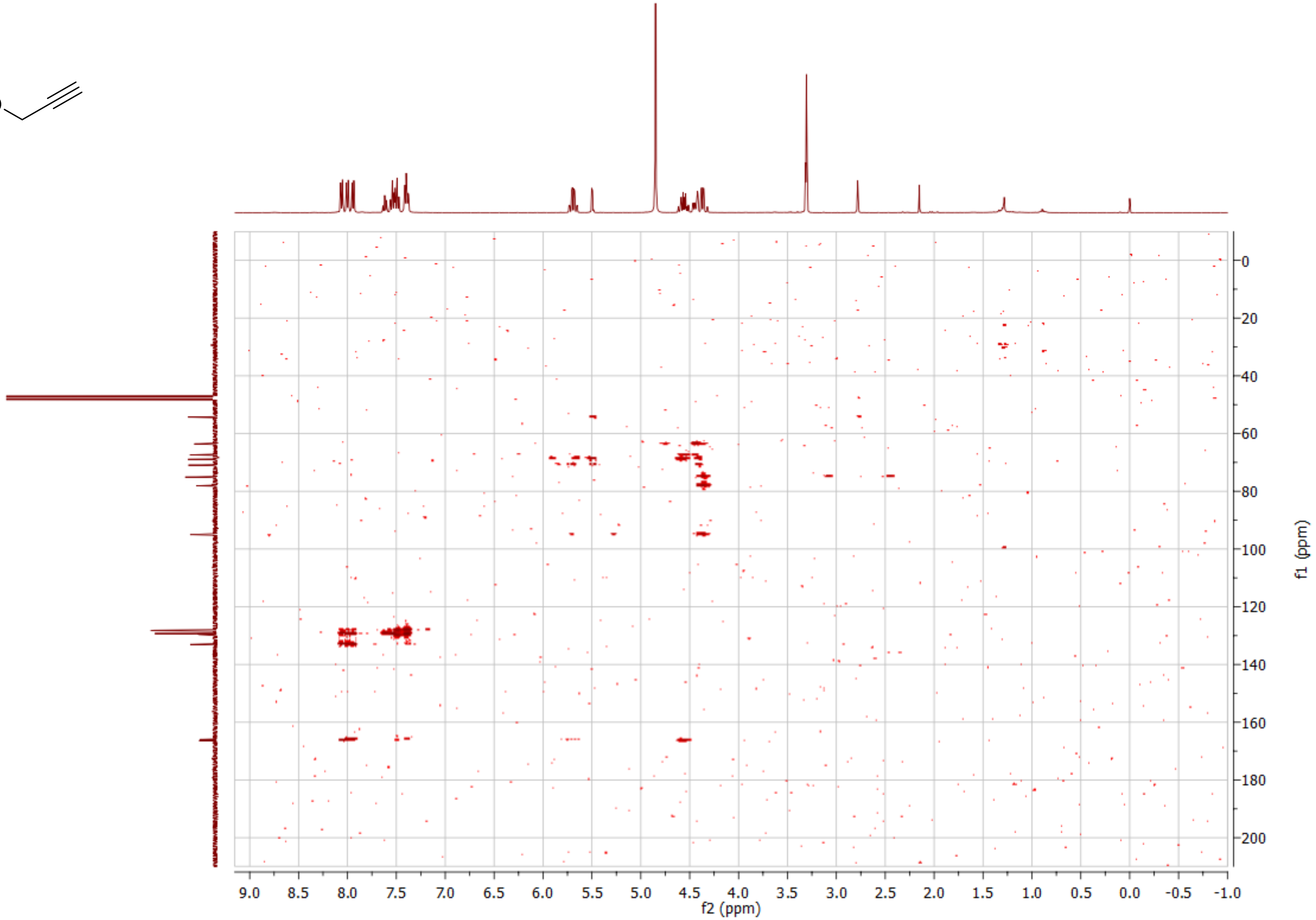

Compound 12

^{1}H NMR (400 MHz, MeOD): Methyl 2,3-di-*O*-benzoyl-6-*O*-triisopropylsilyl-α-D-galactopyranoside 12

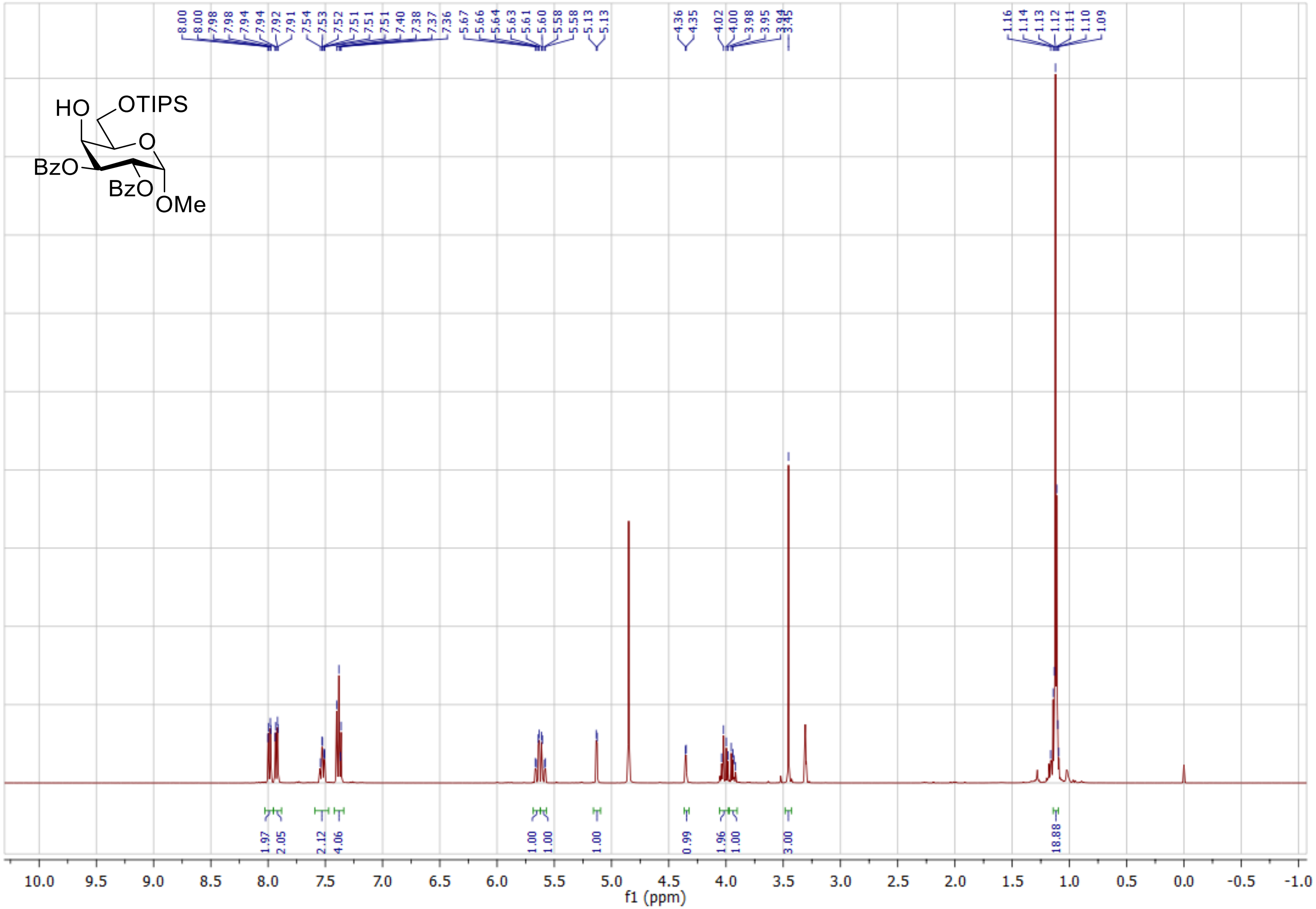

COSY (400 × 400 MHz, MeOD): Methyl 2,3-di-*O*-benzoyl-6-*O*-triisopropylsilyl-α-D-galactopyranoside 12

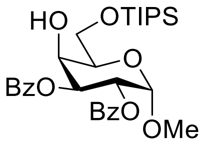

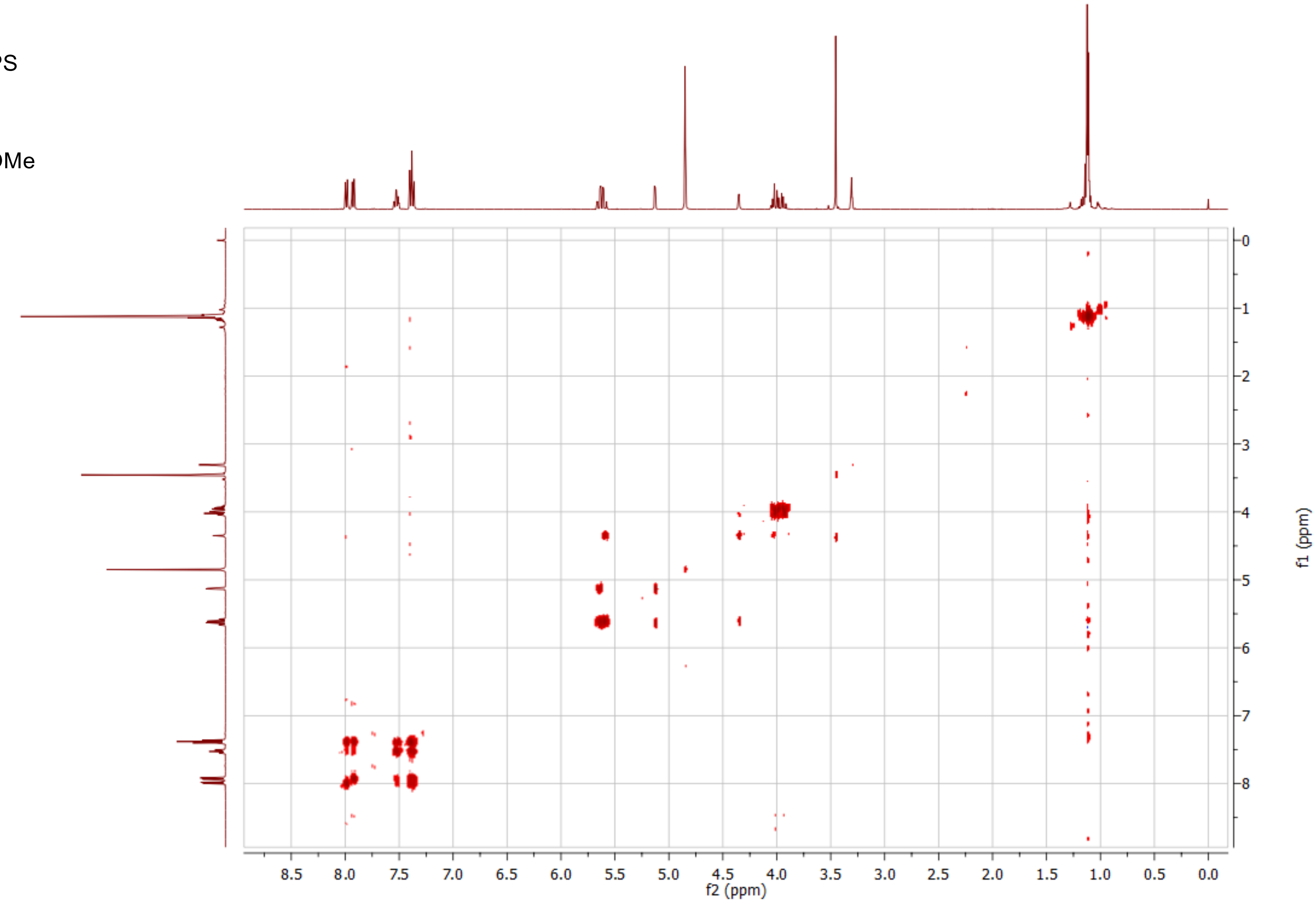

HSQC (400 × 101 MHz, MeOD): Methyl 2,3-di-*O*-benzoyl-6-*O*-triisopropylsilyl-α-D-galactopyranoside 12

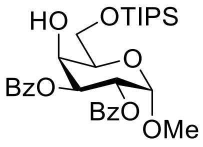

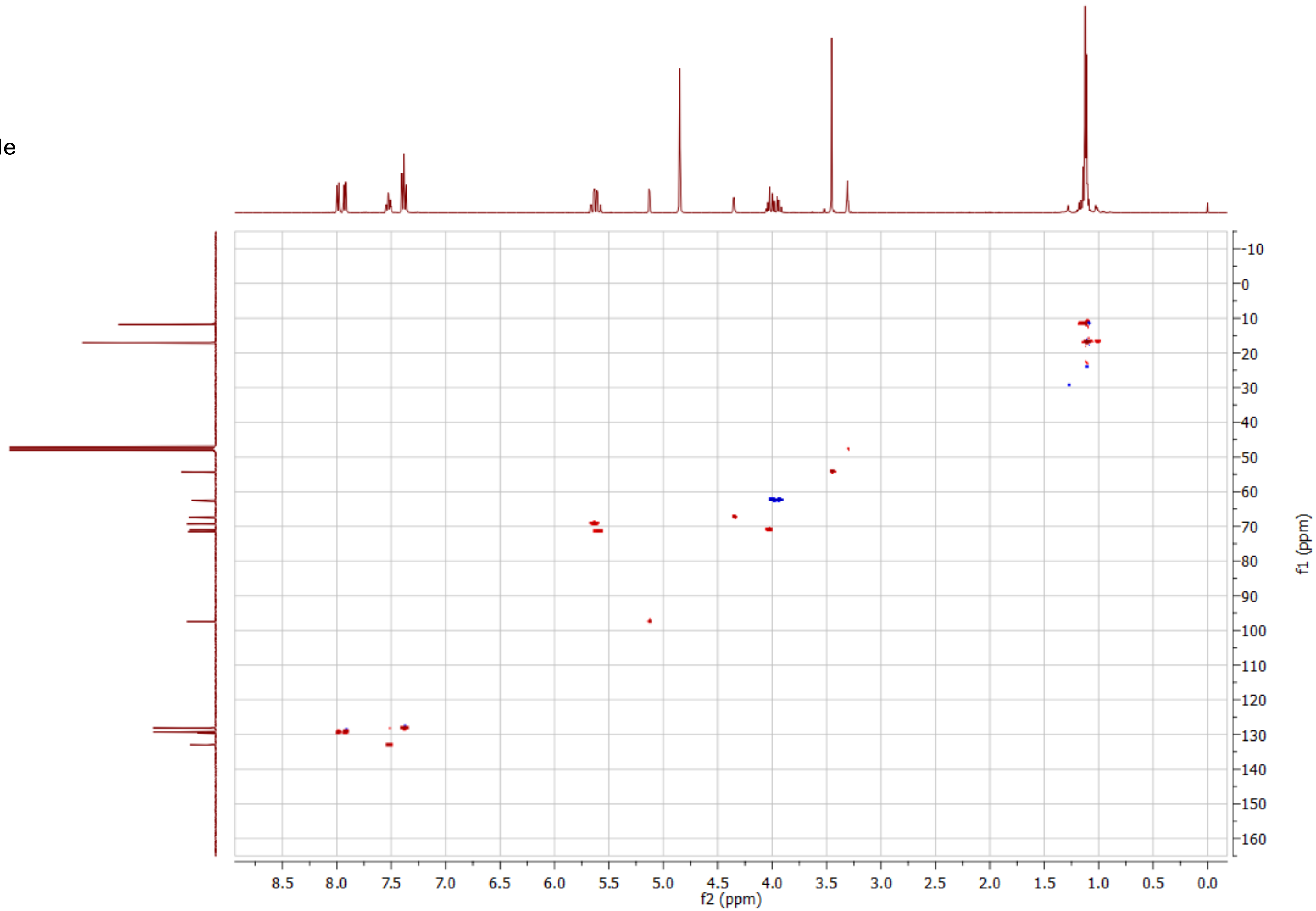

HMBC (400 × 101 MHz, MeOD): Methyl 2,3-di-*O*-benzoyl-6-*O*-triisopropylsilyl-α-D-galactopyranoside 12

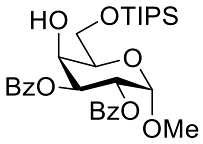

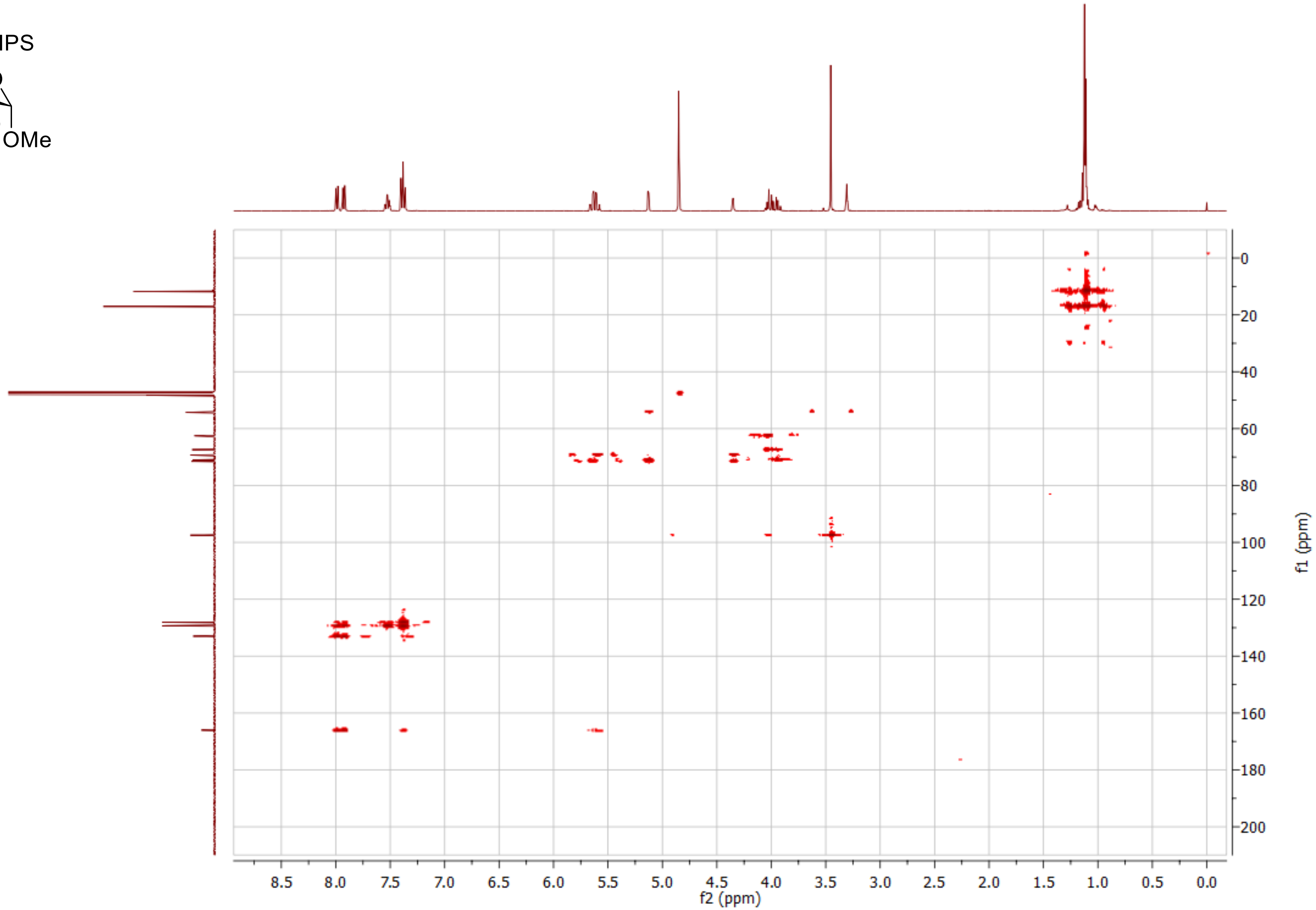

$^{13}C\{1H\}$ NMR (101 MHz, MeOD): Methyl 2,3-di-*O*-benzoyl-6-*O*-triisopropylsilyl-α-D-galactopyranoside 12

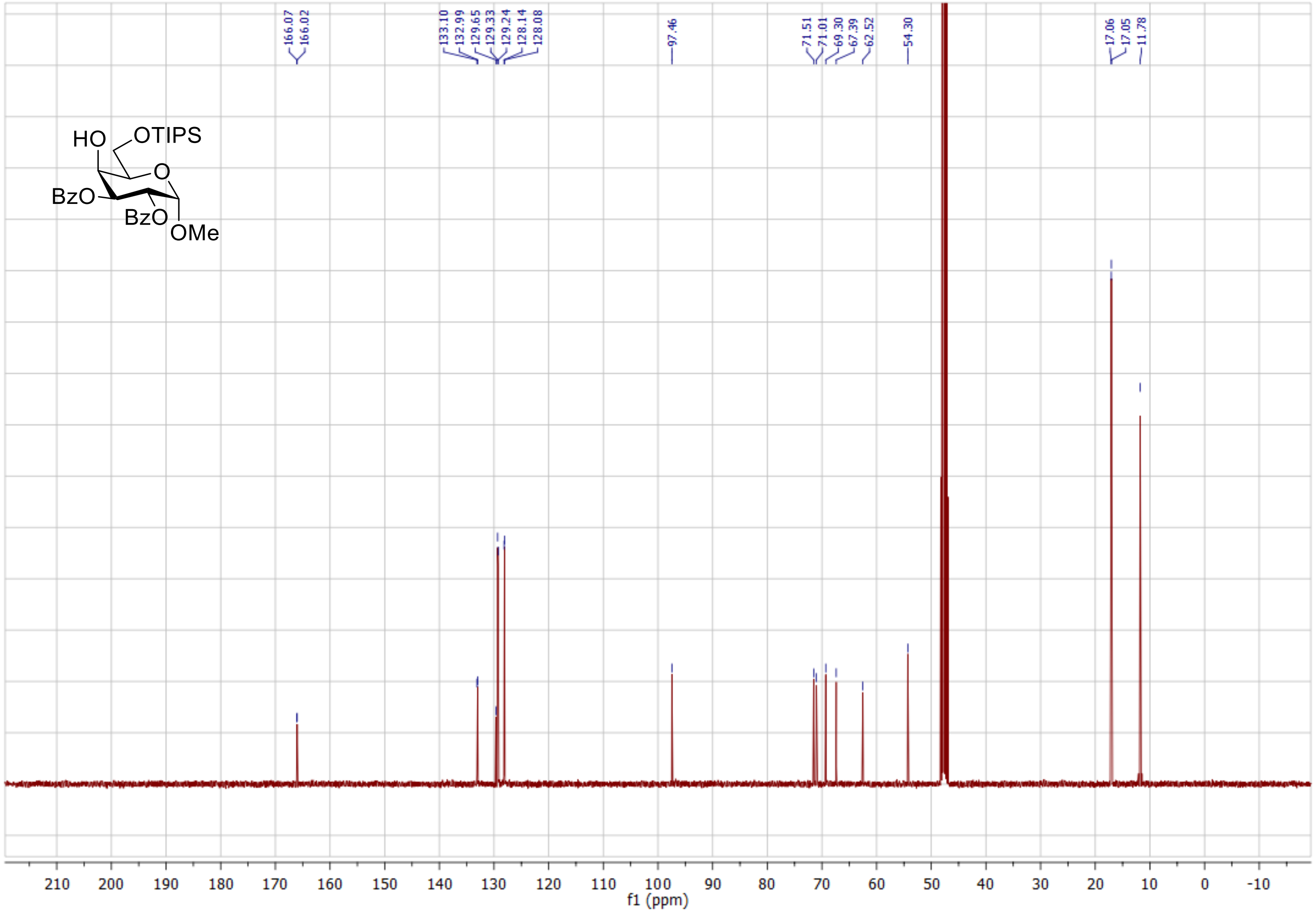

Compound 14

^{1}H NMR (400 MHz, CDCl$_3$): ***p*-Methoxy phenyl 2,3,6-tri-*O*-benzoyl-α-D-galactopyranoside 14**

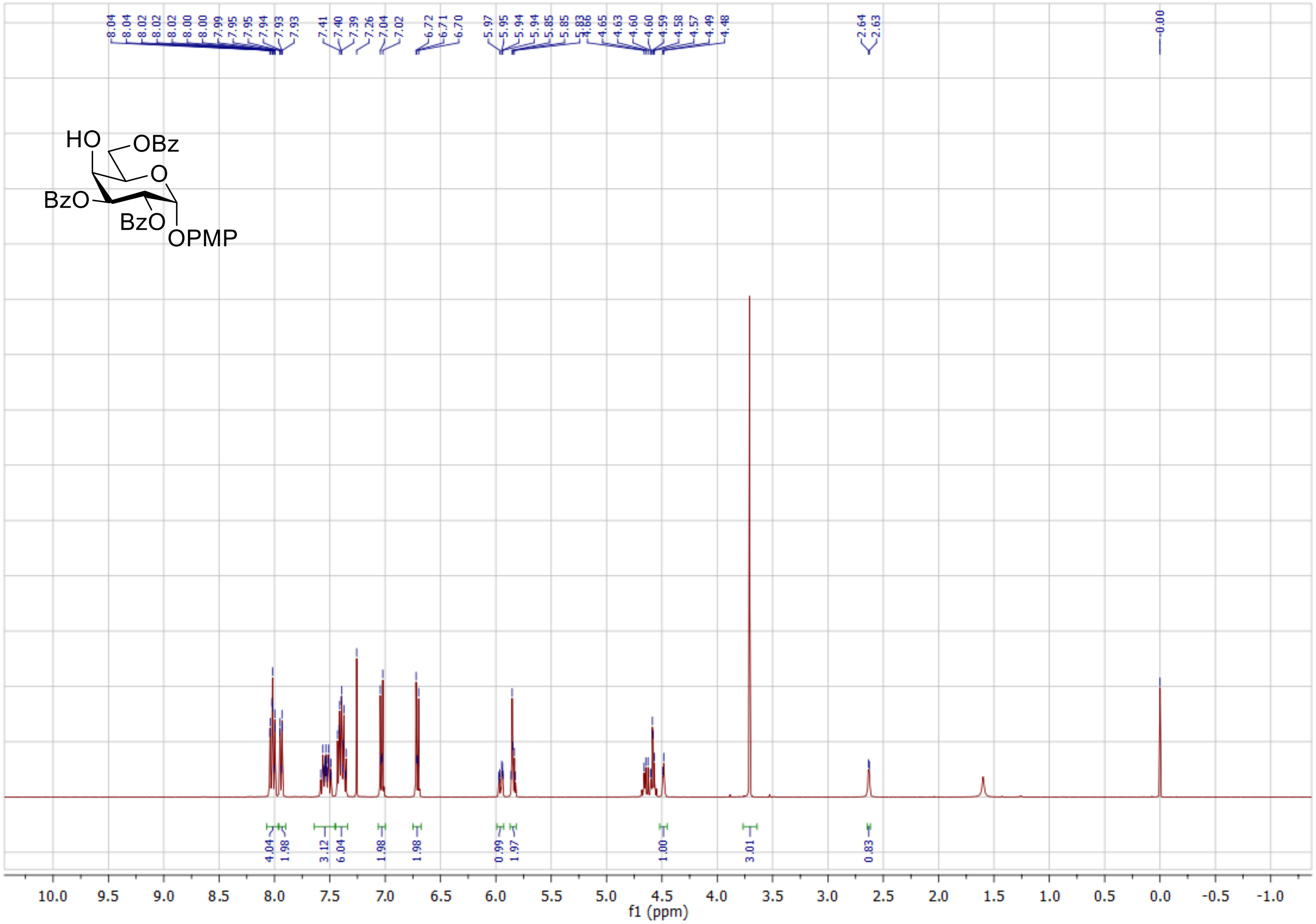

COSY (400 × 400 MHz, $CDCl_3$): ***p*-Methoxy phenyl 2,3,6-tri-*O*-benzoyl-α-D-galactopyranoside 14**

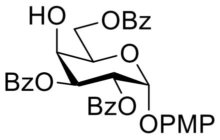

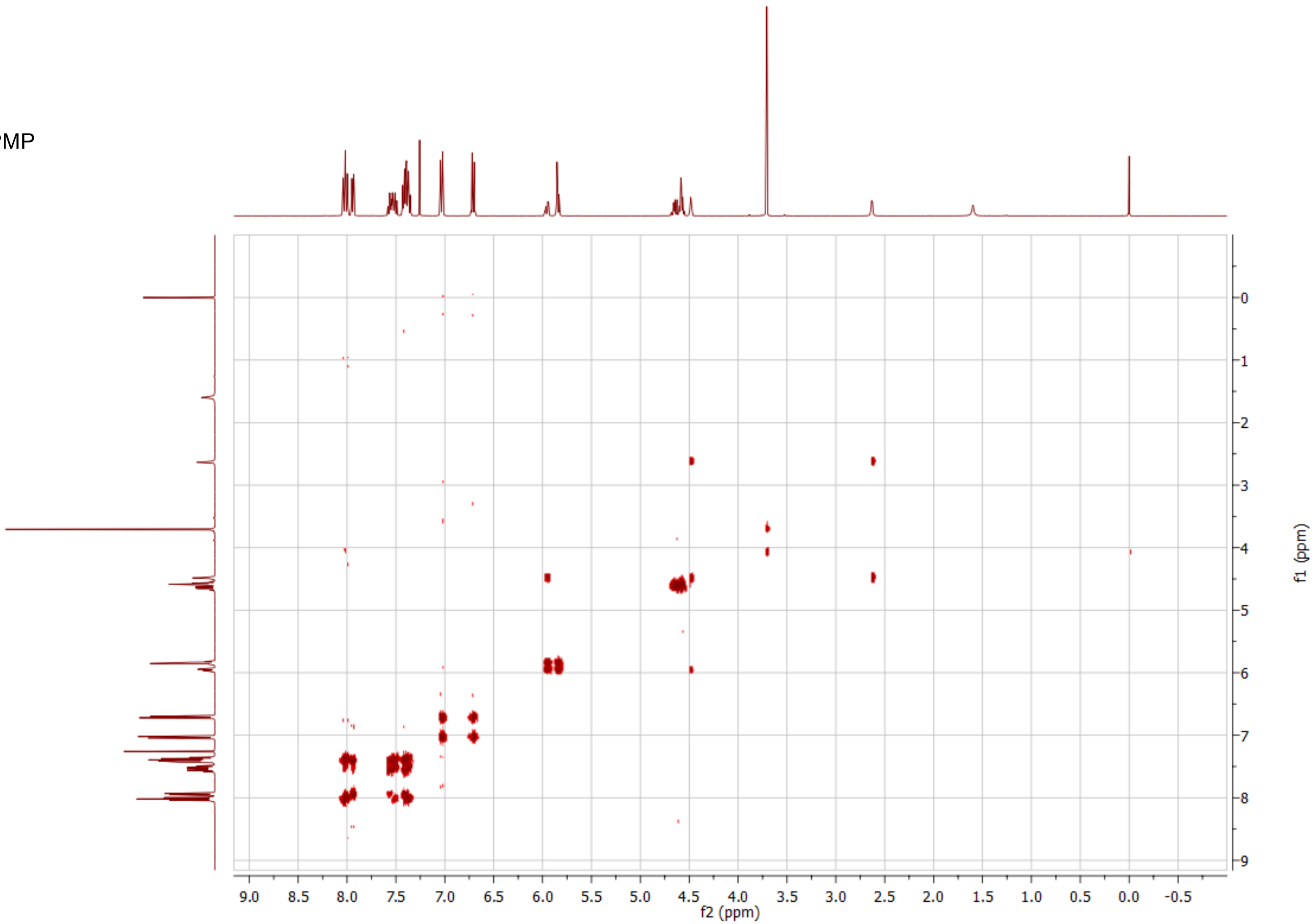

HSQC (101 × 400 MHz, CDCl₃): ***p*-Methoxy phenyl 2,3,6-tri-*O*-benzoyl-α-D-galactopyranoside 14**

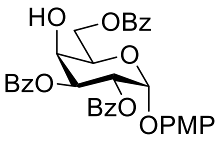

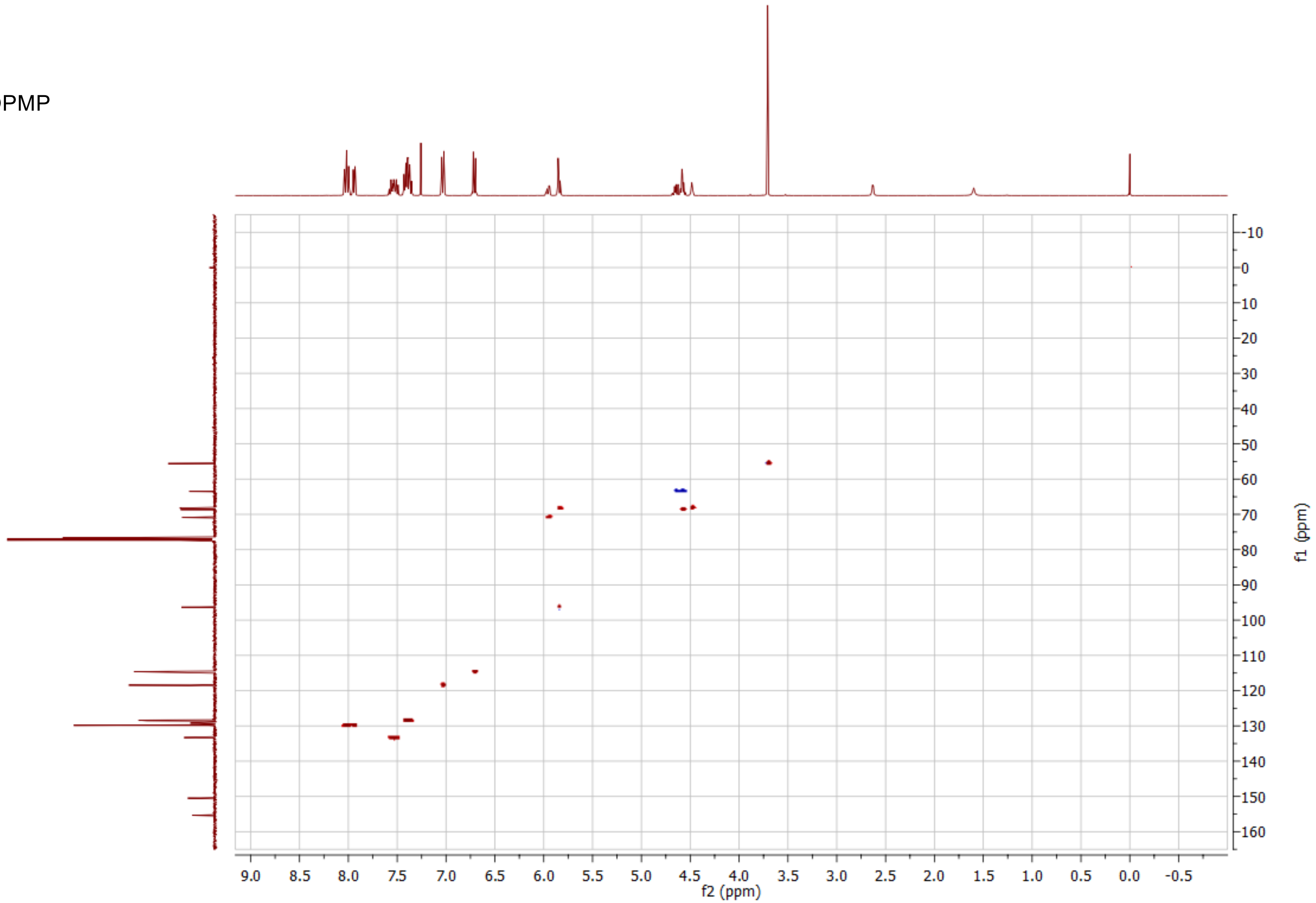

HMBC (101 × 400 MHz, $CDCl_3$): *p*-Methoxy phenyl 2,3,6-tri-*O*-benzoyl-α-D-galactopyranoside 14

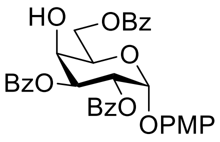

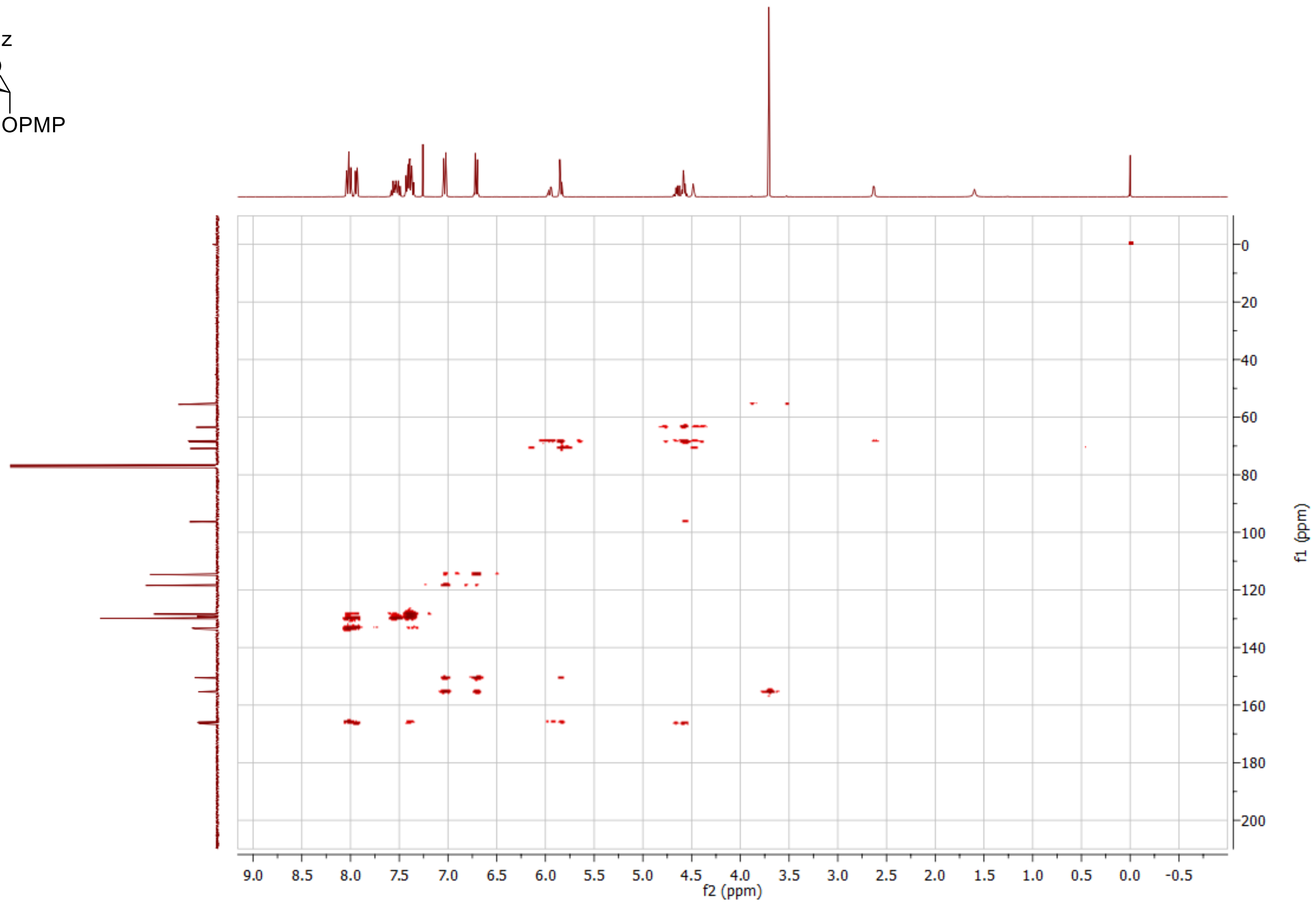

^{13}C{1H} NMR (101 MHz, CDCl₃): ***p*-Methoxy phenyl 2,3,6-tri-*O*-benzoyl-α-D-galactopyranoside 14**

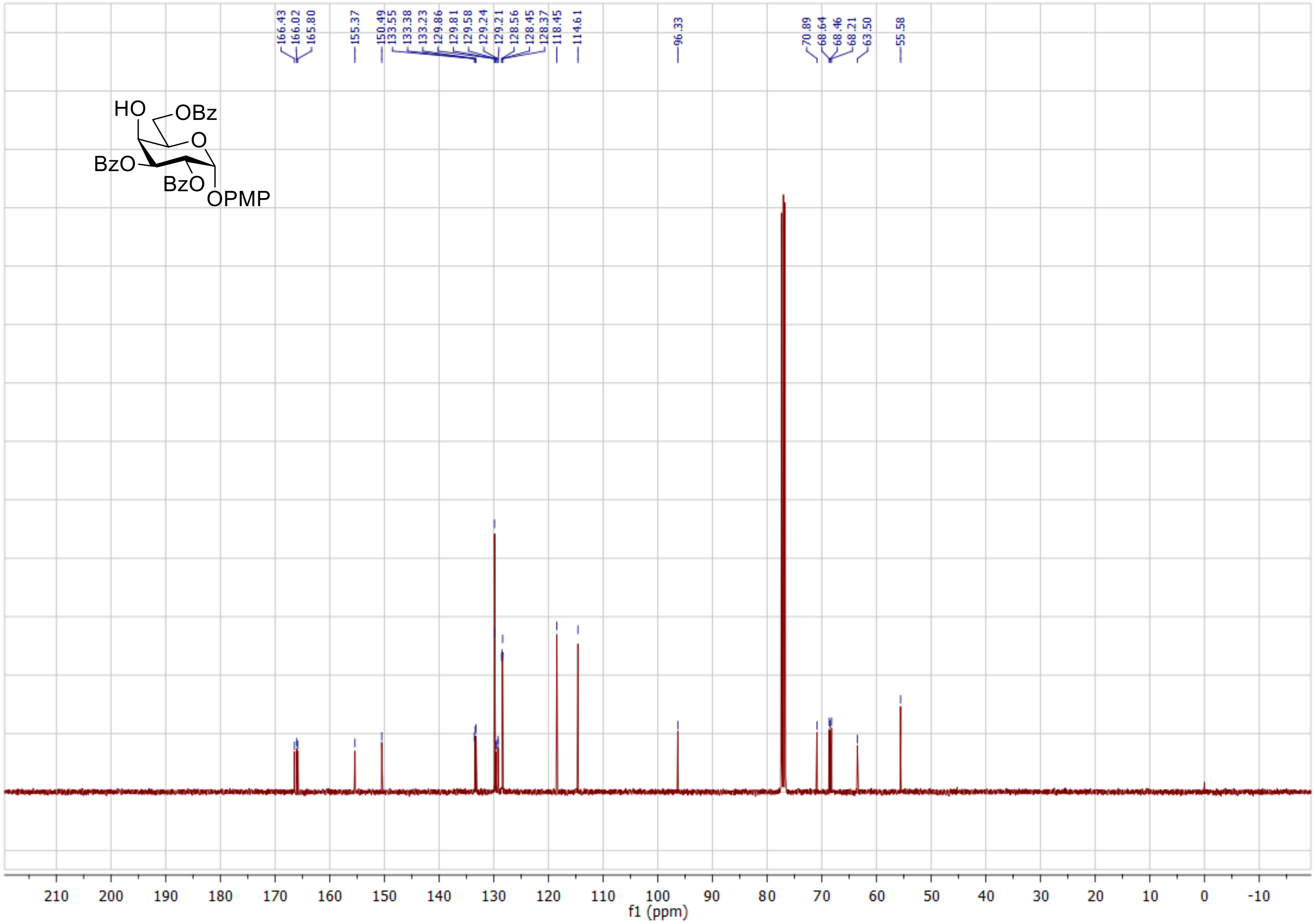

Compound 16

^{1}H NMR (400 MHz MeOD): Allyl 3,6-di-*O*-benzoyl-2-acetamido-2-deoxy-β-D-galactopyranoside 16

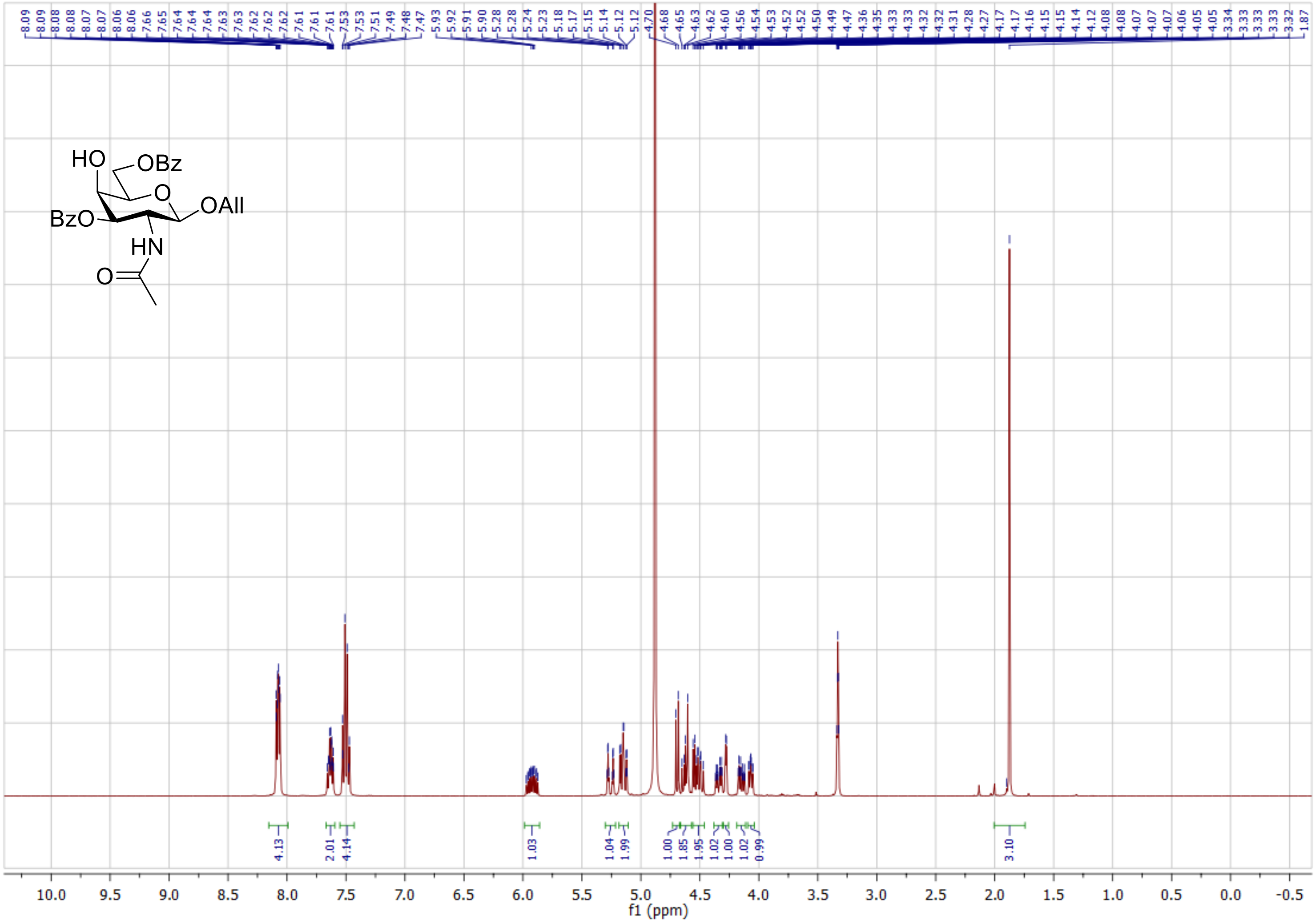

COSY (400 × 400 MHz MeOD): Allyl 3,6-di-*O*-benzoyl-2-acetamido-2-deoxy-β-D-galactopyranoside 16

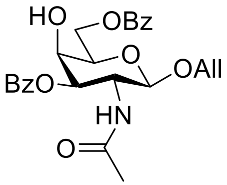

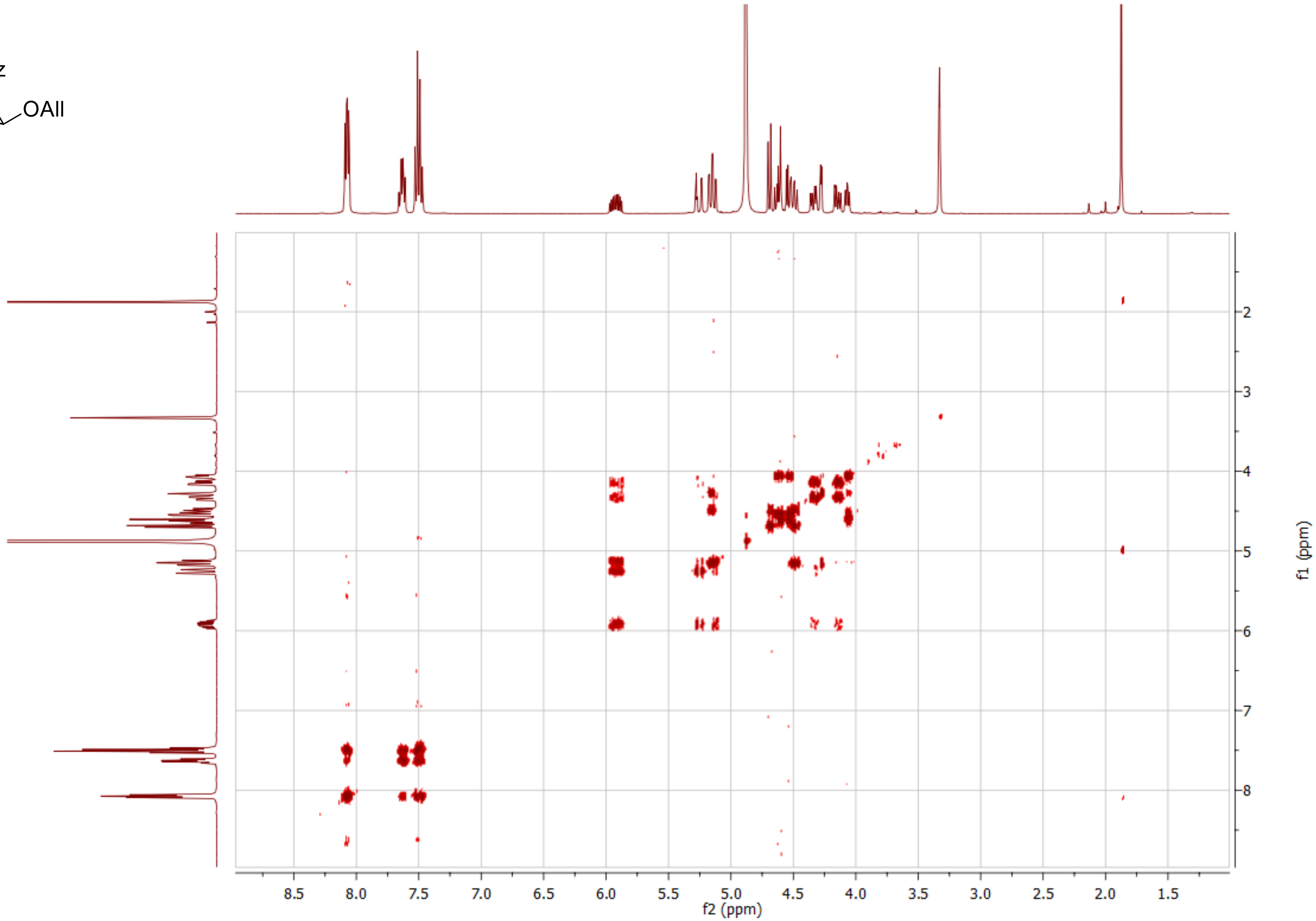

HSQC (400 × 101 MHz MeOD): Allyl 3,6-di-*O*-benzoyl-2-acetamido-2-deoxy-β-D-galactopyranoside 16

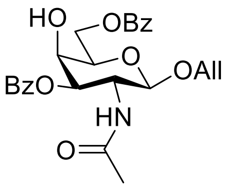

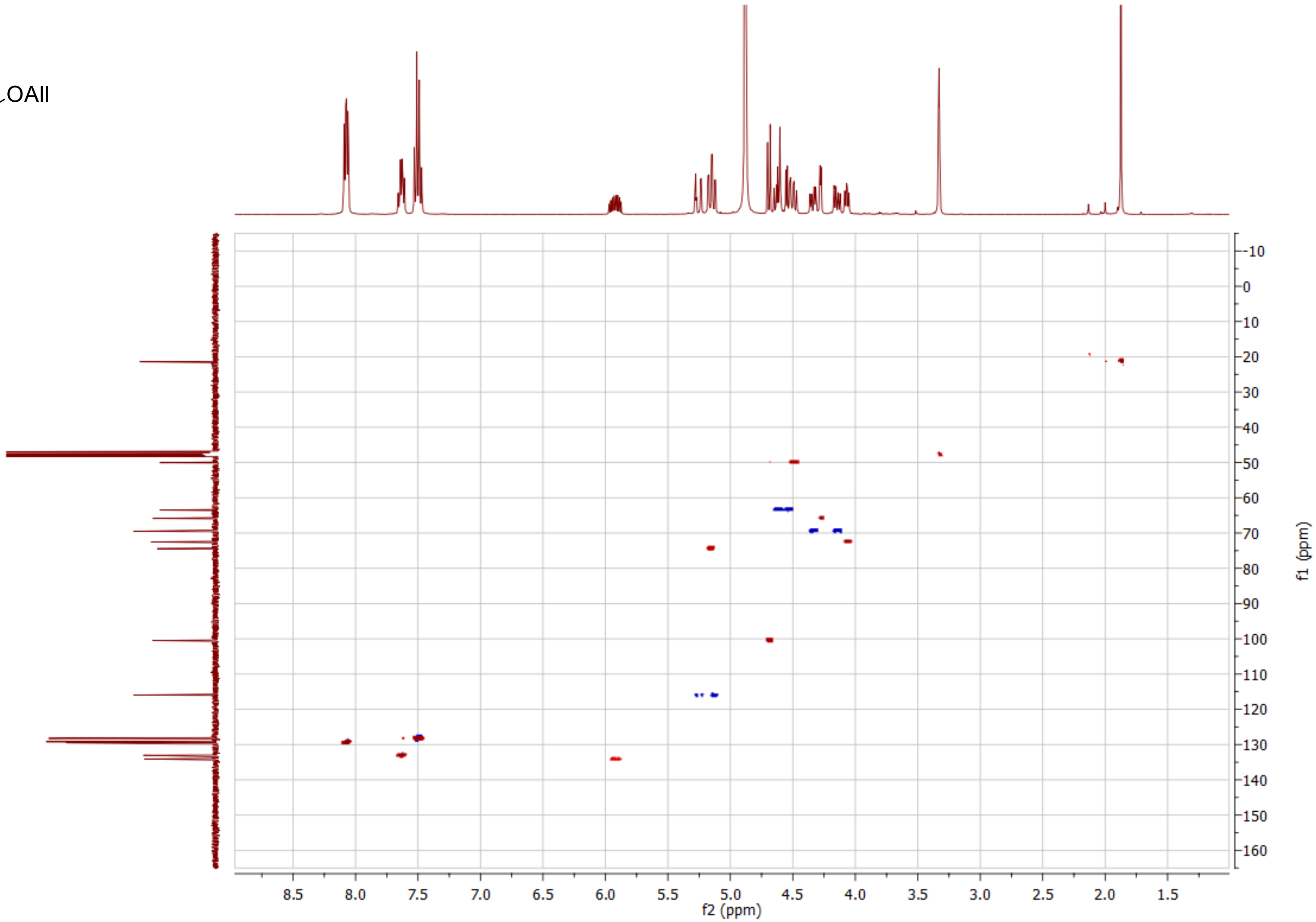

HMBC (400 × 101 MHz MeOD): Allyl 3,6-di-*O*-benzoyl-2-acetamido-2-deoxy-β-D-galactopyranoside 16

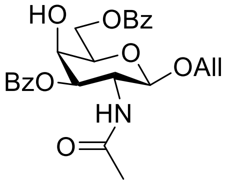

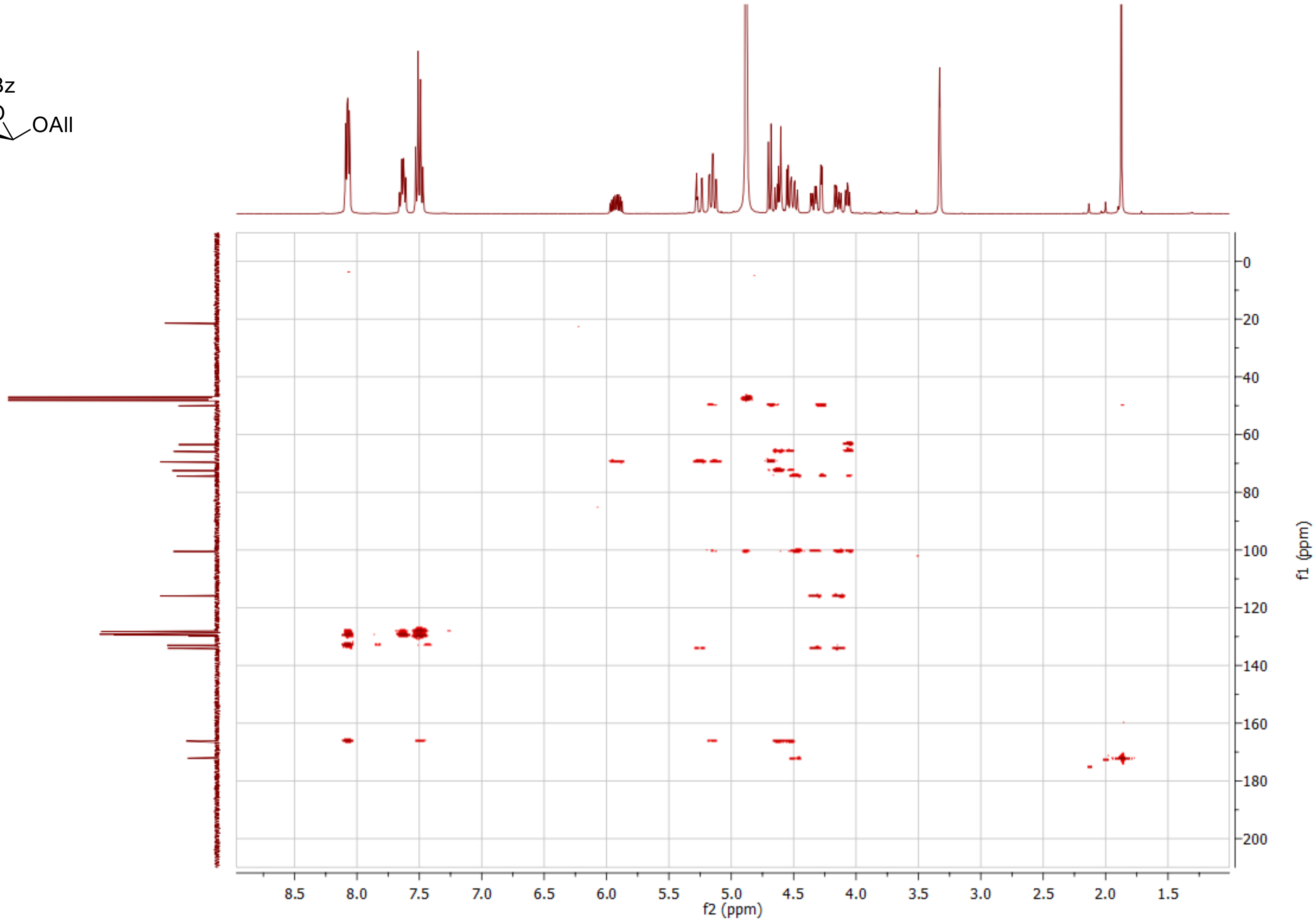

$^{13}C\{1H\}$ NMR (101 MHz, $CDCl_3$): Allyl 3,6-di-*O*-benzoyl-2-acetamido-2-deoxy-β-D-galactopyranoside 16

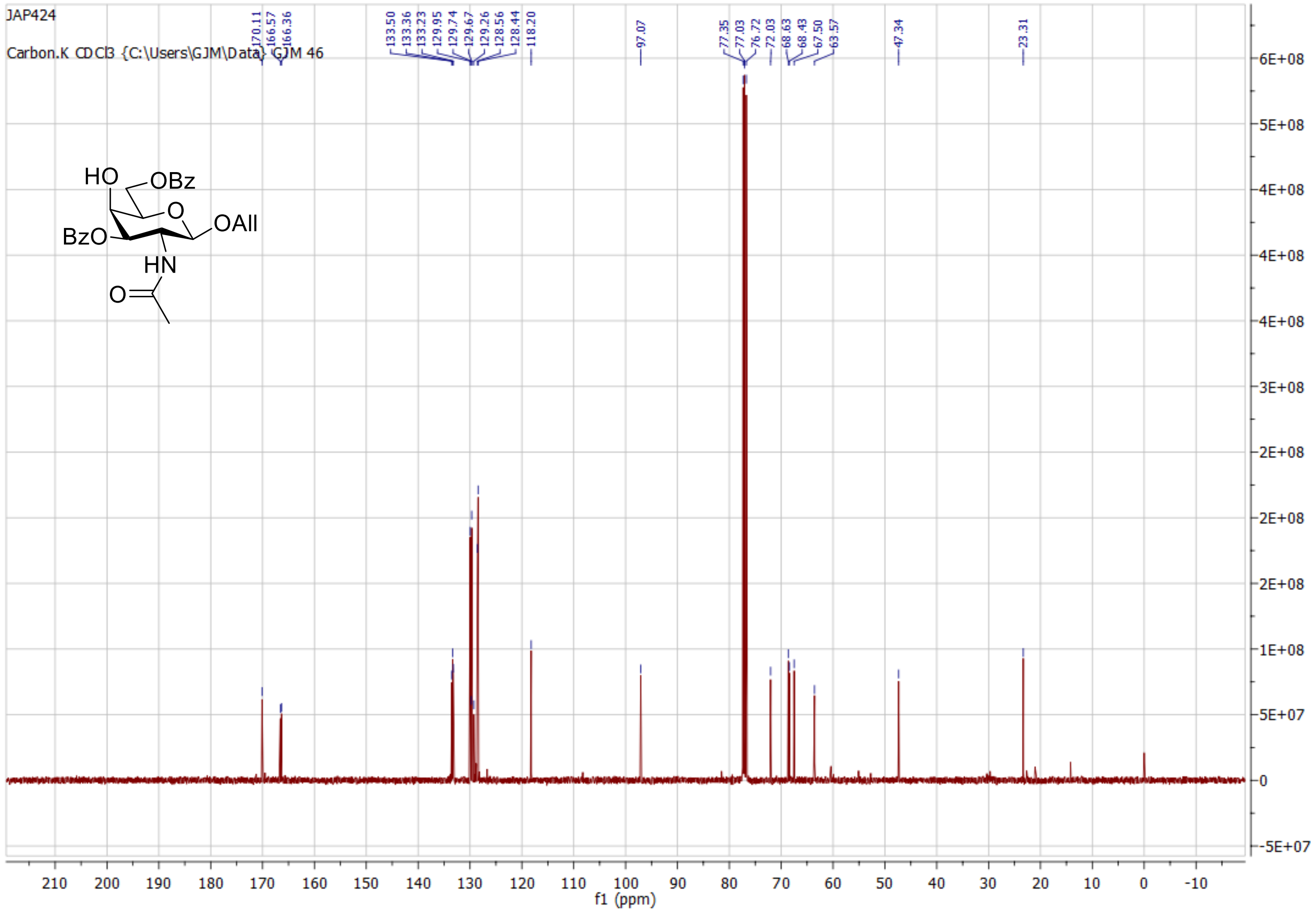

Compound 18

^{1}H NMR (400 MHz, CDCl₃): Allyl 3,6-di-*O*-benzoyl-2-acetamido-2-deoxy-α-D-galactopyranoside 18

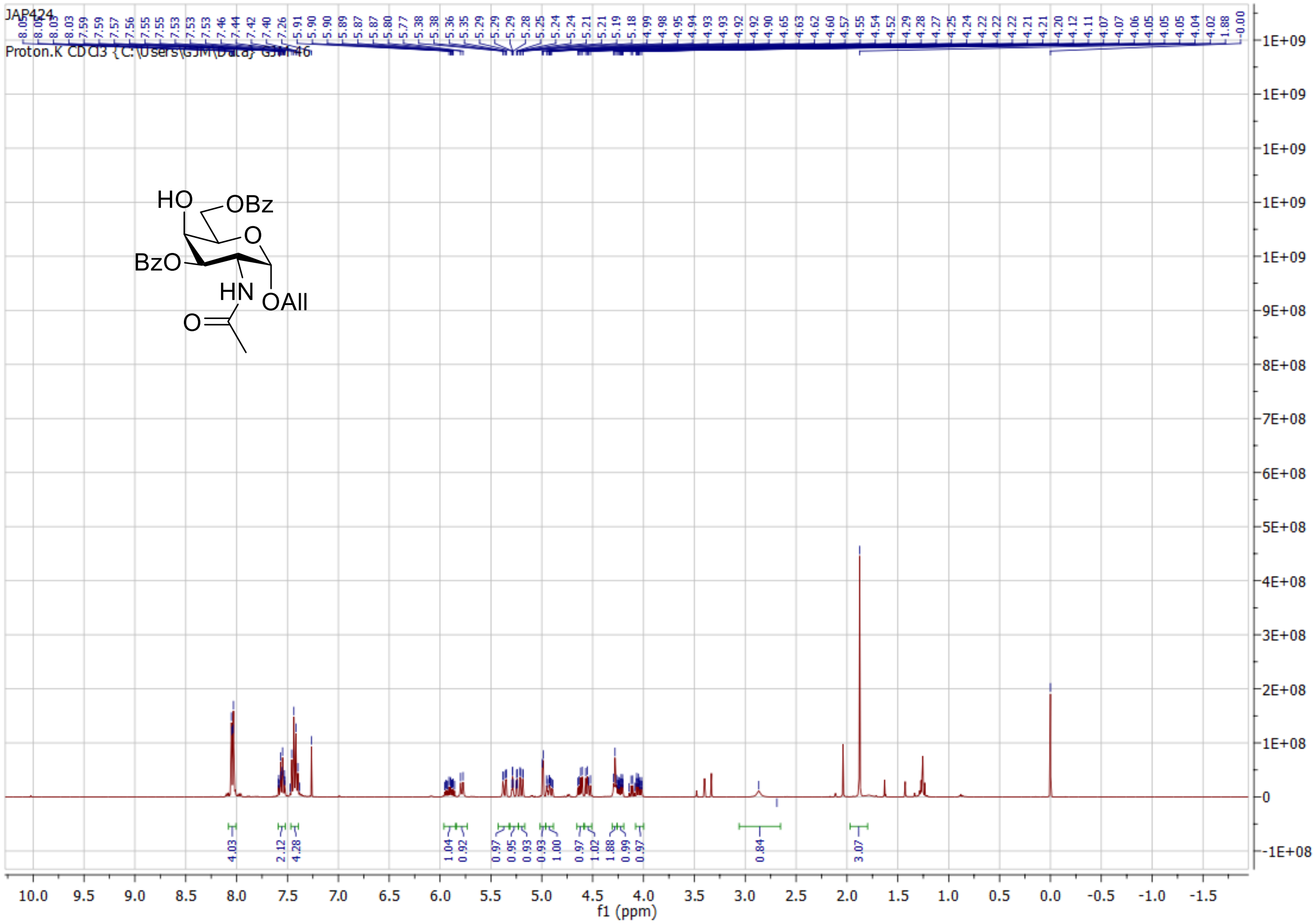

COSY (400 × 400 MHz, $CDCl_3$): Allyl 3,6-di-*O*-benzoyl-2-acetamido-2-deoxy-α-D-galactopyranoside 18

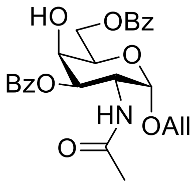

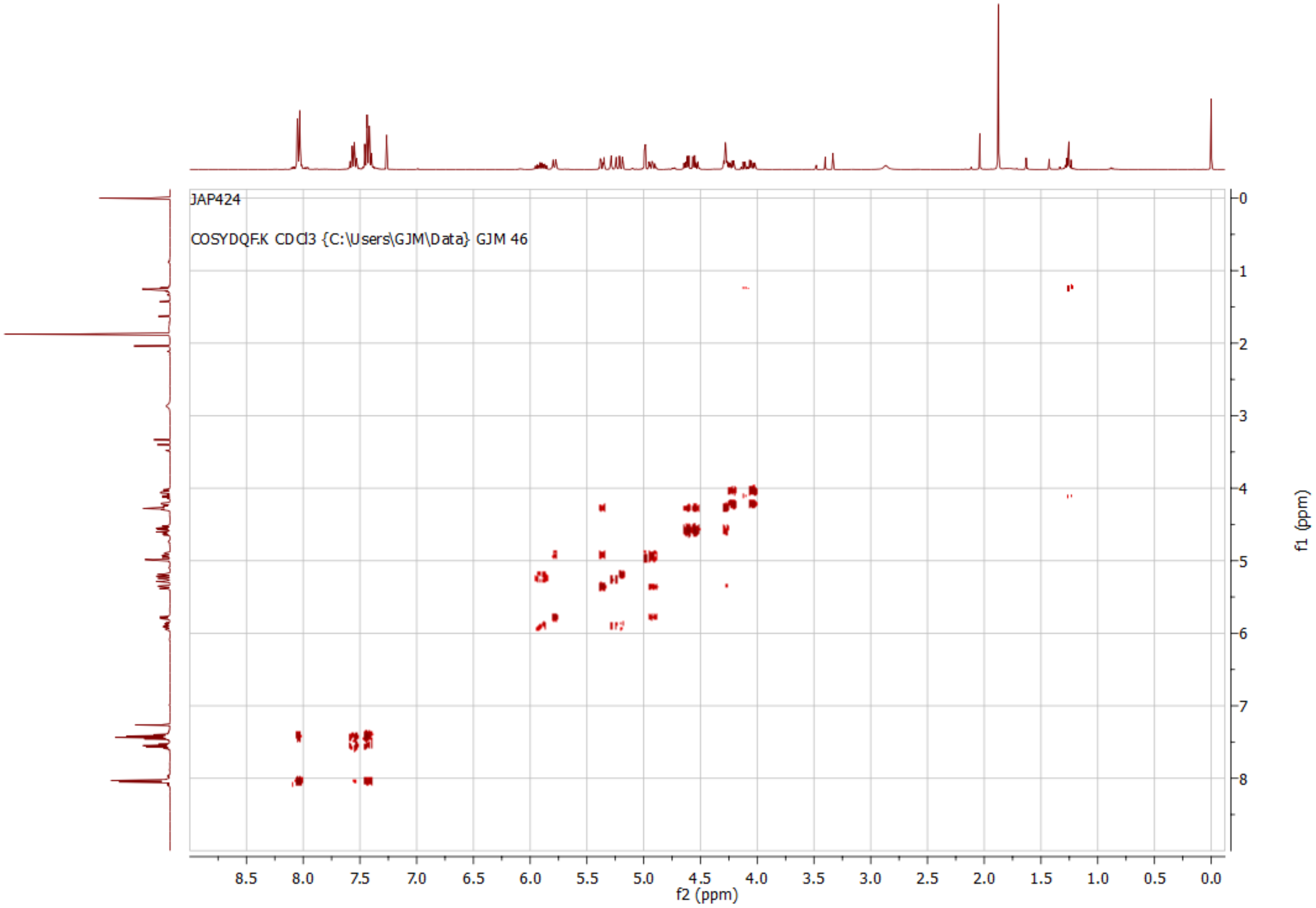

HSQC (400 × 101 MHz, CDCl$_3$): Allyl 3,6-di-*O*-benzoyl-2-acetamido-2-deoxy-α-D-galactopyranoside 18

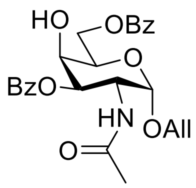

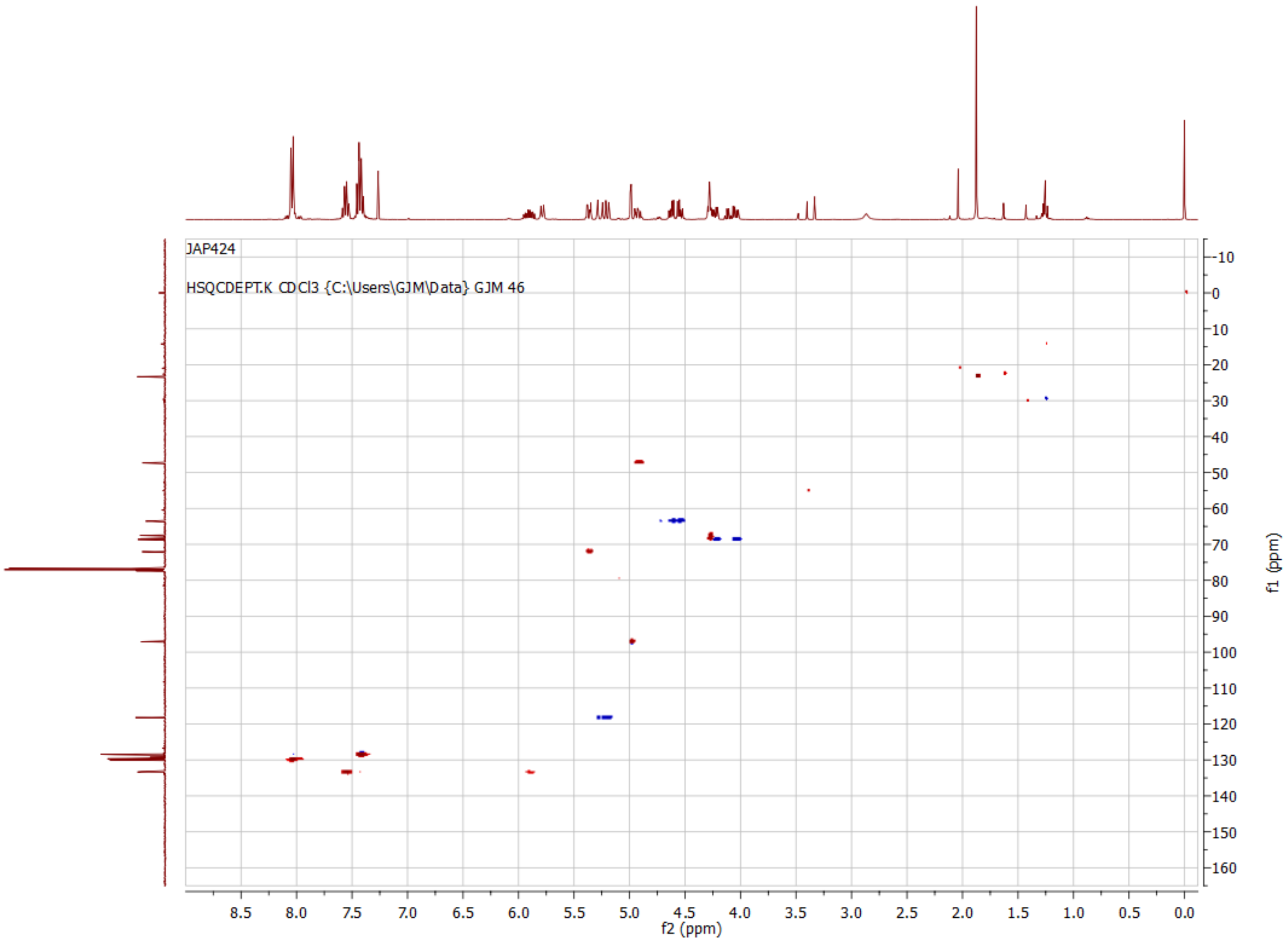

HMBC (400 × 101 MHz, CDCl₃): Allyl 3,6-di-*O*-benzoyl-2-acetamido-2-deoxy-α-D-galactopyranoside 18

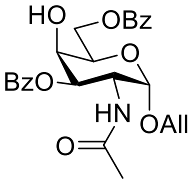

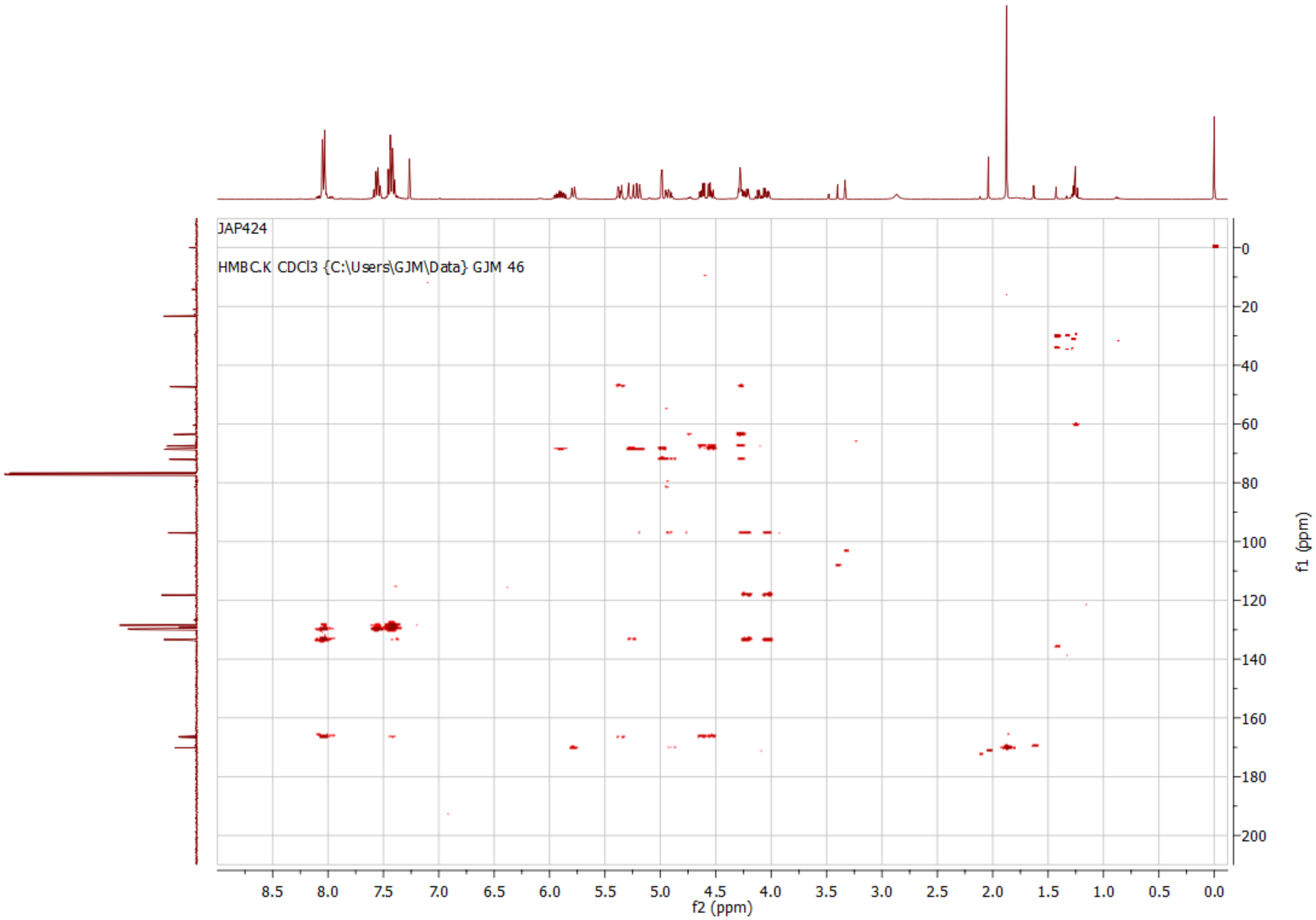

^{13}C{1H} NMR (101 MHz, CDCl$_3$): Allyl 3,6-di-*O*-benzoyl-2-acetamido-2-deoxy-α-D-galactopyranoside 18

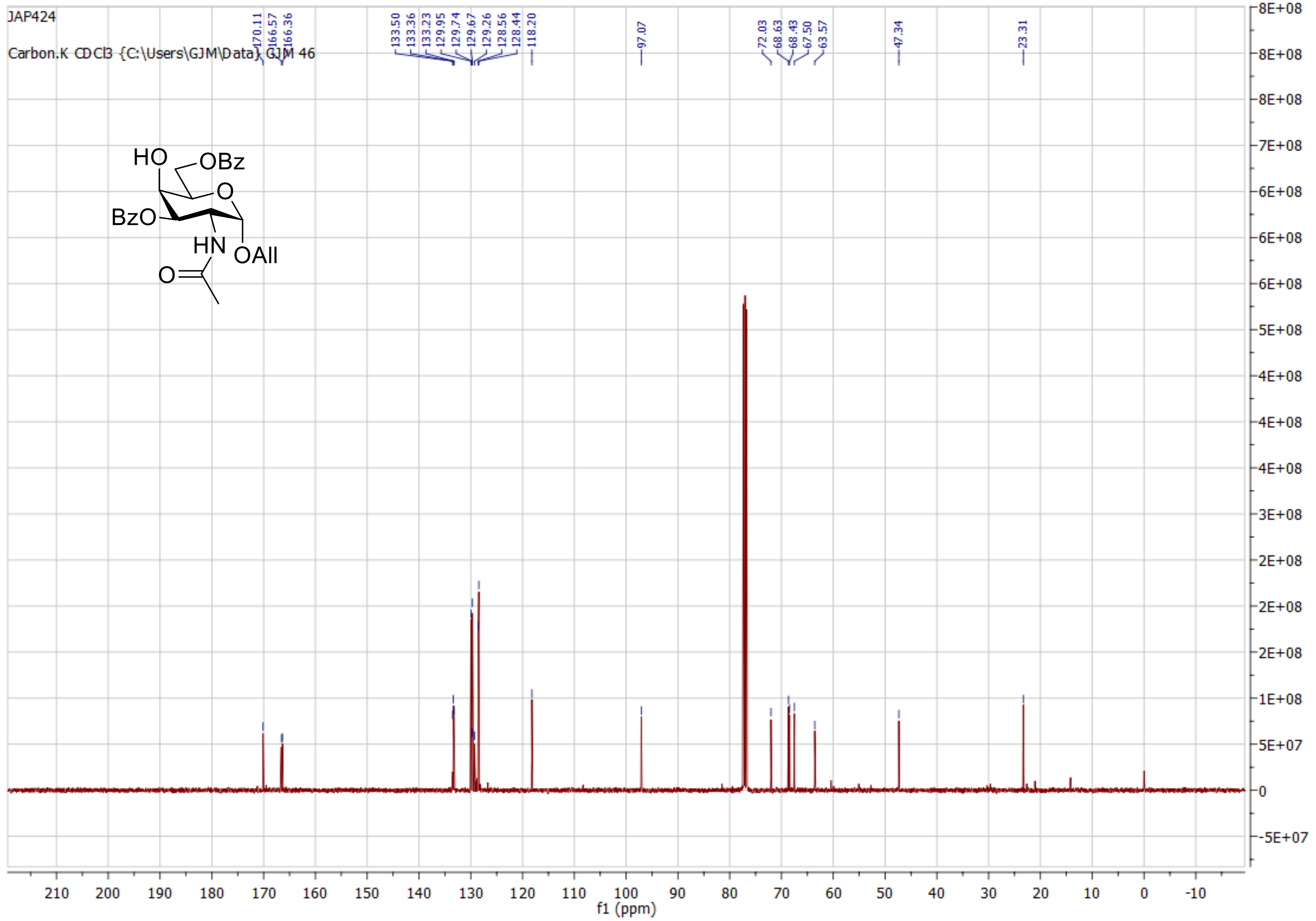

Compound 20

^{1}H NMR (400 MHz CDCl₃): 3-Azidopropyl (2-acetamido-3,6-di-*O*-benzoyl-2-deoxy)-β-D-galactopyranoside 20

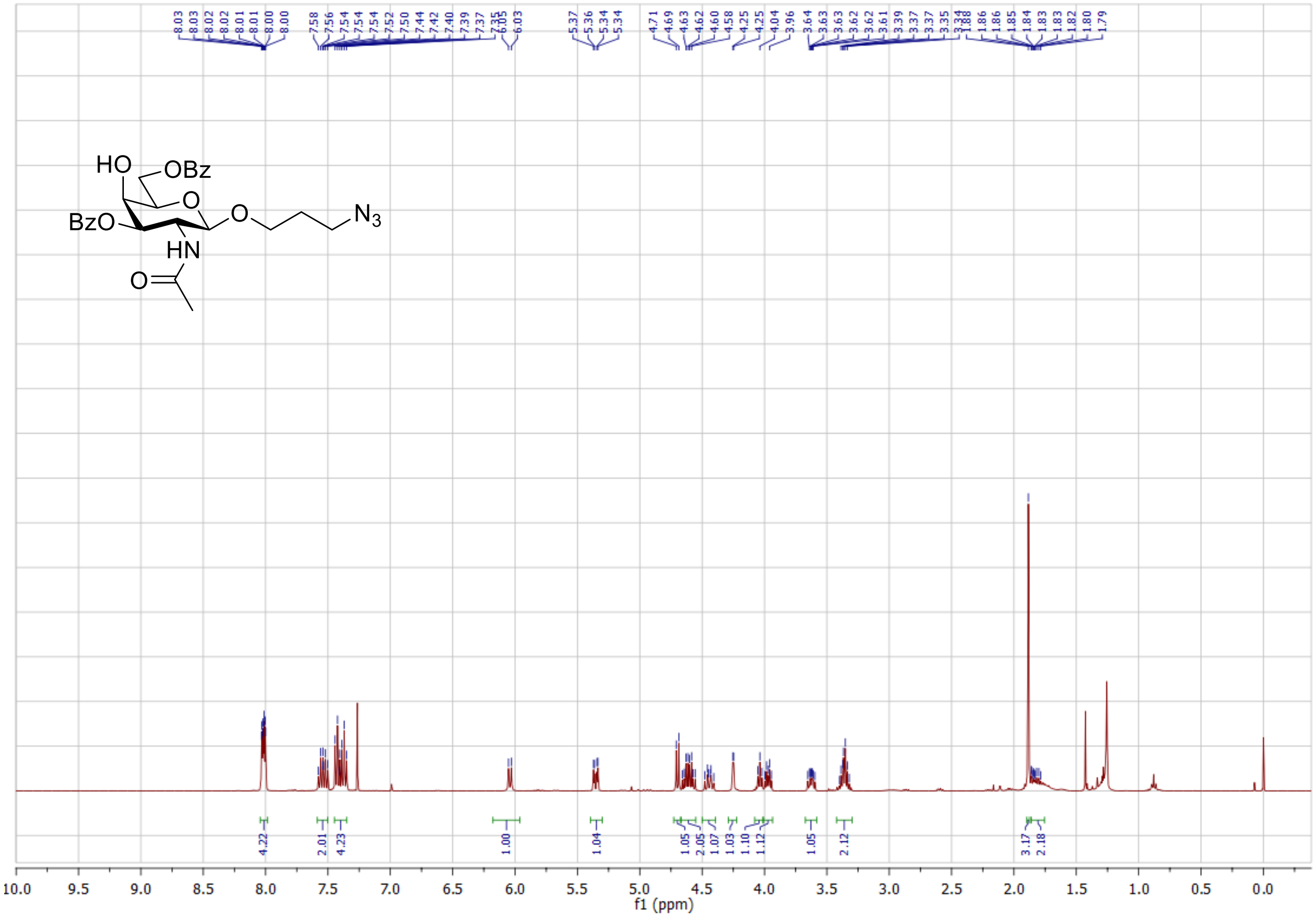

COSY (400 × 400 MHz, $CDCl_3$): 3-Azidopropyl (2-acetamido-3,6-di-*O*-benzoyl-2-deoxy)-β-D-galactopyranoside 20

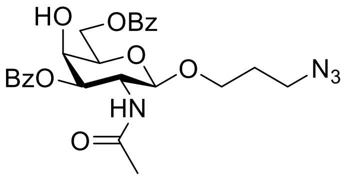

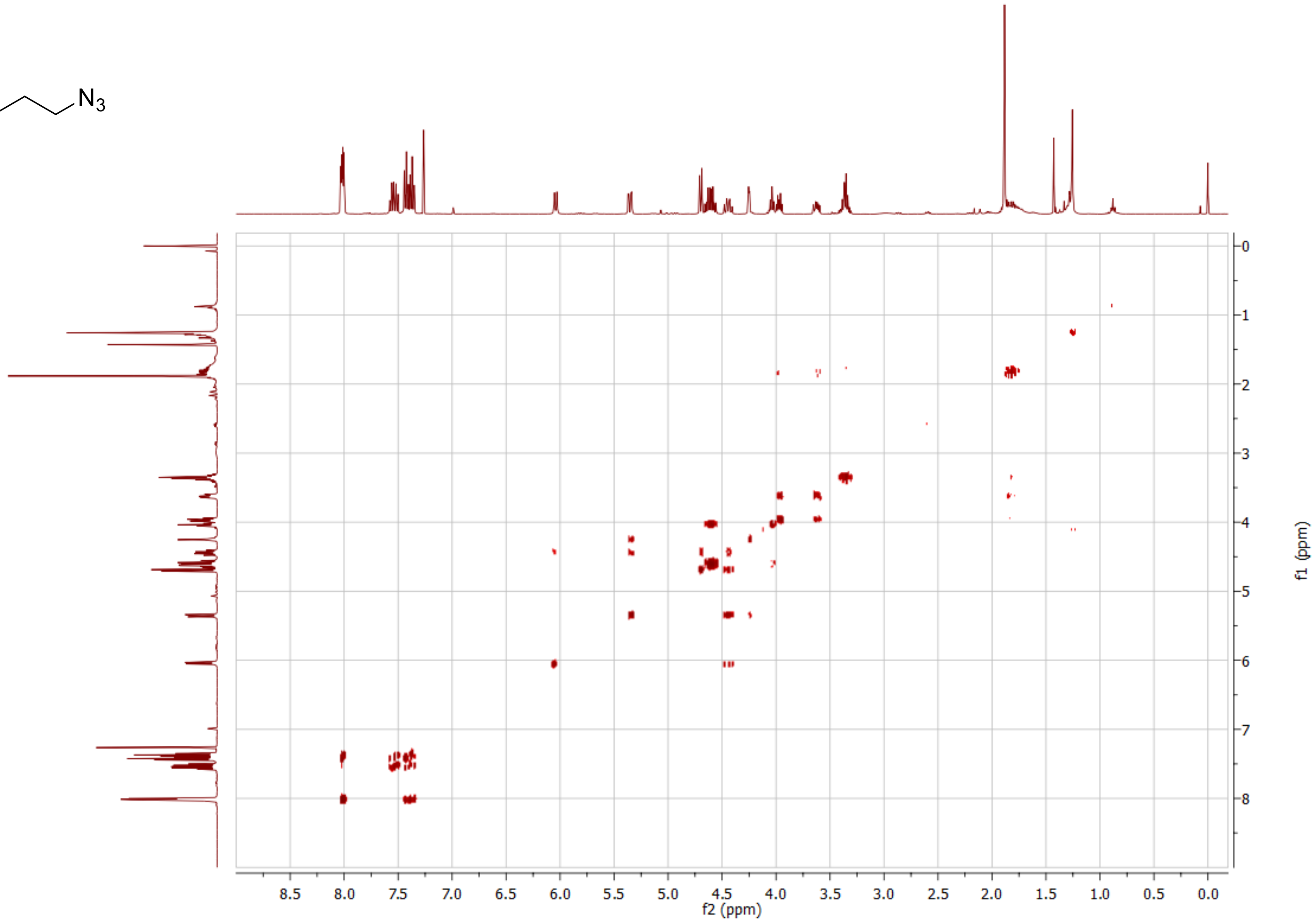

HSQC (400 × 101 MHz, $CDCl_3$): 3-Azidopropyl (2-acetamido-3,6-di-*O*-benzoyl-2-deoxy)-β-D-galactopyranoside 20

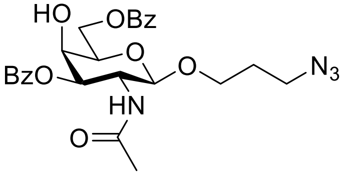

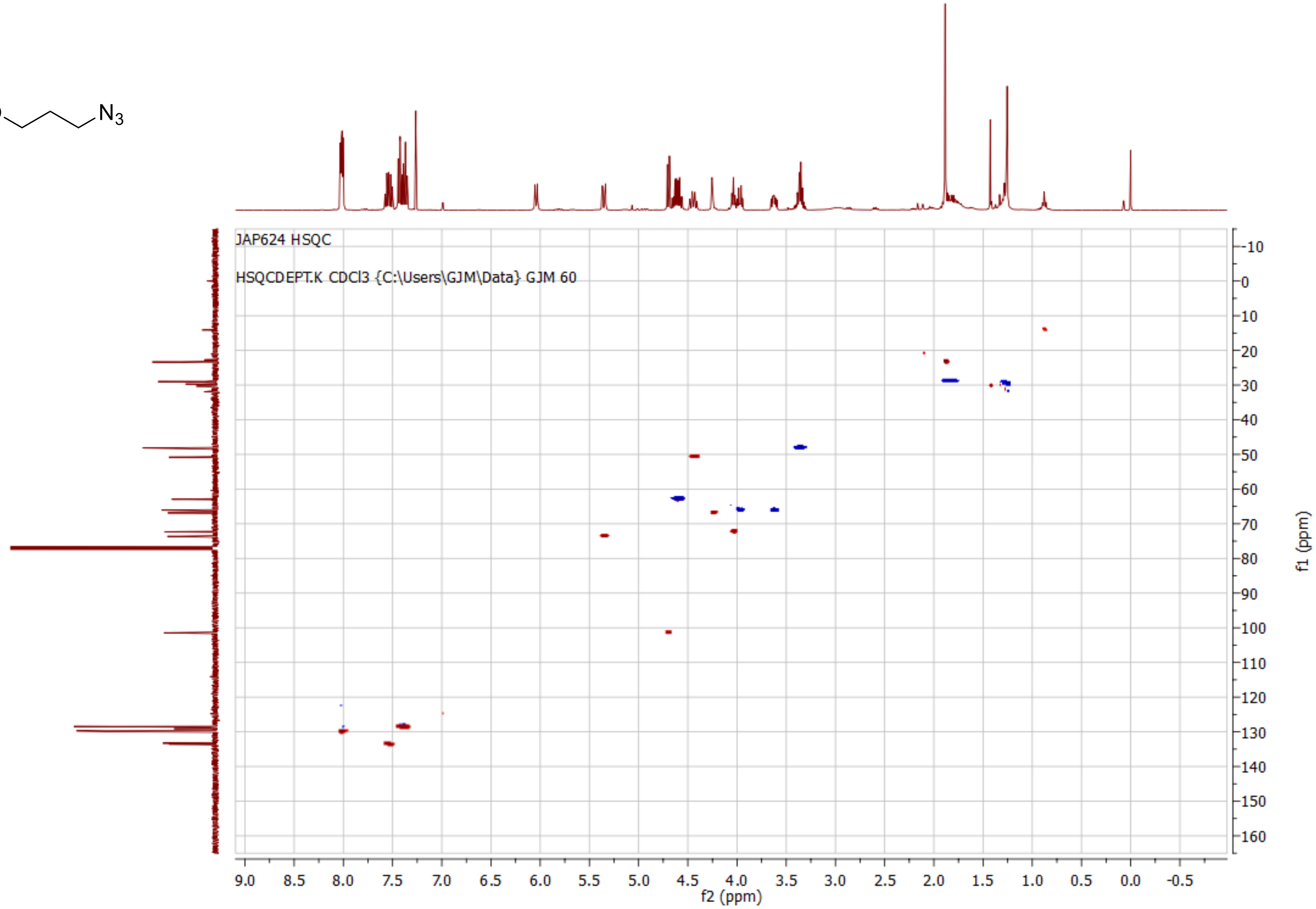

HMBC (400 × 101 MHz, $CDCl_3$): 3-Azidopropyl (2-acetamido-3,6-di-*O*-benzoyl-2-deoxy)-β-D-galactopyranoside 20

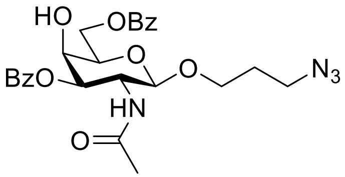

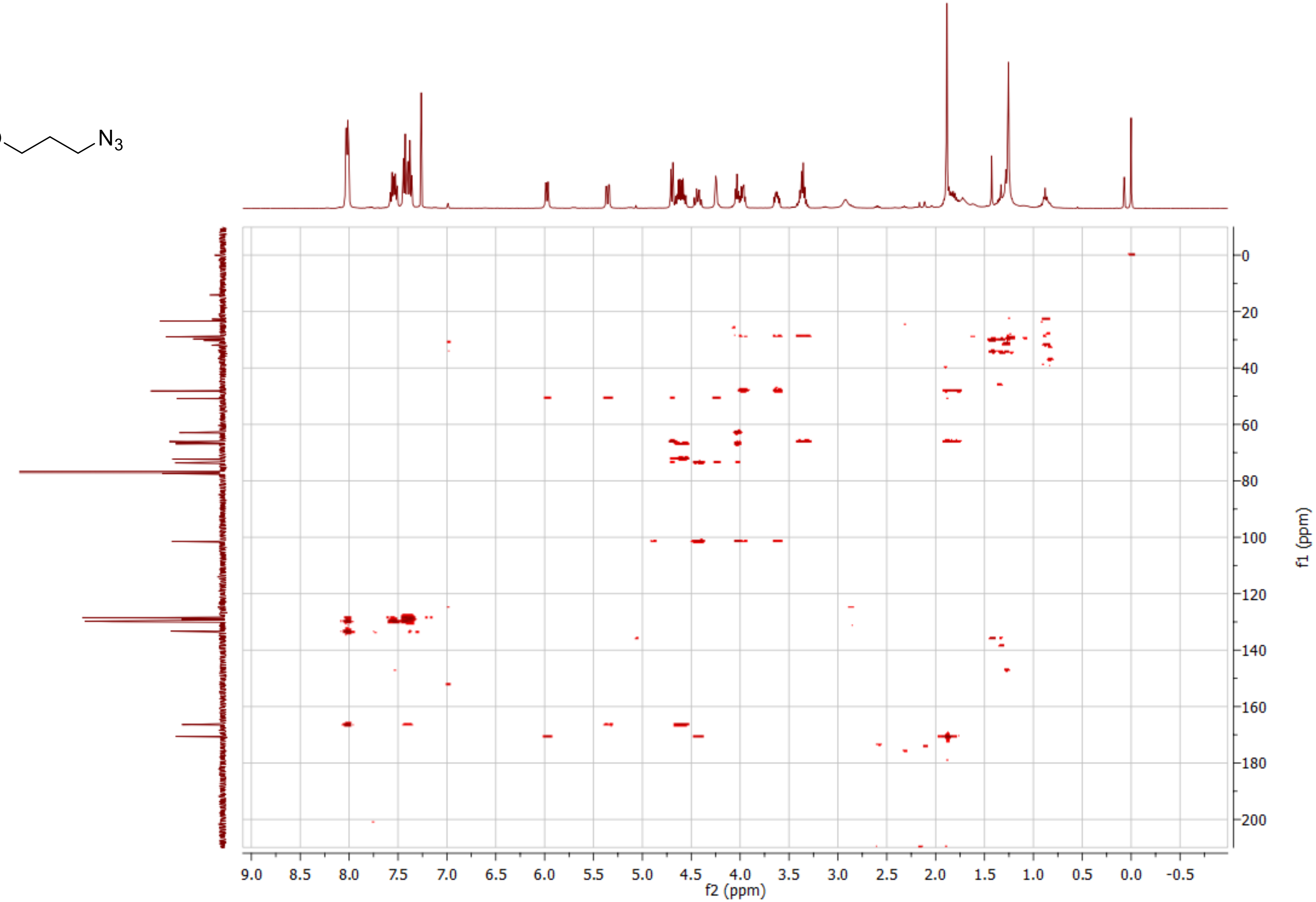

$^{13}C\{1H\}$ NMR (101 MHz $CDCl_3$): 3-Azidopropyl (2-acetamido-3,6-di-*O*-benzoyl-2-deoxy)-β-D-galactopyranoside 20

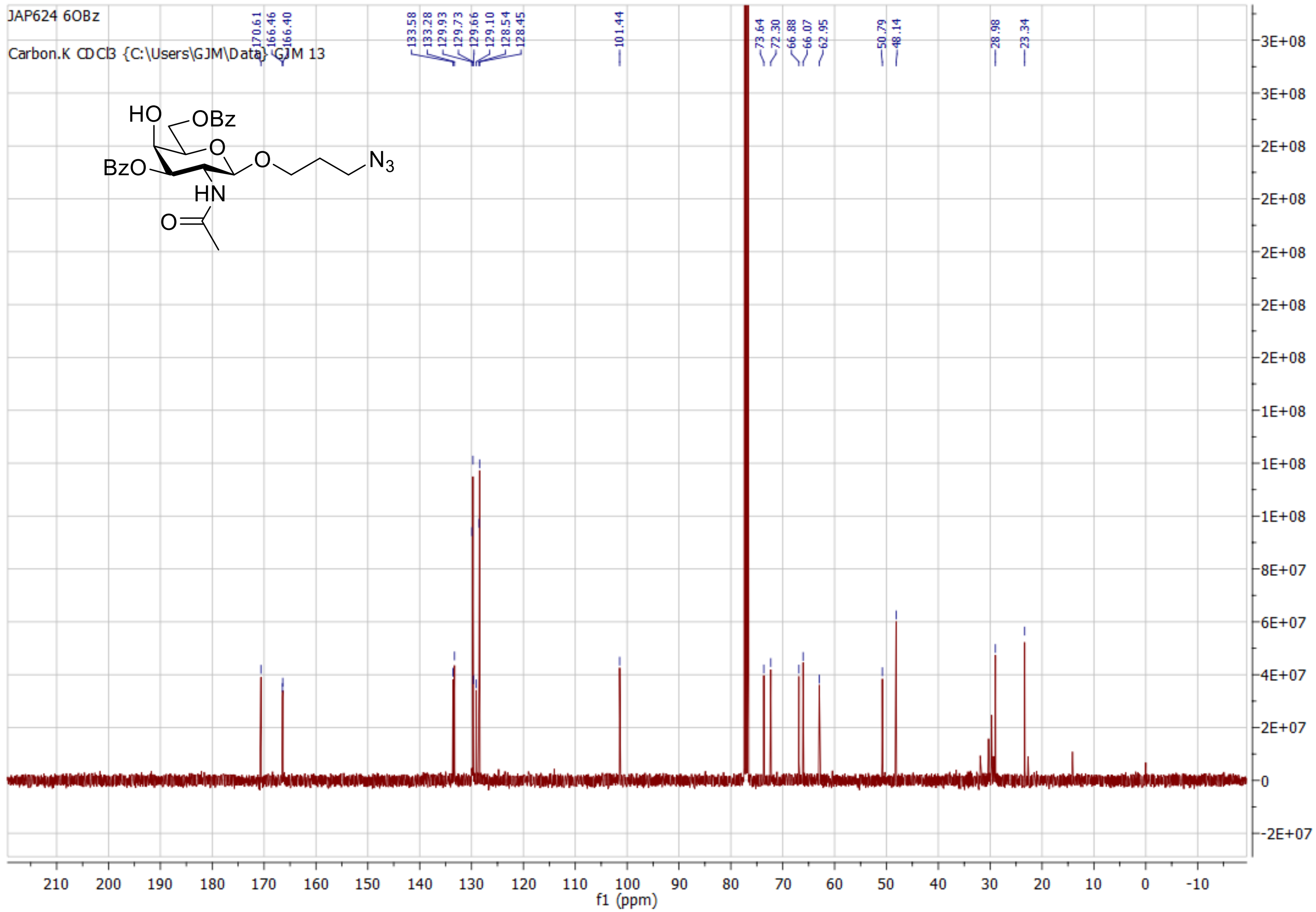

Compound 22

^{1}H NMR (400 MHz CDCl₃): Methyl 2,3,6-tri-*O*-acetyl-α-D-galactopyranoside 22

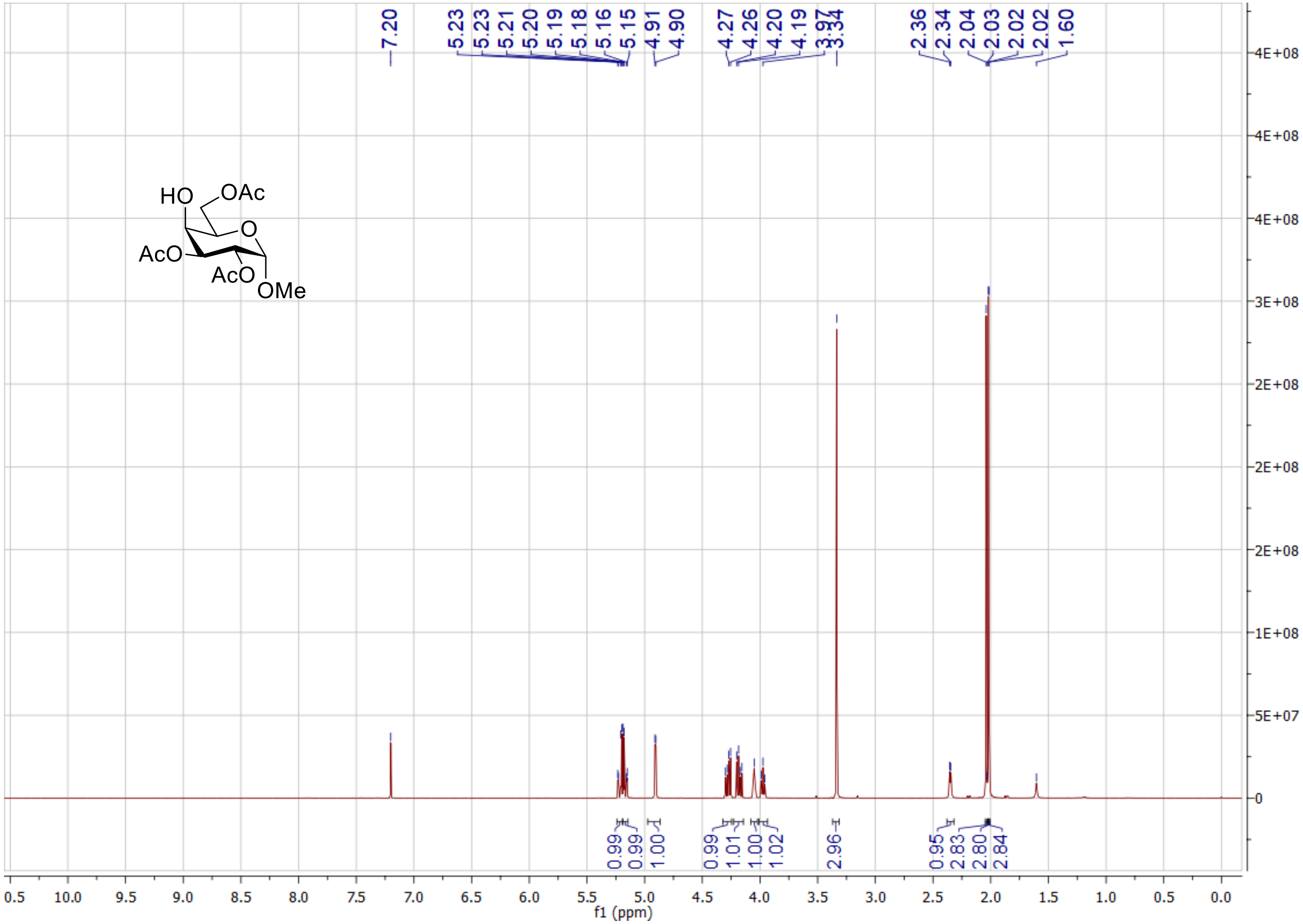

^{13}C{1H} NMR 101 MHz CDCl₃): Methyl 2,3,6-tri-*O*-acetyl-α-D-galactopyranoside 22

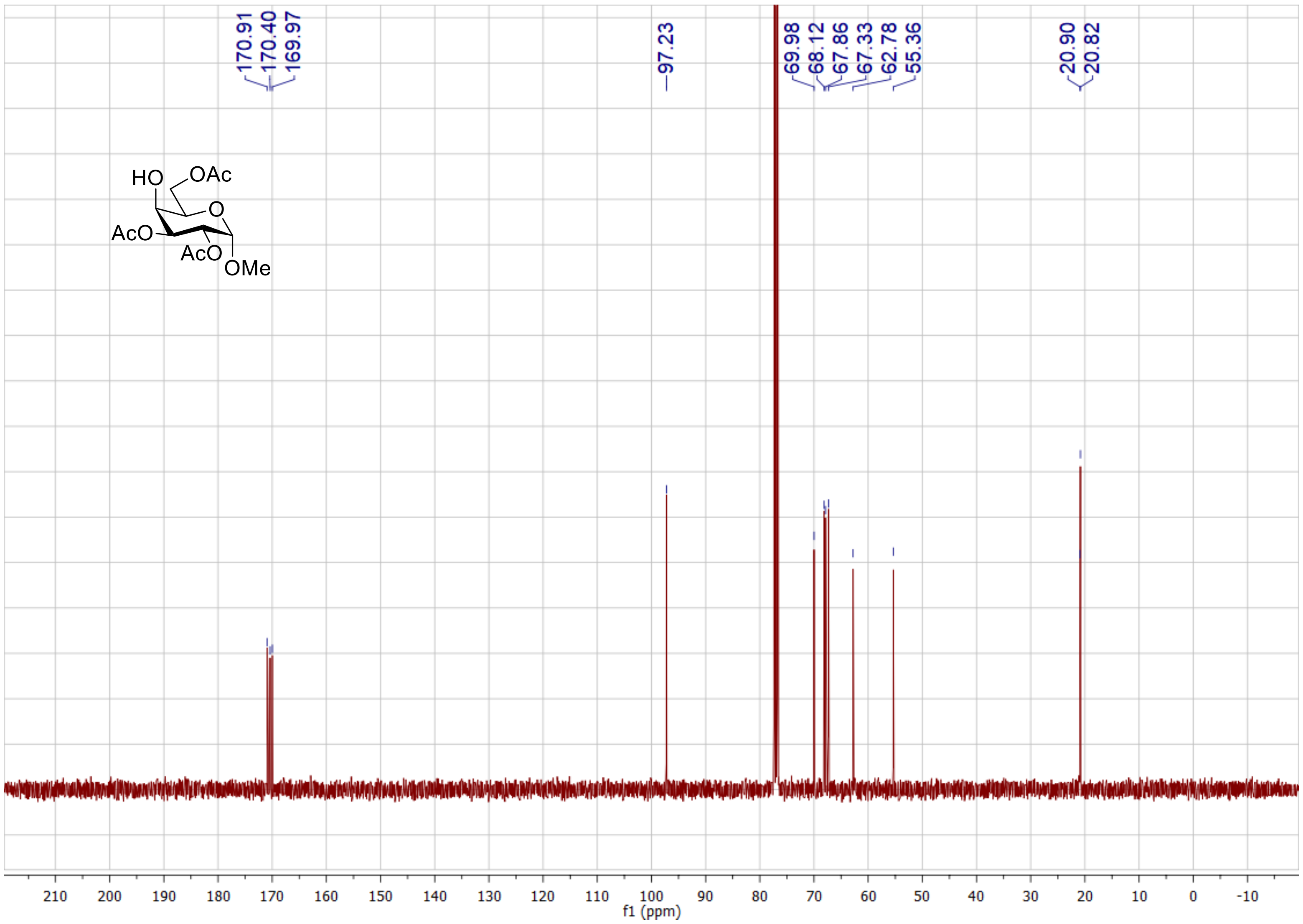

Compound 23

^{1}H NMR (400 MHz CDCl$_3$): Methyl 2,6-di-*O*-*tert*-butylcarbamoyl-3,4-*O*-carbonyl-α-D-galactopyranoside 23

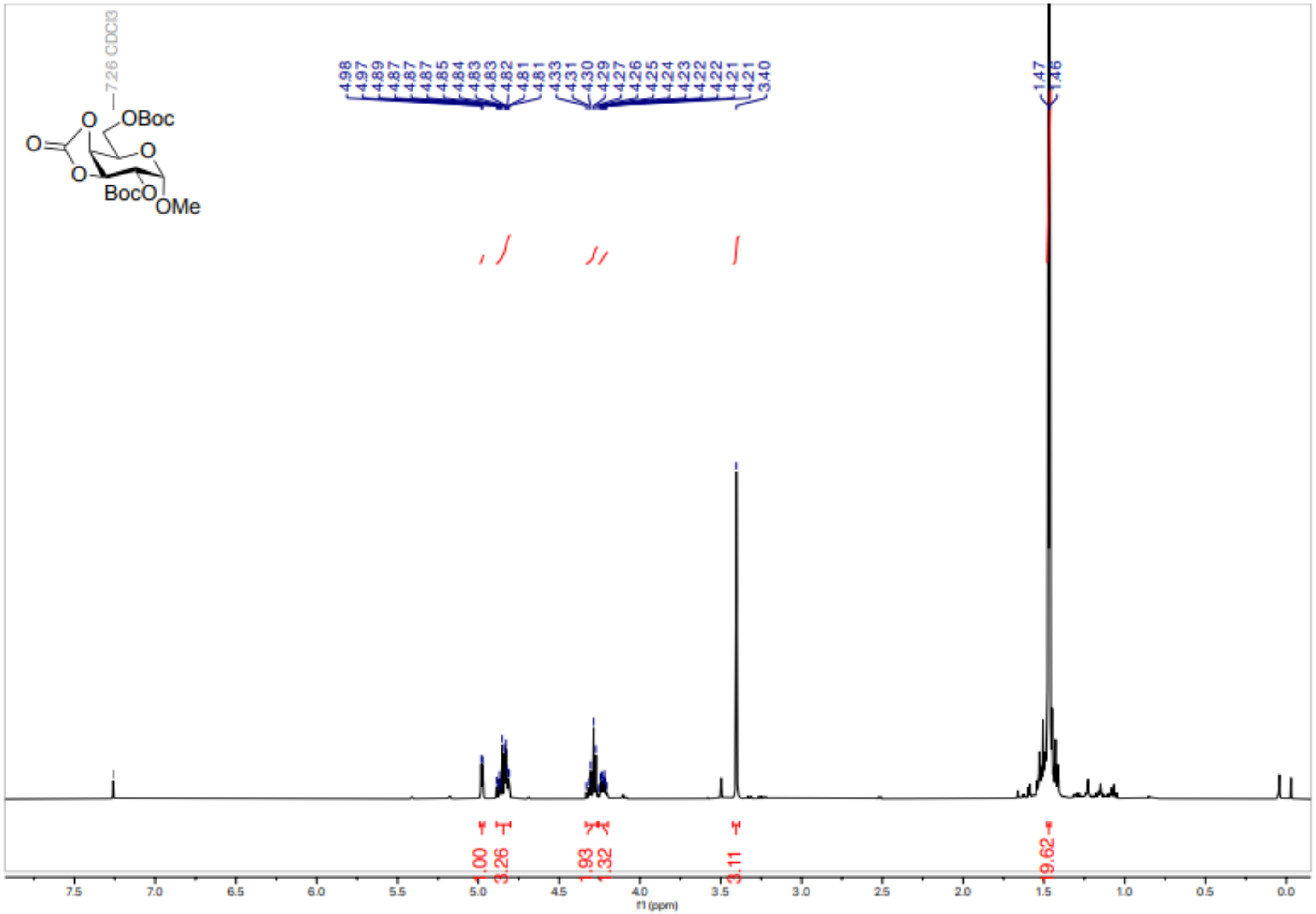

HMBC (400 × 101 MHz CDCl₃): Methyl 2,6-di-*O*-*tert*-butylcarbamoyl-3,4-*O*-carbonyl-α-D-galactopyranoside 23

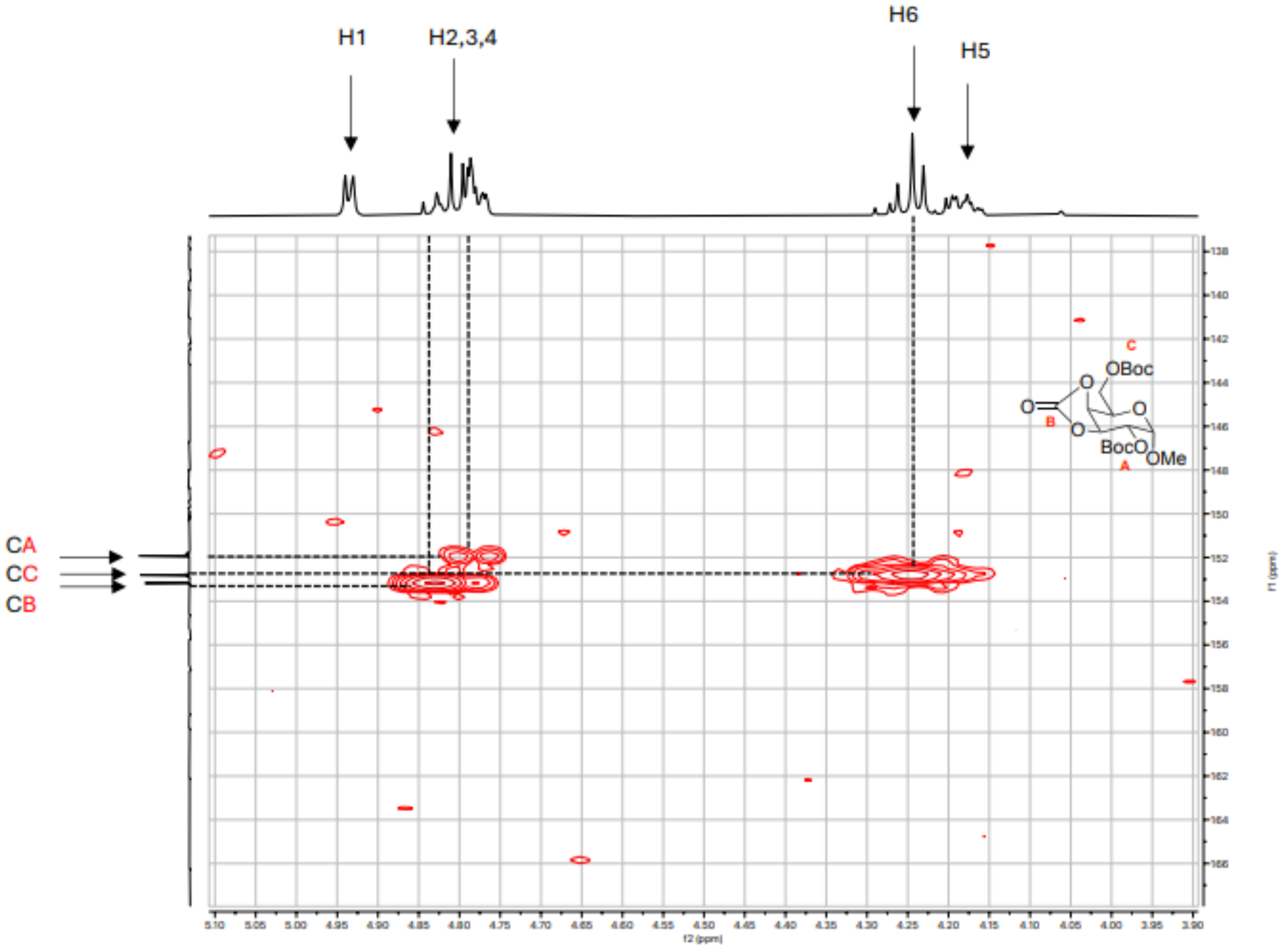

$^{13}C\{1H\}$ NMR (101 MHz $CDCl_3$): Methyl 2,6-di-*O*-*tert*-butylcarbamoyl-3,4-*O*-carbonyl-α-D-galactopyranoside 23

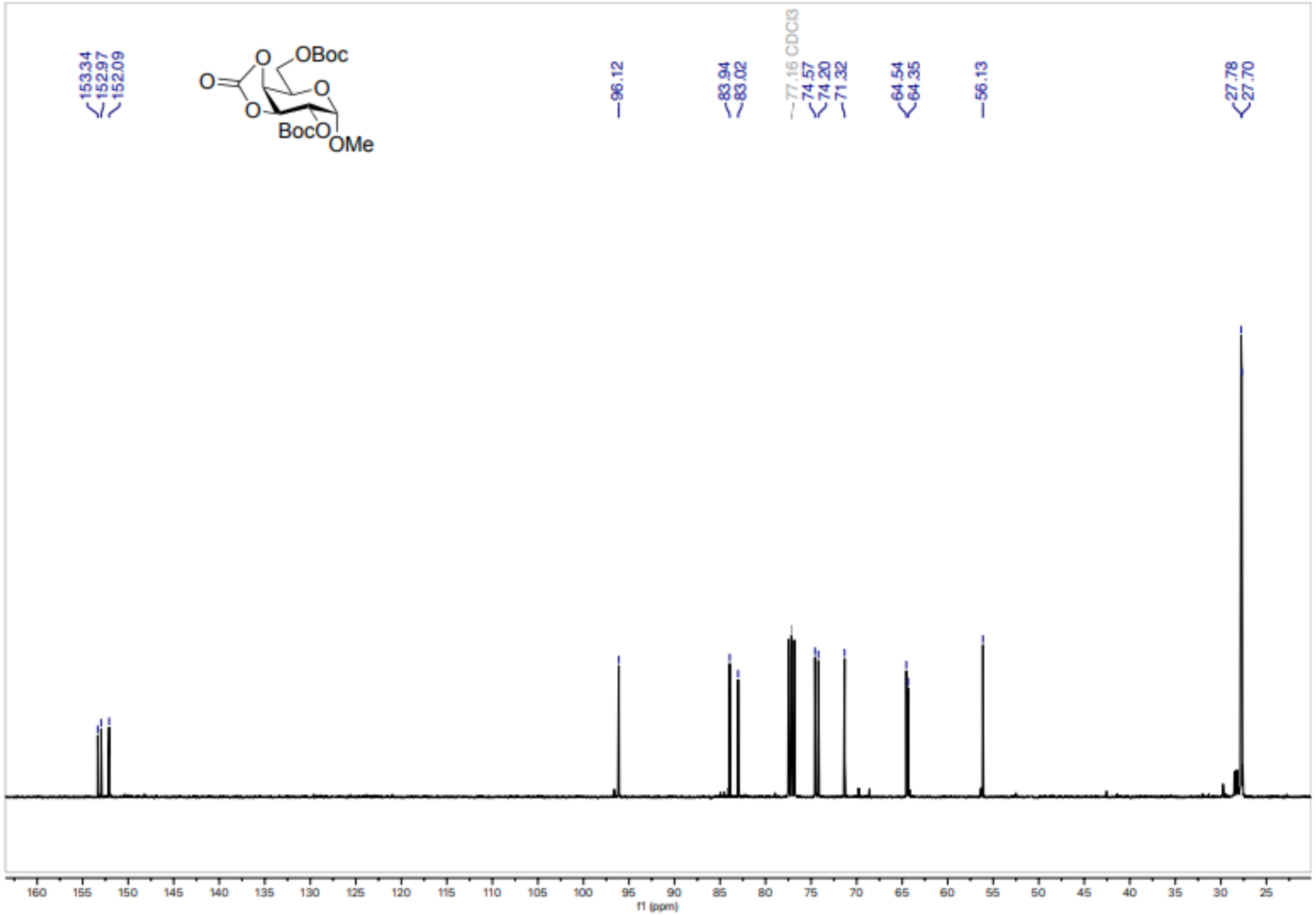

Compound 25

^{1}H NMR (400 MHz CDCl$_3$): Methyl 2,3-di-*O*-benzoyl-α-L-fucopyranoside 25

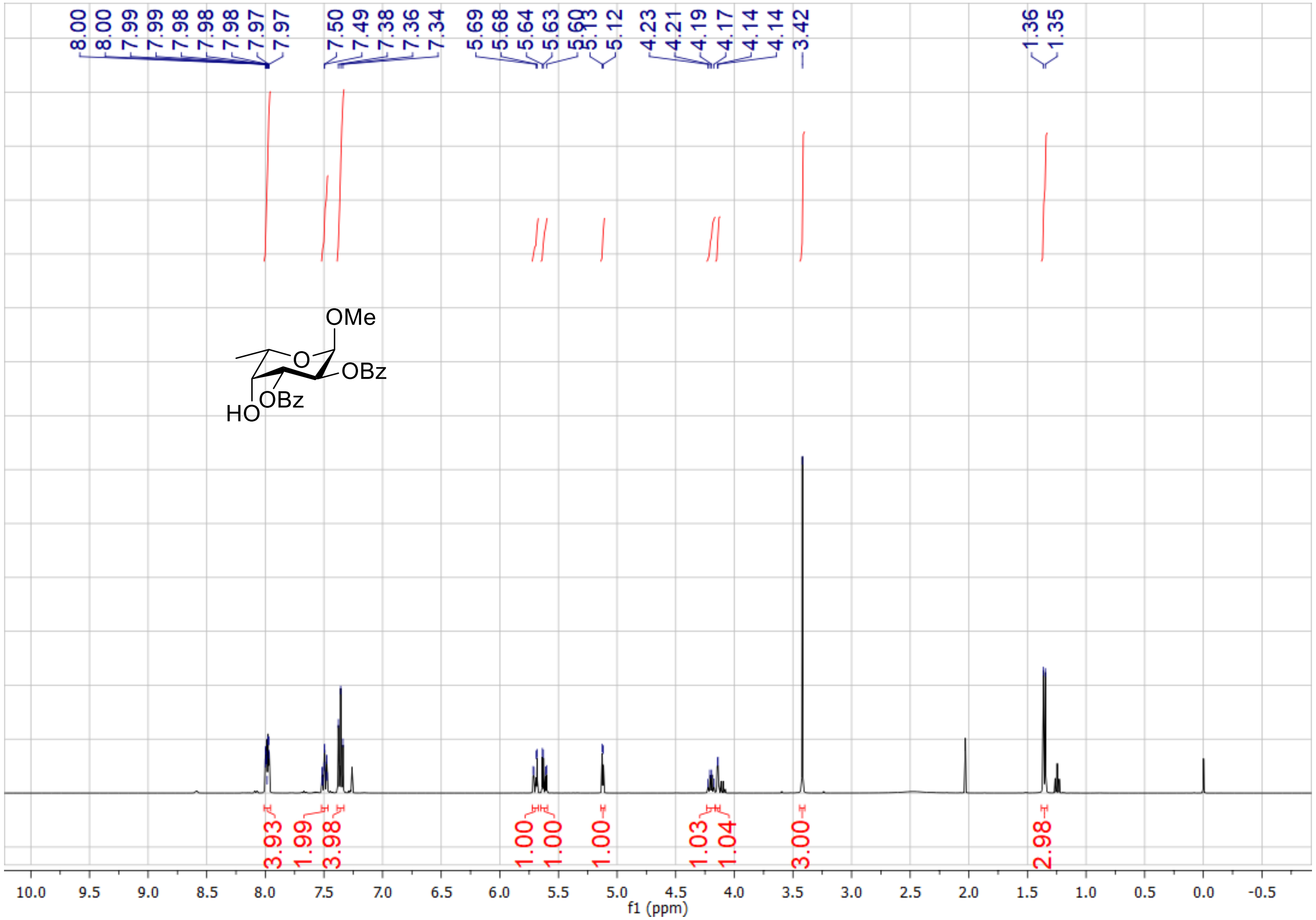

^{13}C{1H} NMR (101 MHz $CDCl_3$): Methyl 2,3-di-*O*-benzoyl-α-L-fucopyranoside 25

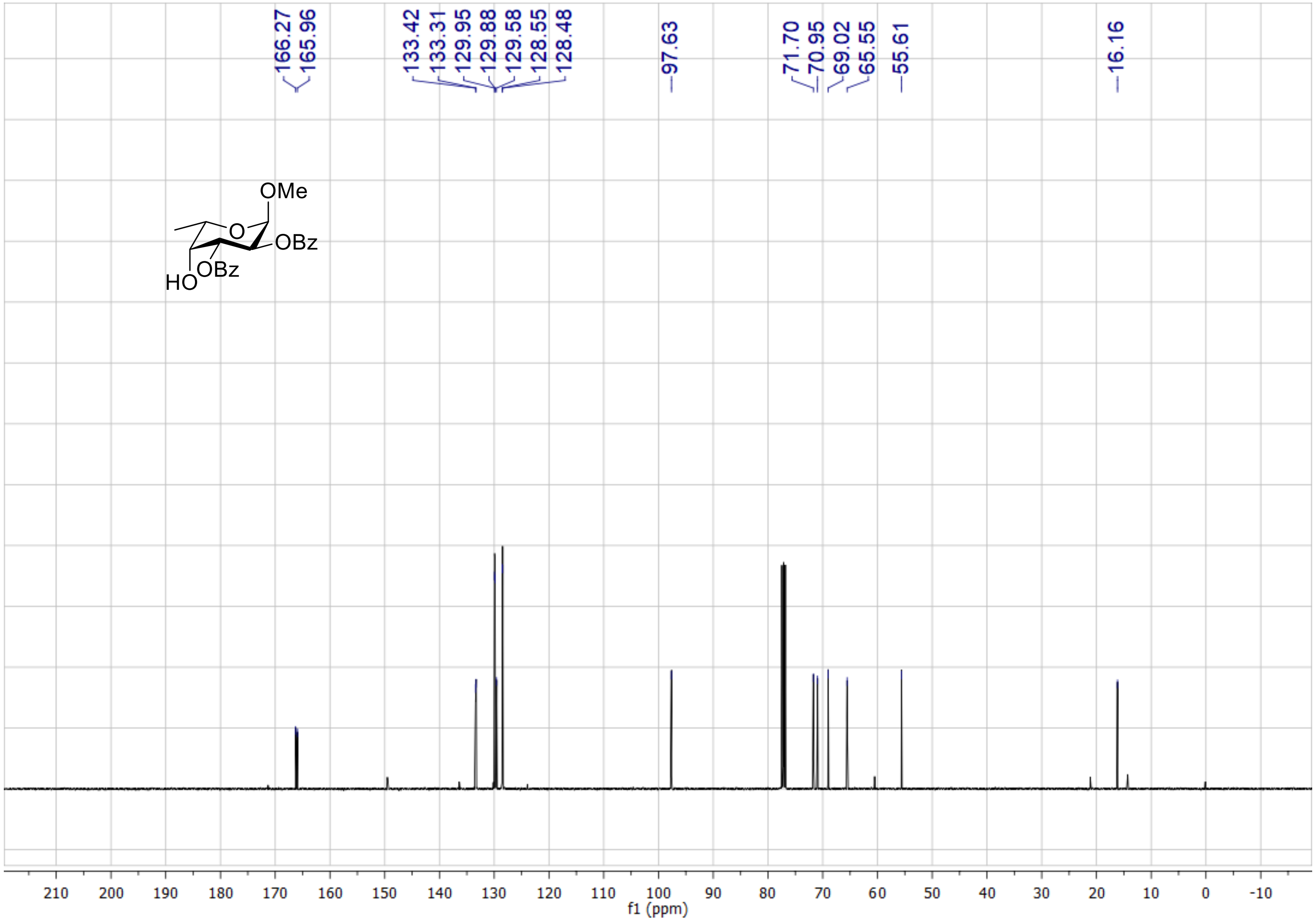

Compound 27

^{1}H NMR (400 MHz, CDCl$_3$) Phenyl 2,3,4,6-tetra-*O*-benzoyl-1-thio-β-D-galactopyranoside 27

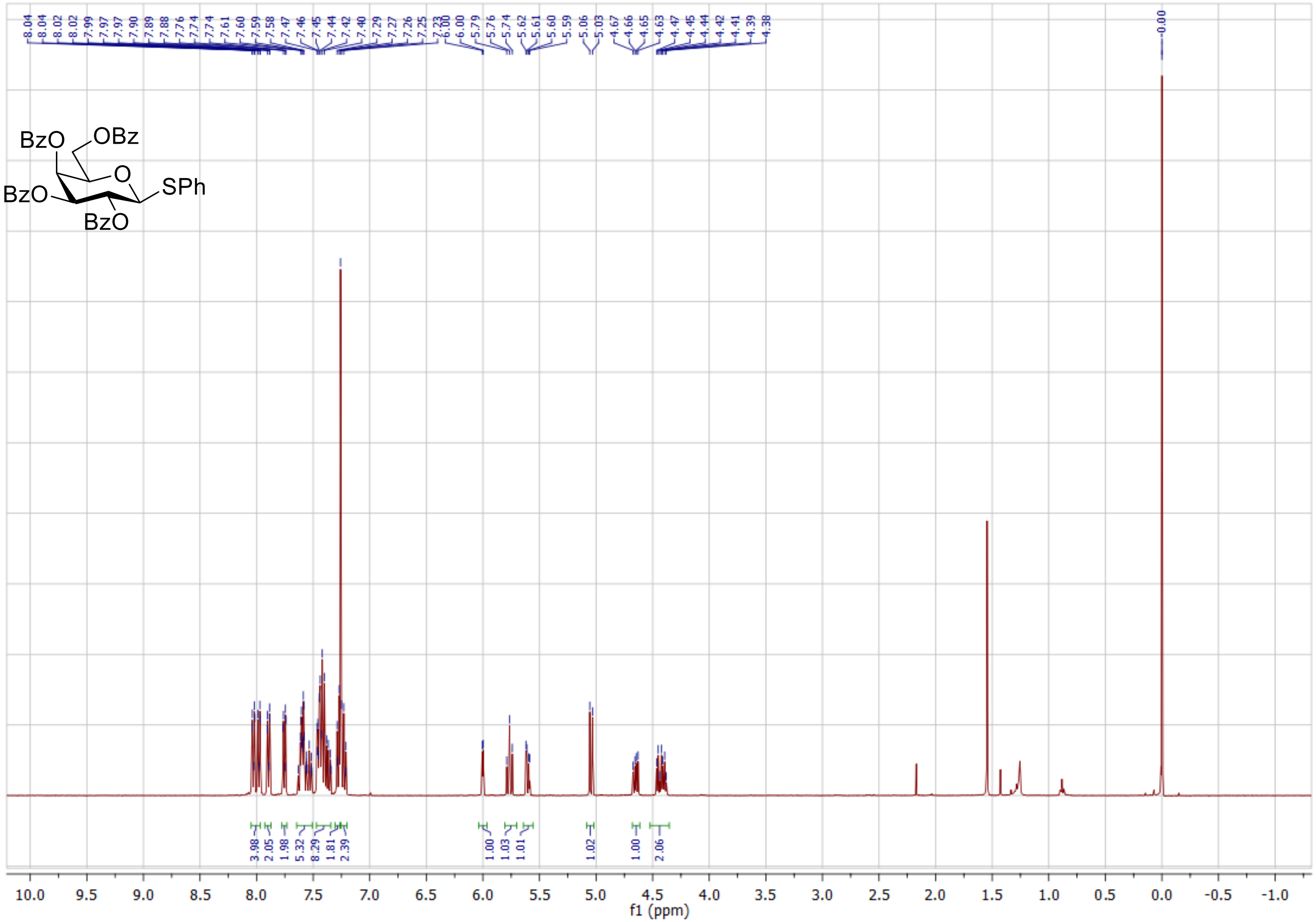

^{13}C{1H} NMR (101 MHz, CDCl₃) Phenyl 2,3,4,6-tetra-*O*-benzoyl-1-thio-β-D-galactopyranoside 27

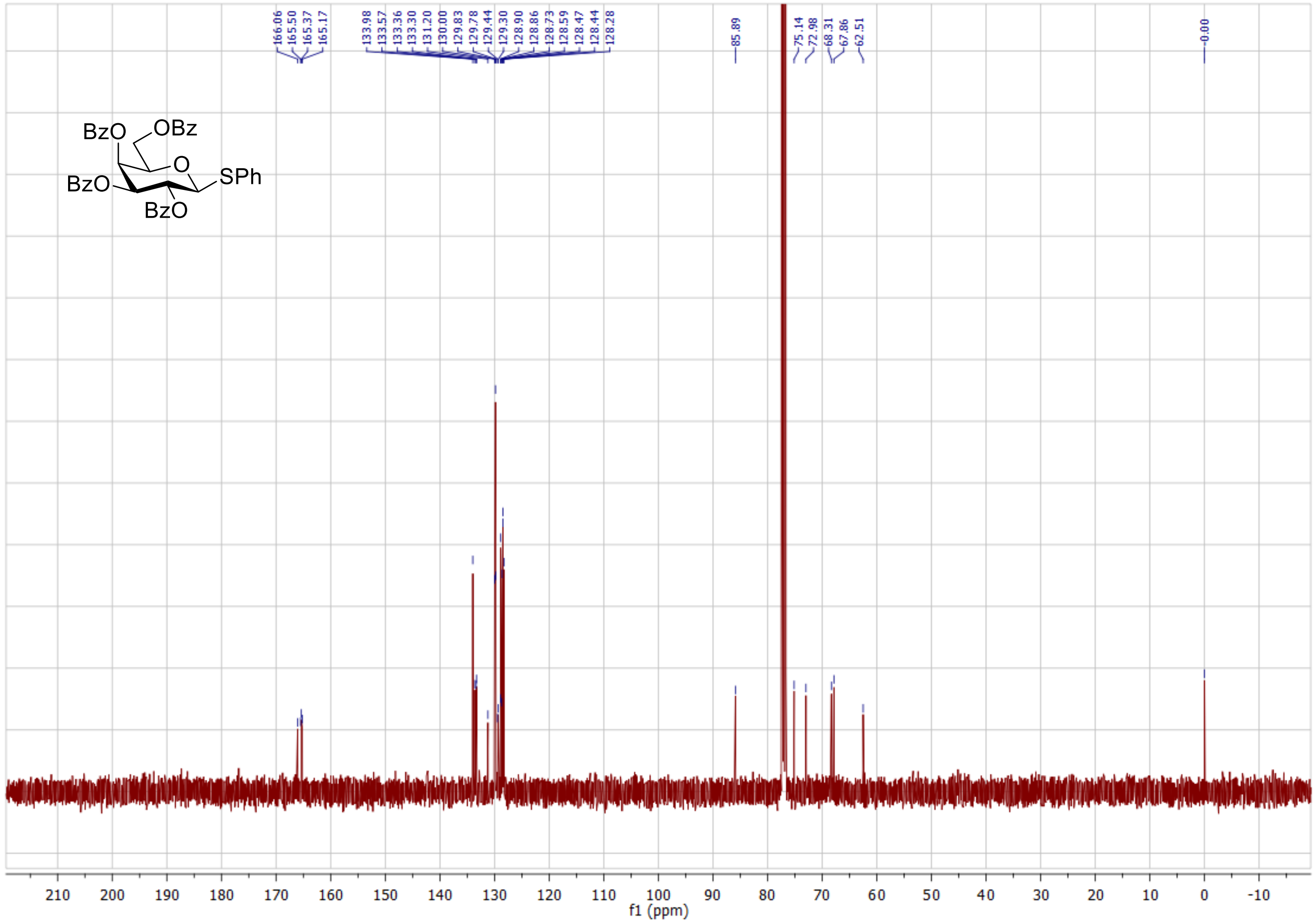

Compounds 28/29

^{1}H NMR (400 MHz, CDCl₃) Phenyl 3,4,6-tri-*O*-benzoyl-1-thio-β-D-galactopyranoside 28 & Phenyl 2,3,6-tri-*O*-benzoyl-1-thio-β-D-galactopyranoside 29

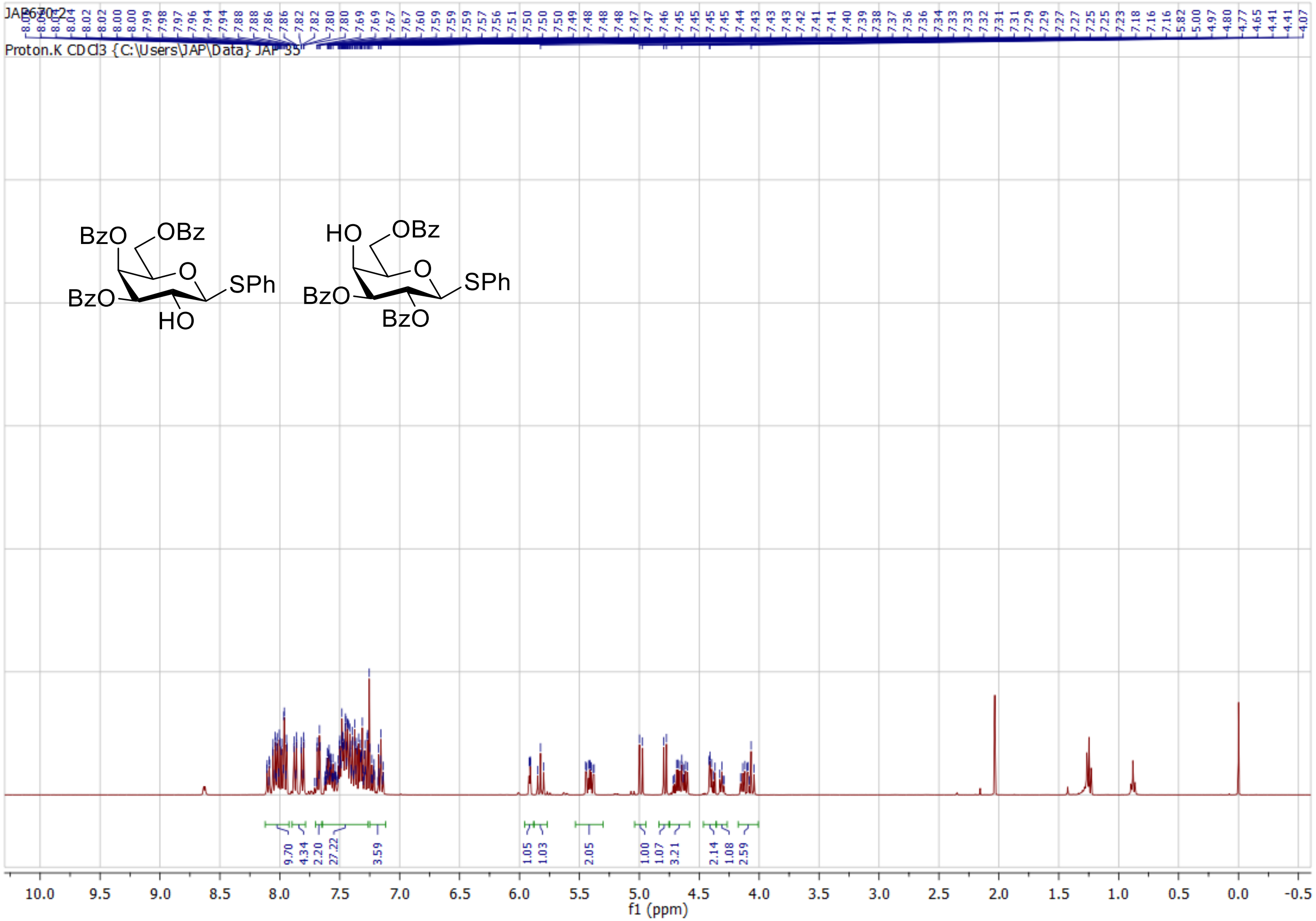

COSY (400 × 400 MHz, CDCl3) Phenyl 3,4,6-tri-*O*-benzoyl-1-thio-β-D-galactopyranoside 28 & Phenyl 2,3,6-tri-*O*-benzoyl-1-thio-β-D-galactopyranoside 29

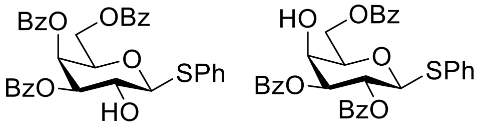

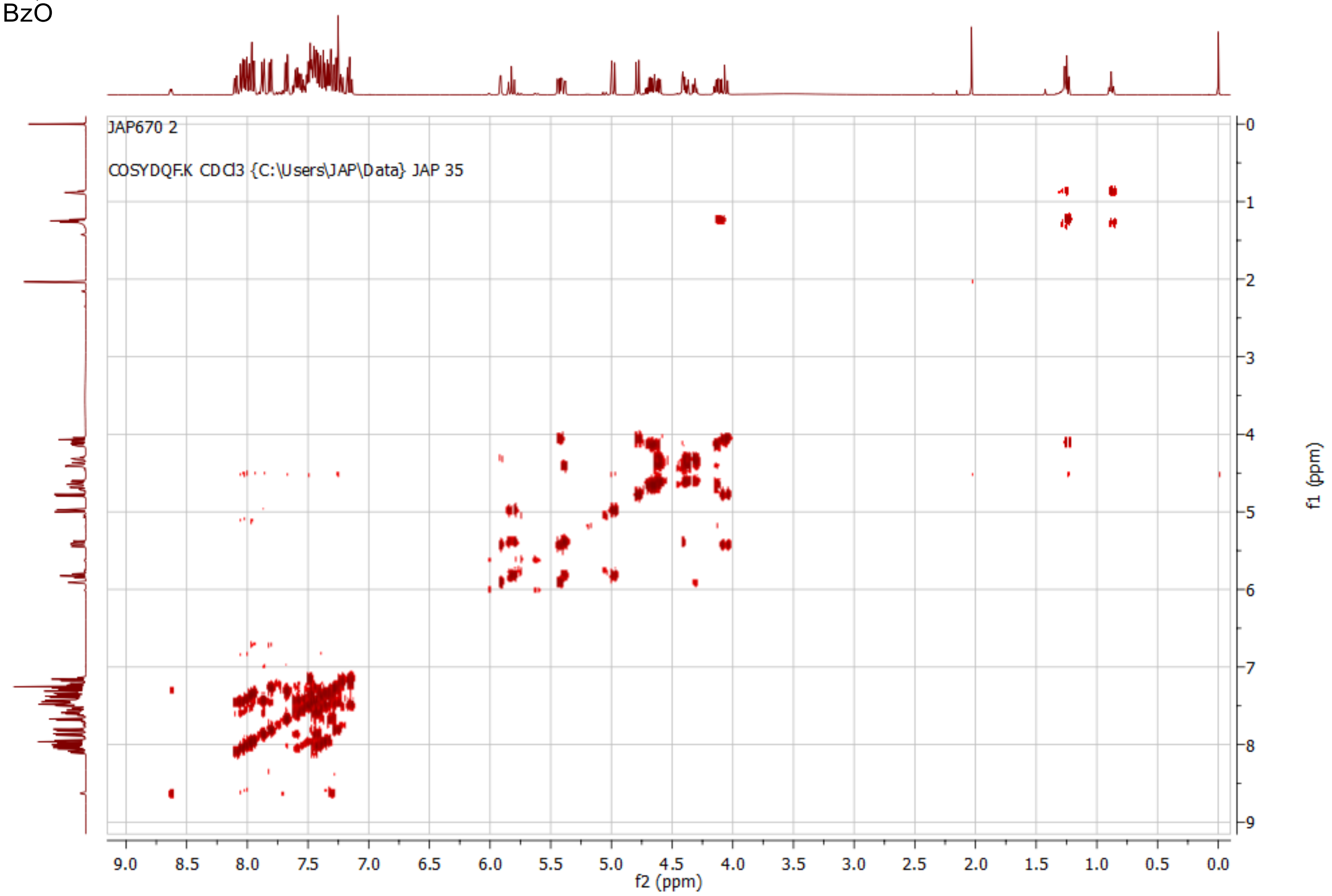

HSQC (400 × 101 MHz, CDCl₃) Phenyl 3,4,6-tri-*O*-benzoyl-1-thio-β-D-galactopyranoside 28 & Phenyl 2,3,6-tri-*O*-benzoyl-1-thio-β-D-galactopyranoside 29

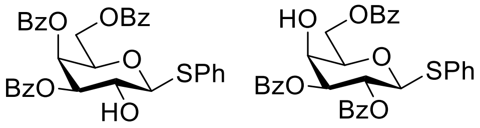

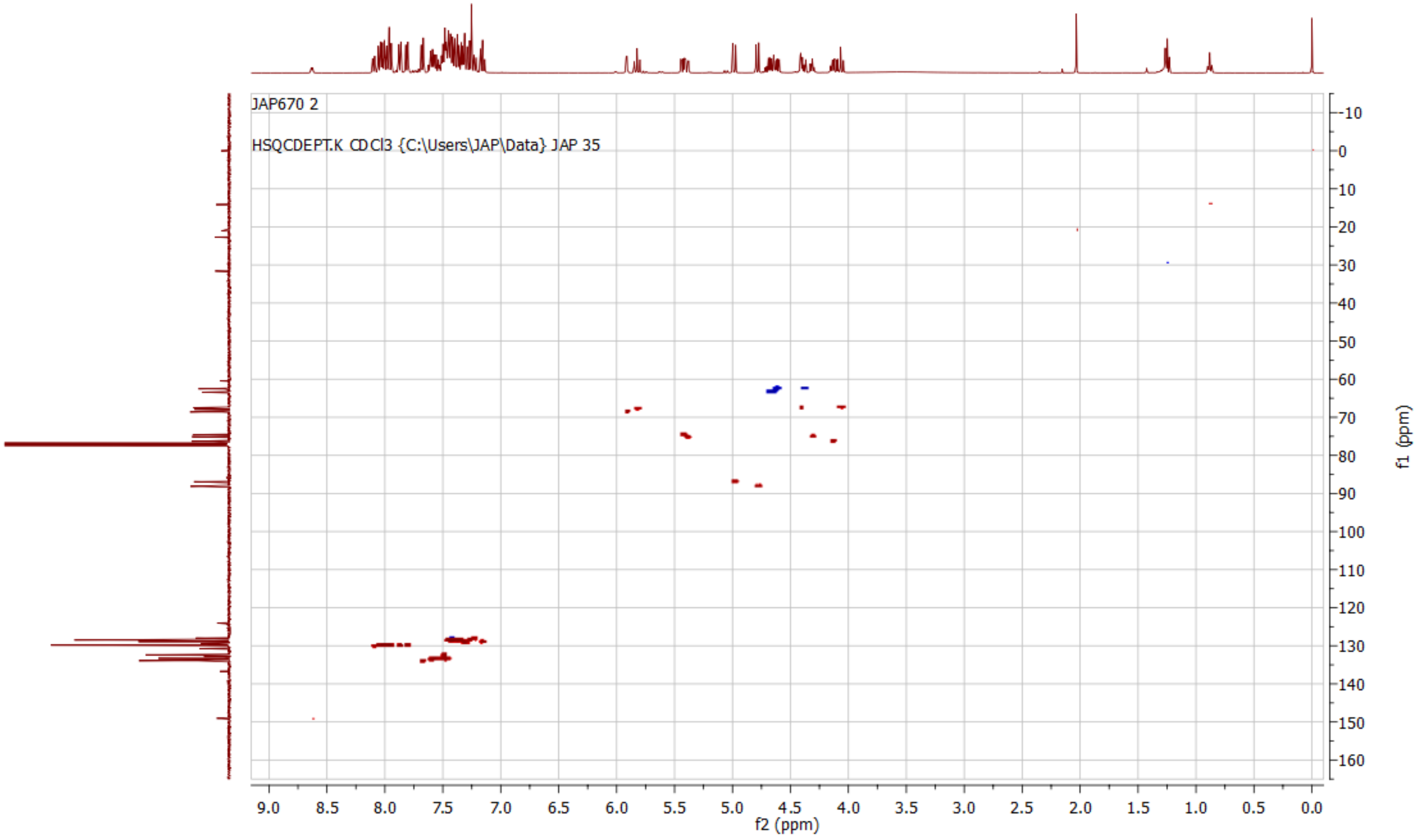

HMBC (400 × 101 MHz, $CDCl_3$) Phenyl 3,4,6-tri-*O*-benzoyl-1-thio-β-D-galactopyranoside 28 & Phenyl 2,3,6-tri-*O*-benzoyl-1-thio-β-D-galactopyranoside 29

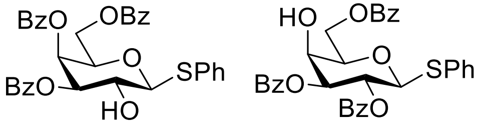

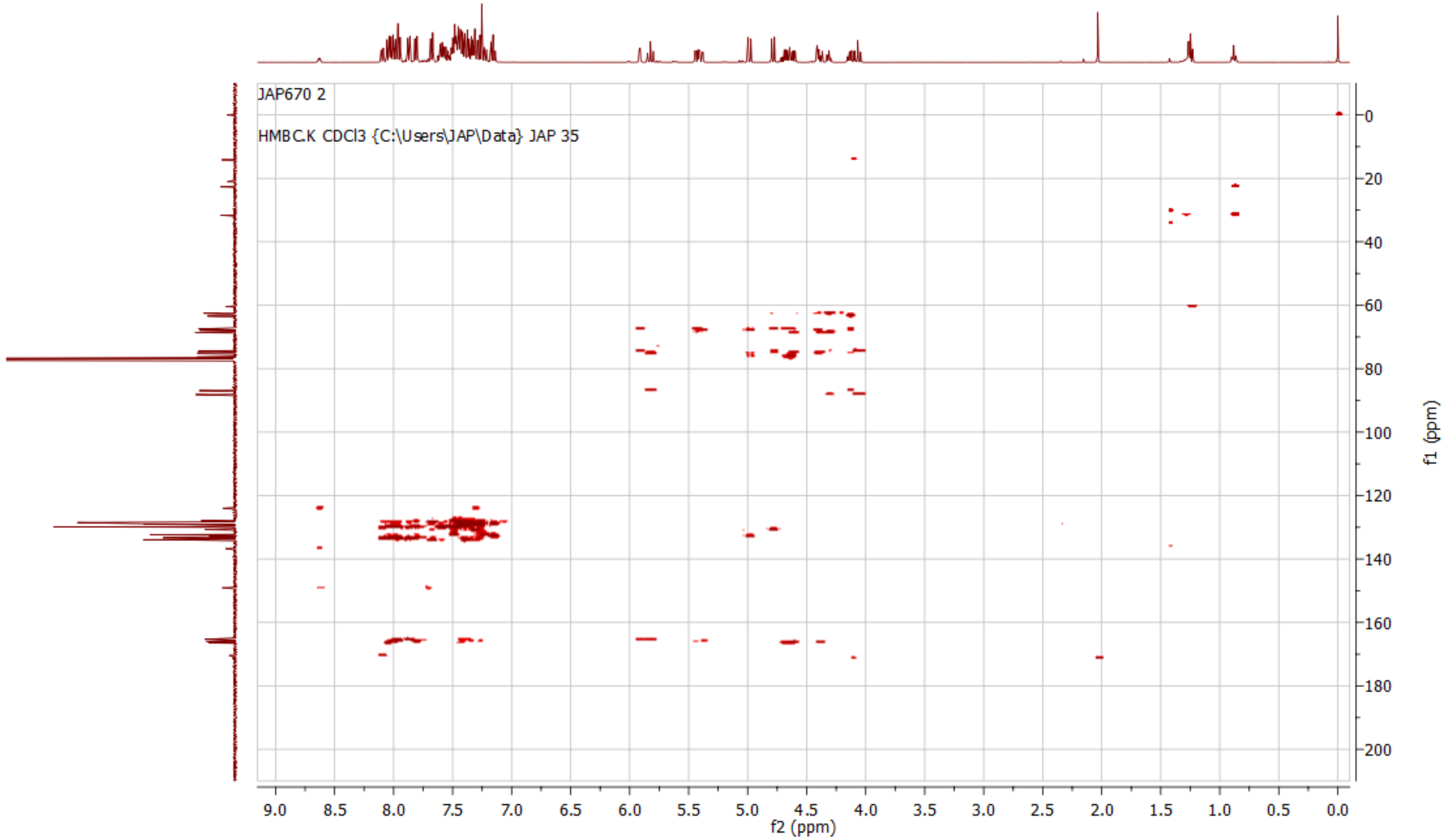

^{13}C{1H} NMR (101 MHz, CDCl$_3$) Phenyl 3,4,6-tri-*O*-benzoyl-1-thio-β-D-galactopyranoside 28 & Phenyl 2,3,6-tri-*O*-benzoyl-1-thio-β-D-galactopyranoside 29

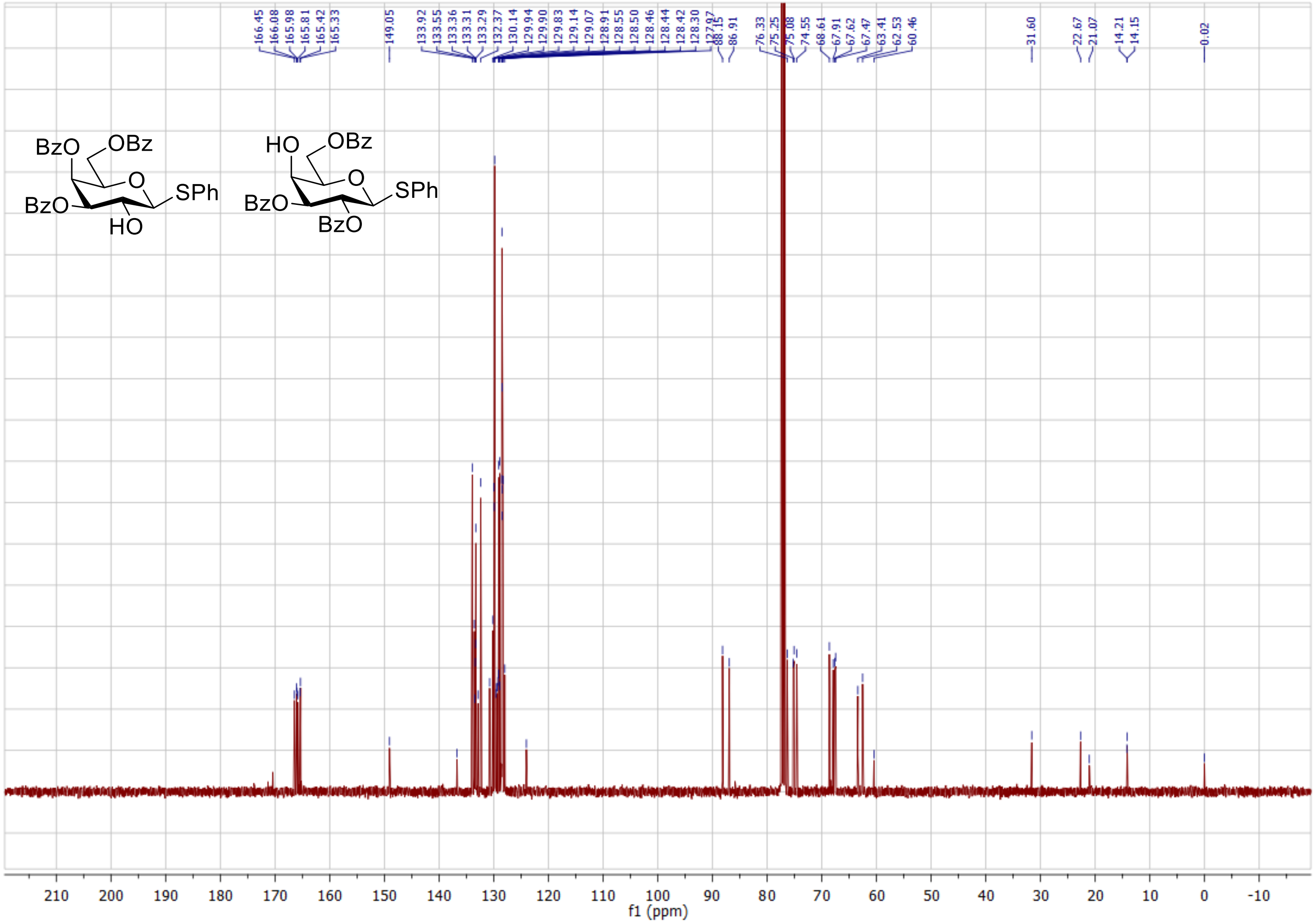

Compound 30

^{1}H NMR (400 MHz, $CDCl_3$) Phenyl 3,6-di-*O*-benzoyl-1-thio-β-ᴅ-galactopyranoside 30

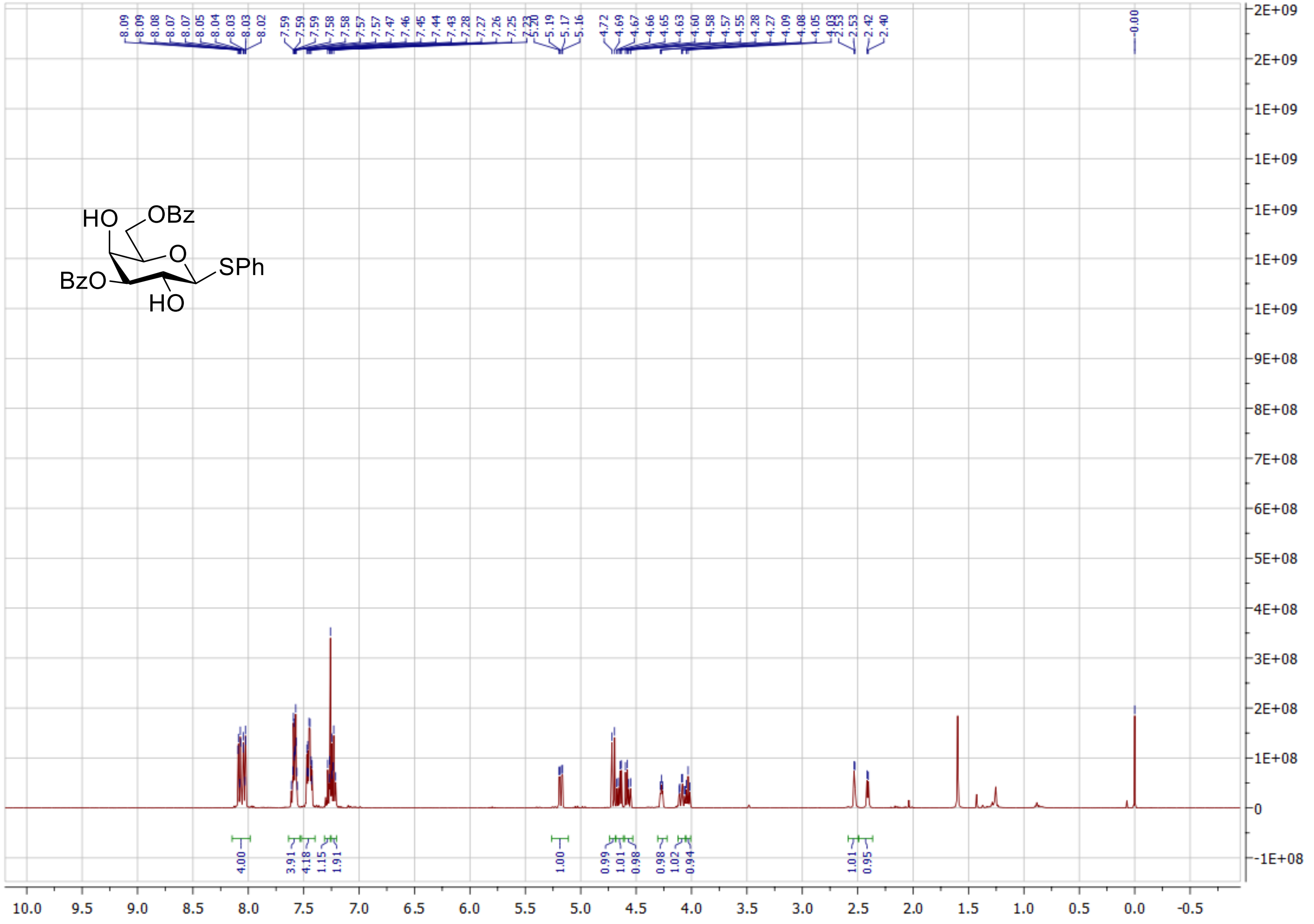

COSY (400 × 400 MHz, CDCl₃) Phenyl 3,6-di-*O*-benzoyl-1-thio-β-D-galactopyranoside 30

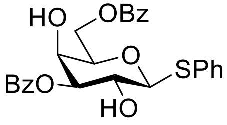

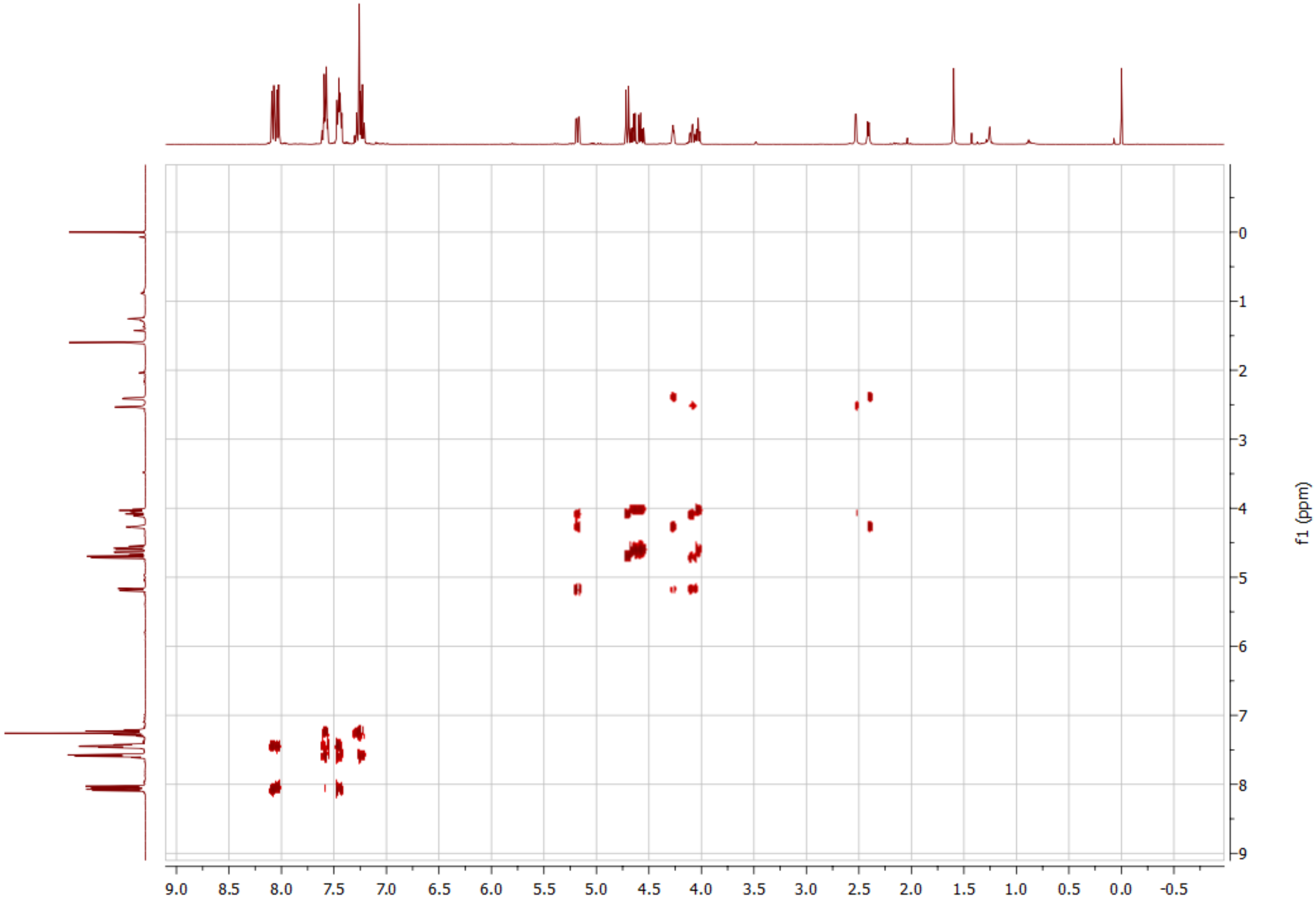

HSQC (400 × 101 MHz, $CDCl_3$) Phenyl 3,6-di-*O*-benzoyl-1-thio-β-D-galactopyranoside 30

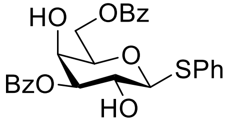

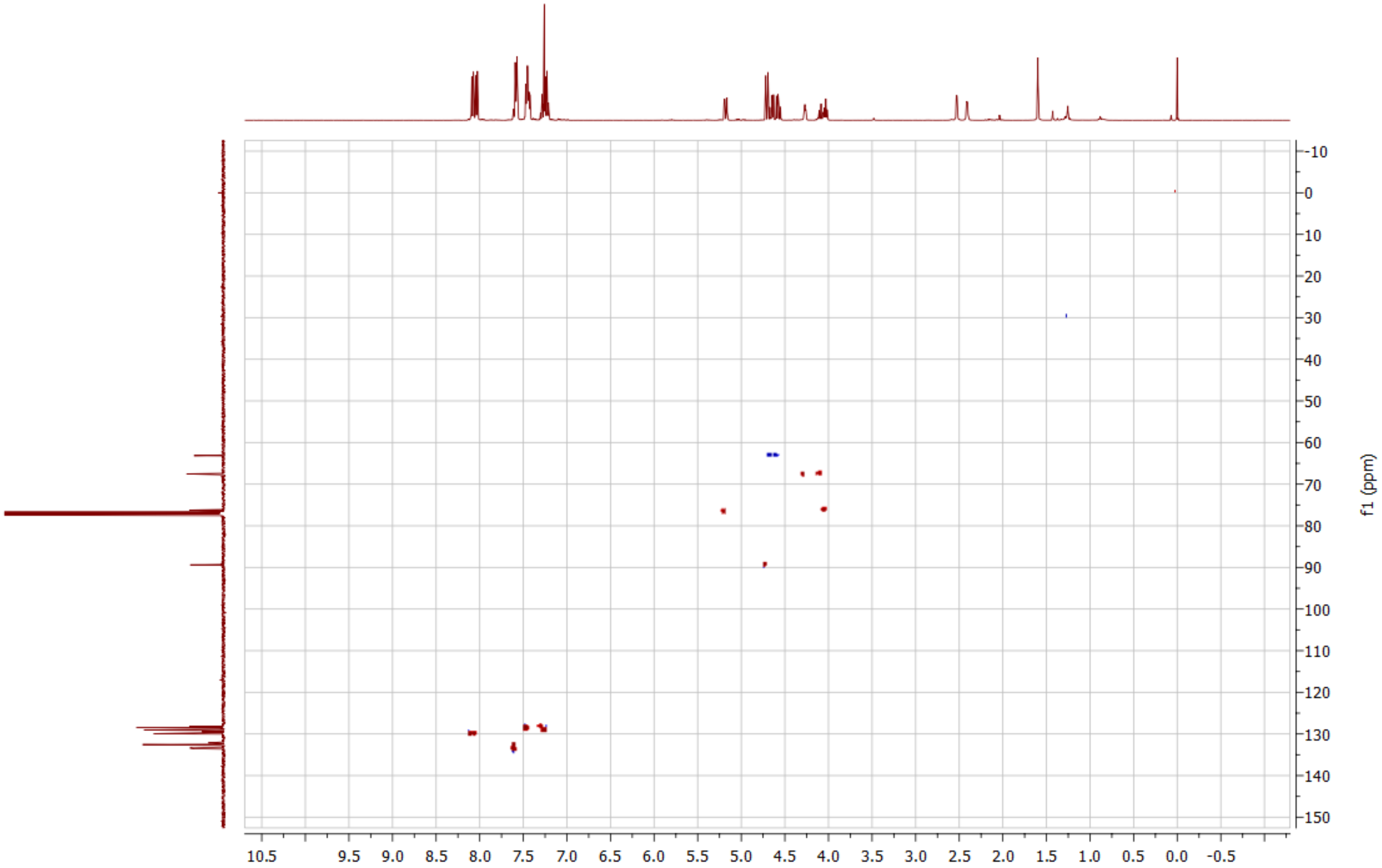

HMBC (400 × 101 MHz, $CDCl_3$) Phenyl 3,6-di-*O*-benzoyl-1-thio-β-D-galactopyranoside 30

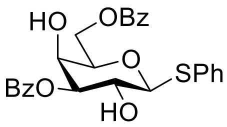

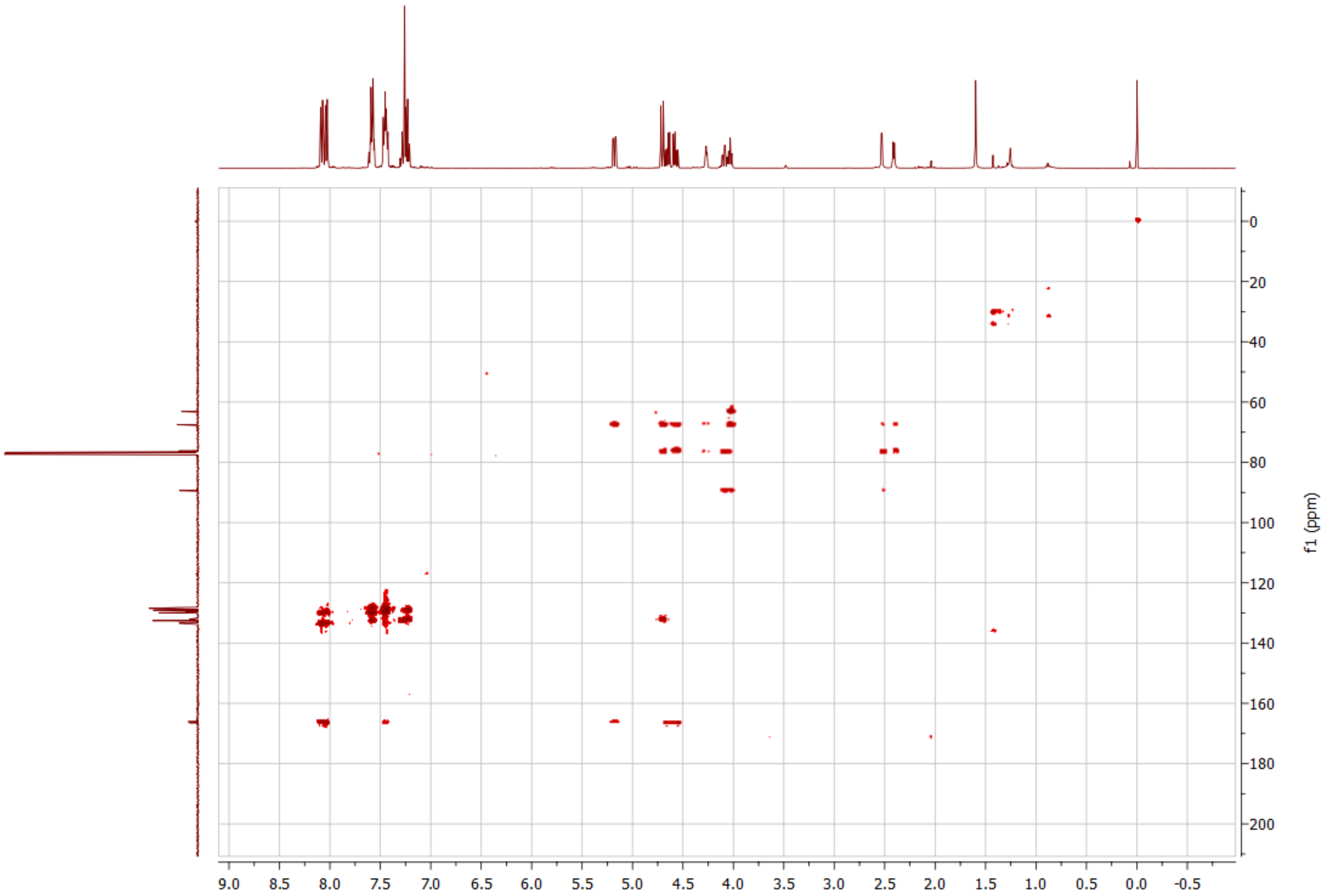

^{1}H NMR (400 MHz, CDCl₃) Phenyl 3,6-di-*O*-benzoyl-1-thio-β-D-galactopyranoside 30

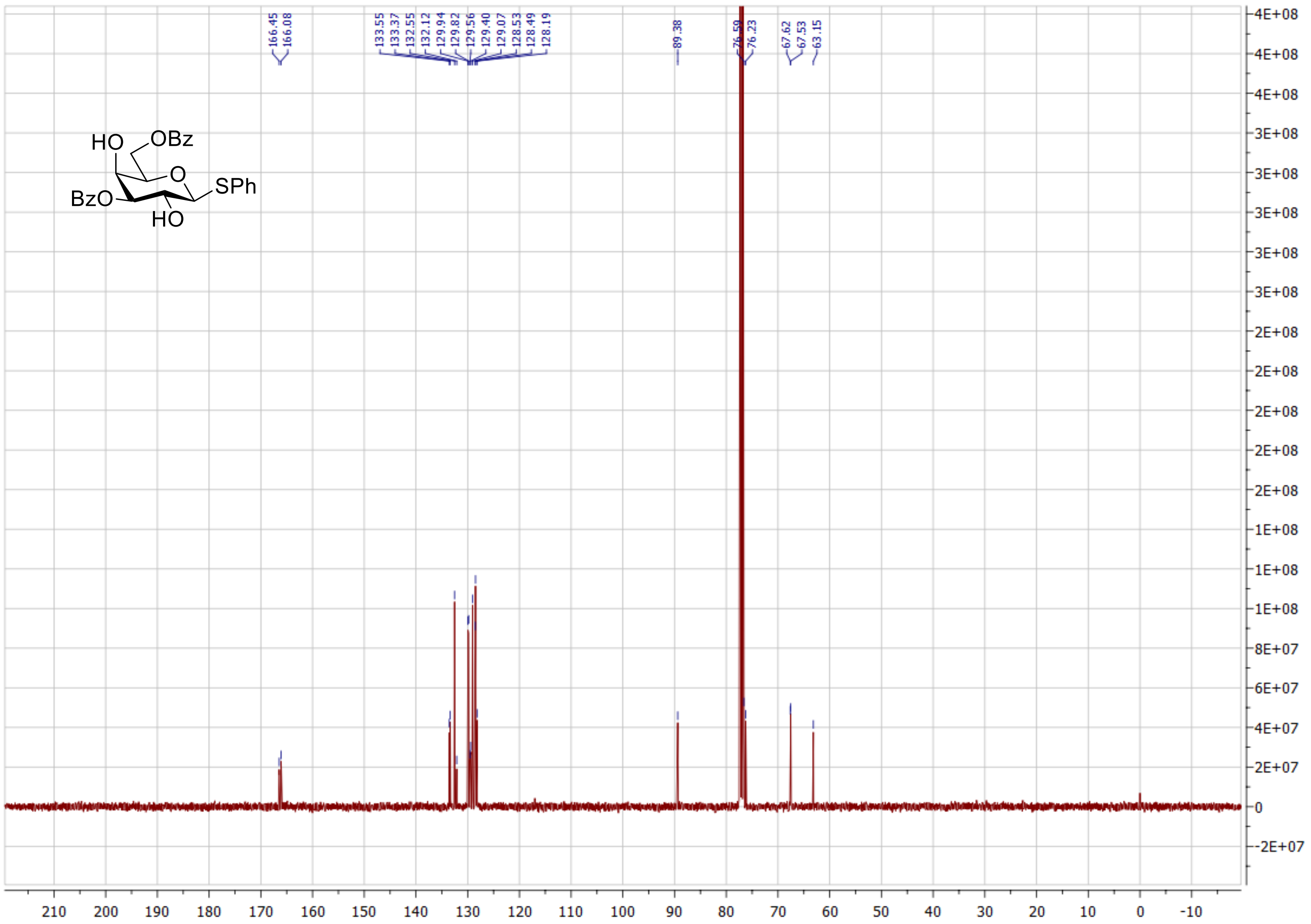

Compound 32

^{1}H NMR (400 MHz, CDCl3) Ethyl 2,3,4,6-tetra-*O*-benzoyl-1-thio-β-D-galactopyranoside 32

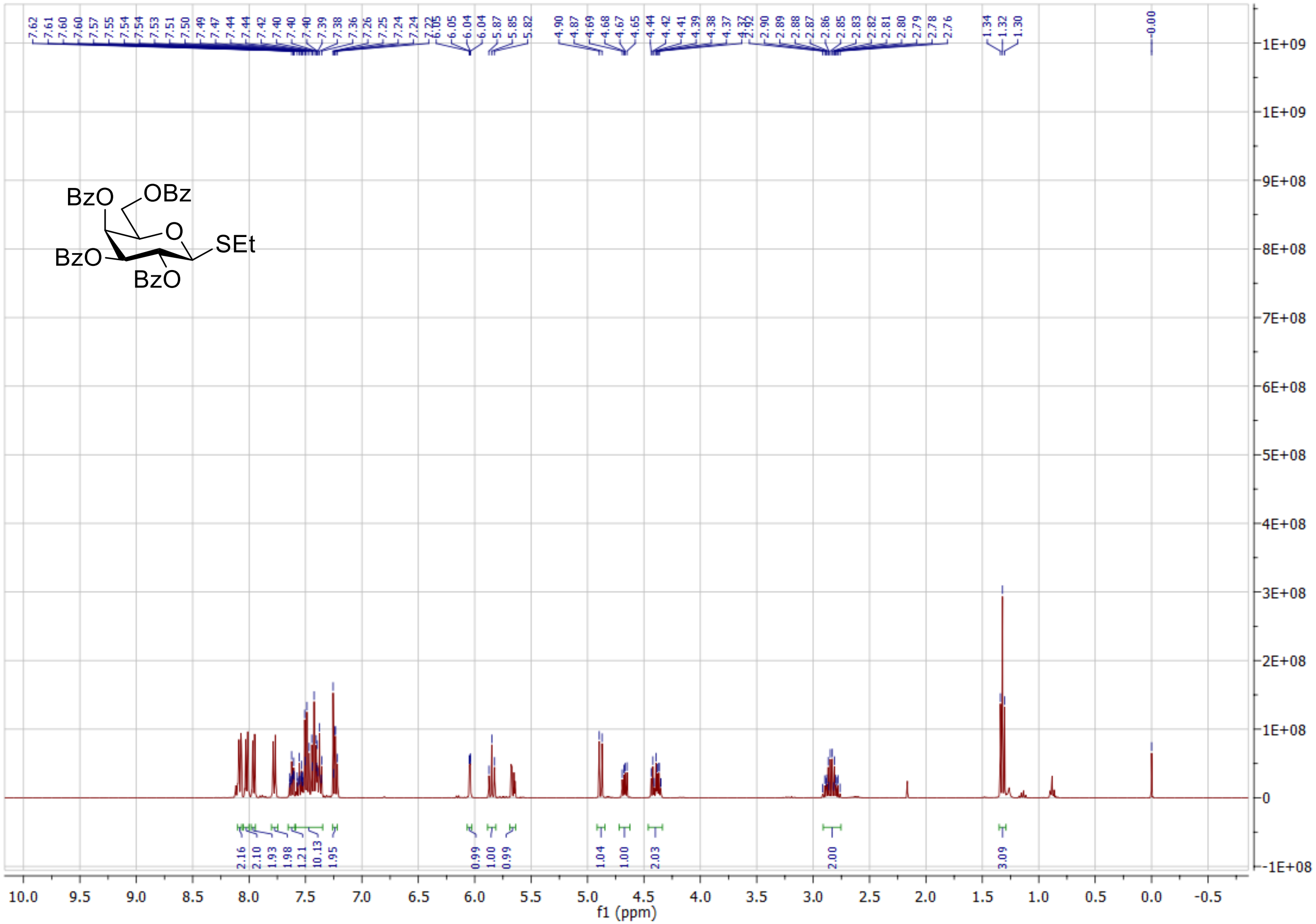

$^{13}C\{1H\}$ NMR (101 MHz, $CDCl_3$) Ethyl 2,3,4,6-tetra-*O*-benzoyl-1-thio-β-D-galactopyranoside 32

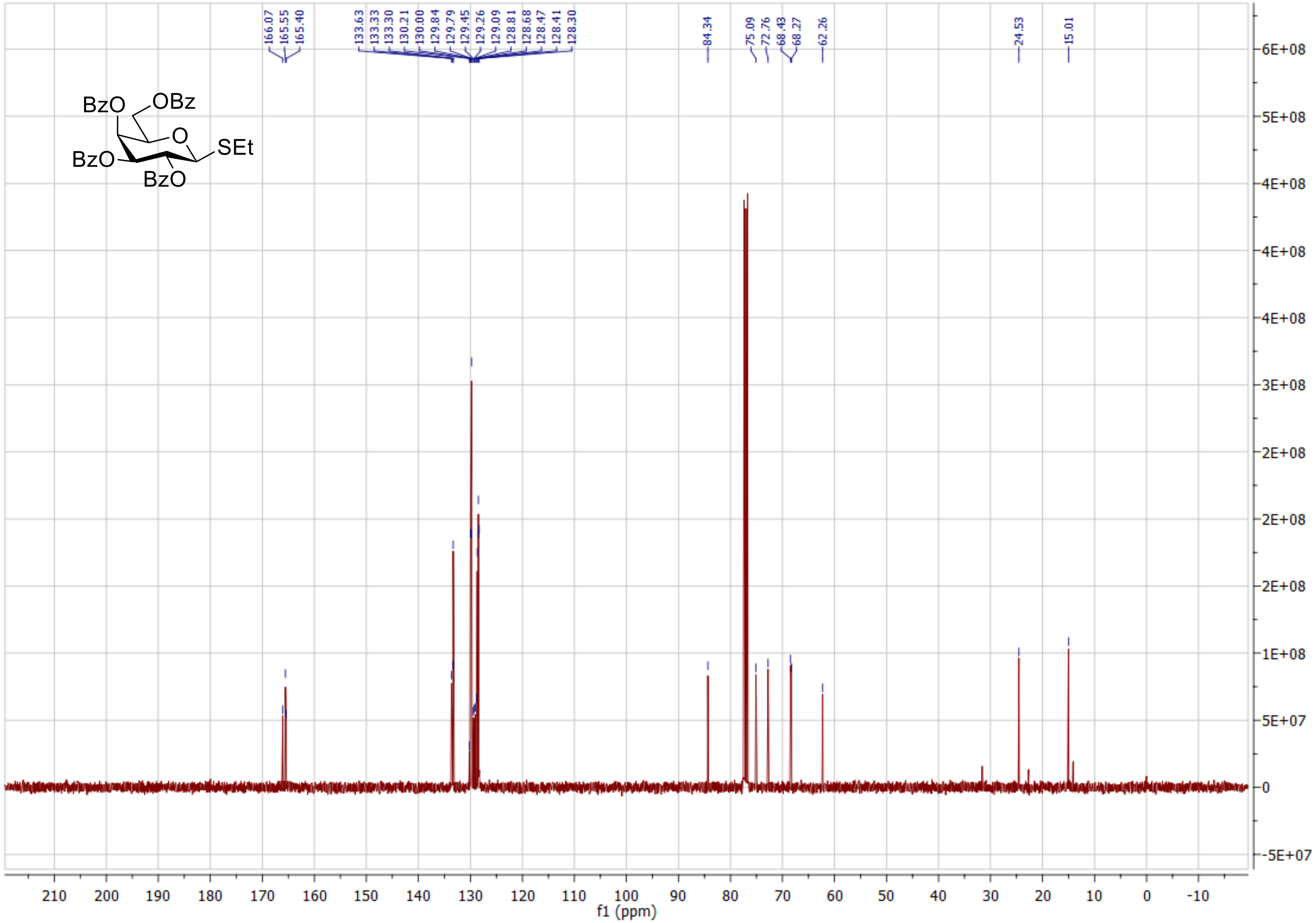

Compound 34

^{1}H NMR (400 MHz, $CDCl_3$) Ethyl 2,3,6-tri-*O*-benzoyl-1-thio-β-D-galactopyranoside 34

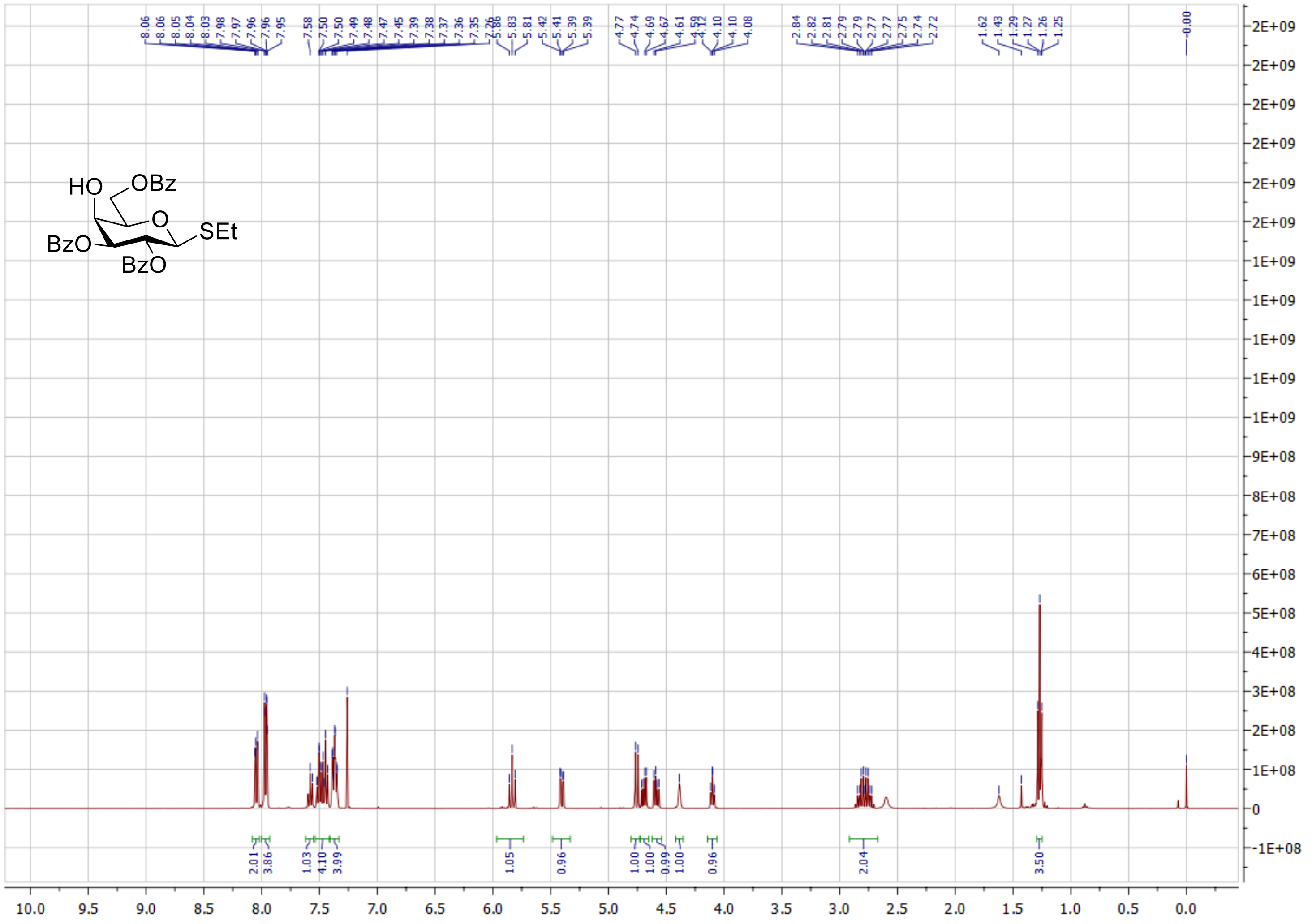

COSY (400 × 400 MHz, $CDCl_3$) Ethyl 2,3,6-tri-*O*-benzoyl-1-thio-β-D-galactopyranoside 34

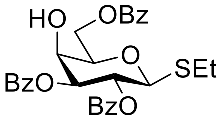

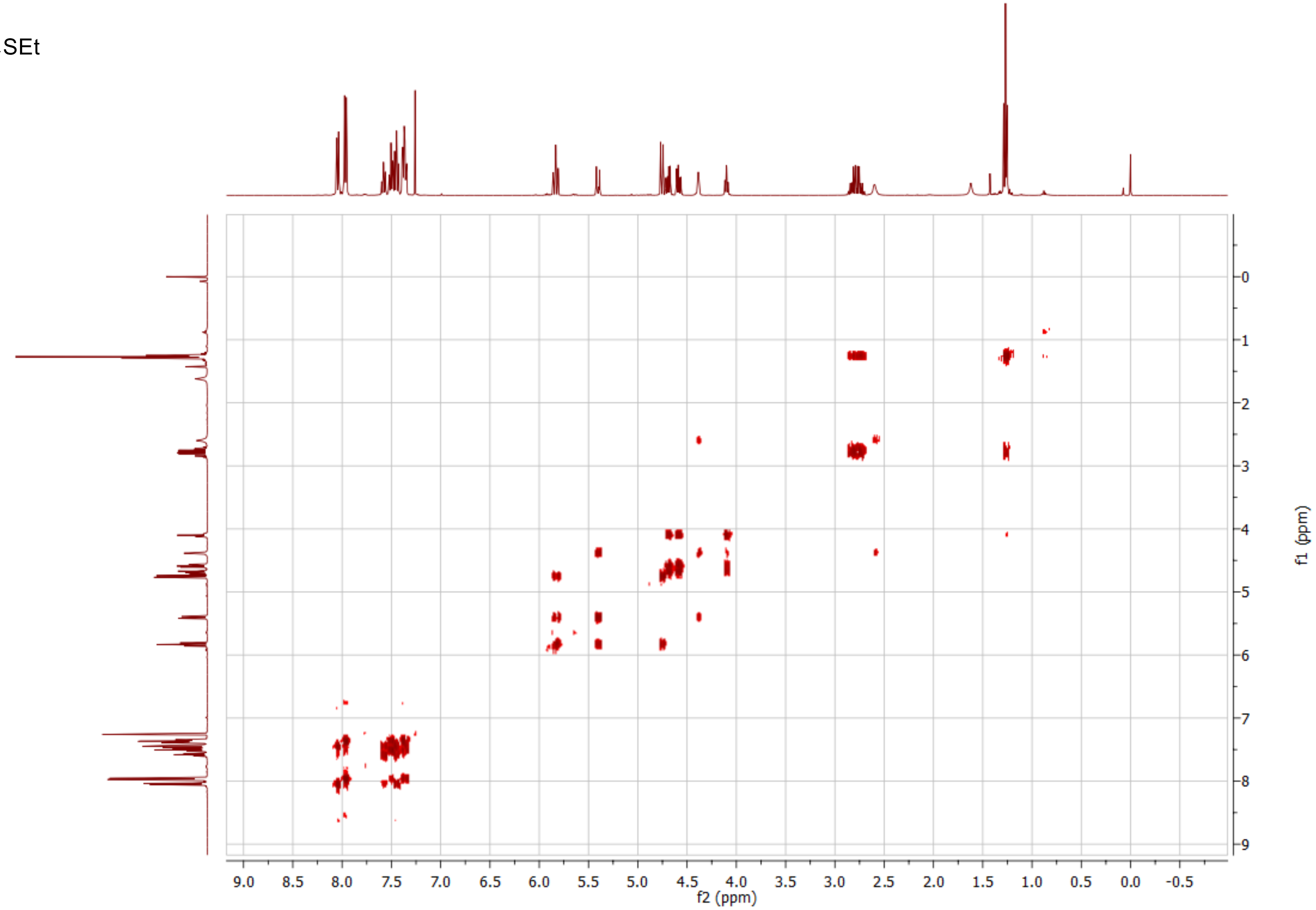

HSQC (400 × 101 MHz, CDCl₃) Ethyl 2,3,6-tri-*O*-benzoyl-1-thio-β-D-galactopyranoside 34

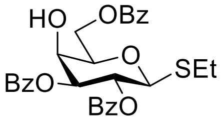

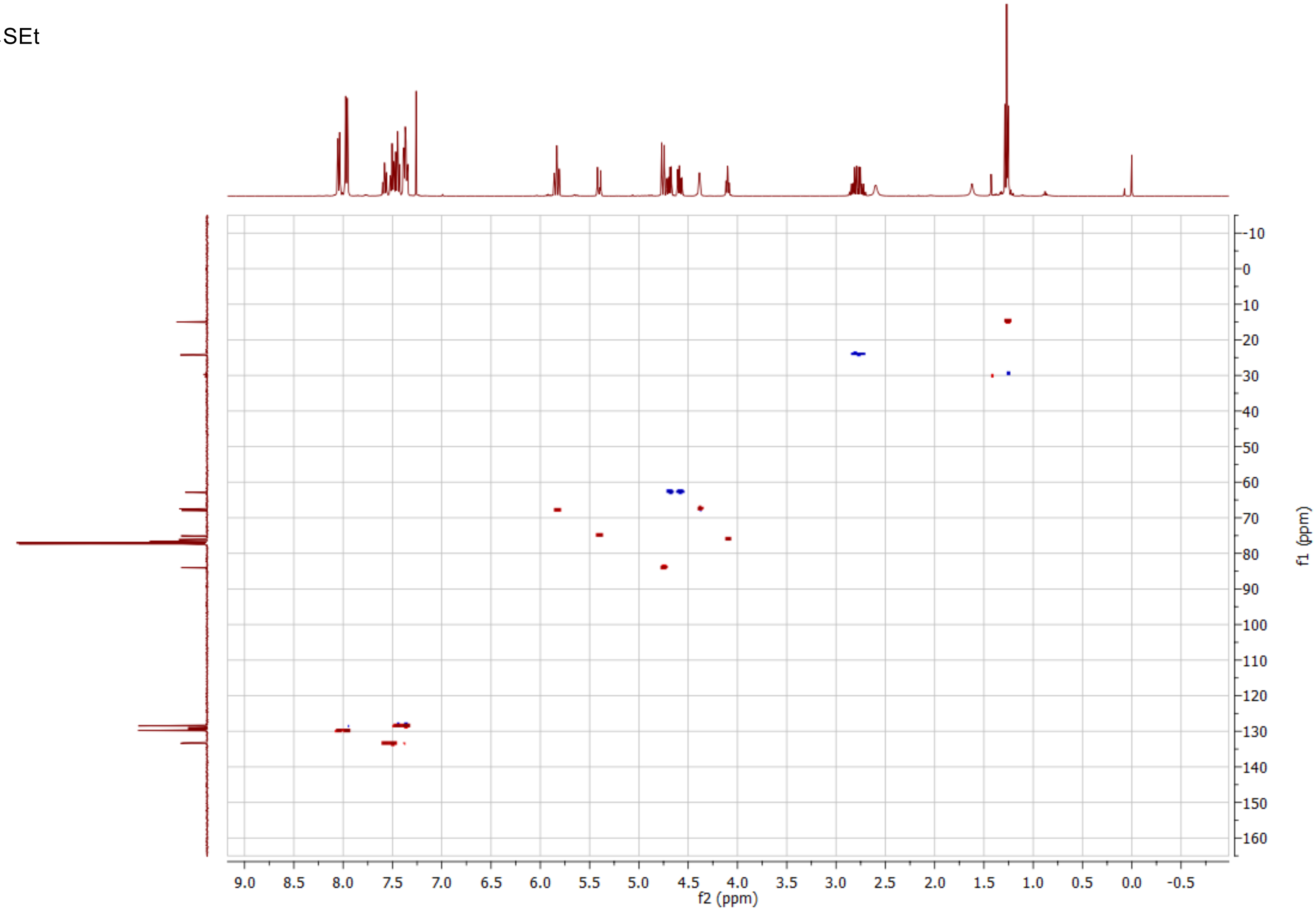

HMBC (400 × 101 MHz, CDCl3) Ethyl 2,3,6-tri-*O*-benzoyl-1-thio-β-D-galactopyranoside 34

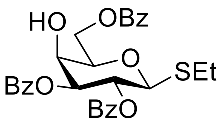

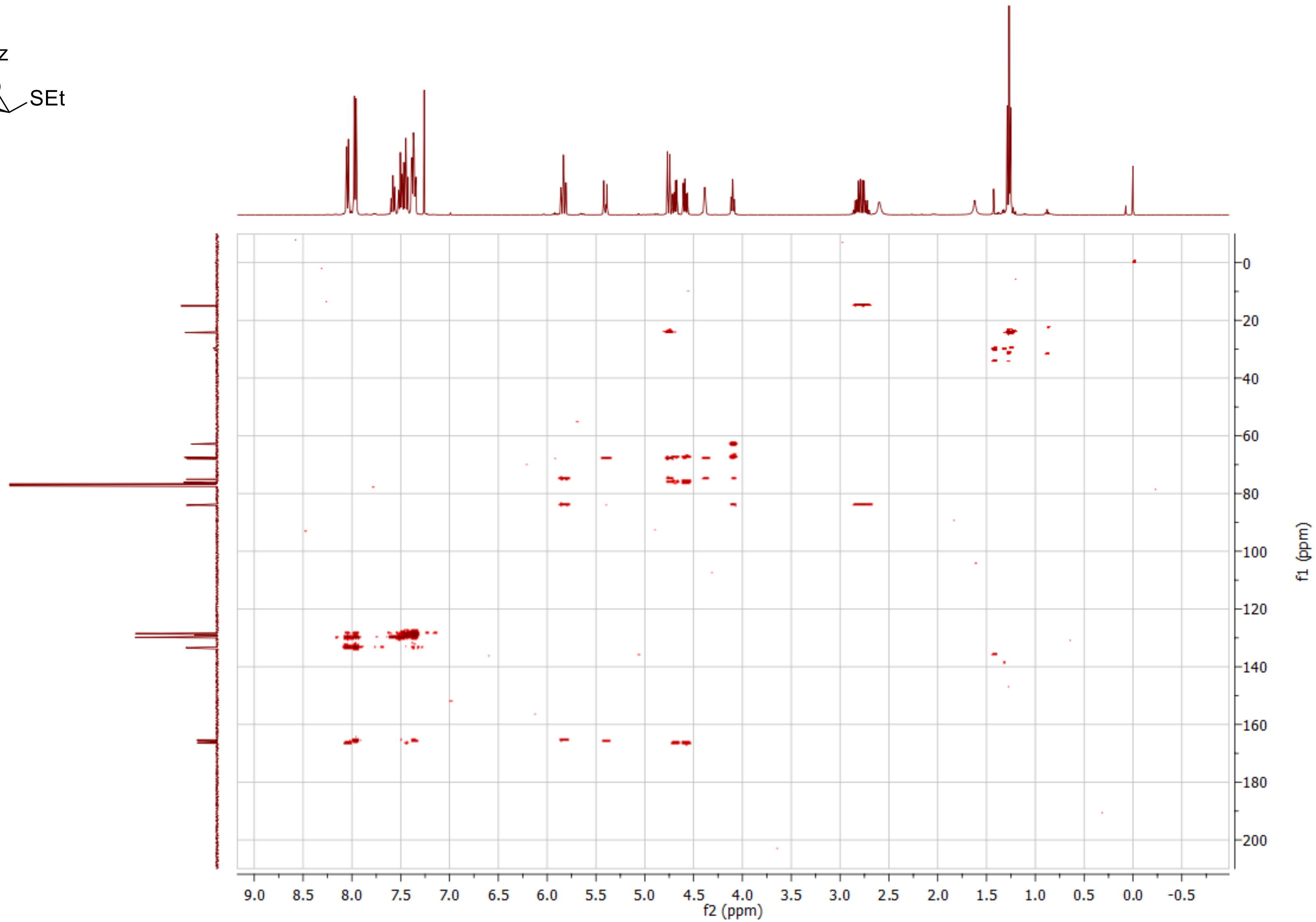

$^{13}C\{1H\}$ NMR (101 MHz, $CDCl_3$) Ethyl 2,3,6-tri-*O*-benzoyl-1-thio-β-D-galactopyranoside 34

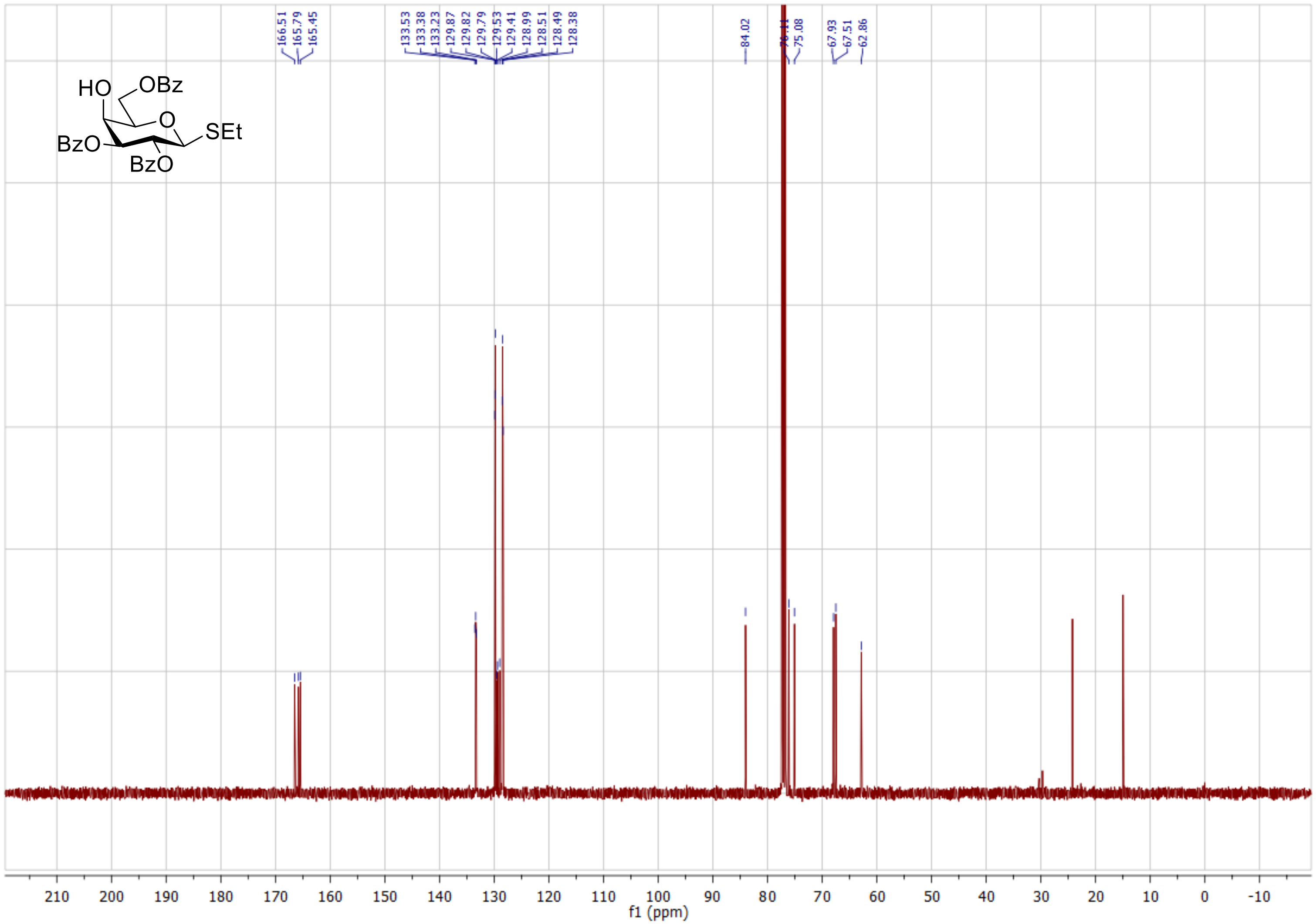

Compound 33

^{1}H NMR (400 MHz, $CDCl_3$) Ethyl 3,4,6-tri-*O*-benzoyl-1-thio-β-D-galactopyranoside 33

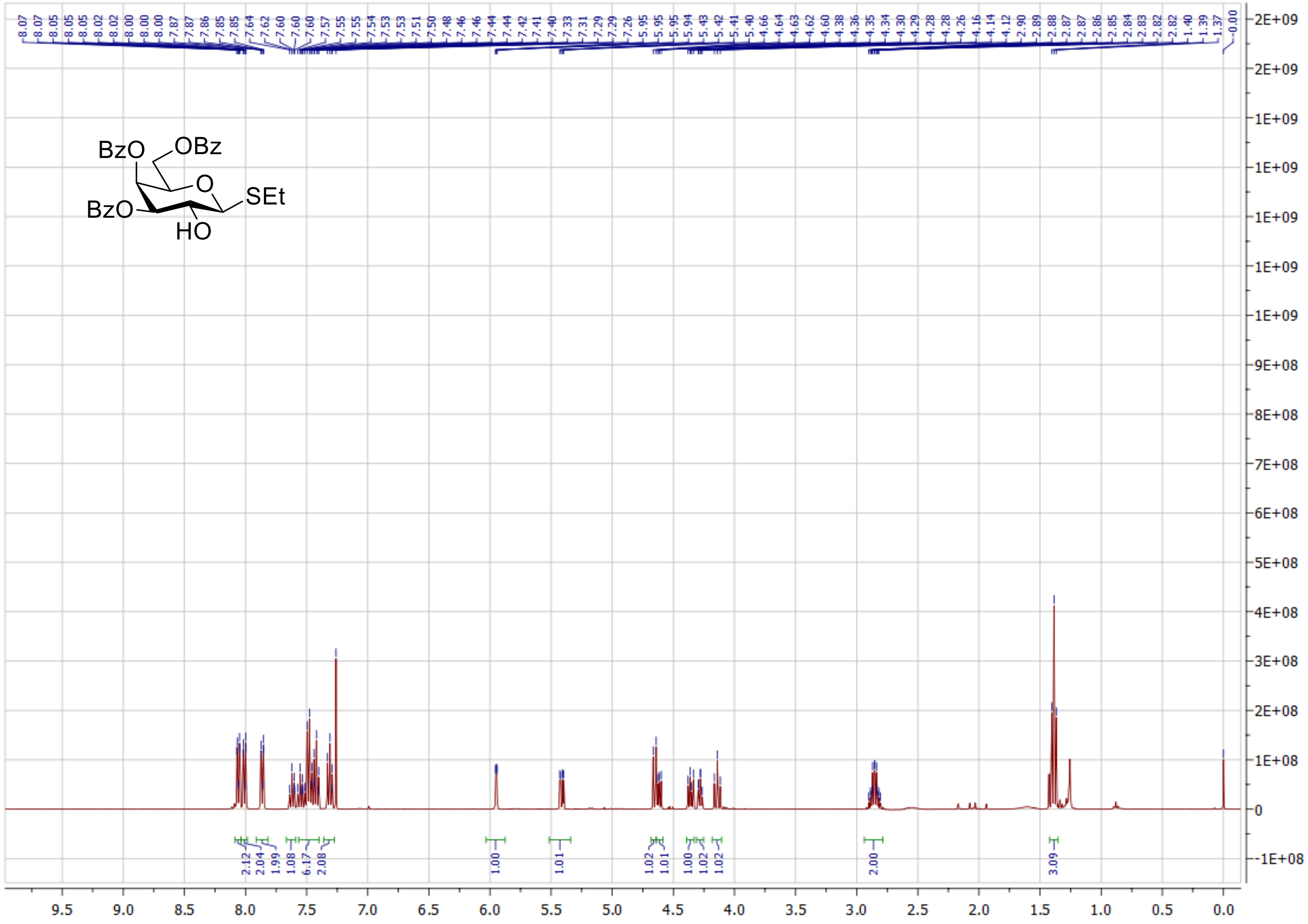

COSY (400 × 400 MHz, $CDCl_3$) Ethyl 3,4,6-tri-*O*-benzoyl-1-thio-β-D-galactopyranoside 33

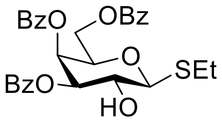

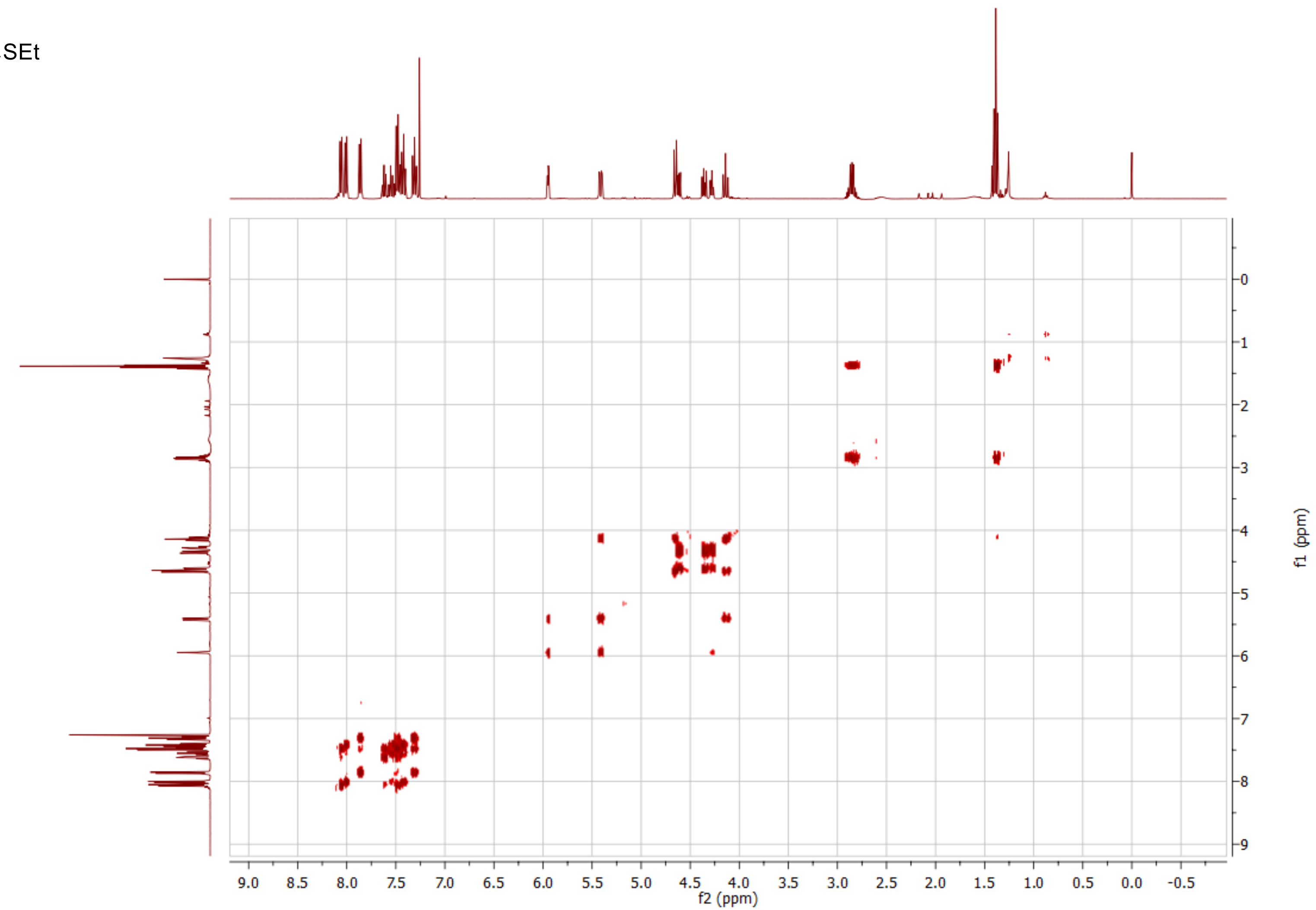

HSQC (400 × 101 MHz, $CDCl_3$) Ethyl 3,4,6-tri-*O*-benzoyl-1-thio-β-D-galactopyranoside 33

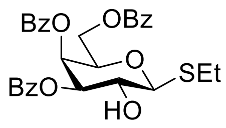

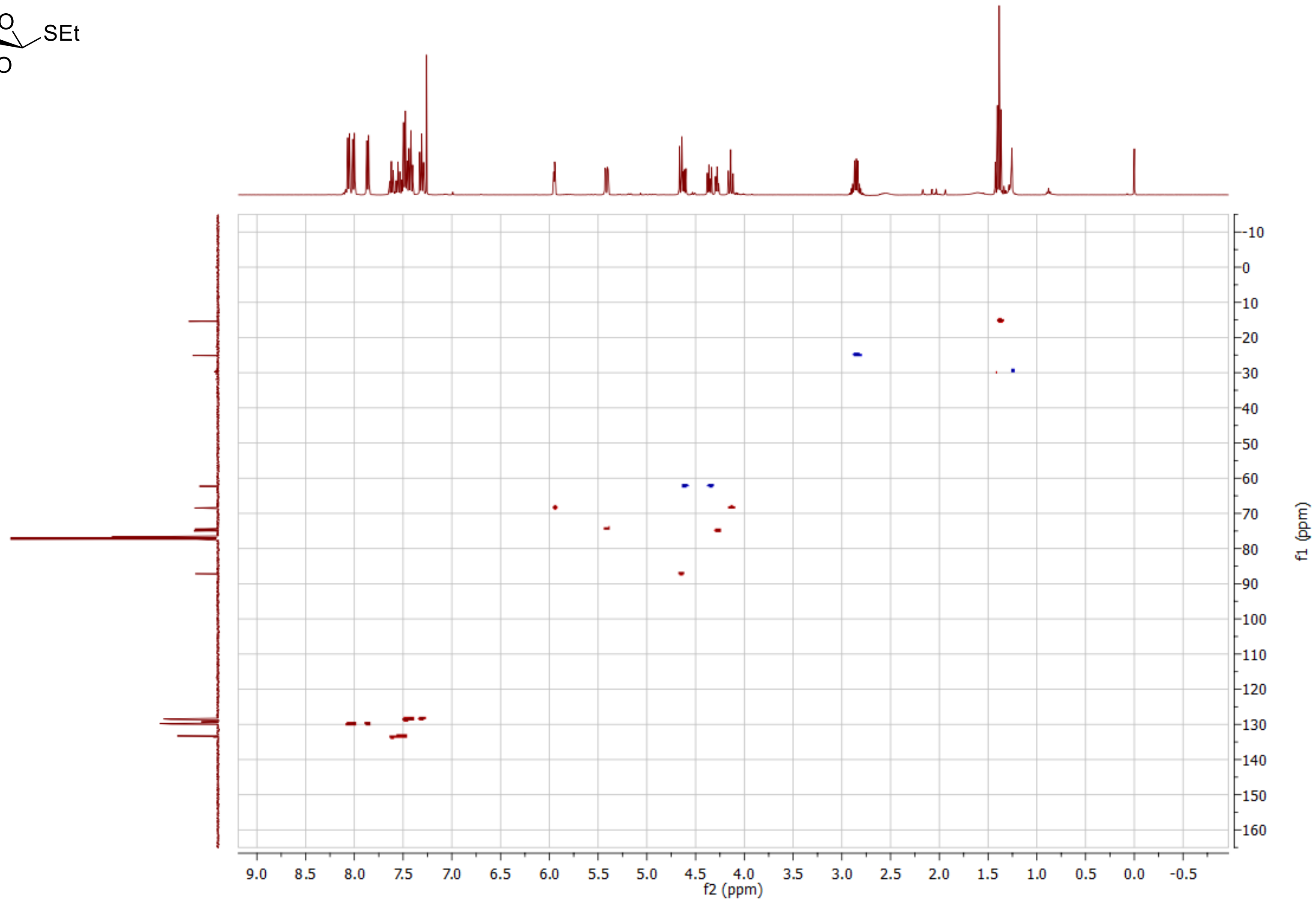

HMBC (400 × 101 MHz, CDCl₃) Ethyl 3,4,6-tri-*O*-benzoyl-1-thio-β-D-galactopyranoside 33

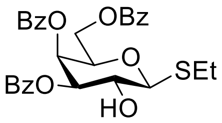

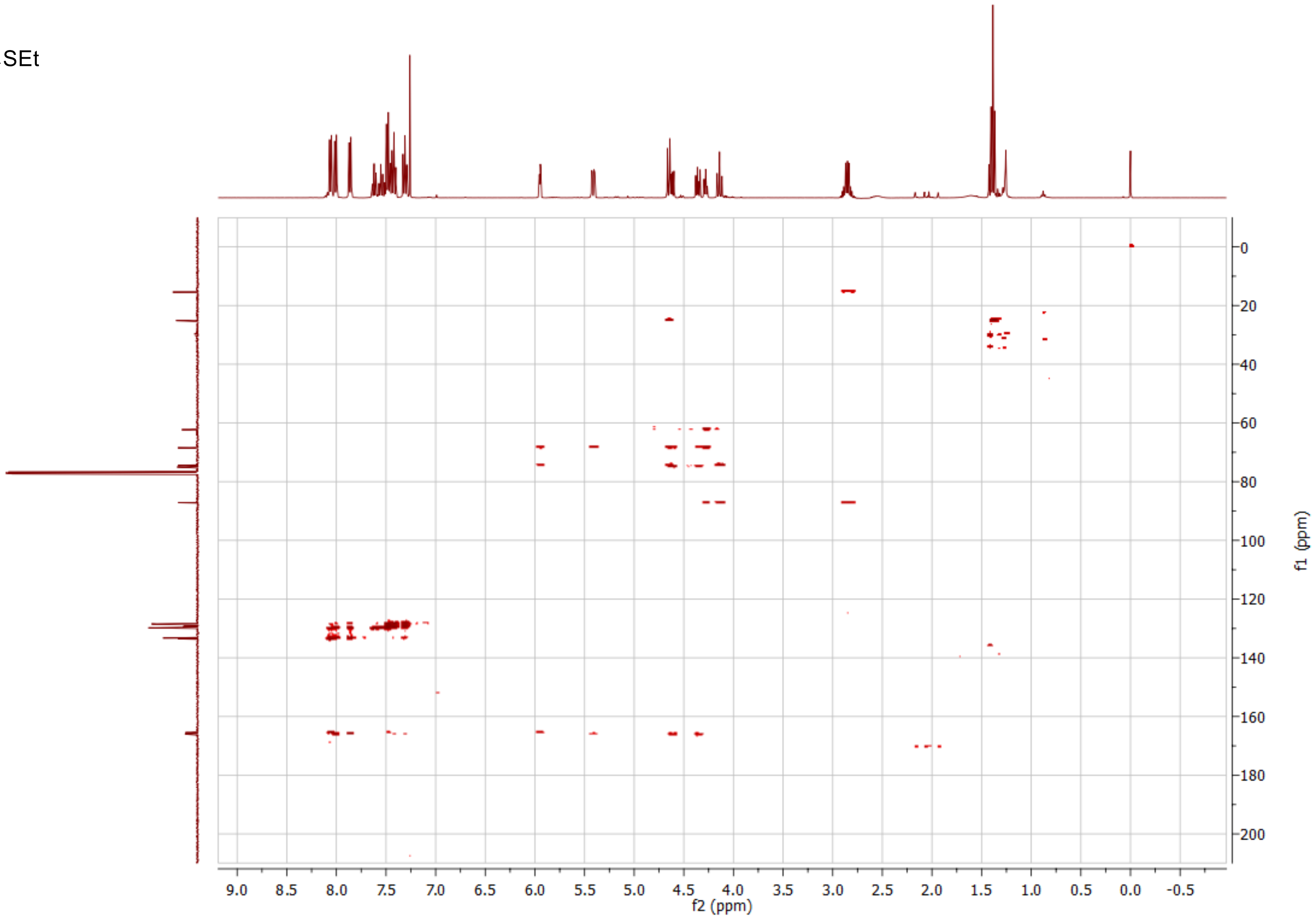

$^{13}C\{1H\}$ NMR (101 MHz, $CDCl_3$) Ethyl 3,4,6-tri-*O*-benzoyl-1-thio-β-D-galactopyranoside 33

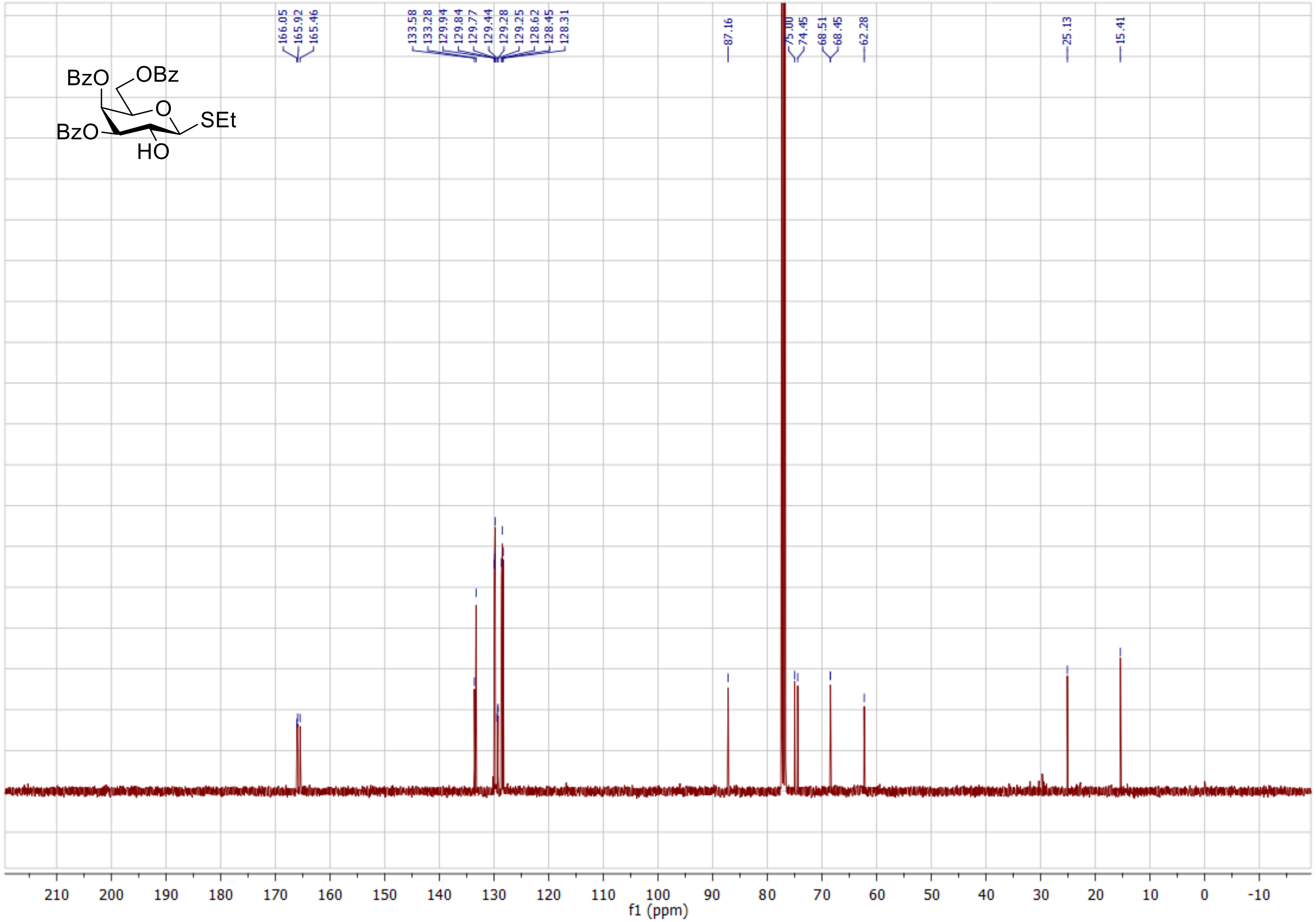

Compound 35

^{1}H NMR (400 MHz, $CDCl_3$) Ethyl 2,6-di-*O*-benzoyl-1-thio-β-D-galactopyranoside 35

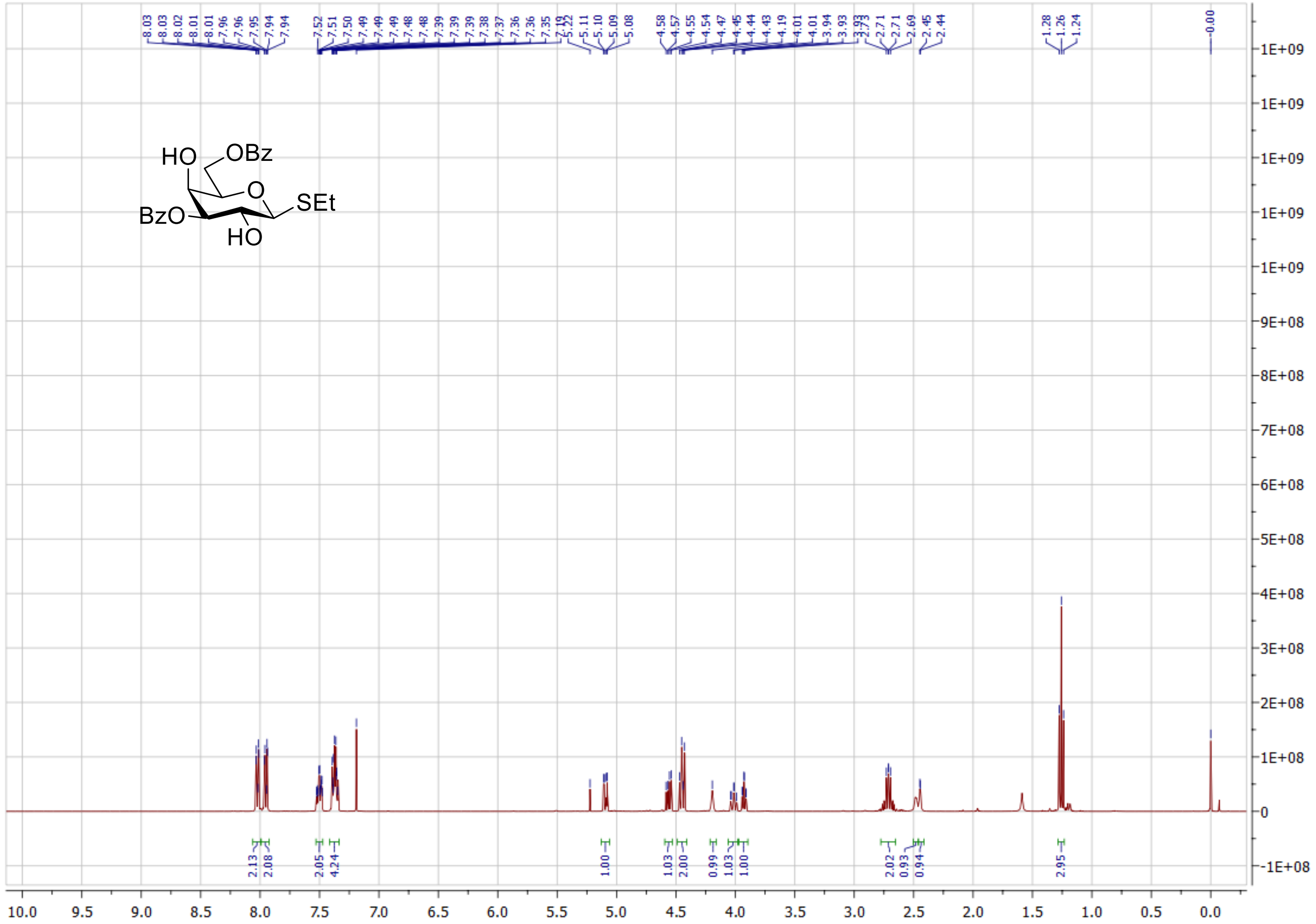

COSY (400 × 400 MHz, $CDCl_3$) Ethyl 2,6-di-*O*-benzoyl-1-thio-β-D-galactopyranoside 35

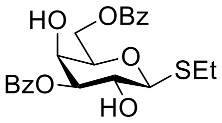

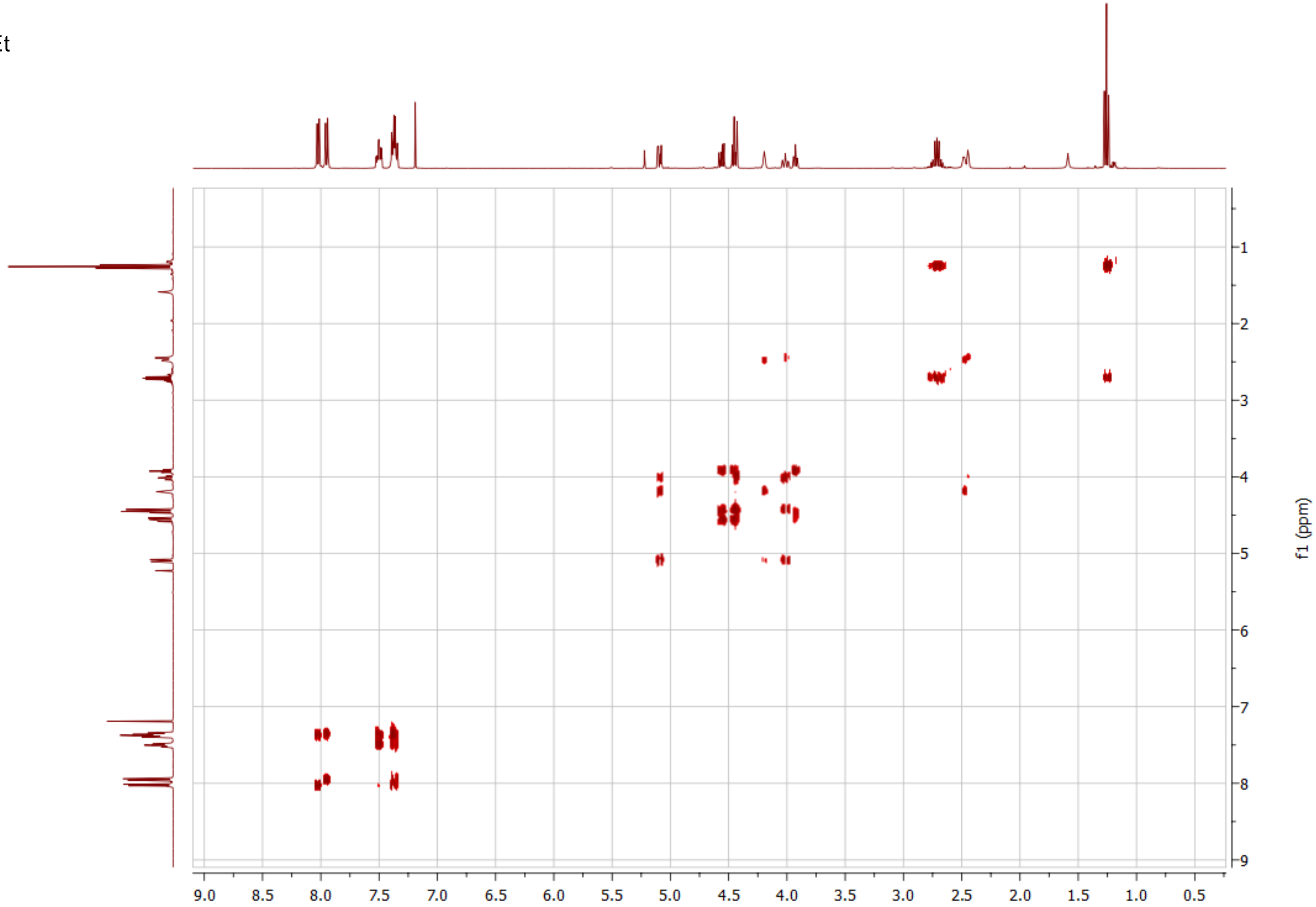

HSQC (400 × 101 MHz, $CDCl_3$) Ethyl 2,6-di-*O*-benzoyl-1-thio-β-D-galactopyranoside 35

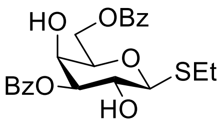

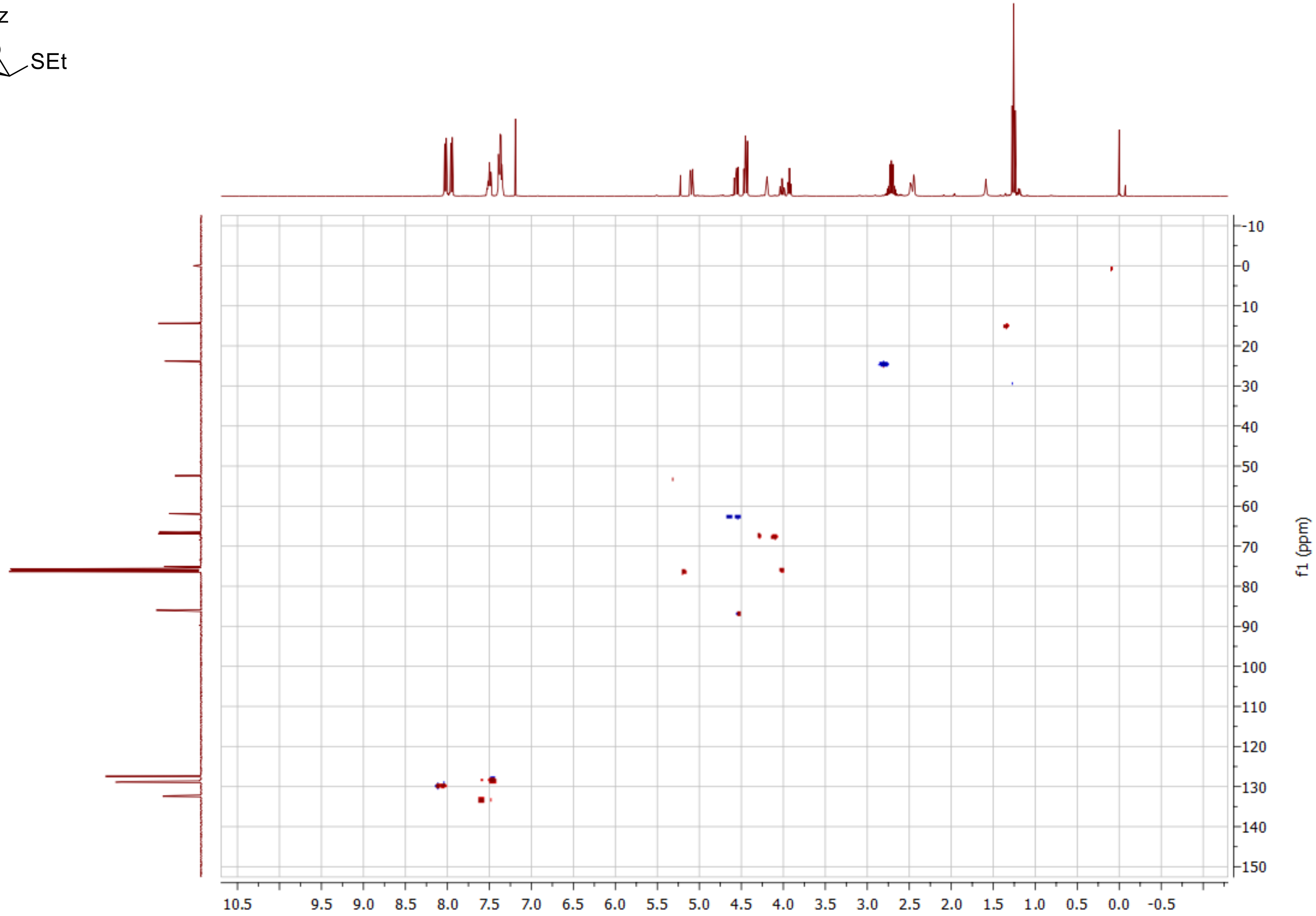

HMBC (400 × 101 MHz, CDCl₃) Ethyl 2,6-di-*O*-benzoyl-1-thio-β-D-galactopyranoside 35

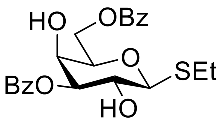

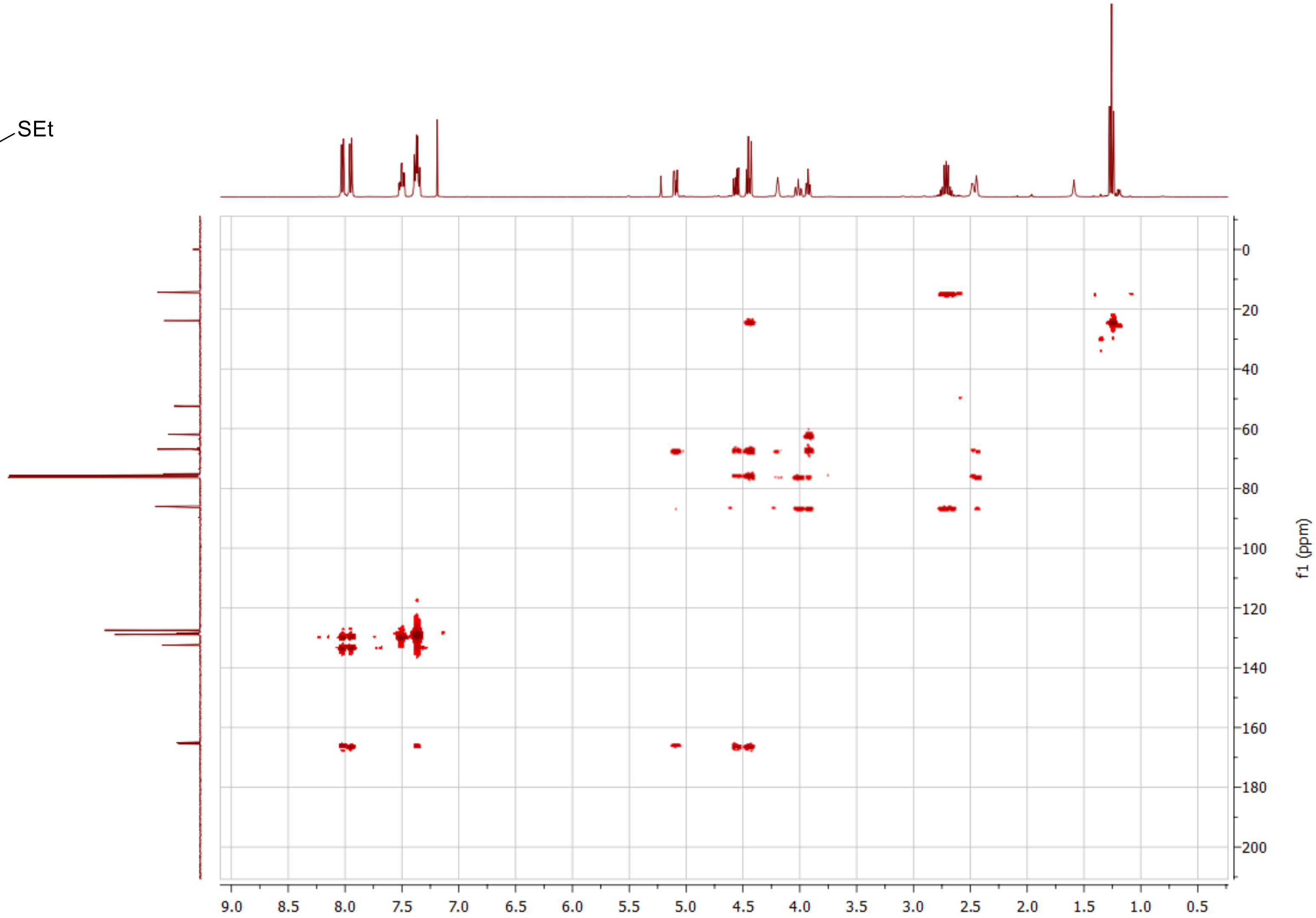

$^{13}C\{1H\}$ NMR (101 MHz, $CDCl_3$) Ethyl 2,6-di-*O*-benzoyl-1-thio-β-D-galactopyranoside 35

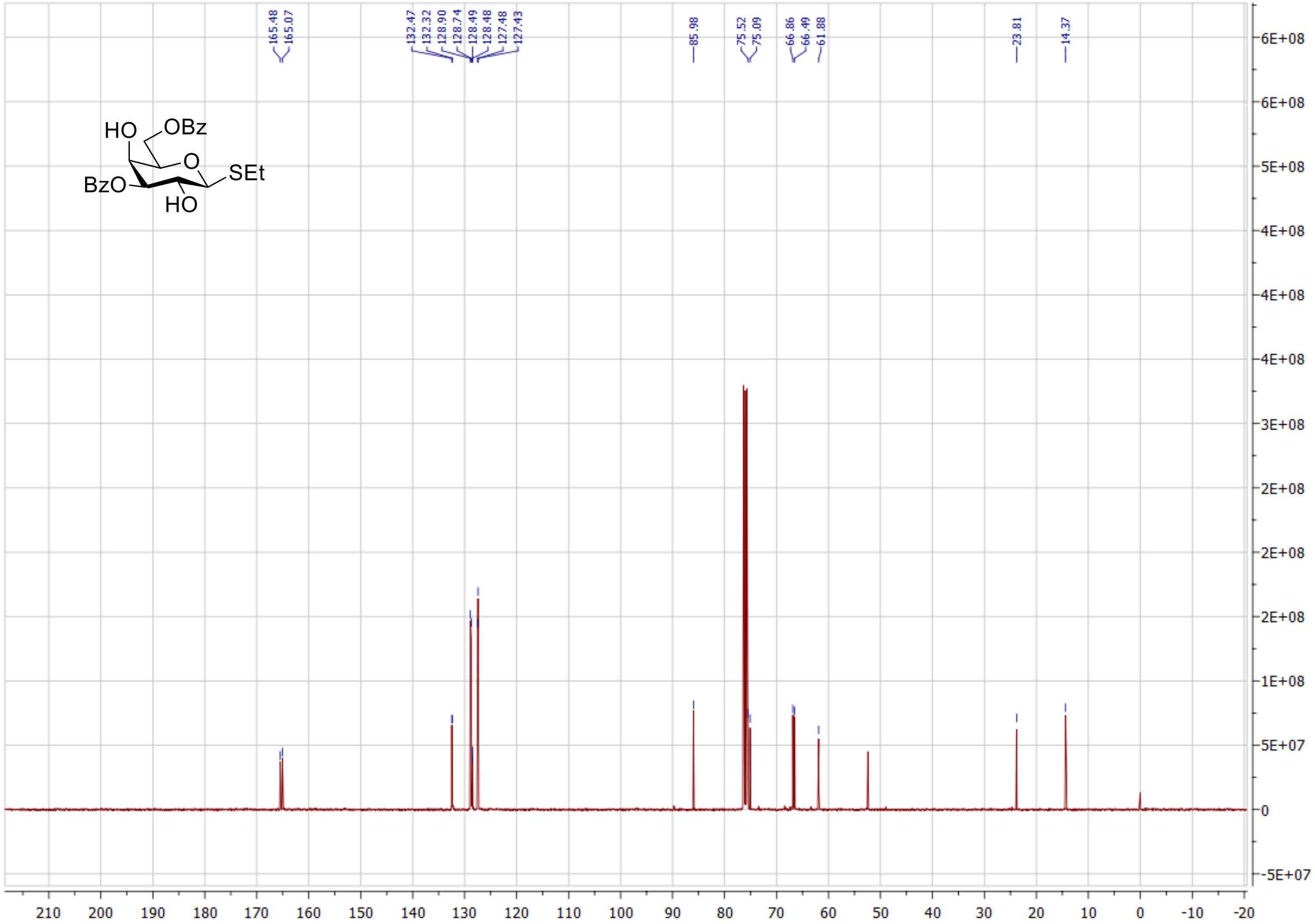

Compound 37

^{1}H NMR (400 MHz, CDCl$_3$): Phenyl 2,3,4,6-tetra-*O*-benzoyl-1-β-D-galactopyranoside 37

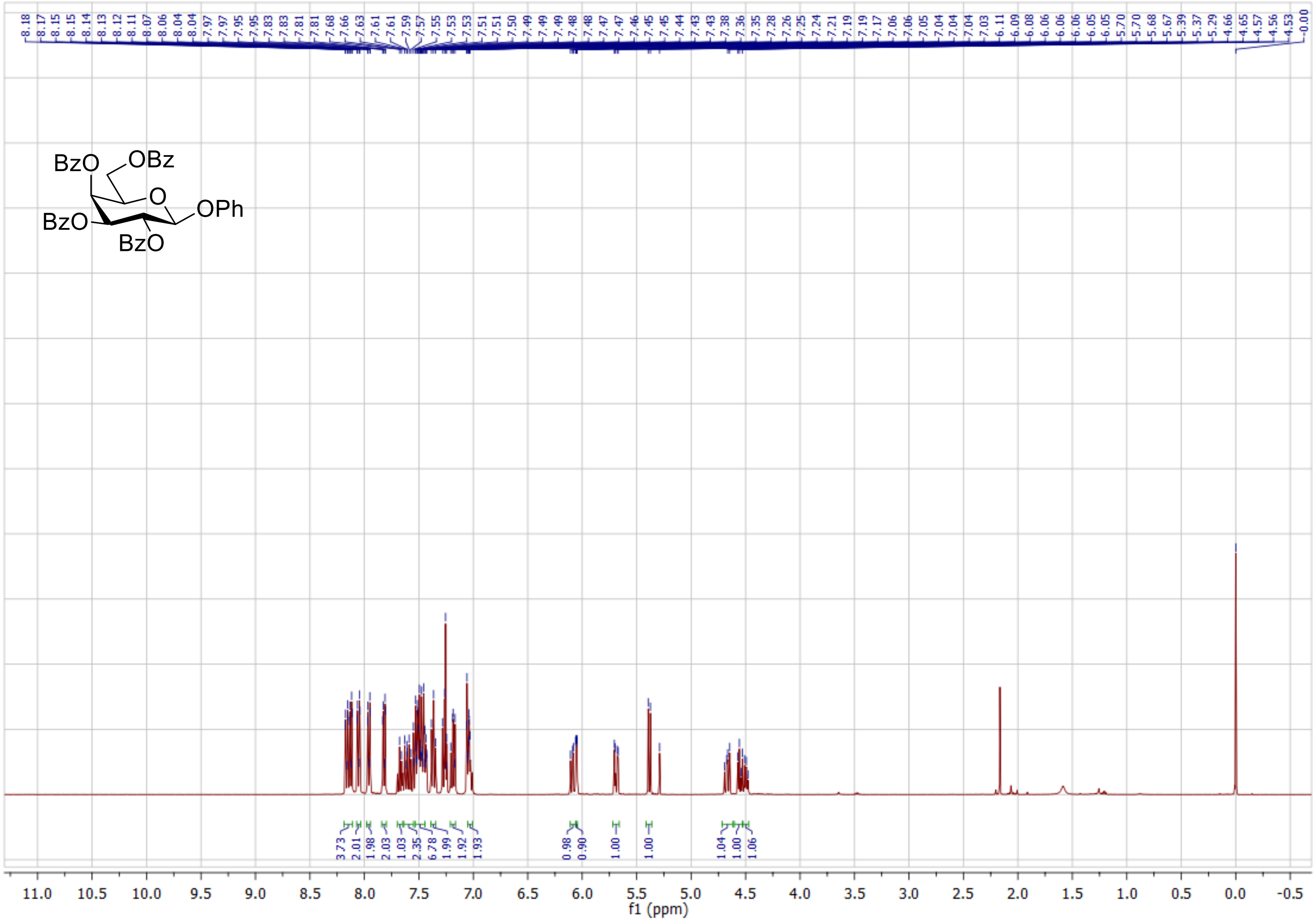

COSY (400 × 400 MHz, CDCl₃): Phenyl 2,3,4,6-tetra-*O*-benzoyl-1-β-D-galactopyranoside 37

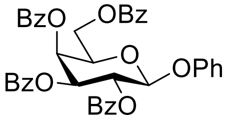

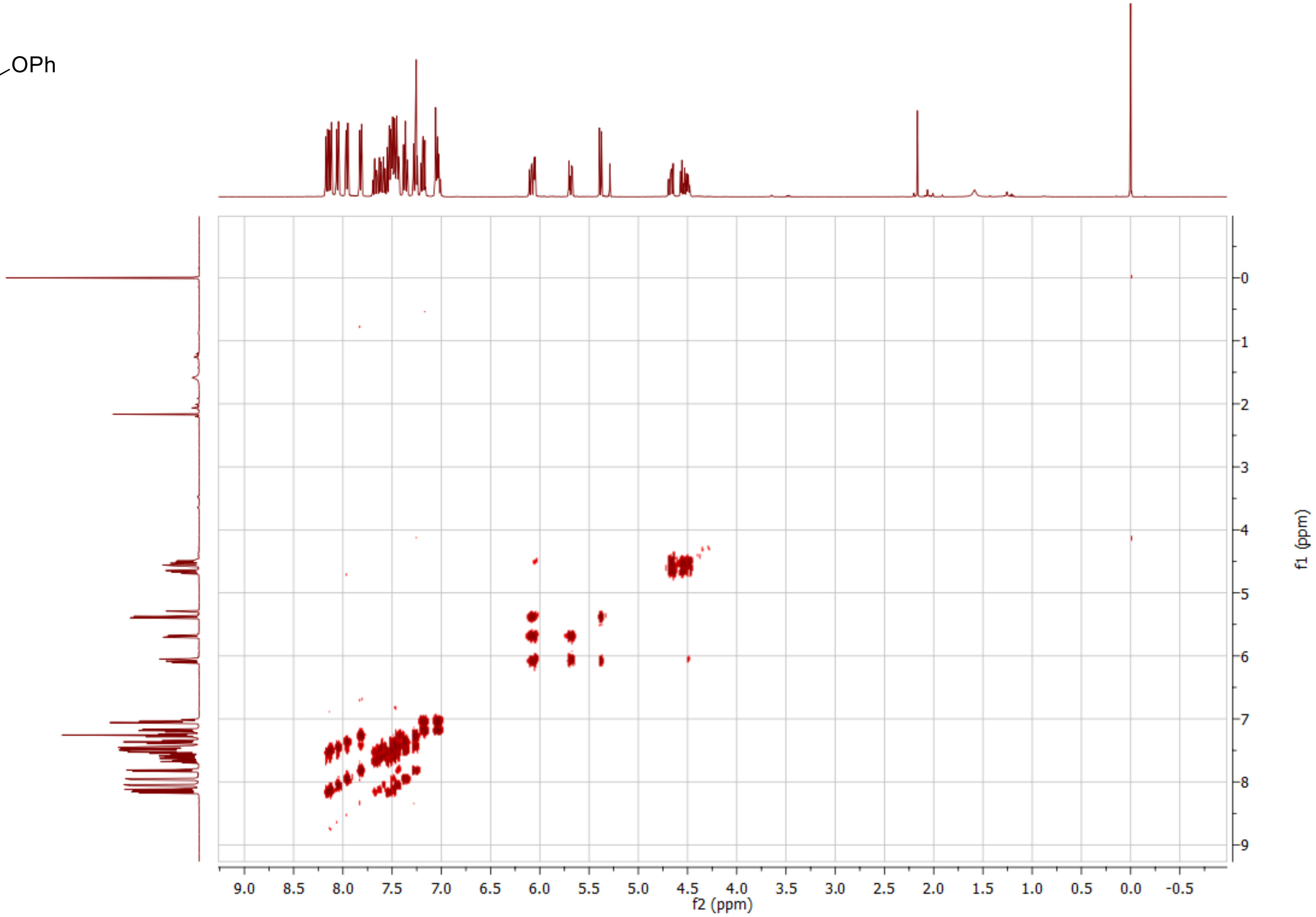

HSQC (400 × 101 MHz, $CDCl_3$): Phenyl 2,3,4,6-tetra-*O*-benzoyl-1-β-D-galactopyranoside 37

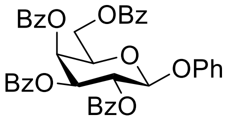

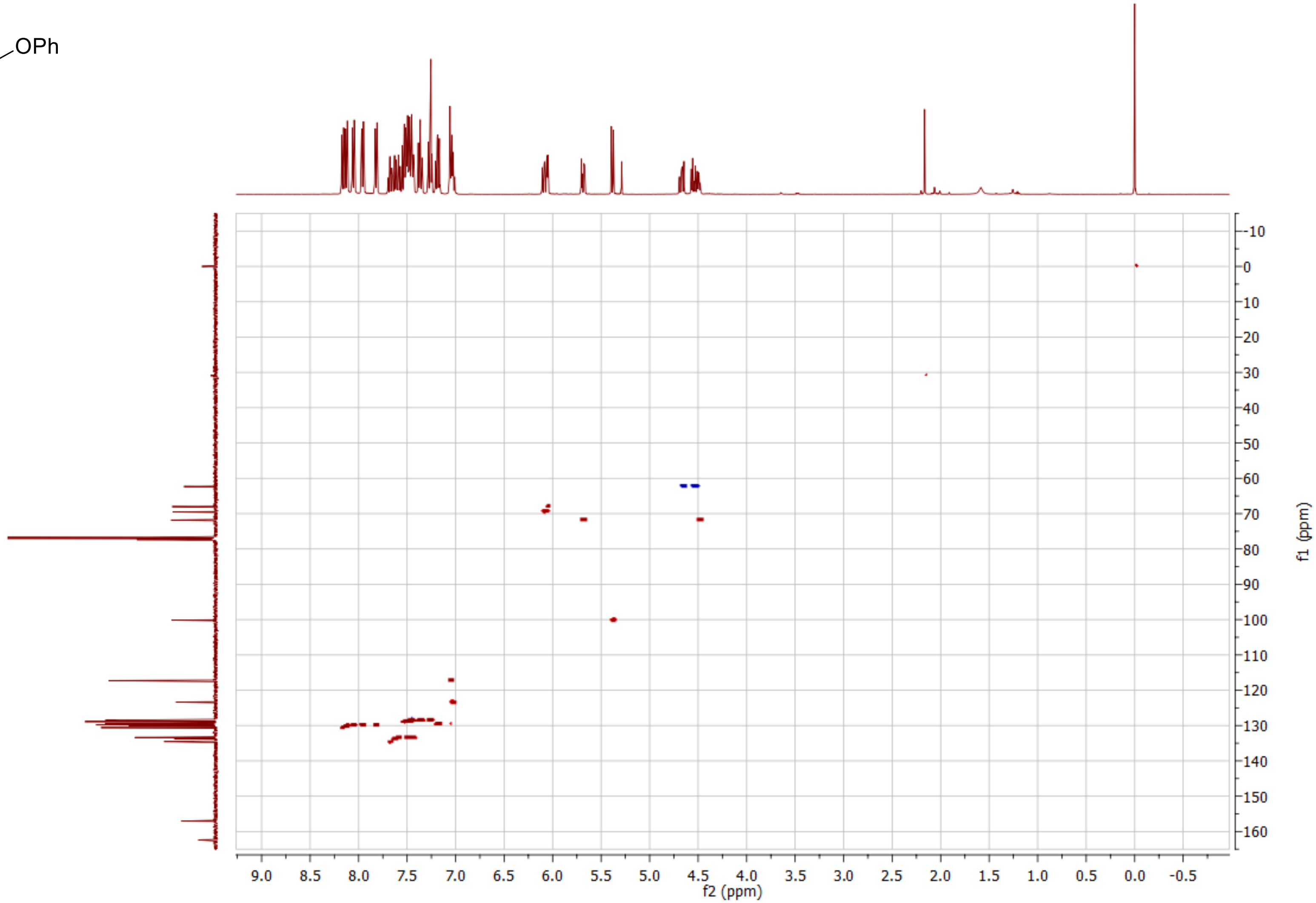

^{13}C{1H} NMR (101 MHz, $CDCl_3$): Phenyl 2,3,4,6-tetra-*O*-benzoyl-1-β-D-galactopyranoside 37

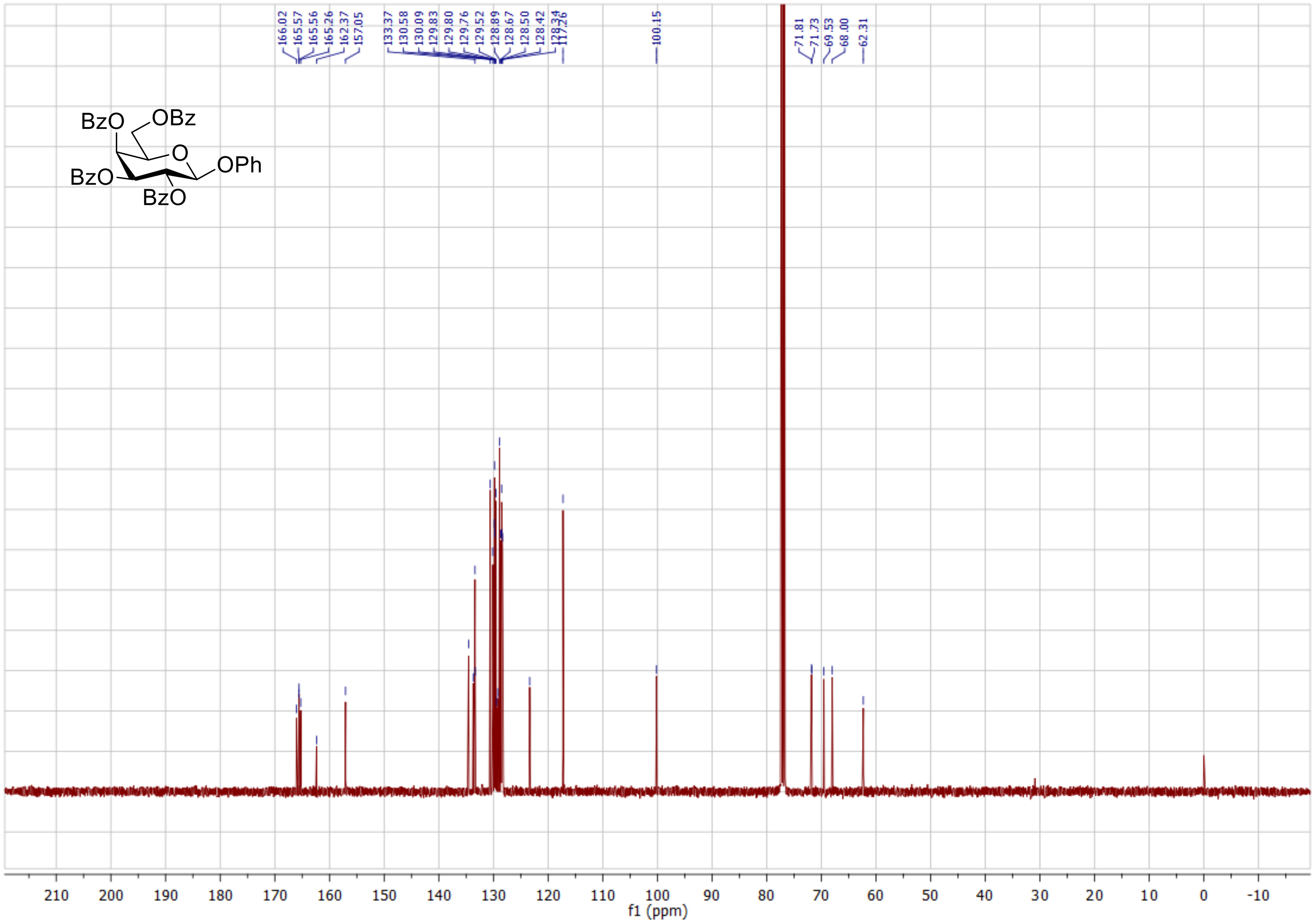

Compound 38/39

^{1}H NMR (400 MHz, $CDCl_3$): Phenyl 3,4,6-tri-*O*-benzoyl-1-β-D-galactopyranoside 38 & Phenyl 2,3,6-tri-*O*-benzoyl-1-β-D-galactopyranoside 39

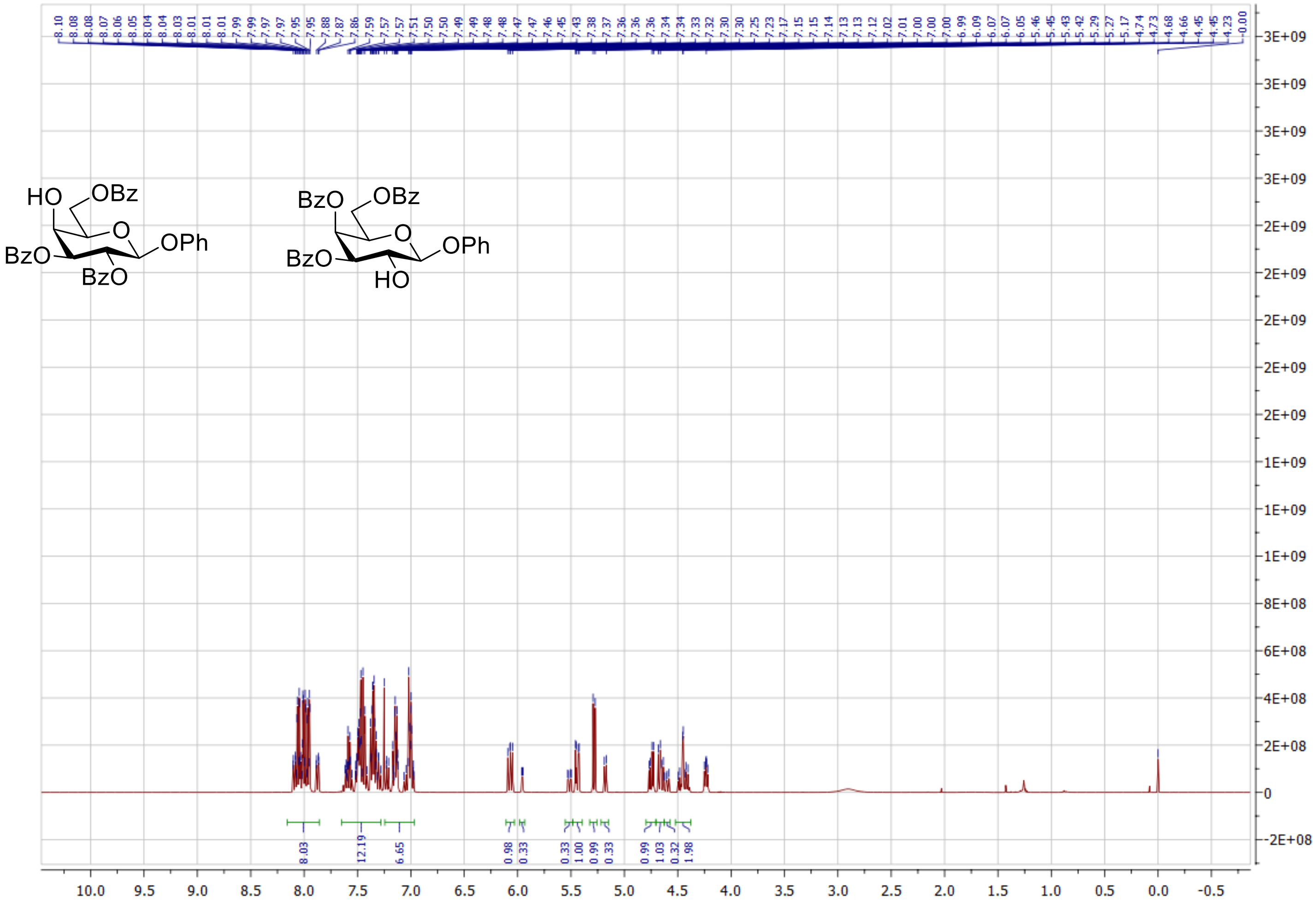

COSY (400 × 400 MHz, CDCl₃): Phenyl 3,4,6-tri-*O*-benzoyl-1-β-ᴅ-galactopyranoside 38 & Phenyl 2,3,6-tri-*O*-benzoyl-1-β-ᴅ-galactopyranoside 39

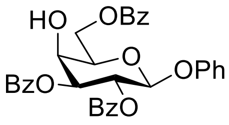

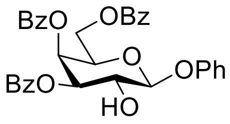

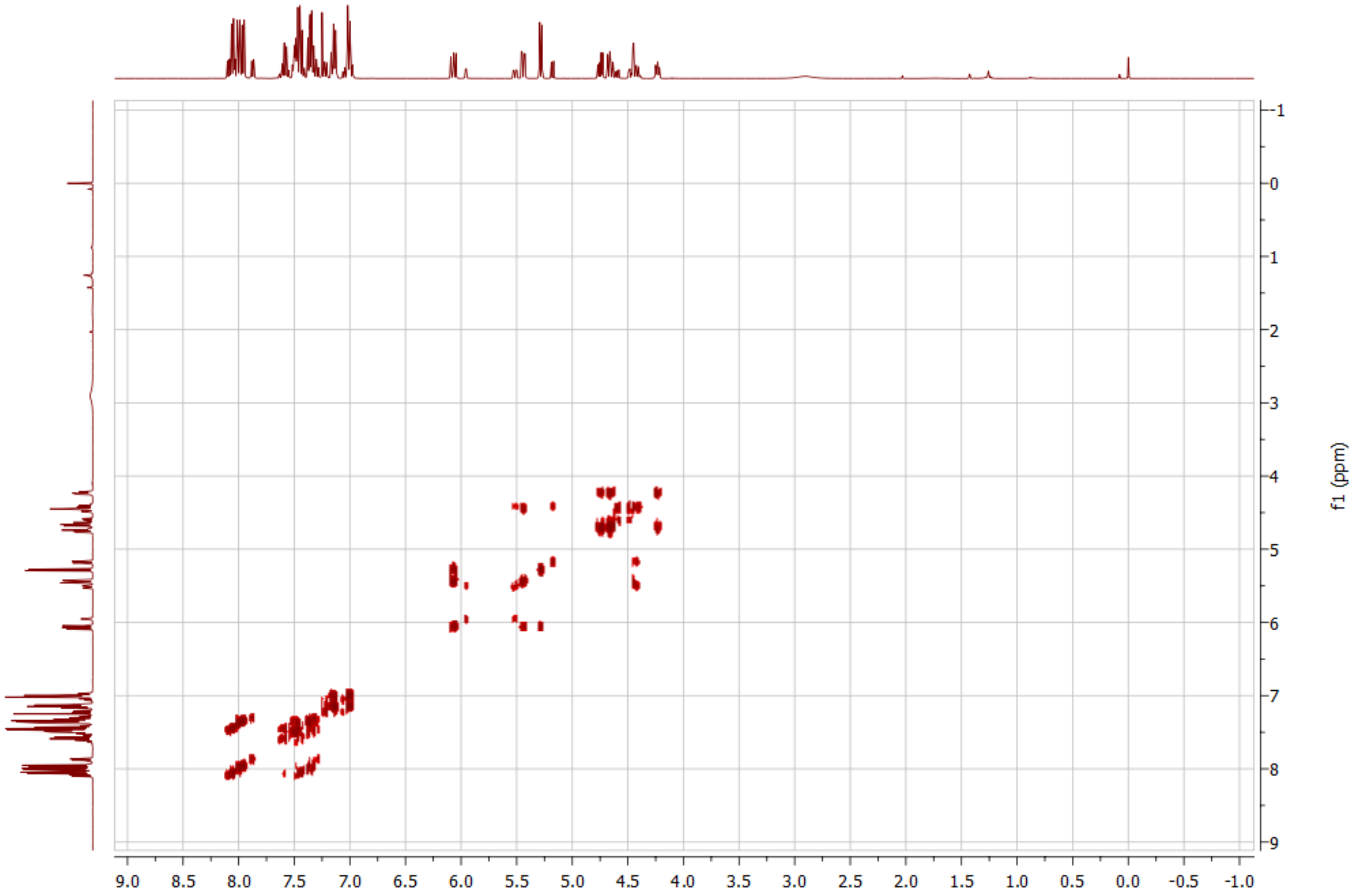

HSQC (400 × 101 MHz, $CDCl_3$): Phenyl 3,4,6-tri-*O*-benzoyl-1-β-D-galactopyranoside 38 & Phenyl 2,3,6-tri-*O*-benzoyl-1-β-D-galactopyranoside 39

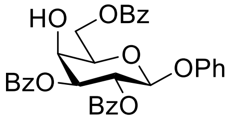

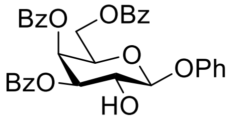

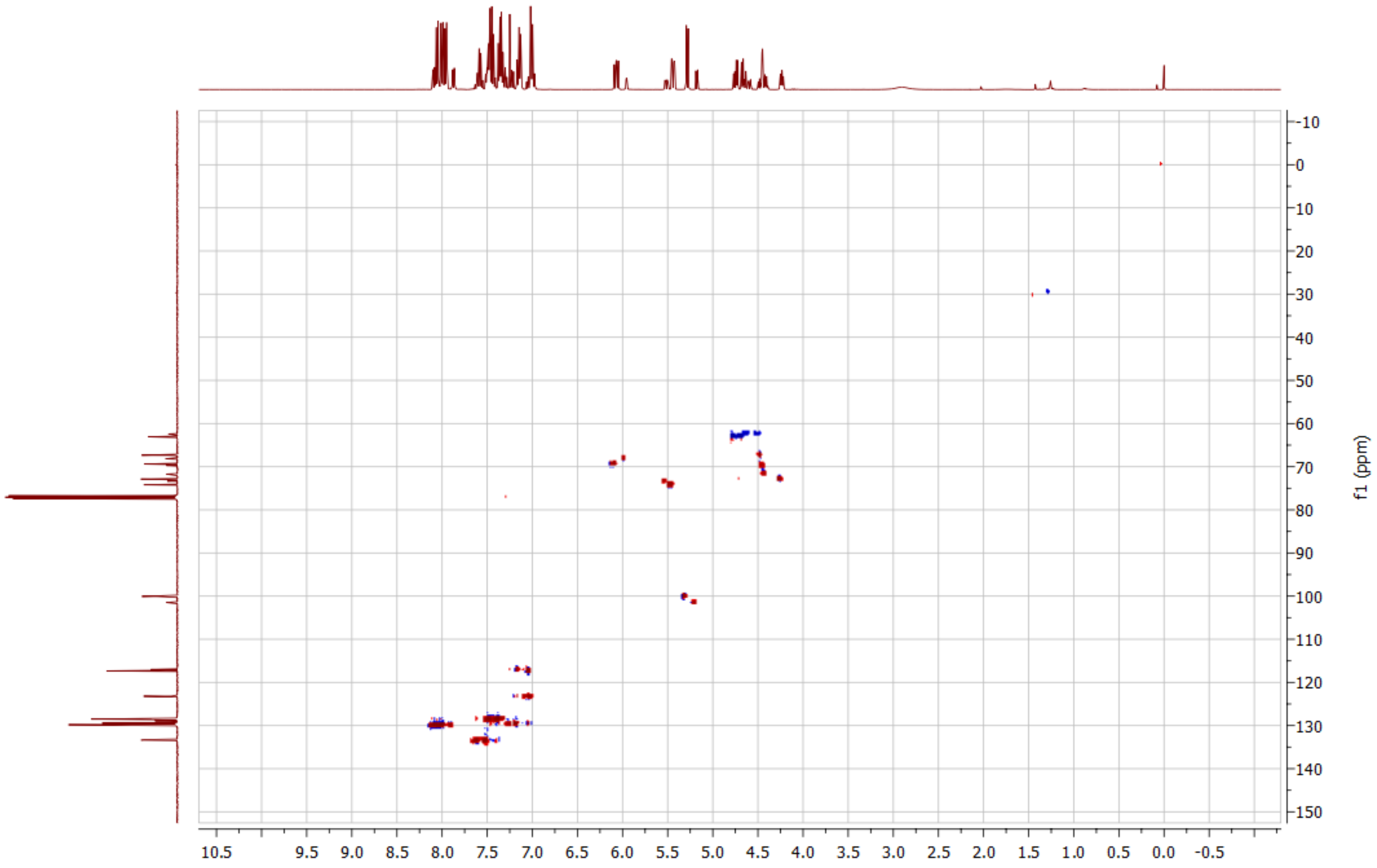

HMBC (400 × 101 MHz, CDCl₃): Phenyl 3,4,6-tri-*O*-benzoyl-1-β-D-galactopyranoside 38 & Phenyl 2,3,6-tri-*O*-benzoyl-1-β-D-galactopyranoside 39

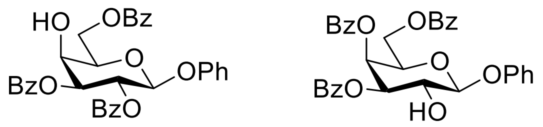

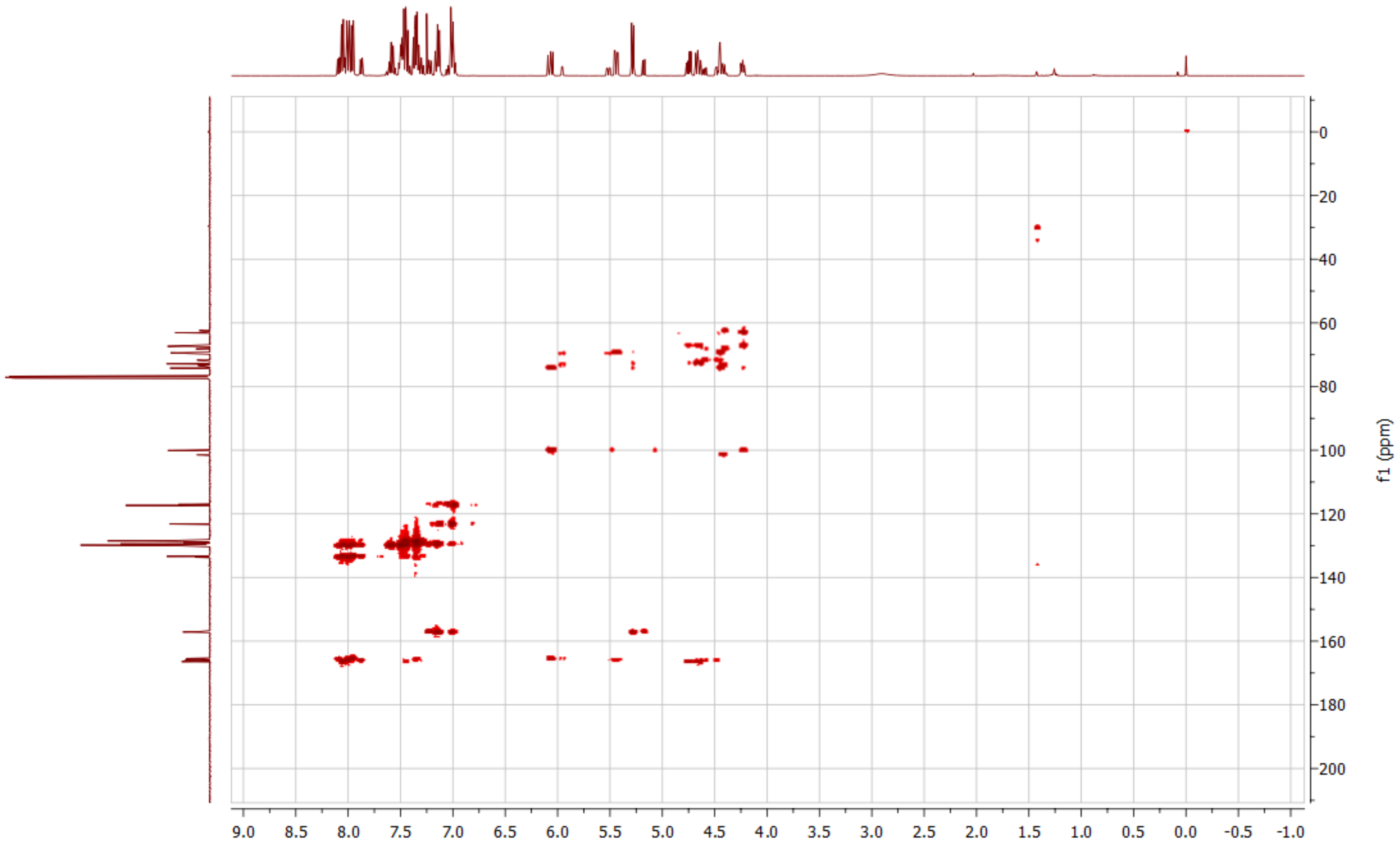

$^{13}C\{1H\}$ NMR (101 MHz, $CDCl_3$): Phenyl 3,4,6-tri-*O*-benzoyl-1-β-D-galactopyranoside 38 & Phenyl 2,3,6-tri-*O*-benzoyl-1-β-D-galactopyranoside 39

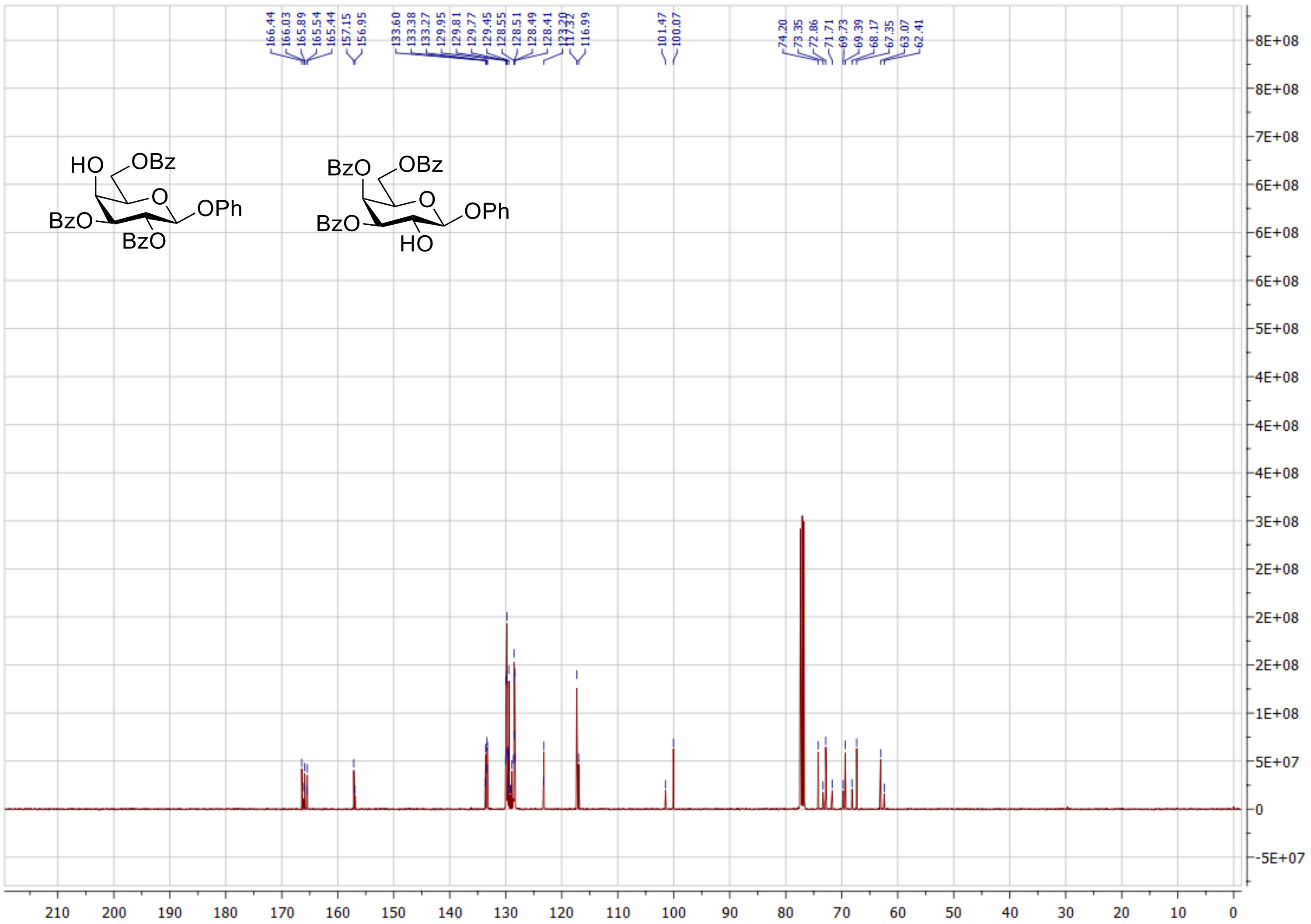

Compound 41

^{1}H NMR (400 MHz, CDCl$_3$): Ethyl 2,3,4,6-tetra-*O*-benzoyl-β-D-galactopyranoside 41

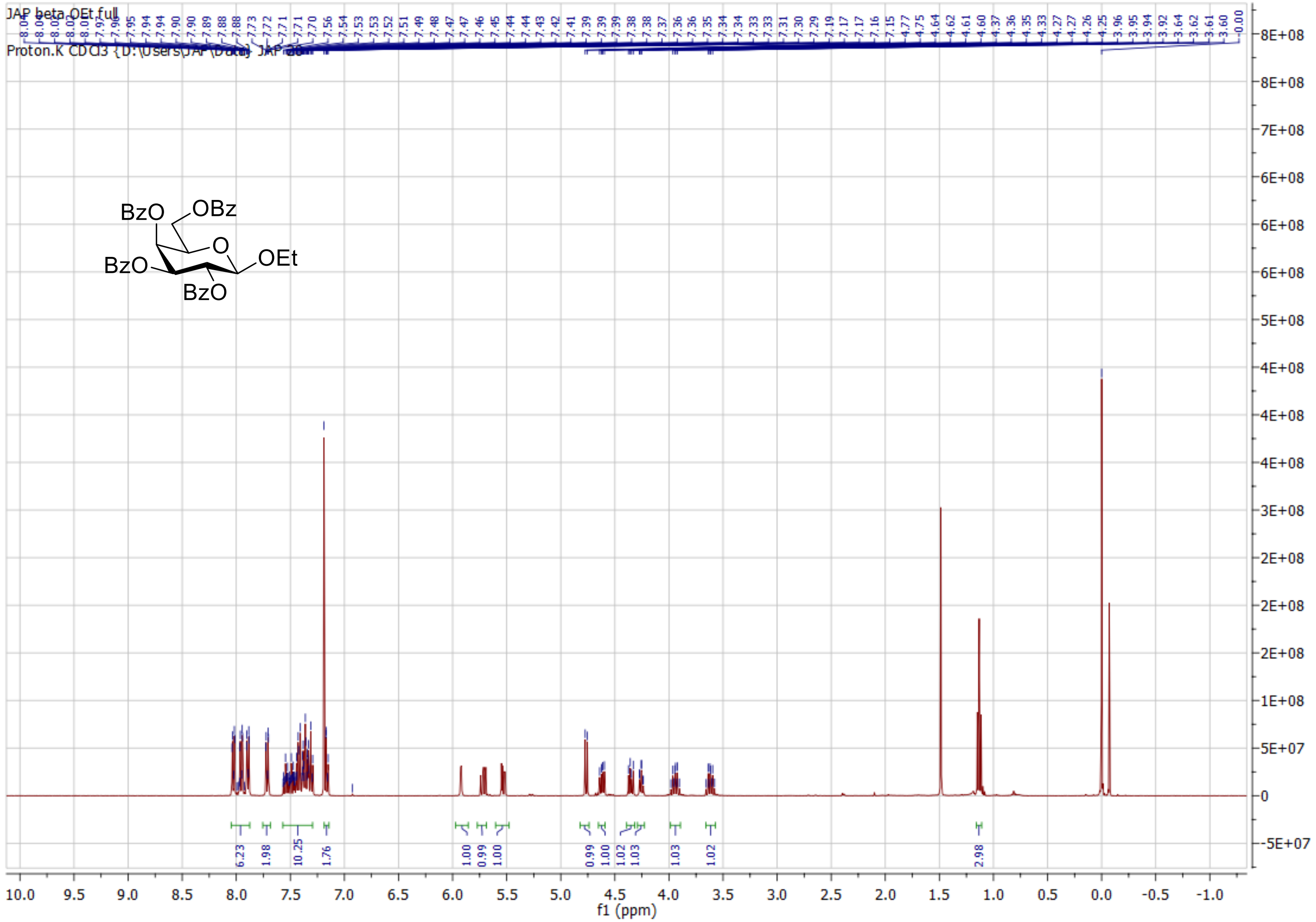

^{13}C NMR{1H} (400 MHz, $CDCl_3$): Ethyl 2,3,4,6-tetra-*O*-benzoyl-β-D-galactopyranoside 41

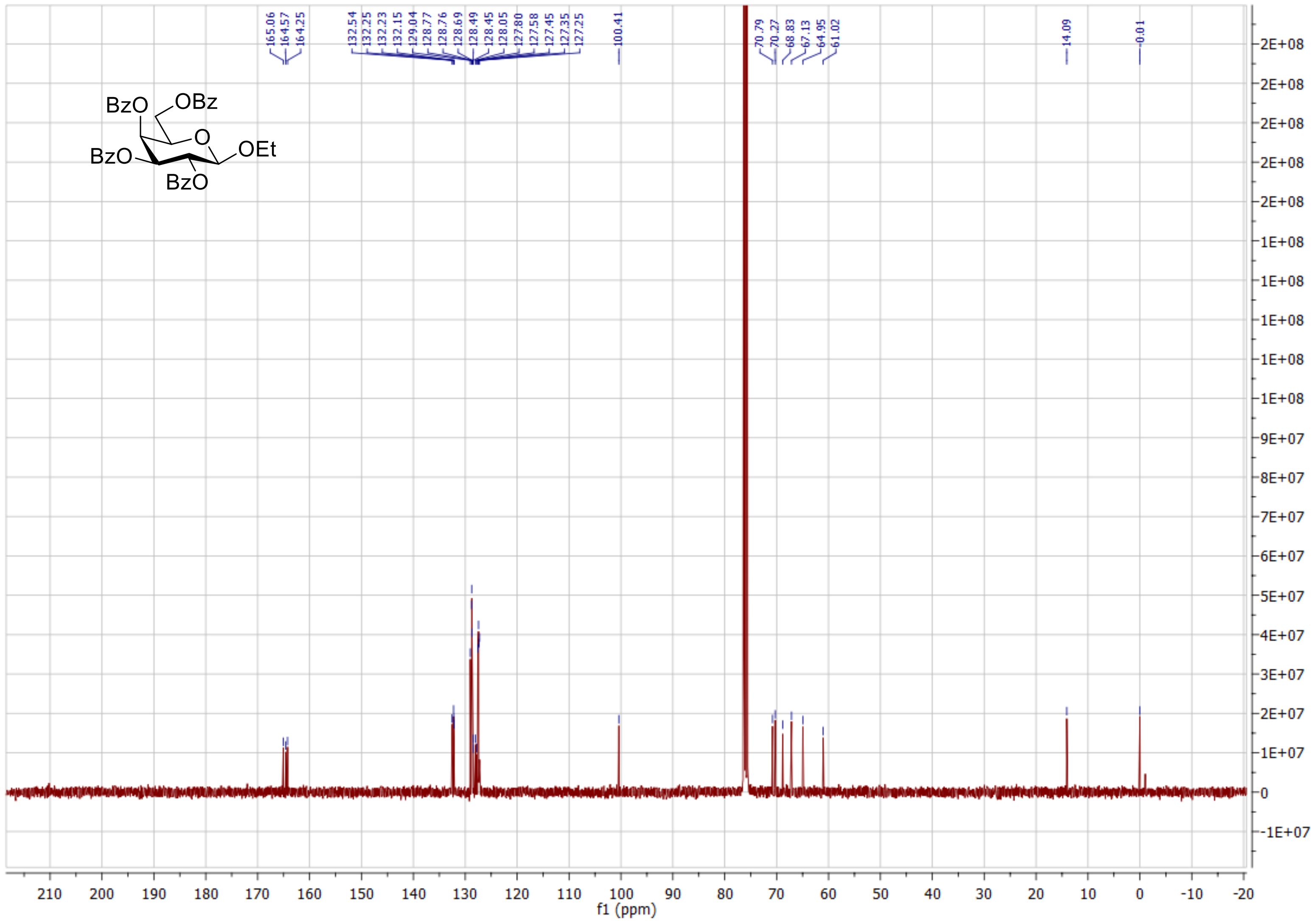

Compound 42

^{1}H NMR (400 MHz, CDCl$_3$): Ethyl 2,3,6-tri-*O*-benzoyl-β-ᴅ-galactopyranoside 42

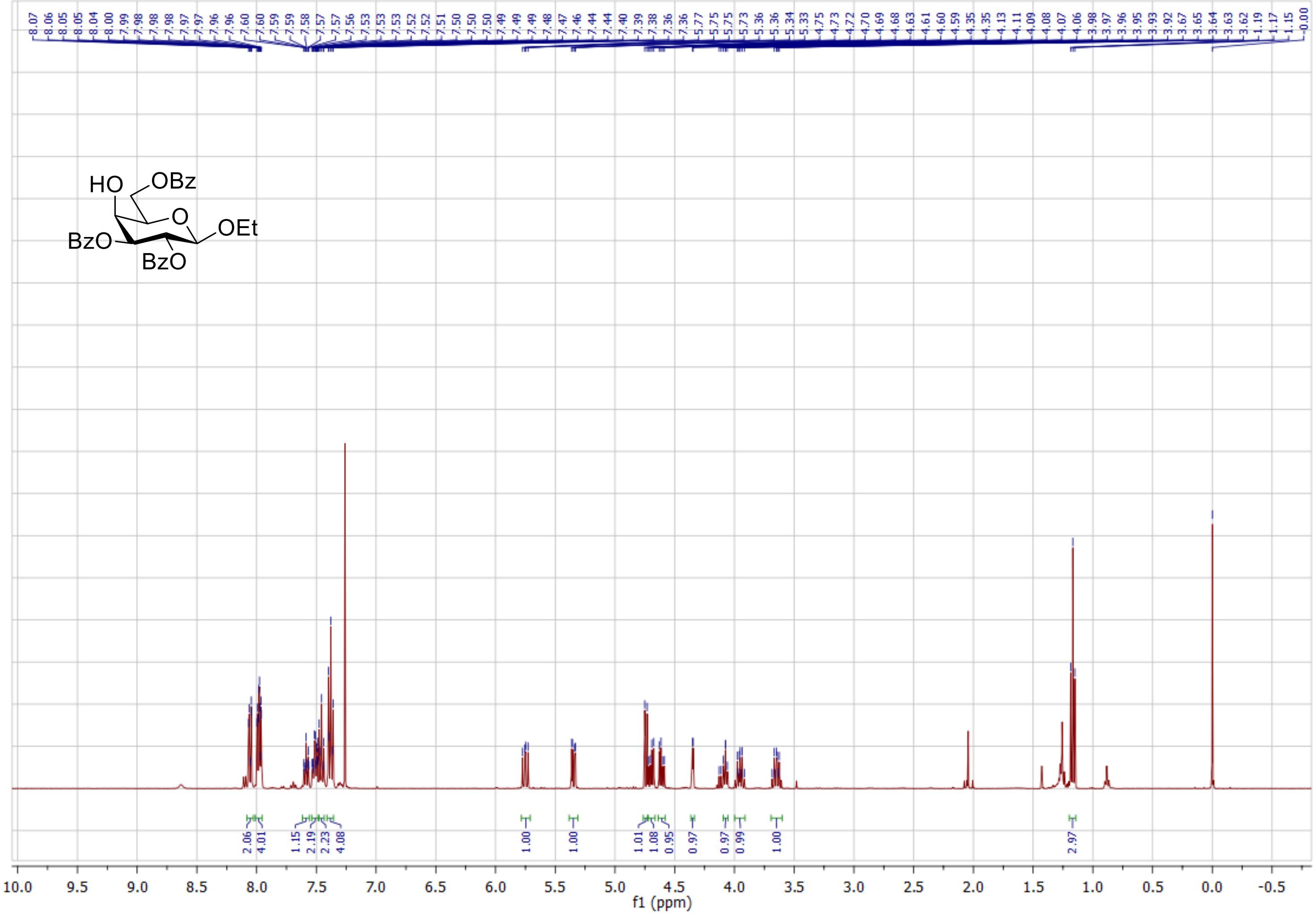

COSY (400 × 400 MHz, $CDCl_3$): Ethyl 2,3,6-tri-*O*-benzoyl-β-D-galactopyranoside 42

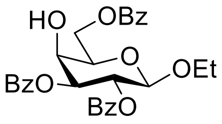

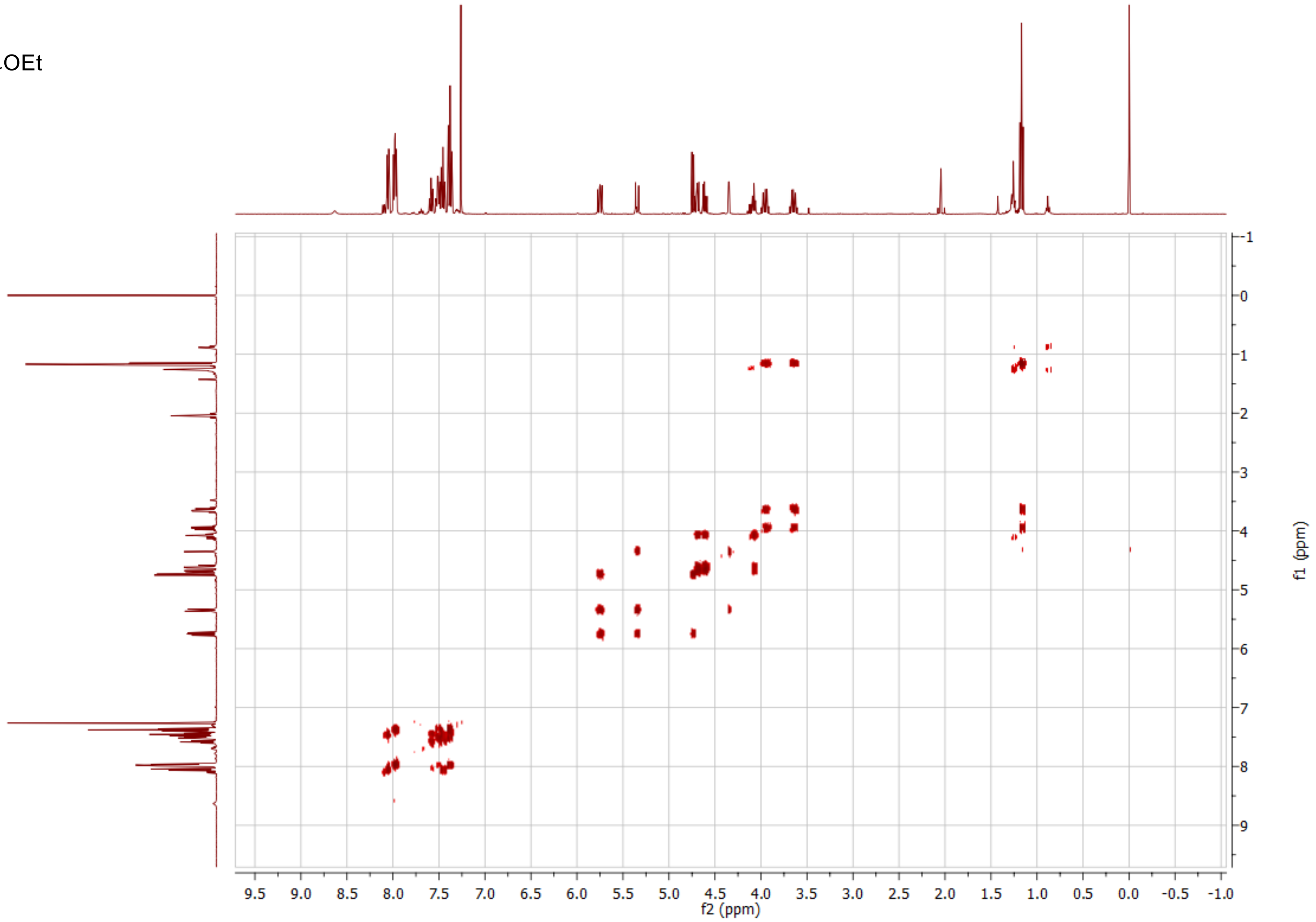

HSQC (400 × 101 MHz, CDCl₃): Ethyl 2,3,6-tri-*O*-benzoyl-β-D-galactopyranoside 42

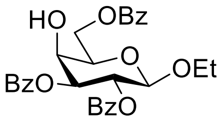

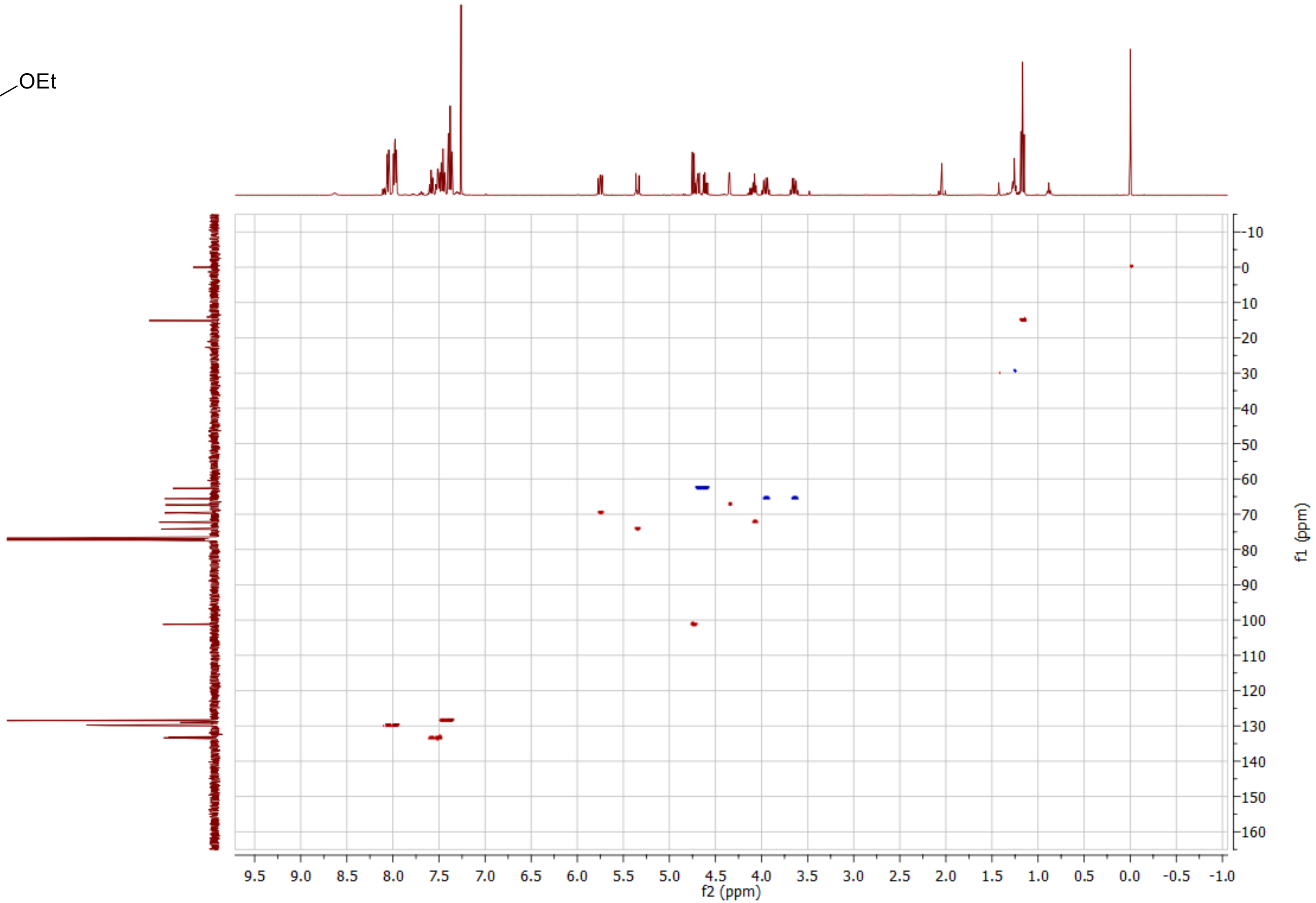

HMBC (400 × 101 MHz, $CDCl_3$): Ethyl 2,3,6-tri-*O*-benzoyl-β-D-galactopyranoside 42

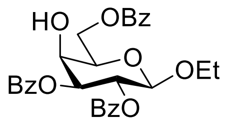

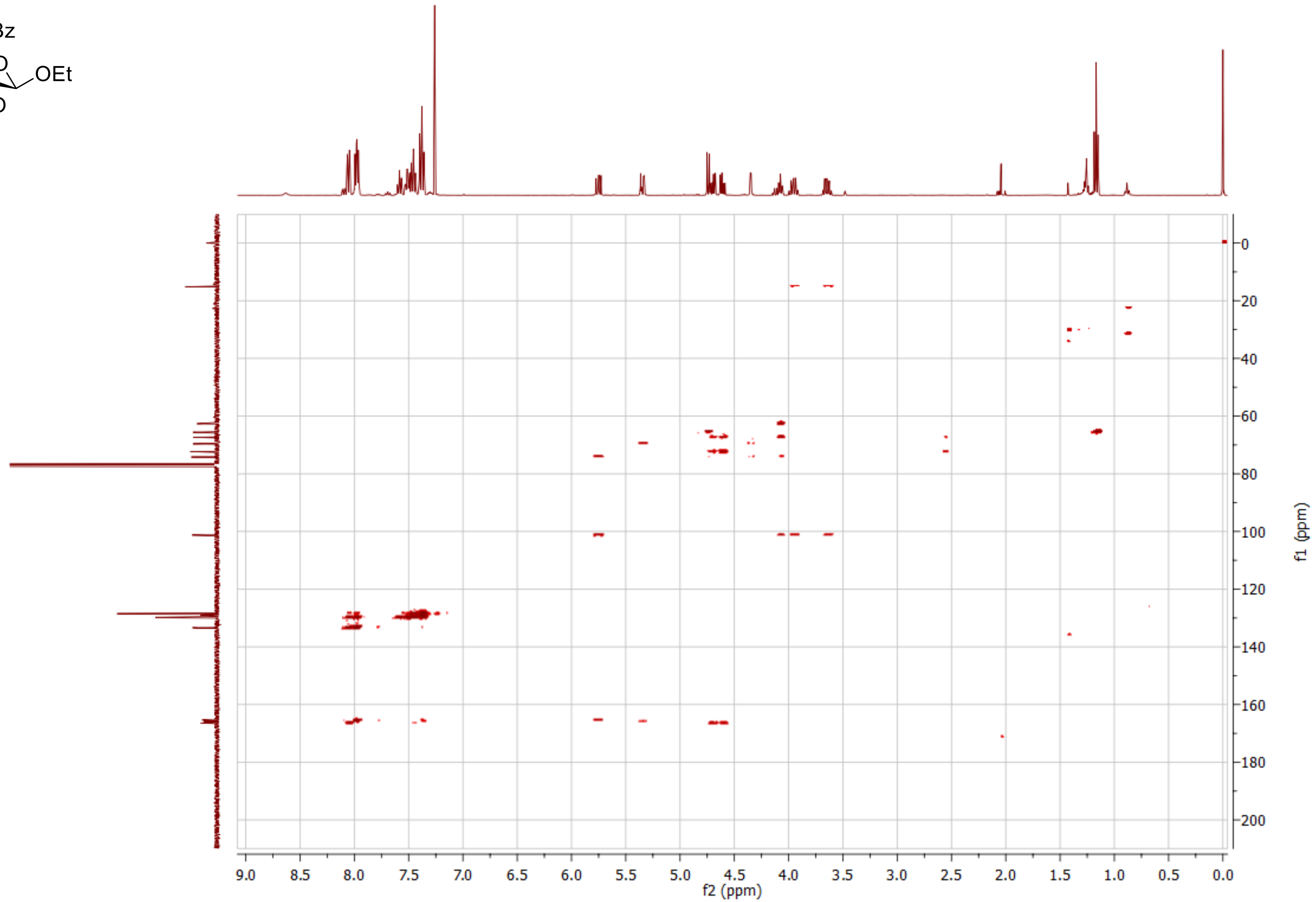

$^{13}C\{1H\}$ NMR (101 MHz, $CDCl_3$): Ethyl 2,3,6-tri-*O*-benzoyl-β-D-galactopyranoside 42

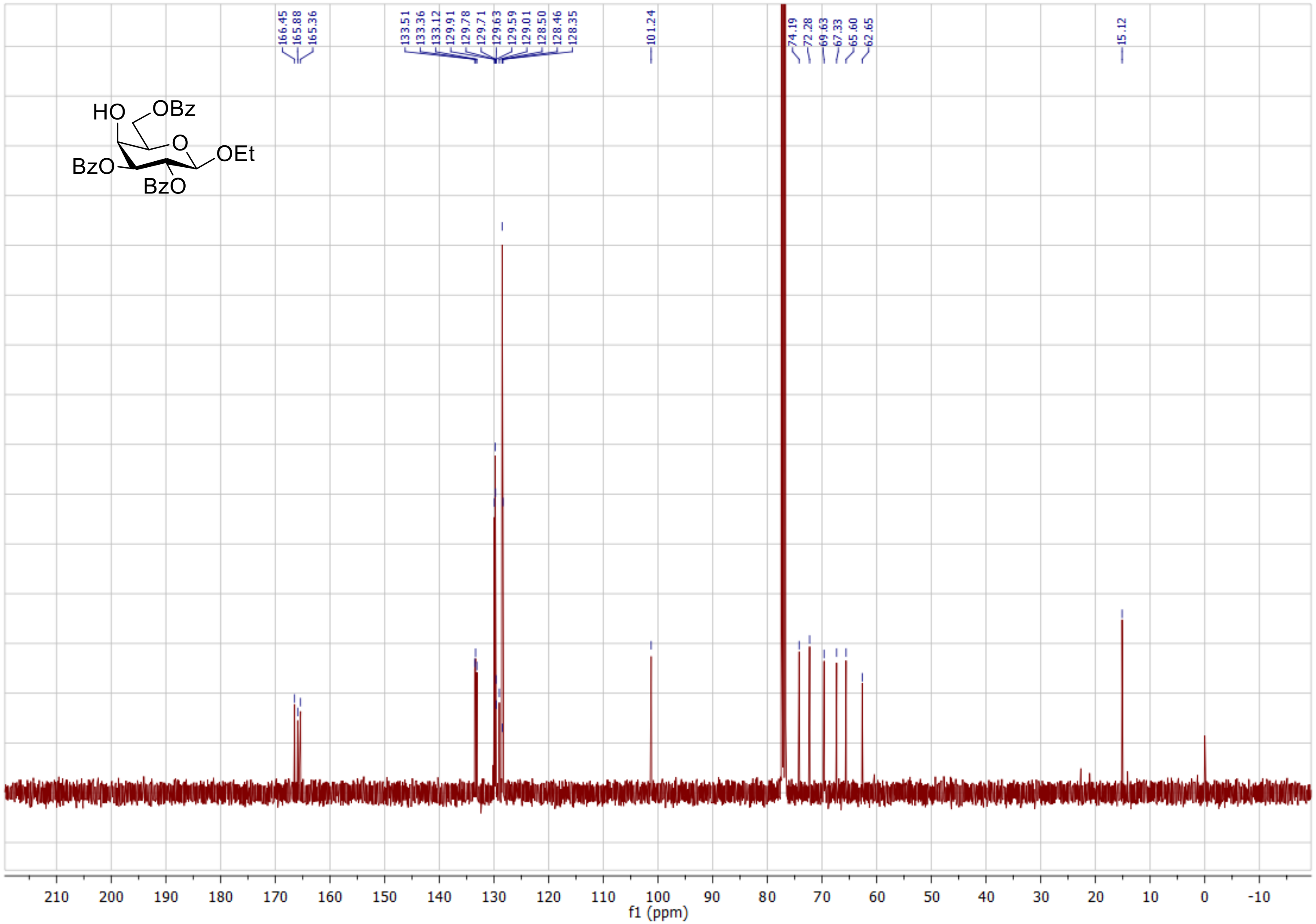

Compound 44

^{1}H NMR (400 MHz, $CDCl_3$): Cyclohexyl 3,6-di-*O*-benzoyl-1-thio-β-D-galactopyranoside 44

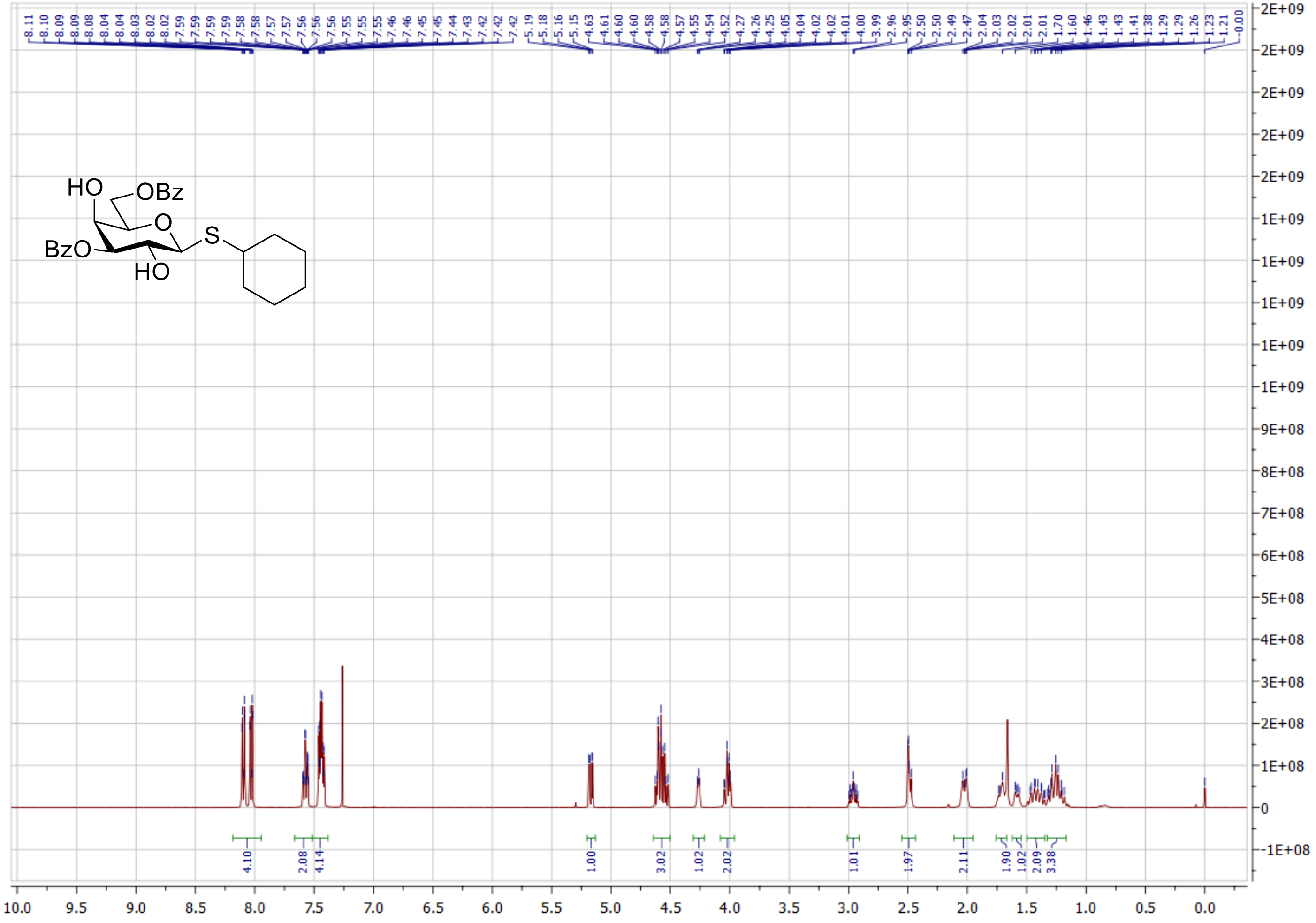

COSY (400 × 400 MHz, $CDCl_3$): Cyclohexyl 3,6-di-*O*-benzoyl-1-thio-β-D-galactopyranoside 44

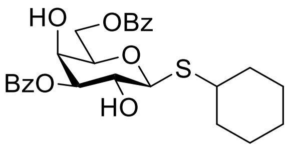

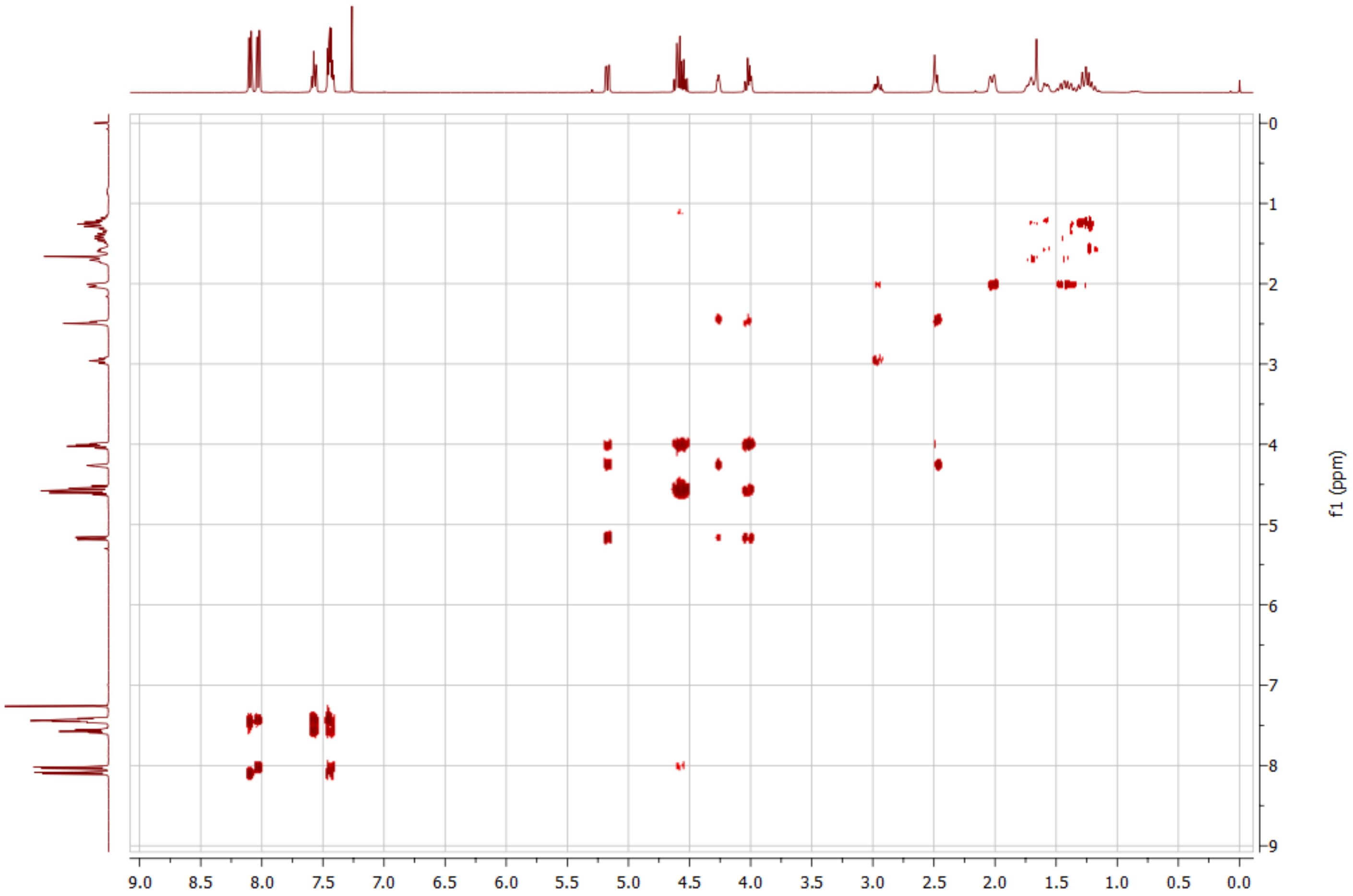

HSQC (400 × 101 MHz, $CDCl_3$): Cyclohexyl 3,6-di-*O*-benzoyl-1-thio-β-D-galactopyranoside 44

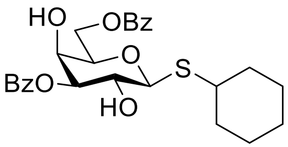

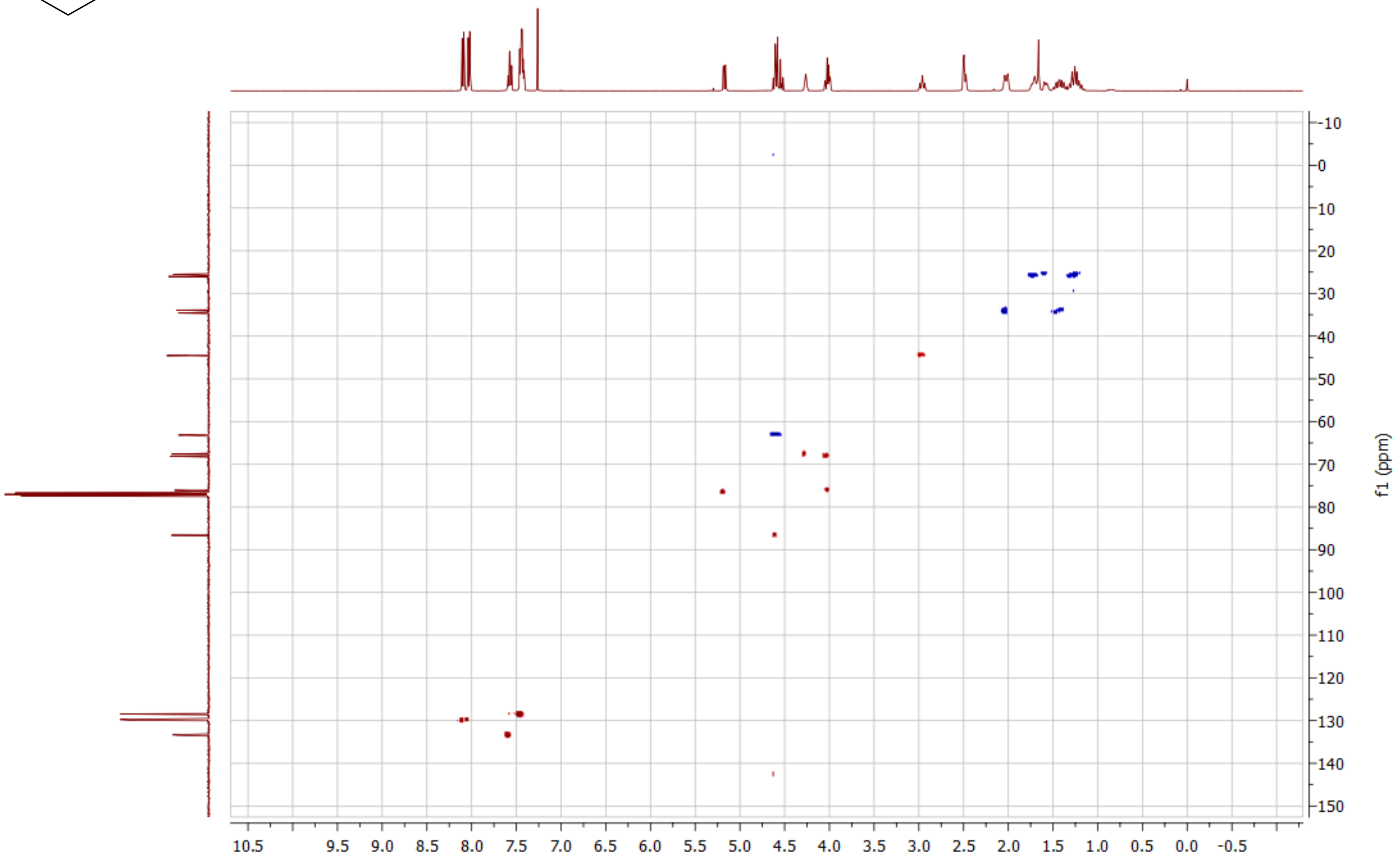

HMBC (400 × 101 MHz, CDCl₃): Cyclohexyl 3,6-di-*O*-benzoyl-1-thio-β-D-galactopyranoside 44

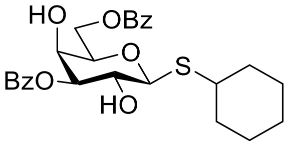

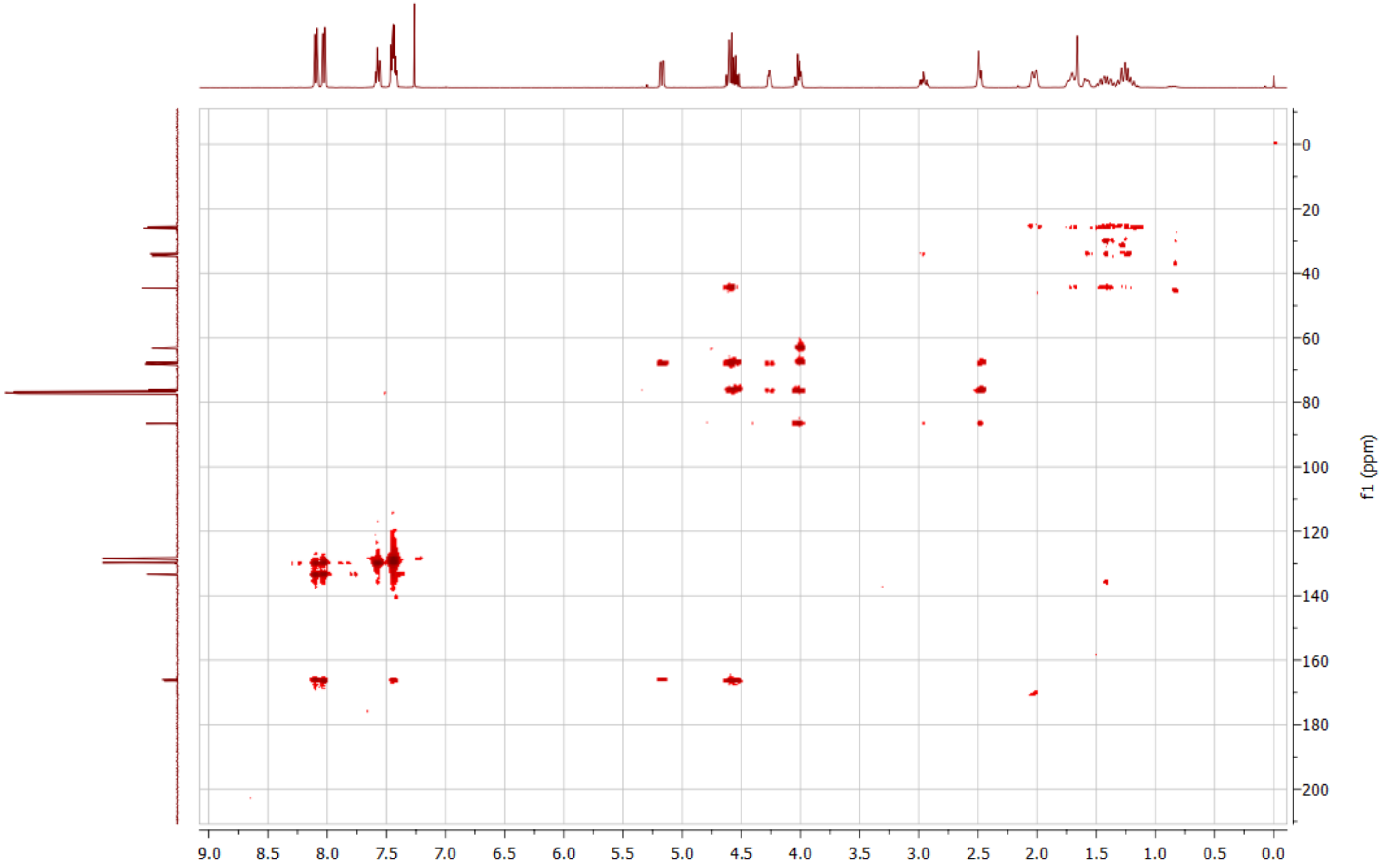

^{13}C{1H} NMR (101 MHz, $CDCl_3$): Cyclohexyl 3,6-di-*O*-benzoyl-1-thio-β-D-galactopyranoside 44

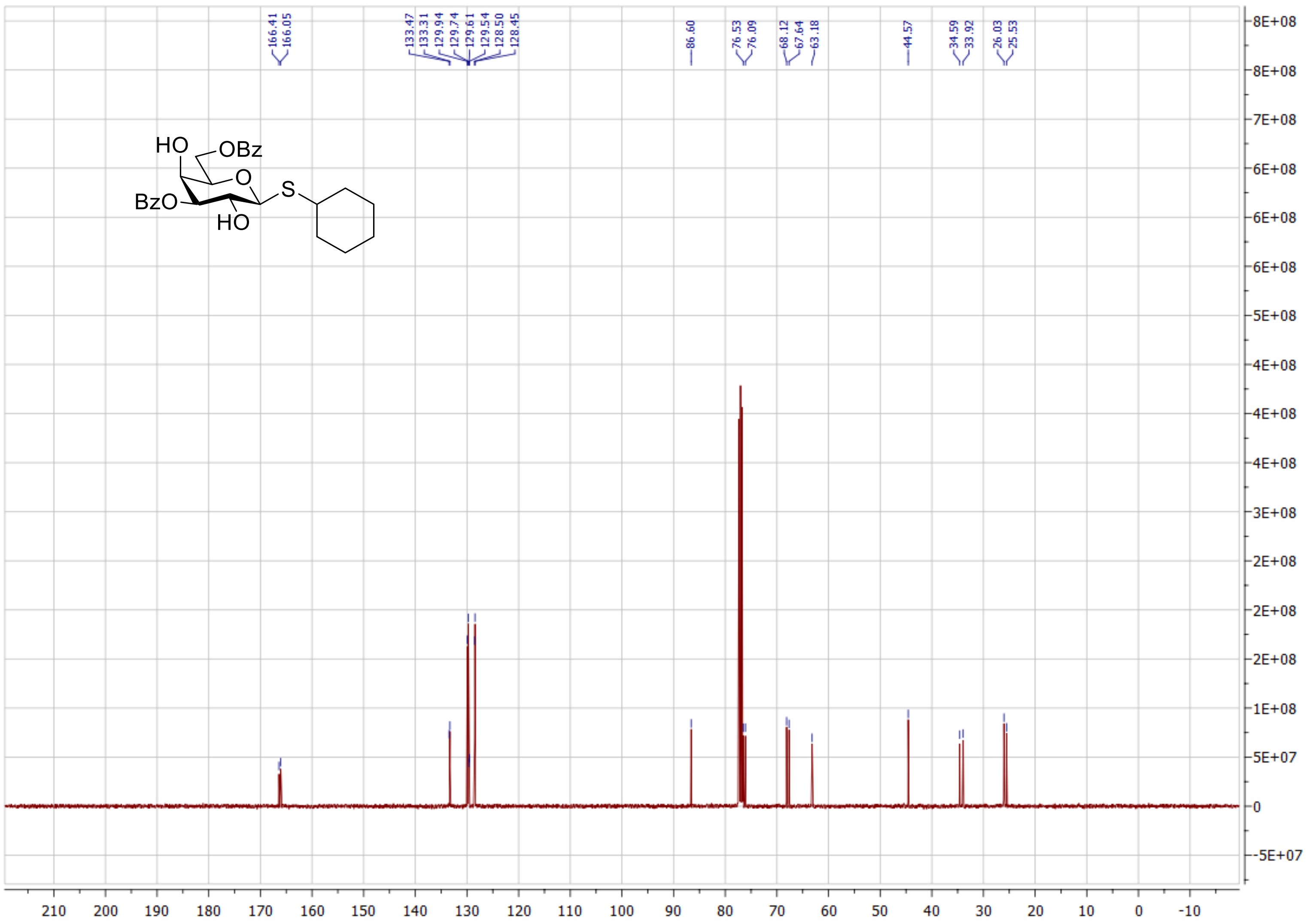

Compound 46

^{1}H NMR (400 MHz, CDCl₃): Trifluoroethyl 2,3,4,6-tetra-*O*-benzoyl-1-thio-β-D-galactopyranoside 46

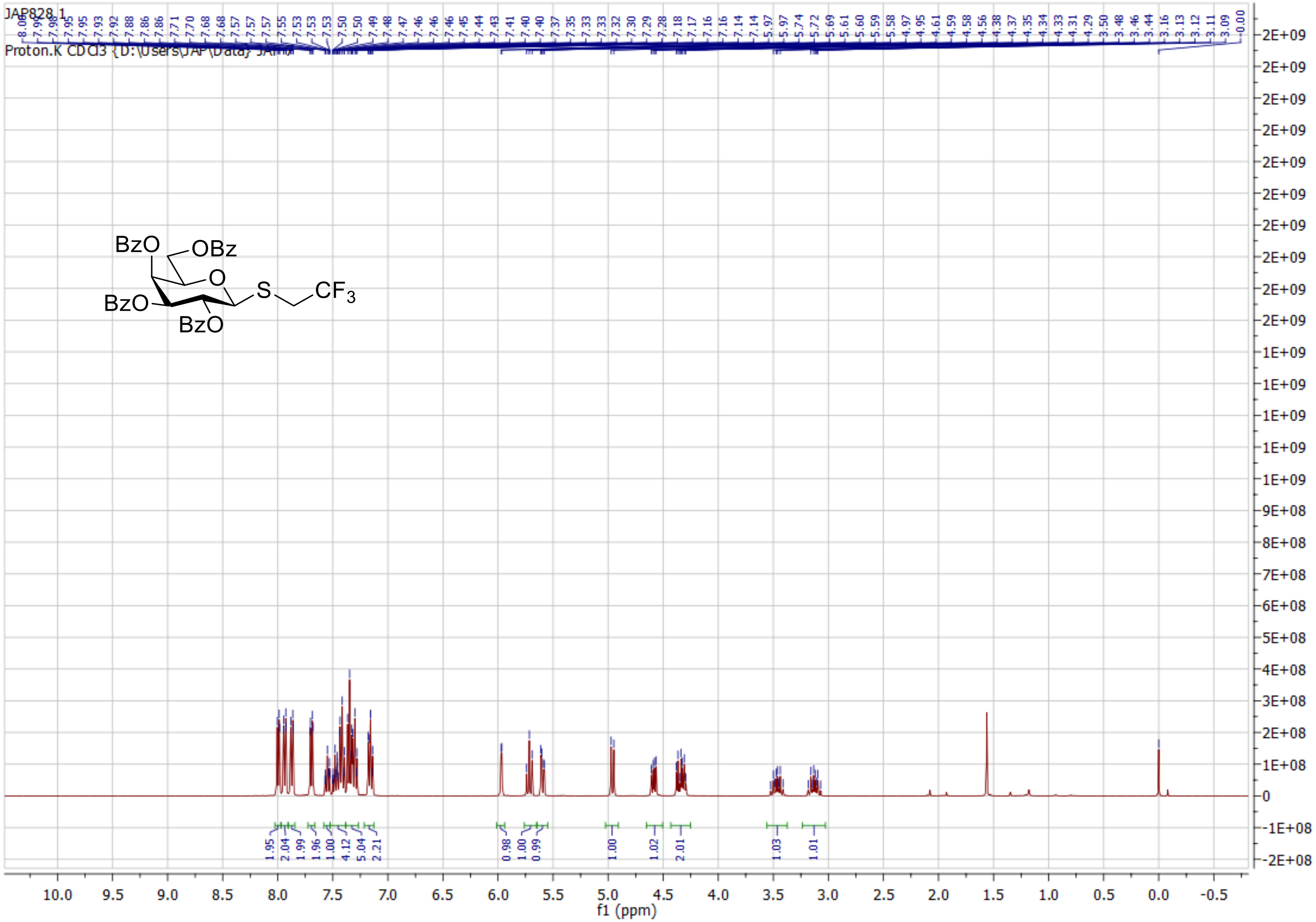

COSY (400 × 400 MHz, $CDCl_3$): Trifluoroethyl 2,3,4,6-tetra-*O*-benzoyl-1-thio-β-D-galactopyranoside 46

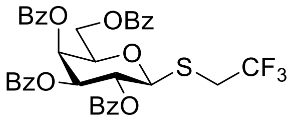

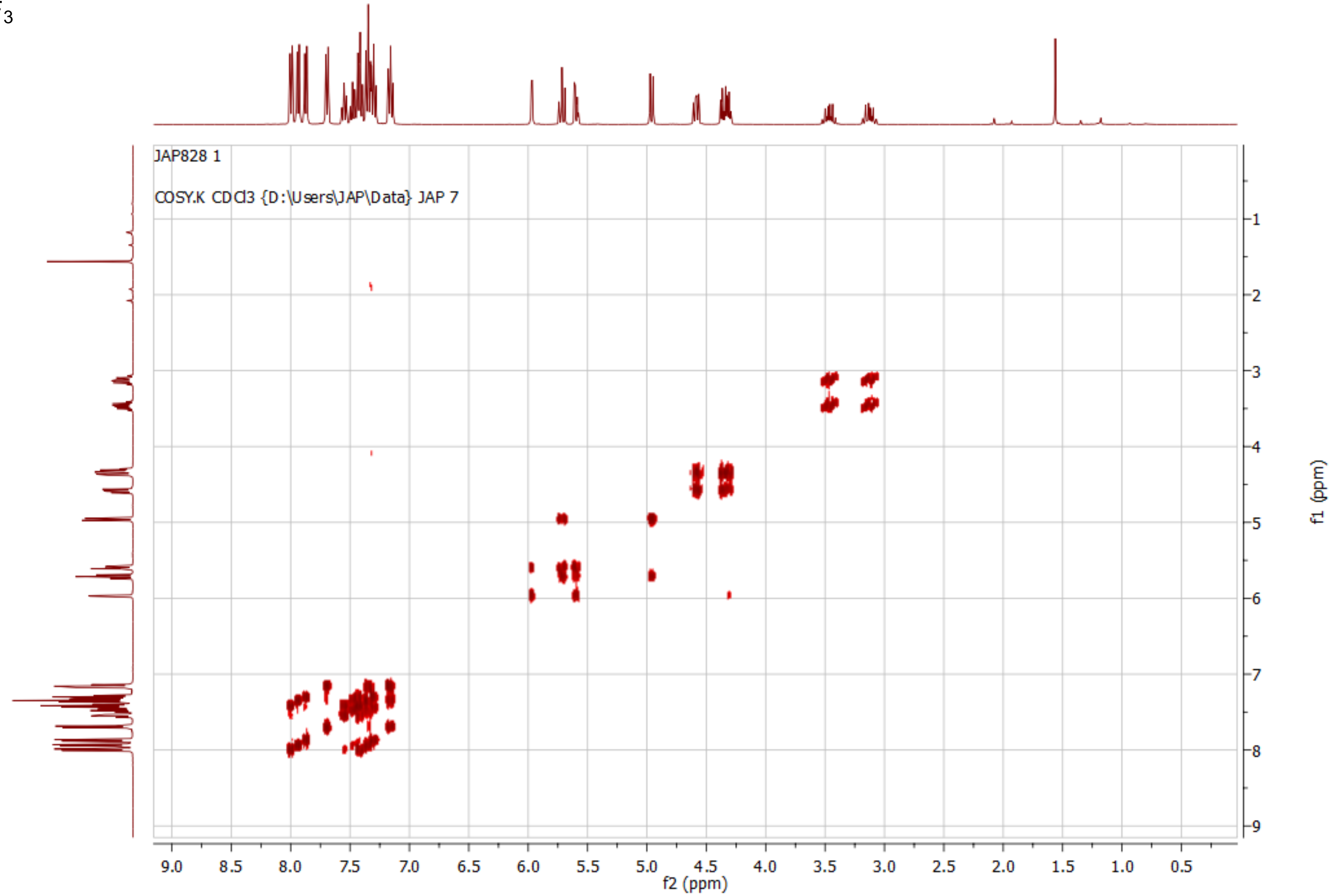

HSQC (400 × 101 MHz, $CDCl_3$): Trifluoroethyl 2,4,3,6-tetra-*O*-benzoyl-1-thio-β-D-galactopyranoside 46

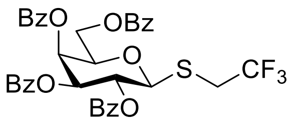

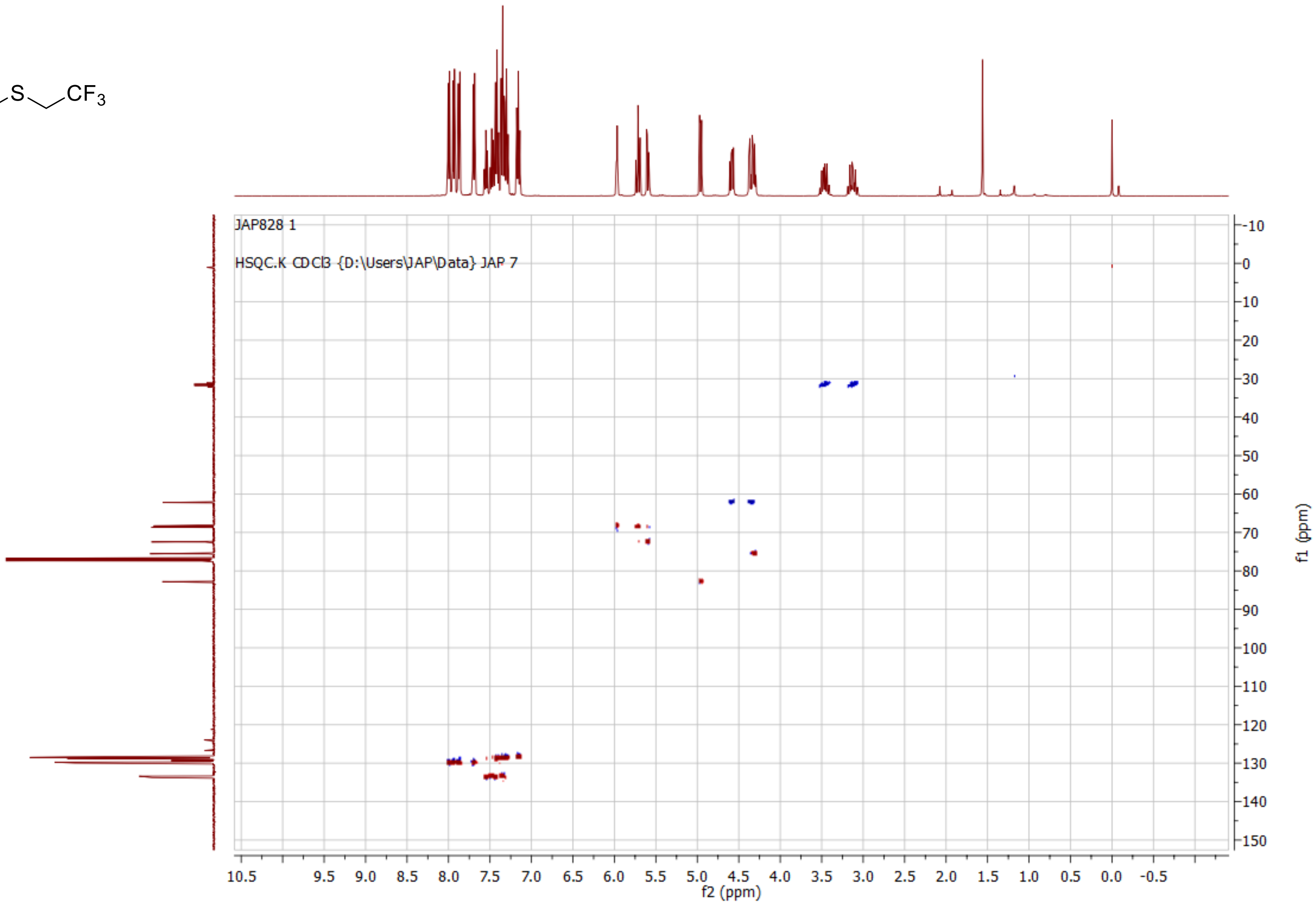

$^{13}C\{1H\}$ NMR (101 MHz, $CDCl_3$): Trifluoroethyl 2,4,3,6-tetra-*O*-benzoyl-1-thio-β-D-galactopyranoside 46

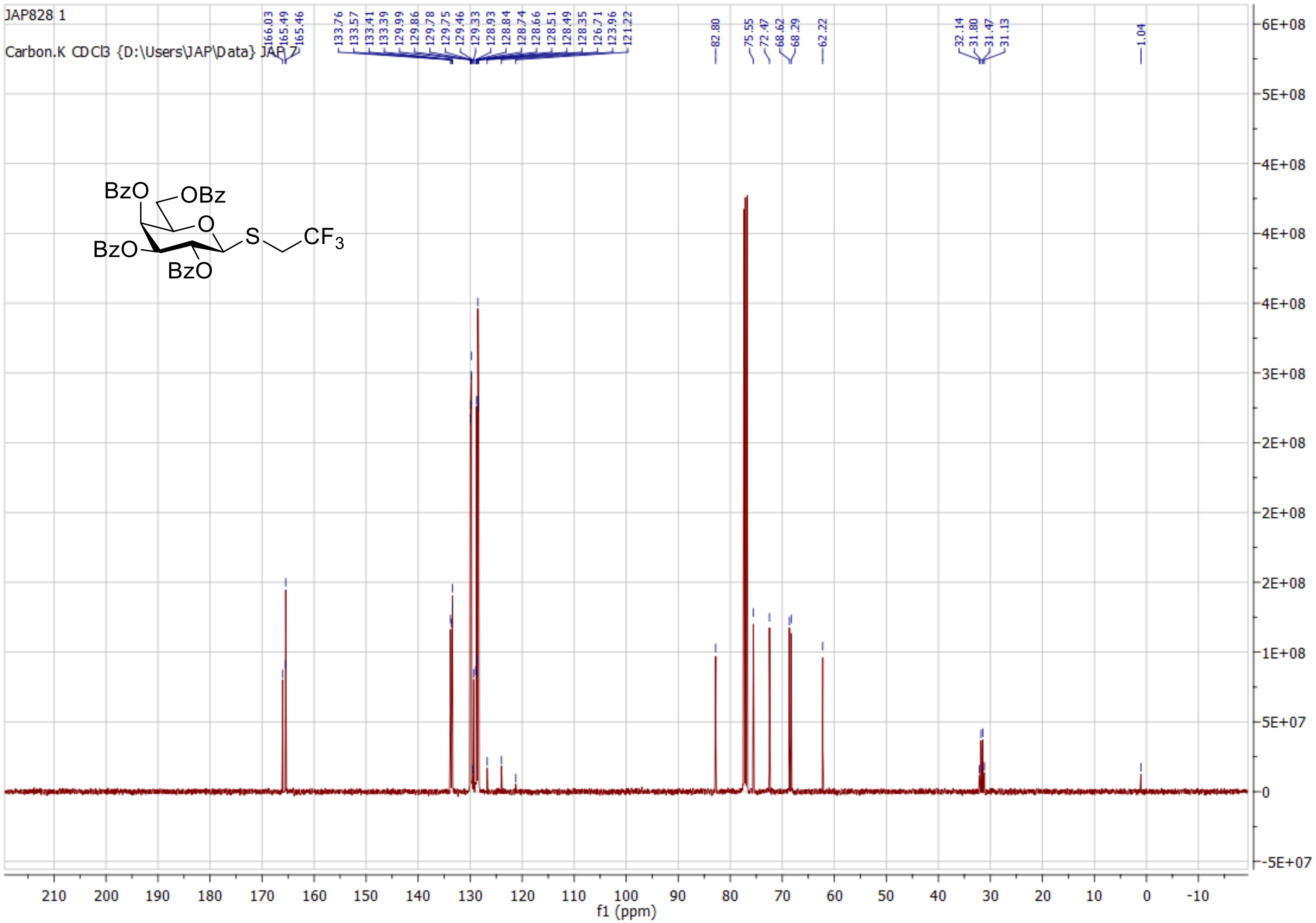

^{19}F NMR (376 MHz, $CDCl_3$): Trifluoroethyl 2,4,3,6-tetra-*O*-benzoyl-1-thio-β-D-galactopyranoside 46

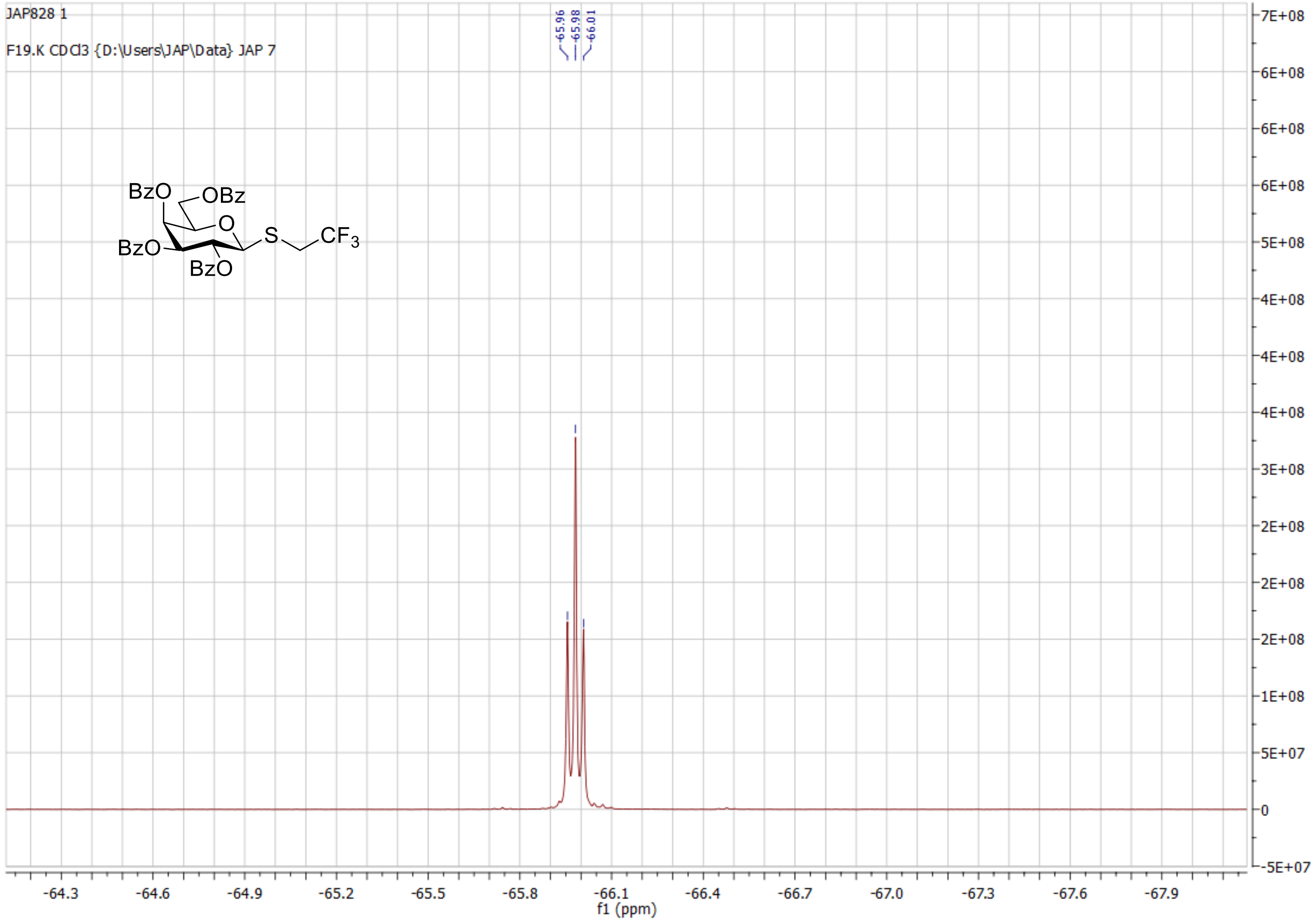

Compounds 47/48

^{1}H NMR (400 MHz, $CDCl_3$): Trifluoroethyl 3,4,6-tri-*O*-benzoyl-1-thio-β-D-galactopyranoside 47 & trifluoroethyl 2,3,6-tri-*O*-benzoyl-1-thio-β-D-galactopyranoside 48

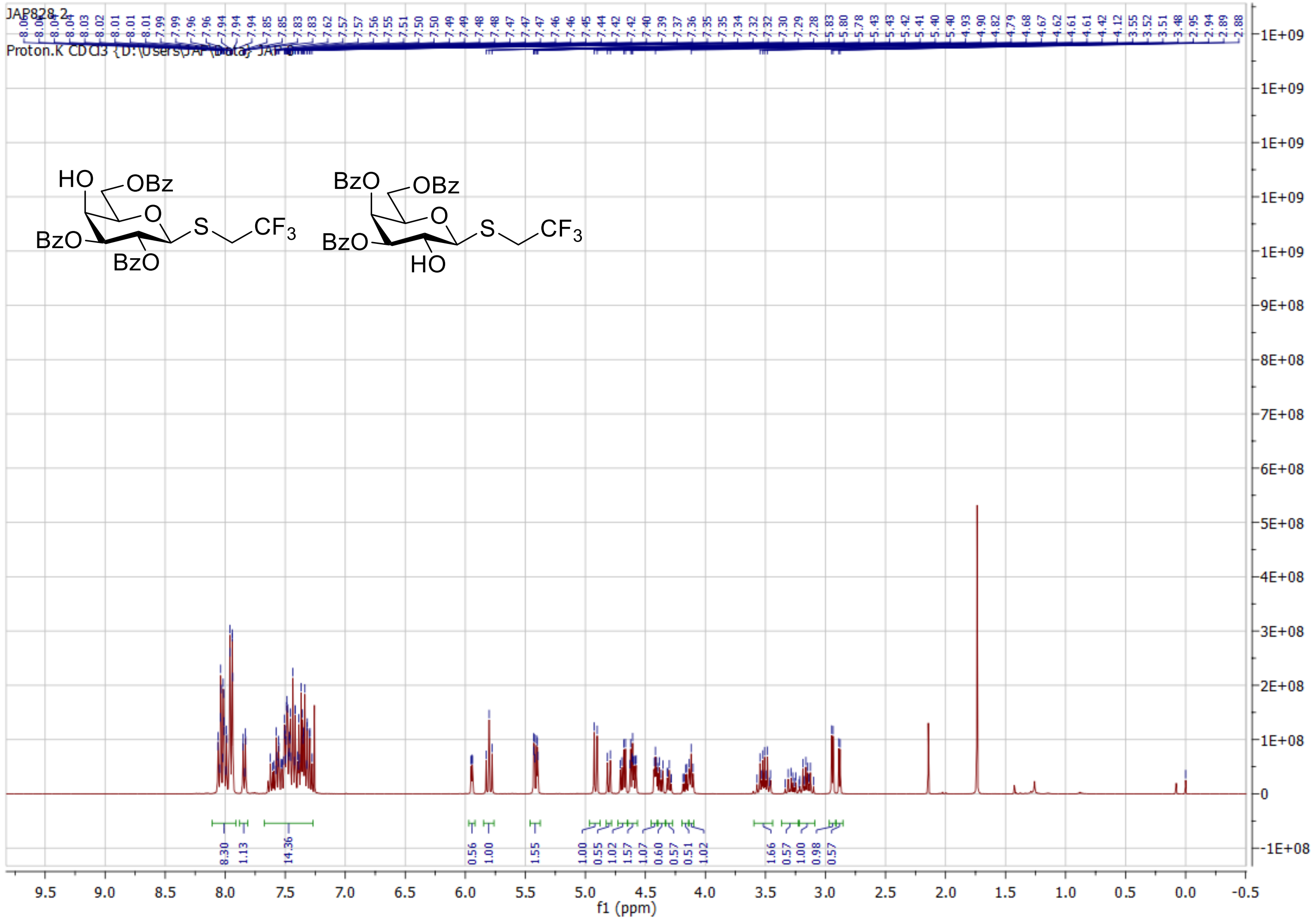

COSY (400 x 400 MHz, CDCl₃): Trifluoroethyl 3,4,6-tri-*O*-benzoyl-1-thio-β-D-galactopyranoside 47 & trifluoroethyl 2,3,6-tri-*O*-benzoyl-1-thio-β-D-galactopyranoside 48

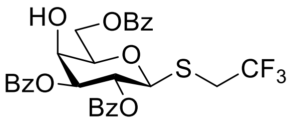

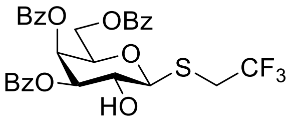

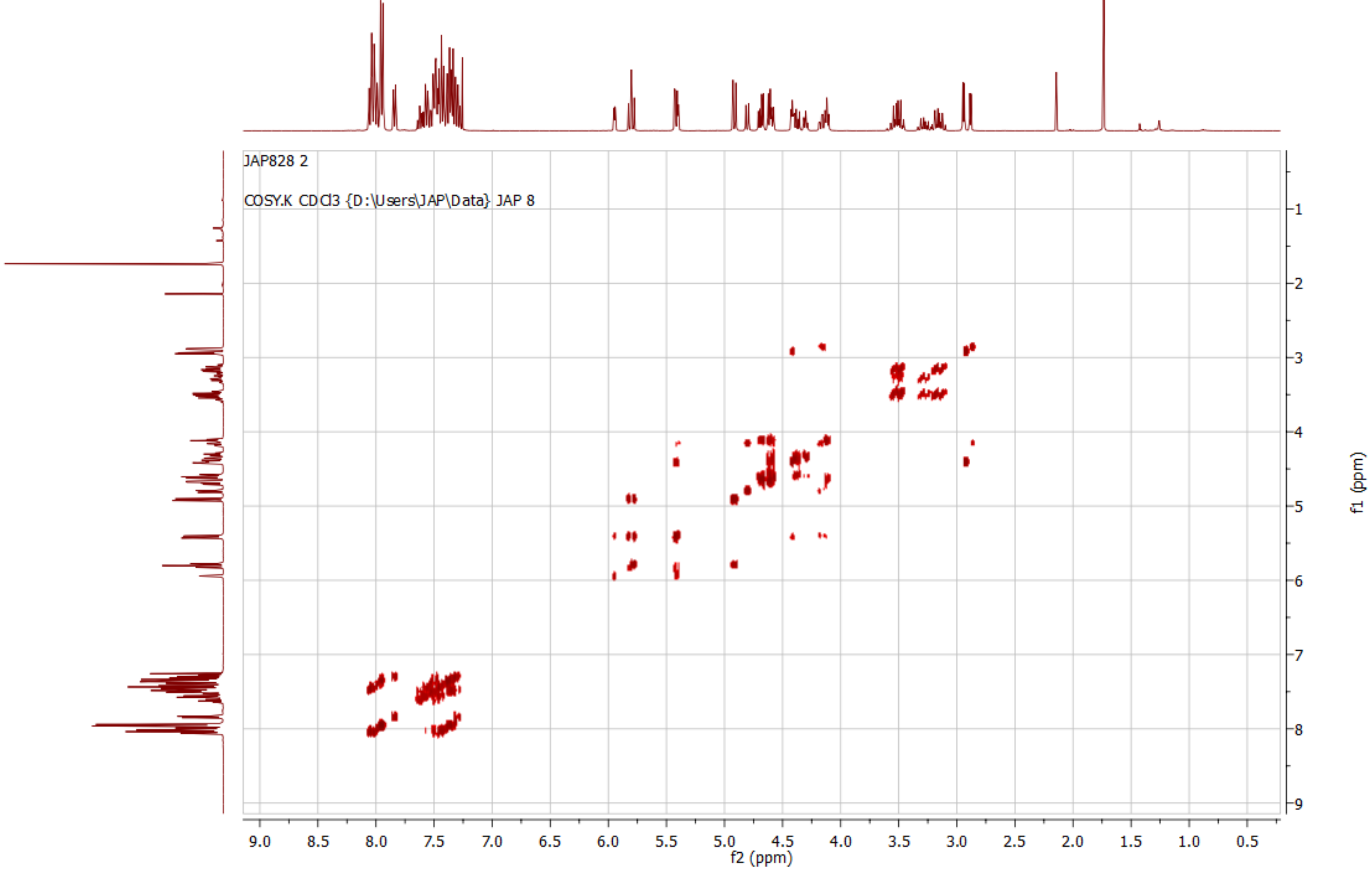

HSQC (400 × 101 MHz, $CDCl_3$): Trifluoroethyl 3,4,6-tri-*O*-benzoyl-1-thio-β-D-galactopyranoside 47 & trifluoroethyl 2,3,6-tri-*O*-benzoyl-1-thio-β-D-galactopyranoside 48

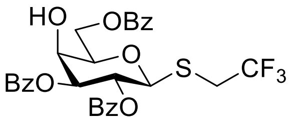

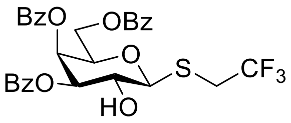

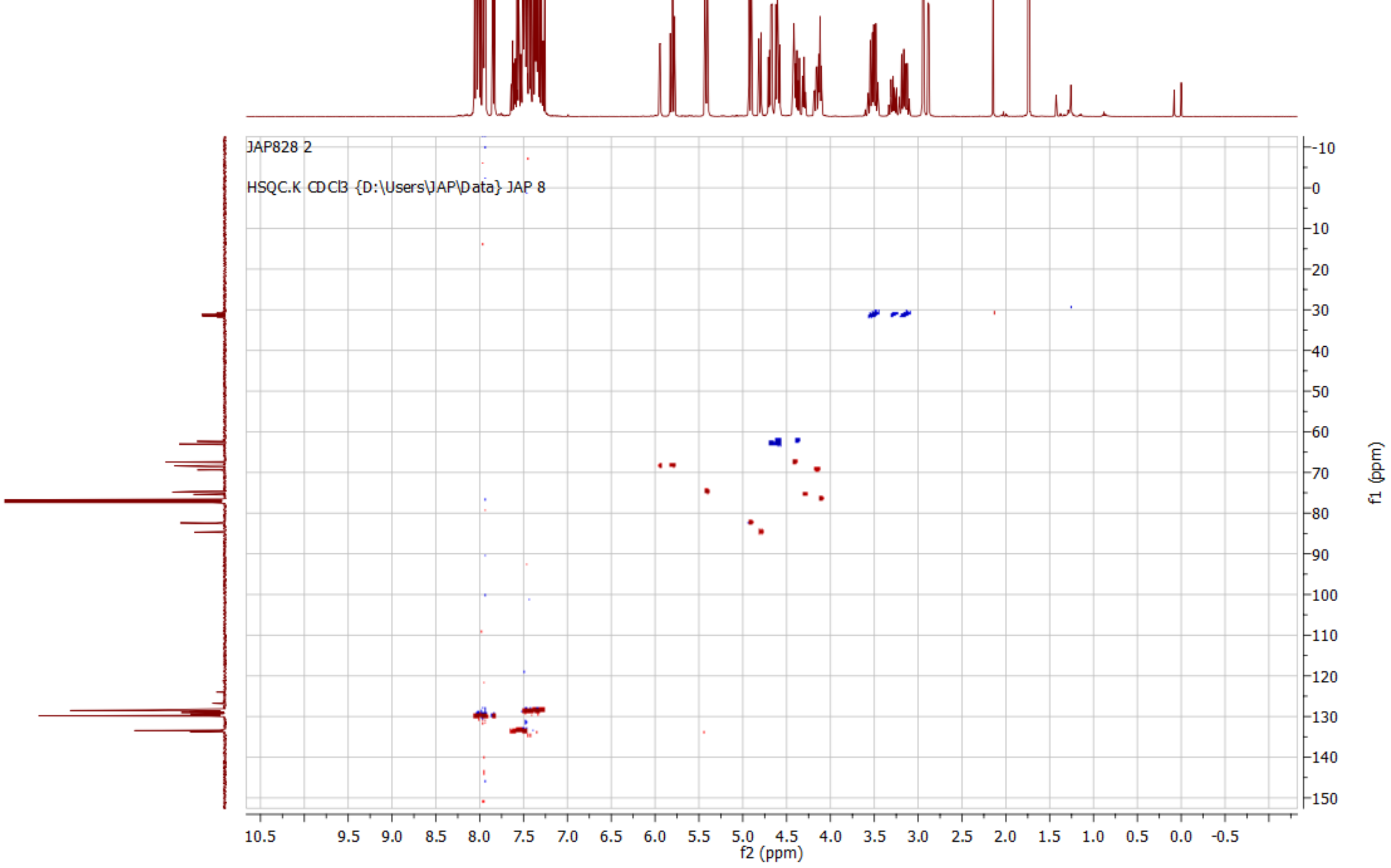

^{13}C{1H} NMR (101 MHz, CDCl₃): Trifluoroethyl 3,4,6-tri-*O*-benzoyl-1-thio-β-D-galactopyranoside 47 & trifluoroethyl 2,3,6-tri-*O*-benzoyl-1-thio-β-D-galactopyranoside 48

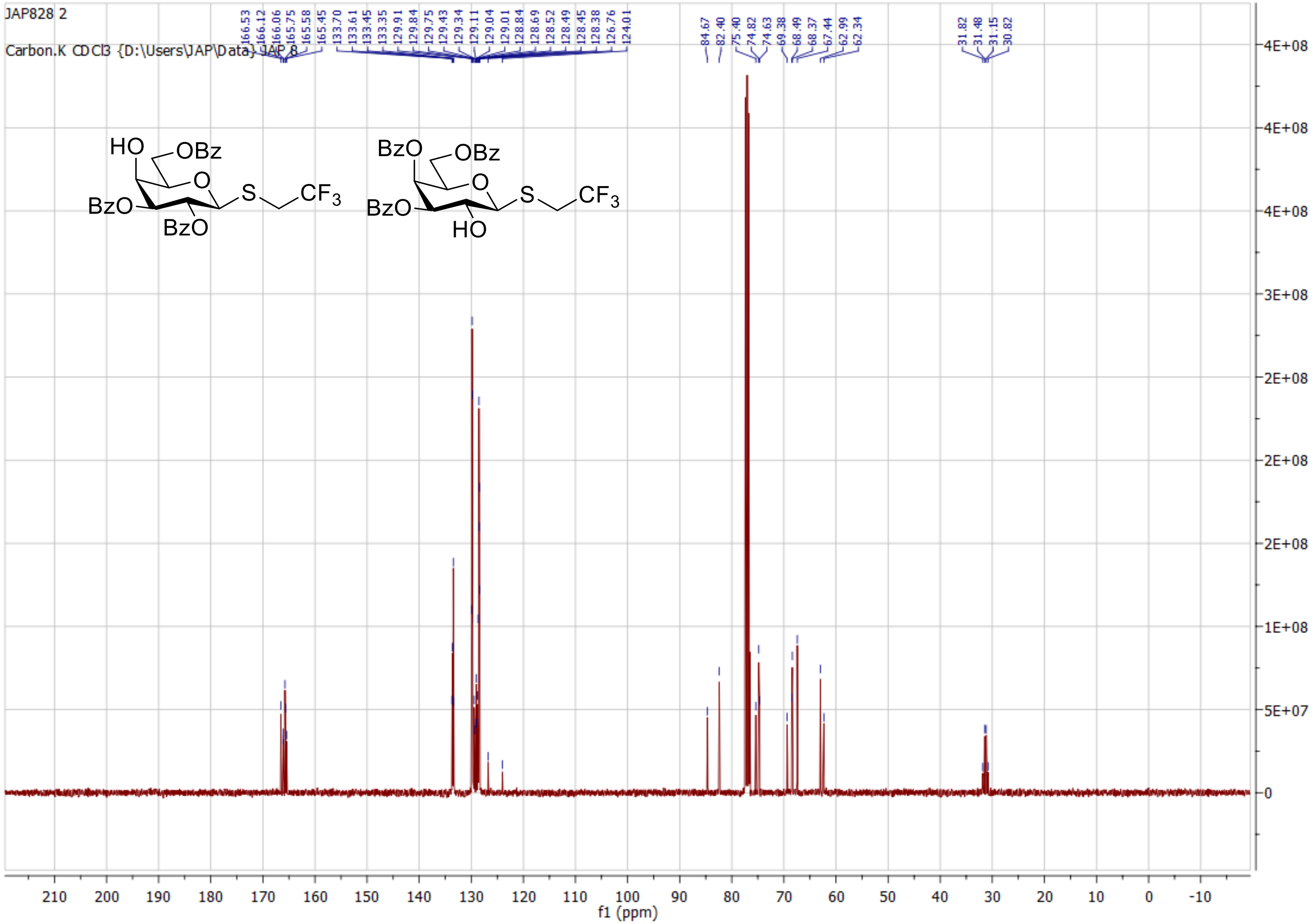

^{19}F NMR (376 MHz, CDCl3): Trifluoroethyl 3,4,6-tri-*O*-benzoyl-1-thio-β-D-galactopyranoside 47 & trifluoroethyl 2,3,6-tri-*O*-benzoyl-1-thio-β-D-galactopyranoside 48

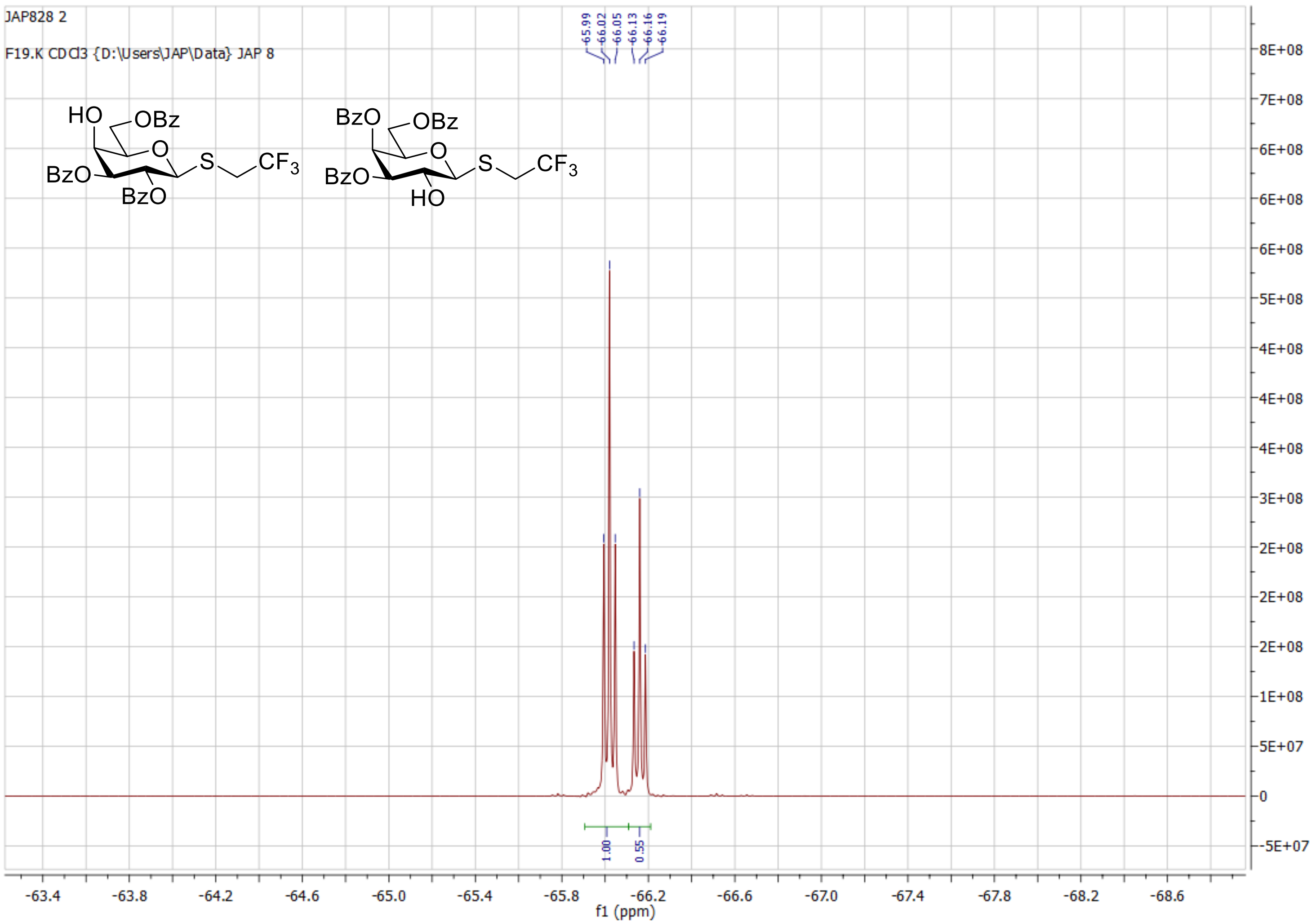

HMBC (400 × 101 MHz, CDCl₃): Trifluoroethyl 3,4,6-tri-*O*-benzoyl-1-thio-β-D-galactopyranoside 47 & trifluoroethyl 2,3,6-tri-*O*-benzoyl-1-thio-β-D-galactopyranoside 48

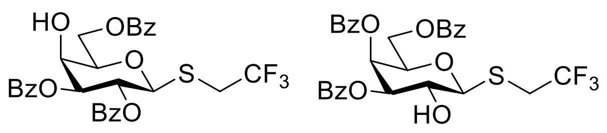

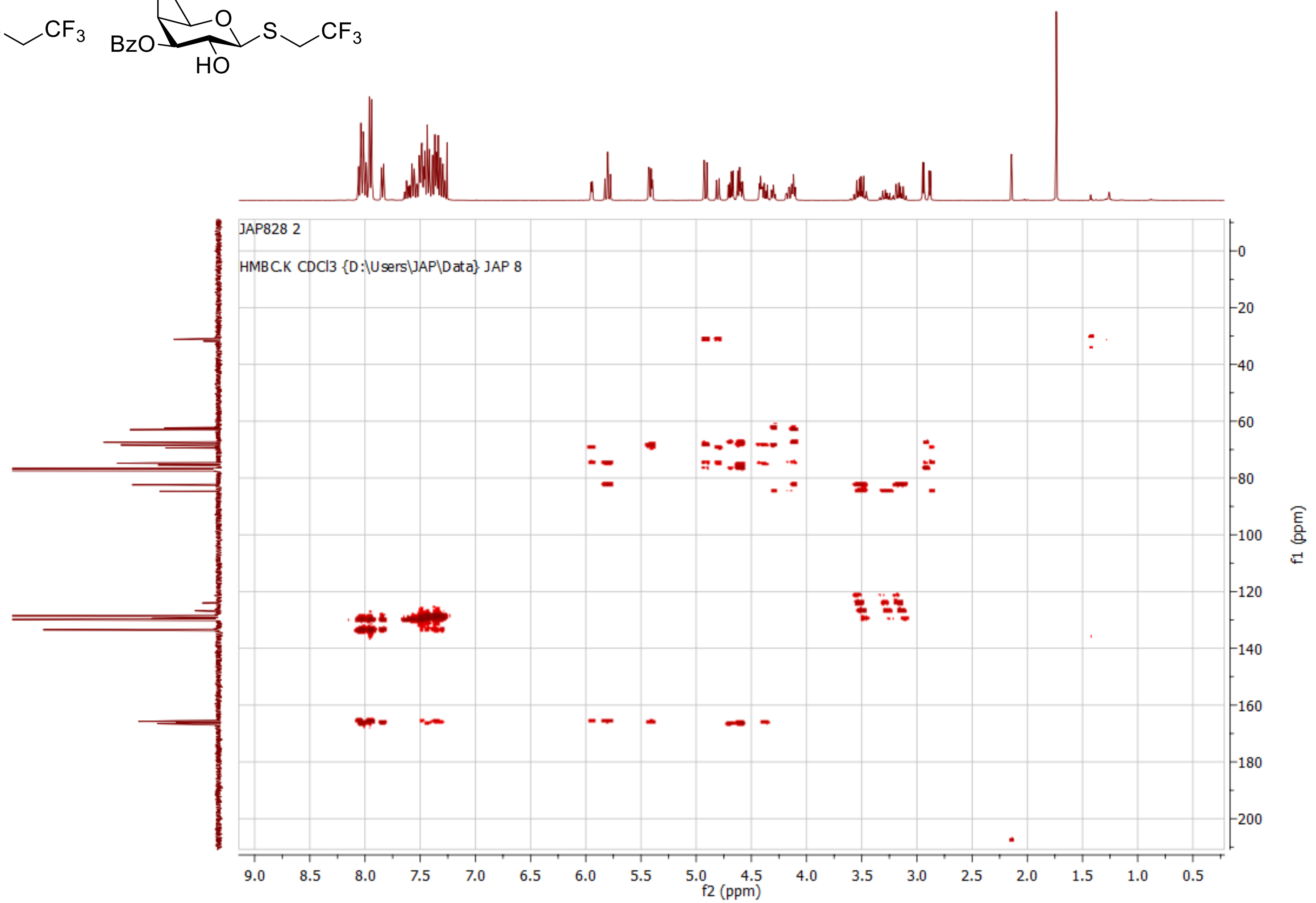

Compound 50

^{1}H NMR (400 MHz, CDCl3): Cyclohexyl 2,3,4,6-tetra-*O*-benozyl-β-D-galactopyranoside 50

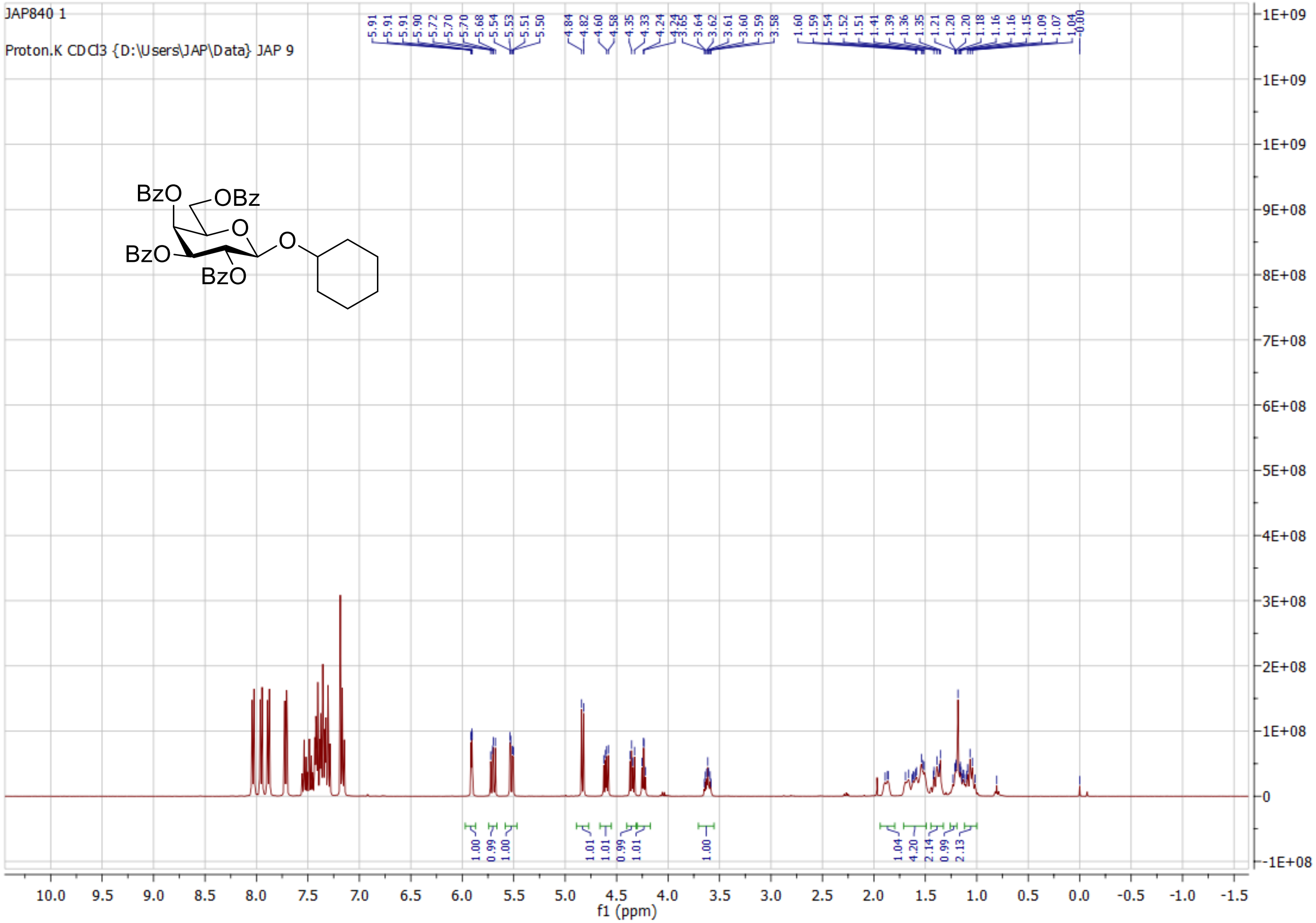

COSY (400 × 400 MHz, $CDCl_3$): Cyclohexyl 2,3,4,6-tetra-*O*-benozyl-β-D-galactopyranoside 50

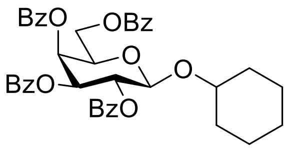

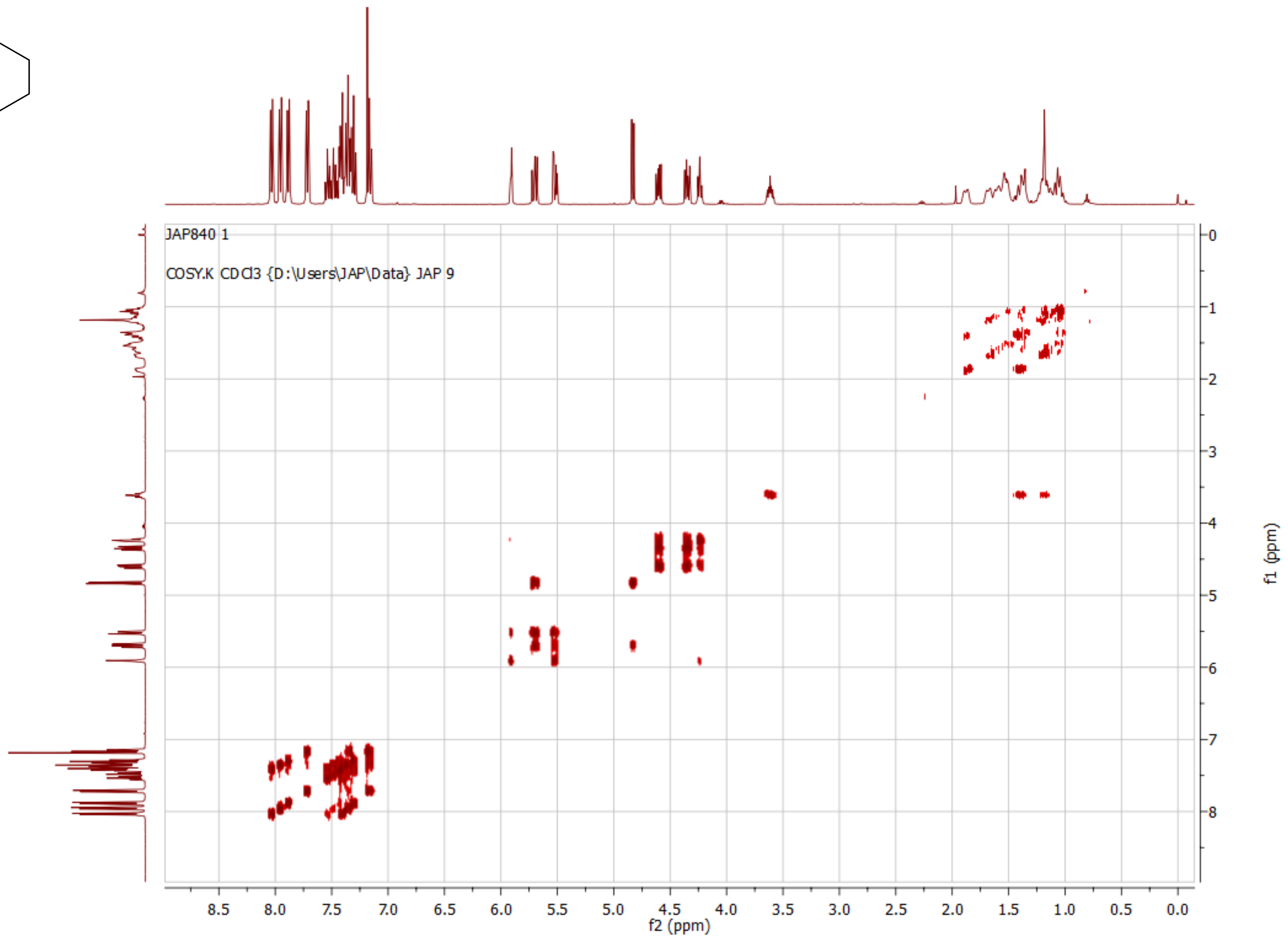

HSQC (400 × 101 MHz, CDCl₃): Cyclohexyl 2,3,4,6-tetra-*O*-benozyl-β-D-galactopyranoside 50

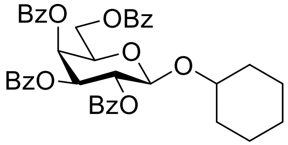

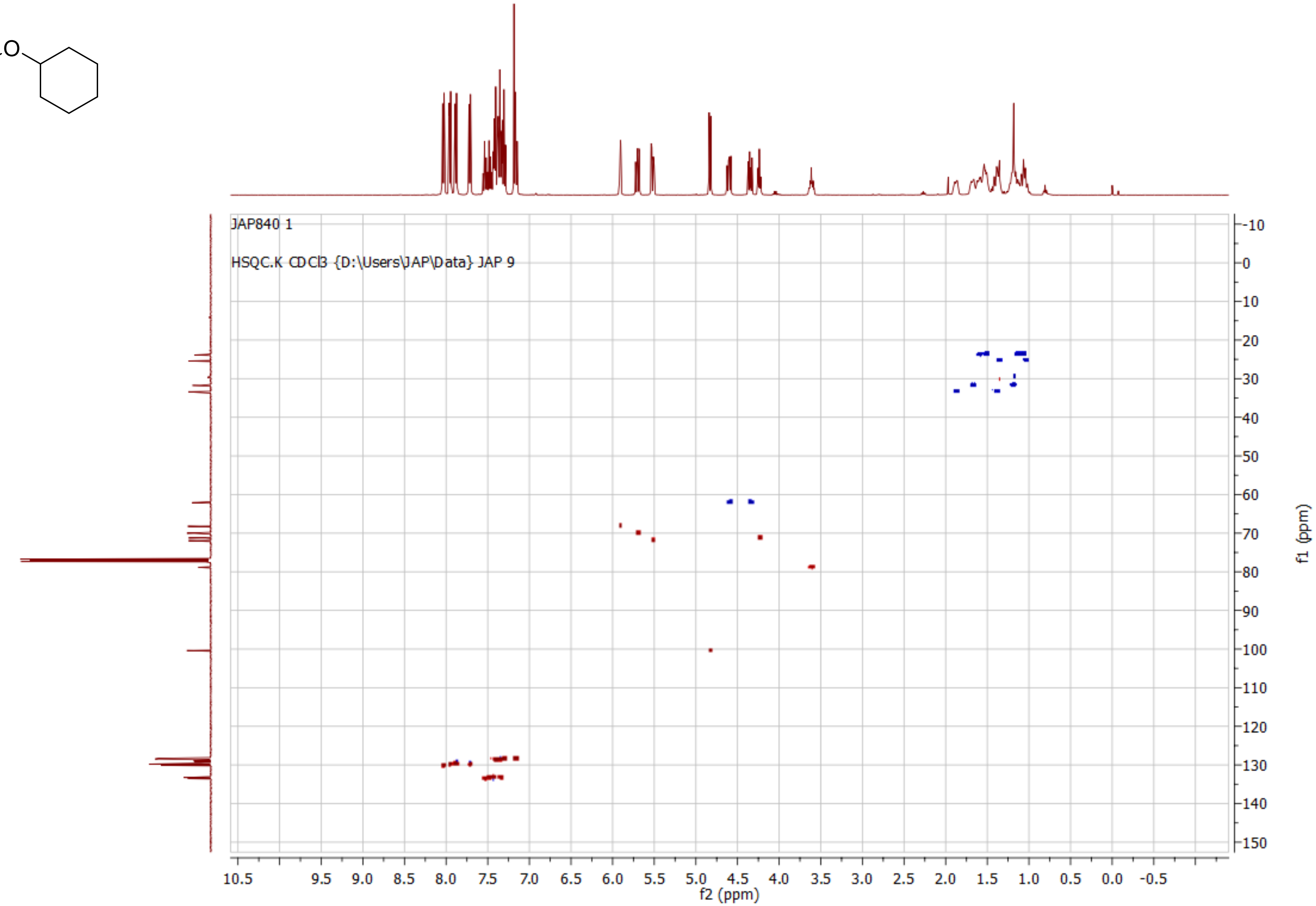

^{13}C{1H} NMR (101 MHz, $CDCl_3$): Cyclohexyl 2,3,4,6-tetra-*O*-benozyl-β-D-galactopyranoside 50

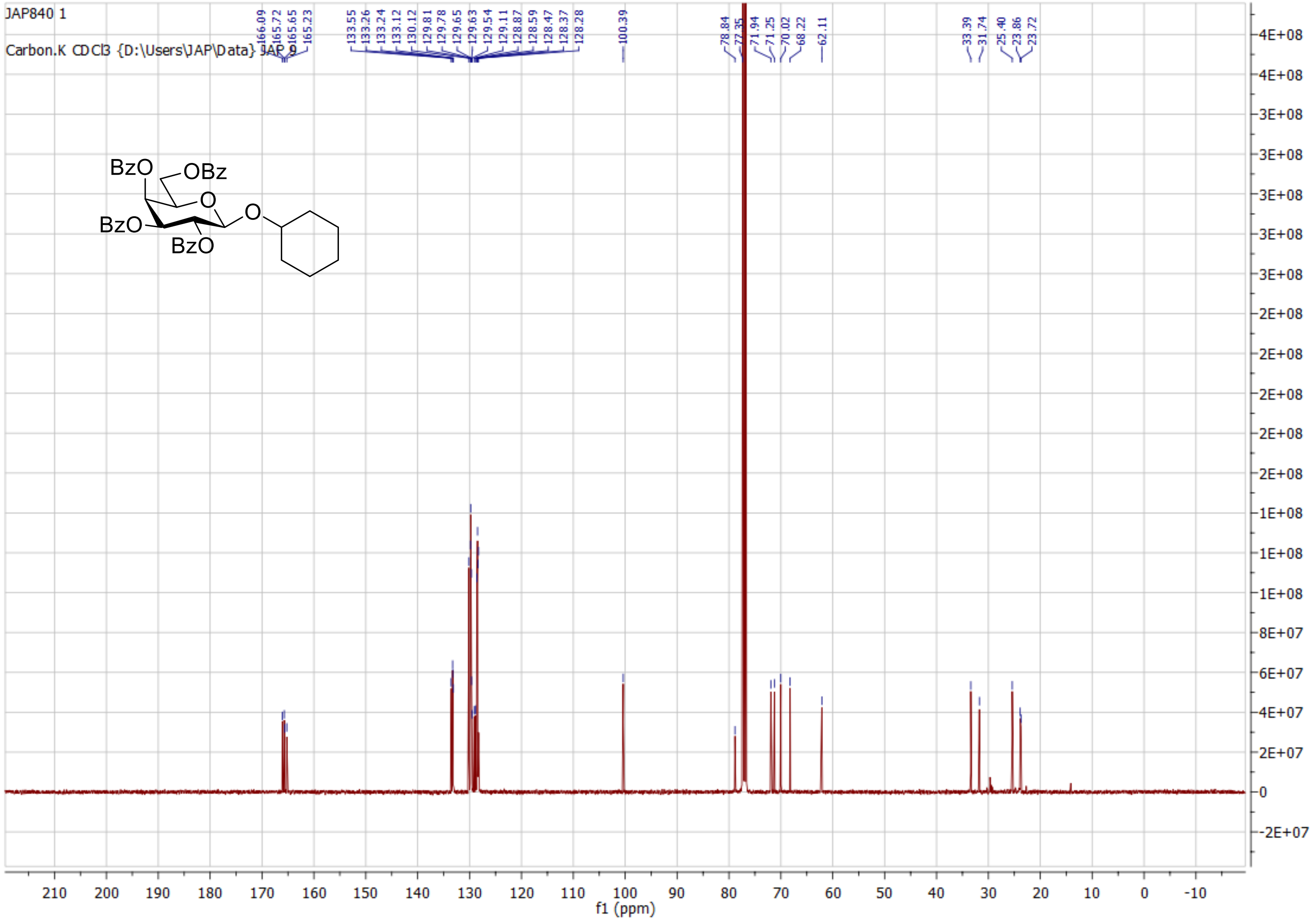

Compound 51/52

^{1}H NMR (400 MHz, CDCl3): Cyclohexyl 2,3,6-tri-*O*-benozyl-β-D-galactopyranoside 52 & Cyclohexyl 3,4,6-tri-*O*-benozyl-β-D-galactopyranoside 51

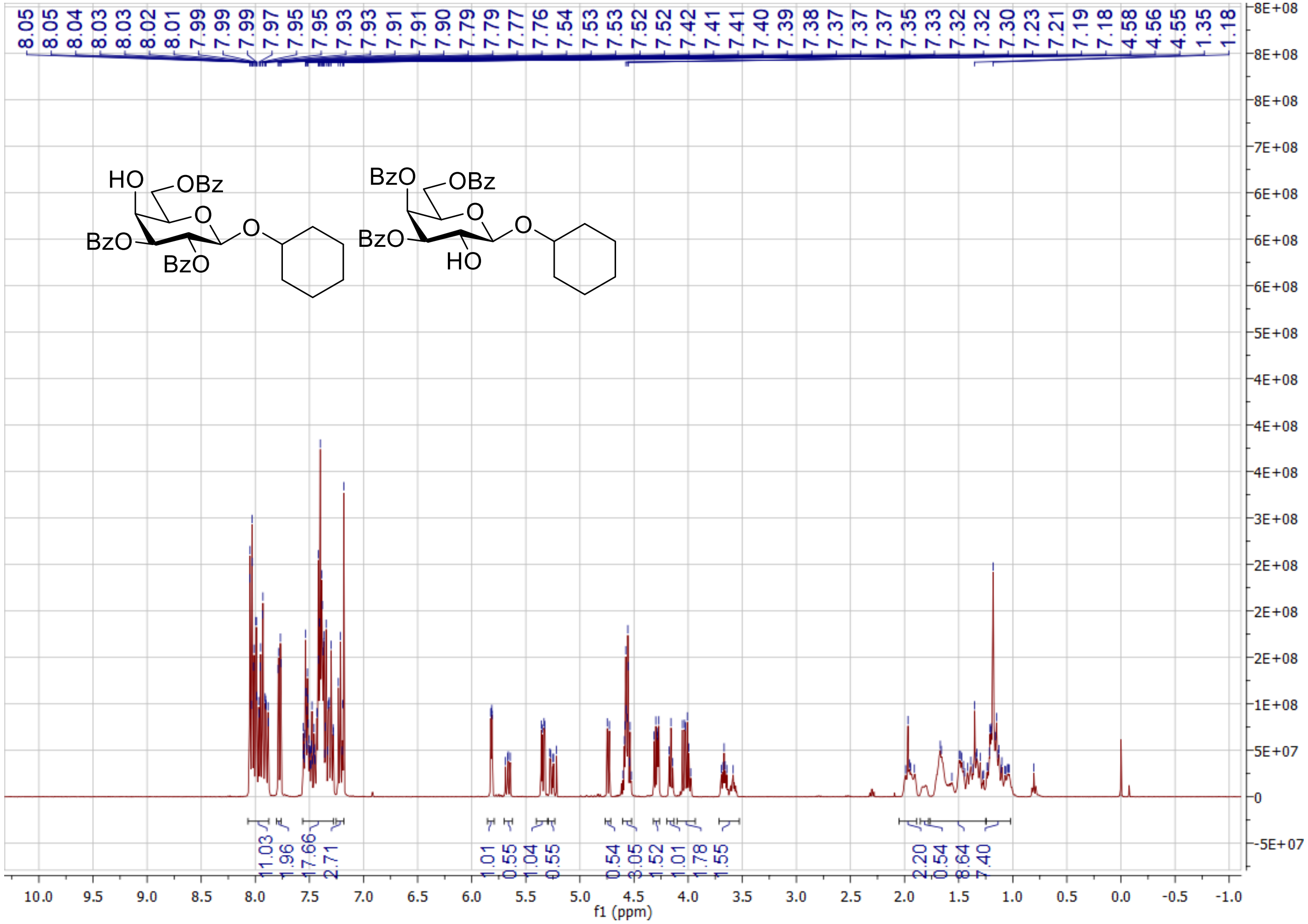

COSY (400 × 400 MHz, CDCl$_3$): Cyclohexyl 2,3,6-tri-*O*-benozyl-β-D-galactopyranoside 52 & Cyclohexyl 3,4,6-tri-*O*-benozyl-β-D-galactopyranoside 51

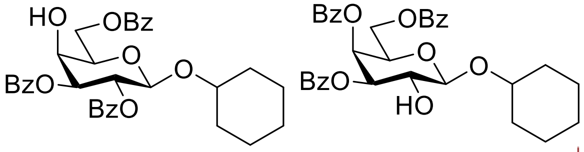

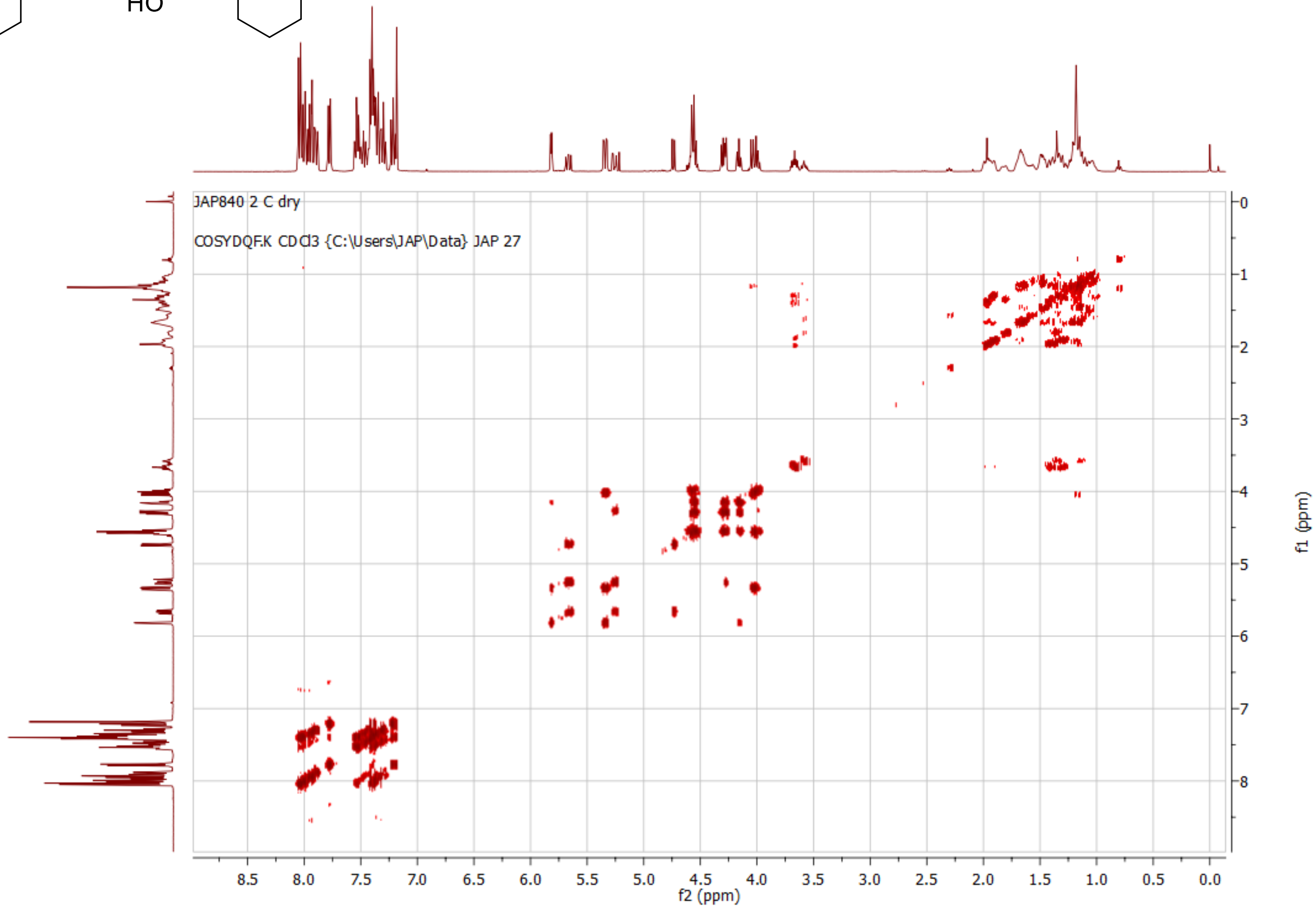

HSQC (400 × 101 MHz, $CDCl_3$): Cyclohexyl 2,3,6-tri-*O*-benozyl-β-D-galactopyranoside 52 & Cyclohexyl 3,4,6-tri-*O*-benozyl-β-D-galactopyranoside 51

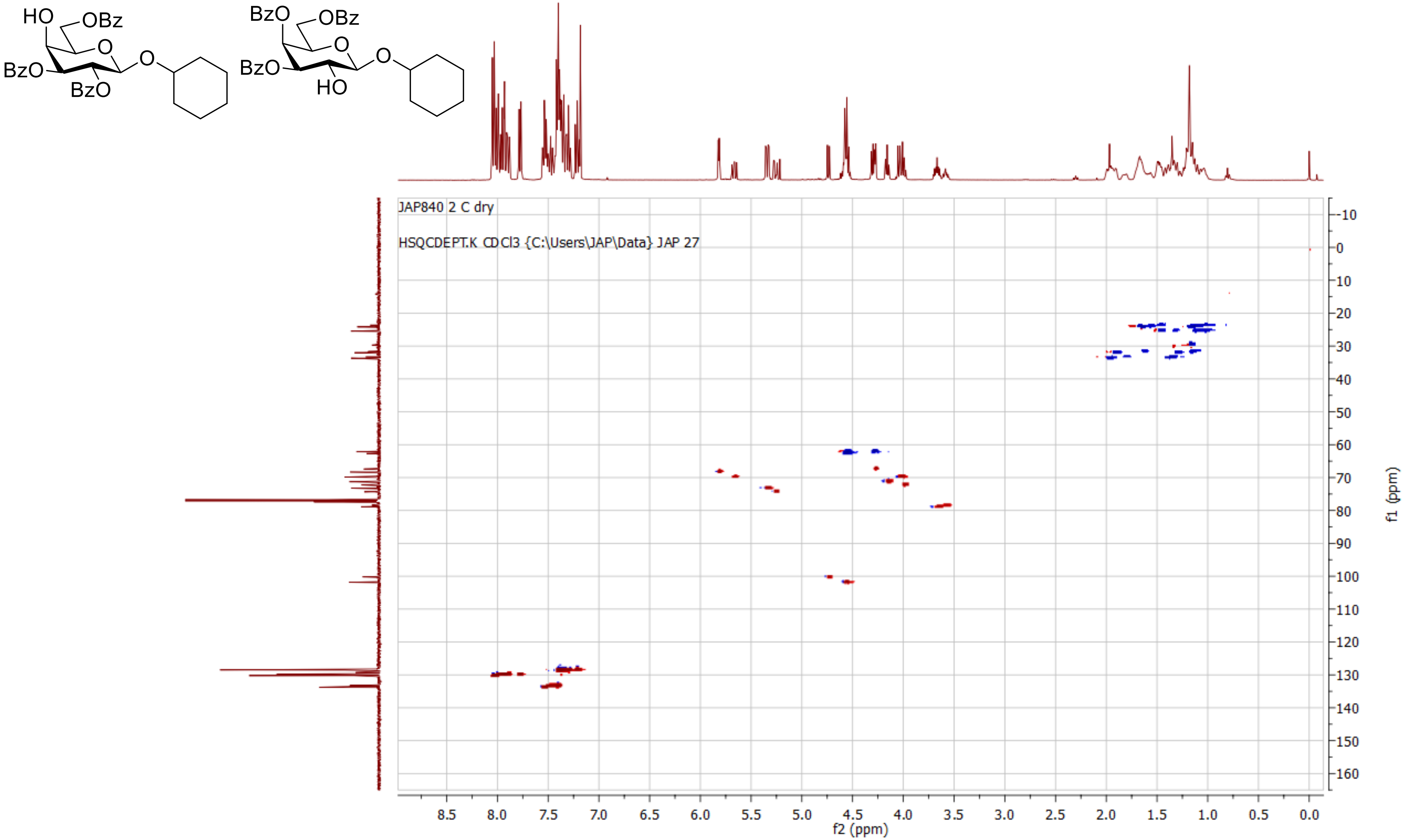

^{13}C{1H} NMR (101 MHz, CDCl₃): Cyclohexyl 2,3,6-tri-*O*-benozyl-β-D-galactopyranoside 52 & Cyclohexyl 3,4,6-tri-*O*-benozyl-β-D-galactopyranoside 51

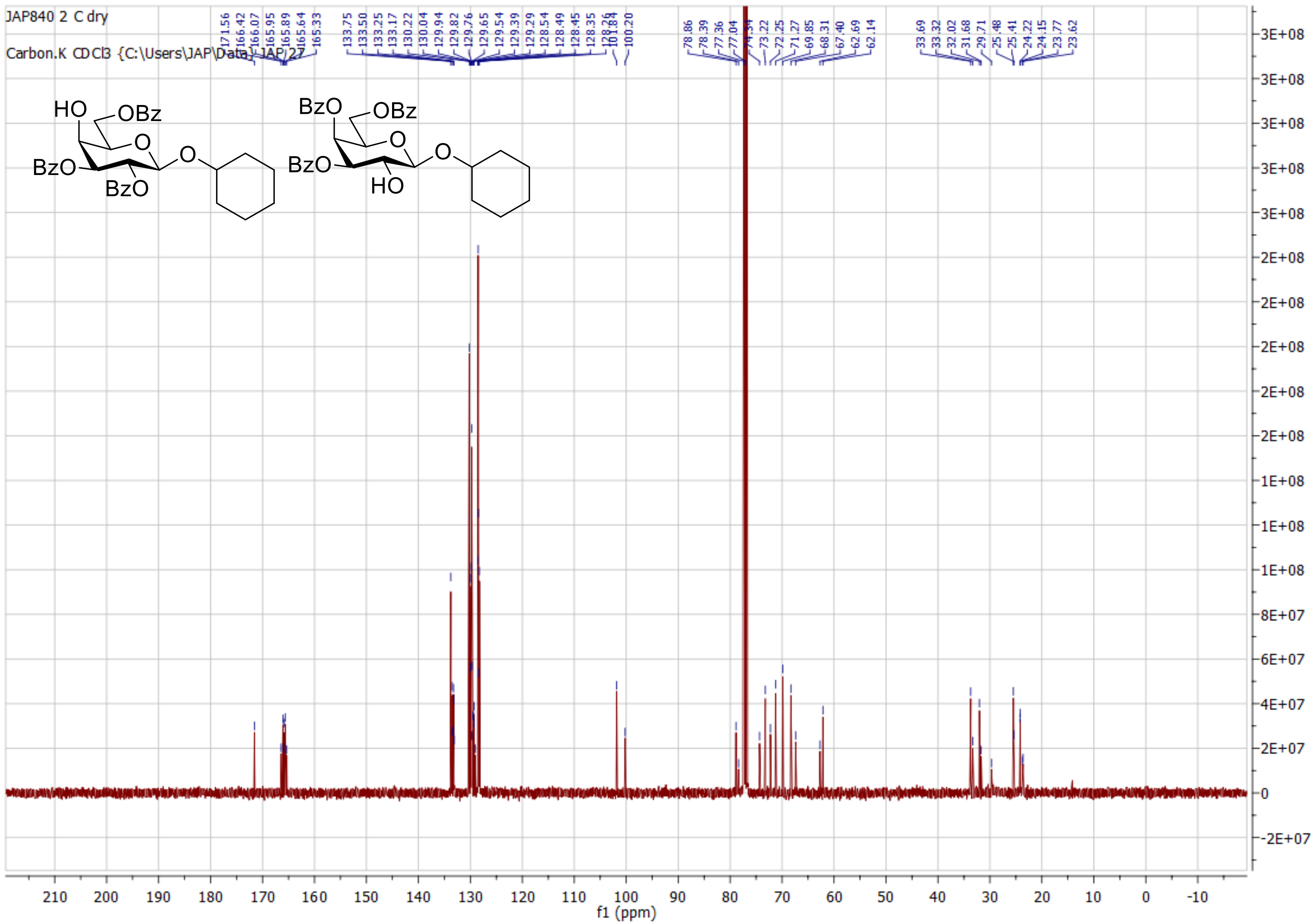

HMBC (400 × 101 MHz, CDCl₃): Cyclohexyl 2,3,6-tri-*O*-benozyl-β-D-galactopyranoside 52 & Cyclohexyl 3,4,6-tri-*O*-benozyl-β-D-galactopyranoside 51

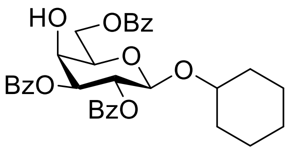

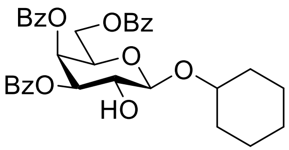

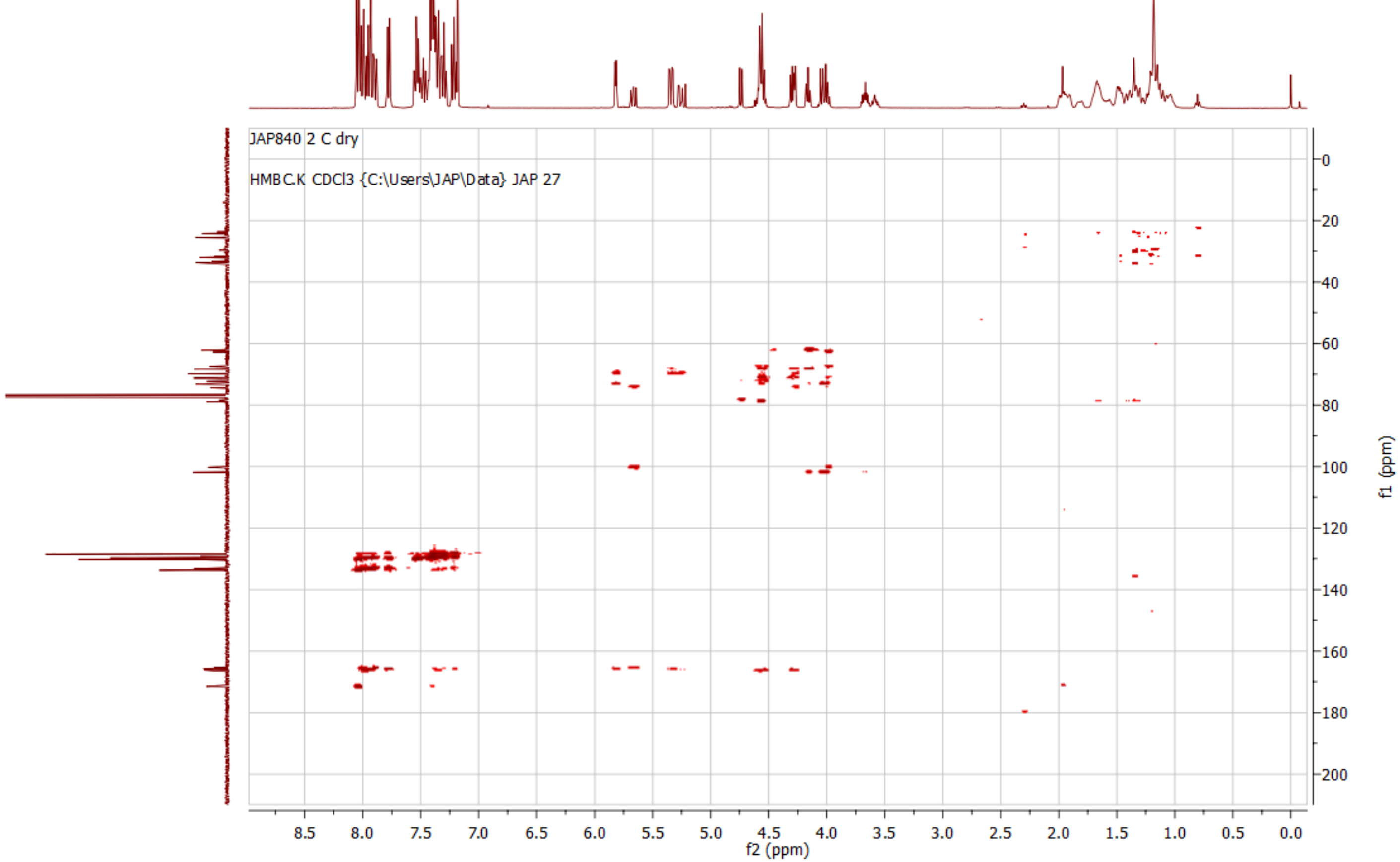

Compound 53

^{1}H NMR (400 MHz, $CDCl_3$): Cyclohexyl 3,6-di-*O*-benozyl-β-D-galactopyranoside 53

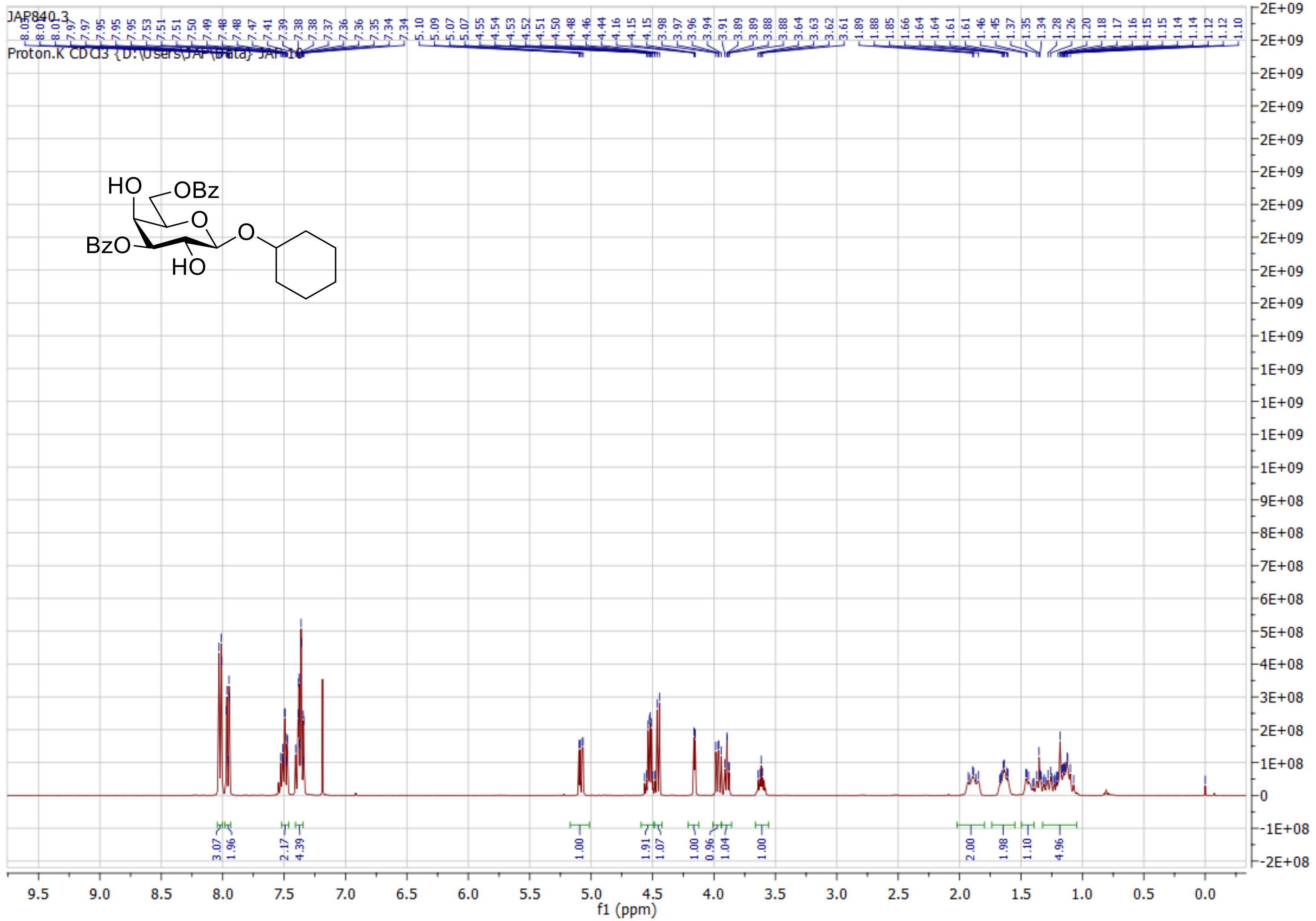

COSY (400 × 400 MHz, CDCl₃): Cyclohexyl 3,6-di-*O*-benozyl-β-D-galactopyranoside 53

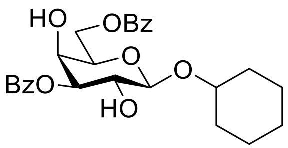

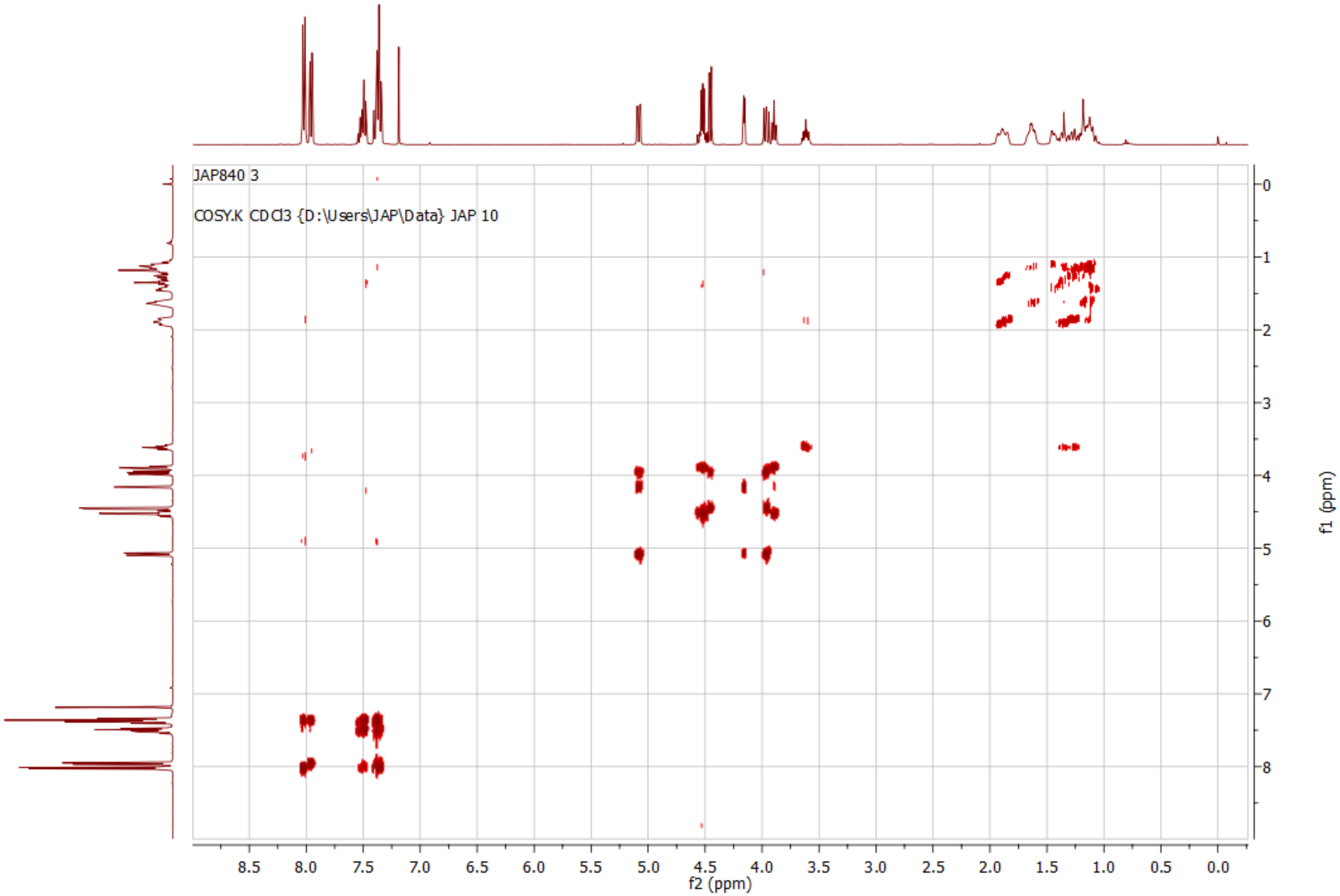

HSQC (400 × 101 MHz, $CDCl_3$): Cyclohexyl 3,6-di-*O*-benozyl-β-D-galactopyranoside 53

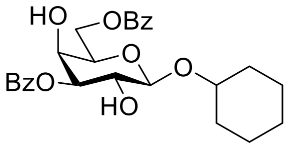

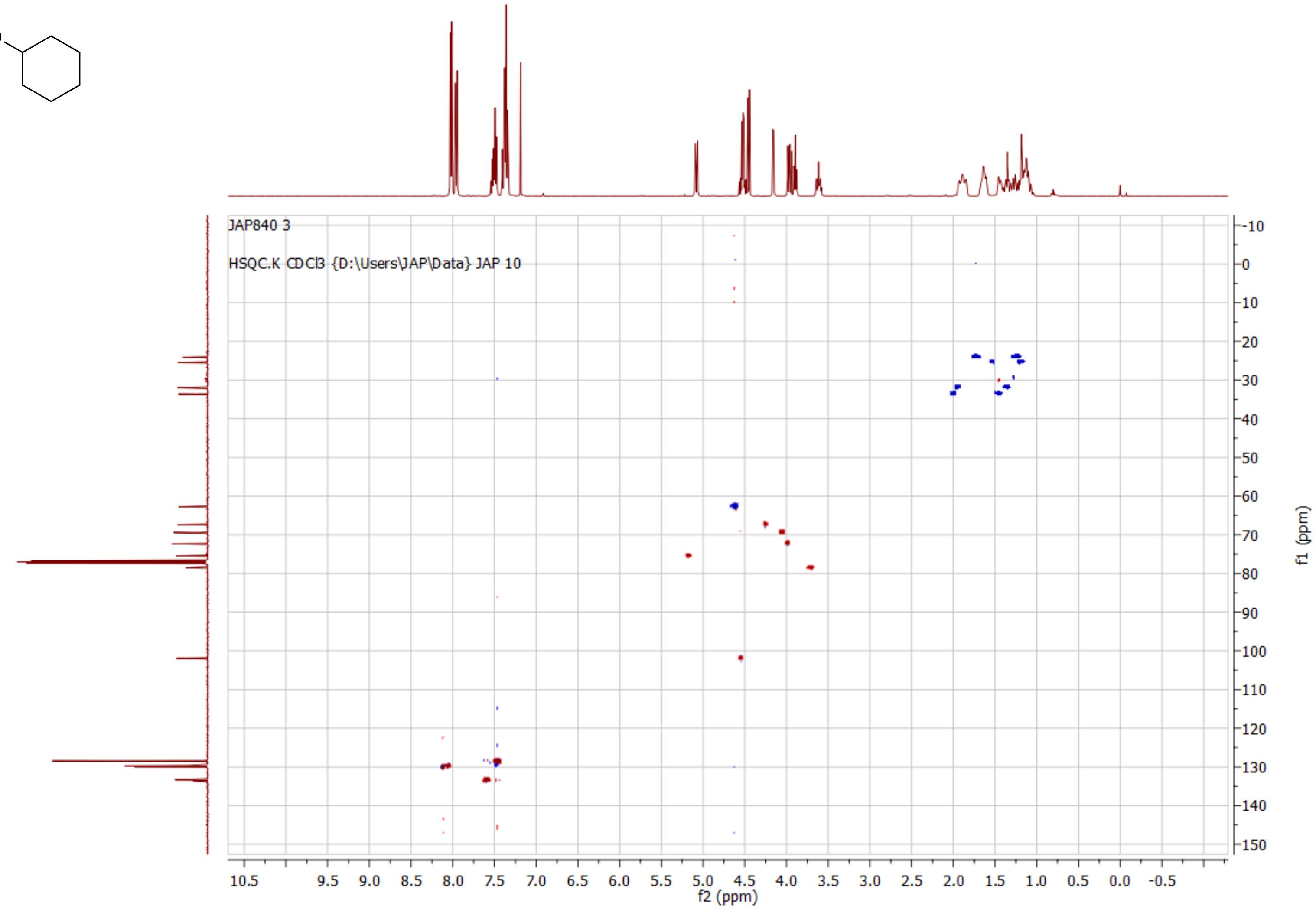

^{13}C{1H} NMR (101 MHz, $CDCl_3$): Cyclohexyl 3,6-di-*O*-benozyl-β-D-galactopyranoside 53

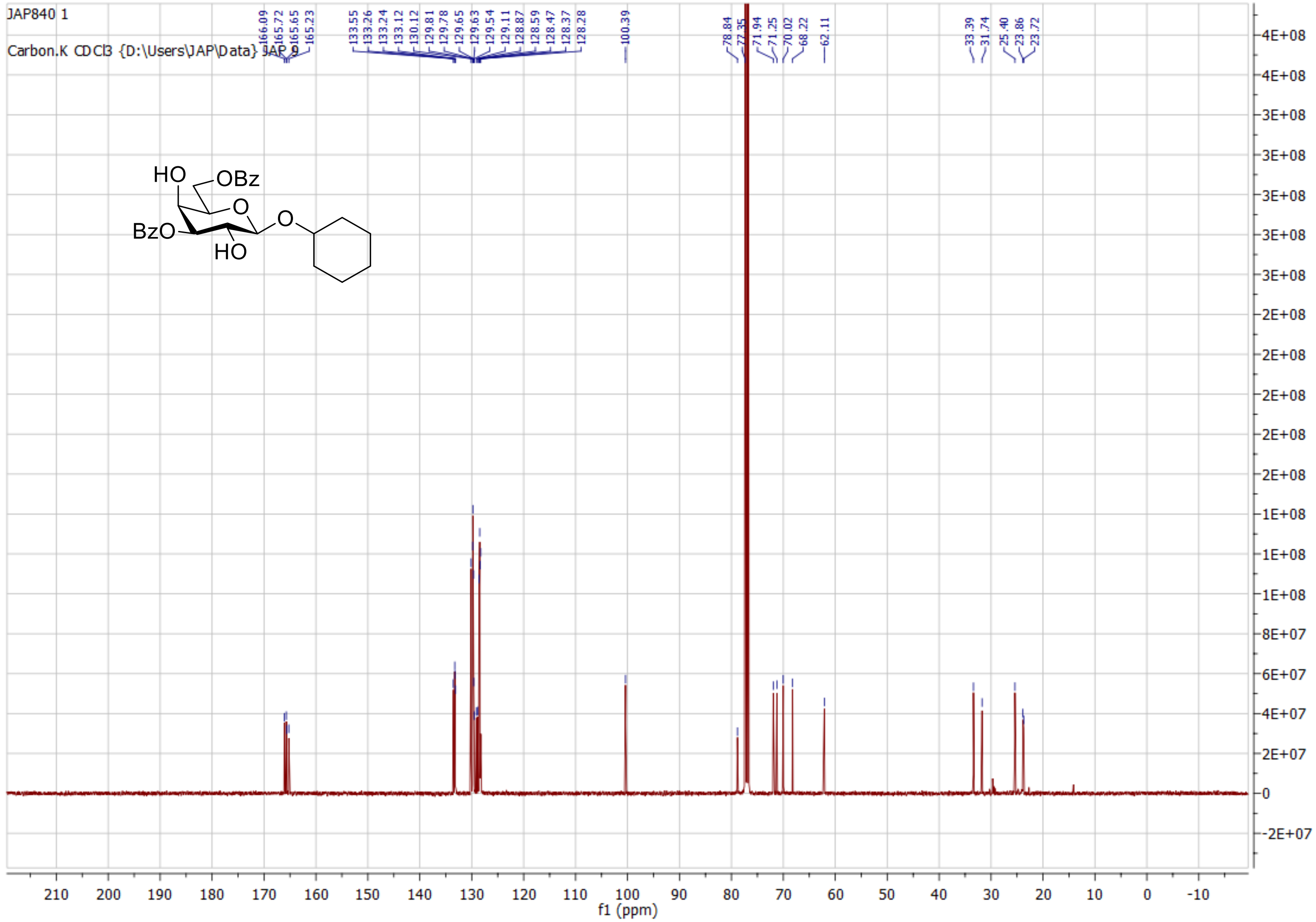

HMBC (400 × 101 MHz, CDCl₃): Cyclohexyl 3,6-di-*O*-benozyl-β-D-galactopyranoside 53

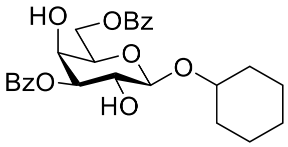

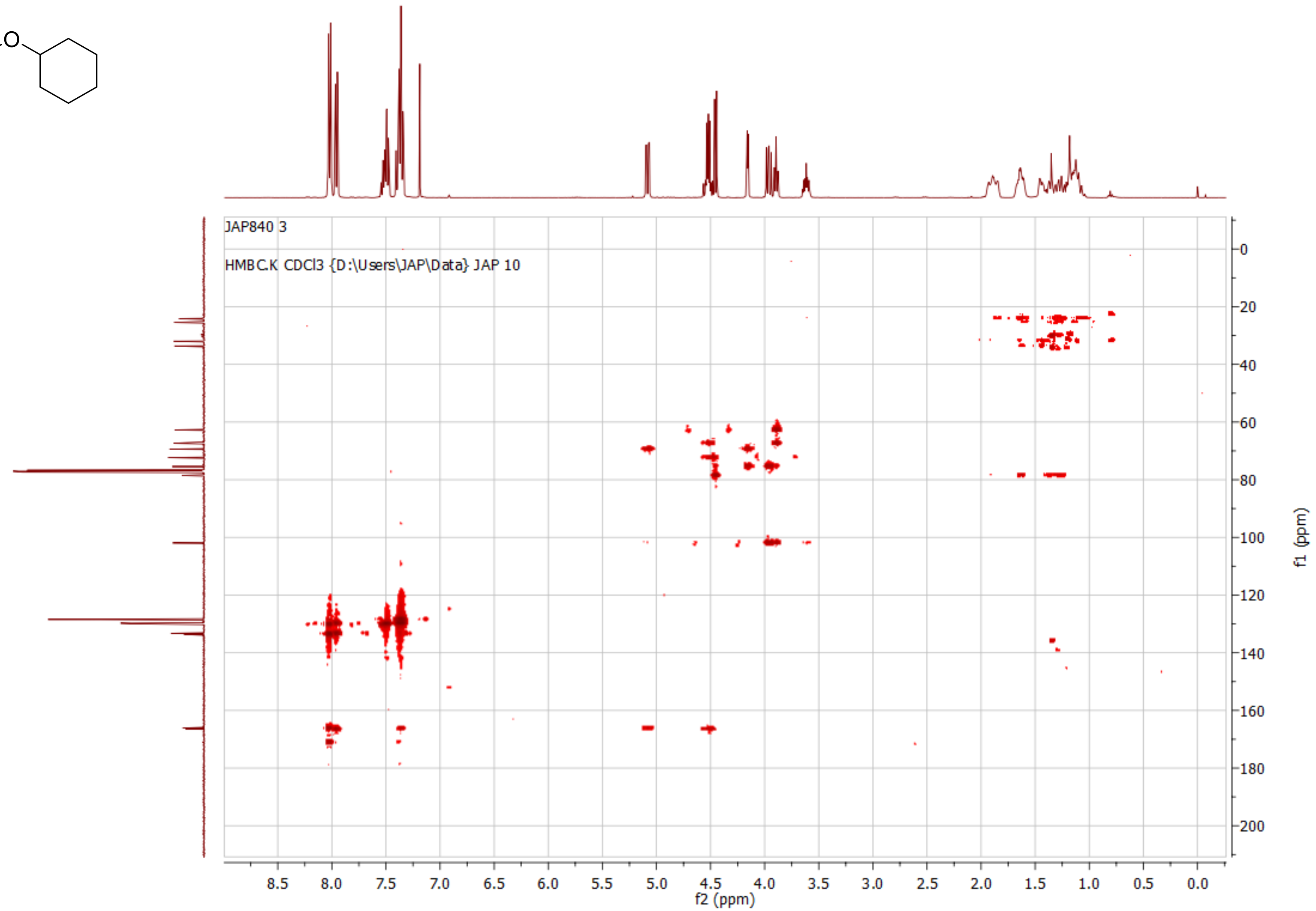

Compound 55

^{1}H NMR (400 MHz, $CDCl_3$): Trifluoroethyl 2,3,4,6-tetra-*O*-benzoyl-β-D-galactopyranoside 55

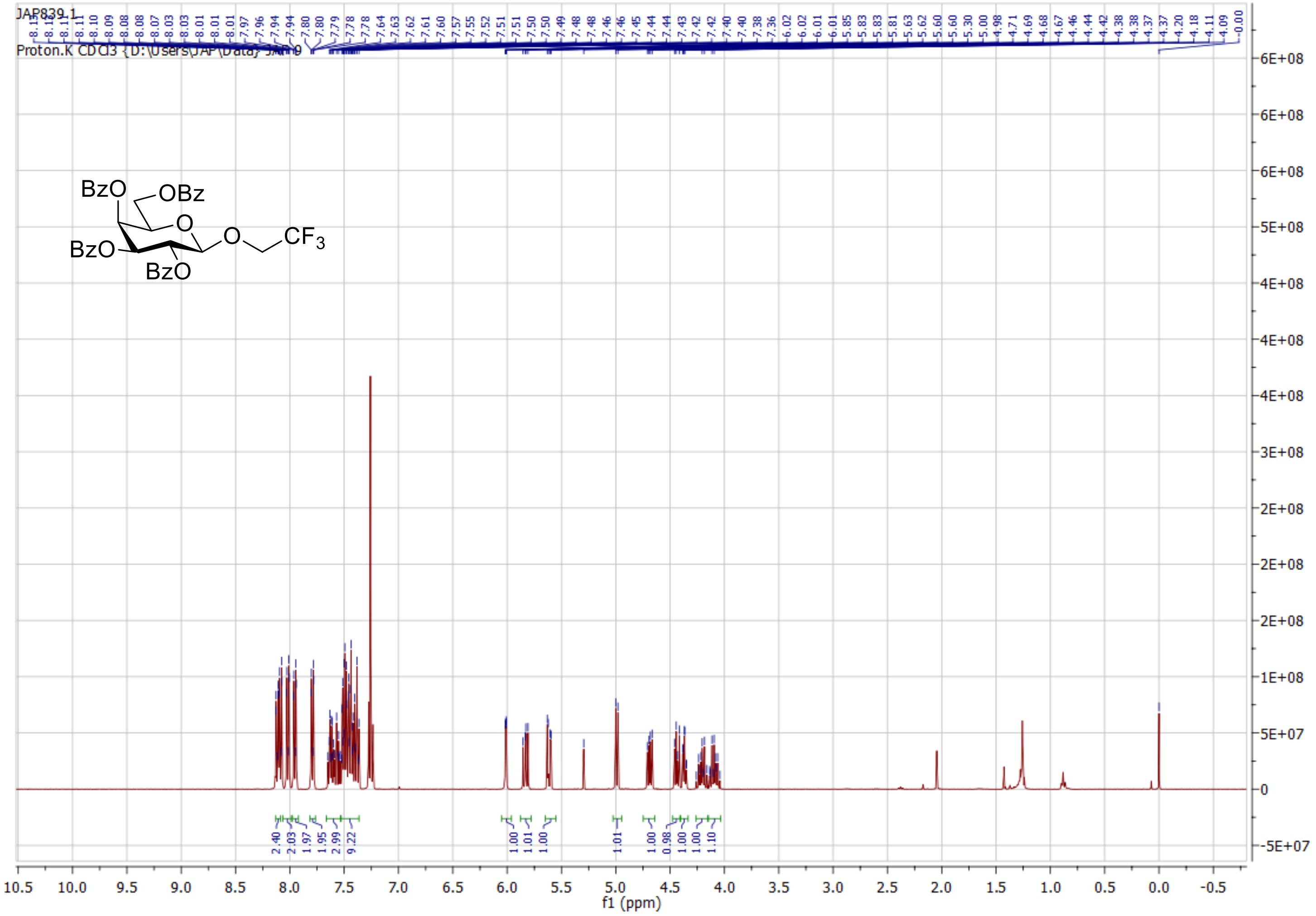

COSY (400 × 400 MHz, $CDCl_3$): Trifluoroethyl 2,3,4,6-tetra-*O*-benzoyl-β-D-galactopyranoside 55

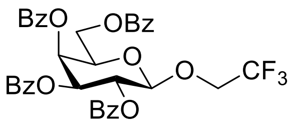

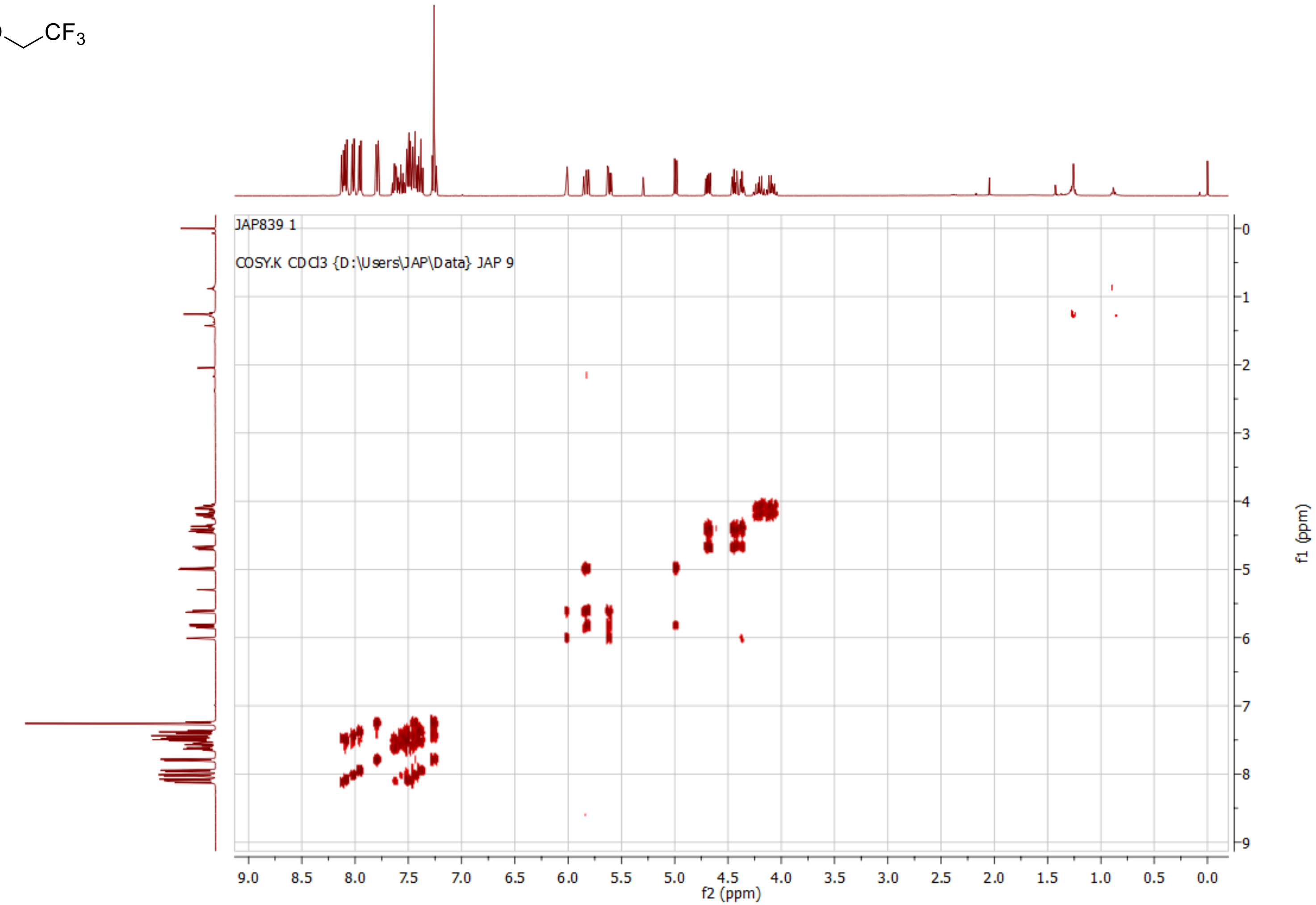

HSQC (400 × 101 MHz, CDCl₃): Trifluoroethyl 2,3,4,6-tetra-*O*-benzoyl-β-D-galactopyranoside 55

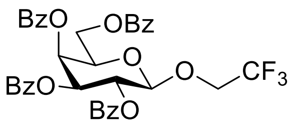

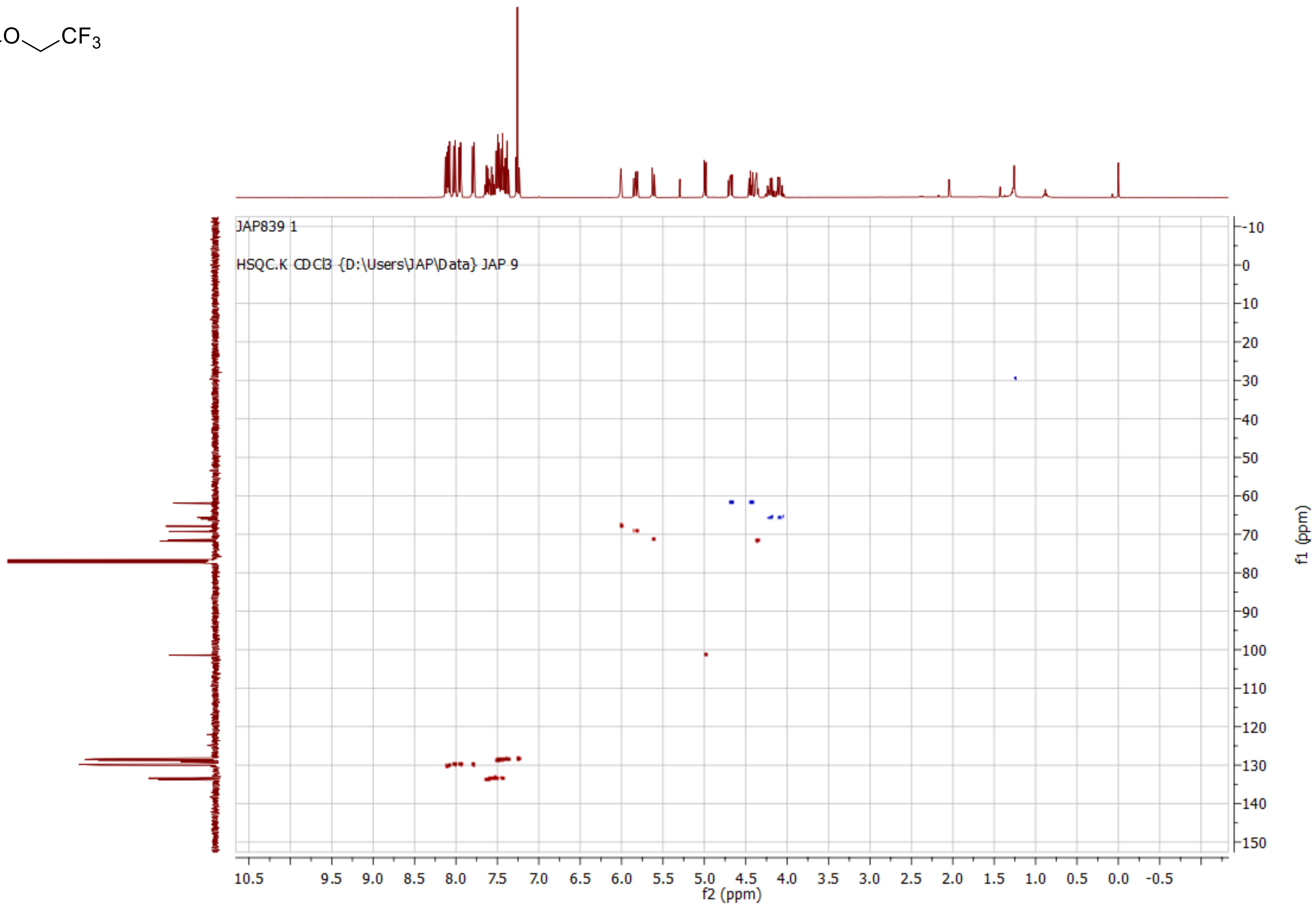

$^{13}C\{1H\}$ NMR (101 MHz, $CDCl_3$): Trifluoroethyl 2,3,4,6-tetra-*O*-benzoyl-β-D-galactopyranoside 55

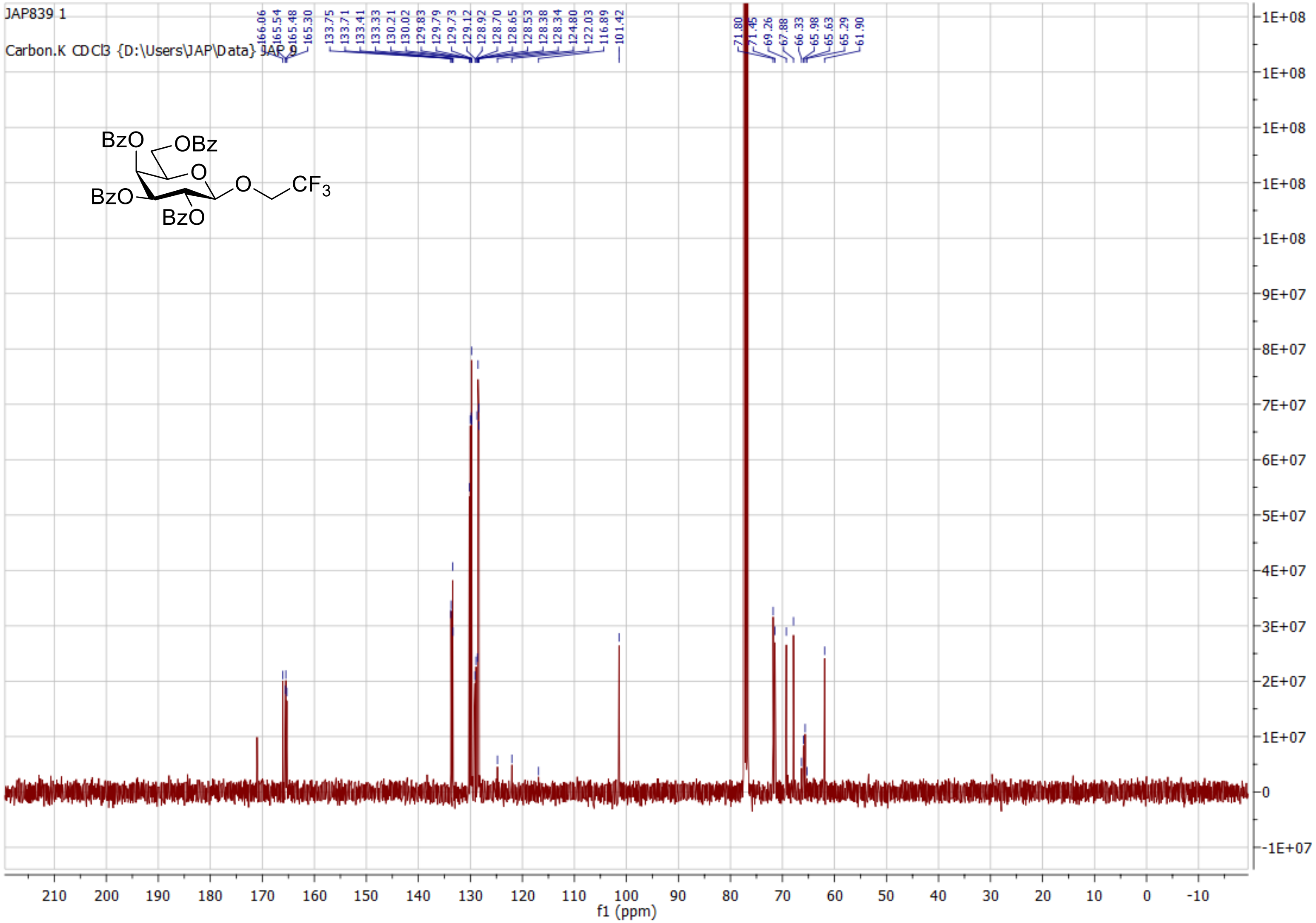

^{19}F NMR (376 MHz, $CDCl_3$): Trifluoroethyl 2,3,4,6-tetra-*O*-benzoyl-β-D-galactopyranoside 55

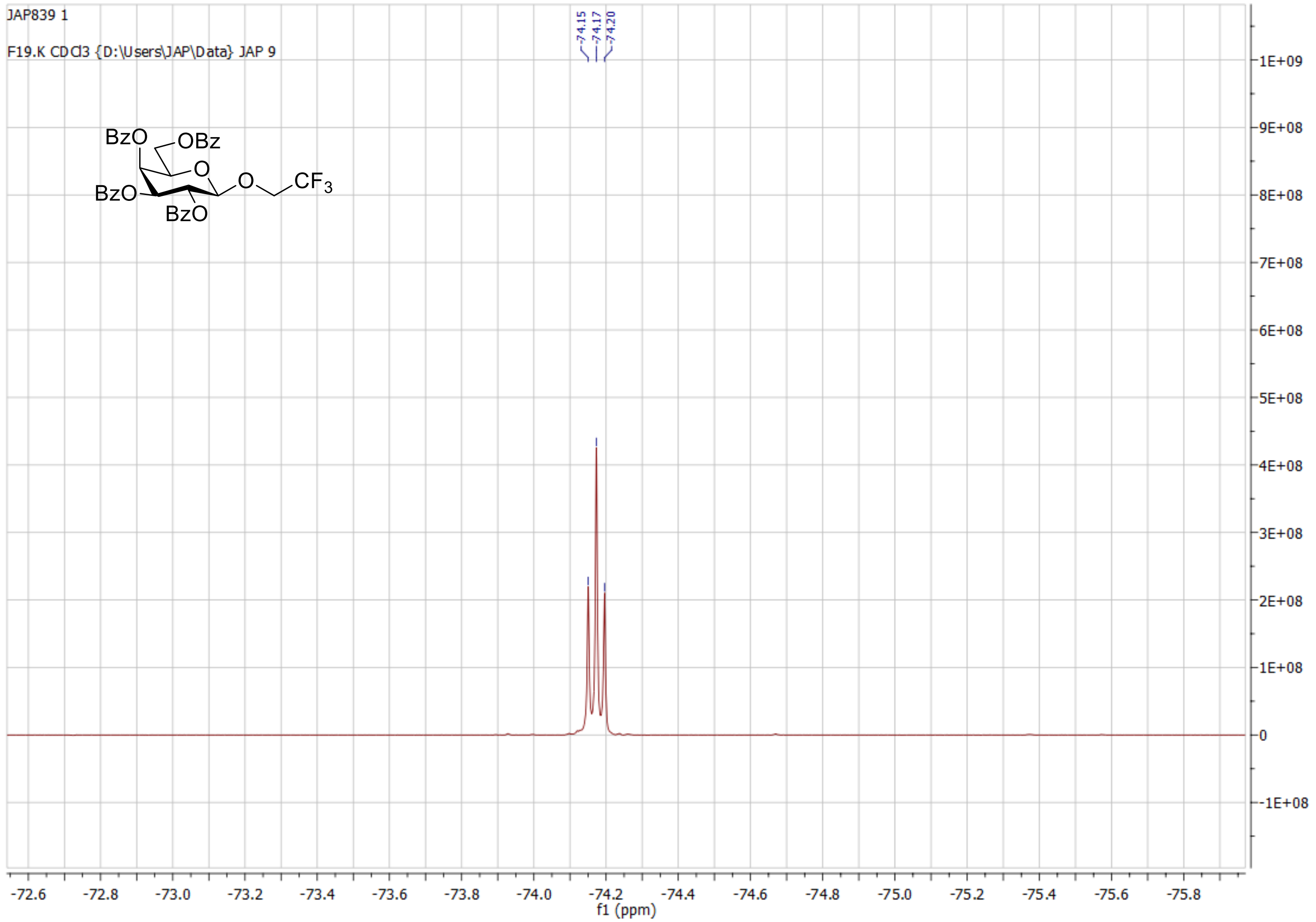

Compound 56

^{1}H NMR (400 MHz, $CDCl_3$): Trifluoroethyl 2,3,6-tri-*O*-benzoyl-β-D-galactopyranoside 56

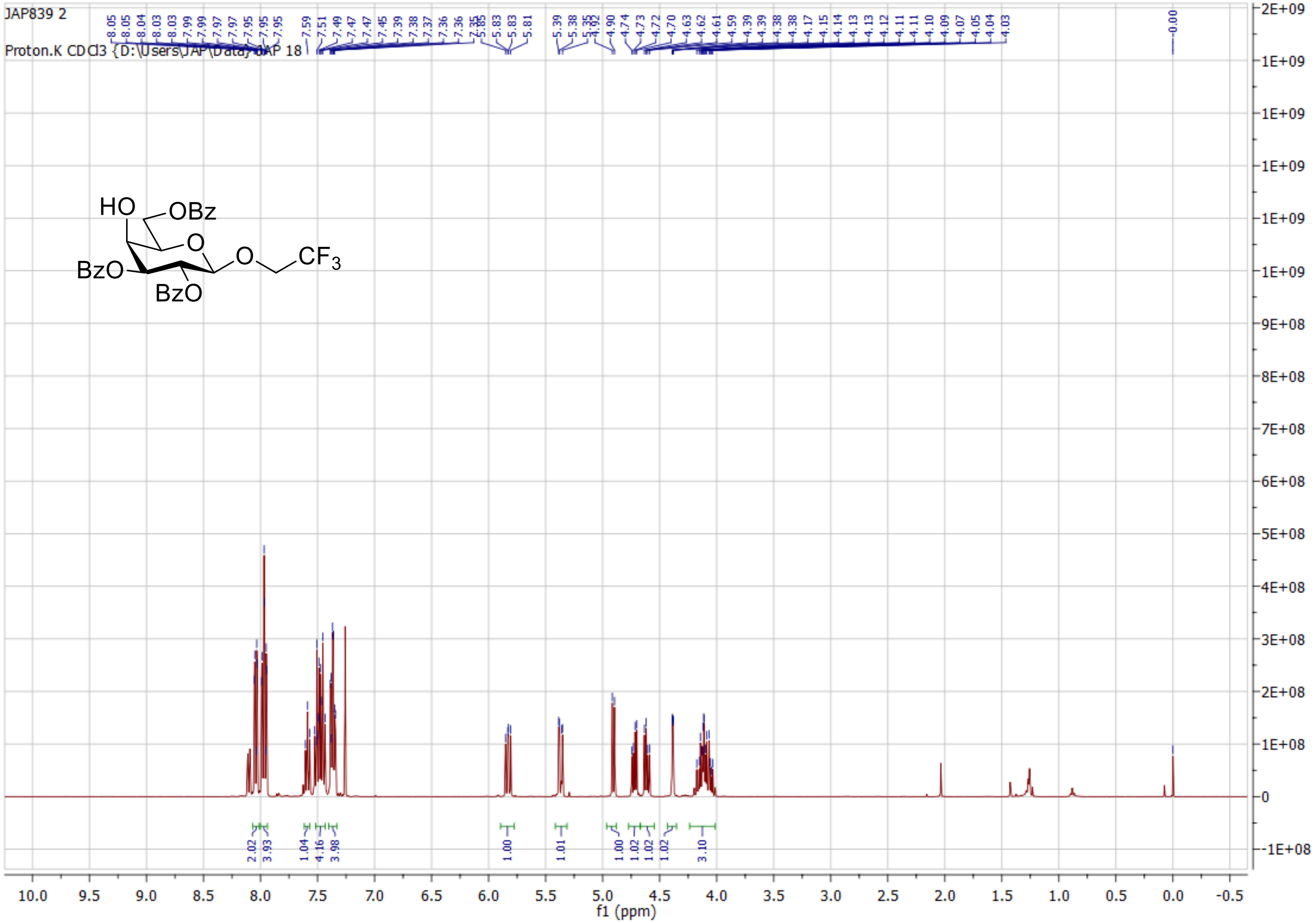

COSY (400 × 400 MHz, $CDCl_3$): Trifluoroethyl 2,3,6-tri-*O*-benzoyl-β-D-galactopyranoside 56

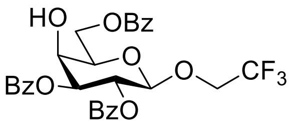

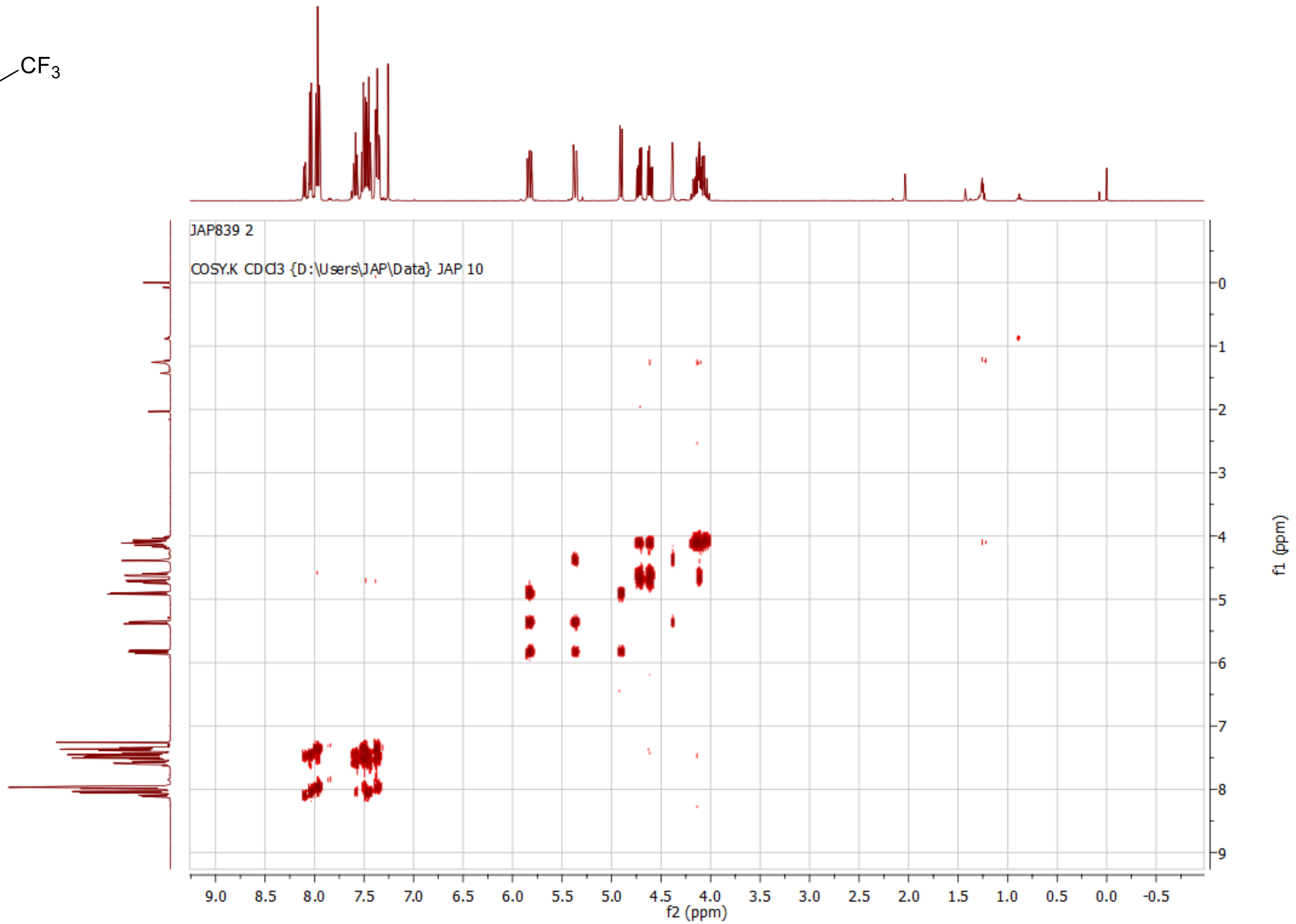

HSQC (400 × 101 MHz, $CDCl_3$): Trifluoroethyl 2,3,6-tri-*O*-benzoyl-β-D-galactopyranoside 56

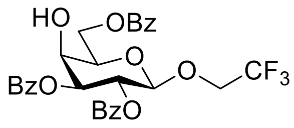

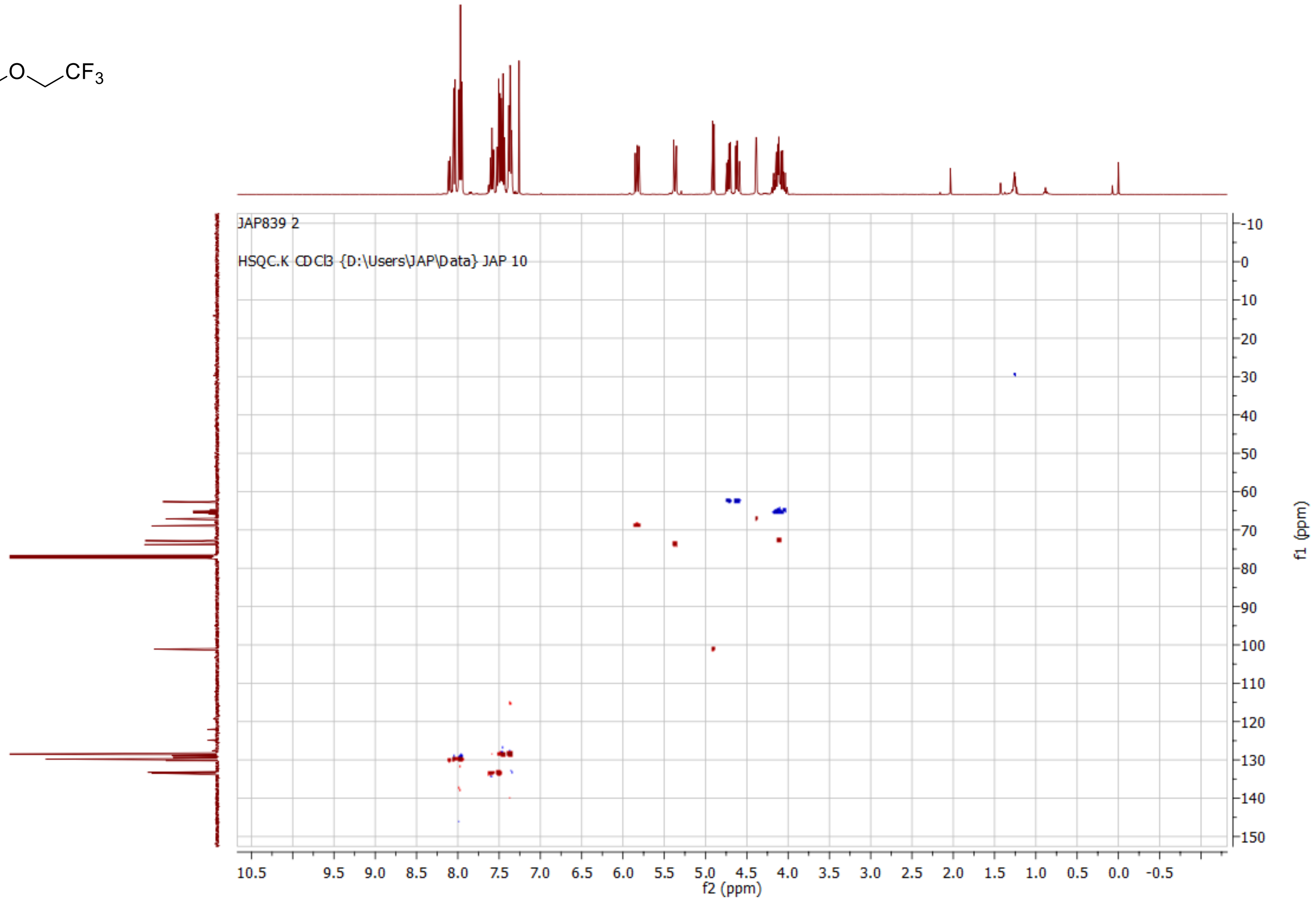

$^{13}C\{1H\}$ NMR (101 MHz, $CDCl_3$): Trifluoroethyl 2,3,6-tri-*O*-benzoyl-β-D-galactopyranoside 56

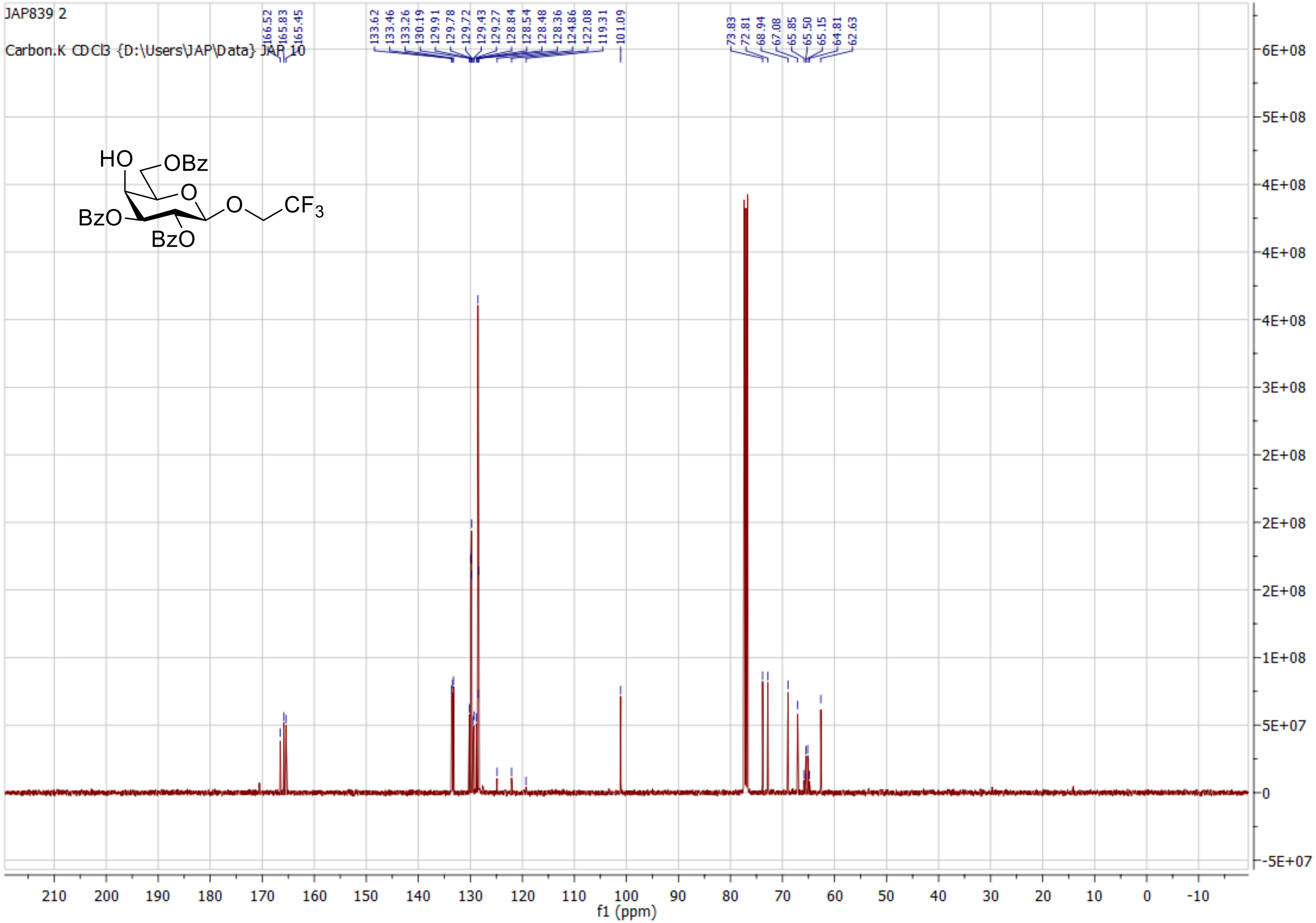

^{19}F NMR (376 MHz, $CDCl_3$): Trifluoroethyl 2,3,6-tri-*O*-benzoyl-β-D-galactopyranoside 56

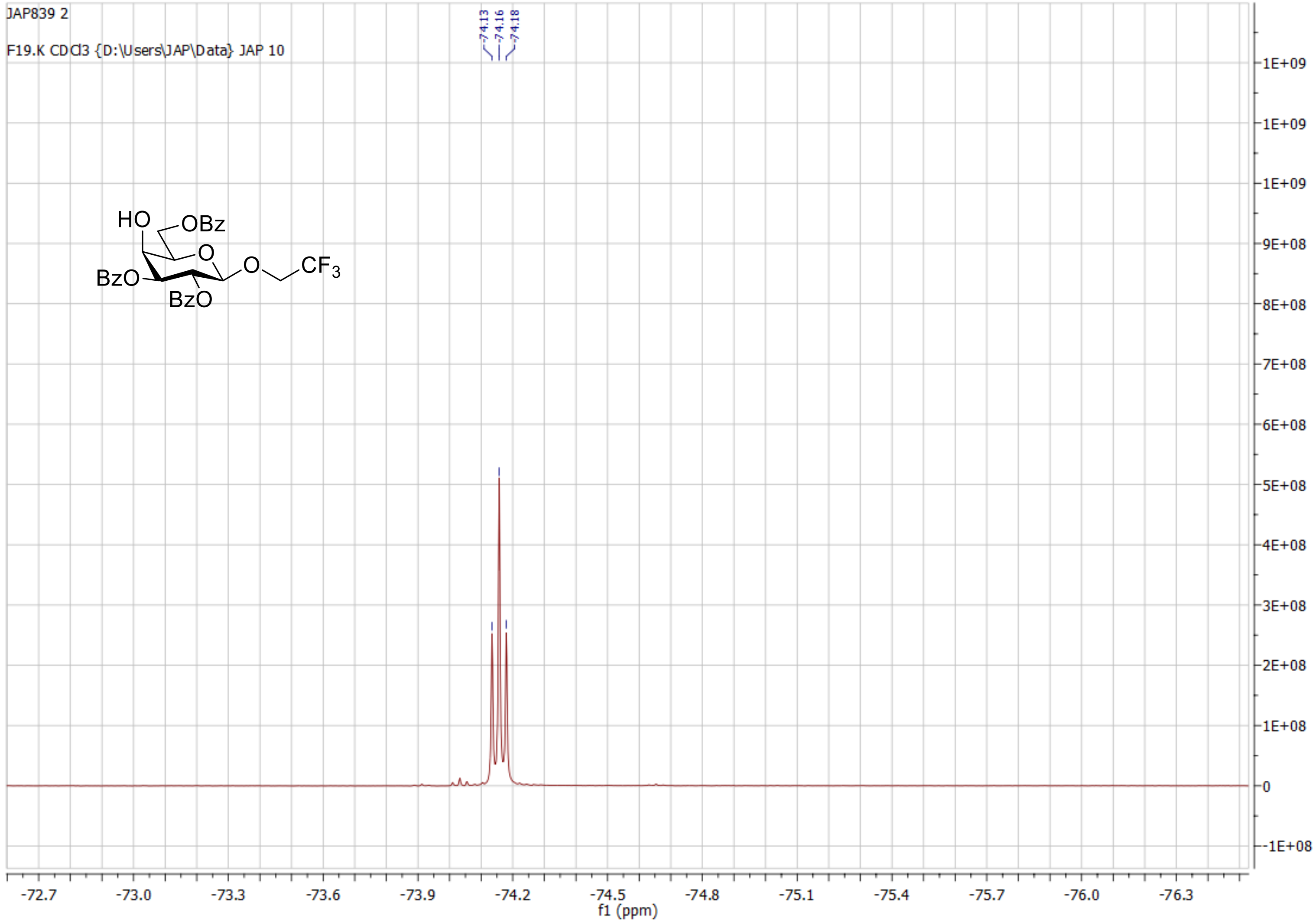

HMBC (400 × 101 MHz, CDCl₃): Trifluoroethyl 2,3,6-tri-*O*-benzoyl-β-D-galactopyranoside 56

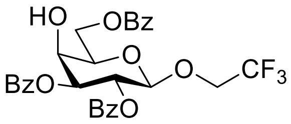

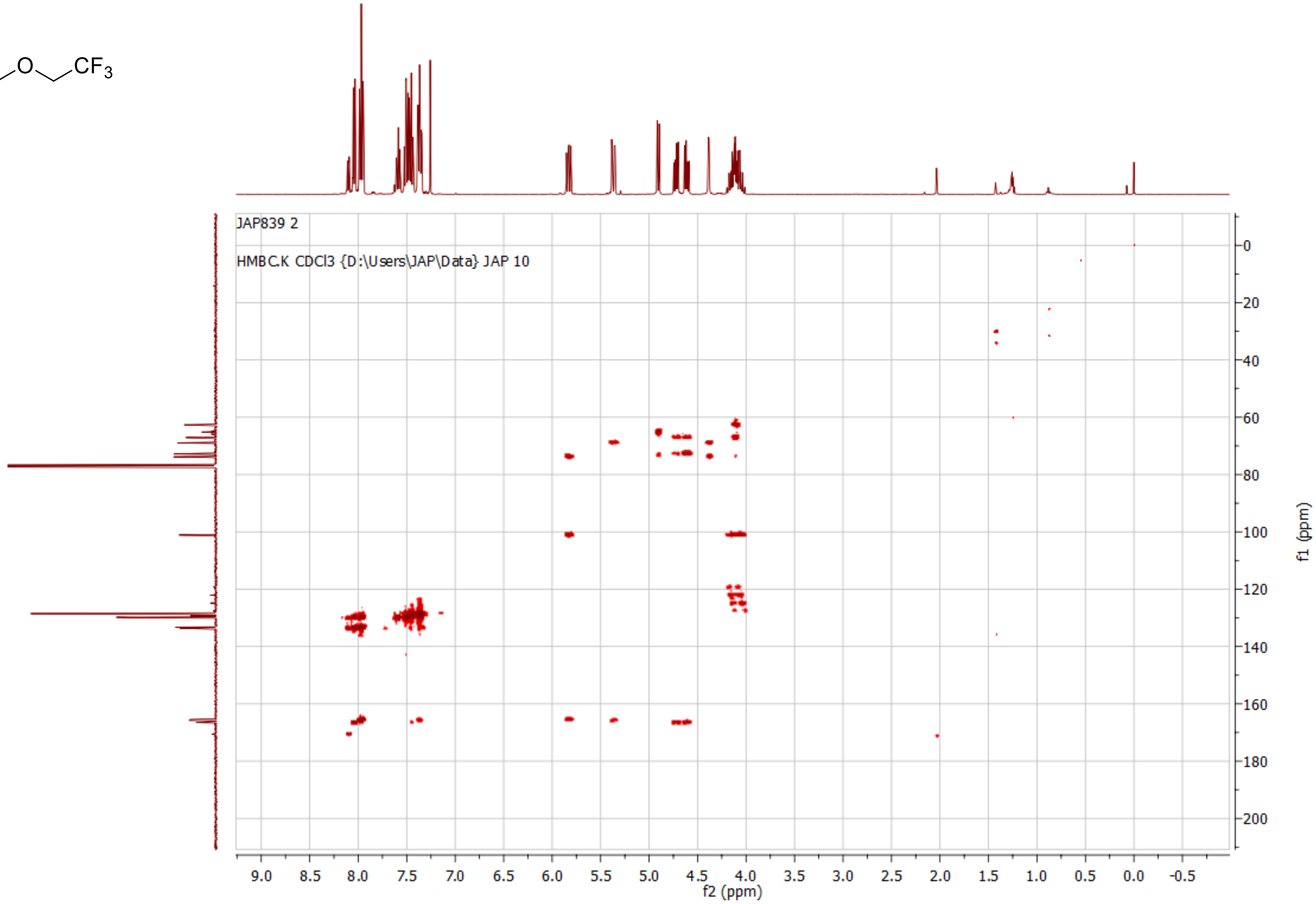

Compound 58

^{1}H NMR (400 MHz, CDCl₃): *p*-(Trifluoromethyl)-phenyl 2,3,4,6-tetra-*O*-benzoyl-1-thio-β-D-galactopyranoside 58

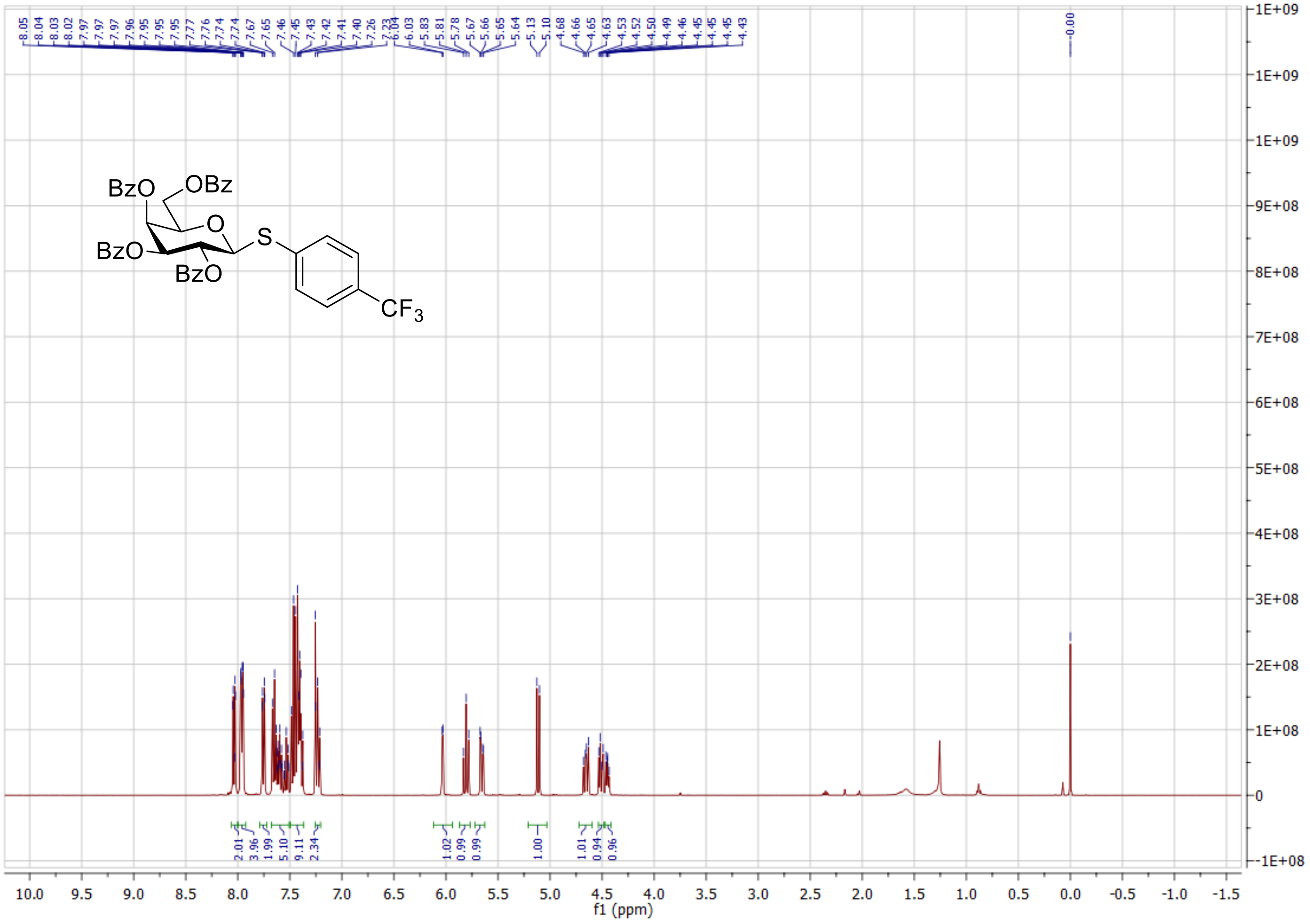

COSY (400 × 400 MHz, $CDCl_3$): *p*-(Trifluoromethyl)-phenyl 2,3,4,6-tetra-*O*-benzoyl-1-thio-β-D-galactopyranoside 58

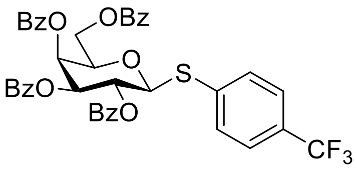

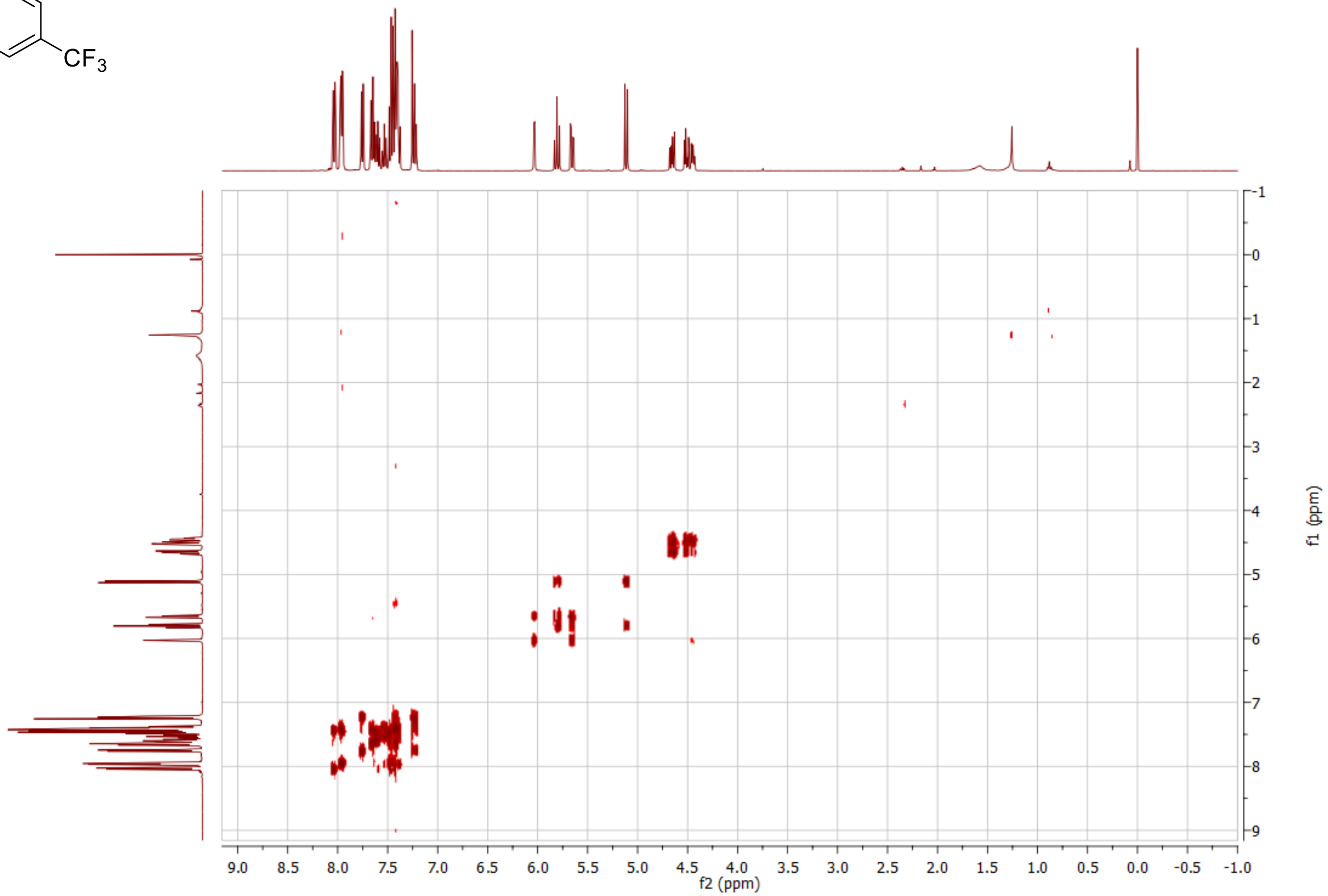

HSQC (400 × 101 MHz, CDCl₃): *p*-(Trifluoromethyl)-phenyl 2,3,4,6-tetra-*O*-benzoyl-1-thio-β-D-galactopyranoside 58

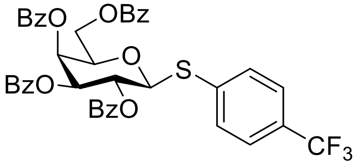

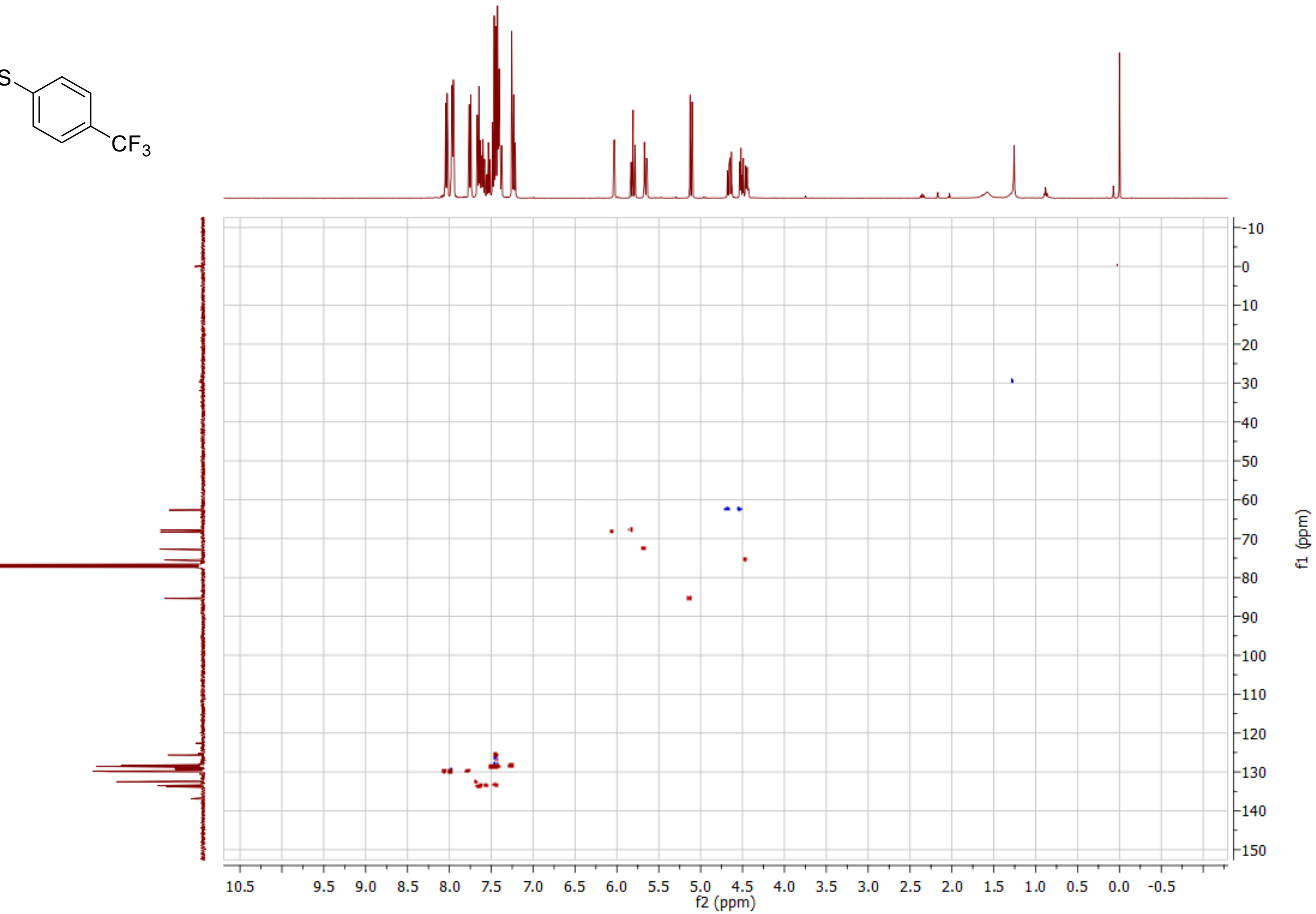

$^{13}C\{1H\}$ NMR (101 MHz, $CDCl_3$): *p*-(Trifluoromethyl)-phenyl 2,3,4,6-tetra-*O*-benzoyl-1-thio-β-D-galactopyranoside 58

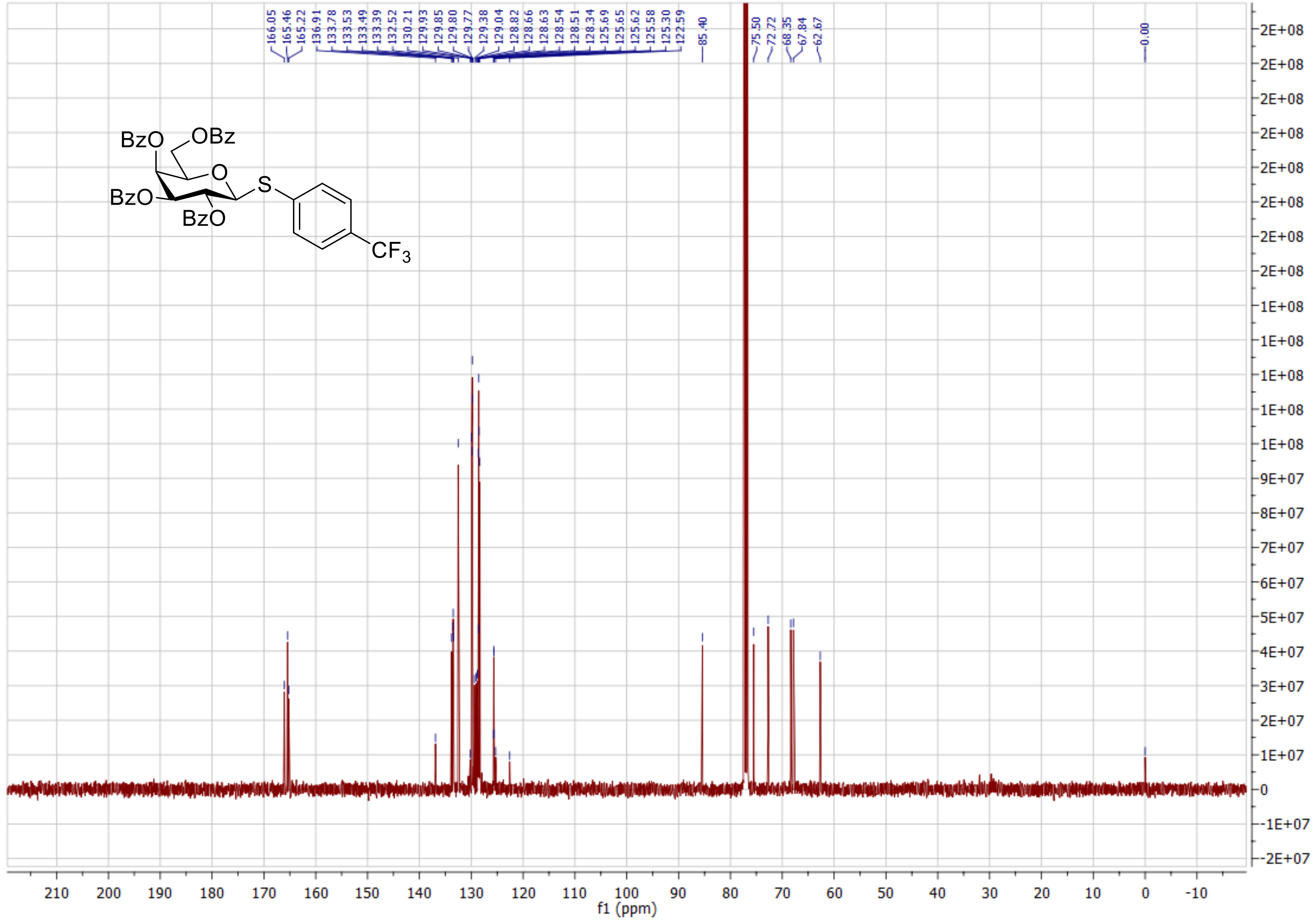

^{19}F NMR (376 MHz, CDCl$_3$): *p*-(Trifluoromethyl)-phenyl 2,3,4,6-tetra-*O*-benzoyl-1-thio-β-D-galactopyranoside 58

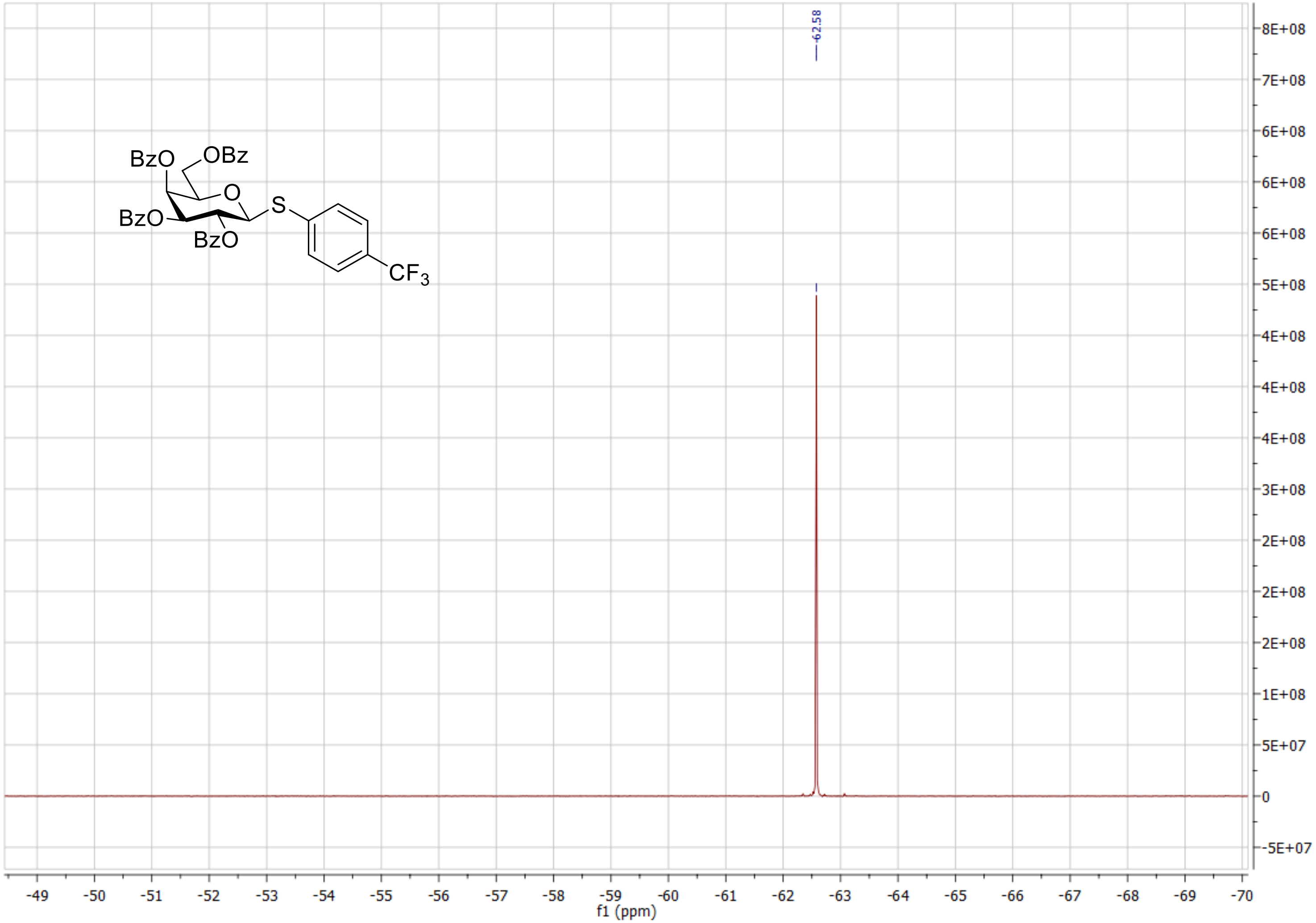

Compound 59/60

^{1}H NMR (400 MHz, CDCl₃): *p*-(Trifluoromethyl)-phenyl 3,4,6-tri-*O*-benzoyl-1-thio-β-D-galactopyranoside 59 & *p*-(trifluoromethyl)-phenyl 2,3,6-tri-*O*-benzoyl-1-thio-β-D-galactopyranoside 60

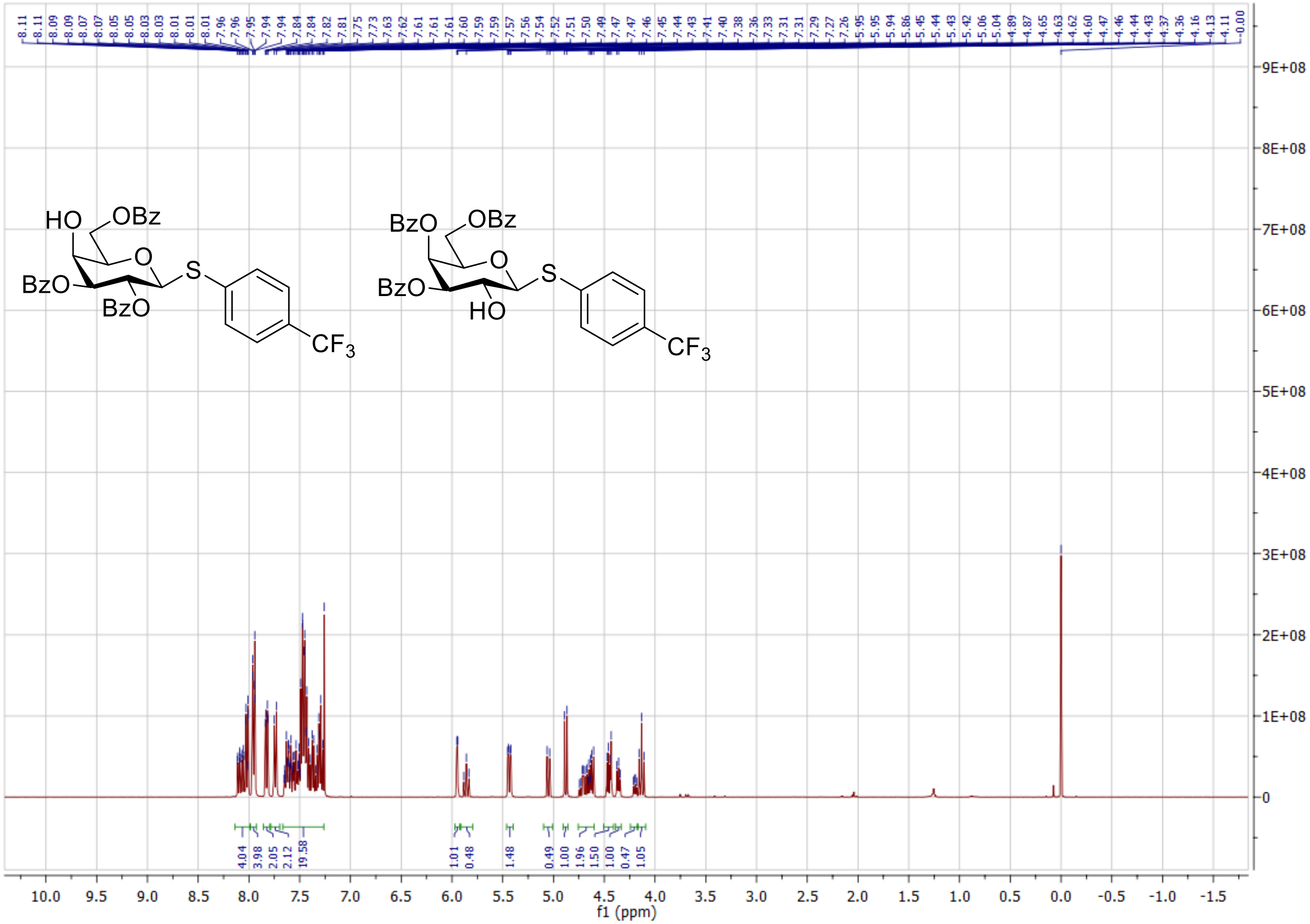

COSY (400 × 400 MHz, CDCl3): *p*-(Trifluoromethyl)-phenyl 3,4,6-tri-*O*-benzoyl-1-thio-β-D-galactopyranoside 59 & *p*-(trifluoromethyl)-phenyl 2,3,6-tri-*O*-benzoyl-1-thio-β-D-galactopyranoside 60

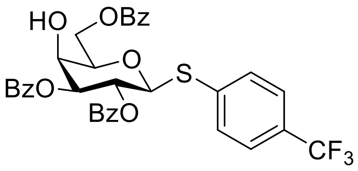

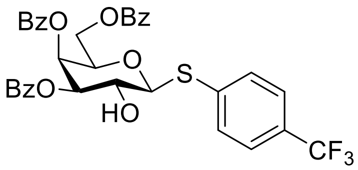

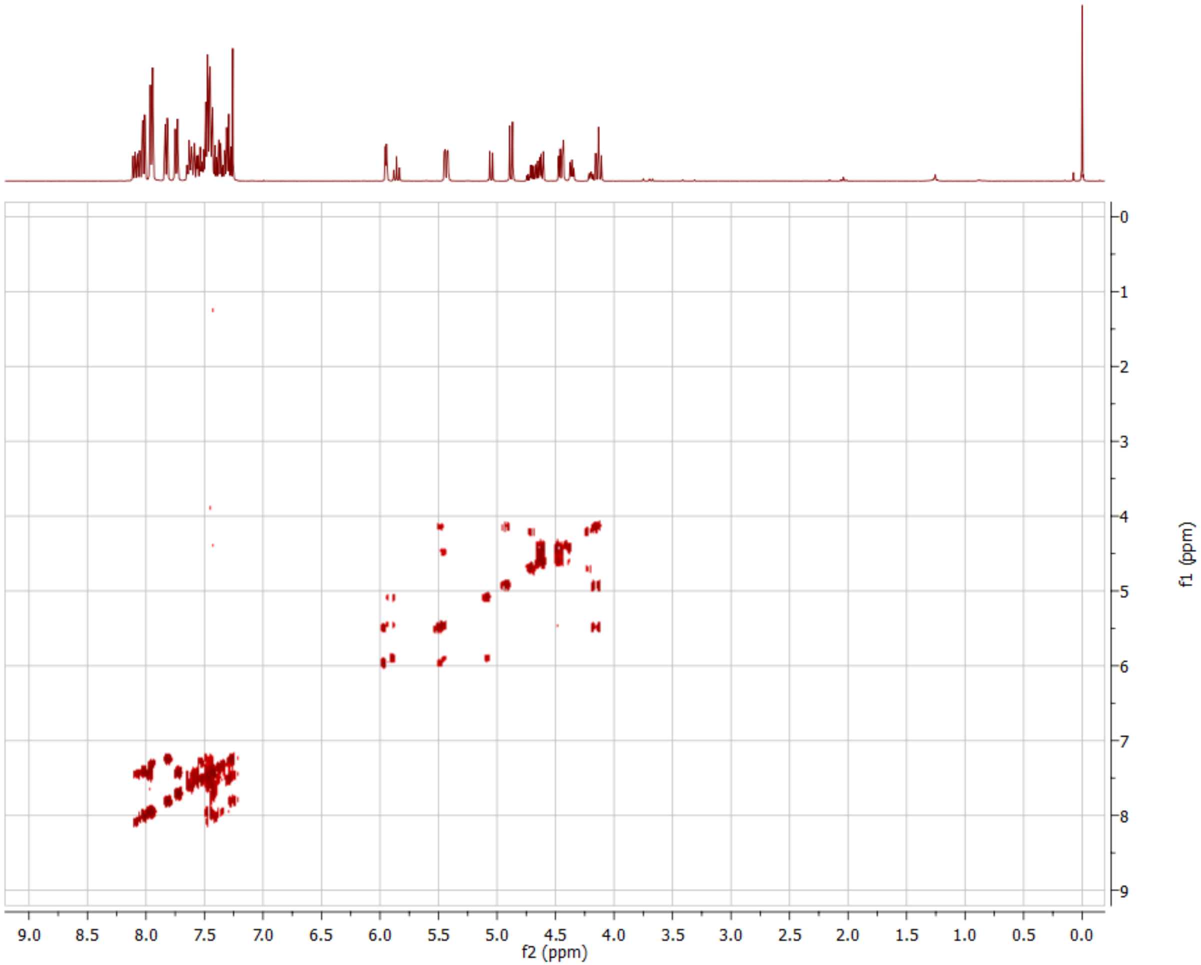

HSQC (400 × 101 MHz, $CDCl_3$): *p*-(Trifluoromethyl)-phenyl 3,4,6-tri-*O*-benzoyl-1-thio-β-D-galactopyranoside 59 & *p*-(trifluoromethyl)-phenyl 2,3,6-tri-*O*-benzoyl-1-thio-β-D-galactopyranoside 60

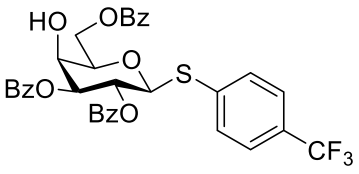

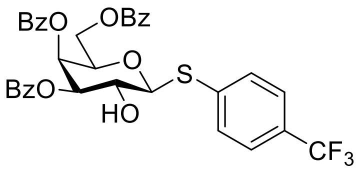

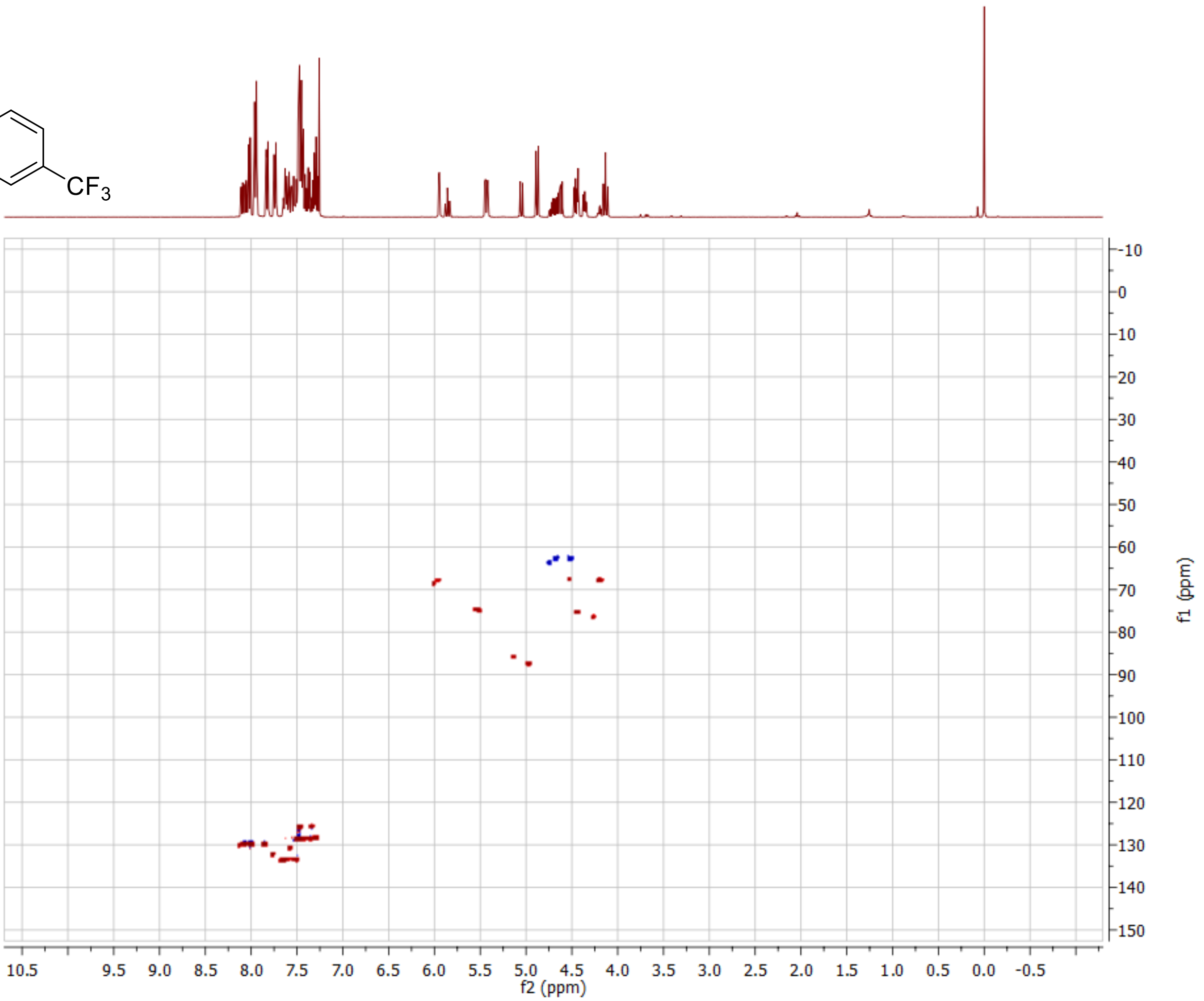

^{13}C{1H} NMR (101 MHz, CDCl₃): *p*-(Trifluoromethyl)-phenyl 3,4,6-tri-*O*-benzoyl-1-thio-β-D-galactopyranoside 59 & *p*-(trifluoromethyl)-phenyl 2,3,6-tri-*O*-benzoyl-1-thio-β-D-galactopyranoside 60

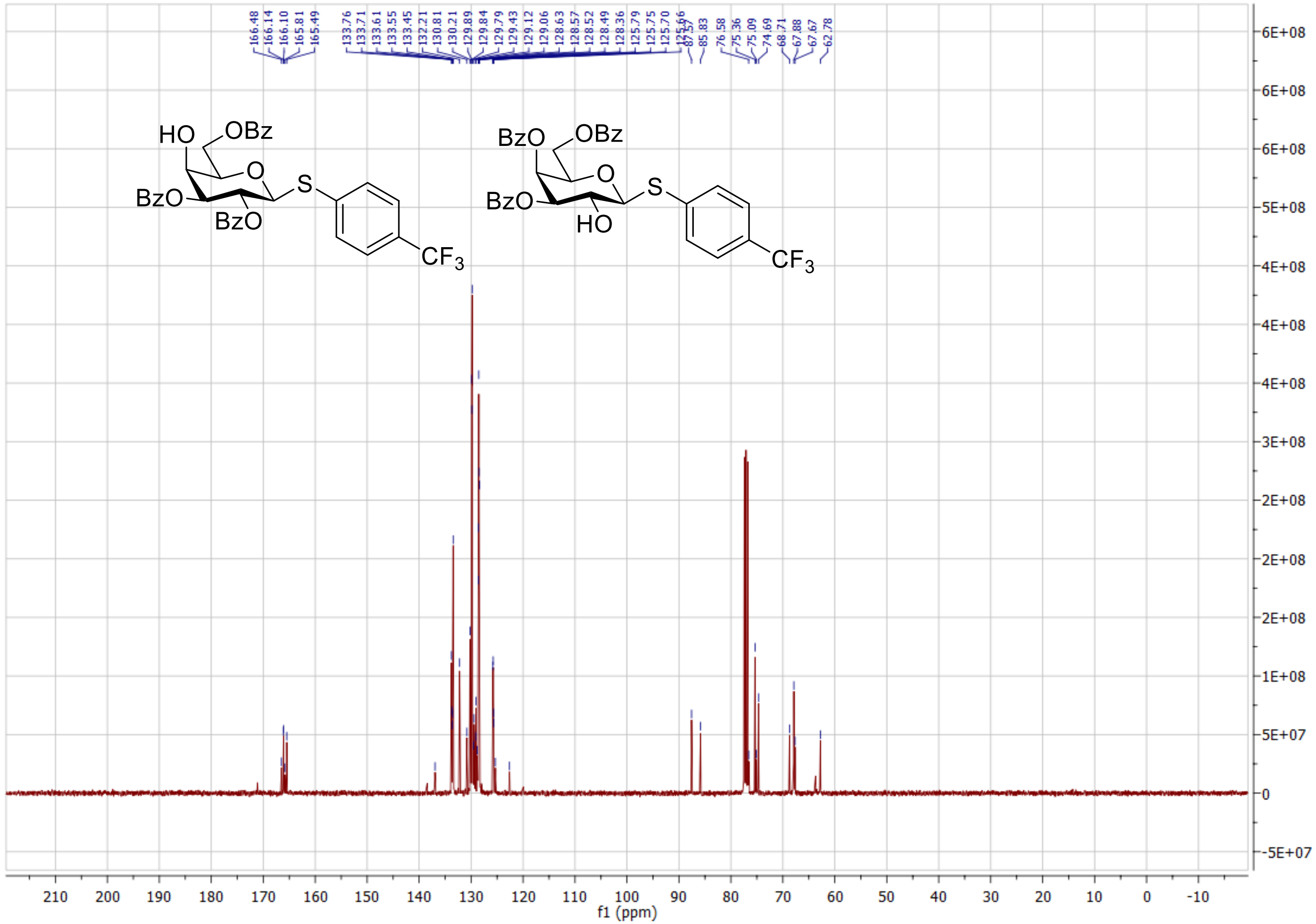

HMBC (400 × 101 MHz, CDCl₃): *p*-(trifluoromethyl)-phenyl 3,4,6-tri-*O*-benzoyl-1-thio-β-D-galactopyranoside 59 & *p*-(trifluoromethyl)-phenyl 2,3,6-tri-*O*-benzoyl-1-thio-β-D-galactopyranoside 60

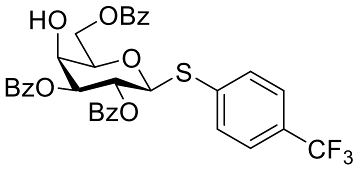

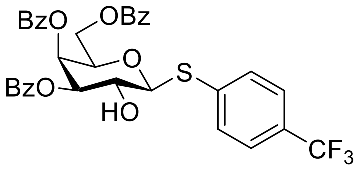

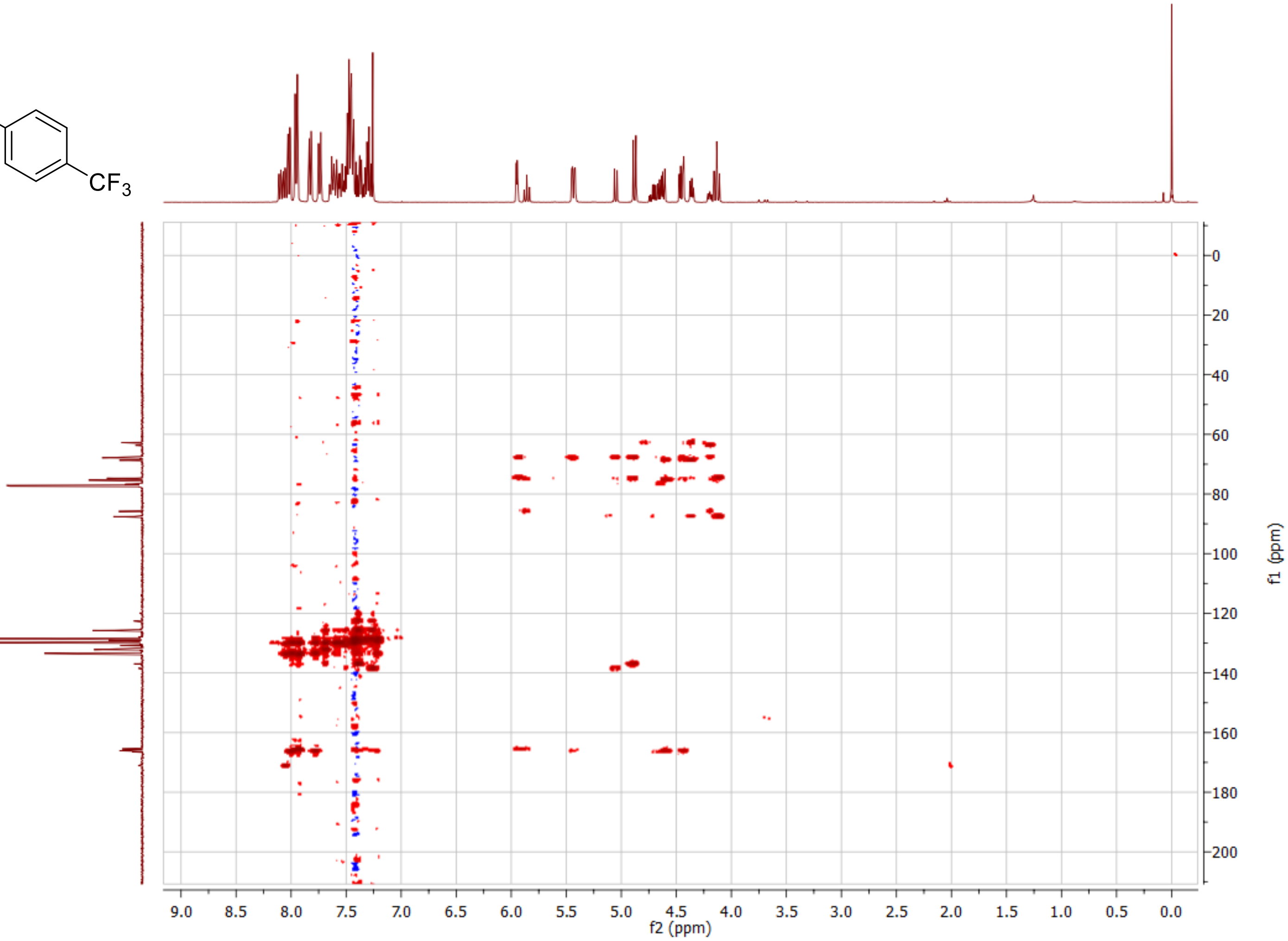

^{19}F NMR (376 MHz, $CDCl_3$): *p*-(Trifluoromethyl)-phenyl 3,4,6-tri-*O*-benzoyl-1-thio-β-D-galactopyranoside 59 & *p*-(trifluoromethyl)-phenyl 2,3,6-tri-*O*-benzoyl-1-thio-β-D-galactopyranoside 60

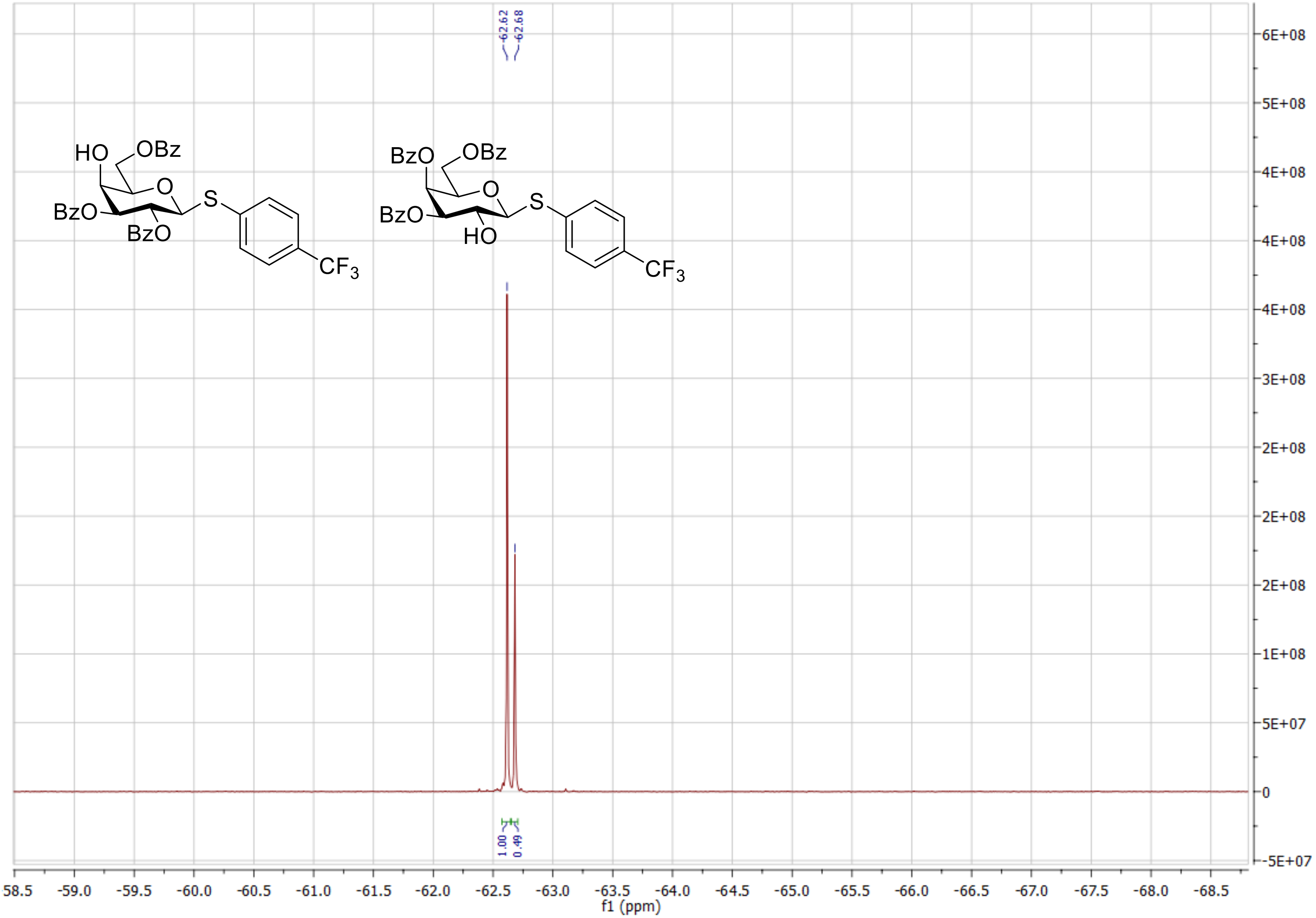

Compound 61

^{1}H NMR (400 MHz, Acetone-d6): *p*-(Trifluoromethyl)-phenyl 3,6-di-*O*-benzoyl-1-thio-β-D-galactopyranoside 61

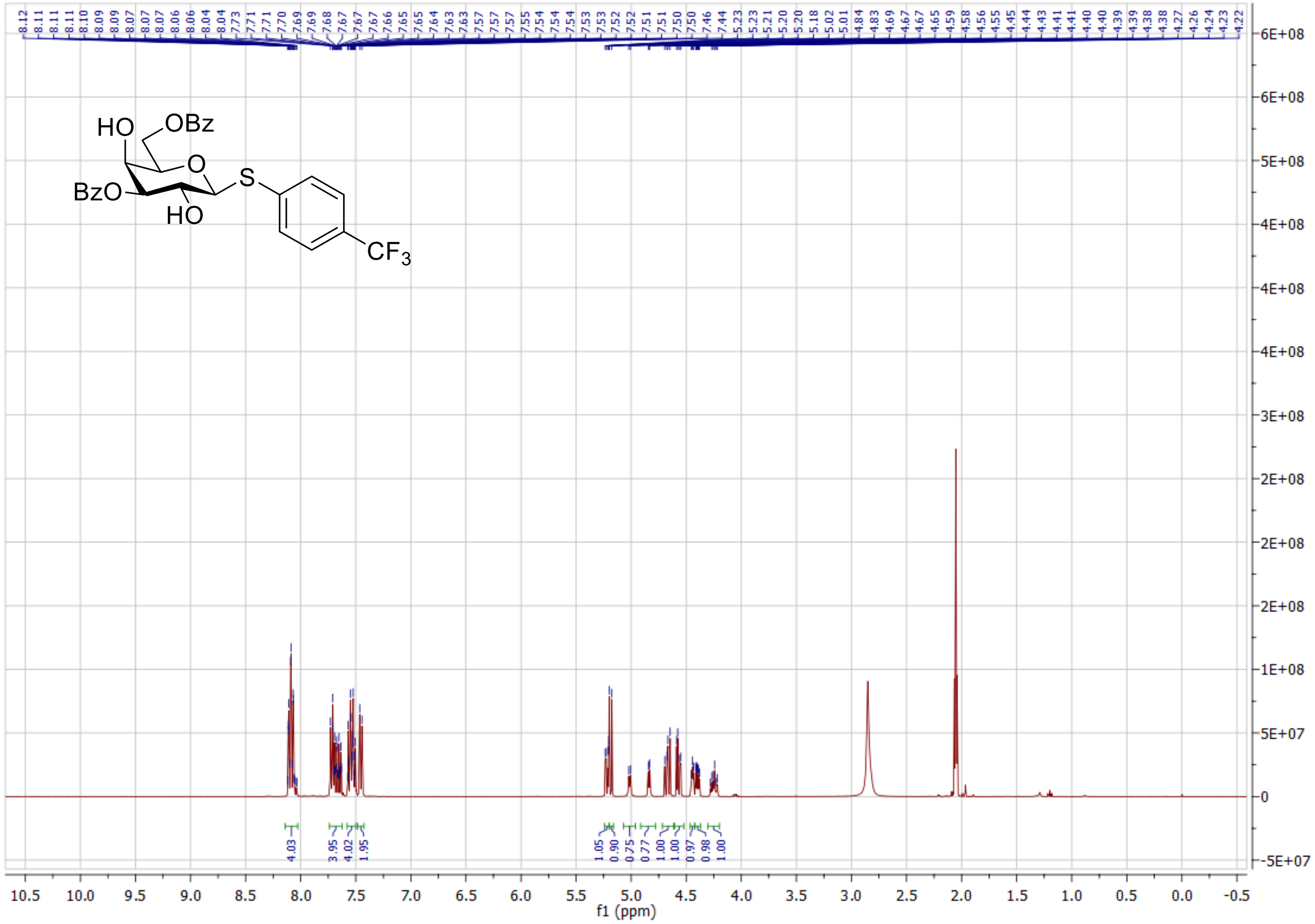

COSY (400 × 400 MHz, Acetone-d6): *p*-(Trifluoromethyl)-phenyl 3,6-di-*O*-benzoyl-1-thio-β-D-galactopyranoside 61

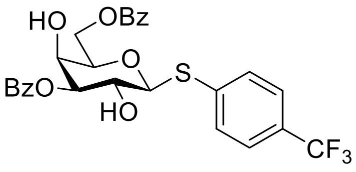

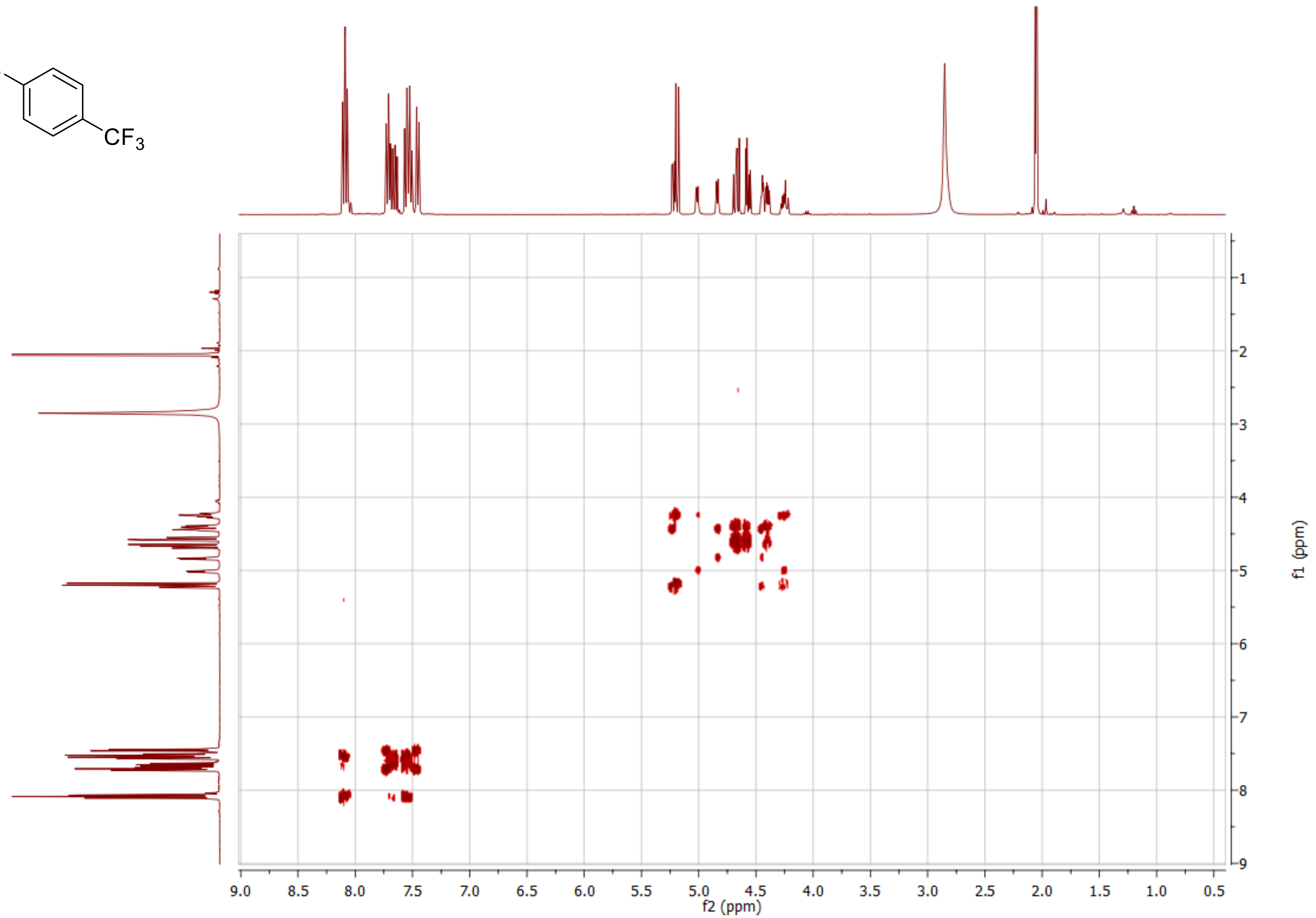

HSQC (400 × 101 MHz, Acetone-d6): *p*-(Trifluoromethyl)-phenyl 3,6-di-*O*-benzoyl-1-thio-β-D-galactopyranoside 61

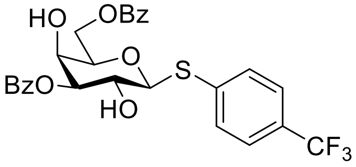

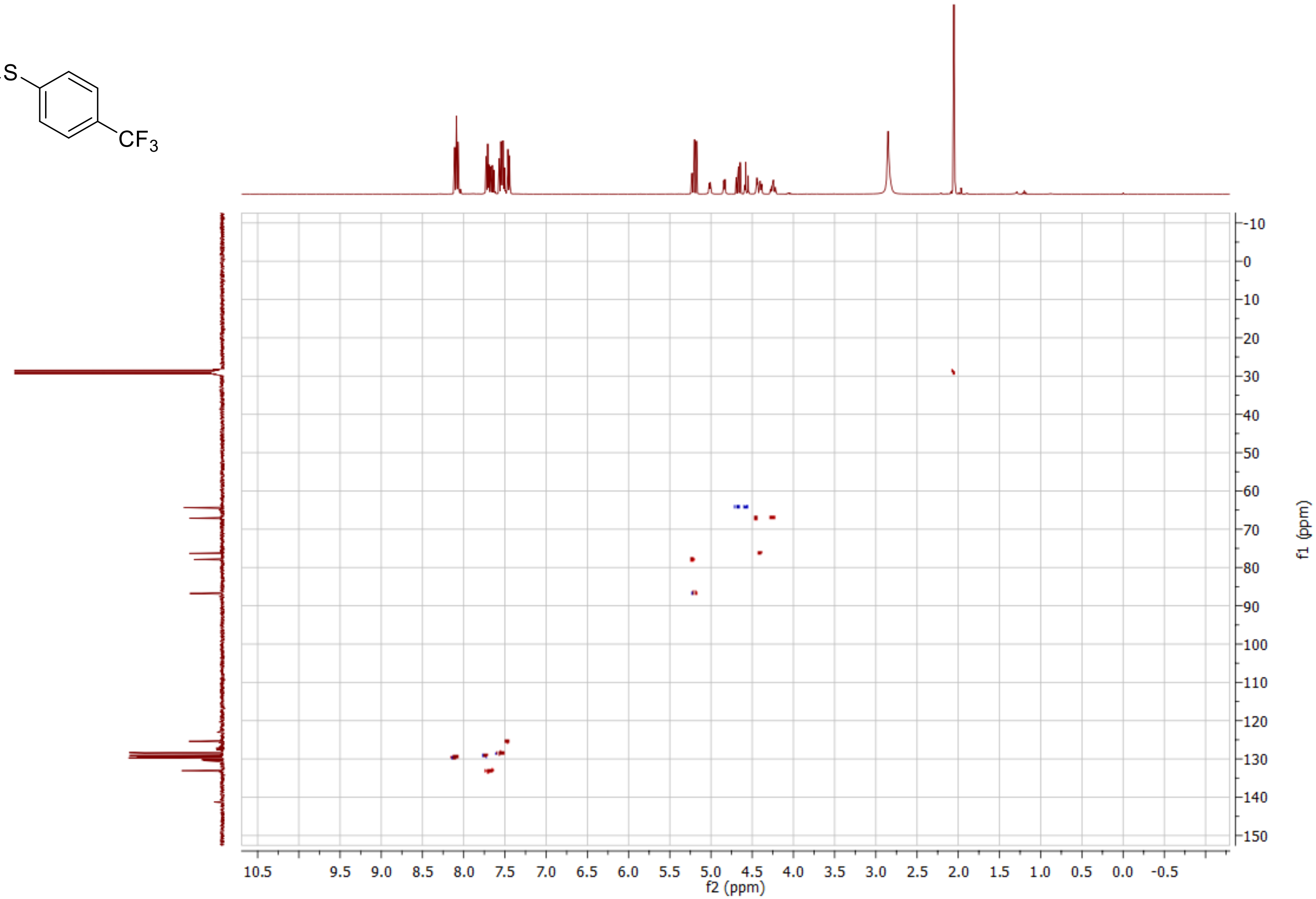

$^{13}C\{1H\}$ NMR (101 MHz, Acetone-d6): *p*-(Trifluoromethyl)-phenyl 3,6-di-*O*-benzoyl-1-thio-β-D-galactopyranoside 61

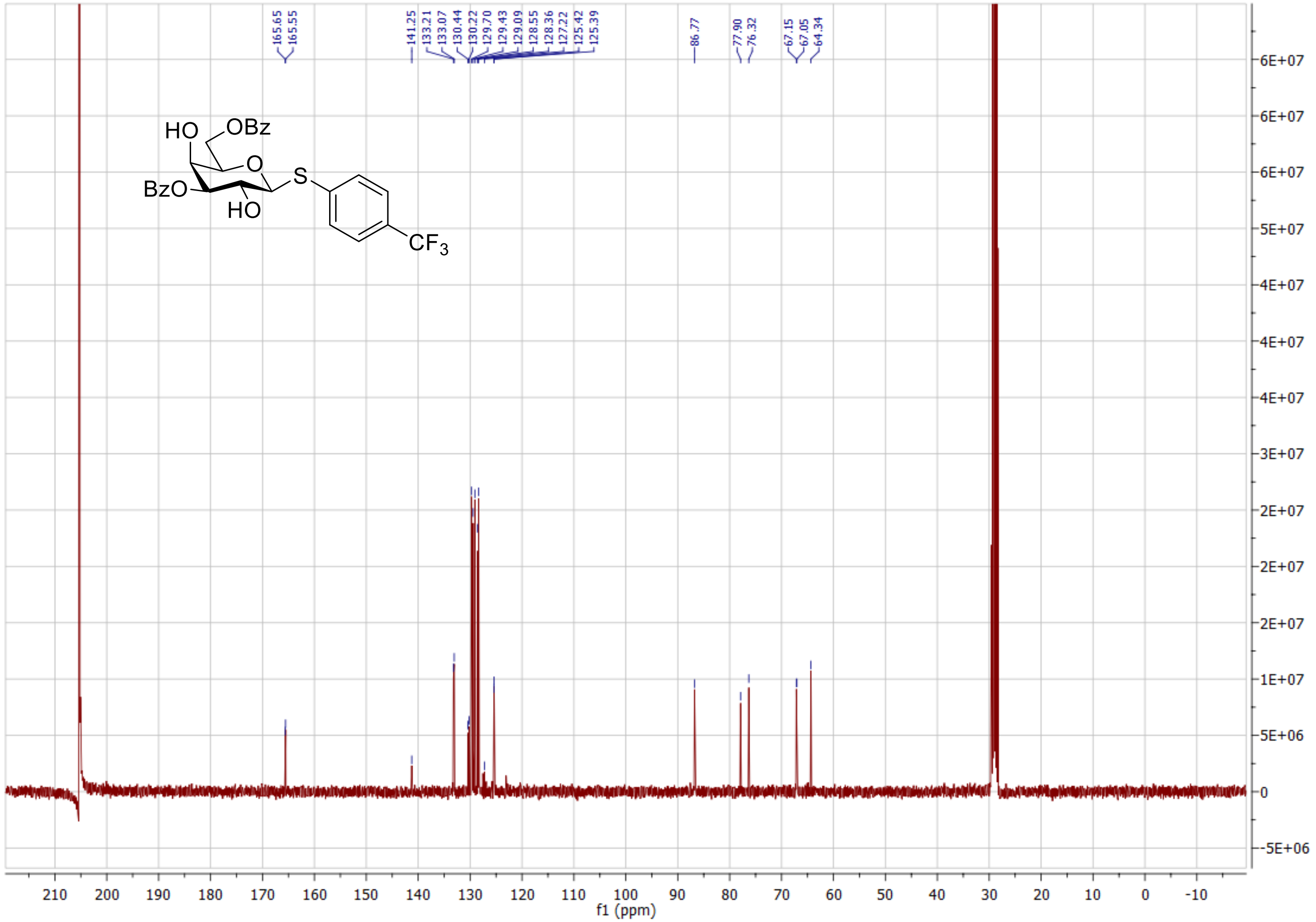

^{19}F NMR (376 MHz, Acetone-d6): *p*-(Trifluoromethyl)-phenyl 3,6-di-*O*-benzoyl-1-thio-β-D-galactopyranoside 61

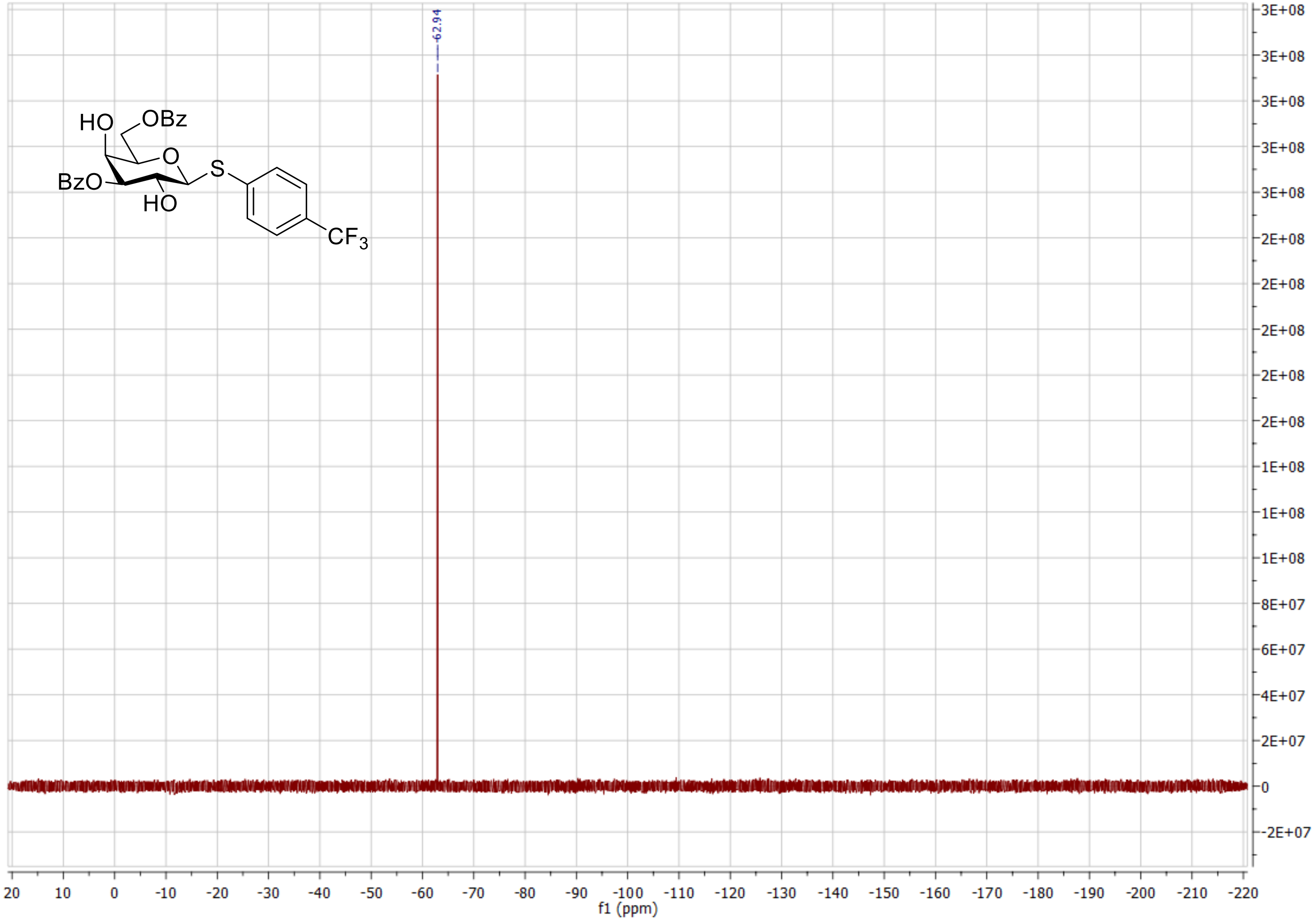

Compound 63

^{1}H NMR (400 MHz, CDCl$_3$): *p*-(Methoxy)-phenyl 2,3,4,6-tetra-*O*-benzoyl-1-thio-β-D-galactopyranoside 63

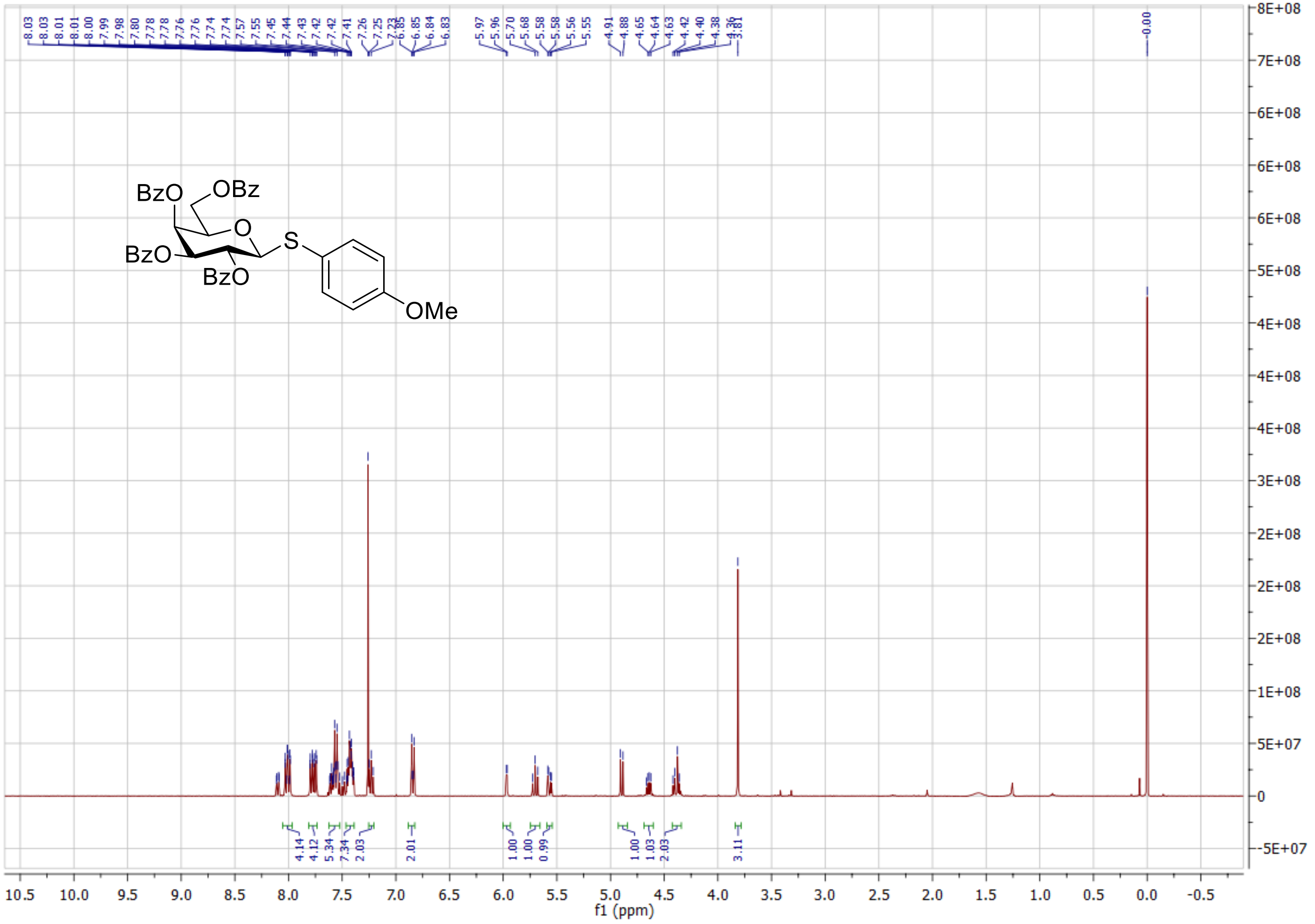

COSY (400 × 400 MHz, CDCl$_3$): *p*-(Methoxy)-phenyl 2,3,4,6-tetra-*O*-benzoyl-1-thio-β-D-galactopyranoside 63

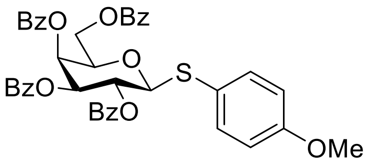

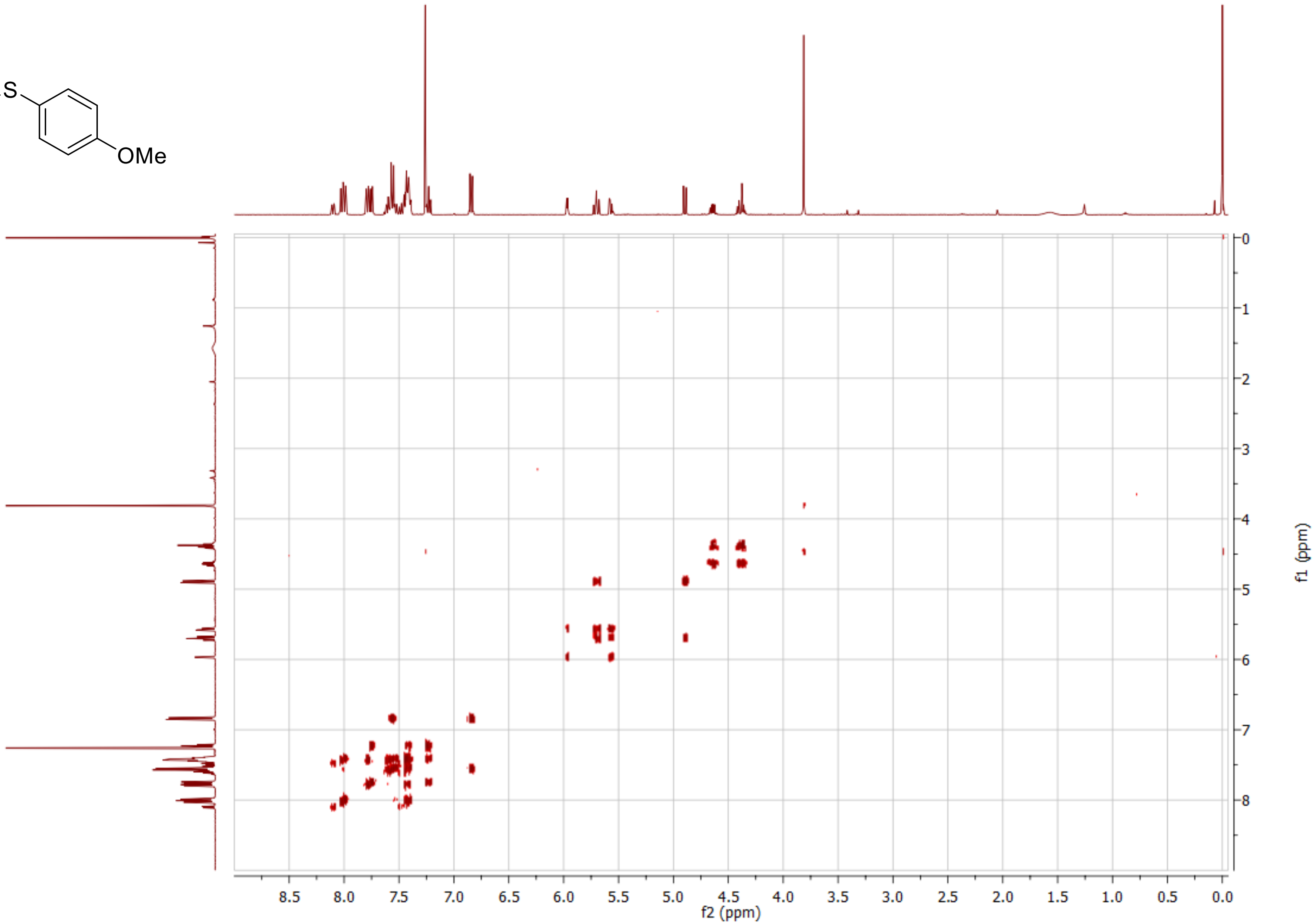

HSQC (400 × 101 MHz, $CDCl_3$): *p*-(Methoxy)-phenyl 2,3,4,6-tetra-*O*-benzoyl-1-thio-β-D-galactopyranoside 63

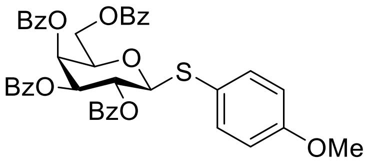

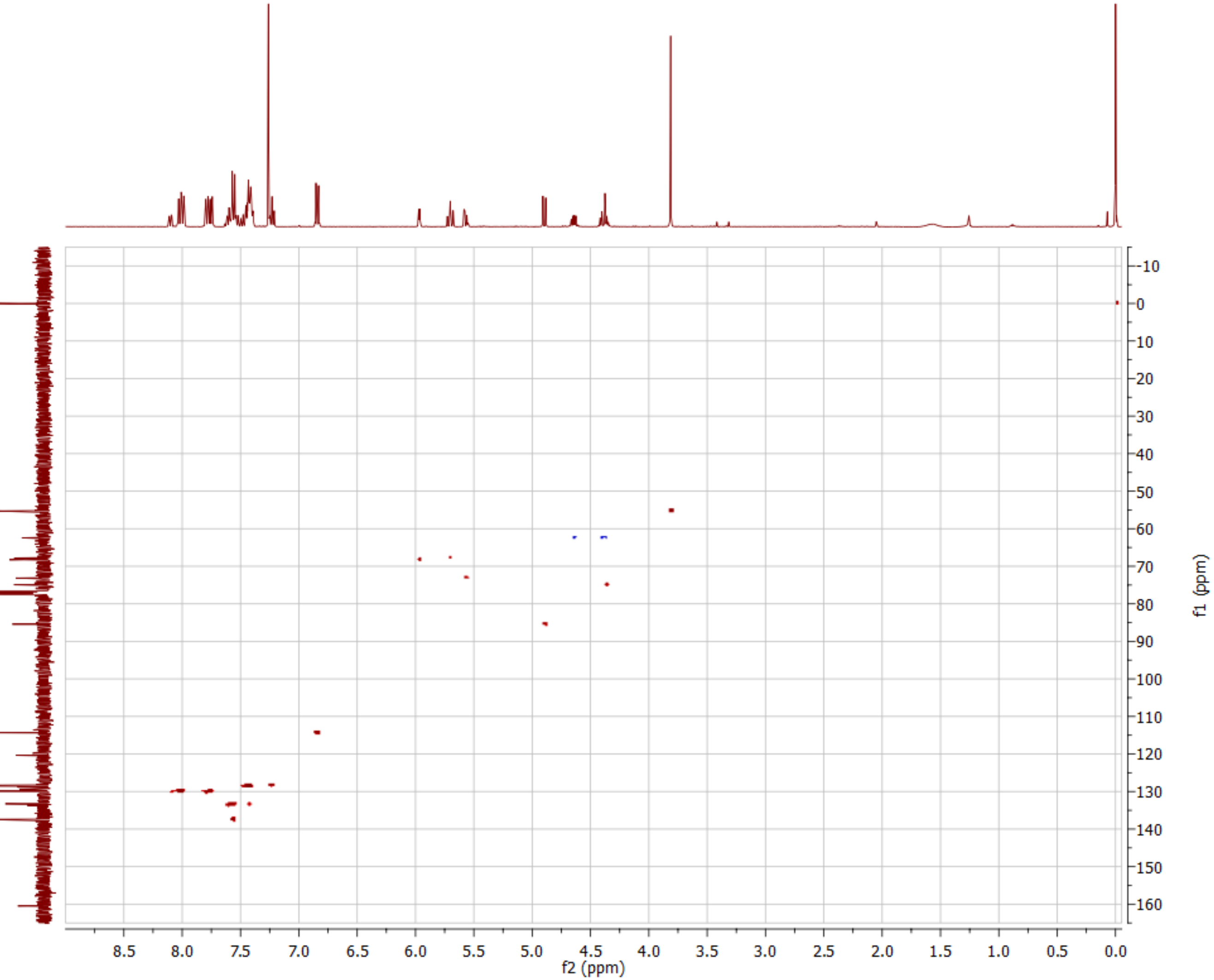

$^{13}C\{1H\}$ NMR (101 MHz, $CDCl_3$): *p*-(Methoxy)-phenyl 2,3,4,6-tetra-*O*-benzoyl-1-thio-β-D-galactopyranoside 63

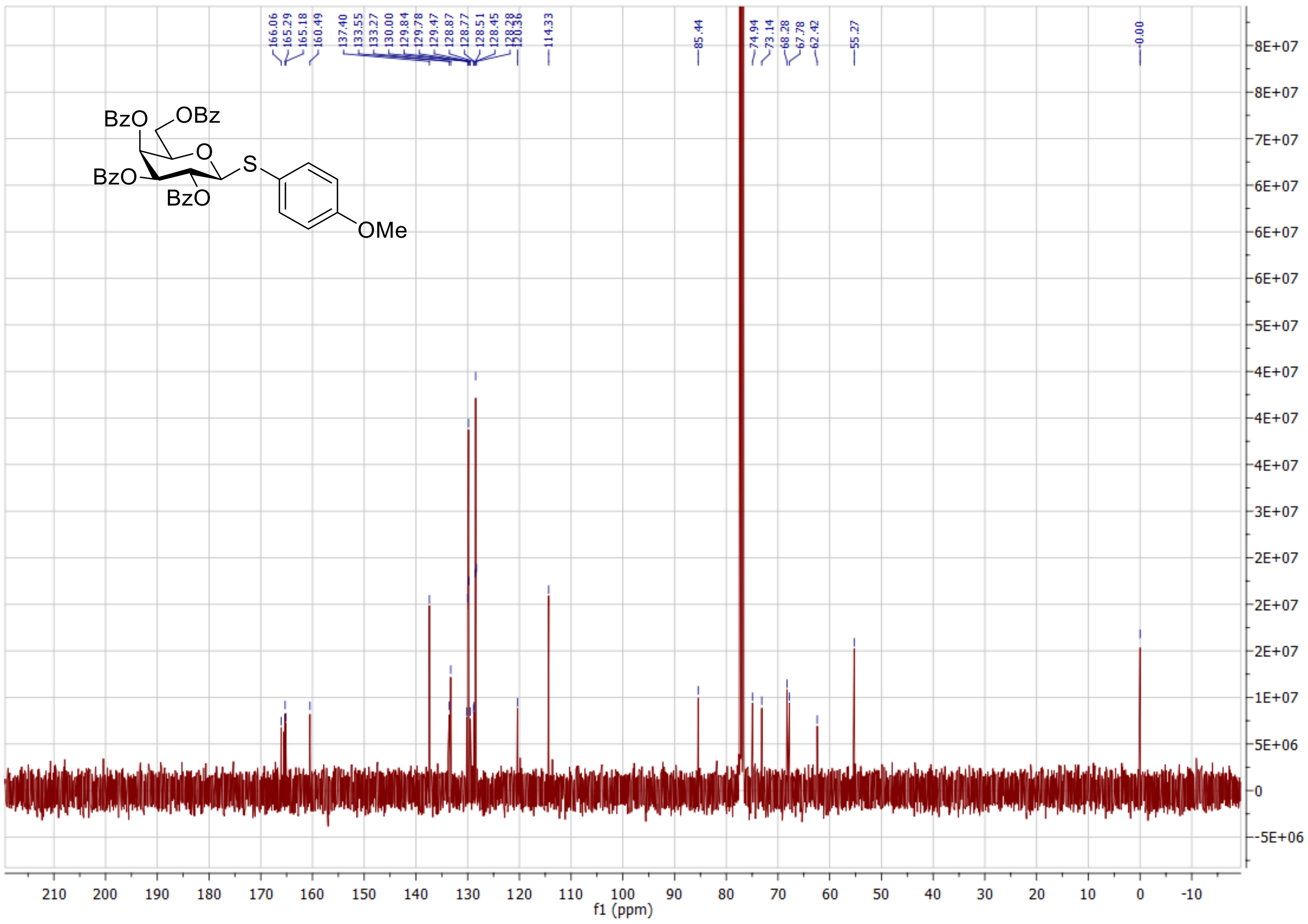

Compound 64/65

^{1}H NMR (400 MHz, $CDCl_3$): *p*-(Methoxy)-phenyl 3,4,6-tri-*O*-benzoyl-1-thio-β-D-galactopyranoside 64 & *p*-(methoxy)-phenyl 2,3,6-tri-*O*-benzoyl-1-thio-β-D-galactopyranoside 65

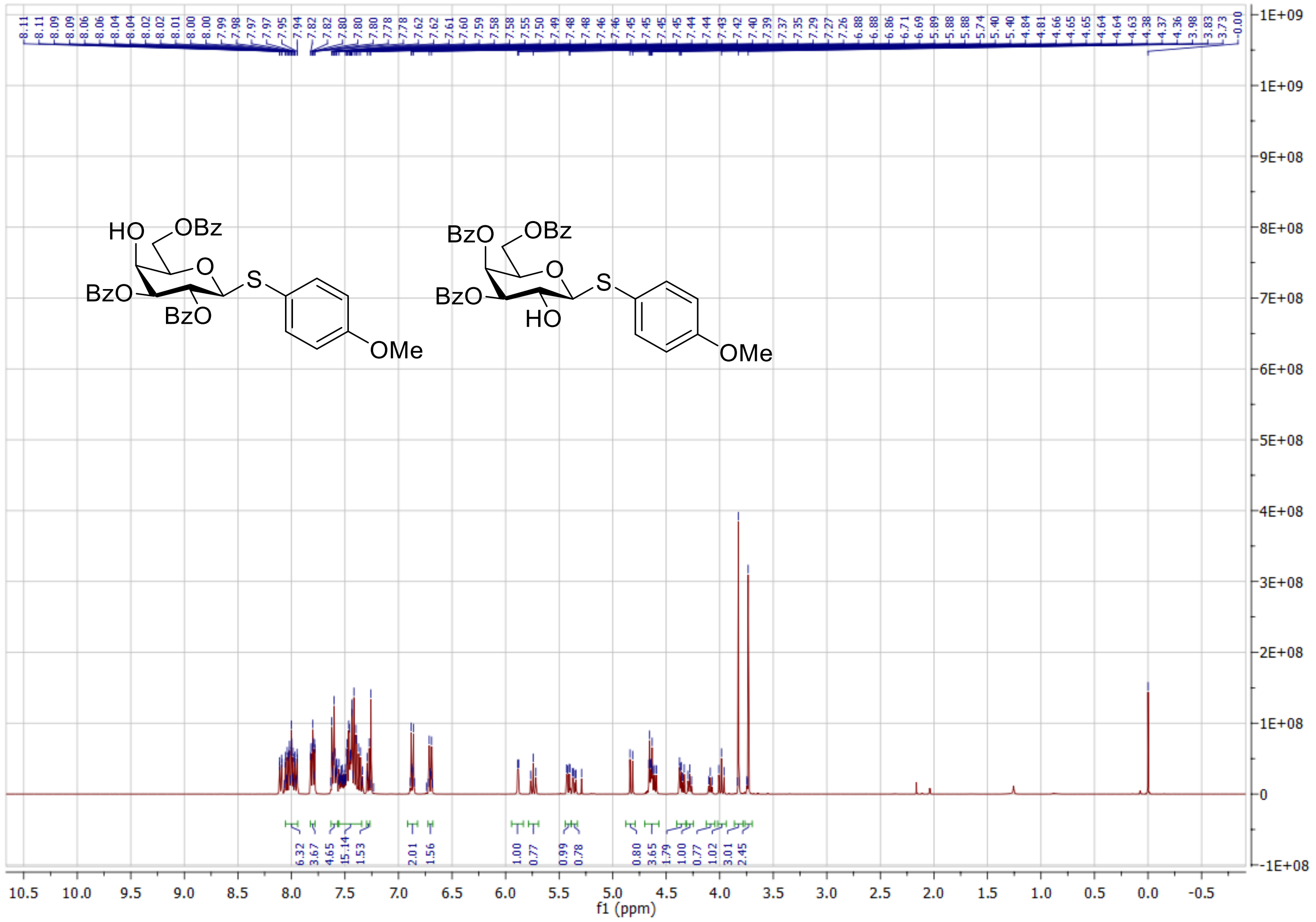

COSY (400 × 400 MHz, $CDCl_3$): *p*-(Methoxy)-phenyl 3,4,6-tri-*O*-benzoyl-1-thio-β-D-galactopyranoside 64 & *p*-(methoxy)-phenyl 2,3,6-tri-*O*-benzoyl-1-thio-β-D-galactopyranoside 65

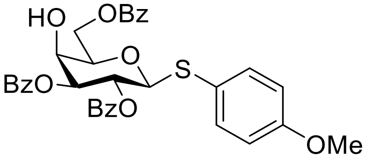

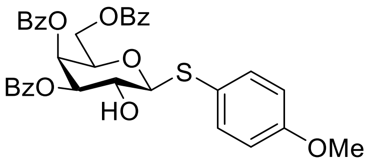

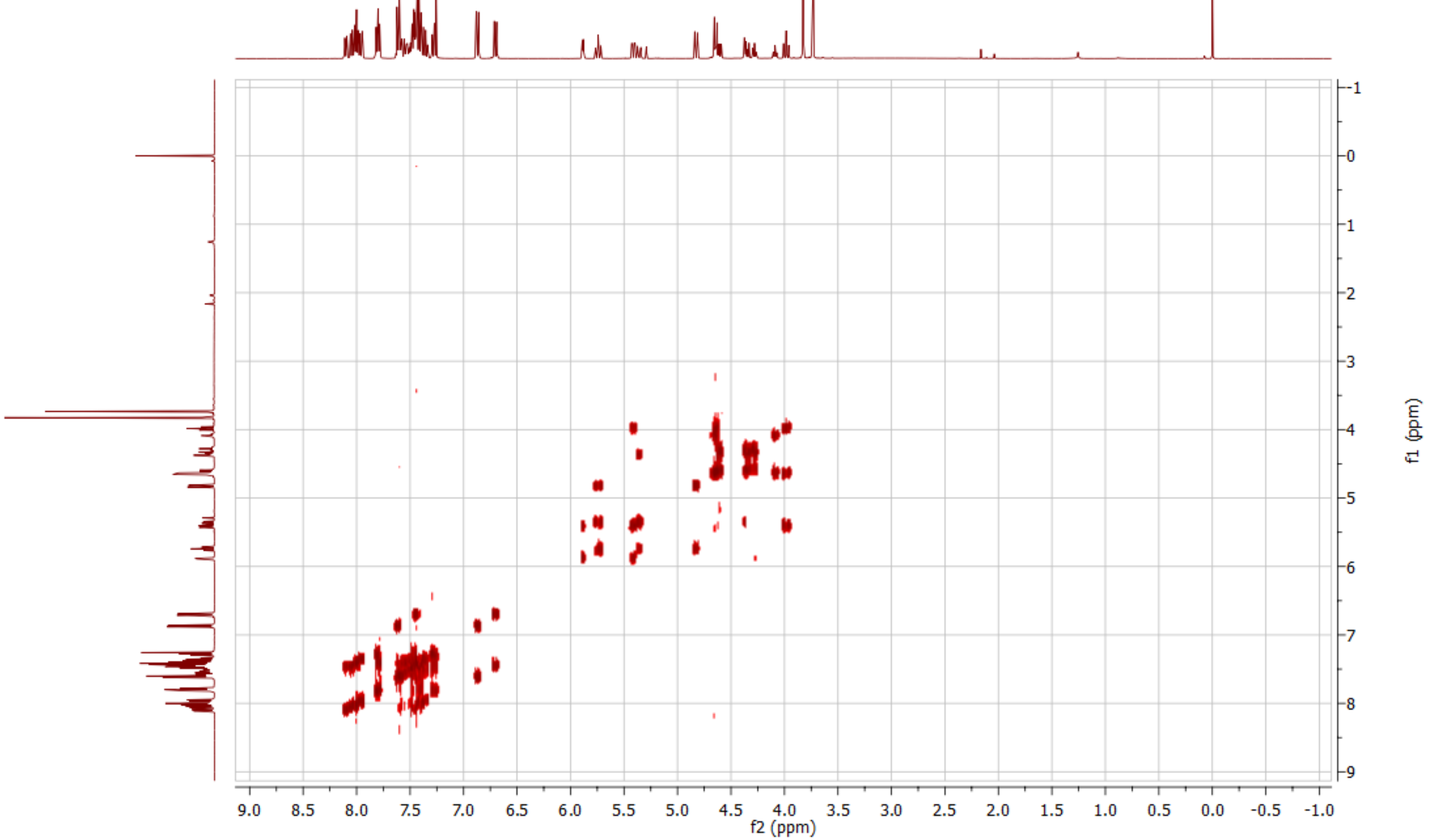

HSQC (400 × 101 MHz, CDCl₃): *p*-(Methoxy)-phenyl 3,4,6-tri-*O*-benzoyl-1-thio-β-D-galactopyranoside 64 & *p*-(methoxy)-phenyl 2,3,6-tri-*O*-benzoyl-1-thio-β-D-galactopyranoside 65

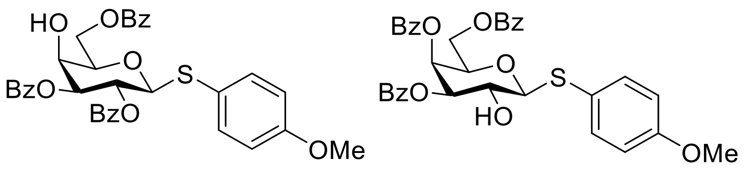

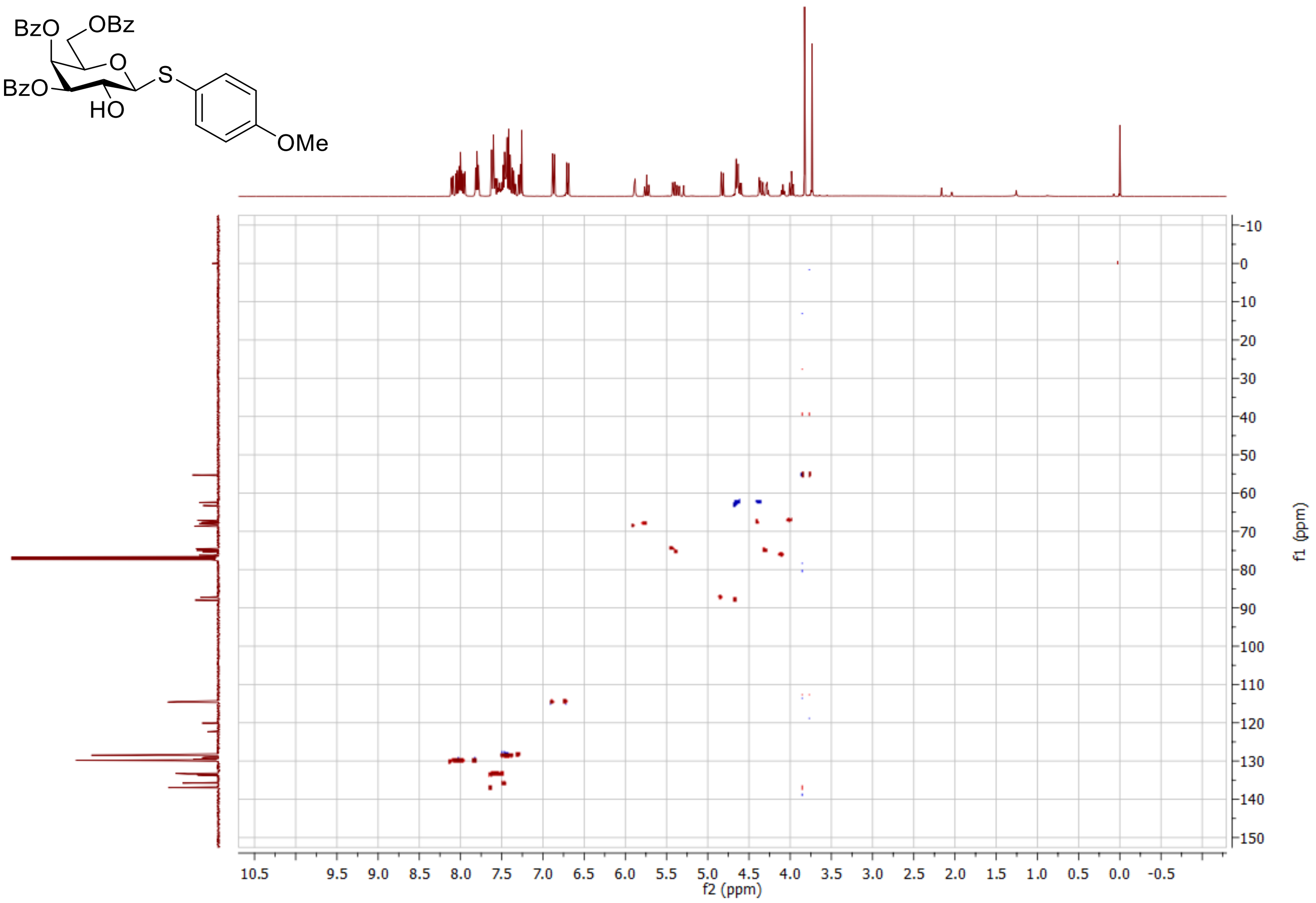

$^{13}C\{1H\}$ NMR (101 MHz, $CDCl_3$): *p*-(Methoxy)-phenyl 3,4,6-tri-*O*-benzoyl-1-thio-β-D-galactopyranoside 64 & *p*-(methoxy)-phenyl 2,3,6-tri-*O*-benzoyl-1-thio-β-D-galactopyranoside 65

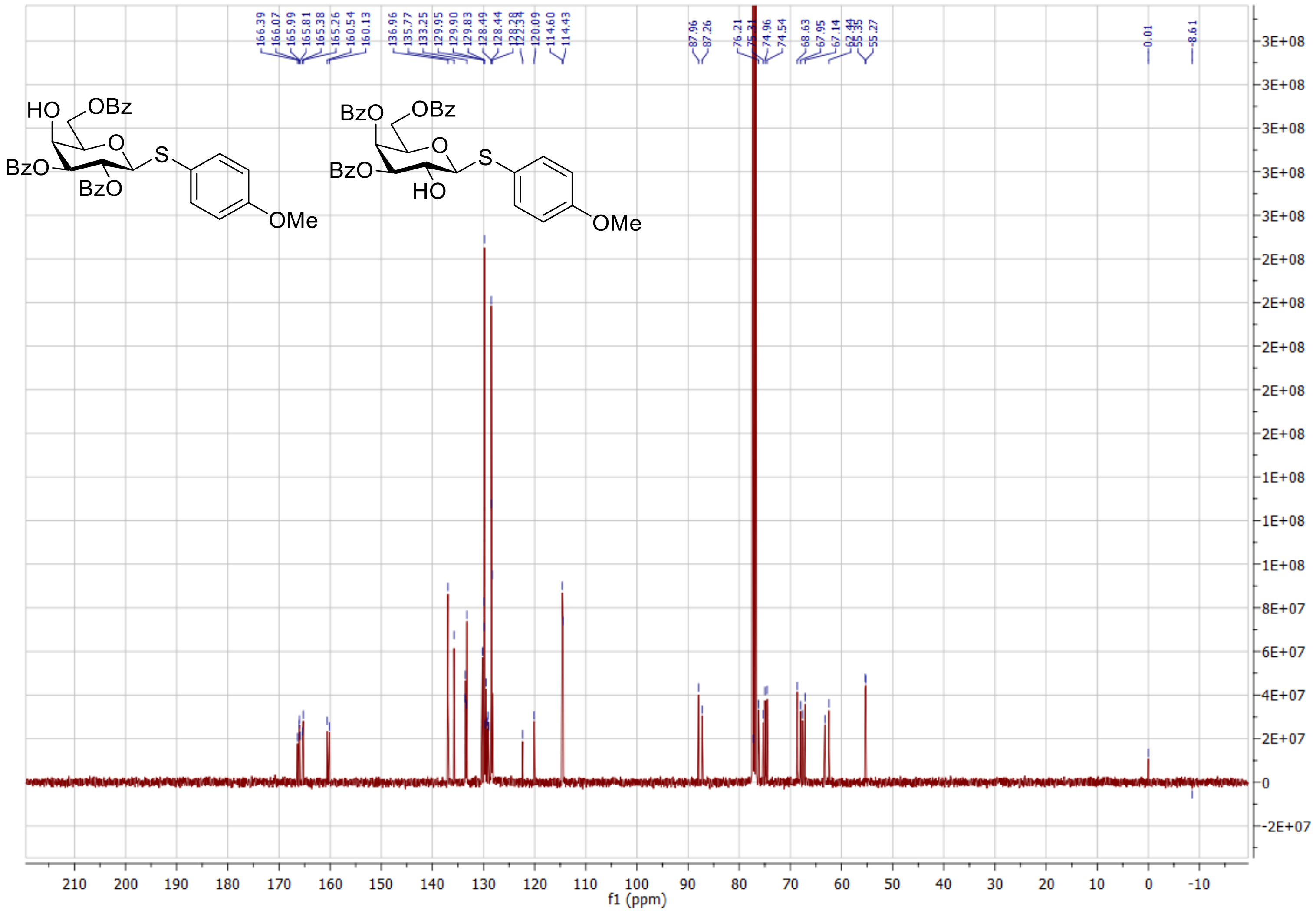

HMBC (400 × 101 MHz, CDCl3): *p*-(Methoxy)-phenyl 3,4,6-tri-*O*-benzoyl-1-thio-β-D-galactopyranoside 64 & *p*-(methoxy)-phenyl 2,3,6-tri-*O*-benzoyl-1-thio-β-D-galactopyranoside 65

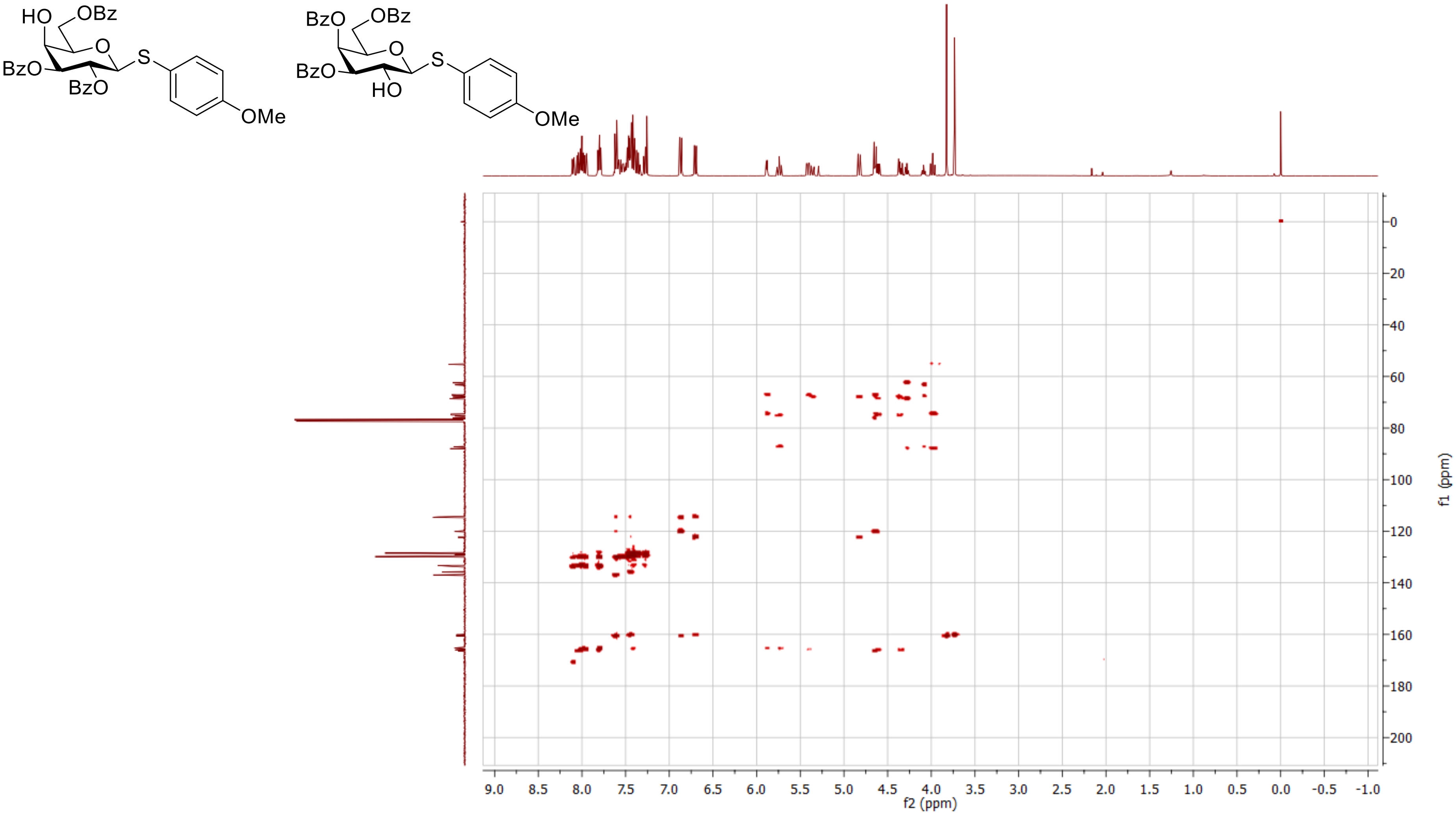

Compound 66

^{1}H NMR (400 MHz, CDCl₃): *p*-(Methoxy)-phenyl 3,6-di-*O*-benzoyl-1-thio-β-D-galactopyranoside 66

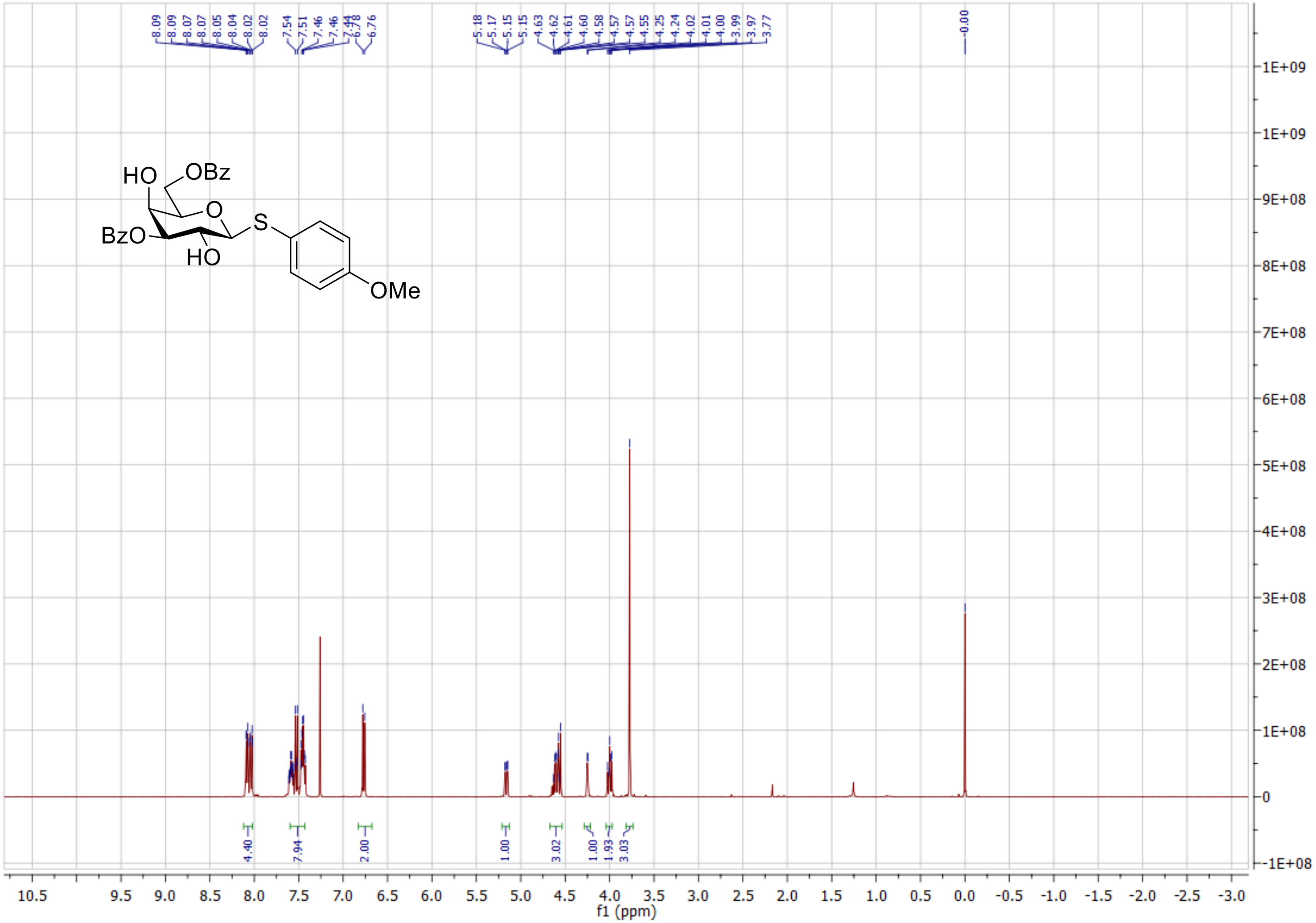

COSY (400 × 400 MHz, $CDCl_3$): *p*-(Methoxy)-phenyl 3,6-di-*O*-benzoyl-1-thio-β-D-galactopyranoside 66

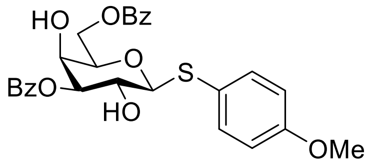

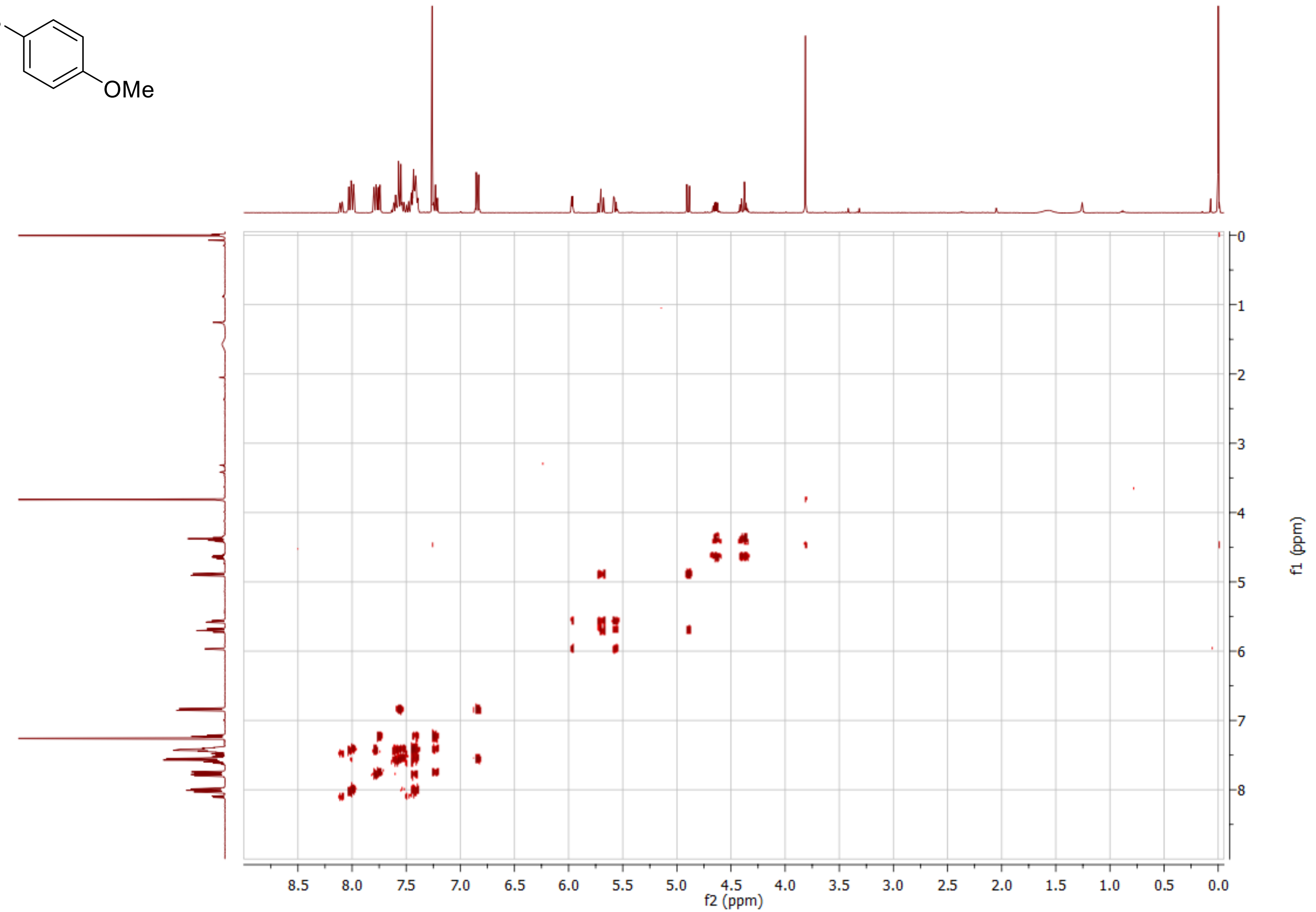

HSQC (400 × 101 MHz, $CDCl_3$): *p*-(Methoxy)-phenyl 3,6-di-*O*-benzoyl-1-thio-β-D-galactopyranoside 66

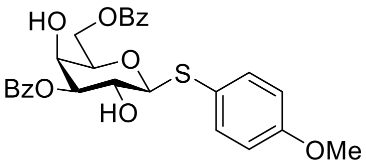

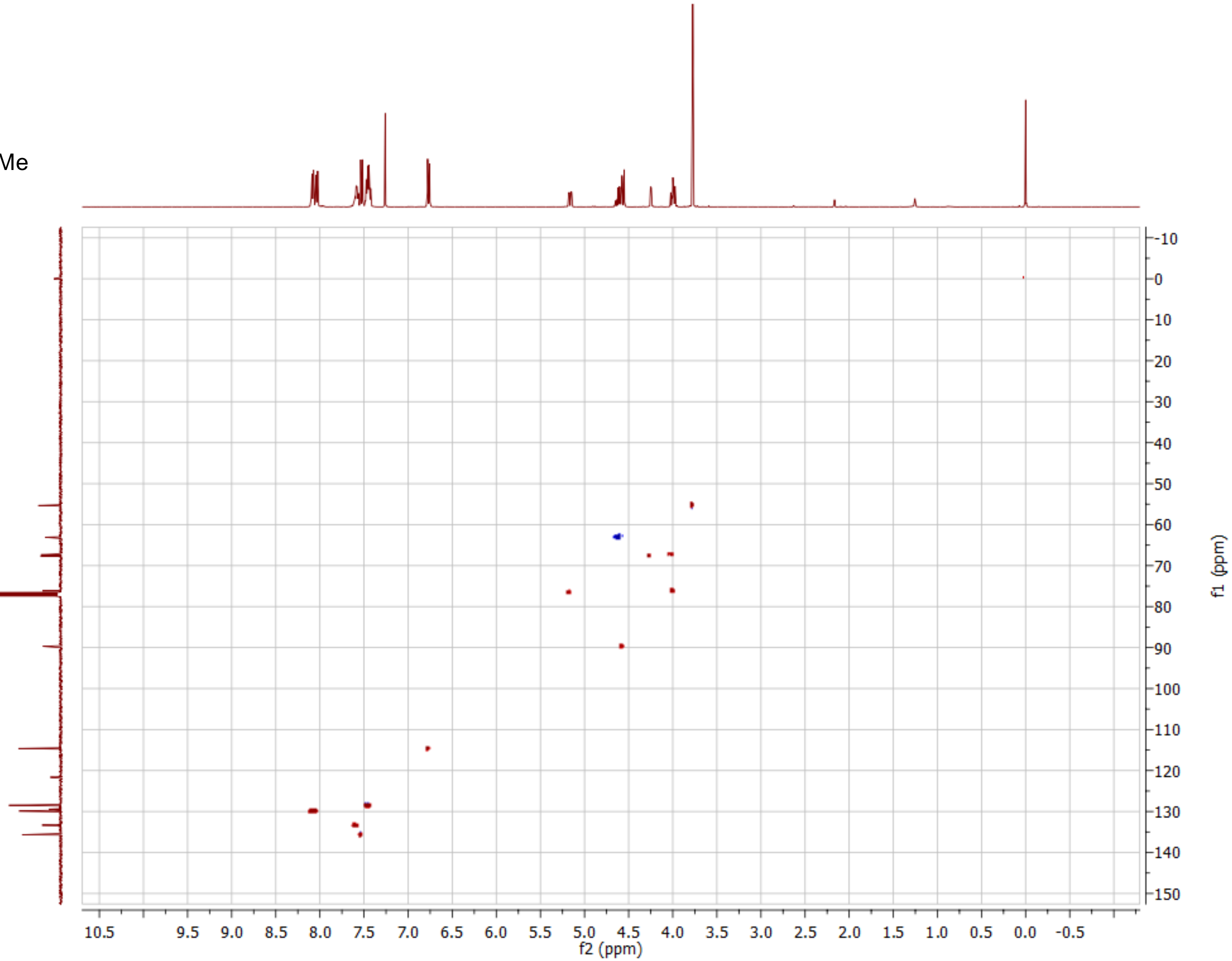

^{13}C{1H} NMR (101 MHz, CDCl$_3$): *p*-(Methoxy)-phenyl 3,6-di-*O*-benzoyl-1-thio-β-D-galactopyranoside 66

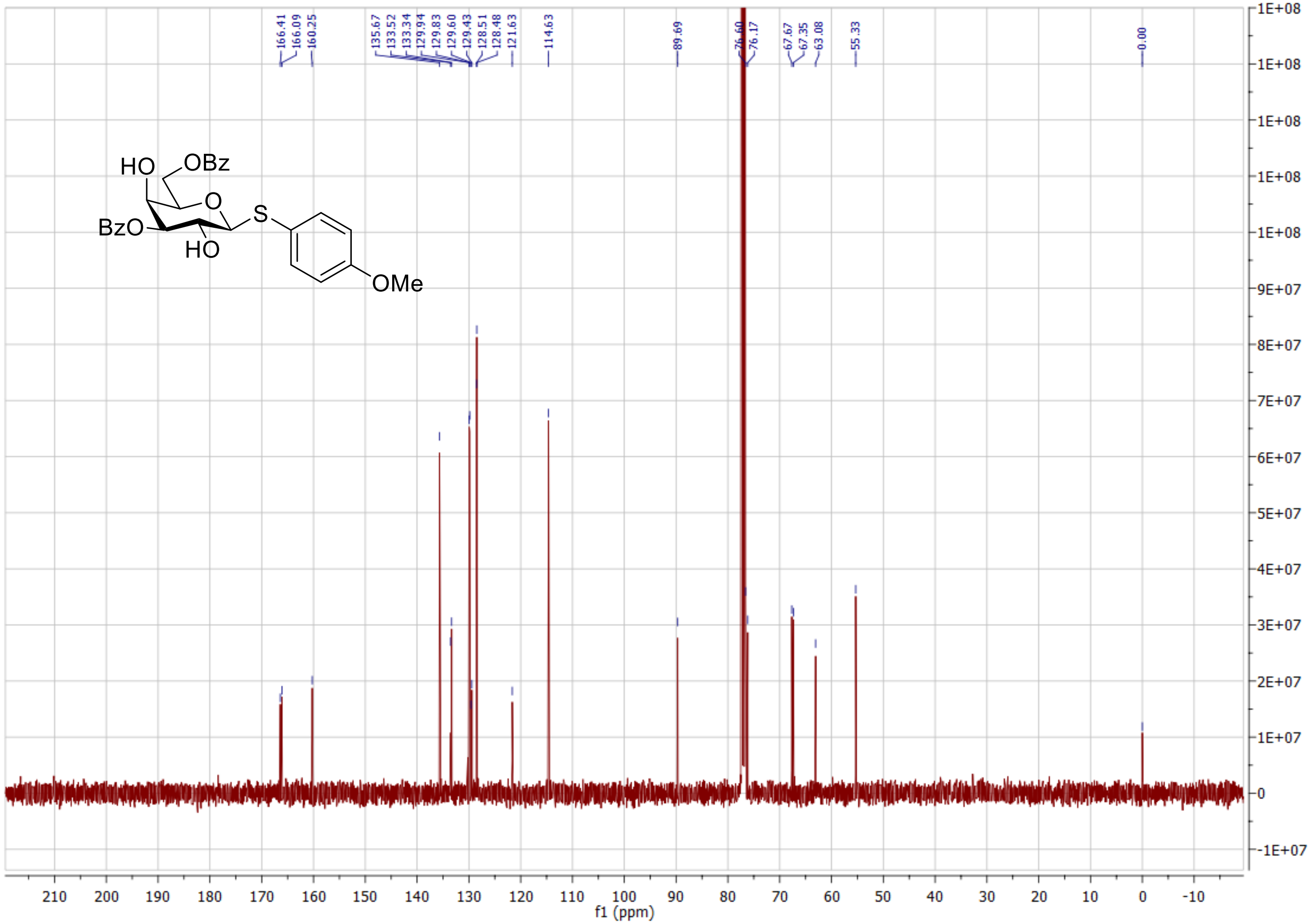

HMBC (400 × 101 MHz, CDCl3): *p*-(Methoxy)-phenyl 3,6-di-*O*-benzoyl-1-thio-β-D-galactopyranoside 66

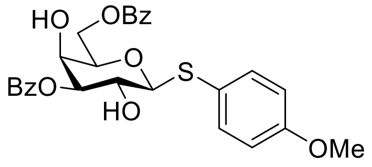

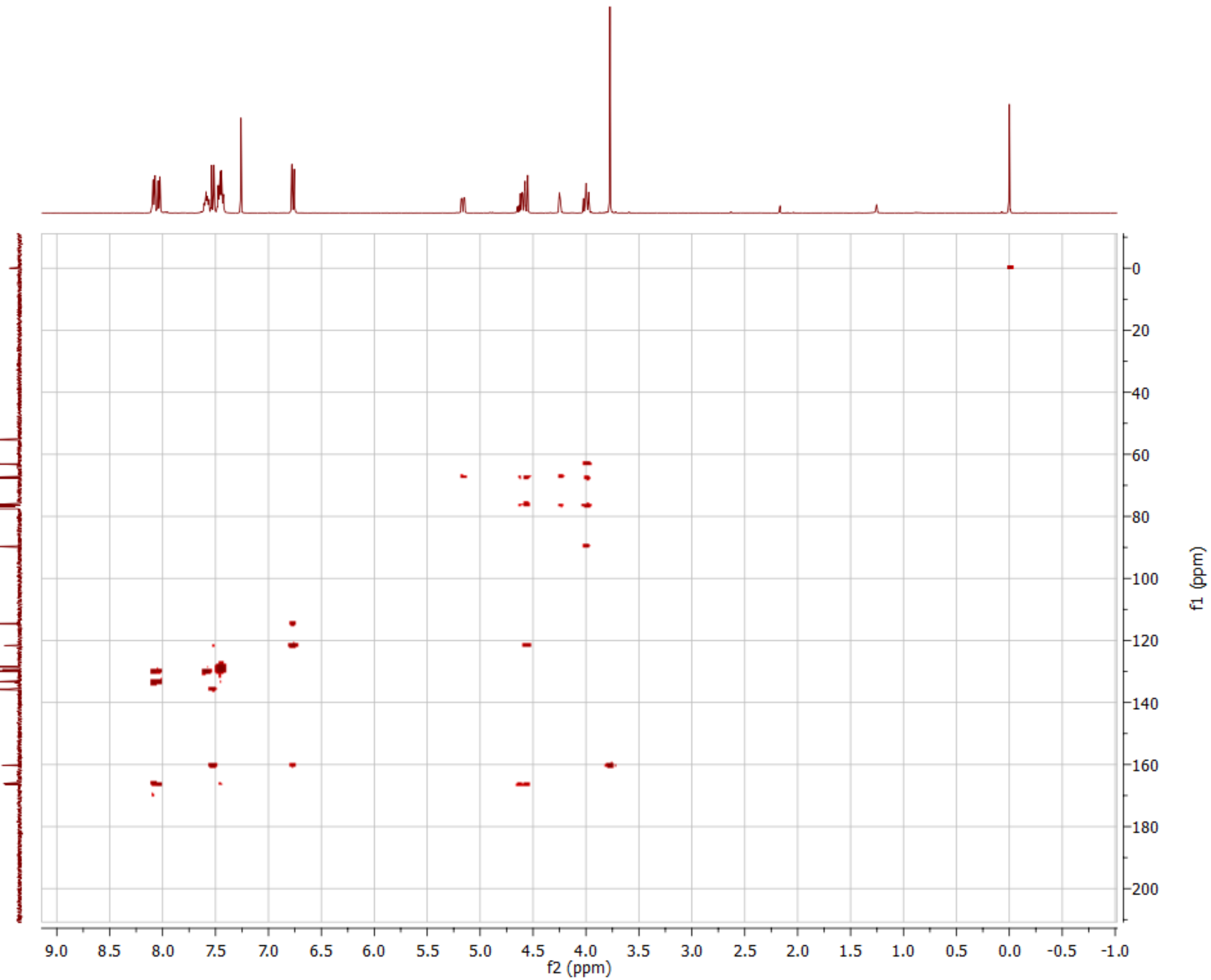

Compound 68

^{1}H NMR (400 MHz, CDCl$_3$): *p*-(Nitro)-phenyl 2,3,4,6-tetra-*O*-benzoyl-1-thio-β-D-galactopyranoside 68

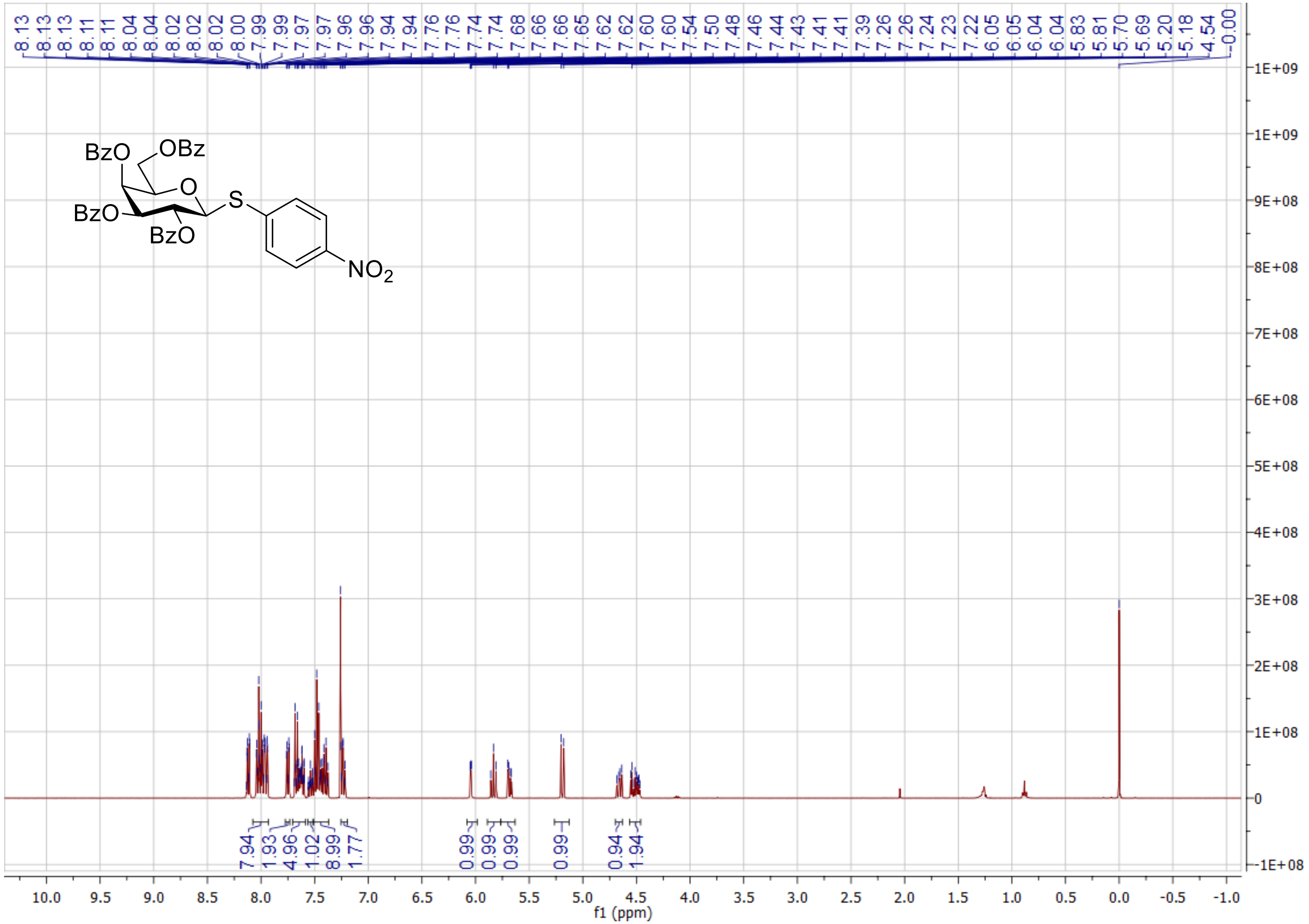

COSY (400 × 400 MHz, CDCl3): *p*-(Nitro)-phenyl 2,3,4,6-tetra-*O*-benzoyl-1-thio-β-D-galactopyranoside 68

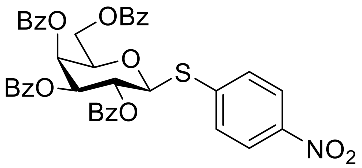

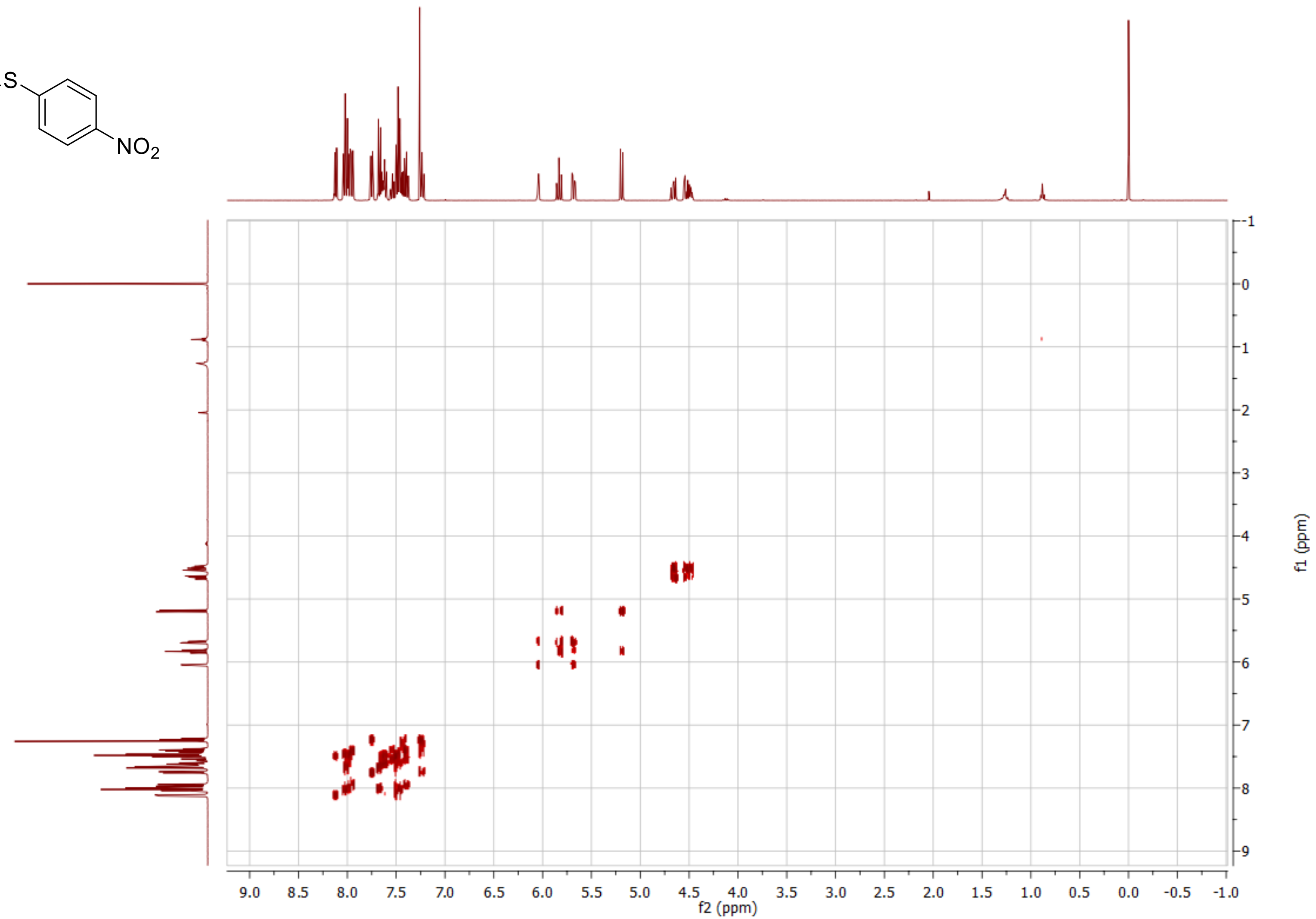

HSQC (400 × 101 MHz, CDCl₃): *p*-(Nitro)-phenyl 2,3,4,6-tetra-*O*-benzoyl-1-thio-β-D-galactopyranoside 68

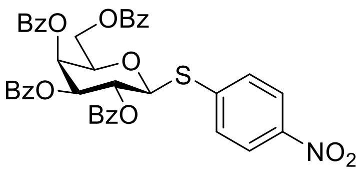

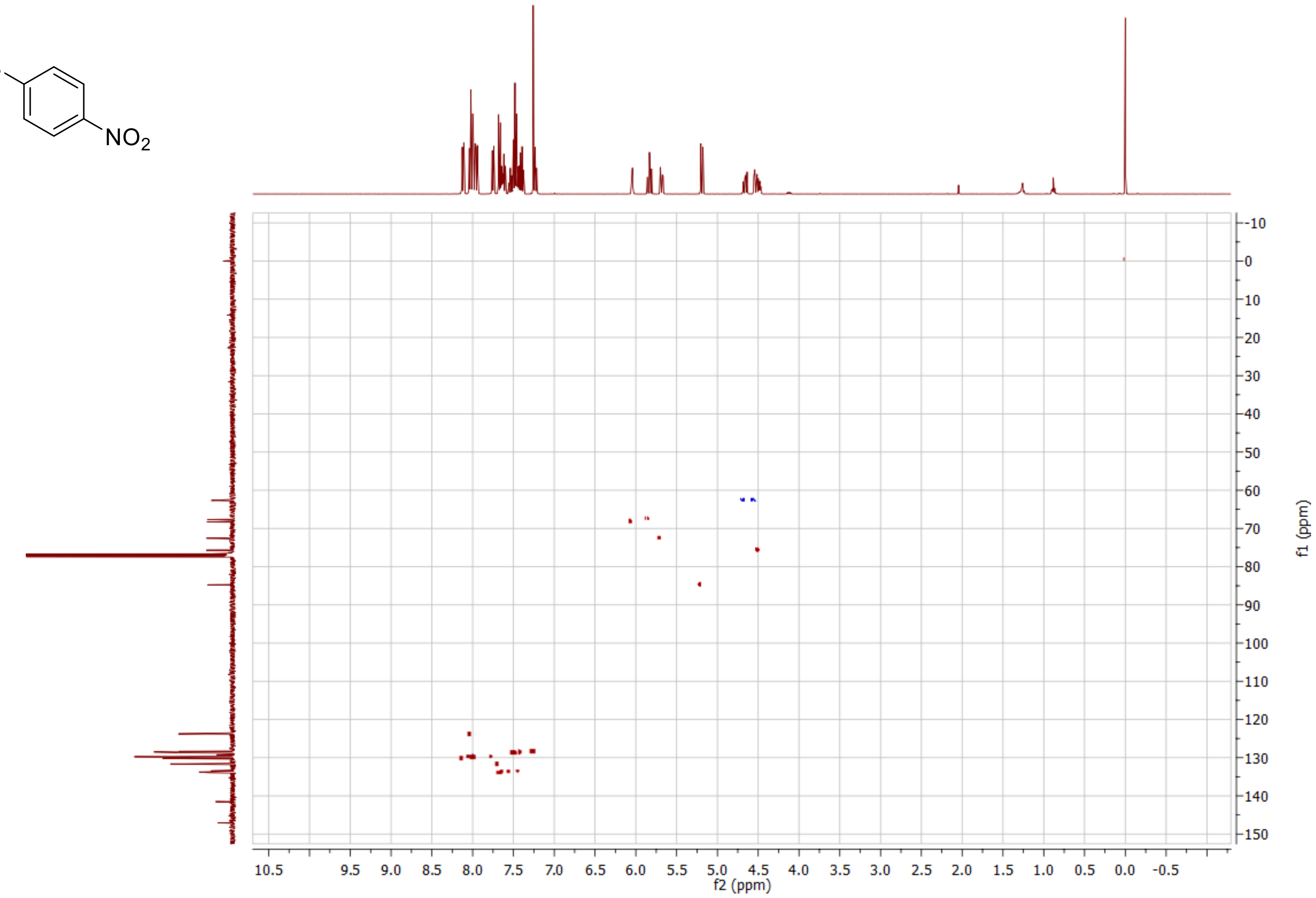

^{13}C{1H} NMR (400 × 101 MHz, CDCl₃): *p*-(Nitro)-phenyl 2,3,4,6-tetra-*O*-benzoyl-1-thio-β-D-galactopyranoside 68

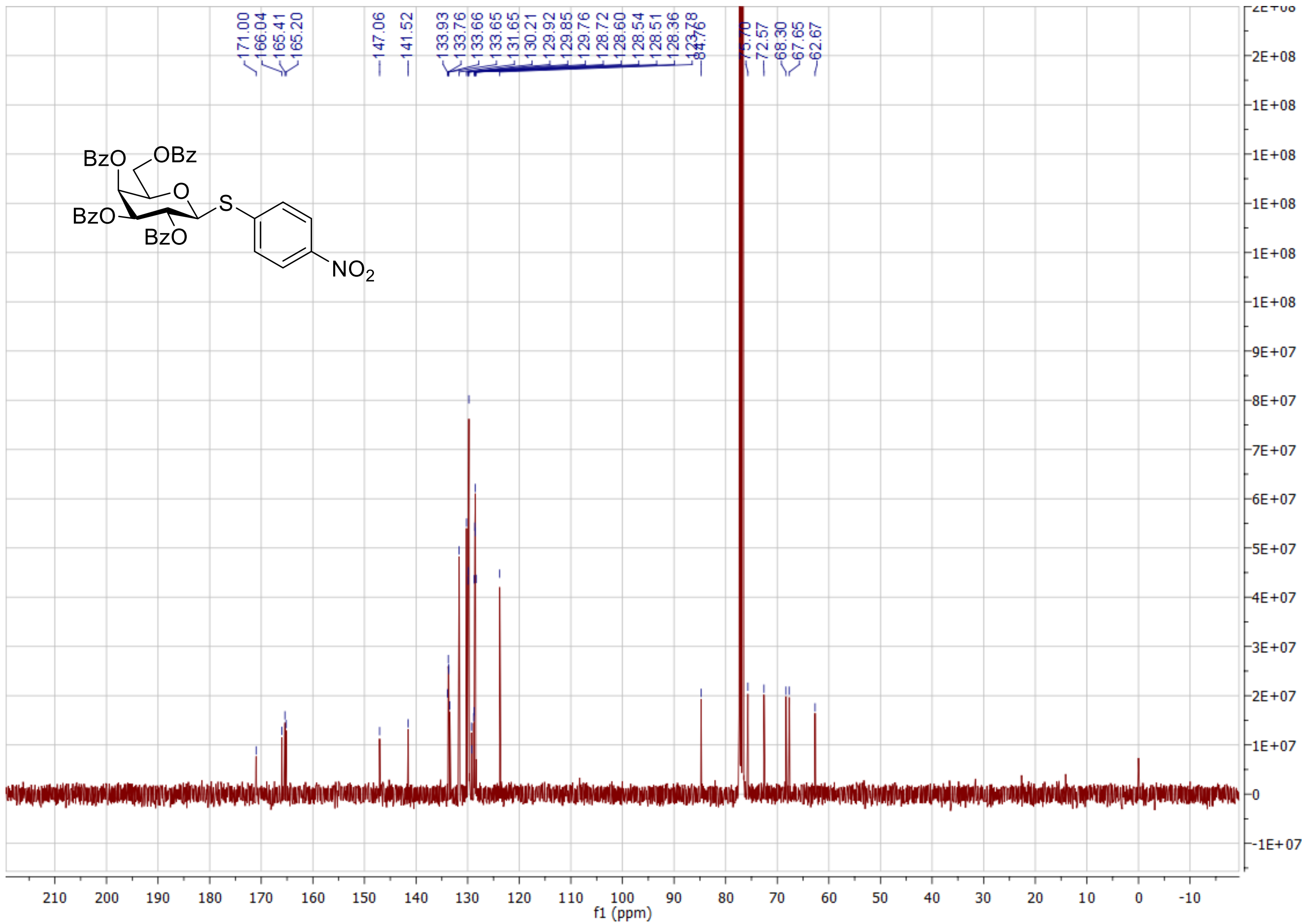

Compounds 69/70

^{1}H NMR (400 MHz, CDCl₃): *p*-(Nitro)-phenyl 2,3,6-tri-*O*-benzoyl-1-thio-β-D-galactopyranoside 70 & *p*-(nitro)-phenyl 3,4,6-tri-*O*-benzoyl-1-thio-β-D-galactopyranoside 69

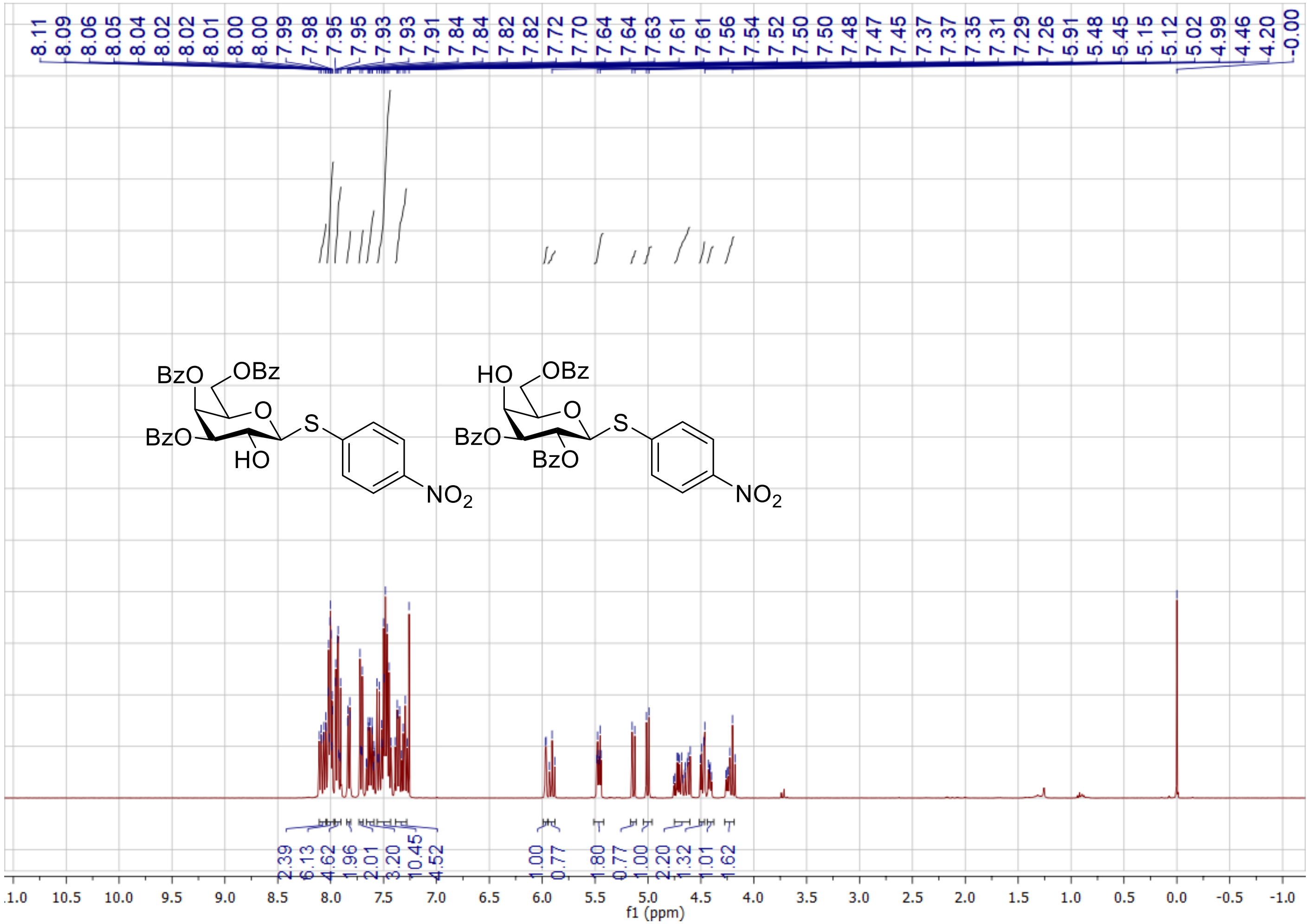

COSY (400 × 400 MHz, CDCl3): *p*-(Nitro)-phenyl 2,3,6-tri-*O*-benzoyl-1-thio-β-D-galactopyranoside 70 & *p*-(nitro)-phenyl 3,4,6-tri-*O*-benzoyl-1-thio-β-D-galactopyranoside 69

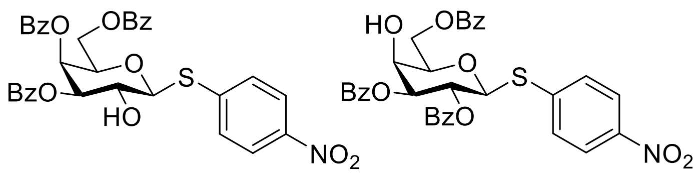

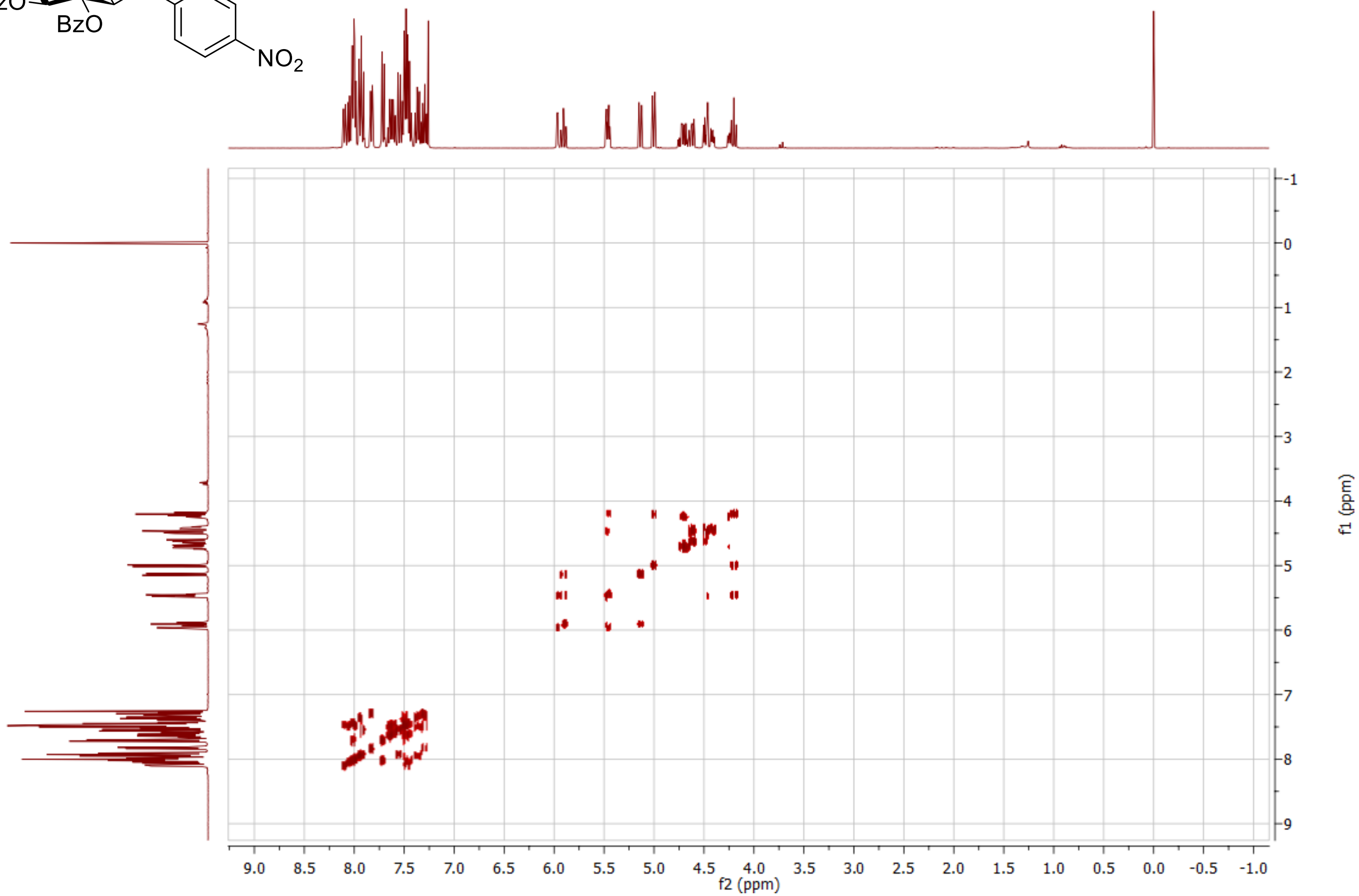

HSQC (400 × 101 MHz, CDCl3): *p*-(Nitro)-phenyl 2,3,6-tri-*O*-benzoyl-1-thio-β-D-galactopyranoside 70 & *p*-(nitro)-phenyl 3,4,6-tri-*O*-benzoyl-1-thio-β-D-galactopyranoside 69

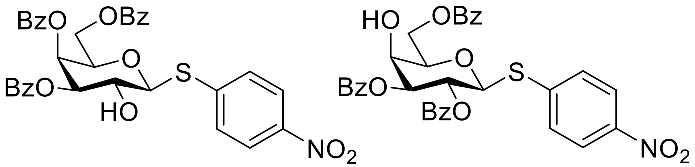

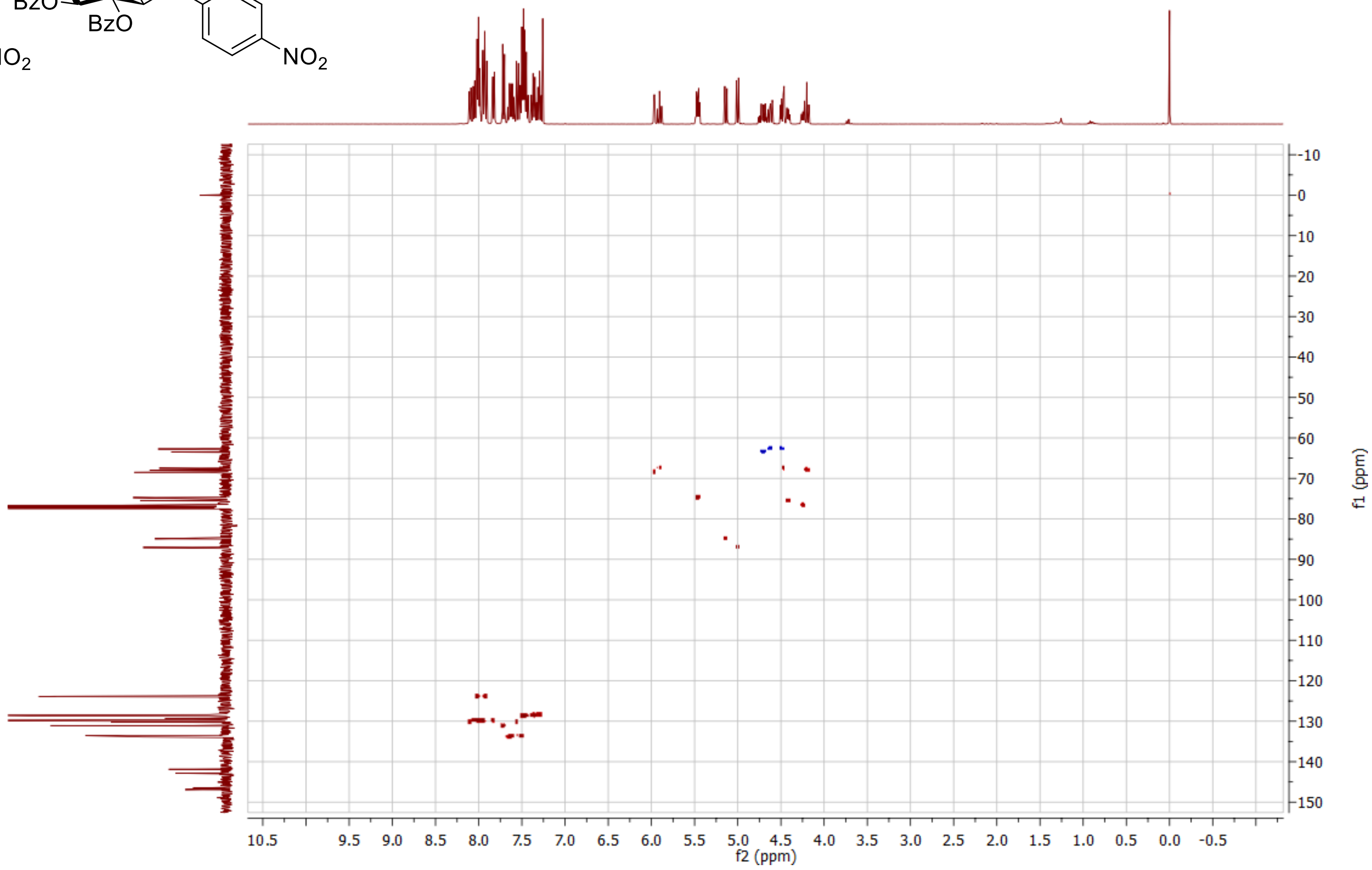

$^{13}C\{1H\}$ NMR (101 MHz, $CDCl_3$): *p*-(Nitro)-phenyl 2,3,6-tri-*O*-benzoyl-1-thio-β-D-galactopyranoside 70 & *p*-(nitro)-phenyl 3,4,6-tri-*O*-benzoyl-1-thio-β-D-galactopyranoside 69

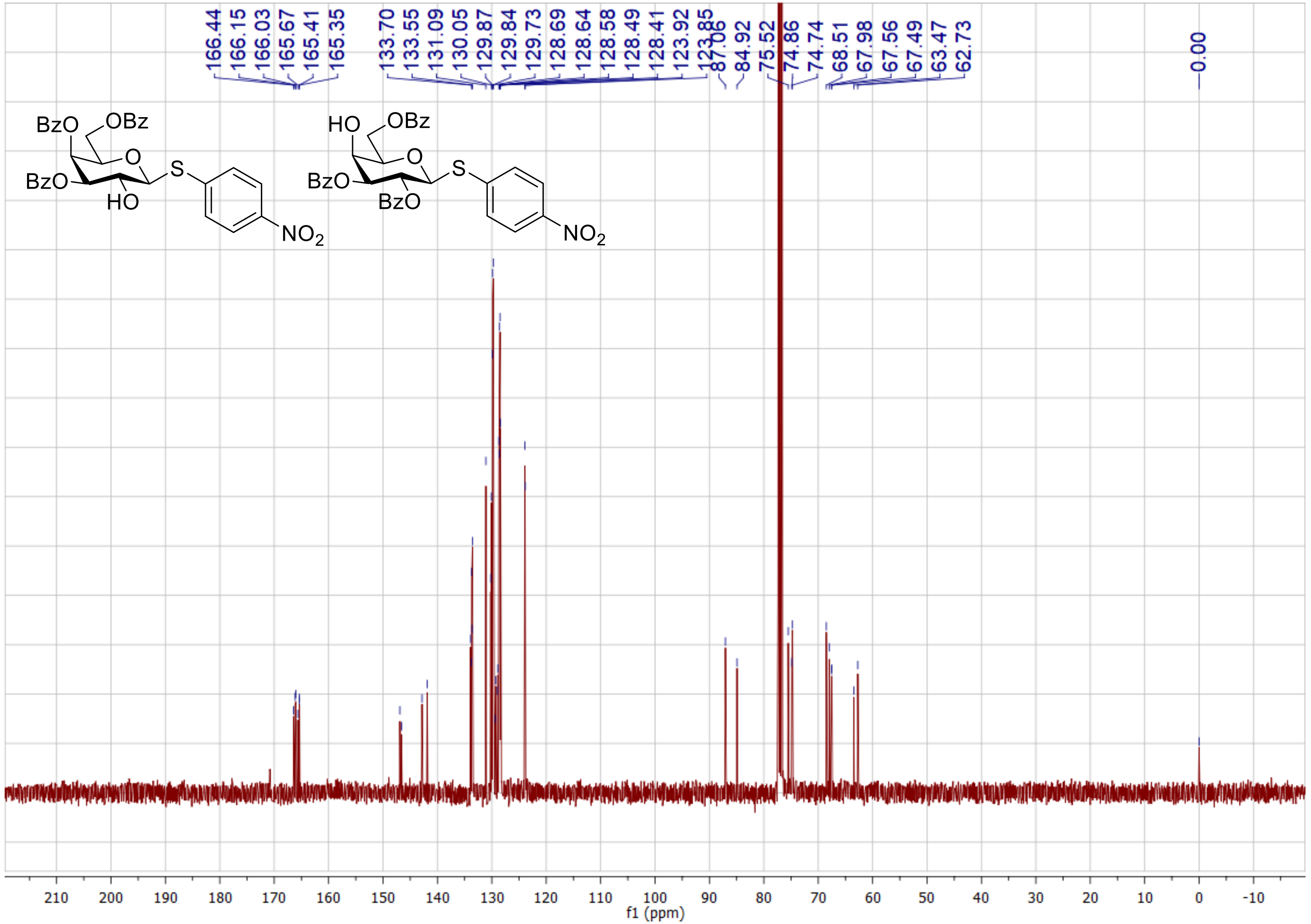

HMBC (400 × 101 MHz, CDCl₃): *p*-(Nitro)-phenyl 2,3,6-tri-*O*-benzoyl-1-thio-β-D-galactopyranoside 70 & *p*-(nitro)-phenyl 3,4,6-tri-*O*-benzoyl-1-thio-β-D-galactopyranoside 69

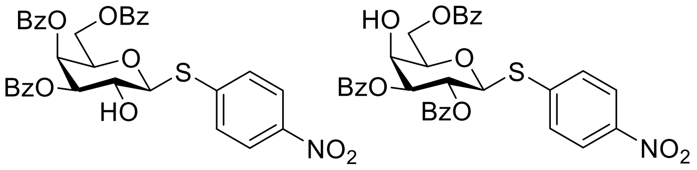

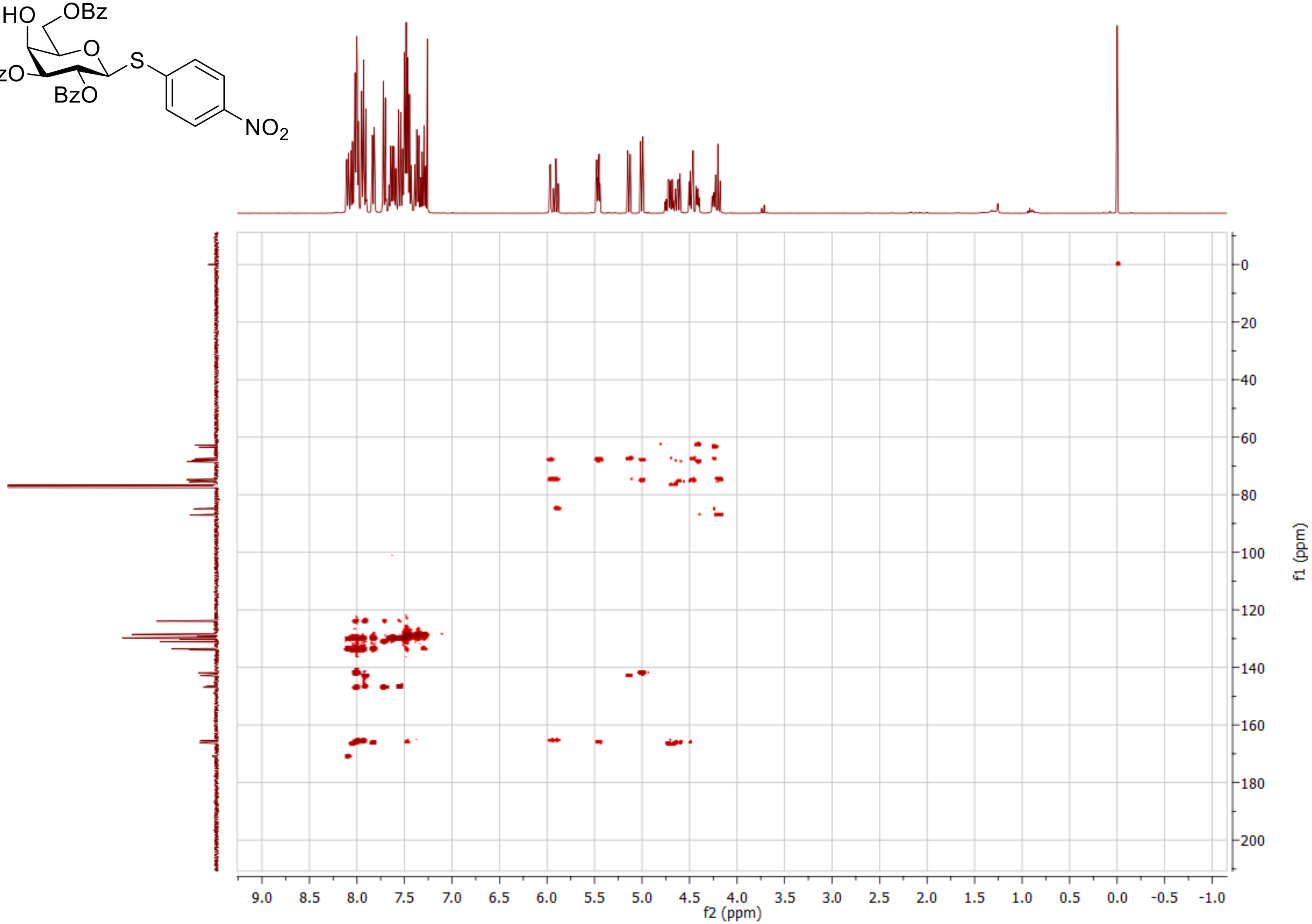

Supplement: Supplementary file 2 — jo4c01508_si_002.pdf [file jo4c01508_si_002.pdf]
